# Genome-Wide Association Study Link Novel Loci to Endometriosis

**DOI:** 10.1371/journal.pone.0058257

**Published:** 2013-03-05

**Authors:** Hans M. Albertsen, Rakesh Chettier, Pamela Farrington, Kenneth Ward

**Affiliations:** Juneau Biosciences, LLC, Salt Lake City, Utah, United States of America; National Taiwan University Hospital, Taiwan

## Abstract

Endometriosis is a common gynecological condition with complex etiology defined by the presence of endometrial glands and stroma outside the womb. Endometriosis is a common cause of both cyclic and chronic pelvic pain, reduced fertility, and reduced quality-of-life. Diagnosis and treatment of endometriosis is, on average, delayed by 7–10 years from the onset of symptoms. Absence of a timely and non-invasive diagnostic tool is presently the greatest barrier to the identification and treatment of endometriosis. Twin and family studies have documented an increased relative risk in families. To identify genetic factors that contribute to endometriosis we conducted a two-stage genome-wide association study (GWAS) of a European cohort including 2,019 surgically confirmed endometriosis cases and 14,471 controls. Three of the SNPs we identify associated at P<5×10^−8^ in our combined analysis belong to two loci: LINC00339-WNT4 on 1p36.12 (rs2235529; P = 8.65×10^−9^, OR = 1.29, CI = 1.18–1.40) and RND3-RBM43 on 2q23.3 (rs1519761; P = 4.70×10^−8^, OR = 1.20, Cl = 1.13–1.29, and rs6757804; P = 4.05×10^−8^, OR = 1.20, Cl = 1.13–1.29). Using an adjusted Bonferoni significance threshold of 4.51×10^−7^ we identify two additional loci in our meta-analysis that associate with endometriosis:, RNF144B-ID4 on 6p22.3 (rs6907340; P = 2.19×10^−7^, OR = 1.20, Cl = 1.12–1.28), and HNRNPA3P1-LOC100130539 on 10q11.21 (rs10508881; P = 4.08×10^−7^, OR = 1.19, Cl = 1.11–1.27). Consistent with previously suggested associations to WNT4 our study implicate a 150 kb region around WNT4 that also include LINC00339 and CDC42. A univariate analysis of documented infertility, age at menarche, and family history did not show allelic association with these SNP markers. Clinical data from patients in our study reveal an average delay in diagnosis of 8.4 years and confirm a strong correlation between endometriosis severity and infertility (n = 1182, P<0.001, OR = 2.18). This GWAS of endometriosis was conducted with high diagnostic certainty in cases, and with stringent handling of population substructure. Our findings broaden the understanding of the genetic factors that play a role in endometriosis.

## Introduction

Endometriosis affects 5–10% of women in their reproductive years with symptoms including pelvic pain, dyspareunia, dysmenorrhea and infertility [1]. Although ectopic endometrium has been observed in female fetuses [2], symptoms of endometriosis usually don't manifest until adolescence, and some patients with severe endometriosis remain asymptomatic. Definitive diagnosis is often delayed 7–10 years after the onset of symptoms severely impacting quality of life [3]–[5]. Family history of endometriosis has been reported in multiple studies to increased relative risk about a 5-fold [6], [7]. A large twin-study based on the Australian Twin Registry has shown that the ratio of mono-zygotic to fraternal twin pair correlations was in excess of 2 fold, suggesting that 51% of the variance of the liability to endometriosis may be attributable to additive genetic influences with minimal influence from environmental factors [8], and two smaller twin-studies report the concordance rate of endometriosis between monozygotic twins to range between 75% and 87% [9], [10]. A large number of candidate genes have been investigated for their role in endometriosis as summarized by Montgomery et al. [11] and Rahmioglu et al. [12], but the first strong evidence to date for genetic association are reported in two large Genome-Wide Association Studies (GWAS). In the first study Uno et al. [13] identified rs10965235 located in an intron of CDKN2BAS on chromosome 9p21 to be associated in a Japanese cohort, and in the second study Painter et al. [14] identified the intergenic SNP rs12700667 on chromosome 7p15.2 to be associated in a European cohort. A meta-analysis of the two studies extend this evidence and identify a total of seven loci associated with endometriosis [15]. To replicate and extend our understanding of the genetic factors that contribute to endometriosis we have undertaken a large two-stage GWAS in a European cohort.

## Results and Discussion

### GWAS and Replication

We conducted a discovery GWAS on surgically confirmed endometriosis patients and population controls using the Illumina OmniExpress BeadChip. SNPs were limited to the autosomes and SNPs with an Illumina Gentrain score ≥0.65. We further eliminated SNPs with callrate <0.98, Hardy-Weinberg Equilibrium (hwe) <0.001 and minor allele frequencies (MAF) <0.01. After filtering 580,699 SNPs remained. Next, samples with callrates <0.98 were eliminated. The remaining samples were tested for unknown familial relationships using genome-wide identity-by-state (IBS), and samples closer than 3^rd^-degree (π>0.2) were removed. We used ADMIXTURE (ver. 1.22) [16] to estimate individual ancestry proportions based on a subset of SNPs on the Illumina OmniExpress chip (see *Materials and Methods*) and restricted our analysis to samples with ≥95% European ancestry. The calculated ancestral distribution of samples within Europe is shown in Figure S1. After applying quality, relatedness and ethnicity filters 1,514 case and 12,660 control samples were used for the discovery phase of the association analysis. The genomic inflation factor lambda (λ) was determined to be 1.18, indicating measurable population stratification across the samples. To account for the elevated λ we performed a PCA adjusted association analysis that resulted in a λ-value of 1.05 shown in QQ-plots in Figure S2. We selected the top 100 SNPs with the lowest PCA-adjusted P-values (ranging between 8.20×10^−5^ and 1.36×10^−7^) for further association analysis in the replication stage (Table S1).

The replication samples included 505 cases and 1811 controls selected for the same criteria as the discovery set. The λ-value for the replication cohort was determined to be 1.01 suggesting no measurable population stratification. After applying the same SNP filters as above we analyzed the top 100 SNPs from the discovery GWAS in the replication set. A significance threshold for the study, allowing for multiple correction, was chosen at 4.51×10^−7^ (0.05/108,699; 108,699 being the number of independent SNPs in the panel of 580,699 filtered SNPs with r^2^<0.20). A meta-analysis of the discovery and replication results was performed using Cochran-Mantel-Hanzel test and revealed 8 SNPs from 4 genomic regions that passed our genome-wide significance threshold including: LINC00339-WNT4 on 1p36.12 (rs2235529; P_meta_ = 3.05×10^−9^, OR = 1.30); RND3-RBM43 on 2q23.3 (rs6757804; P_meta_ = 6.45×10^−8^, OR = 1.20), RNF144B-ID4 on 6p22.3 (rs6907340; P_meta_ = 2.19×10^−7^, OR = 1.20); and HNRNPA3P1-LOC100130539 on 10q11.21 (rs10508881; P_meta_ = 4.08×10^−7^, OR = 1.19) and shown in detail in Table 1. Table 1 also show that three SNPs (rs2235529, rs1519761 and rs6757804) pass a conventional genome wide significance threshold of P<5×10^−8^ in the combined analysis. There was no evidence of heterogeneity between the discovery and replication datasets as judged by the Breslow Day test and shown in Table 1. A second group of 15 SNPs from nine genomic regions that show suggestive replication provide additional candidate loci for endometriosis that merit further investigation. The most significant of these regions encompass IL33 on 9p24.1 (rs10975519; P_meta_ = 9.26×10^−7^, OR = 1.19). IL33 is a chemokine that has been linked to deep infiltrating endometriosis [17]. A summary of the discovery and replication GWAS, together with the meta and combined analysis for all 100 SNPs is presented in Table S1.

**Table 1:** Summary of GWAS and replication results.

SNP	gene	Chr	Pos	allele	stage	Case MAF	Control MAF	P^a^	OR^b^	95%CI	P_het_ d
rs4654783	WNT4	1	22,439,520	a/g	Discovery	0.34	0.295	2.43E−07	1.23	1.14–1.34	
					Replication	0.323	0.298	1.31E−01	1.13	0.97–1.32	
					Meta^c^			1.40E−07	1.21	1.13–1.30	0.332
					Combined Trend^e^	0.336	0.295	1.17E−07	1.21	1.13–1.29	
rs2235529	WNT4	1	22,450,487	a/g	Discovery	0.188	0.153	1.36E−07	1.28	1.16–1.41	
					Replication	0.182	0.142	1.38E−03	1.36	1.13–1.64	
					Meta^c^			3.05E−09	1.3	1.19–1.41	0.583
					Combined Trend^e^	0.186	0.151	8.65E−09	1.29	1.18–1.40	
rs1519754	RND3 | RBM43	2	151,619,693	c/a	Discovery	0.446	0.403	5.67E−05	1.19	1.11–1.30	
					Replication	0.45	0.405	9.63E−03	1.2	1.04–1.38	
					Meta^c^			1.75E−07	1.2	1.12–1.28	0.964
					Combined Trend^e^	0.447	0.403	1.15E−07	1.2	1.12–1.28	
rs6734792	RND3 | RBM43	2	151,624,882	g/a	Discovery	0.448	0.404	3.52E−05	1.2	1.11–1.29	
					Replication	0.453	0.406	7.48E−03	1.21	1.05–1.39	
					Meta^c^			8.18E−08	1.2	1.12–1.28	0.945
					Combined Trend^e^	0.449	0.404	5.19E−08	1.2	1.12–1.28	
rs1519761	RND3 | RBM43	2	151,633,204	g/a	Discovery	0.445	0.401	3.54E−05	1.2	1.11–1.29	
					Replication	0.452	0.403	5.85E−03	1.21	1.05–1.40	
					Meta^c^			7.30E−08	1.2	1.12–1.29	0.886
					Combined Trend^e^	0.447	0.401	4.70E−08	1.2	1.13–1.29	
rs6757804	RND3 | RBM43	2	151,635,832	g/a	Discovery	0.445	0.401	3.43E−05	1.2	1.11–1.29	
					Replication	0.452	0.403	5.44E−03	1.21	1.06–1.40	
					Meta^c^			6.45E−08	1.2	1.13–1.29	0.876
					Combined Trend^e^	0.446	0.4011	4.05E−08	1.2	1.13–1.29	
rs6907340	RNF144B | ID4	6	19,803,768	a/g	Discovery	0.417	0.371	5.49E−06	1.21	1.12–1.31	
					Replication	0.412	0.378	4.54E−02	1.15	1.00–1.33	
					Meta^c^			2.19E−07	1.2	1.12–1.28	0.579
					Combined Trend^e^	0.415	0.372	1.25E−07	1.2	1.12–1.28	
rs10508881	HNRNPA3P1 | LOC100130539	10	44,541,565	a/g	Discovery	0.45	0.405	3.18E−05	1.2	1.11–1.30	
					Replication	0.42	0.387	6.06E−02	1.15	1.00–1.32	
					Meta^c^			4.08E−07	1.19	1.11–1.27	0.589
					Combined Trend^e^	0.442	0.403	1.57E−06	1.18	1.10–1.26	

The discovery stage included 1,514 endometriosis cases and 12,660 population controls, and the replication stage included 505 cases and 1,811 controls.

a The P-values were determined using the Cochrane-Armitage trend test. P-values for the Discovery set reflect PCA adjusted P trend values.

b Odds-ratios (OR) and confidence intervals (CI) are calculated using the non-risk allele as the reference.

c The Meta analysis was performed using Cochran-Mantel-Haenzel statistics.

d P values of heterogeneties (P_het_) across discovery and replication stages calculated using Breslow-Day Test.

e Cochrane-Armitage trend test P-values based on the combined genotypes from the Discovery and Replication data.

Significance threshold is 4.59×10^−7^, and is determined by 0.05/108,699 where 108,699 is the number of independent SNPs in the panel with r^2^ less than 0.20.

**Figure 1:**
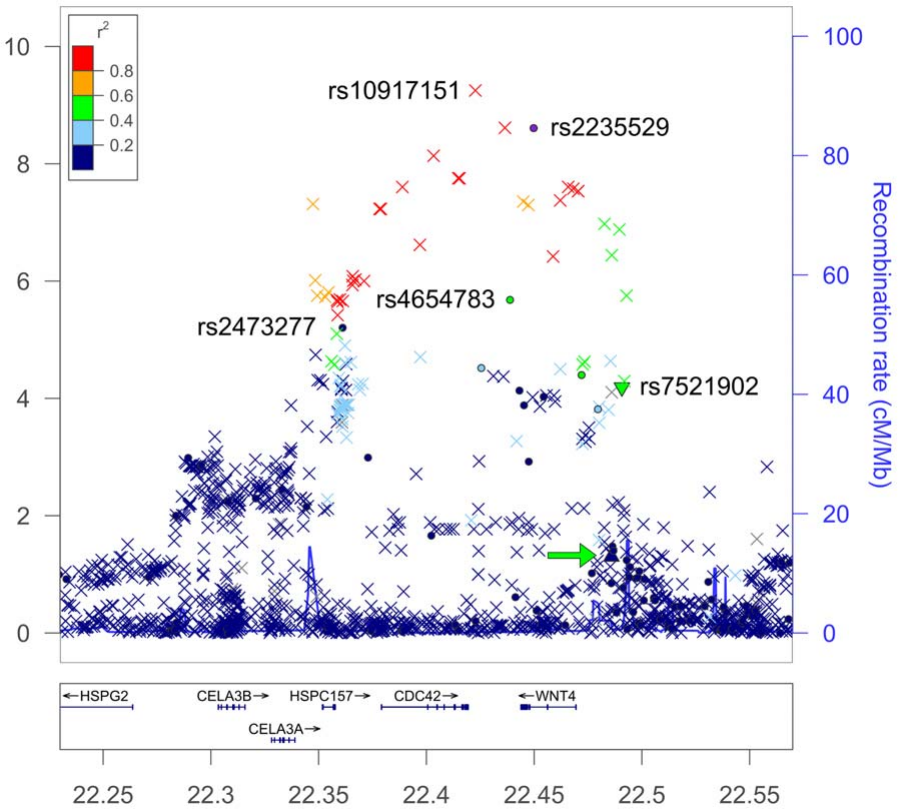
Regional association plot at the WNT4 region on chromosome 1. P-values of genotyped SNPs(•) and imputed SNPs (×) are plotted against their physical position on chromosome 1 as -log_10_(P-value) on the left (hg19/GRCh37). The plot identify a 150 kb LD-block (22.35 Mb-22.50 Mb) that show association with endometriosis and include WNT4, CDC42 and HSPC157 (gene symbol: LINC00339). Key SNPs are indicated in the Figure with their rsID. Two SNPs, rs16826658 (green arrow) and rs7521902 (green triangle), previously suggested to be associated with endometriosis (Uno et al. 2010; Painter et al. 2011), are located at the right-most boundary of the associated region. A third SNP, rs2473277, located at the left-most boundary of the LD-region was also tentatively associated by Uno et al. (2010). The genetic recombination rates estimated from 1000 Genome samples (EUR) are shown with a blue line according to the scale indicated to the right. The chromosomal position is indicated in Mb at the bottom of the figure.

To further characterize the signals from the four most strongly associated regions and the IL33 region, we performed imputation using 1000-Genome and dataset utilizing IMPUTE2 (ver. 2.2.2) [18]. The results from the imputed dataset for the WNT4 region is shown in Table S2a. Figure 1 show a regional association plot around WNT4 and reveal that the associated region extends across 150 kb and include two new genes, HSPC157 (gene symbol: LINC00339) and CDC42, in addition to WNT4. The imputation results identify 25 additional SNPs that are strongly associated to endometriosis and reveal that the imputed SNP, rs10917151, is the most strongly associated SNP in the region (P_imputed_ = 5.63×10^−10^, OR = 1.3). WNT4 is important for steroidogenesis, ovarian follicle development and the development of the female reproductive tract and a very plausible candidate for endometriosis based on its biological functions [19]. Cell division cycle 42, CDC42, is a small GTPase of the Rho-subfamily, which regulates signaling pathways that control diverse cellular functions including cell morphology, migration, endocytosis and cell cycle progression. CDC42 is in part regulated by estrogen, expressed in endometrium, and has been shown to be differentially expressed in endometriosis [20]. HSPC157 is an alias for the Long Intergenic Non-protein Coding RNA 339 (gene symbol: LINC00339). HSPC157 has been found to be differentially expressed in endometriosis versus autologous uterine endometrium [21]. Based on this biological evidence both CDC42 and LINC00339 must also be considered candidates for endometriosis.

WNT4 has previously been associated with endometriosis, first by Uno et al. (2010) who noted that rs16826658, approximately 16 kb upstream of WNT4, showed a possible association to endometriosis (P = 1.66×10^−6^, OR  = 1.20) in a Japanese population, and again by Painter et al. (2011) who reported that rs7210902, located approximately 22 kb upstream of WNT4, also showed evidence for association in a European cohort (P = 9.0×10^−5^, OR = 1.16). Our imputation analysis replicate the association of rs7210902 with endometriosis (P = 6.4×10^−5^,OR = 1.17) and confirm the involvement of the WNT4-region in the pathogenesis of endometriosis. We only found weak evidence of association with rs16826658 (P = 0.05, OR = 1.07), because the minor allele is very common and because of the different ethnic backgrounds between the studies. To further evaluate the signals from the WNT4 region, we performed a haplotype analysis of three key SNPs from our study (rs10917151, rs4654783 and rs2235529) together with rs16826658, and rs7210902 using the imputed data from our population. The haplotype-results are summarized in Table S3 and show that the risk for endometriosis is confined to a single haplotype anchored in rs10917151, rs4654783 and rs2235529 (P_HAP-1_ = 7.26E-09, OR_HAP-1_ = 1.28), and that this haplotype starts to deteriorate with the addition of rs16826658 and rs7210902 (P_HAP-1a_ = 8.33E-07, OR_HAP-1a_ = 1.25). This analysis suggests that the risk allele observed in the present study and the two previously published reports is located on the same ancestral haplotype. Imputation was also performed on the three other significant regions on chromosome 2, 6, and 10, and on the region surrounding IL33 on chromosome 9 (Table S2a–e, Figure S3).

### Overlap with Other Reported Loci

Uno et al. [13] reported association between endometriosis and rs10965235, located in intron 19 of CDKN2BAS, in a Japanese population. The protective minor allele A, that has a minor allele frequency of 0.20 in the Japanese population, is not observed in the European population preventing a direct comparison between the two ethnic groups. In lieu of a direct comparison between the two ethnic groups we scanned 66 SNPs from a region of 200 kb surrounding rs10965235 in our European population. After correcting for multiple-testing (P-value ≤0.05/66 = 0.00076) we didn't find any evidence that CDKN2BAS is associated with endometriosis in the European population (Table S4a). In contrast, a second SNP (rs13271465) located on 8p22 between MTMR7 and SLC7A2, that Uno et al. identified as being associated with endometriosis (P_Combined_ = 9.84×10^−6^, OR = 1.18), is present in both studies, show weak association (P = 0.0057, OR = 1.14) in our study, while none of the other 88 SNPs from the 200 kb region surrounding rs13271465 showed any evidence for association with endometriosis (Table S4b). Uno et al. provided a supplementary list of 100 additional candidate SNPs from their study of which 60 are present in our analysis. Among the 60 SNPs only rs2473277 has a P-value <0.001 in our study (P = 5.97×10^−6^, OR = 1.17). SNP rs2473277 is located between LINC00339 and CDC42 at the left-most boundary of the WNT4 LD-block discussed above (Figure 1), and the risk allele of rs2473277 tag perfectly with the risk haplotype HAP-1 shown in Table S3 (data not shown).

Painter et al. [14] reported significant association of moderate and severe endometriosis with rs12700667 on 7p15.2 (P_all_ = 2.6×10^−7^, OR = 1.22), with flanking support from rs7798431 located 41 kb away. The two SNPs were reported to be in strong linkage disequilibrium (LD), but unfortunately neither SNP is included in our study. A review of the Hapmap3 data from the region show that three SNPs in our study (rs12535837, rs10282436, rs10232819) are in moderate to strong LD with rs12700667 and rs7798431, but we find no evidence for association between endometriosis and any of these markers in our analyses of all endometriosis cases together nor do we find any evidence for association to the moderate and severe subset (Table S4c). A broader scan of the 200 kb region surrounding rs12700667, suggests weak association with rs4722551 (P = 0.000867) about 90 kb downstream of rs12700667 (Table S4c). Painter et al. provided a supplementary list of 73 candidate SNPs, but none of the thirty-nine SNPs present in our study reach a P-value threshold <0.001.

A recent meta-analysis published by Nyholt et al. [15] extend the findings by Uno et al. and Painter et al. and report a total of seven SNPs that pass a genome-wide significance (P<5×10^−8^). A comparison of our results to each of the seven loci show evidence for association to endometriosis for three of the seven loci as detailed in Table S7.

A recent candidate gene study investigating the *LCF6* variant (rs61764370) in the 3′-UTR of *KRAS* showed very strong association with endometriosis among 132 women (MAF_case_ = 0.311, MAF_control_ = 0.076) [22], and proposed that *let*-7 microRNA play a functional role in the development of endometriosis. We investigated this association by Taqman assay in a set of 1123 cases and 832 controls from our European population and found the allele frequencies to be identical in the two populations (MAF_case_ = 0.095, MAF_control_ = 0.093). Our result show that the *LCS6* variant of *KRAS* does not provide any value as an indicator for endometriosis risk in a European population in agreement with results reported by Luong et al. [23].

ENDO1 is a susceptibility locus on chromosome 10q26 (OMIM phenotype number 131200) identified by linkage analysis [24], but we find no evidence for SNP association (P-value <0.0001) in this 16 Mb region (data not shown).

### Clinical Stratification and Diagnostic Delay

Clinical features commonly used to characterize and stratify endometriosis include infertility, pelvic pain, severity, age-at-menarche and familiality. To determine the diagnostic delay in our patient-population we identified a group of women (n = 874) that reported both age at onset-of-symptoms (mean-age-onset = 19.04 years) and age at diagnosis (mean-age-diagnosis = 27.49), and observed an average diagnostic delay of 8.44 years, similar to previous studies. We then went on to examine if our samples showed any clinical correlations using logistic regression. The analysis revealed strong correlations between severity and infertility (P<0.001, OR = 2.19), and between severity and diagnostic delay (P<0.001, OR = 1.04) as shown in Table 2. To identify loci associated with the progression of endometriosis, we compared patients with mild endometriosis to patients with moderate or severe endometriosis in a two-stage GWAS. Stage one included 657 patients with mild endometriosis vs. 525 patients with moderate-or-severe endometriosis and a stage two replication set of 318 mild vs. 519 moderate-or-severe patients. A meta-analysis using the CMH test in this limited sample set, found no loci that pass the genome-wide significant threshold which suggest the SNPs identified in the primary study contribute to the general endometriosis risk rather than endometriosis progression. A separate analysis of the top five SNPs using logistic regression also showed no correlation with severity as shown in Table S5a, and Table S5b shows no noticeable increase in effect size when comparing moderate and severe disease against all controls.

**Table 2:** Endometriosis severity correlate with infertility and diagnostic delay.

Clinical Feature	Moderate or Severe endometriosis (n = 842)	Mild endometriosis (n = 1177)	Category	OR	Beta	SE	P
Infertility (1182)	525	657	Yes or No	2.19	0.78	0.12	8.52E-11
Family History (1881)	790	1091	Yes or No	0.89	-0.11	0.09	0.23
Age at Menarche (921)	405	516	< = 12 or >12 yrs	1.20	0.18	0.13	0.18
Diagnostic Delay (874)	383	491	0 to 35	1.04	0.04	0.01	2.18E-05

Clinical features were correlated to severity. Only patients that could be unambiguously categorized were included in the analysis with total counts provided in parenthesis next to the clinical feature. P-values (P) are calculated using Wald test. Beta is the regression coefficients and SE the standard error from logistic regression.

### Risk Analysis of Endometriosis

After removing markers with r^2^>0.8 among the top 5 associated regions (incl. the IL33 locus), we conducted multivariate logistic regression using the combined set of 2,019 cases and 14,471 controls. All of the 5 SNPs rs101917151, rs6757804, rs6907340, rs10975519 and rs10508881 analyzed remained significant with OR of 1.3, 1.2, 1.18, 1.17 and 1.17. Each marker appear to be an independent risk factors for endometriosis. Comparison of the OR between the discovery and replication datasets, shown in Table 1, does not suggest any significant inflation of effect size in the discovery dataset (winner's curse), but this conclusion remain tentative due to the difference in size between the two datasets.

### Conclusion

A two-stage GWAS and a replication study involving 2,019 cases and 14,471 controls was performed which identified four novel loci strongly associated with endometriosis and confirmed the involvement of a region around WNT4 which previously have been suggested as being associated to endometriosis. Nine other regions identified in the study also hold promise as candidate loci for endometriosis. Utmost care was taken in the clinical classification of patients and only surgically-confirmed cases with >95% European ancestry were considered in this large GWAS of endometriosis. The study is well powered (>90%) to identify a marker at or above 10% minor allele frequency (MAF) with odds-ratio (OR) >1.20, but we estimate the top 5 loci only explain about 1.5% of the phenotypic variance of endometriosis. Since the few risk loci we detected all have odds ratios <1.30 it must be assumed that any new endometriosis loci that contribute to the “missing heritability” must be rare, recent, or show minimal effect. GWAS, by design, detects only very old founder effects. When a phenotype includes infertility, like endometriosis, a high mutation rate would be required to replenish the disease-causing alleles lost from the gene-pool due to infertility. One suitable avenue to investigate under that scenario is to use whole genome sequencing of high-risk families rather than SNP-based GWAS. Little is presently known about the pathophysiology of endometriosis, but we hope that a more detailed investigation of the loci presented in this paper will help elucidate the pathogenesis of endometriosis and clarify its genetic underpinnings.

## Materials and Methods

### Ethics Statement

All subjects and controls provided written informed consent in accordance with study protocols approved by Quorum Review IRB (Seattle, WA 98101).

### Participant Recruitment

Patients included in the present study were invited to participate via an outreach program at www.endtoendo.com, where our research initiative is described in more detail. Briefly, the “End to Endo” website provides general information regarding endometriosis and our research project, and invites women diagnosed with endometriosis to participate in our study.

### Medical Review

The inclusion criteria in the endometriosis case population in the present study is surgically confirmed diagnosis of endometriosis with laparoscopy being the preferred method. Trained OB/GYN clinicians performed the medical record review and clinical assessment of each individual patient. Patients were considered to be affected if they had biopsy-proven lesions or if operative reports revealed unambiguous gross lesions. Patients were further categorized by severity, clinical history of pelvic pain, infertility, dyspareunia or dysmenorrhea and family history. Patients were grouped into one of three classes of severity: mild, moderate or severe, following the general guidelines set forth by ASRM [25]. *Exclusion* of endometriosis also requires surgical intervention and we made no attempt to exclude endometriosis in the population controls. Thus, in this analysis we are comparing cases with 100% prevalence of endometriosis to controls with the population prevalence of endometriosis (5–10%), which leads to a systematical underestimation of the true odds ratios and a decrease in statistical power to detect associations.

### DNA Extraction

Saliva samples were collected using the Oragene 300 saliva collection kit (DNA Genotek; Ottawa, Ontario, Canada) and DNA was extracted using an automated extraction instrument, AutoPure LS (Qiagen; Valencia, CA), and manufacturer's reagents and protocols. DNA quality was evaluated by calculation absorbance ratio OD_260_/OD_280_, and DNA quantification was measured using PicoGreen® (Life Technologies; Grand Island, NY).

### Microarray Genotyping

The discovery set of 1514 cases and 12660 controls and replication set of 505 cases and 1811 controls were genotyped using the Illumina Human OmniExpress Chip (Illumina; San Diego, CA) according to protocols provided by the manufacture. Figure S4 show the genotype clusters for the top eight SNPs in our study. All SNPs reported in the present study passed visual inspection for cluster quality. It is our experience that technical replication does not affect genotype calls of SNPs with high quality clusters and due to cost we could not justify independent technical replication.

### Taqman Genotyping

A Taqman® 7900 instrument (Life Technologies; Grand Island, NY) and manufacturer’s protocols were used to genotype rs61764370. Genotypes were determined using Taqman genotyping software SDS (v2.3) and the genotype cluster was visually inspected. Genotyping QC for rs61764370 passed standard criteria of call rate >95% and no deviation from HWE (p<0.001) was observed.

### Sample Quality Control

Samples were excluded from the analysis if they missed any of the following quality thresholds:

- Evidence of familial relationship closer that 3^rd^-degree (π>0.2) using genome-wide Identity-By-State (IBS) estimation implemented in PLINK
- Samples with missing genotypes >0.02
- Samples with non-European admixture >0.05 as determined by ADMIXTURE

### SNP Quality Control

SNPS were excluded from the analysis if they missed any of the following quality thresholds:

- SNPs with Illumina GenTrain Score <0.65
- SNPs from copy number variant regions or regions with adjacent SNPs
- SNPs failing Hardy-Weinberg Equilibrium (HWE) P≤10^−3^
- SNPs with minor allele frequency (MAF) ≤0.01 in the control population
- SNP call rate ≤98%

### Admixture

ADMIXTURE (ver. 1.22) was used to estimate the individual ancestry proportion [16]. The software estimates the relative admixture proportions of a given number of a priori defined ancestral groups contributing to the genome of each individual. We used the POPRES dataset [26] as a reference group to create a supervised set of 9 ancestral clusters. Seven of them belong to the European subgroups along with African and Asian groups. Since POPRES dataset utilized Affymetrix 5.0 chip, we used 105,079 autosomal SNPs that overlapped with the Illumina OmniExpress dataset. Among the 105,079 SNPs we selected a subset of 33,067 SNPs that showed greater genetic variation (absolute difference in frequency) among the 9 reference groups. The pair-wise autosomal genetic distance determined by Fixation Index (*F*_ST_) using 33,067 SNPs was calculated for the 9 reference groups and show in Table S6 [27]. Subsequently, a conditional test was used to estimate the admixture proportions in the unknown samples as described by Alexander et al. (2009).

### Principal Component Analysis (PCA)

PCA was applied to account for population stratification among the European subgroups. We selected the previously identified 33,067 SNPs to infer the axes of variation using EIGENSTRAT [28]. Only the top 10 eigenvectors were analyzed. Most of the variance among the European populations was observed in the first and second eigenvector. The first eigenvector accounts for the east-west European geographical variation while the second accounts for the north-south component. Only the top 10 eigenvectors showed population differences using Anova statistics (p<0.01). We then calculated the PCA adjusted Armitrage trend P-values using the top 10 eigenvectors as covariates.

### Power Analysis

Power calculations was performed using QUANTO (ver. 1.2), using a log-additive model. The analysis included 2019 cases and 14471 controls with the following assumptions: Type I error  = 0.05, a minor allele frequency ≥0.10 and the odds-ratio ≥1.2.

### Association Analysis

After the quality of all data was confirmed for accuracy, genetic association was determined using the whole-genome association analysis toolset, PLINK (ver. 1.07) [29].

Differences in allele frequencies between endometriosis patients and population controls were tested for each SNP by a 1-degree-of-freedom Cochran-Armitrage Trend test.

The allelic odds ratios were calculated with a confidence interval of 95%. SNPs that passed the quality control parameters were used to calculate the genomic inflation factor (λ) as well as to generate Quantile-Quantile (QQ) plots (Figure S2), which were generated by ranking a set of –log_10_ P-values and plotting them against their expected values. PCA adjusted Cochran-Armitrage trend test P-values were also determined. The combined/meta-analysis of discovery and replication dataset was performed using Cochran-Mantel-Hanszel method. Breslow Day test was used to determine between-cluster heterogeneity in the odds ratio for the disease/SNP association. Multivariate Logistic regression was used to test for independence of SNP effects. Univariate Logistic regression was used to test for correlation of clinical factors to the severity of the disease.

Control samples include both male and female samples in approximately equal proportions. The allele frequencies for the 8 strongly associated SNPs and the 15 SNPs with suggested associations did not show any significant gender bias.

Haplotype-based association tests were calculated by 1-degree of freedom χ^2^-test, along with their respective odds ratios using PLINK.

The variance explained by logistic regression model is calculated using the Cox Snell and Nagelkerke pseudo R^2^ method which is similar to the R^2^ concept of linear regression [30].

### Imputation Analysis

IMPUTE2 (ver. 2.2.2) was used for imputing SNPs against the 1000-Genome (version 3 of the Phase 1 integrated data). Samples were pre-phased with IMPUTE2 using actual genotypes and then imputed for SNPs included in the 1000-Genome reference panel to form imputed haplotypes. Imputation was carried out within +/−250 kb of the main marker of interest. Only SNPs that pass the confidence score of > = 0.9 from imputation, call rate of 0.95 and with MAF>0.01 are reported. The imputation was performed on the total dataset of 2,019 cases and 14,471 control subjects.

### Software Used

PLINK (version 1.07; http://pngu.mgh.harvard.edu/~purcell/plink/index.shtml).

QUANTO (version 1.2; http://hydra.usc.edu/gxe).

R (version 2.15.0; http://www.r-project.org/).

Impute2 (version 2.2.2; http://mathgen.stats.ox.ac.uk/impute/impute_v2.html) and haplotype analysis was performed using PLINK (ver. 1.07).

LocusZoom (version 1.1; http://csg.sph.umich.edu/locuszoom/) was used for regional association plots.

EIGENSTRAT (version 3.0; http://genepath.med.harvard.edu/~reich/Software.htm).

## Supporting Information

Figure S1 **PCA classification of Case and Control samples.** A reference set of samples previously identified as European are shown in Panel A (reference). Samples selected for being 95% European are projected onto the European map and shown in Panel B. The Figure show that our Case and Control populations are geographically identical. A preponderance of the participants have ancestral roots in the north-western part of Europe with a Southern trend towards Italy.
(PDF)

Figure S2 **Quantile-quantile plots for the Discovery set of 1,514 endometriosis cases and 12,660 population controls before and after PCA-based adjustment.** The unadjusted QQ plot in Panel A is showing a λ = 1.18. The adjusted QQ plot in Panel B show λ = 1.05. The association analysis include 580,699 SNPs and included only samples that passed our Ethnicity, SNP and Sample quality filters.
(PDF)

Figure S3 **Regional association plots for the five top regions (Panel A–E).**
(PDF)

Figure S4 **Genotype clusters for the 8 most strongly associated SNPs.**
(PDF)

Table S1 **Top 100 SNPs from Discovery and Replication GWAS.**
(PDF)

Table S2 **a Genotyped and Imputed P-values for the LINC00339-WNT4 region on 1p36.12.** b Genotyped and Imputed P-values for the RND3-RBM43 region on 2q23.3. c Genotyped and Imputed P-values for the RNF144B-ID4 region on 6p22.3. d Genotyped and Imputed P-values for the IL33-TPD52L3 region on 9p24.1. d Genotyped and Imputed P-values for the HNRNPA3P1-LOC100130539 region on 10q11.21.
(PDF)

Table S3 **Haplotype analysis for the WNT4-region.**
(PDF)

Table S4 **a No association with CDKN2BAS on 9p21 in Europeans.** b Tentative replication of rs13271465 located on 8p22 between MTMR7 and SLC7A2. c SNP rs12700667 fails replication.
(PDF)

Table S5 a Severity of endometriosis is independent of the five top loci by logistic regression analysis. b Severity of endometriosis is independent of the top five loci by association analysis.
(PDF)

Table S6 **Pair-wise autosomal genetic distance among ethnic groups as measured by the Fixation Index (** ***F*** **_ST_).**
(PDF)

Table S7 **Support for endometriosis association in a Caucasian cohort found at 3 of 7 loci reported by Nyholt et al.**
(PDF)

## References

[1] Giudice LC, Kao LC (2004) Endometriosis. Lancet 364: 1789–1799. 15541453 10.1016/S0140-6736(04)17403-5

[2] Signorile PG, Baldi F, Bussani R, D'Armiento M, De Falco M, et al (2009) Ectopic endometrium in human foetuses is a common event and sustains the theory of mullerianosis in the pathogenesis of endometriosis, a disease that predisposes to cancer. J Exp Clin Cancer Res 28: 49. 19358700 10.1186/1756-9966-28-49 PMC2671494

[3] Husby GK, Haugen RS, Moen MH (2003) Diagnostic delay in women with pain and endometriosis. Acta Obstet Gynecol Scand 82: 649–653. 12790847 10.1034/j.1600-0412.2003.00168.x

[4] Ballard K, Lowton K, Wright J (2006) What's the delay? A qualitative study of women's experiences of reaching a diagnosis of endometriosis. Fertil Steril 86: 1296–1301. 17070183 10.1016/j.fertnstert.2006.04.054

[5] Arruda MS, Petta CA, Abrao MS, Benetti-Pinto CL (2003) Time elapsed from onset of symptoms to diagnosis of endometriosis in a cohort study of Brazilian women. Hum Reprod 18: 756–759. 12660267 10.1093/humrep/deg136

[6] Hansen KA, Eyster KM (2010) Genetics and genomics of endometriosis. Clin Obstet Gynecol 53: 403–412. 20436317 10.1097/GRF.0b013e3181db7ca1 PMC4346178

[7] Stefansson H, Geirsson RT, Steinthorsdottir V, Jonsson H, Manolescu A, et al (2002) Genetic factors contribute to the risk of developing endometriosis. Hum Reprod 17: 555–559. 11870102 10.1093/humrep/17.3.555

[8] Treloar SA, O'Connor DT, O'Connor VM, Martin NG (1999) Genetic influences on endometriosis in an Australian twin sample. Fertil Steril 71: 701–710. 10202882 10.1016/s0015-0282(98)00540-8

[9] Hadfield RM, Mardon HJ, Barlow DH, Kennedy SH (1997) Endometriosis in monozygotic twins. Fertil Steril 68: 941–942. 9389831 10.1016/s0015-0282(97)00359-2

[10] Moen MH (1994) Endometriosis in monozygotic twins. Acta Obstet Gynecol Scand 73: 59–62. 8304029 10.3109/00016349409013396

[11] Montgomery GW, Nyholt DR, Zhao ZZ, Treloar SA, Painter JN, et al (2008) The search for genes contributing to endometriosis risk. Hum Reprod Update 14: 447–457. 18535005 10.1093/humupd/dmn016 PMC2574950

[12] Rahmioglu N, Missmer SA, Montgomery GW, Zondervan KT (2012) Insights into Assessing the Genetics of Endometriosis. Curr Obstet Gynecol Rep 1: 124–137. 22924156 10.1007/s13669-012-0016-5 PMC3410033

[13] Uno S, Zembutsu H, Hirasawa A, Takahashi A, Kubo M, et al (2010) A genome-wide association study identifies genetic variants in the CDKN2BAS locus associated with endometriosis in Japanese. Nat Genet 42: 707–710. 20601957 10.1038/ng.612

[14] Painter JN, Anderson CA, Nyholt DR, Macgregor S, Lin J, et al (2011) Genome-wide association study identifies a locus at 7p15.2 associated with endometriosis. Nat Genet 43: 51–54. 21151130 10.1038/ng.731 PMC3019124

[15] Nyholt DR, Low SK, Anderson CA, Painter JN, Uno S, et al.. (2012) Genome-wide association meta-analysis identifies new endometriosis risk loci. Nat Genet. 10.1038/ng.2445 PMC3527416 23104006

[16] Alexander DH, Novembre J, Lange K (2009) Fast model-based estimation of ancestry in unrelated individuals. Genome Res 19: 1655–1664. 19648217 10.1101/gr.094052.109 PMC2752134

[17] Santulli P, Borghese B, Chouzenoux S, Vaiman D, Borderie D, et al (2012) Serum and peritoneal interleukin-33 levels are elevated in deeply infiltrating endometriosis. Hum Reprod 27: 2001–2009. 22587998 10.1093/humrep/des154

[18] Howie BN, Donnelly P, Marchini J (2009) A flexible and accurate genotype imputation method for the next generation of genome-wide association studies. PLoS Genet 5: e1000529. 19543373 10.1371/journal.pgen.1000529 PMC2689936

[19] Franco HL, Dai D, Lee KY, Rubel CA, Roop D, et al (2011) WNT4 is a key regulator of normal postnatal uterine development and progesterone signaling during embryo implantation and decidualization in the mouse. FASEB J 25: 1176–1187. 21163860 10.1096/fj.10-175349 PMC3058697

[20] Goteri G, Ciavattini A, Lucarini G, Montik N, Filosa A, et al (2006) Expression of motility-related molecule Cdc42 in endometrial tissue in women with adenomyosis and ovarian endometriomata. Fertil Steril 86: 559–565. 16854417 10.1016/j.fertnstert.2006.01.031

[21] Hu WP, Tay SK, Zhao Y (2006) Endometriosis-specific genes identified by real-time reverse transcription-polymerase chain reaction expression profiling of endometriosis versus autologous uterine endometrium. J Clin Endocrinol Metab 91: 228–238. 16249290 10.1210/jc.2004-1594

[22] Grechukhina O, Petracco R, Popkhadze S, Massasa E, Paranjape T, et al.. (2012) A polymorphism in a let-7 microRNA binding site of KRAS in women with endometriosis. EMBO Mol Med. 10.1002/emmm.201100200 PMC3376847 22307873

[23] Luong HT, Nyholt DR, Painter JN, Chapman B, Kennedy S, et al.. (2012) No evidence for genetic association with the let-7 microRNA-binding site or other common KRAS variants in risk of endometriosis. Hum Reprod. 10.1093/humrep/des329 PMC3501245 23010532

[24] Treloar SA, Wicks J, Nyholt DR, Montgomery GW, Bahlo M, et al (2005) Genomewide linkage study in 1,176 affected sister pair families identifies a significant susceptibility locus for endometriosis on chromosome 10q26. Am J Hum Genet 77: 365–376. 16080113 10.1086/432960 PMC1226203

[25] American Society for Reproductive Medicine (1997) Revised American Society for Reproductive Medicine classification of endometriosis: 1996. Fertil Steril 67: 817–821. 9130884 10.1016/s0015-0282(97)81391-x

[26] Nelson MR, Bryc K, King KS, Indap A, Boyko AR, et al (2008) The Population Reference Sample, POPRES: a resource for population, disease, and pharmacological genetics research. Am J Hum Genet 83: 347–358. 18760391 10.1016/j.ajhg.2008.08.005 PMC2556436

[27] Holsinger KE, Weir BS (2009) Genetics in geographically structured populations: defining, estimating and interpreting F(ST). Nat Rev Genet 10: 639–650. 19687804 10.1038/nrg2611 PMC4687486

[28] Price AL, Patterson NJ, Plenge RM, Weinblatt ME, Shadick NA, et al (2006) Principal components analysis corrects for stratification in genome-wide association studies. Nature Genetics 38: 904–909. 16862161 10.1038/ng1847

[29] Purcell S, Neale B, Todd-Brown K, Thomas L, Ferreira MA, et al (2007) PLINK: a tool set for whole-genome association and population-based linkage analyses. Am J Hum Genet 81: 559–575. 17701901 10.1086/519795 PMC1950838

[30] Nagelkerke NJD (1991) A note on a general definition of the coefficient of determination. Biometrika 78: 691–692.

