# Supplementary material for: Genome-Wide Association Study Link Novel Loci to Endometriosis
Source: PLoS One. 2013 Mar 5;8(3):e58257. doi: 10.1371/journal.pone.0058257 (PMC3589333; doi:10.1371/journal.pone.0058257)
Supplement: Figure S1 — PCA classification of Case and Control samples. A reference set of samples previously identified as European are shown in Panel A (reference). Samples selected for being 95% European are projected onto the European map and shown in Panel B. The Figure show that our Case and Control populations are geographically identical. A preponderance of the participants have ancestral roots in the north-western part of Europe with a Southern trend towards Italy. (PDF) [file pone.0058257.s001.pdf]

Panel A

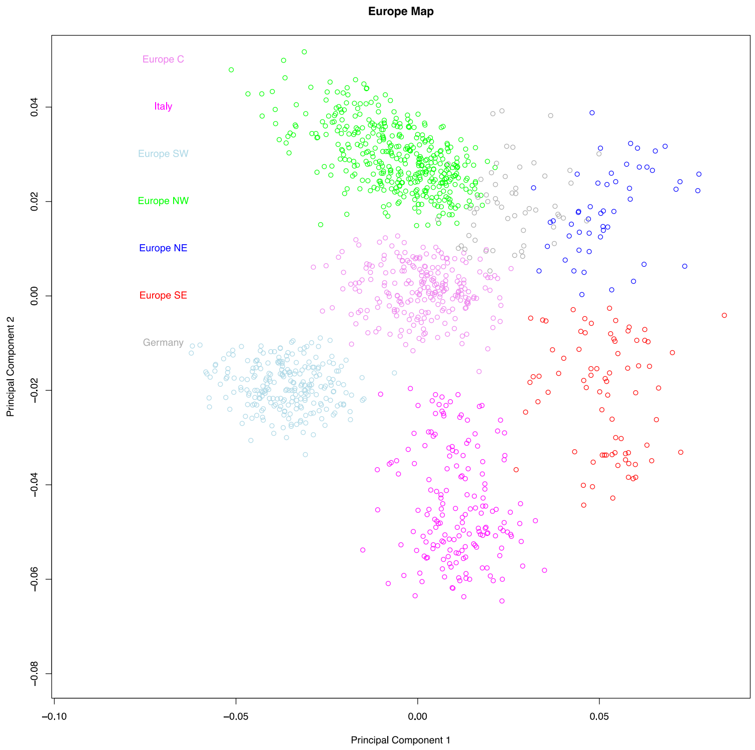

Panel B

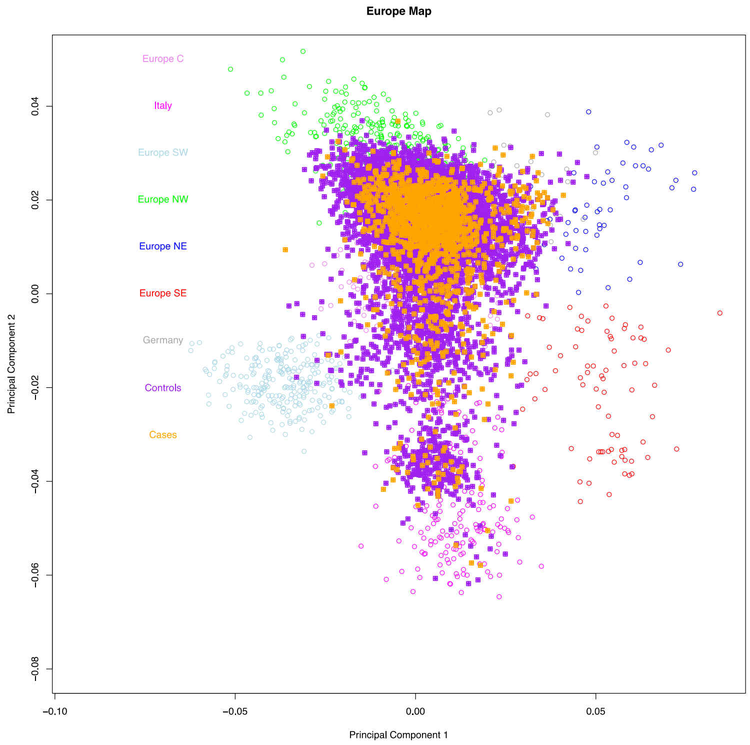

Figure S1 PCA plot of samples with greater than 95% Caucasian ancestry component mapped against samples of known European origin. Panel A show a set of POPRES samples of known geographic origin within Europe (Nelson et al. 2008). Panel B show the Case samples (orange dots) and Control samples (purple dots) from the present study mapped into the same space identified in Panel A. The preponderance of both Case and Control samples locate to Northern Europe, with a trend and secondary cluster in Northern Italy.
