# Supplementary material for: Genome-Wide Association Study Link Novel Loci to Endometriosis
Source: PLoS One. 2013 Mar 5;8(3):e58257. doi: 10.1371/journal.pone.0058257 (PMC3589333; doi:10.1371/journal.pone.0058257)
Supplement: Figure S2 — Quantile-quantile plots for the Discovery set of 1,514 endometriosis cases and 12,660 population controls before and after PCA-based adjustment. The unadjusted QQ plot in Panel A is showing a λ = 1.18. The adjusted QQ plot in Panel B show λ = 1.05. The association analysis include 580,699 SNPs and included only samples that passed our Ethnicity, SNP and Sample quality filters. (PDF) [file pone.0058257.s002.pdf]

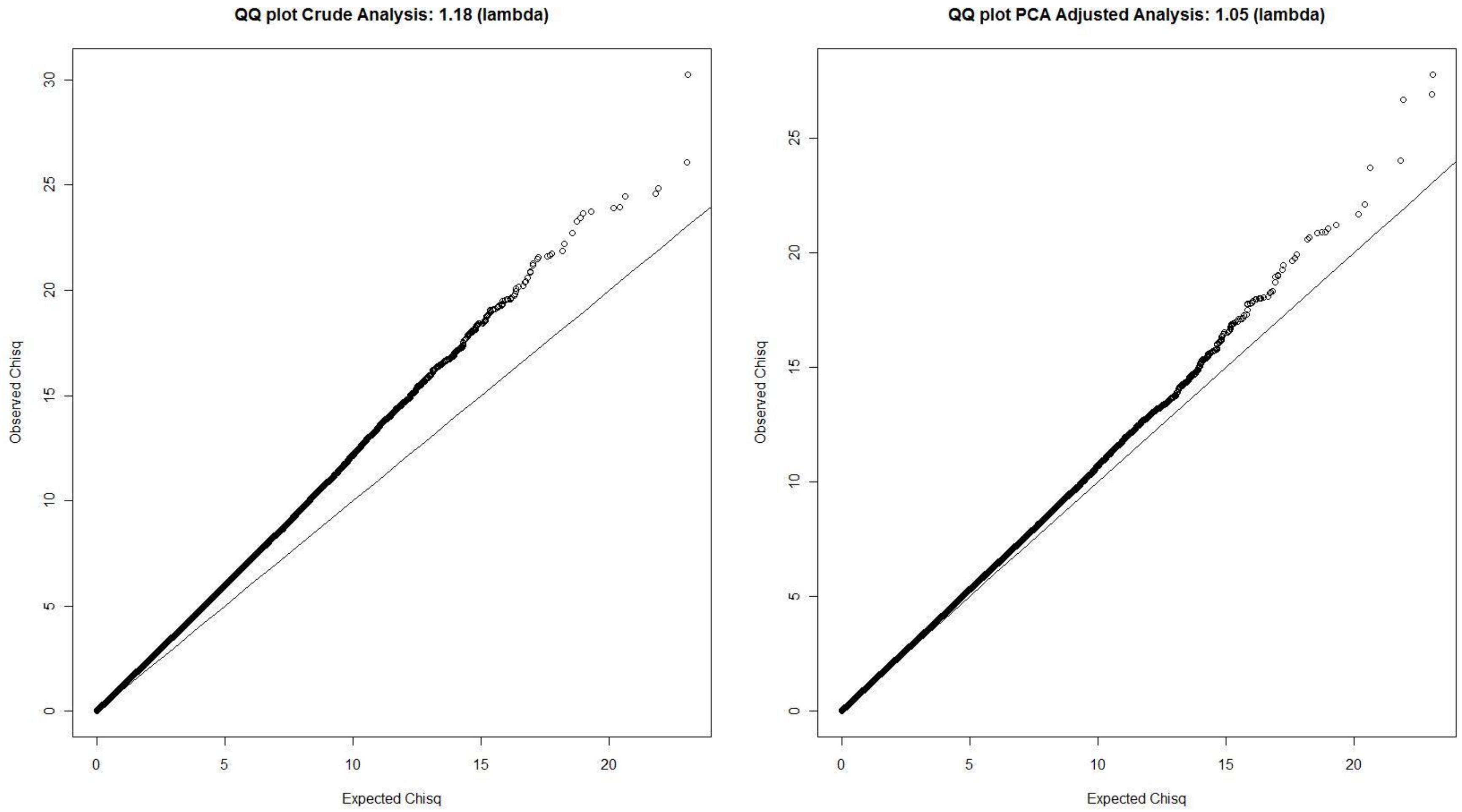

Figure S2 Genomic inflation factor λ before and after PCA-based adjustment. The QQ plot in the left panel show the unadjusted genomic inflation factor (λ=1.18). The right panel show the QQ plot after PCA-based adjustment. After adjustment λ is reduced to 1.05.
