# Supplementary material for: Genome-Wide Association Study Link Novel Loci to Endometriosis
Source: PLoS One. 2013 Mar 5;8(3):e58257. doi: 10.1371/journal.pone.0058257 (PMC3589333; doi:10.1371/journal.pone.0058257)
Supplement: Figure S3 — Regional association plots for the five top regions (Panel A–E). (PDF) [file pone.0058257.s003.pdf]

Panel A

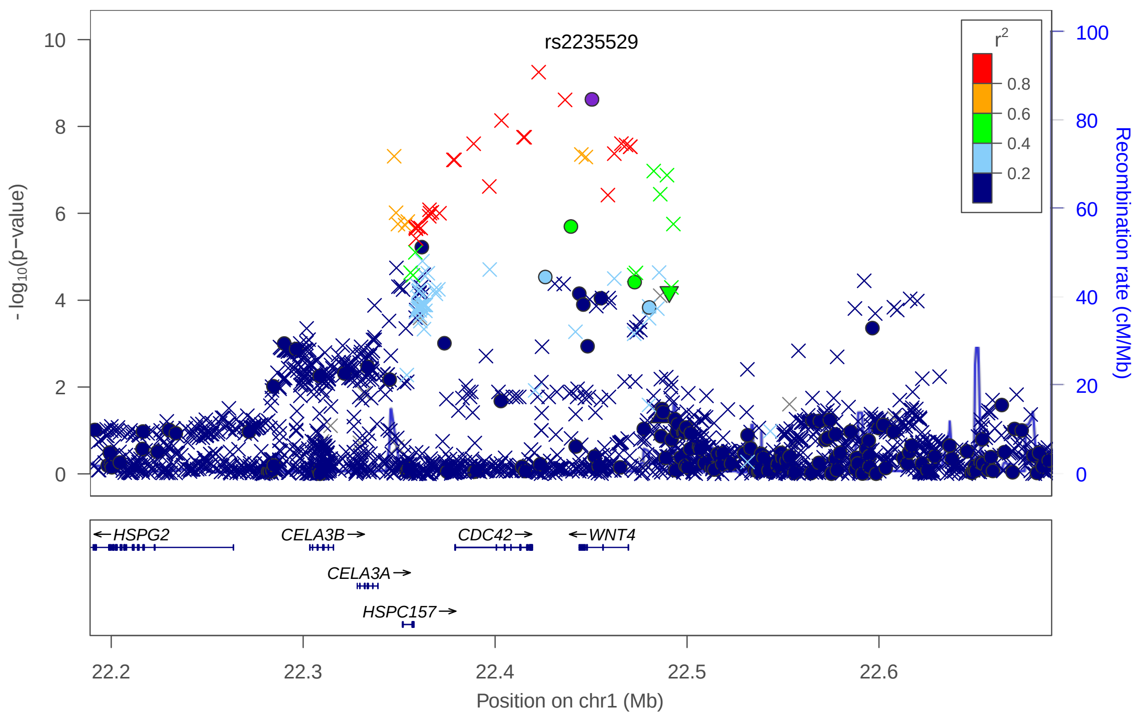

Panel B

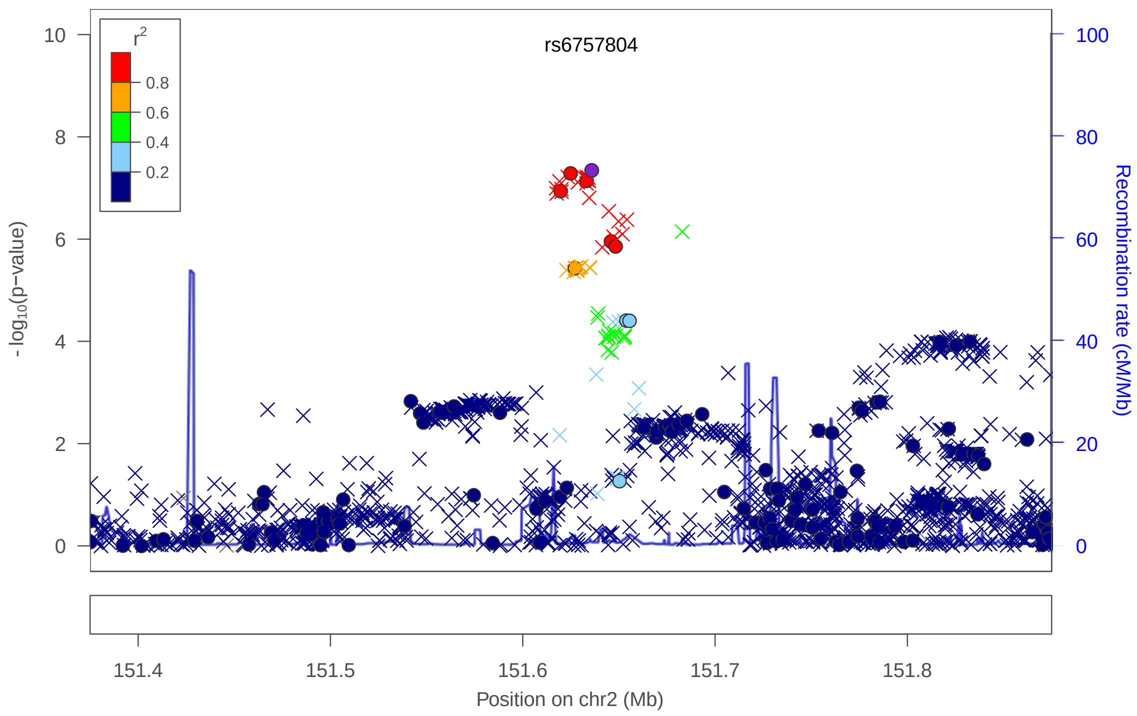

Panel C

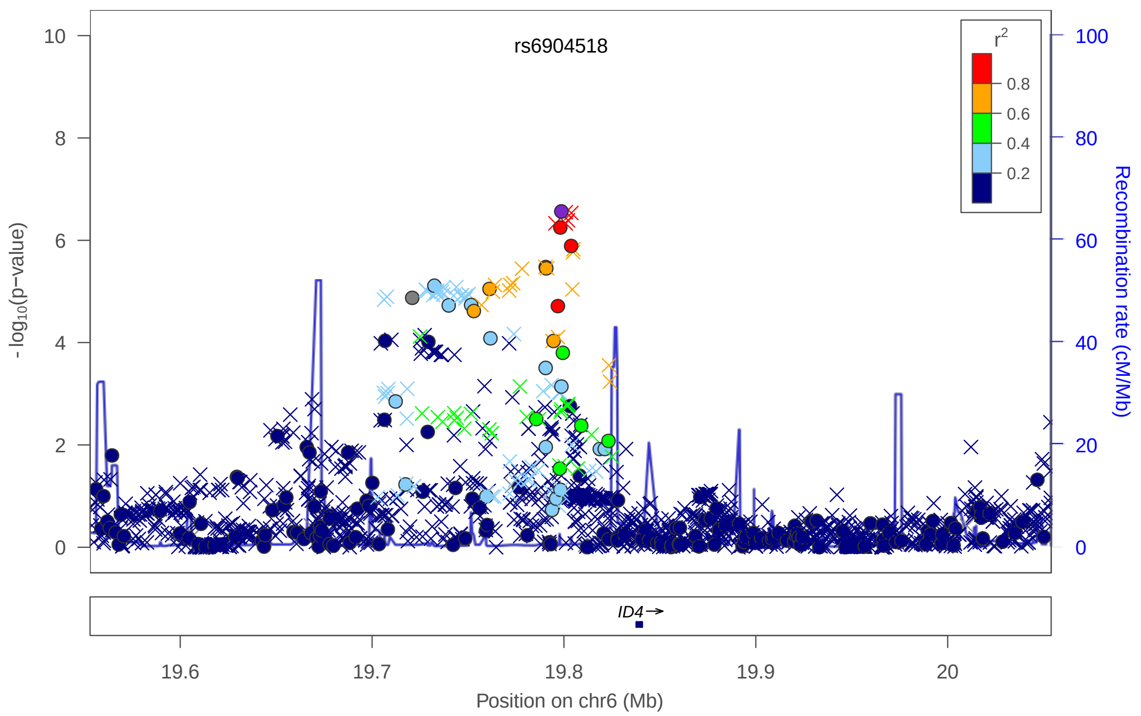

Panel D

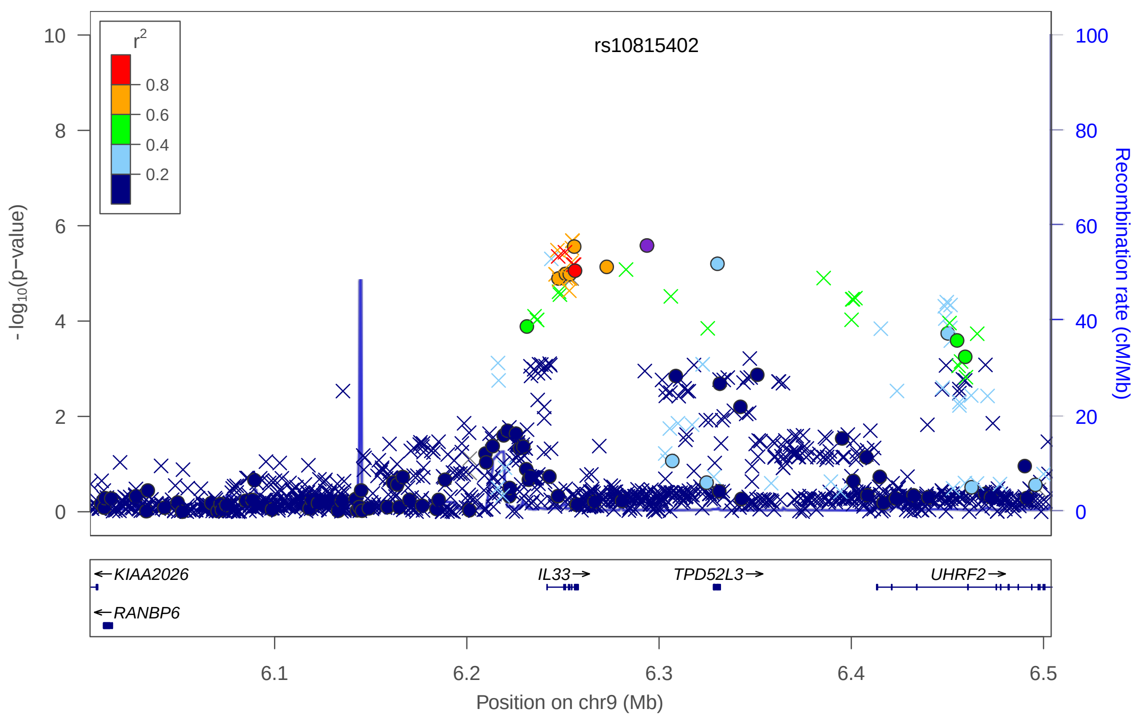

Panel E

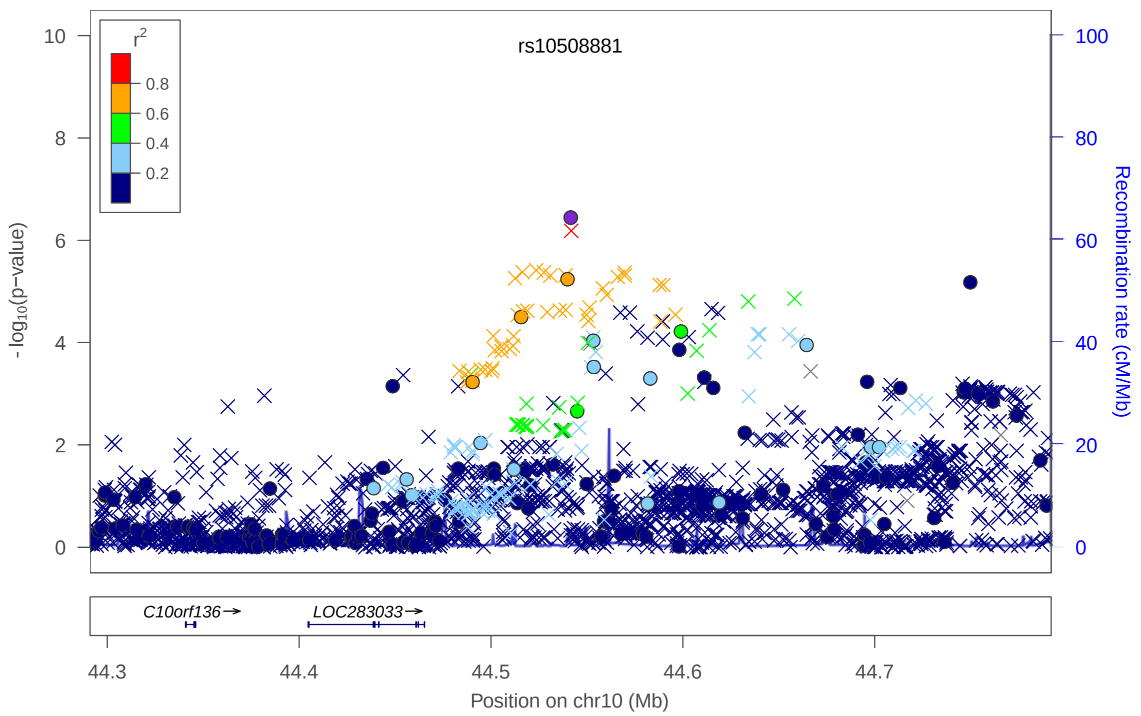

Figure S3 Association plots for five candidate endometriosis regions. The five panels show the association plots for the four regions that reached genome-wide significance in the meta analysis (Panels A, B, C and E) together with the association plot around IL33 on chromosome 9 (Panel D). The association plots are based on P-values determined from the imputed genotypes and listed in Tables S4 A-E. P-values of genotyped SNPs(●) and imputed SNPs (×) are plotted against their physical position on chromosome 1 as -log10(P-value) on the left (hg19/GRCh37). The genetic recombination rates estimated from 1000 Genome samples (EUR) are shown with a blue line according to the scale indicated to the right. The location and identity of known genes and the chromosomal positions are shown at the bottom of each Panel. The imputed regions each span 500kb with the most significant, genotyped SNP, at the center. Samples were pre-phased with IMPUTE2 using actual genotypes and then imputed for SNPs included in the 1000-Genome reference panel to form imputed haplotypes. Only SNPs that pass the confidence score of >=0.9 from imputation, call-rate >0.95 and with MAF >0.01 are shown. Panel A show a gene, HSPC157, also known by its gene symbol: LINC00339.
