# Supplementary material for: Genome-Wide Association Study Link Novel Loci to Endometriosis
Source: PLoS One. 2013 Mar 5;8(3):e58257. doi: 10.1371/journal.pone.0058257 (PMC3589333; doi:10.1371/journal.pone.0058257)
Supplement: Figure S4 — Genotype clusters for the 8 most strongly associated SNPs. (PDF) [file pone.0058257.s004.pdf]

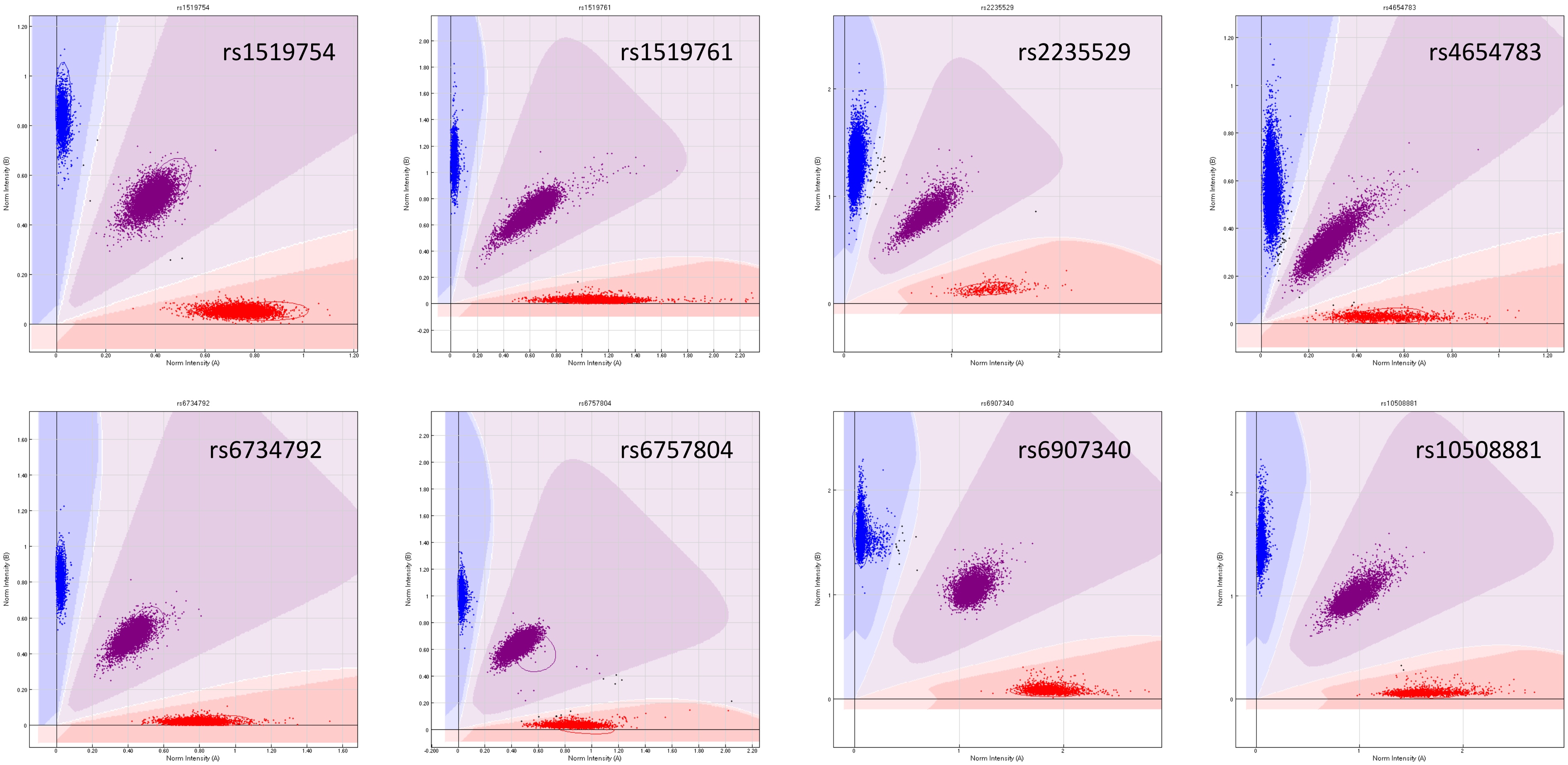

Figure S4 Genotype clusters for the 8 most strongly associated SNPs. The cluster plots represent the 2,019 endometriosis cases and 14,471 population controls included in the study. The clusters are clearly defined and each SNP has very high call-rate (>0.998). It is our experience with data of this quality, that technical replication doesn't affect the genotype calls significantly.
