# Supplementary material for: Genome-Wide Association Study Link Novel Loci to Endometriosis
Source: PLoS One. 2013 Mar 5;8(3):e58257. doi: 10.1371/journal.pone.0058257 (PMC3589333; doi:10.1371/journal.pone.0058257)
Supplement: Table S1 — Top 100 SNPs from Discovery and Replication GWAS. (PDF) [file pone.0058257.s005.pdf]

Table S1 Discovery, Replication, Meta analysis and Combined analysis.

							Discovery Set [a)]				Replication Set [b)]				Meta-Analysis [c)]		Combined-Analysis [d)]			
SNP	Rep	Chr	Base-Pair	Minor Allele	Gene	Location	MAF Cases	MAF Controls	PCA Adjusted P-trend	OR	MAF Case	MAF Controls	P-value	OR	P-value	OR	MAF Case	MAF Control	P-value	OR
rs4654783	S	1	22,439,520	A	LOC100289113 | WNT4	INTERGENIC	0.340	0.295	2.43E-07	1.23	0.323	0.298	1.31E-01	1.13	1.40E-07	1.21	0.336	0.295	1.17E-07	1.21
rs3765351	-	1	22,445,991	G	WNT4 / LOC100289113	COMPLEX	0.491	0.455	7.33E-05	1.16	0.474	0.455	2.73E-01	1.08	1.05E-04	1.14	0.487	0.455	1.22E-04	1.14
rs2235529	S	1	22,450,487	A	WNT4	INTRON	0.188	0.153	1.36E-07	1.28	0.182	0.142	1.38E-03	1.36	3.05E-09	1.30	0.187	0.151	8.45E-09	1.29
rs2473241	-	1	22,596,600	A	WNT4 | ZBTB40	INTERGENIC	0.162	0.136	7.16E-05	1.22	0.136	0.133	7.67E-01	1.04	3.96E-04	1.18	0.155	0.136	6.65E-04	1.17
rs882024	-	1	23,050,262	C	EPHB2	INTRON	0.464	0.422	2.51E-05	1.19	0.421	0.431	5.73E-01	0.96	2.64E-04	1.13	0.453	0.423	2.63E-04	1.13
rs882025	-	1	23,050,352	A	EPHB2	INTRON	0.467	0.425	3.20E-05	1.19	0.421	0.432	5.23E-01	0.96	3.09E-04	1.13	0.455	0.426	3.43E-04	1.13
rs2983118	-	1	37,163,878	G	CSF3R | GRIK3	INTERGENIC	0.362	0.318	2.61E-06	1.22	0.302	0.316	3.84E-01	0.93	1.04E-04	1.15	0.346	0.317	2.15E-04	1.14
rs1395455	-	1	37,185,190	A	CSF3R | GRIK3	INTERGENIC	0.351	0.312	2.19E-05	1.19	0.296	0.310	3.97E-01	0.93	6.25E-04	1.13	0.337	0.311	1.10E-03	1.12
rs2786485	-	1	42,378,784	A	HIVEP3	INTRON	0.149	0.124	6.31E-05	1.24	0.114	0.121	5.35E-01	0.94	1.47E-03	1.17	0.140	0.124	2.98E-03	1.16
rs4660584	-	1	42,390,317	A	HIVEP3 | GUCA2B	INTERGENIC	0.148	0.124	7.48E-05	1.23	0.112	0.120	5.34E-01	0.94	1.68E-03	1.17	0.139	0.123	3.56E-03	1.15
rs1039871	-	1	162,694,593	G	DDR2	INTRON	0.213	0.181	4.51E-06	1.23	0.167	0.186	1.72E-01	0.88	1.61E-03	1.14	0.202	0.181	1.96E-03	1.14
rs1368087	-	2	43,964,837	G	PLEKHH2	INTRON	0.314	0.279	5.39E-05	1.19	0.269	0.278	5.97E-01	0.96	6.47E-04	1.13	0.303	0.279	9.74E-04	1.13
rs12473304	-	2	69,558,112	A	GFPT1	INTRON	0.179	0.155	6.01E-05	1.19	0.182	0.172	4.51E-01	1.08	6.13E-04	1.16	0.180	0.157	1.78E-04	1.18
rs4284854	-	2	80,025,940	A	CTNNA2	INTRON	0.277	0.238	8.71E-06	1.23	0.234	0.236	8.95E-01	0.99	2.77E-05	1.18	0.267	0.237	4.76E-05	1.17
rs1160581	-	2	80,026,432	G	CTNNA2	INTRON	0.299	0.257	5.01E-06	1.23	0.250	0.252	8.79E-01	0.99	1.96E-05	1.17	0.287	0.256	4.26E-05	1.16
rs1519754	S	2	151,619,693	C	RND3 | RBM43	INTERGENIC	0.446	0.403	5.67E-05	1.19	0.450	0.405	9.63E-03	1.20	1.75E-07	1.20	0.447	0.403	1.15E-07	1.20
rs6734792	S	2	151,624,882	G	RND3 | RBM43	INTERGENIC	0.448	0.404	3.52E-05	1.20	0.453	0.406	7.48E-03	1.21	8.18E-08	1.20	0.449	0.404	5.15E-08	1.20
rs1519761	S	2	151,633,204	G	RND3 | RBM43	INTERGENIC	0.445	0.401	3.54E-05	1.20	0.452	0.403	5.85E-03	1.21	7.30E-08	1.20	0.447	0.402	4.81E-08	1.20
rs6757804	S	2	151,635,832	G	RND3 | RBM43	INTERGENIC	0.445	0.401	3.43E-05	1.20	0.452	0.403	5.44E-03	1.21	6.45E-08	1.20	0.446	0.401	4.19E-08	1.20
rs1434094	-	2	179,236,048	A	OSBPL6	INTRON	0.529	0.484	4.14E-06	1.20	0.460	0.483	1.84E-01	0.91	5.29E-04	1.13	0.512	0.484	1.05E-03	1.12
rs6706330	-	2	179,242,526	A	OSBPL6	INTRON	0.442	0.484	4.87E-06	0.84	0.509	0.490	2.82E-01	1.08	8.30E-04	0.89	0.459	0.485	1.94E-03	0.90
rs6738749	-	2	179,243,248	A	OSBPL6	INTRON	0.486	0.449	3.83E-05	1.16	0.421	0.445	1.72E-01	0.91	7.00E-03	1.10	0.469	0.449	1.37E-02	1.09
rs10171524	-	2	212,137,884	A	CPS1 | ERBB4	INTERGENIC	0.435	0.471	4.66E-05	0.87	0.459	0.472	4.46E-01	0.95	2.96E-04	0.88	0.441	0.471	3.84E-04	0.89
rs11713777	-	3	32,245,557	G	GPD1L | LOC100129194	INTERGENIC	0.107	0.084	1.85E-05	1.31	0.085	0.081	6.54E-01	1.05	7.68E-05	1.25	0.102	0.084	1.67E-04	1.24
rs2236951	r	3	50,421,081	G	CACNA2D2	INTRON	0.226	0.195	8.20E-05	1.21	0.215	0.199	2.87E-01	1.10	4.47E-05	1.18	0.223	0.196	3.71E-05	1.18
rs4305418	-	3	116,281,408	G	LSAMP | BZW1L1	INTERGENIC	0.378	0.339	4.68E-05	1.18	0.352	0.341	5.24E-01	1.06	4.69E-05	1.15	0.372	0.340	5.03E-05	1.15
rs907059	-	3	156,292,361	A	SSR3 | LOC100287227	INTERGENIC	0.288	0.248	9.61E-07	1.23	0.258	0.269	4.99E-01	0.94	1.12E-04	1.16	0.280	0.250	4.22E-05	1.17
rs1510272	-	3	156,300,724	A	SSR3 | LOC100287227	INTERGENIC	0.296	0.261	2.50E-05	1.19	0.268	0.279	5.14E-01	0.94	9.67E-04	1.13	0.289	0.263	4.93E-04	1.14
rs10513491	-	3	156,320,230	A	SSR3 | LOC100287227	INTERGENIC	0.260	0.227	3.98E-05	1.20	0.241	0.245	7.84E-01	0.98	5.71E-04	1.14	0.255	0.229	2.49E-04	1.15
rs1373475	-	4	36,931,424	A	FLJ16686 | KIAA1239	INTERGENIC	0.176	0.146	8.10E-05	1.26	0.149	0.153	7.64E-01	0.98	1.32E-04	1.19	0.170	0.146	1.22E-04	1.19
rs11724057	-	4	62,686,490	A	LPHN3	INTRON	0.141	0.114	3.96E-05	1.27	0.118	0.114	6.79E-01	1.05	5.82E-05	1.22	0.136	0.114	8.80E-05	1.22
rs978335	-	4	133,833,738	A	LOC100128747 | PCDH10	INTERGENIC	0.064	0.046	4.12E-05	1.40	0.045	0.045	9.64E-01	0.99	1.84E-04	1.31	0.059	0.046	3.36E-04	1.30
rs3922934	-	4	153,548,037	A	TMEM154	UTR	0.083	0.064	3.75E-05	1.32	0.050	0.067	5.84E-02	0.73	1.07E-02	1.18	0.074	0.064	1.46E-02	1.17
rs17403181	-	4	162,552,883	A	FSTL5	INTRON	0.408	0.444	8.12E-05	0.87	0.442	0.443	9.40E-01	0.99	9.17E-04	0.89	0.416	0.443	1.10E-03	0.90
rs6835945	r	4	182,585,855	A	LOC100288373 | MGC45800	INTERGENIC	0.320	0.362	1.54E-05	0.83	0.353	0.366	4.45E-01	0.95	1.35E-05	0.86	0.328	0.362	2.58E-05	0.86
rs17279486	-	4	182,592,541	G	LOC100288373 | MGC45800	INTERGENIC	0.320	0.361	2.22E-05	0.83	0.354	0.364	5.59E-01	0.96	2.63E-05	0.86	0.328	0.361	4.68E-05	0.86
rs12517129	-	5	25,436,319	A	LOC100288253 | CDH9	INTERGENIC	0.184	0.158	6.98E-05	1.21	0.145	0.154	4.59E-01	0.93	2.92E-03	1.14	0.174	0.157	5.43E-03	1.13
rs11740761	-	5	25,446,757	A	LOC100288253 | CDH9	INTERGENIC	0.184	0.156	3.50E-05	1.21	0.145	0.153	5.60E-01	0.95	1.46E-03	1.15	0.174	0.156	2.84E-03	1.14
rs12186488	-	5	25,471,919	A	LOC100288253 | CDH9	INTERGENIC	0.186	0.158	5.24E-05	1.22	0.143	0.155	3.52E-01	0.91	2.32E-03	1.15	0.175	0.158	4.61E-03	1.14
rs4594818	-	5	25,992,457	G	LOC100288253 | CDH9	INTERGENIC	0.160	0.131	2.56E-05	1.27	0.130	0.131	9.05E-01	0.99	1.02E-04	1.20	0.152	0.131	1.34E-04	1.20
rs2918439	-	5	85,251,243	A	LOC645181 | NBPF22P	INTERGENIC	0.112	0.136	4.66E-05	0.80	0.146	0.131	2.27E-01	1.13	8.52E-03	0.87	0.120	0.135	9.10E-03	0.87
rs2748359	-	6	7,943,101	A	TXNDC5 | MUTED	INTERGENIC	0.376	0.337	4.98E-05	1.19	0.331	0.340	6.16E-01	0.97	3.91E-04	1.13	0.365	0.338	5.08E-04	1.13
rs426518	-	6	13,262,589	G	PHACTR1	INTRON	0.113	0.090	8.03E-06	1.29	0.084	0.086	8.37E-01	0.95	5.07E-04	1.21	0.106	0.090	1.20E-03	1.20
rs6916251	r	6	19,761,215	G	RNF144B | ID4	INTERGENIC	0.467	0.426	5.21E-05	1.18	0.459	0.429	9.36E-02	1.13	6.44E-06	1.17	0.465	0.427	4.76E-06	1.17
rs760794	r	6	19,790,560	A	RNF144B | ID4	INTERGENIC	0.470	0.428	3.82E-05	1.18	0.466	0.434	6.99E-02	1.14	3.11E-06	1.17	0.469	0.429	1.88E-06	1.17
rs2223361	r	6	19,790,809	A	RNF144B | ID4	INTERGENIC	0.470	0.428	3.97E-05	1.18	0.464	0.433	8.52E-02	1.13	3.83E-06	1.17	0.468	0.429	2.44E-06	1.17
rs2206034	r	6	19,796,863	A	RNF144B | ID4	INTERGENIC	0.479	0.439	2.15E-05	1.18	0.475	0.450	1.53E-01	1.11	9.49E-06	1.16	0.478	0.440	3.46E-06	1.17
rs6903595	r	6	19,798,141	A	RNF144B | ID4	INTERGENIC	0.422	0.381	4.22E-05	1.19	0.420	0.390	8.93E-02	1.13	3.15E-06	1.17	0.422	0.382	1.33E-06	1.18
rs6904518	r	6	19,798,704	G	RNF144B | ID4	INTERGENIC	0.425	0.383	2.24E-05	1.19	0.426	0.388	2.75E-02	1.17	5.74E-07	1.19	0.425	0.383	2.67E-07	1.19
rs6907340	S	6	19,803,768	A	RNF144B | ID4	INTERGENIC	0.417	0.371	5.49E-06	1.21	0.412	0.378	4.54E-02	1.15	2.19E-07	1.20	0.415	0.372	9.65E-08	1.20
rs11964747	-	6	20,485,898	A	E2F3	INTRON	0.210	0.183	6.44E-05	1.19	0.180	0.185	7.43E-01	0.96	2.63E-03	1.14	0.203	0.183	3.55E-03	1.13
rs3129304	-	6	32,973,743	G	HLA-DOA	UTR	0.186	0.160	7.50E-05	1.20	0.148	0.161	3.07E-01	0.91	5.48E-03	1.13	0.176	0.160	7.76E-03	1.13
rs3129303	-	6	32,973,878	G	HLA-DOA	UTR	0.186	0.160	6.10E-05	1.20	0.147	0.161	2.89E-01	0.90	5.23E-03	1.13	0.176	0.160	7.66E-03	1.13
rs711274	-	6	91,611,628	A	MAP3K7 | EPHA7	INTERGENIC	0.155	0.188	1.36E-05	0.79	0.190	0.184	6.69E-01	1.04	2.16E-04	0.84	0.164	0.188	2.67E-04	0.85
rs10265932	-	7	31,903,346	A	PDE1C	INTRON	0.382	0.342	2.25E-05	1.19	0.319	0.331	4.63E-01	0.95	3.69E-04	1.13	0.366	0.340	1.12E-03	1.12
rs2270221	-	7	31,904,027	A	PDE1C	INTRON	0.314	0.267	2.14E-07	1.26	0.267	0.266	9.09E-01	1.00	1.05E-06	1.20	0.302	0.267	2.33E-06	1.19
rs1860786	-	7	31,918,879	G	PDE1C	INTRON	0.450	0.408	3.91E-05	1.19	0.378	0.395	3.63E-01	0.93	4.79E-04	1.13	0.433	0.406	1.57E-03	1.12
rs6462315	-	7	31,981,713	A	PDE1C	INTRON	0.349	0.311	2.21E-05	1.19	0.306	0.305	9.69E-01	1.00	1.47E-04	1.15	0.338	0.310	3.06E-04	1.14
rs12701165	-	7	32,018,676	G	PDE1C	INTRON	0.219	0.188	1.29E-05	1.21	0.184	0.184	9.81E-01	1.00	3.95E-04	1.16	0.210	0.188	7.10E-04	1.15
rs2429213	-	7	139,960,630	G	LOC100134229 | SLC37A3	INTERGENIC	0.193	0.227	1.95E-05	0.81	0.232	0.224	5.84E-01	1.05	5.59E-04	0.87	0.203	0.227	6.87E-04	0.87
rs7816936	r	8	5,071,412	A	CSMD1 | LOC100287015	INTERGENIC	0.404	0.446	3.85E-05	0.84	0.414	0.438	1.79E-01	0.90	5.87E-06	0.86	0.406	0.445	3.16E-06	0.85

rs1330383	r	9	6,251,507	A	IL33	INTRON	0.337	0.299	7.91E-05	1.20	0.321	0.297	1.46E-01	1.12	5.93E-06	1.18	0.333	0.299	7.32E-06	1.17
rs10975519	r	9	6,253,571	A	IL33	CODING	0.344	0.300	4.86E-06	1.23	0.321	0.302	2.53E-01	1.09	9.26E-07	1.19	0.338	0.300	9.88E-07	1.19
rs1332290	r	9	6,255,881	A	IL33	INTRON	0.396	0.353	1.91E-05	1.20	0.374	0.350	1.62E-01	1.11	1.75E-06	1.18	0.390	0.352	2.42E-06	1.18
rs1048274	r	9	6,256,292	A	IL33	UTR	0.337	0.298	7.12E-05	1.20	0.320	0.297	1.69E-01	1.11	6.35E-06	1.18	0.333	0.298	7.53E-06	1.17
rs10815398	r	9	6,272,766	C	IL33 | LOC645969	INTERGENIC	0.400	0.358	2.34E-05	1.20	0.371	0.355	3.45E-01	1.07	8.57E-06	1.17	0.393	0.358	1.38E-05	1.16
rs10815402	r	9	6,293,715	A	LOC645969 | TPD52L3	INTERGENIC	0.309	0.271	7.69E-05	1.20	0.293	0.266	8.41E-02	1.15	2.58E-06	1.19	0.305	0.270	4.24E-06	1.18
rs2492813	-	9	27,497,792	G	MOBKL2B | IFNK	INTERGENIC	0.020	0.011	2.27E-05	1.88	0.011	0.011	9.28E-01	0.97	1.02E-04	1.66	0.018	0.011	1.36E-04	1.65
rs815845	-	9	84,216,090	C	TLE1	INTRON	0.344	0.308	5.92E-05	1.18	0.309	0.311	8.94E-01	0.99	5.03E-04	1.13	0.335	0.308	5.60E-04	1.13
rs10739696	-	9	130,527,786	C	SH2D3C	INTRON	0.393	0.426	5.94E-05	0.87	0.432	0.412	2.63E-01	1.08	1.09E-02	0.92	0.403	0.424	9.23E-03	0.91
rs4836579	-	9	130,528,548	G	SH2D3C	INTRON	0.412	0.445	5.76E-05	0.87	0.440	0.431	6.34E-01	1.03	4.80E-03	0.91	0.419	0.443	3.66E-03	0.91
rs10760500	-	9	130,536,717	G	SH2D3C	COMPLEX	0.411	0.445	5.84E-05	0.87	0.440	0.432	6.44E-01	1.03	4.45E-03	0.91	0.419	0.443	3.48E-03	0.90
rs1875005	-	10	1,661,398	A	NCRNA00168 | ADARB2	INTERGENIC	0.435	0.476	8.08E-05	0.85	0.471	0.471	9.80E-01	1.00	1.80E-04	0.88	0.444	0.475	1.92E-04	0.88
rs2942366	r	10	17,030,854	A	CUBN	INTRON	0.483	0.443	7.94E-05	1.18	0.462	0.454	6.42E-01	1.04	7.72E-05	1.14	0.478	0.444	5.03E-05	1.15
rs10508881	S	10	44,541,565	A	HNRNPA3P1 | LOC100130539	INTERGENIC	0.450	0.405	3.18E-05	1.20	0.420	0.387	6.06E-02	1.15	4.08E-07	1.19	0.442	0.403	1.61E-06	1.18
rs11193561	-	10	109,374,384	A	SORCS1 | LOC100128304	INTERGENIC	0.279	0.239	3.24E-06	1.24	0.239	0.231	6.12E-01	1.04	6.04E-06	1.19	0.269	0.238	1.58E-05	1.18
rs17608302	-	10	121,129,167	A	GRK5	INTRON	0.194	0.227	7.27E-05	0.82	0.244	0.226	2.27E-01	1.09	2.13E-03	0.88	0.206	0.227	3.13E-03	0.89
rs4910169	-	11	10,709,168	G	MRVI1	INTRON	0.271	0.237	9.35E-06	1.20	0.246	0.236	5.10E-01	1.06	5.09E-05	1.17	0.265	0.237	7.48E-05	1.17
rs7129273	-	11	10,724,434	G	MRVI1 | CTR9	INTERGENIC	0.273	0.240	2.43E-05	1.19	0.249	0.237	4.65E-01	1.06	1.20E-04	1.16	0.267	0.240	1.88E-04	1.15
rs7106873	-	11	70,425,843	A	SHANK2	INTRON	0.402	0.441	3.55E-05	0.85	0.415	0.434	2.78E-01	0.93	3.85E-05	0.87	0.406	0.440	2.90E-05	0.87
rs10765405	-	11	90,677,706	G	LOC399942 | FAT3	INTERGENIC	0.291	0.328	7.28E-05	0.84	0.345	0.320	1.35E-01	1.12	4.12E-03	0.90	0.305	0.327	4.49E-03	0.90
rs12426819	-	12	60,103,232	G	SLC16A7	INTRON	0.446	0.489	7.11E-05	0.84	0.496	0.493	8.54E-01	1.01	9.61E-05	0.88	0.458	0.490	1.89E-04	0.88
rs7963889	-	12	74,850,683	G	TRHDE | LOC552889	INTERGENIC	0.500	0.461	1.13E-05	1.17	0.450	0.459	6.01E-01	0.97	8.11E-04	1.12	0.487	0.460	1.42E-03	1.11
rs10859856	r	12	95,631,276	G	VEZT	INTRON	0.514	0.470	1.04E-05	1.19	0.482	0.460	2.02E-01	1.10	4.23E-06	1.17	0.506	0.469	1.10E-05	1.16
rs3596	r	12	95,696,420	G	VEZT	UTR	0.517	0.472	5.69E-06	1.20	0.483	0.460	1.89E-01	1.10	2.33E-06	1.17	0.508	0.471	6.74E-06	1.16
rs1362969	-	12	96,014,946	A	USP44 | LOC100132594	INTERGENIC	0.258	0.291	4.46E-05	0.85	0.269	0.282	3.90E-01	0.93	1.98E-04	0.87	0.261	0.290	1.33E-04	0.86
rs10431397	-	12	118,106,194	A	KSR2	INTRON	0.022	0.012	1.13E-06	1.93	0.015	0.013	6.55E-01	1.15	7.11E-06	1.73	0.020	0.012	5.74E-06	1.74
rs2138077	-	12	128,666,287	A	LOC100288417 | TMEM132C	INTERGENIC	0.396	0.356	1.33E-05	1.19	0.350	0.357	6.77E-01	0.97	3.11E-04	1.13	0.384	0.356	4.27E-04	1.13
rs1533352	-	12	128,667,419	G	LOC100288417 | TMEM132C	INTERGENIC	0.362	0.325	2.13E-05	1.18	0.317	0.325	6.02E-01	0.96	6.23E-04	1.13	0.351	0.325	8.80E-04	1.12
rs10847559	-	12	128,672,901	A	LOC100288417 | TMEM132C	INTERGENIC	0.374	0.338	7.37E-05	1.17	0.331	0.335	8.29E-01	0.99	9.60E-04	1.12	0.363	0.338	1.63E-03	1.12
rs9579955	-	13	21,376,974	G	XPO4	INTRON	0.111	0.140	4.85E-05	0.77	0.143	0.140	8.04E-01	1.02	1.71E-04	0.82	0.119	0.140	2.40E-04	0.83
rs1512883	-	13	63,551,022	A	PCDH20 | OR7E156P	INTERGENIC	0.177	0.147	3.29E-05	1.24	0.135	0.150	2.34E-01	0.89	1.34E-03	1.16	0.167	0.148	1.72E-03	1.15
rs10132077	r	14	59,013,971	C	KIAA0586	INTRON	0.252	0.221	7.69E-05	1.19	0.252	0.221	3.99E-02	1.17	1.13E-05	1.19	0.252	0.221	9.72E-06	1.19
rs1268843	r	14	93,644,379	A	ITPK1 | MOAP1	INTERGENIC	0.302	0.263	2.88E-05	1.22	0.281	0.263	2.53E-01	1.10	3.12E-06	1.19	0.297	0.263	3.82E-06	1.19
rs2445751	-	15	51,696,003	A	GLDN	INTRON	0.509	0.469	7.91E-05	1.18	0.464	0.471	6.86E-01	0.97	4.51E-04	1.13	0.498	0.469	5.72E-04	1.12
rs12449465	r	17	3,029,554	G	OR1D2 | OR1G1	INTERGENIC	0.152	0.125	4.77E-05	1.26	0.146	0.119	2.32E-02	1.27	1.43E-06	1.26	0.151	0.124	2.50E-06	1.25
rs6139282	-	20	4,045,738	A	RNF24 | SMOX	INTERGENIC	0.058	0.041	2.20E-05	1.43	0.048	0.047	8.41E-01	1.04	1.45E-04	1.33	0.055	0.042	7.20E-05	1.35
rs8050	-	20	34,237,224	A	RBM12	UTR	0.017	0.029	3.90E-05	0.58	0.027	0.024	5.91E-01	1.13	2.08E-03	0.69	0.020	0.028	1.42E-03	0.68
rs909182	-	21	41,033,816	A	B3GALT5	UTR	0.346	0.312	4.26E-05	1.17	0.300	0.322	1.86E-01	0.90	6.43E-03	1.10	0.335	0.314	6.53E-03	1.10
rs5767685	-	22	47,718,677	G	LOC100289420 | FAM19A5	INTERGENIC	0.088	0.067	1.94E-05	1.35	0.079	0.074	5.78E-01	1.07	6.14E-05	1.28	0.086	0.068	3.59E-05	1.29

Table S1 summarize the top 100 SNPs identified in the discovery GWAS and show the behaviour of these SNPs in the replication GWAS. The meta-Analysis was performed using the CMH-test and revealed 29 SNPs that were replicated. The column "Rep" identify significant SNPs (S) and replicating SNPs (r). SNPs are ordered by chromsome and position.

[a)] The Discovery Set included 1,514 surgically diagnosed endometriosis patients and 12,660 population controls of European ancestry and analyzed using the Cochran-Armitage trend test as implemented in PLINK (ver 1.07).

[b)] The Replication Set included 505 surgically diagnosed endometriosis patients and 1,811 population controls of European ancestry and analyzed using the Cochran-Armitage trend test as implemented in PLINK (ver 1.07).

[c)] For the Meta Analysis we employed the Cochran-Mantel-Hanzel test and identified 8 SNPs from 4 distinct loci that passed genome-wide significance.

[d)] The Combined-Analysis include all study samples (cases=2,019; controls=14,471) analyzed using the Cochran-Armitage trend test. Three of the four loci remained significant while one SNP on chromsome 10, (rs10508881), fell below the significance threshold.
