# Supplementary material for: Genome-Wide Association Study Link Novel Loci to Endometriosis
Source: PLoS One. 2013 Mar 5;8(3):e58257. doi: 10.1371/journal.pone.0058257 (PMC3589333; doi:10.1371/journal.pone.0058257)
Supplement: Table S2 — a Genotyped and Imputed P-values for the LINC00339-WNT4 region on 1p36.12. b Genotyped and Imputed P-values for the RND3-RBM43 region on 2q23.3. c Genotyped and Imputed P-values for the RNF144B-ID4 region on 6p22.3. d Genotyped and Imputed P-values for the IL33-TPD52L3 region on 9p24.1. d Genotyped and Imputed P-values for the HNRNPA3P1-LOC100130539 region on 10q11.21. (PDF) [file pone.0058257.s006.pdf]

Table S2a WNT4 region on chromosome 1

SNP	CHR	BP	Allele 1	Allele 2	F_A	F_U	P cmh test	OR cmh	SE	L95	U95	P het	Method
rs13375327	1	22,189,852	T	C	0.221	0.218	0.598	1.022	0.041	0.943	1.107	0.595	Imputed
chr1:22190826:I	1	22,190,826	GC	G	0.215	0.221	0.470	0.971	0.041	0.896	1.052	0.411	Imputed
rs12404444	1	22,190,933	A	G	0.177	0.180	0.659	0.981	0.044	0.899	1.070	0.820	Imputed
rs897471	1	22,191,454	G	A	0.261	0.275	0.097	0.938	0.038	0.870	1.012	0.800	Genotyped
rs12407706	1	22,193,202	A	C	0.234	0.225	0.182	1.055	0.040	0.975	1.141	0.765	Imputed
rs1076028	1	22,193,238	C	T	0.034	0.033	0.821	1.021	0.094	0.850	1.227	0.331	Imputed
rs6672220	1	22,193,692	C	T	0.034	0.033	0.821	1.021	0.094	0.850	1.227	0.331	Imputed
rs2085272	1	22,194,796	A	G	0.227	0.218	0.167	1.058	0.040	0.977	1.145	0.917	Imputed
rs56013780	1	22,196,236	T	C	0.069	0.065	0.315	1.070	0.067	0.938	1.221	0.116	Imputed
rs36086776	1	22,196,254	T	C	0.045	0.051	0.099	0.875	0.081	0.747	1.026	0.737	Imputed
rs75793979	1	22,196,574	C	G	0.026	0.028	0.553	0.939	0.106	0.762	1.156	0.580	Imputed
chr1:22196900:D	1	22,196,900	C	CA	0.230	0.220	0.132	1.063	0.040	0.982	1.150	0.891	Imputed
rs3767139	1	22,197,336	T	C	0.230	0.220	0.131	1.063	0.040	0.982	1.150	0.892	Imputed
rs3820280	1	22,197,648	G	C	0.142	0.153	0.094	0.922	0.048	0.839	1.014	0.869	Imputed
rs7528670	1	22,197,940	G	A	0.230	0.220	0.134	1.062	0.040	0.982	1.149	0.887	Imputed
rs11586789	1	22,198,190	T	C	0.035	0.034	0.641	1.044	0.092	0.871	1.251	0.276	Genotyped
rs2290497	1	22,198,955	A	G	0.067	0.064	0.490	1.048	0.068	0.917	1.198	0.204	Imputed
rs2229482	1	22,199,245	C	T	0.378	0.371	0.323	1.035	0.035	0.967	1.108	0.813	Genotyped
rs16826011	1	22,199,414	A	G	0.034	0.033	0.742	1.031	0.093	0.860	1.237	0.285	Imputed
rs2454290	1	22,199,821	G	A	0.147	0.151	0.606	0.976	0.048	0.889	1.071	0.960	Genotyped
rs34855688	1	22,199,982	A	G	0.045	0.051	0.102	0.877	0.081	0.748	1.027	0.758	Imputed
rs41307814	1	22,200,052	A	G	0.016	0.017	0.436	0.901	0.134	0.692	1.172	0.396	Imputed
rs2247741	1	22,200,067	A	G	0.331	0.318	0.081	1.065	0.036	0.992	1.143	0.784	Imputed
rs11587853	1	22,200,080	T	C	0.033	0.032	0.590	1.052	0.094	0.875	1.265	0.369	Imputed
rs11584053	1	22,200,300	A	G	0.034	0.033	0.705	1.036	0.093	0.863	1.243	0.187	Genotyped
rs28546127	1	22,200,473	T	C	0.033	0.031	0.384	1.086	0.095	0.902	1.308	0.468	Imputed
rs2229481	1	22,200,998	T	C	0.037	0.035	0.617	1.046	0.090	0.877	1.248	0.566	Imputed
rs2290494	1	22,201,331	T	C	0.228	0.220	0.231	1.050	0.040	0.970	1.136	0.645	Imputed
rs41307806	1	22,201,438	T	C	0.062	0.064	0.706	0.974	0.070	0.848	1.118	0.649	Imputed
rs7355175	1	22,201,796	C	G	0.033	0.032	0.666	1.042	0.095	0.865	1.254	0.211	Imputed
rs7354994	1	22,201,880	T	C	0.099	0.097	0.549	1.035	0.057	0.926	1.156	0.544	Imputed
rs2445134	1	22,201,903	A	G	0.342	0.331	0.101	1.060	0.036	0.989	1.137	0.722	Imputed
rs2454295	1	22,203,259	A	G	0.342	0.330	0.100	1.060	0.036	0.989	1.137	0.734	Imputed
rs2077271	1	22,203,529	G	A	0.349	0.337	0.086	1.063	0.036	0.992	1.140	0.780	Imputed
chr1:22203808:I	1	22,203,808	TA	T	0.098	0.097	0.588	1.031	0.057	0.923	1.153	0.564	Imputed
rs2088507	1	22,204,164	C	T	0.012	0.014	0.475	0.897	0.152	0.665	1.209	0.676	Imputed
rs77666184	1	22,204,318	G	C	0.019	0.017	0.314	1.133	0.124	0.889	1.445	0.805	Imputed

rs747546	1	22,204,506	T	C	0.099	0.097	0.547	1.035	0.057	0.926	1.156	0.597	Genotyped
rs7513608	1	22,204,630	A	G	0.228	0.217	0.112	1.066	0.040	0.985	1.154	0.895	Imputed
rs144786571	1	22,205,191	A	G	0.012	0.013	0.959	0.992	0.152	0.737	1.337	0.214	Imputed
rs55728940	1	22,205,755	A	C	0.065	0.065	0.677	1.029	0.068	0.900	1.177	0.852	Imputed
rs34212628	1	22,206,076	T	C	0.014	0.015	0.846	0.973	0.142	0.737	1.285	0.752	Imputed
rs77828146	1	22,207,065	A	G	0.015	0.015	0.941	0.990	0.139	0.755	1.299	0.742	Imputed
rs76061126	1	22,207,618	T	G	0.013	0.015	0.370	0.876	0.148	0.656	1.170	0.879	Imputed
rs921847	1	22,208,030	C	T	0.016	0.016	0.782	0.963	0.136	0.738	1.257	0.774	Imputed
rs12740543	1	22,208,689	T	G	0.228	0.218	0.123	1.064	0.040	0.983	1.152	0.942	Imputed
rs114546866	1	22,208,834	A	G	0.015	0.016	0.565	0.922	0.141	0.700	1.215	0.446	Imputed
rs2445138	1	22,208,864	C	T	0.016	0.017	0.885	0.981	0.133	0.756	1.273	0.672	Imputed
rs71636996	1	22,209,192	T	C	0.015	0.015	0.941	0.990	0.139	0.755	1.299	0.742	Imputed
rs35799434	1	22,209,827	C	T	0.015	0.015	0.941	0.990	0.139	0.755	1.299	0.742	Imputed
rs2501263	1	22,210,044	C	A	0.345	0.333	0.100	1.060	0.036	0.989	1.137	0.659	Imputed
rs115600691	1	22,210,134	C	T	0.011	0.011	0.960	1.008	0.163	0.733	1.386	0.491	Imputed
rs114502069	1	22,210,597	A	G	0.013	0.014	0.666	0.938	0.149	0.701	1.255	0.108	Imputed
rs76100124	1	22,210,627	A	G	0.044	0.042	0.662	1.037	0.083	0.881	1.220	0.416	Imputed
rs71636997	1	22,210,765	C	T	0.015	0.015	0.941	0.990	0.139	0.755	1.299	0.742	Imputed
rs2290493	1	22,211,217	A	G	0.228	0.218	0.132	1.063	0.040	0.982	1.150	0.935	Imputed
rs4654994	1	22,211,222	T	C	0.067	0.067	0.861	1.012	0.068	0.886	1.155	0.871	Imputed
rs113201880	1	22,211,680	T	C	0.033	0.032	0.627	1.047	0.095	0.870	1.260	0.294	Imputed
rs28653609	1	22,212,125	A	C	0.100	0.098	0.609	1.029	0.056	0.922	1.150	0.591	Imputed
rs2497632	1	22,212,244	C	T	0.344	0.333	0.108	1.059	0.036	0.988	1.136	0.674	Imputed
rs143016062	1	22,212,311	A	G	0.016	0.017	0.452	0.904	0.134	0.695	1.176	0.388	Imputed
rs3753781	1	22,212,485	G	A	0.147	0.153	0.407	0.961	0.048	0.875	1.055	0.789	Imputed
rs77707927	1	22,212,909	G	A	0.010	0.010	0.853	0.969	0.169	0.697	1.349	0.913	Imputed
rs115839045	1	22,213,628	A	G	0.062	0.064	0.808	0.983	0.070	0.857	1.128	0.762	Imputed
rs41310388	1	22,214,040	A	G	0.012	0.011	0.615	1.082	0.157	0.796	1.471	0.337	Imputed
rs41307868	1	22,214,046	T	C	0.062	0.063	0.783	0.981	0.070	0.855	1.126	0.761	Imputed
rs2229478	1	22,214,127	G	A	0.345	0.334	0.125	1.056	0.036	0.985	1.133	0.647	Imputed
rs114537356	1	22,214,279	A	C	0.019	0.021	0.541	0.928	0.123	0.730	1.180	0.383	Imputed
rs3767140	1	22,214,645	A	C	0.229	0.219	0.131	1.063	0.040	0.982	1.150	0.962	Imputed
rs41266013	1	22,215,242	T	C	0.016	0.017	0.452	0.904	0.134	0.695	1.176	0.388	Imputed
rs11587885	1	22,215,419	C	T	0.032	0.031	0.631	1.047	0.096	0.868	1.264	0.299	Imputed
rs10917060	1	22,215,733	A	G	0.287	0.282	0.377	1.034	0.037	0.961	1.112	0.971	Imputed
rs59335091	1	22,216,061	G	C	0.015	0.015	0.826	0.970	0.138	0.740	1.272	0.751	Imputed
rs3767141	1	22,216,279	T	C	0.317	0.309	0.267	1.041	0.036	0.970	1.118	0.754	Genotyped
rs41307818	1	22,216,422	A	G	0.018	0.017	0.786	1.035	0.127	0.807	1.328	0.806	Imputed
rs35863848	1	22,216,442	A	G	0.015	0.015	0.930	0.988	0.139	0.753	1.296	0.748	Imputed
rs2254358	1	22,216,574	C	A	0.345	0.333	0.106	1.059	0.036	0.988	1.136	0.684	Genotyped

rs2254357	1	22,216,604	C	G	0.344	0.333	0.115	1.058	0.036	0.986	1.134	0.672	Imputed
rs10799718	1	22,217,900	A	C	0.317	0.310	0.288	1.039	0.036	0.968	1.116	0.736	Imputed
rs181063796	1	22,218,853	A	G	0.011	0.012	0.447	0.885	0.162	0.644	1.216	0.031	Imputed
rs2454289	1	22,219,012	C	T	0.344	0.333	0.117	1.058	0.036	0.986	1.134	0.675	Imputed
rs113877852	1	22,219,292	G	A	0.012	0.011	0.603	1.085	0.157	0.798	1.475	0.331	Imputed
rs113627058	1	22,219,522	T	C	0.012	0.012	0.930	1.014	0.157	0.746	1.378	0.134	Imputed
rs7520609	1	22,220,610	T	A	0.032	0.031	0.623	1.048	0.096	0.869	1.265	0.297	Imputed
rs115351721	1	22,220,638	A	G	0.013	0.014	0.433	0.890	0.149	0.665	1.192	0.320	Imputed
rs114539176	1	22,220,780	T	C	0.011	0.010	0.767	1.050	0.164	0.761	1.448	0.639	Imputed
rs12402710	1	22,221,355	G	C	0.317	0.310	0.278	1.040	0.036	0.969	1.117	0.727	Imputed
rs897474	1	22,221,431	T	C	0.345	0.333	0.105	1.059	0.036	0.988	1.136	0.669	Imputed
chr1:22221623:D	1	22,221,623	CG	C	0.345	0.333	0.103	1.060	0.036	0.988	1.136	0.666	Imputed
rs71636998	1	22,221,910	T	C	0.020	0.019	0.846	1.024	0.123	0.806	1.302	0.971	Imputed
rs11810496	1	22,224,102	G	A	0.286	0.280	0.309	1.039	0.037	0.965	1.118	0.958	Genotyped
rs76305492	1	22,224,877	G	C	0.066	0.066	0.752	1.022	0.068	0.894	1.168	0.944	Imputed
rs141061782	1	22,225,373	A	G	0.014	0.013	0.454	1.112	0.143	0.841	1.472	0.199	Imputed
rs2445142	1	22,225,743	C	G	0.345	0.333	0.100	1.060	0.036	0.989	1.137	0.708	Imputed
rs79928839	1	22,225,921	T	C	0.010	0.013	0.151	0.788	0.166	0.569	1.092	0.817	Imputed
rs34942222	1	22,226,166	G	T	0.219	0.214	0.387	1.036	0.041	0.956	1.123	0.943	Imputed
rs34565080	1	22,226,588	C	T	0.016	0.016	0.846	1.026	0.135	0.789	1.336	0.500	Imputed
rs71637000	1	22,226,919	T	G	0.015	0.015	0.948	1.009	0.138	0.770	1.321	0.686	Imputed
rs55823448	1	22,226,998	C	T	0.286	0.279	0.313	1.039	0.037	0.965	1.118	0.938	Imputed
rs878949	1	22,227,091	C	T	0.351	0.340	0.116	1.057	0.035	0.986	1.133	0.574	Imputed
rs11589829	1	22,227,188	T	G	0.317	0.308	0.190	1.049	0.036	0.977	1.126	0.835	Imputed
rs34798947	1	22,227,751	A	G	0.016	0.015	0.825	1.030	0.136	0.790	1.344	0.627	Imputed
rs79704247	1	22,228,226	C	G	0.028	0.029	0.719	0.964	0.102	0.790	1.177	0.638	Imputed
rs12752439	1	22,228,239	C	G	0.286	0.279	0.296	1.040	0.037	0.966	1.119	0.897	Imputed
rs76504298	1	22,228,566	A	G	0.023	0.025	0.344	0.899	0.113	0.721	1.121	0.849	Imputed
rs116698906	1	22,228,614	C	G	0.013	0.014	0.823	0.968	0.147	0.725	1.292	0.088	Imputed
rs2501257	1	22,228,836	G	A	0.346	0.333	0.088	1.063	0.036	0.991	1.139	0.734	Imputed
rs12758401	1	22,228,841	T	C	0.015	0.015	0.948	1.009	0.138	0.770	1.321	0.686	Imputed
rs1809207	1	22,228,938	C	T	0.027	0.022	0.062	1.219	0.106	0.989	1.501	0.840	Imputed
rs6680566	1	22,229,090	C	T	0.319	0.310	0.219	1.046	0.036	0.974	1.123	0.729	Imputed
rs148796759	1	22,229,352	G	C	0.014	0.013	0.464	1.110	0.143	0.839	1.468	0.202	Imputed
rs12744726	1	22,229,356	C	T	0.318	0.310	0.243	1.043	0.036	0.972	1.120	0.783	Imputed
rs71569856	1	22,229,832	A	G	0.015	0.015	0.948	1.009	0.138	0.770	1.321	0.686	Imputed
rs2501255	1	22,230,207	C	T	0.345	0.333	0.097	1.061	0.036	0.989	1.138	0.739	Genotyped
rs34788602	1	22,230,964	T	C	0.015	0.015	0.948	1.009	0.138	0.770	1.321	0.686	Imputed
rs143551401	1	22,231,156	A	G	0.013	0.014	0.823	0.968	0.147	0.725	1.292	0.088	Imputed
chr1:22231828:I	1	22,231,828	AT	A	0.286	0.279	0.276	1.042	0.037	0.968	1.121	0.932	Imputed

rs10917062	1	22,232,376	T	C	0.318	0.310	0.226	1.045	0.036	0.973	1.122	0.796	Imputed
rs2501254	1	22,232,477	T	C	0.346	0.333	0.091	1.062	0.036	0.991	1.139	0.719	Imputed
rs11581438	1	22,232,552	A	C	0.032	0.030	0.624	1.048	0.096	0.868	1.266	0.296	Imputed
rs11581439	1	22,232,553	A	C	0.032	0.030	0.624	1.048	0.096	0.868	1.266	0.296	Imputed
chr1:22233217:I	1	22,233,217	TG	T	0.340	0.332	0.223	1.045	0.036	0.974	1.120	0.624	Imputed
chr1:22233222:I	1	22,233,222	GGTT	G	0.072	0.066	0.174	1.094	0.066	0.961	1.246	0.097	Imputed
rs187784422	1	22,233,440	A	G	0.019	0.021	0.364	0.894	0.123	0.702	1.138	0.345	Imputed
rs34160855	1	22,233,501	C	G	0.330	0.324	0.332	1.036	0.036	0.965	1.111	0.612	Imputed
rs66850327	1	22,233,584	C	T	0.330	0.324	0.340	1.035	0.036	0.964	1.111	0.592	Imputed
rs34860998	1	22,233,593	A	G	0.015	0.015	0.948	1.009	0.138	0.770	1.321	0.686	Imputed
rs1545593	1	22,233,670	G	T	0.361	0.349	0.116	1.057	0.035	0.986	1.133	0.698	Genotyped
rs192797333	1	22,234,226	T	C	0.010	0.012	0.376	0.866	0.164	0.628	1.194	0.036	Imputed
rs2501253	1	22,234,424	C	G	0.359	0.348	0.136	1.054	0.035	0.984	1.130	0.572	Imputed
rs4654995	1	22,234,654	T	C	0.367	0.373	0.341	0.967	0.035	0.902	1.036	0.727	Imputed
rs147545934	1	22,236,364	T	C	0.014	0.015	0.498	0.909	0.143	0.687	1.202	0.017	Imputed
rs7519770	1	22,236,549	A	G	0.025	0.023	0.372	1.101	0.108	0.891	1.362	0.728	Imputed
rs115895693	1	22,237,438	T	C	0.032	0.030	0.624	1.048	0.096	0.868	1.266	0.296	Imputed
rs34215629	1	22,238,165	A	G	0.015	0.015	0.808	1.034	0.139	0.788	1.358	0.771	Imputed
rs1002480	1	22,238,775	G	C	0.341	0.328	0.089	1.063	0.036	0.991	1.140	0.619	Imputed
rs2445131	1	22,239,774	T	C	0.394	0.384	0.188	1.047	0.035	0.978	1.120	0.456	Imputed
rs12732887	1	22,240,204	G	A	0.015	0.014	0.790	1.038	0.141	0.787	1.370	0.758	Imputed
rs34951142	1	22,240,753	T	C	0.245	0.234	0.106	1.066	0.039	0.987	1.151	0.720	Imputed
rs11578002	1	22,241,017	A	T	0.015	0.014	0.886	1.020	0.141	0.774	1.346	0.811	Imputed
rs78951217	1	22,241,185	A	G	0.104	0.105	0.801	0.986	0.055	0.885	1.099	0.281	Imputed
rs71637001	1	22,241,544	T	C	0.015	0.014	0.790	1.038	0.141	0.787	1.370	0.758	Imputed
chr1:22241561:D	1	22,241,561	GCCTTTGTTA	G	0.010	0.013	0.139	0.781	0.168	0.562	1.085	0.692	Imputed
rs114568494	1	22,241,660	A	G	0.015	0.018	0.134	0.809	0.140	0.615	1.065	0.007	Imputed
rs35316462	1	22,242,312	A	G	0.015	0.014	0.802	1.036	0.141	0.785	1.367	0.764	Imputed
chr1:22242558:I	1	22,242,558	GA	G	0.138	0.129	0.134	1.077	0.049	0.978	1.187	0.379	Imputed
rs7524618	1	22,242,761	A	G	0.243	0.232	0.107	1.066	0.040	0.986	1.152	0.752	Imputed
rs71637002	1	22,242,820	T	C	0.015	0.014	0.778	1.041	0.141	0.789	1.373	0.751	Imputed
rs35639774	1	22,243,722	G	T	0.009	0.010	0.491	0.884	0.179	0.623	1.255	0.833	Imputed
rs6698486	1	22,244,023	T	C	0.256	0.244	0.086	1.069	0.039	0.991	1.153	0.795	Imputed
rs9426781	1	22,244,592	C	T	0.391	0.380	0.135	1.053	0.035	0.984	1.127	0.760	Imputed
rs77506000	1	22,244,732	T	C	0.017	0.015	0.569	1.079	0.133	0.831	1.401	0.827	Imputed
rs76922915	1	22,244,755	T	C	0.015	0.014	0.778	1.041	0.141	0.789	1.373	0.751	Imputed
rs67932885	1	22,244,923	G	A	0.242	0.230	0.070	1.074	0.040	0.994	1.161	0.643	Imputed
rs4233277	1	22,245,756	T	C	0.390	0.379	0.148	1.051	0.035	0.982	1.126	0.655	Imputed
rs12039740	1	22,245,850	T	C	0.102	0.104	0.765	0.984	0.056	0.882	1.097	0.558	Imputed
rs12759561	1	22,247,090	A	G	0.015	0.014	0.648	1.067	0.142	0.808	1.408	0.789	Imputed

rs12131384	1	22,247,498	C	T	0.394	0.380	0.071	1.065	0.035	0.995	1.139	0.916	Imputed
rs10917064	1	22,247,719	G	T	0.233	0.224	0.148	1.060	0.040	0.980	1.146	0.466	Imputed
rs35542927	1	22,248,047	T	G	0.015	0.014	0.648	1.067	0.142	0.808	1.408	0.789	Imputed
rs114945908	1	22,248,241	A	G	0.019	0.017	0.435	1.103	0.126	0.863	1.411	0.654	Imputed
rs6661141	1	22,248,466	T	C	0.342	0.331	0.123	1.057	0.036	0.985	1.133	0.943	Imputed
rs115702407	1	22,248,738	A	G	0.019	0.017	0.435	1.103	0.126	0.863	1.411	0.654	Imputed
rs10799719	1	22,248,881	G	A	0.389	0.376	0.084	1.062	0.035	0.992	1.137	0.909	Imputed
rs11580829	1	22,248,903	C	G	0.016	0.014	0.506	1.095	0.136	0.838	1.430	0.849	Imputed
rs9426783	1	22,249,057	C	T	0.237	0.227	0.123	1.063	0.040	0.983	1.150	0.476	Imputed
rs35304121	1	22,249,307	A	C	0.015	0.014	0.682	1.060	0.142	0.803	1.398	0.809	Imputed
rs11579545	1	22,249,333	T	C	0.391	0.376	0.057	1.068	0.035	0.998	1.144	0.890	Imputed
rs34600791	1	22,249,344	G	C	0.015	0.014	0.682	1.060	0.142	0.803	1.398	0.809	Imputed
chr1:22249533:D	1	22,249,533	CAT	C	0.388	0.377	0.138	1.053	0.035	0.984	1.127	0.896	Imputed
chr1:22249534:D	1	22,249,534	ATT	A	0.382	0.370	0.102	1.059	0.035	0.989	1.134	0.899	Imputed
rs9426785	1	22,249,589	G	A	0.353	0.342	0.134	1.054	0.035	0.984	1.130	0.671	Imputed
rs9426677	1	22,249,836	T	C	0.251	0.240	0.128	1.061	0.039	0.983	1.146	0.599	Imputed
rs4626854	1	22,250,029	G	C	0.235	0.224	0.109	1.066	0.040	0.986	1.153	0.433	Imputed
rs35230410	1	22,250,558	C	G	0.015	0.014	0.682	1.060	0.142	0.803	1.398	0.809	Imputed
rs9426786	1	22,251,260	T	C	0.250	0.239	0.114	1.064	0.039	0.985	1.149	0.518	Imputed
rs7512059	1	22,251,607	T	C	0.019	0.017	0.350	1.124	0.125	0.880	1.435	0.662	Imputed
rs9426788	1	22,251,891	T	C	0.250	0.239	0.122	1.062	0.039	0.984	1.147	0.506	Imputed
rs4654773	1	22,252,262	A	G	0.142	0.140	0.587	1.027	0.048	0.934	1.129	0.273	Imputed
rs78318011	1	22,252,415	T	C	0.255	0.242	0.068	1.073	0.039	0.995	1.159	0.483	Imputed
rs12748891	1	22,252,529	T	G	0.250	0.239	0.121	1.063	0.039	0.984	1.147	0.508	Imputed
rs35210982	1	22,252,939	A	G	0.015	0.014	0.682	1.060	0.142	0.803	1.398	0.809	Imputed
rs4989428	1	22,253,062	C	A	0.259	0.248	0.107	1.064	0.039	0.987	1.148	0.862	Imputed
chr1:22254026:D	1	22,254,026	AC	A	0.012	0.013	0.710	0.945	0.154	0.698	1.278	0.039	Imputed
rs112663661	1	22,254,088	T	C	0.019	0.017	0.336	1.127	0.124	0.884	1.437	0.779	Imputed
rs116452354	1	22,254,558	G	A	0.010	0.010	0.766	0.950	0.173	0.677	1.332	0.396	Imputed
rs13374960	1	22,254,648	G	T	0.019	0.017	0.343	1.125	0.124	0.882	1.434	0.773	Imputed
rs142831215	1	22,255,484	T	C	0.019	0.017	0.343	1.125	0.124	0.882	1.434	0.773	Imputed
rs34857065	1	22,255,499	T	C	0.234	0.224	0.121	1.064	0.040	0.984	1.151	0.520	Imputed
rs139561469	1	22,255,522	T	G	0.019	0.017	0.343	1.125	0.124	0.882	1.434	0.773	Imputed
rs35970964	1	22,255,754	T	C	0.236	0.225	0.106	1.067	0.040	0.986	1.154	0.471	Imputed
rs6697265	1	22,256,725	C	G	0.237	0.225	0.069	1.075	0.040	0.994	1.163	0.560	Imputed
rs72662467	1	22,256,814	A	G	0.028	0.027	0.619	1.053	0.103	0.860	1.288	0.259	Imputed
rs12033039	1	22,257,202	T	G	0.102	0.103	0.909	0.994	0.056	0.891	1.109	0.789	Imputed
rs6658920	1	22,257,739	C	T	0.395	0.381	0.051	1.070	0.035	1.000	1.145	0.747	Imputed
rs35077760	1	22,257,769	T	C	0.015	0.014	0.671	1.062	0.142	0.805	1.402	0.802	Imputed
rs146833986	1	22,258,134	A	C	0.015	0.014	0.671	1.062	0.142	0.805	1.402	0.802	Imputed

rs12129809	1	22,258,599	G	C	0.395	0.380	0.051	1.070	0.035	1.000	1.145	0.725	Imputed
rs12034979	1	22,259,146	A	G	0.102	0.103	0.884	0.992	0.056	0.889	1.107	0.721	Imputed
rs17467346	1	22,260,012	A	G	0.042	0.045	0.493	0.944	0.084	0.801	1.113	0.754	Imputed
rs11587857	1	22,260,308	C	T	0.247	0.235	0.074	1.073	0.039	0.993	1.159	0.755	Imputed
rs35620292	1	22,260,421	A	G	0.015	0.014	0.671	1.062	0.142	0.805	1.402	0.802	Imputed
rs6672273	1	22,261,339	C	G	0.380	0.364	0.031	1.078	0.035	1.007	1.155	0.662	Imputed
rs6684979	1	22,261,395	G	A	0.041	0.046	0.096	0.868	0.085	0.734	1.026	0.876	Imputed
rs35337208	1	22,261,544	A	C	0.238	0.226	0.069	1.075	0.040	0.994	1.162	0.539	Imputed
rs71638803	1	22,261,841	A	G	0.015	0.014	0.671	1.062	0.142	0.805	1.402	0.802	Imputed
rs7541238	1	22,261,918	A	C	0.035	0.033	0.313	1.097	0.092	0.916	1.313	0.330	Imputed
rs7529613	1	22,261,919	C	A	0.047	0.047	0.916	1.009	0.080	0.862	1.180	0.435	Imputed
rs12081298	1	22,262,225	A	G	0.109	0.110	0.972	0.998	0.054	0.898	1.110	0.461	Imputed
rs78570036	1	22,262,504	A	G	0.102	0.103	0.884	0.992	0.056	0.889	1.107	0.721	Imputed
rs35901947	1	22,262,870	A	G	0.015	0.014	0.671	1.062	0.142	0.805	1.402	0.802	Imputed
rs71514204	1	22,263,551	A	G	0.242	0.232	0.111	1.065	0.040	0.986	1.151	0.968	Imputed
rs192259893	1	22,263,913	C	G	0.075	0.075	0.899	1.008	0.064	0.889	1.144	0.968	Imputed
rs184332240	1	22,263,977	A	C	0.015	0.013	0.340	1.142	0.139	0.870	1.501	0.489	Imputed
chr1:22263988:D	1	22,263,988	CT	C	0.385	0.367	0.020	1.084	0.035	1.013	1.161	0.876	Imputed
rs190446136	1	22,264,019	T	C	0.035	0.041	0.118	0.868	0.091	0.726	1.037	0.742	Imputed
rs4469694	1	22,264,610	G	T	0.225	0.214	0.090	1.071	0.041	0.989	1.160	0.403	Imputed
rs184335096	1	22,264,614	G	T	0.021	0.019	0.303	1.130	0.119	0.895	1.427	0.779	Imputed
rs10917067	1	22,265,293	G	A	0.395	0.381	0.055	1.069	0.035	0.999	1.144	0.760	Imputed
rs79899643	1	22,265,351	A	G	0.102	0.103	0.942	0.996	0.056	0.893	1.111	0.692	Imputed
rs115979533	1	22,266,330	G	C	0.021	0.018	0.271	1.140	0.119	0.903	1.440	0.704	Imputed
rs12733606	1	22,267,007	C	G	0.018	0.019	0.862	0.979	0.126	0.765	1.252	0.299	Imputed
rs80092743	1	22,267,048	A	T	0.102	0.103	0.942	0.996	0.056	0.893	1.111	0.692	Imputed
rs12735933	1	22,267,684	T	C	0.015	0.014	0.659	1.064	0.142	0.806	1.405	0.795	Imputed
rs12035401	1	22,267,926	G	A	0.100	0.103	0.622	0.973	0.057	0.871	1.086	0.606	Imputed
rs4076760	1	22,267,966	T	C	0.237	0.226	0.076	1.073	0.040	0.993	1.161	0.483	Imputed
rs182609273	1	22,268,118	T	C	0.017	0.018	0.645	0.942	0.130	0.730	1.216	0.692	Imputed
rs76718639	1	22,268,692	G	C	0.102	0.103	0.871	0.991	0.056	0.888	1.106	0.727	Imputed
rs34697800	1	22,269,250	G	C	0.015	0.014	0.569	1.083	0.141	0.822	1.427	0.743	Imputed
rs12566023	1	22,269,260	C	T	0.237	0.226	0.084	1.071	0.040	0.991	1.158	0.507	Imputed
rs151077008	1	22,269,485	C	T	0.019	0.017	0.366	1.119	0.124	0.877	1.427	0.754	Imputed
rs6656791	1	22,269,559	G	A	0.373	0.362	0.117	1.056	0.035	0.986	1.131	0.889	Imputed
chr1:22270241:I	1	22,270,241	A	AAAAC	0.403	0.390	0.076	1.063	0.035	0.994	1.138	0.784	Imputed
chr1:22270290:D	1	22,270,290	ATTTC	A	0.372	0.360	0.089	1.061	0.035	0.991	1.137	0.652	Imputed
chr1:22270299:D	1	22,270,299	TTTC	T	0.378	0.364	0.059	1.068	0.035	0.998	1.144	0.394	Imputed
rs74656569	1	22,270,390	C	G	0.238	0.226	0.085	1.071	0.040	0.991	1.158	0.492	Imputed
rs79230226	1	22,270,766	T	C	0.102	0.103	0.885	0.992	0.056	0.889	1.107	0.742	Imputed

rs6688119	1	22,270,907	C	T	0.395	0.380	0.046	1.072	0.035	1.001	1.147	0.694	Imputed
rs10753521	1	22,271,228	T	C	0.363	0.351	0.092	1.061	0.035	0.990	1.137	0.995	Imputed
rs111887321	1	22,271,579	T	C	0.106	0.108	0.738	0.982	0.055	0.882	1.093	0.468	Imputed
rs7544500	1	22,271,839	C	T	0.371	0.359	0.107	1.058	0.035	0.988	1.133	0.779	Genotyped
rs112855015	1	22,272,266	A	T	0.020	0.021	0.668	0.949	0.122	0.748	1.205	0.670	Imputed
rs71638804	1	22,272,323	A	G	0.015	0.014	0.569	1.083	0.141	0.822	1.427	0.743	Imputed
rs35631798	1	22,272,624	T	C	0.238	0.226	0.086	1.071	0.040	0.990	1.158	0.489	Imputed
rs190963274	1	22,272,872	A	C	0.011	0.013	0.415	0.879	0.159	0.643	1.201	0.397	Imputed
rs9728140	1	22,272,913	G	A	0.376	0.365	0.124	1.055	0.035	0.985	1.130	0.667	Imputed
rs35601247	1	22,272,915	A	G	0.016	0.016	0.755	1.042	0.133	0.803	1.354	0.406	Imputed
rs12567706	1	22,273,045	A	G	0.238	0.226	0.086	1.071	0.040	0.991	1.158	0.491	Imputed
rs11576772	1	22,273,292	T	C	0.083	0.084	0.958	1.003	0.061	0.890	1.131	0.804	Imputed
rs4077296	1	22,273,648	G	T	0.371	0.359	0.097	1.060	0.035	0.990	1.135	0.760	Imputed
rs16826074	1	22,273,833	T	C	0.102	0.103	0.891	0.992	0.056	0.889	1.107	0.739	Imputed
rs61776286	1	22,274,057	C	T	0.371	0.359	0.091	1.061	0.035	0.991	1.136	0.748	Imputed
rs61776287	1	22,274,061	C	T	0.391	0.377	0.058	1.068	0.035	0.998	1.143	0.802	Imputed
rs4565679	1	22,274,172	C	T	0.371	0.359	0.091	1.061	0.035	0.991	1.136	0.748	Imputed
rs4488002	1	22,274,178	G	A	0.371	0.359	0.088	1.062	0.035	0.991	1.137	0.741	Imputed
rs4593792	1	22,274,205	A	G	0.238	0.226	0.086	1.071	0.040	0.990	1.158	0.490	Imputed
rs4393123	1	22,274,510	T	C	0.238	0.226	0.076	1.073	0.040	0.993	1.161	0.510	Imputed
chr1:22274668:I	1	22,274,668	C	CTG	0.375	0.363	0.110	1.057	0.035	0.987	1.133	0.644	Imputed
rs11587362	1	22,274,691	T	C	0.375	0.362	0.077	1.064	0.035	0.993	1.139	0.646	Imputed
chr1:22274704:I	1	22,274,704	CA	C	0.013	0.015	0.313	0.860	0.150	0.641	1.154	0.586	Imputed
rs71638805	1	22,274,729	A	C	0.015	0.014	0.580	1.081	0.141	0.821	1.424	0.750	Imputed
rs6666534	1	22,275,646	A	C	0.371	0.359	0.088	1.062	0.035	0.991	1.137	0.755	Imputed
rs12025633	1	22,276,058	T	C	0.102	0.103	0.918	0.994	0.056	0.891	1.109	0.747	Imputed
rs12025807	1	22,276,637	C	G	0.254	0.242	0.077	1.071	0.039	0.993	1.156	0.969	Imputed
rs12046762	1	22,276,686	G	A	0.254	0.242	0.077	1.071	0.039	0.993	1.156	0.969	Imputed
rs12741423	1	22,276,814	C	A	0.238	0.226	0.084	1.071	0.040	0.991	1.158	0.502	Imputed
rs10493010	1	22,277,184	C	T	0.102	0.103	0.922	0.995	0.056	0.891	1.110	0.745	Imputed
rs79689468	1	22,277,532	C	G	0.102	0.103	0.922	0.995	0.056	0.891	1.110	0.745	Imputed
rs77655576	1	22,277,659	G	T	0.010	0.012	0.643	0.927	0.165	0.671	1.280	0.338	Imputed
rs34372058	1	22,278,138	T	C	0.028	0.025	0.304	1.111	0.103	0.908	1.359	0.330	Imputed
rs35407091	1	22,278,186	T	C	0.238	0.226	0.073	1.074	0.040	0.993	1.161	0.524	Imputed
rs10917069	1	22,278,426	C	T	0.117	0.118	0.972	0.998	0.053	0.901	1.106	0.231	Imputed
rs10917070	1	22,278,790	G	A	0.117	0.118	0.968	0.998	0.053	0.900	1.106	0.232	Imputed
rs7354857	1	22,279,191	A	G	0.117	0.118	0.964	0.998	0.053	0.900	1.106	0.233	Imputed
rs12118516	1	22,279,260	A	C	0.058	0.057	0.993	0.999	0.073	0.867	1.152	0.951	Imputed
rs7355161	1	22,279,280	C	A	0.117	0.118	0.964	0.998	0.053	0.900	1.106	0.233	Imputed
rs6673001	1	22,279,626	A	G	0.019	0.017	0.575	1.073	0.125	0.839	1.371	0.771	Imputed

rs12723814	1	22,280,036	G	T	0.015	0.014	0.580	1.081	0.141	0.821	1.424	0.750	Imputed
rs12567548	1	22,280,365	C	T	0.241	0.229	0.066	1.076	0.040	0.995	1.163	0.453	Imputed
rs6426729	1	22,280,382	T	C	0.117	0.118	0.964	0.998	0.053	0.900	1.106	0.233	Imputed
rs6676266	1	22,280,415	A	G	0.019	0.018	0.624	1.063	0.125	0.832	1.359	0.741	Imputed
rs7533718	1	22,281,393	G	A	0.137	0.138	0.927	0.996	0.049	0.904	1.096	0.160	Imputed
rs10917071	1	22,281,479	G	A	0.120	0.122	0.863	0.991	0.052	0.895	1.097	0.147	Imputed
rs10917072	1	22,281,664	G	A	0.117	0.118	0.894	0.993	0.053	0.896	1.101	0.212	Imputed
rs10917073	1	22,281,690	A	G	0.117	0.118	0.894	0.993	0.053	0.896	1.101	0.212	Imputed
rs12140171	1	22,281,718	T	A	0.117	0.118	0.894	0.993	0.053	0.896	1.101	0.212	Imputed
rs10917074	1	22,281,759	G	A	0.117	0.118	0.889	0.993	0.053	0.896	1.100	0.204	Genotyped
rs12748739	1	22,281,813	G	C	0.242	0.229	0.060	1.077	0.040	0.997	1.164	0.455	Imputed
rs78538821	1	22,281,927	T	C	0.101	0.103	0.788	0.985	0.056	0.883	1.099	0.633	Imputed
rs75577500	1	22,282,086	T	C	0.015	0.013	0.474	1.106	0.141	0.839	1.457	0.738	Imputed
rs4406609	1	22,282,117	C	A	0.117	0.119	0.878	0.992	0.053	0.895	1.100	0.214	Imputed
rs75571160	1	22,282,249	T	C	0.102	0.103	0.857	0.990	0.056	0.887	1.105	0.600	Imputed
rs7529220	1	22,282,619	T	C	0.136	0.136	0.889	1.007	0.049	0.914	1.109	0.300	Genotyped
rs79932633	1	22,282,824	A	C	0.102	0.103	0.829	0.988	0.056	0.886	1.102	0.522	Imputed
rs12754385	1	22,282,920	G	C	0.246	0.234	0.088	1.070	0.039	0.990	1.155	0.671	Imputed
rs12122512	1	22,282,938	C	T	0.400	0.384	0.027	1.080	0.035	1.009	1.155	0.673	Imputed
rs6662815	1	22,283,172	C	T	0.111	0.113	0.885	0.992	0.054	0.893	1.102	0.292	Imputed
rs35760029	1	22,283,380	C	T	0.015	0.013	0.464	1.108	0.141	0.841	1.461	0.732	Imputed
rs6675669	1	22,283,583	A	G	0.118	0.119	0.961	0.997	0.052	0.900	1.105	0.242	Imputed
rs10917075	1	22,283,671	C	T	0.392	0.374	0.018	1.086	0.035	1.014	1.162	0.805	Imputed
rs10917076	1	22,283,735	C	T	0.396	0.377	0.012	1.091	0.035	1.019	1.167	0.783	Imputed
rs6689414	1	22,283,845	C	G	0.265	0.246	9.81E-03	1.104	0.038	1.024	1.191	0.609	Imputed
rs4655001	1	22,283,991	C	A	0.112	0.114	0.832	0.989	0.054	0.890	1.098	0.292	Genotyped
rs36106877	1	22,284,155	T	C	0.015	0.013	0.464	1.108	0.141	0.841	1.461	0.732	Imputed
rs6426730	1	22,284,463	T	A	0.013	0.013	0.787	1.041	0.150	0.777	1.396	0.278	Imputed
rs11589754	1	22,284,495	G	T	0.259	0.241	9.60E-03	1.106	0.039	1.025	1.193	0.458	Genotyped
rs6681966	1	22,284,708	T	C	0.011	0.011	0.637	1.078	0.161	0.787	1.477	0.064	Genotyped
rs4501814	1	22,285,357	G	T	0.101	0.103	0.817	0.987	0.056	0.885	1.102	0.599	Imputed
rs36070253	1	22,285,529	A	G	0.015	0.013	0.614	1.074	0.142	0.814	1.418	0.712	Imputed
rs112529471	1	22,285,712	C	A	0.101	0.103	0.786	0.985	0.056	0.883	1.099	0.613	Imputed
rs7539092	1	22,286,061	A	G	0.112	0.114	0.888	0.993	0.054	0.894	1.102	0.276	Imputed
rs68065177	1	22,286,607	T	C	0.261	0.241	4.69E-03	1.116	0.039	1.034	1.203	0.417	Imputed
rs36068757	1	22,286,707	G	T	0.259	0.241	9.79E-03	1.105	0.039	1.024	1.193	0.378	Imputed
rs112034183	1	22,287,082	A	G	0.047	0.047	0.844	1.016	0.080	0.869	1.188	0.965	Imputed
rs112962027	1	22,287,201	T	C	0.107	0.106	0.750	1.018	0.055	0.914	1.133	0.463	Imputed
chr1:22287335:I	1	22,287,335	AG	A	0.107	0.106	0.779	1.015	0.055	0.912	1.131	0.508	Imputed
rs61776316	1	22,287,510	C	A	0.029	0.034	0.081	0.840	0.100	0.691	1.022	0.920	Imputed

rs190563633	1	22,287,544	T	C	0.010	0.012	0.598	0.917	0.166	0.663	1.268	0.815	Imputed
rs143684062	1	22,287,607	T	C	0.009	0.012	0.073	0.726	0.178	0.512	1.029	0.022	Imputed
rs35182048	1	22,287,622	C	T	0.032	0.029	0.292	1.107	0.097	0.916	1.339	0.488	Imputed
rs11588597	1	22,287,632	C	T	0.255	0.237	9.97E-03	1.106	0.039	1.024	1.194	0.590	Imputed
rs11589871	1	22,287,667	T	G	0.033	0.031	0.473	1.070	0.095	0.889	1.288	0.320	Imputed
rs11578663	1	22,287,674	T	C	0.031	0.029	0.495	1.069	0.098	0.882	1.296	0.521	Imputed
rs11588615	1	22,287,689	C	T	0.038	0.033	0.173	1.129	0.089	0.948	1.346	0.320	Imputed
rs10917077	1	22,287,730	G	A	0.363	0.344	0.011	1.094	0.035	1.021	1.173	0.876	Imputed
rs17427572	1	22,287,866	T	C	0.057	0.064	0.117	0.892	0.073	0.774	1.029	0.809	Imputed
rs12410694	1	22,287,941	A	G	0.260	0.237	1.20E-03	1.134	0.039	1.051	1.223	0.512	Imputed
rs114768142	1	22,288,375	A	G	0.012	0.014	0.302	0.853	0.154	0.630	1.155	0.681	Imputed
chr1:22288394:I	1	22,288,394	TA	T	0.361	0.342	0.011	1.094	0.035	1.021	1.172	0.957	Imputed
rs12410759	1	22,288,468	A	G	0.259	0.237	1.27E-03	1.133	0.039	1.050	1.222	0.493	Imputed
rs75508464	1	22,288,656	C	T	0.070	0.073	0.707	0.976	0.066	0.857	1.110	0.633	Imputed
chr1:22289253:D	1	22,289,253	G	GT	0.098	0.100	0.806	0.986	0.057	0.882	1.102	0.311	Imputed
rs12740881	1	22,289,672	G	A	0.357	0.337	6.40E-03	1.101	0.035	1.027	1.180	0.989	Imputed
rs55689106	1	22,289,842	G	A	0.357	0.337	6.92E-03	1.100	0.035	1.027	1.179	0.916	Imputed
rs189125017	1	22,289,920	C	A	0.030	0.035	0.092	0.848	0.098	0.700	1.028	0.222	Imputed
rs55997695	1	22,289,945	T	G	0.259	0.237	1.40E-03	1.132	0.039	1.049	1.221	0.498	Imputed
rs8179386	1	22,290,110	G	A	0.357	0.337	6.23E-03	1.102	0.035	1.028	1.181	0.992	Imputed
rs8179387	1	22,290,156	G	A	0.260	0.237	9.93E-04	1.136	0.039	1.053	1.226	0.484	Genotyped
rs72662482	1	22,290,379	G	C	0.259	0.237	1.44E-03	1.132	0.039	1.049	1.221	0.495	Imputed
rs72662484	1	22,290,429	T	C	0.259	0.237	1.40E-03	1.132	0.039	1.049	1.221	0.498	Imputed
rs35806133	1	22,290,623	T	C	0.259	0.237	1.40E-03	1.132	0.039	1.049	1.221	0.498	Imputed
rs34277514	1	22,290,630	A	G	0.357	0.337	6.23E-03	1.102	0.035	1.028	1.181	0.992	Imputed
rs12730501	1	22,290,929	A	C	0.259	0.237	1.40E-03	1.132	0.039	1.049	1.221	0.498	Imputed
rs114158976	1	22,291,122	A	G	0.012	0.011	0.506	1.109	0.156	0.818	1.504	0.918	Imputed
rs35963112	1	22,291,226	T	C	0.259	0.237	1.44E-03	1.132	0.039	1.049	1.221	0.495	Imputed
rs142797632	1	22,291,841	A	G	0.012	0.014	0.302	0.853	0.154	0.630	1.155	0.681	Imputed
rs183394817	1	22,291,880	C	G	0.009	0.012	0.130	0.762	0.179	0.537	1.081	0.010	Imputed
rs11590712	1	22,291,939	C	A	0.259	0.237	1.32E-03	1.133	0.039	1.050	1.222	0.489	Imputed
rs11591127	1	22,292,018	C	T	0.259	0.237	1.53E-03	1.131	0.039	1.048	1.220	0.516	Imputed
rs187351243	1	22,292,098	A	G	0.017	0.018	0.563	0.927	0.132	0.715	1.200	0.719	Imputed
rs150498744	1	22,292,207	T	C	0.012	0.014	0.302	0.853	0.154	0.630	1.155	0.681	Imputed
rs111567686	1	22,292,231	T	C	0.066	0.067	0.935	0.995	0.068	0.870	1.137	0.429	Imputed
rs6699127	1	22,292,266	G	A	0.385	0.363	3.68E-03	1.106	0.035	1.033	1.184	0.320	Imputed
chr1:22292449:D	1	22,292,449	A	AG	0.257	0.235	1.52E-03	1.131	0.039	1.048	1.221	0.612	Imputed
rs10917078	1	22,292,517	A	G	0.259	0.237	1.44E-03	1.131	0.039	1.049	1.221	0.495	Imputed
rs10799720	1	22,292,657	C	T	0.259	0.237	1.42E-03	1.132	0.039	1.049	1.221	0.496	Imputed
rs113807400	1	22,292,785	C	T	0.066	0.067	0.935	0.995	0.068	0.870	1.137	0.429	Imputed

rs12740670	1	22,293,056	T	G	0.048	0.044	0.272	1.091	0.079	0.934	1.274	0.618	Imputed
rs7533101	1	22,293,275	T	C	0.259	0.238	2.00E-03	1.127	0.039	1.045	1.216	0.476	Imputed
rs7554864	1	22,293,301	T	G	0.259	0.237	1.46E-03	1.131	0.039	1.049	1.221	0.493	Imputed
rs7547232	1	22,293,527	C	T	0.259	0.237	1.46E-03	1.131	0.039	1.049	1.221	0.493	Imputed
rs11576231	1	22,293,723	C	A	0.259	0.237	1.45E-03	1.131	0.039	1.049	1.221	0.494	Imputed
rs12745683	1	22,293,897	A	G	0.255	0.233	1.94E-03	1.128	0.039	1.045	1.218	0.584	Imputed
rs138920950	1	22,294,102	C	A	0.086	0.087	0.711	0.978	0.061	0.868	1.101	0.870	Imputed
rs79862129	1	22,294,244	C	T	0.024	0.022	0.701	1.044	0.112	0.838	1.301	0.811	Imputed
rs186275588	1	22,294,396	T	C	0.012	0.014	0.282	0.847	0.154	0.626	1.147	0.648	Imputed
rs6698084	1	22,294,971	A	C	0.387	0.365	3.96E-03	1.105	0.035	1.033	1.183	0.372	Imputed
rs11578958	1	22,295,098	A	G	0.259	0.237	1.42E-03	1.132	0.039	1.049	1.221	0.427	Imputed
rs12061483	1	22,295,152	G	T	0.013	0.016	0.224	0.837	0.147	0.628	1.116	0.545	Imputed
rs148268474	1	22,295,551	A	G	0.009	0.012	0.126	0.760	0.179	0.535	1.078	0.009	Imputed
rs7539859	1	22,295,701	C	T	0.387	0.365	3.77E-03	1.106	0.035	1.033	1.184	0.368	Imputed
rs11583492	1	22,296,196	T	C	0.259	0.237	1.42E-03	1.132	0.039	1.049	1.221	0.427	Imputed
rs141723072	1	22,296,250	G	C	0.009	0.011	0.362	0.853	0.175	0.606	1.202	0.256	Imputed
rs10917079	1	22,296,307	A	C	0.259	0.237	1.33E-03	1.132	0.039	1.050	1.222	0.457	Genotyped
rs116176850	1	22,296,343	A	G	0.012	0.014	0.288	0.849	0.154	0.628	1.149	0.643	Imputed
rs10917080	1	22,296,373	C	G	0.019	0.015	0.098	1.229	0.126	0.960	1.573	0.009	Imputed
rs10917081	1	22,296,375	A	G	0.019	0.015	0.105	1.224	0.126	0.956	1.567	0.009	Imputed
rs7511851	1	22,296,627	C	T	0.011	0.010	0.375	1.152	0.161	0.840	1.579	0.015	Imputed
rs149423999	1	22,296,676	T	C	0.057	0.060	0.584	0.961	0.073	0.834	1.108	0.880	Imputed
rs111651468	1	22,296,701	T	C	0.085	0.087	0.795	0.984	0.061	0.874	1.108	0.442	Imputed
rs10917082	1	22,296,758	T	G	0.216	0.203	0.036	1.090	0.041	1.006	1.182	0.656	Imputed
rs10917083	1	22,296,773	G	A	0.356	0.343	0.071	1.066	0.036	0.995	1.143	0.791	Imputed
rs4655002	1	22,296,937	T	G	0.249	0.228	2.77E-03	1.125	0.039	1.041	1.215	0.339	Imputed
rs76930762	1	22,296,961	C	T	0.247	0.226	2.83E-03	1.125	0.039	1.041	1.215	0.431	Imputed
rs12745005	1	22,296,962	C	G	0.251	0.231	4.17E-03	1.119	0.039	1.036	1.208	0.553	Imputed
rs57030248	1	22,296,994	T	C	0.062	0.064	0.622	0.966	0.070	0.842	1.108	0.685	Imputed
rs12745053	1	22,297,025	A	G	0.258	0.236	1.58E-03	1.131	0.039	1.048	1.220	0.519	Imputed
rs59134693	1	22,297,114	G	A	0.067	0.069	0.789	0.982	0.067	0.861	1.121	0.432	Imputed
rs10917084	1	22,298,008	T	C	0.264	0.241	1.78E-03	1.128	0.039	1.046	1.217	0.386	Imputed
rs12144125	1	22,298,039	A	G	0.012	0.011	0.276	1.180	0.153	0.874	1.594	0.046	Imputed
rs113385886	1	22,298,241	C	T	0.068	0.069	0.843	0.987	0.067	0.865	1.126	0.411	Imputed
rs6661287	1	22,298,481	C	T	0.056	0.059	0.429	0.943	0.074	0.816	1.090	0.954	Imputed
rs34547676	1	22,298,526	T	C	0.248	0.226	9.93E-04	1.138	0.039	1.054	1.230	0.426	Imputed
rs75386540	1	22,298,546	C	T	0.218	0.201	9.15E-03	1.113	0.041	1.027	1.207	0.357	Imputed
rs76270008	1	22,298,548	G	A	0.216	0.200	0.016	1.104	0.041	1.018	1.198	0.297	Imputed
rs144691985	1	22,298,602	T	C	0.012	0.014	0.282	0.847	0.154	0.626	1.147	0.648	Imputed
rs34080449	1	22,298,773	C	T	0.350	0.329	4.71E-03	1.106	0.036	1.031	1.185	0.991	Imputed

rs188469314	1	22,299,064	T	C	0.066	0.068	0.654	0.970	0.068	0.849	1.108	0.541	Imputed
rs191934270	1	22,299,065	G	A	0.070	0.072	0.655	0.971	0.066	0.853	1.105	0.369	Imputed
rs56219455	1	22,299,142	G	A	0.348	0.328	7.47E-03	1.100	0.036	1.026	1.179	0.935	Imputed
rs55953638	1	22,299,193	T	C	0.358	0.338	8.80E-03	1.097	0.035	1.024	1.176	0.776	Imputed
rs56337767	1	22,299,393	T	C	0.259	0.237	1.48E-03	1.131	0.039	1.048	1.220	0.435	Imputed
rs4466619	1	22,299,523	T	G	0.037	0.037	0.978	1.002	0.089	0.842	1.194	0.008	Imputed
rs4233282	1	22,299,656	T	G	0.387	0.366	5.21E-03	1.102	0.035	1.029	1.180	0.358	Imputed
rs4233283	1	22,299,966	C	A	0.386	0.365	6.40E-03	1.099	0.035	1.027	1.177	0.387	Imputed
rs143003994	1	22,300,114	T	C	0.012	0.014	0.282	0.847	0.154	0.626	1.147	0.648	Imputed
rs7537004	1	22,300,465	T	A	0.387	0.366	5.46E-03	1.101	0.035	1.029	1.179	0.362	Imputed
rs190195927	1	22,300,599	T	C	0.027	0.027	0.931	1.009	0.103	0.824	1.235	0.288	Imputed
rs34836203	1	22,300,968	G	A	0.240	0.219	2.80E-03	1.127	0.040	1.042	1.218	0.319	Imputed
rs34481877	1	22,301,039	T	C	0.344	0.325	8.60E-03	1.098	0.036	1.024	1.178	0.996	Imputed
rs36117763	1	22,301,165	T	C	0.257	0.235	1.36E-03	1.133	0.039	1.049	1.222	0.484	Imputed
rs35522699	1	22,301,180	A	G	0.266	0.246	4.43E-03	1.116	0.038	1.035	1.203	0.438	Imputed
rs36029501	1	22,301,199	T	C	0.257	0.236	2.02E-03	1.128	0.039	1.045	1.217	0.425	Imputed
rs12029994	1	22,301,419	T	C	0.353	0.332	5.49E-03	1.103	0.035	1.029	1.183	0.918	Imputed
chr1:22301533:D	1	22,301,533	AG	A	0.388	0.367	5.72E-03	1.101	0.035	1.028	1.178	0.362	Imputed
rs4988919	1	22,301,622	A	G	0.242	0.220	1.42E-03	1.135	0.040	1.050	1.227	0.281	Imputed
rs71638808	1	22,301,635	C	T	0.215	0.196	4.87E-03	1.124	0.041	1.036	1.219	0.167	Imputed
rs4988922	1	22,301,701	C	T	0.249	0.227	9.62E-04	1.139	0.039	1.054	1.230	0.460	Imputed
rs6671775	1	22,301,713	C	T	0.378	0.358	6.74E-03	1.099	0.035	1.026	1.177	0.324	Imputed
rs4988924	1	22,301,755	A	G	0.015	0.013	0.332	1.147	0.141	0.870	1.512	0.695	Imputed
rs11577154	1	22,301,769	A	G	0.139	0.136	0.625	1.024	0.049	0.930	1.128	0.846	Imputed
rs11581147	1	22,301,770	T	C	0.136	0.136	0.964	1.002	0.050	0.910	1.104	0.624	Imputed
rs71638809	1	22,301,824	C	T	0.242	0.218	4.48E-04	1.150	0.040	1.064	1.243	0.338	Imputed
rs11591033	1	22,302,082	C	T	0.081	0.080	0.781	1.017	0.062	0.901	1.148	0.396	Imputed
rs10917087	1	22,302,427	G	C	0.357	0.335	3.15E-03	1.110	0.035	1.036	1.190	0.953	Imputed
rs11581262	1	22,302,509	T	C	0.095	0.095	0.955	1.003	0.058	0.896	1.123	0.195	Imputed
rs34196101	1	22,302,659	T	C	0.027	0.023	0.261	1.127	0.107	0.915	1.389	0.640	Imputed
rs12031846	1	22,302,783	A	C	0.262	0.238	8.12E-04	1.138	0.039	1.055	1.228	0.387	Imputed
rs11576396	1	22,303,024	C	T	0.096	0.097	0.932	0.995	0.057	0.890	1.113	0.179	Imputed
rs7554039	1	22,303,188	A	G	0.258	0.236	1.39E-03	1.132	0.039	1.049	1.222	0.546	Imputed
rs116802276	1	22,303,465	T	C	0.083	0.084	0.995	1.000	0.061	0.887	1.127	0.527	Imputed
rs4415533	1	22,303,702	A	C	0.357	0.335	3.09E-03	1.110	0.035	1.036	1.190	0.919	Imputed
rs11576963	1	22,304,142	A	T	0.096	0.097	0.987	0.999	0.057	0.893	1.118	0.283	Imputed
rs111512189	1	22,304,229	C	T	0.069	0.069	0.958	0.997	0.067	0.874	1.136	0.570	Imputed
rs57648880	1	22,304,272	G	T	0.360	0.337	3.00E-03	1.110	0.035	1.036	1.190	0.755	Imputed
rs12074015	1	22,304,428	C	G	0.051	0.053	0.731	0.974	0.076	0.839	1.132	0.082	Imputed
rs181658556	1	22,304,431	C	G	0.062	0.064	0.810	0.983	0.070	0.858	1.128	0.860	Imputed

rs12058649	1	22,304,463	A	G	0.099	0.099	0.885	1.008	0.057	0.902	1.127	0.496	Imputed
rs12059804	1	22,304,585	C	T	0.381	0.371	0.177	1.048	0.035	0.979	1.123	0.913	Imputed
rs10917088	1	22,304,712	A	G	0.309	0.289	6.21E-03	1.106	0.037	1.029	1.188	0.874	Imputed
rs143312313	1	22,304,831	A	C	0.047	0.052	0.316	0.924	0.079	0.791	1.079	0.109	Imputed
rs4655004	1	22,305,098	A	G	0.026	0.026	0.888	0.985	0.106	0.800	1.213	0.121	Imputed
rs187706659	1	22,305,128	G	C	0.027	0.027	0.940	1.008	0.103	0.823	1.234	0.291	Imputed
rs190938264	1	22,305,175	G	A	0.027	0.027	0.940	1.008	0.103	0.823	1.234	0.291	Imputed
rs7418481	1	22,305,318	T	C	0.055	0.057	0.577	0.960	0.074	0.831	1.109	0.187	Imputed
rs144783508	1	22,305,626	C	T	0.024	0.018	0.012	1.325	0.112	1.064	1.650	0.510	Imputed
rs74919852	1	22,305,627	A	G	0.024	0.018	0.015	1.312	0.113	1.053	1.636	0.539	Imputed
rs146473048	1	22,305,628	C	T	0.024	0.019	0.015	1.309	0.112	1.052	1.631	0.459	Imputed
rs76783483	1	22,305,629	A	G	0.024	0.018	0.011	1.329	0.112	1.067	1.656	0.499	Imputed
rs79051226	1	22,305,651	T	C	0.039	0.035	0.249	1.107	0.088	0.931	1.315	0.244	Imputed
rs184826684	1	22,305,652	A	G	0.246	0.228	8.29E-03	1.110	0.039	1.027	1.199	0.430	Imputed
rs61776318	1	22,305,708	G	A	0.023	0.023	0.944	1.008	0.112	0.810	1.255	0.160	Imputed
rs61776319	1	22,305,712	T	C	0.022	0.023	0.795	0.971	0.115	0.776	1.215	0.126	Imputed
rs61776320	1	22,305,734	T	C	0.386	0.365	5.49E-03	1.101	0.035	1.029	1.179	0.372	Imputed
rs112174348	1	22,305,791	T	G	0.012	0.015	0.185	0.819	0.151	0.610	1.101	0.483	Imputed
rs4504857	1	22,305,906	T	C	0.012	0.015	0.185	0.819	0.151	0.610	1.101	0.483	Imputed
rs6688236	1	22,305,915	C	T	0.386	0.365	6.04E-03	1.100	0.035	1.028	1.178	0.381	Imputed
rs6700770	1	22,306,009	A	G	0.025	0.025	0.842	0.979	0.109	0.791	1.211	0.153	Imputed
rs6703434	1	22,306,042	T	A	0.021	0.022	0.654	0.949	0.117	0.755	1.194	0.094	Imputed
rs71514207	1	22,306,044	C	T	0.021	0.022	0.654	0.949	0.117	0.755	1.194	0.094	Imputed
rs71514208	1	22,306,051	T	C	0.021	0.021	0.744	0.962	0.118	0.763	1.214	0.115	Imputed
rs4417037	1	22,306,094	T	C	0.013	0.011	0.318	1.161	0.151	0.864	1.559	0.034	Imputed
rs4329480	1	22,306,193	T	C	0.026	0.023	0.228	1.137	0.107	0.923	1.402	0.956	Imputed
rs4520391	1	22,306,208	C	A	0.020	0.022	0.614	0.942	0.119	0.746	1.189	0.085	Imputed
rs138872554	1	22,306,236	T	C	0.067	0.068	0.907	0.992	0.067	0.869	1.132	0.535	Imputed
rs148758365	1	22,306,481	G	A	0.054	0.048	0.104	1.131	0.076	0.975	1.311	0.339	Imputed
chr1:22306507:I	1	22,306,507	A	AC	0.059	0.056	0.361	1.067	0.072	0.928	1.228	0.025	Imputed
rs144937366	1	22,306,533	C	G	0.036	0.040	0.319	0.915	0.089	0.768	1.090	0.022	Imputed
rs148271788	1	22,306,552	C	T	0.041	0.042	0.893	0.989	0.085	0.837	1.167	0.069	Imputed
rs141259626	1	22,306,555	A	G	0.036	0.038	0.625	0.957	0.090	0.802	1.142	0.014	Imputed
rs11580978	1	22,306,809	T	C	0.062	0.061	0.835	1.015	0.070	0.884	1.165	0.483	Imputed
rs4295857	1	22,306,832	C	T	0.030	0.032	0.716	0.965	0.098	0.797	1.169	0.009	Imputed
rs181364686	1	22,306,838	C	G	0.028	0.027	0.884	1.015	0.103	0.830	1.242	0.397	Imputed
rs12401894	1	22,306,949	A	G	0.256	0.235	2.55E-03	1.125	0.039	1.042	1.214	0.304	Imputed
rs4412569	1	22,306,955	A	G	0.032	0.037	0.102	0.856	0.095	0.711	1.032	0.229	Imputed
rs58363133	1	22,307,003	T	C	0.011	0.010	0.707	1.063	0.164	0.771	1.466	0.025	Imputed
rs58598600	1	22,307,004	A	G	0.011	0.010	0.707	1.063	0.164	0.771	1.466	0.025	Imputed

rs56084150	1	22,307,082	G	C	0.091	0.095	0.490	0.961	0.059	0.857	1.077	0.099	Imputed
rs60580853	1	22,307,189	A	T	0.077	0.078	0.986	1.001	0.063	0.884	1.133	0.092	Imputed
rs112577356	1	22,307,199	C	T	0.025	0.024	0.637	1.053	0.109	0.850	1.305	0.325	Imputed
rs7528110	1	22,307,206	C	A	0.321	0.299	5.28E-03	1.107	0.036	1.031	1.189	0.752	Imputed
rs113430470	1	22,307,216	C	G	0.011	0.014	0.257	0.837	0.157	0.615	1.139	0.516	Imputed
rs12401939	1	22,307,252	A	G	0.257	0.235	2.33E-03	1.126	0.039	1.043	1.215	0.396	Imputed
rs7528228	1	22,307,340	C	T	0.073	0.072	0.932	0.994	0.065	0.875	1.130	0.628	Imputed
rs7528300	1	22,307,358	C	G	0.076	0.076	0.790	0.983	0.064	0.867	1.115	0.907	Imputed
rs72662488	1	22,307,478	A	G	0.012	0.015	0.117	0.786	0.154	0.581	1.063	0.863	Imputed
rs143622107	1	22,307,680	T	C	0.031	0.033	0.348	0.912	0.098	0.753	1.105	0.988	Imputed
rs6689384	1	22,307,743	T	C	0.026	0.026	0.973	0.996	0.106	0.810	1.226	0.087	Imputed
rs6691837	1	22,307,757	T	A	0.034	0.031	0.301	1.101	0.094	0.917	1.323	0.150	Imputed
rs28497722	1	22,307,763	A	G	0.051	0.053	0.841	0.985	0.077	0.847	1.145	0.630	Imputed
rs7552725	1	22,307,875	G	A	0.020	0.021	0.554	0.932	0.120	0.736	1.179	0.104	Imputed
rs7531316	1	22,308,160	C	T	0.020	0.021	0.554	0.932	0.120	0.736	1.179	0.104	Imputed
rs79366202	1	22,308,165	T	G	0.013	0.016	0.258	0.849	0.145	0.638	1.129	0.559	Imputed
rs7553024	1	22,308,178	G	A	0.022	0.022	0.822	0.975	0.115	0.777	1.222	0.292	Genotyped
rs111402056	1	22,308,184	A	G	0.028	0.027	0.884	1.015	0.103	0.830	1.242	0.397	Imputed
rs7542953	1	22,308,213	A	G	0.020	0.021	0.555	0.932	0.120	0.736	1.179	0.104	Imputed
rs184890506	1	22,308,363	T	C	0.012	0.014	0.177	0.810	0.156	0.596	1.099	0.272	Imputed
rs12759318	1	22,308,435	G	A	0.030	0.033	0.371	0.914	0.100	0.752	1.112	0.121	Imputed
rs145249052	1	22,308,498	G	A	0.015	0.016	0.678	0.945	0.138	0.721	1.238	0.694	Imputed
rs10799722	1	22,308,516	C	A	0.082	0.092	0.041	0.882	0.062	0.781	0.995	0.694	Imputed
rs12024382	1	22,308,641	G	C	0.058	0.058	0.964	1.003	0.072	0.871	1.156	0.005	Imputed
rs12049408	1	22,308,704	A	G	0.373	0.351	5.53E-03	1.102	0.035	1.029	1.180	0.276	Genotyped
rs10917096	1	22,308,708	G	A	0.020	0.021	0.605	0.940	0.120	0.742	1.190	0.108	Imputed
rs10917097	1	22,308,807	A	G	0.058	0.059	0.991	1.001	0.072	0.869	1.152	0.020	Genotyped
rs111795157	1	22,308,853	T	C	0.013	0.015	0.235	0.838	0.150	0.625	1.123	0.680	Imputed
rs34637160	1	22,308,983	C	T	0.253	0.234	4.95E-03	1.116	0.039	1.034	1.205	0.515	Imputed
rs6426731	1	22,309,296	G	A	0.038	0.042	0.203	0.895	0.088	0.753	1.062	0.659	Imputed
rs4466621	1	22,309,320	G	A	0.033	0.035	0.476	0.935	0.095	0.777	1.125	0.051	Imputed
rs4466622	1	22,309,466	G	T	0.051	0.050	0.795	1.020	0.077	0.877	1.187	0.099	Imputed
rs6426732	1	22,309,690	T	A	0.021	0.021	0.972	0.996	0.118	0.791	1.254	0.205	Imputed
rs6426733	1	22,309,706	A	G	0.045	0.046	0.793	0.979	0.081	0.835	1.148	0.047	Imputed
rs6426734	1	22,309,709	G	A	0.044	0.045	0.895	0.989	0.082	0.843	1.162	0.013	Imputed
rs12121300	1	22,309,805	A	G	0.021	0.021	0.976	1.003	0.118	0.797	1.263	0.001	Imputed
rs12730562	1	22,309,808	G	A	0.254	0.236	7.55E-03	1.110	0.039	1.028	1.198	0.559	Imputed
rs12060528	1	22,309,867	A	T	0.012	0.015	0.179	0.816	0.152	0.605	1.099	0.533	Imputed
rs6698785	1	22,309,874	T	A	0.020	0.021	0.658	0.948	0.120	0.749	1.201	0.100	Imputed
rs6698795	1	22,309,938	T	C	0.039	0.040	0.830	0.982	0.087	0.828	1.164	0.295	Imputed

rs12023741	1	22,309,957	T	C	0.391	0.372	0.012	1.092	0.035	1.020	1.169	0.243	Imputed
rs4468136	1	22,310,127	A	G	0.010	0.011	0.582	0.911	0.168	0.656	1.267	0.331	Imputed
rs10733031	1	22,310,139	T	G	0.030	0.029	0.844	1.020	0.099	0.839	1.239	0.232	Imputed
rs10733032	1	22,310,145	C	T	0.030	0.029	0.844	1.020	0.099	0.839	1.239	0.232	Imputed
rs41266015	1	22,310,190	C	T	0.066	0.066	0.948	1.004	0.068	0.879	1.148	0.847	Imputed
rs10733033	1	22,310,262	G	C	0.031	0.030	0.839	1.020	0.098	0.842	1.236	0.606	Imputed
rs6426735	1	22,310,662	G	T	0.050	0.049	0.870	1.013	0.077	0.870	1.179	0.058	Imputed
rs7538207	1	22,310,674	C	T	0.020	0.021	0.667	0.950	0.120	0.750	1.203	0.098	Imputed
rs3820287	1	22,310,725	G	C	0.050	0.049	0.870	1.013	0.077	0.870	1.179	0.058	Imputed
rs114365157	1	22,310,807	G	A	0.029	0.028	0.629	1.050	0.101	0.862	1.280	0.310	Imputed
rs112944567	1	22,310,811	A	G	0.011	0.014	0.227	0.828	0.157	0.608	1.126	0.540	Imputed
rs1803531	1	22,310,824	T	C	0.015	0.012	0.074	1.287	0.142	0.974	1.700	0.607	Imputed
rs189853639	1	22,310,847	C	G	0.030	0.029	0.548	1.061	0.099	0.874	1.288	0.279	Imputed
rs12130164	1	22,310,933	C	G	0.051	0.050	0.741	1.026	0.077	0.882	1.193	0.044	Imputed
rs12122445	1	22,311,011	G	A	0.050	0.050	0.901	1.010	0.077	0.868	1.175	0.061	Imputed
rs12122466	1	22,311,059	G	C	0.284	0.265	8.08E-03	1.105	0.038	1.026	1.189	0.470	Imputed
rs112413154	1	22,311,115	A	C	0.011	0.014	0.250	0.835	0.157	0.613	1.137	0.520	Imputed
rs111796525	1	22,311,240	C	G	0.012	0.015	0.189	0.819	0.152	0.608	1.104	0.524	Imputed
rs61777960	1	22,311,348	G	A	0.507	0.480	1.61E-03	1.113	0.034	1.041	1.190	0.784	Imputed
rs10799724	1	22,311,413	C	A	0.050	0.049	0.870	1.013	0.077	0.870	1.179	0.058	Imputed
rs112325137	1	22,311,418	T	C	0.030	0.029	0.548	1.061	0.099	0.874	1.288	0.279	Imputed
rs148889413	1	22,311,440	C	A	0.032	0.033	0.945	0.993	0.096	0.823	1.199	0.703	Imputed
rs113692408	1	22,311,556	C	T	0.011	0.014	0.227	0.828	0.157	0.608	1.126	0.540	Imputed
rs12024927	1	22,311,623	T	A	0.379	0.361	0.016	1.088	0.035	1.016	1.165	0.356	Imputed
rs112686052	1	22,311,642	A	G	0.012	0.014	0.282	0.846	0.156	0.624	1.148	0.493	Imputed
rs144616172	1	22,311,656	T	G	0.063	0.064	0.903	0.992	0.070	0.865	1.136	0.686	Imputed
rs12025676	1	22,311,767	T	C	0.053	0.052	0.891	1.010	0.075	0.871	1.171	0.055	Imputed
rs7520526	1	22,311,908	A	G	0.337	0.317	5.32E-03	1.105	0.036	1.030	1.186	0.963	Imputed
rs71638813	1	22,311,970	C	T	0.390	0.370	7.27E-03	1.098	0.035	1.025	1.175	0.472	Imputed
rs7520656	1	22,312,039	G	A	0.056	0.055	0.699	1.029	0.073	0.891	1.188	0.133	Imputed
rs186362232	1	22,312,106	T	C	0.030	0.029	0.578	1.056	0.099	0.870	1.283	0.266	Imputed
rs7512878	1	22,312,111	T	G	0.033	0.033	0.985	1.002	0.094	0.833	1.205	0.020	Imputed
rs191847293	1	22,312,112	C	T	0.011	0.014	0.250	0.835	0.157	0.613	1.137	0.520	Imputed
rs183713485	1	22,312,131	A	T	0.042	0.042	0.876	0.987	0.084	0.836	1.165	0.202	Imputed
rs35194649	1	22,312,187	G	A	0.243	0.223	4.11E-03	1.120	0.040	1.037	1.211	0.266	Imputed
rs7512976	1	22,312,201	T	G	0.094	0.091	0.570	1.034	0.058	0.922	1.159	0.017	Imputed
rs34346609	1	22,312,264	A	G	0.240	0.221	5.98E-03	1.116	0.040	1.032	1.206	0.286	Imputed
rs7520954	1	22,312,287	G	A	0.059	0.059	0.847	1.014	0.071	0.881	1.166	0.137	Imputed
rs112193378	1	22,312,307	C	G	0.393	0.371	4.91E-03	1.102	0.035	1.030	1.180	0.628	Imputed
rs7544164	1	22,312,325	C	A	0.074	0.069	0.342	1.063	0.065	0.936	1.208	0.179	Imputed

rs191505409	1	22,312,341	A	G	0.030	0.029	0.548	1.061	0.099	0.874	1.288	0.279	Imputed
rs7521037	1	22,312,360	G	C	0.054	0.052	0.566	1.044	0.075	0.902	1.208	0.089	Imputed
rs111766277	1	22,312,370	C	T	0.387	0.366	6.03E-03	1.100	0.035	1.028	1.178	0.340	Imputed
rs61777961	1	22,312,419	T	G	0.065	0.061	0.362	1.065	0.069	0.930	1.218	0.075	Imputed
rs7411116	1	22,312,455	T	C	0.099	0.095	0.324	1.058	0.057	0.946	1.182	0.216	Imputed
chr1:22312489:D	1	22,312,489	C	CT	0.031	0.029	0.441	1.078	0.098	0.890	1.307	0.426	Imputed
rs193003375	1	22,312,502	T	C	0.012	0.013	0.644	0.931	0.156	0.685	1.264	0.182	Imputed
rs35389875	1	22,312,617	T	G	0.387	0.366	5.63E-03	1.101	0.035	1.028	1.179	0.325	Imputed
rs185470168	1	22,312,634	A	G	0.011	0.010	0.792	1.044	0.164	0.757	1.440	0.094	Imputed
rs10917098	1	22,312,667	A	G	0.053	0.050	0.487	1.054	0.076	0.909	1.222	0.021	Imputed
rs12121127	1	22,312,714	T	C	0.055	0.053	0.583	1.041	0.074	0.901	1.203	0.009	Imputed
rs12121128	1	22,312,718	T	C	0.055	0.053	0.539	1.046	0.074	0.905	1.209	0.010	Imputed
rs10917099	1	22,312,755	A	G	0.082	0.077	0.333	1.062	0.062	0.940	1.199	0.009	Imputed
rs146511980	1	22,312,793	T	C	0.030	0.029	0.496	1.069	0.099	0.881	1.297	0.262	Imputed
rs12121096	1	22,312,823	A	T	0.384	0.364	8.45E-03	1.096	0.035	1.024	1.173	0.341	Imputed
rs77736376	1	22,312,850	C	G	0.031	0.029	0.530	1.063	0.098	0.878	1.287	0.411	Imputed
rs115366432	1	22,312,998	T	C	0.030	0.029	0.548	1.061	0.099	0.874	1.288	0.279	Imputed
rs61777963	1	22,313,017	T	G	0.162	0.164	0.876	0.993	0.046	0.907	1.087	0.269	Imputed
rs114895362	1	22,313,103	G	C	0.011	0.014	0.250	0.835	0.157	0.613	1.137	0.520	Imputed
chr1:22313197	1	22,313,197	C	A	0.291	0.271	6.71E-03	1.107	0.037	1.028	1.191	0.530	Imputed
rs34262568	1	22,313,371	A	G	0.254	0.235	6.42E-03	1.112	0.039	1.030	1.201	0.449	Imputed
rs3806296	1	22,313,469	C	G	0.333	0.313	5.46E-03	1.105	0.036	1.030	1.186	0.935	Imputed
rs6426736	1	22,313,689	A	T	0.051	0.050	0.677	1.032	0.077	0.888	1.200	0.036	Imputed
rs111384408	1	22,313,730	G	A	0.030	0.029	0.555	1.060	0.099	0.873	1.287	0.281	Imputed
rs112407443	1	22,313,735	T	C	0.028	0.028	0.836	1.021	0.102	0.836	1.248	0.462	Imputed
rs7516572	1	22,313,920	A	G	0.041	0.040	0.756	1.027	0.085	0.868	1.214	0.021	Imputed
chr1:22314015:D	1	22,314,015	G	GA	0.254	0.234	4.68E-03	1.117	0.039	1.034	1.206	0.352	Imputed
rs187149470	1	22,314,034	T	C	0.025	0.028	0.259	0.886	0.108	0.716	1.094	0.134	Imputed
rs141453473	1	22,314,078	A	T	0.038	0.039	0.895	0.989	0.088	0.832	1.175	0.304	Imputed
rs61777965	1	22,314,261	C	G	0.080	0.074	0.078	1.117	0.063	0.988	1.264	0.928	Imputed
rs115300812	1	22,314,406	A	T	0.038	0.037	0.633	1.043	0.088	0.877	1.239	0.029	Imputed
rs61200828	1	22,314,444	A	C	0.113	0.109	0.446	1.042	0.054	0.938	1.157	0.024	Imputed
rs77885439	1	22,314,470	A	G	0.261	0.245	0.028	1.090	0.039	1.009	1.176	0.417	Imputed
rs61777966	1	22,314,566	G	A	0.374	0.351	3.12E-03	1.109	0.035	1.035	1.188	0.400	Imputed
rs147748078	1	22,314,588	G	C	0.031	0.029	0.516	1.065	0.098	0.880	1.290	0.249	Imputed
rs12133550	1	22,314,650	A	C	0.029	0.027	0.558	1.061	0.101	0.870	1.293	0.208	Imputed
rs149399639	1	22,314,850	T	C	0.031	0.029	0.546	1.061	0.098	0.875	1.286	0.344	Imputed
rs139785469	1	22,315,238	G	C	0.010	0.011	0.451	0.879	0.172	0.627	1.231	0.819	Imputed
rs78652364	1	22,315,250	A	G	0.020	0.014	3.73E-03	1.439	0.126	1.124	1.842	0.567	Imputed
rs111414053	1	22,315,318	C	G	0.016	0.015	0.658	1.062	0.135	0.815	1.384	0.550	Imputed

chr1:22315397:D	1	22,315,397	C	CT	0.230	0.215	0.032	1.091	0.041	1.007	1.181	0.462	Imputed
chr1:22315421:D	1	22,315,421	T	TATAC	0.360	0.341	0.011	1.093	0.035	1.020	1.172	0.496	Imputed
chr1:22315462:I	1	22,315,462	GTA	G	0.030	0.029	0.577	1.056	0.099	0.871	1.281	0.246	Imputed
rs139168829	1	22,315,462	A	G	0.052	0.051	0.798	1.020	0.076	0.878	1.184	0.354	Imputed
rs187581857	1	22,315,574	T	C	0.030	0.029	0.525	1.065	0.099	0.878	1.291	0.272	Imputed
rs10917117	1	22,316,408	T	C	0.055	0.053	0.669	1.032	0.075	0.892	1.195	0.464	Imputed
rs141740713	1	22,317,979	G	C	0.052	0.052	0.843	1.015	0.076	0.875	1.178	0.369	Imputed
rs61778002	1	22,318,263	A	G	0.360	0.336	1.81E-03	1.116	0.035	1.042	1.197	0.533	Imputed
rs12132088	1	22,318,407	G	C	0.347	0.325	4.16E-03	1.107	0.036	1.033	1.187	0.586	Imputed
chr1:22318473:D	1	22,318,473	T	TGTG	0.237	0.223	0.051	1.081	0.040	1.000	1.169	0.539	Imputed
rs10917104	1	22,318,517	G	A	0.361	0.338	2.69E-03	1.112	0.035	1.037	1.191	0.519	Imputed
rs148996046	1	22,318,597	A	G	0.052	0.052	0.830	1.016	0.076	0.876	1.180	0.365	Imputed
rs12068538	1	22,318,609	A	G	0.086	0.084	0.521	1.039	0.060	0.924	1.170	0.165	Imputed
rs115082613	1	22,318,840	T	C	0.052	0.052	0.836	1.016	0.076	0.875	1.179	0.367	Imputed
rs146143806	1	22,319,471	G	A	0.012	0.014	0.405	0.881	0.152	0.655	1.187	0.816	Imputed
rs4292925	1	22,320,593	T	C	0.086	0.083	0.454	1.046	0.060	0.930	1.177	0.030	Imputed
rs7519537	1	22,320,611	C	A	0.269	0.245	1.11E-03	1.133	0.038	1.051	1.222	0.969	Imputed
rs182952929	1	22,320,718	G	A	0.051	0.052	0.902	0.991	0.077	0.853	1.151	0.223	Imputed
rs4394607	1	22,320,787	A	T	0.330	0.308	3.25E-03	1.112	0.036	1.036	1.194	0.639	Imputed
rs12404110	1	22,320,801	G	T	0.284	0.264	5.28E-03	1.111	0.038	1.032	1.196	0.493	Imputed
rs10917105	1	22,321,305	A	C	0.353	0.331	2.78E-03	1.112	0.035	1.037	1.192	0.719	Imputed
rs12405048	1	22,321,550	A	G	0.257	0.237	4.88E-03	1.116	0.039	1.034	1.204	0.306	Genotyped
rs12566806	1	22,321,929	T	C	0.296	0.275	3.75E-03	1.114	0.037	1.035	1.198	0.969	Imputed
rs12069407	1	22,321,955	C	A	0.056	0.055	0.770	1.022	0.074	0.884	1.180	0.261	Imputed
rs7528083	1	22,322,274	T	C	0.012	0.010	0.312	1.174	0.159	0.860	1.603	0.588	Imputed
rs10799728	1	22,322,512	G	A	0.349	0.327	3.49E-03	1.109	0.036	1.035	1.190	0.639	Imputed
rs10799729	1	22,323,053	C	G	0.353	0.331	3.10E-03	1.111	0.035	1.036	1.191	0.669	Imputed
rs10917106	1	22,323,189	C	T	0.353	0.331	3.14E-03	1.110	0.035	1.036	1.190	0.670	Imputed
rs4521970	1	22,323,602	C	T	0.349	0.328	6.22E-03	1.102	0.036	1.028	1.182	0.551	Imputed
rs139028896	1	22,324,154	G	C	0.051	0.052	0.975	0.998	0.077	0.858	1.159	0.337	Imputed
rs12031887	1	22,324,246	T	A	0.352	0.330	3.78E-03	1.108	0.036	1.034	1.188	0.715	Imputed
rs12042161	1	22,324,262	T	C	0.352	0.330	4.03E-03	1.107	0.036	1.033	1.187	0.738	Imputed
rs7534936	1	22,324,705	C	T	0.351	0.329	3.10E-03	1.111	0.036	1.036	1.191	0.777	Imputed
rs12032777	1	22,324,930	T	A	0.257	0.237	6.11E-03	1.113	0.039	1.031	1.201	0.308	Imputed
rs7523386	1	22,324,977	T	C	0.055	0.055	0.757	1.023	0.074	0.885	1.182	0.321	Imputed
rs7537414	1	22,325,016	C	T	0.353	0.331	3.66E-03	1.109	0.035	1.034	1.188	0.698	Imputed
rs4655011	1	22,325,061	G	A	0.358	0.335	2.72E-03	1.112	0.035	1.037	1.192	0.515	Imputed
rs12139337	1	22,325,281	T	C	0.296	0.275	4.19E-03	1.112	0.037	1.034	1.196	0.954	Imputed
rs140479399	1	22,325,300	G	C	0.010	0.012	0.295	0.838	0.169	0.601	1.167	0.818	Imputed
rs34193248	1	22,325,358	T	C	0.256	0.237	6.28E-03	1.112	0.039	1.031	1.200	0.306	Imputed

rs57080435	1	22,325,564	G	A	0.071	0.068	0.447	1.052	0.066	0.924	1.198	0.487	Imputed
chr1:22325825:I	1	22,325,825	TC	T	0.055	0.054	0.605	1.039	0.074	0.899	1.202	0.388	Imputed
rs71638817	1	22,325,897	T	C	0.241	0.218	1.55E-03	1.135	0.040	1.049	1.227	0.559	Imputed
rs143111861	1	22,325,898	A	G	0.047	0.047	0.696	1.032	0.080	0.882	1.206	0.884	Imputed
rs12076248	1	22,326,109	C	T	0.055	0.054	0.585	1.041	0.074	0.901	1.203	0.360	Imputed
rs115145848	1	22,326,324	A	G	0.040	0.042	0.686	0.966	0.086	0.817	1.143	0.609	Imputed
rs144319499	1	22,326,327	T	C	0.037	0.040	0.468	0.938	0.089	0.788	1.116	0.474	Imputed
rs118152700	1	22,326,328	G	A	0.037	0.040	0.468	0.938	0.089	0.788	1.116	0.474	Imputed
rs114091988	1	22,326,401	A	C	0.221	0.209	0.080	1.075	0.041	0.991	1.165	0.473	Imputed
rs116422437	1	22,326,416	A	G	0.144	0.139	0.318	1.050	0.049	0.955	1.154	0.810	Imputed
rs116052319	1	22,326,422	T	C	0.218	0.212	0.317	1.042	0.041	0.961	1.130	0.153	Imputed
rs148749262	1	22,326,455	C	T	0.213	0.201	0.066	1.079	0.042	0.995	1.171	0.437	Imputed
rs116204932	1	22,326,488	C	T	0.315	0.301	0.049	1.075	0.037	1.000	1.154	0.475	Imputed
rs144748160	1	22,326,534	T	C	0.031	0.029	0.462	1.075	0.098	0.887	1.303	0.251	Imputed
rs28675219	1	22,326,587	A	G	0.235	0.217	7.15E-03	1.114	0.040	1.030	1.205	0.509	Imputed
rs140866220	1	22,326,778	T	C	0.080	0.079	0.640	1.029	0.062	0.911	1.163	0.118	Imputed
chr1:22326902:I	1	22,326,902	CA	C	0.010	0.010	0.668	0.929	0.172	0.663	1.302	0.972	Imputed
rs146452480	1	22,327,220	A	C	0.051	0.052	0.983	0.998	0.077	0.859	1.160	0.355	Imputed
rs115305682	1	22,327,305	T	C	0.051	0.052	0.879	0.988	0.077	0.850	1.149	0.262	Imputed
rs116773295	1	22,327,398	C	T	0.051	0.052	0.991	1.001	0.077	0.862	1.163	0.347	Imputed
rs183431262	1	22,327,870	T	C	0.019	0.018	0.769	1.037	0.124	0.813	1.322	0.036	Imputed
rs60202359	1	22,327,912	T	C	0.052	0.052	0.916	1.008	0.076	0.868	1.171	0.343	Imputed
rs60703480	1	22,327,961	G	C	0.077	0.078	0.858	0.989	0.063	0.873	1.120	0.498	Imputed
rs141986775	1	22,327,963	G	A	0.025	0.026	0.660	0.954	0.108	0.772	1.178	0.719	Imputed
rs61685243	1	22,327,990	A	C	0.052	0.052	0.924	1.007	0.076	0.867	1.170	0.326	Imputed
rs60569739	1	22,328,059	C	G	0.033	0.033	0.889	1.013	0.095	0.841	1.220	0.222	Imputed
rs58302058	1	22,328,061	T	G	0.032	0.031	0.750	1.031	0.096	0.854	1.246	0.408	Imputed
rs59115023	1	22,328,228	G	C	0.053	0.052	0.736	1.026	0.076	0.885	1.190	0.397	Imputed
rs12751986	1	22,328,279	T	C	0.257	0.237	4.85E-03	1.116	0.039	1.034	1.204	0.277	Imputed
rs143221848	1	22,328,917	T	C	0.010	0.010	0.636	1.080	0.165	0.782	1.493	0.002	Imputed
rs55638883	1	22,329,220	A	G	0.186	0.171	0.017	1.110	0.044	1.019	1.210	0.103	Imputed
rs10917107	1	22,329,414	G	A	0.310	0.288	2.72E-03	1.116	0.037	1.039	1.200	0.580	Imputed
rs7531336	1	22,329,523	G	A	0.017	0.014	0.193	1.189	0.134	0.915	1.546	0.695	Imputed
rs7533776	1	22,329,525	C	T	0.017	0.014	0.151	1.211	0.134	0.932	1.575	0.645	Imputed
rs7519660	1	22,329,543	A	C	0.019	0.018	0.647	1.059	0.126	0.828	1.355	0.943	Imputed
rs41307874	1	22,329,603	T	C	0.025	0.022	0.242	1.134	0.108	0.918	1.402	0.082	Imputed
rs4543768	1	22,329,645	C	T	0.300	0.280	7.22E-03	1.105	0.037	1.027	1.188	0.699	Imputed
rs41300124	1	22,329,681	C	T	0.037	0.036	0.609	1.047	0.089	0.879	1.247	0.280	Imputed
rs4381156	1	22,329,687	T	C	0.021	0.019	0.448	1.093	0.118	0.868	1.377	0.791	Imputed
rs41302021	1	22,329,732	A	G	0.299	0.278	4.48E-03	1.111	0.037	1.033	1.195	0.954	Imputed

rs4543769	1	22,329,826	A	T	0.022	0.019	0.386	1.107	0.117	0.880	1.393	0.749	Imputed
rs57573643	1	22,329,878	A	G	0.269	0.246	1.81E-03	1.127	0.038	1.045	1.215	0.530	Imputed
rs4992899	1	22,329,951	G	A	0.082	0.081	0.592	1.033	0.062	0.916	1.166	0.066	Imputed
rs147109197	1	22,329,990	A	C	0.031	0.029	0.371	1.091	0.098	0.901	1.321	0.221	Imputed
rs12073339	1	22,330,123	G	A	0.295	0.272	1.57E-03	1.125	0.037	1.046	1.210	0.923	Imputed
rs143313123	1	22,330,197	G	T	0.031	0.029	0.371	1.091	0.098	0.901	1.321	0.221	Imputed
rs12088555	1	22,330,205	G	C	0.290	0.266	1.19E-03	1.129	0.037	1.049	1.215	0.618	Imputed
rs34010029	1	22,330,397	A	G	0.024	0.023	0.716	1.041	0.110	0.839	1.292	0.690	Imputed
rs35811644	1	22,330,411	G	A	0.114	0.110	0.319	1.054	0.053	0.950	1.170	0.027	Imputed
rs4556328	1	22,330,539	C	G	0.021	0.020	0.481	1.086	0.118	0.863	1.368	0.605	Imputed
rs4446937	1	22,330,740	T	C	0.343	0.325	0.020	1.087	0.036	1.013	1.166	0.722	Imputed
rs28501339	1	22,330,827	A	C	0.310	0.290	7.07E-03	1.104	0.037	1.027	1.186	0.748	Imputed
rs4317779	1	22,331,571	G	C	0.059	0.059	0.836	0.985	0.072	0.855	1.135	0.810	Imputed
rs4454516	1	22,331,574	C	A	0.055	0.055	0.892	0.990	0.075	0.855	1.146	0.780	Imputed
rs4993601	1	22,331,622	G	T	0.446	0.426	0.014	1.087	0.034	1.017	1.163	0.460	Imputed
chr1:22332114:D	1	22,332,114	T	TG	0.022	0.023	0.764	0.966	0.116	0.769	1.213	0.245	Imputed
rs34380605	1	22,332,676	C	A	0.288	0.269	0.014	1.097	0.038	1.019	1.182	0.615	Imputed
rs7531773	1	22,332,757	C	A	0.379	0.356	3.06E-03	1.109	0.035	1.036	1.188	0.615	Imputed
rs111353443	1	22,332,956	T	G	0.031	0.029	0.342	1.096	0.097	0.906	1.327	0.193	Imputed
rs12760192	1	22,333,078	G	A	0.267	0.245	2.27E-03	1.124	0.038	1.043	1.212	0.744	Imputed
rs61778018	1	22,333,144	A	G	0.367	0.343	1.85E-03	1.116	0.035	1.041	1.195	0.293	Imputed
rs192194964	1	22,333,271	C	T	0.012	0.014	0.238	0.835	0.154	0.617	1.129	0.405	Imputed
rs11583776	1	22,333,538	G	A	0.023	0.024	0.701	0.957	0.113	0.767	1.195	0.130	Imputed
rs3820290	1	22,333,595	G	A	0.368	0.346	3.43E-03	1.108	0.035	1.034	1.187	0.251	Genotyped
rs114302506	1	22,333,620	C	T	0.020	0.017	0.173	1.182	0.123	0.929	1.504	0.716	Imputed
rs3820289	1	22,333,688	C	T	0.235	0.216	5.40E-03	1.118	0.040	1.034	1.210	0.362	Imputed
rs35519693	1	22,334,117	G	C	0.247	0.228	6.76E-03	1.113	0.039	1.030	1.202	0.363	Imputed
rs140356857	1	22,334,151	A	G	0.054	0.053	0.816	1.018	0.075	0.879	1.179	0.371	Imputed
rs12746090	1	22,334,343	T	C	0.235	0.216	5.10E-03	1.119	0.040	1.034	1.210	0.344	Imputed
rs11589764	1	22,334,381	C	A	0.146	0.137	0.119	1.078	0.048	0.981	1.185	0.068	Imputed
rs6696789	1	22,334,415	T	C	0.015	0.016	0.745	0.957	0.137	0.732	1.250	0.150	Imputed
rs6681913	1	22,334,519	G	C	0.018	0.018	0.913	1.014	0.127	0.790	1.301	0.724	Imputed
rs55854648	1	22,334,704	T	C	0.367	0.343	1.93E-03	1.115	0.035	1.041	1.195	0.296	Imputed
rs12746780	1	22,334,716	T	C	0.267	0.245	1.85E-03	1.127	0.038	1.045	1.215	0.736	Imputed
rs116768932	1	22,334,734	G	C	0.054	0.053	0.823	1.017	0.075	0.878	1.178	0.373	Imputed
rs12750883	1	22,334,851	T	C	0.235	0.216	4.96E-03	1.119	0.040	1.035	1.211	0.347	Imputed
chr1:22334957:I	1	22,334,957	GA	G	0.228	0.209	4.88E-03	1.121	0.041	1.035	1.214	0.337	Imputed
rs111650992	1	22,335,023	T	C	0.031	0.029	0.342	1.096	0.097	0.906	1.327	0.193	Imputed
rs187718899	1	22,335,373	T	C	0.017	0.020	0.168	0.836	0.131	0.647	1.080	0.375	Imputed
rs10753525	1	22,335,404	A	T	0.200	0.184	0.018	1.106	0.043	1.017	1.203	0.501	Imputed

rs10917108	1	22,335,584	C	T	0.268	0.246	1.66E-03	1.128	0.038	1.046	1.216	0.741	Imputed
rs182465934	1	22,335,633	A	C	0.014	0.015	0.678	0.942	0.145	0.709	1.251	0.592	Imputed
rs34552571	1	22,335,655	A	G	0.236	0.216	3.67E-03	1.123	0.040	1.039	1.215	0.369	Imputed
rs146131710	1	22,335,723	G	A	0.031	0.029	0.342	1.096	0.097	0.906	1.327	0.193	Imputed
rs34840017	1	22,335,731	T	C	0.236	0.217	3.99E-03	1.122	0.040	1.037	1.214	0.361	Imputed
rs35025763	1	22,335,964	C	G	0.233	0.214	6.77E-03	1.115	0.040	1.031	1.207	0.439	Imputed
rs4344303	1	22,336,120	C	T	0.236	0.217	4.35E-03	1.121	0.040	1.036	1.212	0.377	Imputed
chr1:22336179:D	1	22,336,179	T	TC	0.031	0.029	0.342	1.096	0.097	0.906	1.327	0.193	Imputed
chr1:22336180:D	1	22,336,180	C	CCT	0.009	0.012	0.140	0.768	0.179	0.541	1.092	0.789	Imputed
rs3820285	1	22,336,277	C	G	0.015	0.015	0.897	0.982	0.140	0.746	1.293	0.070	Imputed
rs9187	1	22,336,305	T	C	0.269	0.246	1.49E-03	1.129	0.038	1.048	1.217	0.785	Imputed
rs12908	1	22,336,308	A	G	0.269	0.246	1.49E-03	1.129	0.038	1.048	1.217	0.785	Imputed
rs3806297	1	22,336,371	C	A	0.299	0.275	8.23E-04	1.132	0.037	1.053	1.217	0.987	Imputed
rs60189068	1	22,336,643	C	G	0.087	0.084	0.494	1.042	0.060	0.926	1.172	0.203	Imputed
chr1:22336812:I	1	22,336,812	AC	A	0.014	0.016	0.631	0.935	0.141	0.709	1.232	0.929	Imputed
rs191816083	1	22,336,841	A	C	0.021	0.021	0.972	0.996	0.120	0.788	1.259	0.020	Imputed
rs184799444	1	22,336,842	G	A	0.021	0.021	0.972	0.996	0.120	0.788	1.259	0.020	Imputed
rs10917109	1	22,337,181	T	C	0.285	0.260	7.13E-04	1.136	0.038	1.055	1.223	0.554	Imputed
rs74464236	1	22,337,182	G	C	0.302	0.274	1.32E-04	1.152	0.037	1.071	1.239	0.551	Imputed
rs10917111	1	22,337,271	C	A	0.280	0.256	8.21E-04	1.135	0.038	1.054	1.223	0.700	Imputed
rs116482722	1	22,338,481	C	G	0.064	0.073	0.071	0.883	0.069	0.772	1.011	0.262	Imputed
rs10917116	1	22,339,279	C	A	0.023	0.022	0.797	1.030	0.113	0.825	1.286	0.420	Imputed
rs56046273	1	22,339,495	C	G	0.020	0.022	0.429	0.911	0.118	0.722	1.149	0.014	Imputed
rs2092321	1	22,341,319	G	A	0.030	0.029	0.521	1.065	0.099	0.878	1.292	0.198	Imputed
rs2501309	1	22,341,714	T	C	0.373	0.351	5.06E-03	1.103	0.035	1.030	1.182	0.102	Imputed
rs10917118	1	22,341,755	C	T	0.387	0.365	4.93E-03	1.103	0.035	1.030	1.180	0.074	Imputed
rs150478288	1	22,342,214	T	A	0.012	0.014	0.344	0.865	0.154	0.639	1.170	0.340	Imputed
rs148504001	1	22,342,596	C	T	0.020	0.016	0.072	1.250	0.124	0.981	1.594	0.221	Imputed
rs2501306	1	22,342,837	T	G	0.056	0.053	0.491	1.052	0.074	0.910	1.215	0.011	Imputed
rs2501305	1	22,342,876	A	G	0.345	0.325	8.83E-03	1.098	0.036	1.024	1.177	0.277	Imputed
rs2473309	1	22,342,883	C	T	0.350	0.331	0.015	1.091	0.036	1.017	1.169	0.346	Imputed
rs57860778	1	22,342,928	T	C	0.021	0.019	0.562	1.072	0.120	0.848	1.355	0.741	Imputed
rs6694116	1	22,343,070	T	C	0.021	0.019	0.571	1.070	0.120	0.846	1.353	0.747	Imputed
rs12022453	1	22,343,298	T	C	0.055	0.054	0.942	1.005	0.074	0.869	1.163	0.942	Imputed
rs2501304	1	22,343,468	C	G	0.341	0.321	7.52E-03	1.100	0.036	1.026	1.180	0.295	Imputed
rs2501303	1	22,343,924	C	G	0.346	0.325	5.21E-03	1.105	0.036	1.030	1.185	0.289	Imputed
rs2501302	1	22,343,971	T	C	0.346	0.325	5.21E-03	1.105	0.036	1.030	1.185	0.289	Imputed
rs2501301	1	22,344,027	T	C	0.034	0.029	0.094	1.169	0.094	0.973	1.405	0.108	Imputed
rs2501300	1	22,344,031	G	A	0.346	0.325	5.21E-03	1.105	0.036	1.030	1.185	0.289	Imputed
chr1:22344498:D	1	22,344,498	T	TG	0.017	0.017	0.736	0.956	0.132	0.739	1.238	0.271	Imputed

chr1:22344681:I	1	22,344,681	A	AT	0.343	0.320	2.05E-03	1.116	0.036	1.041	1.197	0.312	Imputed
rs75526061	1	22,344,725	A	G	0.315	0.288	3.02E-04	1.142	0.037	1.062	1.227	0.961	Imputed
rs185486524	1	22,344,736	C	T	0.036	0.036	0.732	0.969	0.092	0.810	1.160	0.722	Imputed
rs4654778	1	22,345,006	T	C	0.333	0.313	6.87E-03	1.102	0.036	1.027	1.183	0.638	Imputed
rs3117048	1	22,345,093	A	G	0.352	0.332	6.75E-03	1.101	0.035	1.027	1.180	0.590	Genotyped
rs113556747	1	22,345,375	A	G	0.048	0.054	0.101	0.880	0.079	0.754	1.026	0.204	Imputed
rs2501299	1	22,345,647	C	T	0.360	0.337	1.91E-03	1.116	0.035	1.041	1.196	0.634	Imputed
rs2501298	1	22,345,860	A	G	0.030	0.030	0.862	0.983	0.099	0.809	1.194	0.188	Imputed
rs2501296	1	22,346,009	A	G	0.010	0.011	0.668	0.930	0.170	0.666	1.297	0.896	Imputed
rs2501295	1	22,346,158	G	A	0.032	0.029	0.349	1.095	0.097	0.905	1.324	0.301	Imputed
rs75806297	1	22,346,184	C	T	0.023	0.024	0.840	0.978	0.113	0.784	1.219	0.199	Imputed
rs187114668	1	22,346,339	G	C	0.017	0.014	0.246	1.169	0.134	0.899	1.521	0.323	Imputed
rs2473308	1	22,346,416	A	G	0.038	0.033	0.159	1.134	0.089	0.952	1.351	0.221	Imputed
rs139286514	1	22,346,653	A	G	0.042	0.046	0.232	0.905	0.084	0.768	1.066	0.829	Imputed
rs12097709	1	22,347,138	T	C	0.244	0.243	0.922	1.004	0.039	0.929	1.085	0.144	Imputed
rs12097775	1	22,347,252	T	C	0.241	0.242	0.979	0.999	0.040	0.924	1.080	0.101	Imputed
rs12407439	1	22,347,396	G	A	0.191	0.158	4.86E-08	1.267	0.043	1.164	1.380	0.828	Imputed
chr1:22347656:I	1	22,347,656	AG	A	0.185	0.155	4.83E-07	1.247	0.044	1.144	1.360	0.882	Imputed
rs2865198	1	22,348,104	T	C	0.253	0.250	0.714	1.014	0.039	0.940	1.095	0.117	Imputed
rs2501293	1	22,348,110	C	T	0.126	0.122	0.550	1.031	0.051	0.933	1.139	0.028	Imputed
rs10917119	1	22,348,409	A	G	0.184	0.155	9.71E-07	1.240	0.044	1.138	1.352	0.957	Imputed
rs11586488	1	22,348,556	C	T	0.253	0.250	0.678	1.016	0.039	0.942	1.097	0.104	Imputed
rs3117047	1	22,348,587	C	A	0.518	0.482	1.81E-05	1.156	0.034	1.082	1.236	0.534	Imputed
rs11588236	1	22,349,012	G	T	0.089	0.082	0.149	1.090	0.060	0.970	1.224	0.304	Imputed
rs11588264	1	22,349,150	G	T	0.364	0.353	0.239	1.043	0.035	0.973	1.118	0.704	Imputed
rs79050195	1	22,349,386	T	C	0.189	0.160	1.77E-06	1.231	0.044	1.131	1.341	0.846	Imputed
rs10799730	1	22,349,615	G	A	0.517	0.483	4.90E-05	1.147	0.034	1.074	1.226	0.624	Imputed
rs10917120	1	22,350,165	A	G	0.251	0.249	0.713	1.014	0.039	0.940	1.095	0.149	Imputed
rs2501292	1	22,350,541	C	T	0.076	0.073	0.606	1.034	0.064	0.912	1.172	0.246	Imputed
rs12061255	1	22,350,547	T	C	0.251	0.249	0.717	1.014	0.039	0.940	1.095	0.148	Imputed
rs2473307	1	22,350,834	T	C	0.517	0.483	4.90E-05	1.147	0.034	1.074	1.226	0.624	Imputed
rs2473306	1	22,350,933	T	C	0.074	0.071	0.627	1.032	0.065	0.909	1.171	0.210	Imputed
rs3754505	1	22,351,326	C	G	0.251	0.249	0.734	1.013	0.039	0.939	1.094	0.144	Imputed
rs61778042	1	22,351,947	T	G	0.187	0.184	0.575	1.025	0.044	0.941	1.116	0.367	Imputed
rs2473305	1	22,351,976	C	T	0.074	0.071	0.627	1.032	0.065	0.909	1.171	0.209	Imputed
rs2255282	1	22,352,040	G	T	0.519	0.485	5.70E-05	1.146	0.034	1.072	1.225	0.718	Imputed
rs11801382	1	22,352,108	T	C	0.253	0.251	0.783	1.011	0.039	0.937	1.091	0.141	Imputed
rs1883422	1	22,352,179	A	C	0.023	0.020	0.366	1.109	0.115	0.886	1.389	0.961	Imputed
chr1:22352395:D	1	22,352,395	T	GCTCAGCTCCCCCCCACCA	0.217	0.219	0.801	0.990	0.041	0.913	1.073	0.193	Imputed
rs79643821	1	22,352,561	G	A	0.013	0.019	8.14E-03	0.682	0.145	0.513	0.906	0.071	Imputed

rs114095512	1	22,352,716	T	C	0.009	0.012	0.230	0.814	0.172	0.580	1.141	0.387	Imputed
rs3768579	1	22,353,136	A	G	0.190	0.161	1.87E-06	1.230	0.044	1.130	1.340	0.759	Imputed
rs12044816	1	22,353,298	G	A	0.055	0.054	0.888	1.010	0.074	0.874	1.169	0.103	Imputed
rs2473304	1	22,353,327	C	A	0.248	0.249	0.845	0.992	0.039	0.919	1.072	0.950	Imputed
chr1:22353345:I	1	22,353,345	AC	A	0.165	0.170	0.474	0.968	0.046	0.885	1.058	0.190	Imputed
rs2501291	1	22,353,414	G	A	0.247	0.248	0.827	0.992	0.039	0.918	1.071	0.917	Imputed
rs2473303	1	22,353,491	C	T	0.494	0.465	4.51E-04	1.126	0.034	1.054	1.203	0.280	Imputed
rs2473302	1	22,353,824	C	T	0.246	0.248	0.777	0.989	0.039	0.916	1.068	0.947	Imputed
chr1:22353835:D	1	22,353,835	TTCTA	T	0.249	0.250	0.825	0.991	0.039	0.918	1.070	0.961	Imputed
rs2473301	1	22,353,874	G	A	0.246	0.248	0.777	0.989	0.039	0.916	1.068	0.947	Imputed
rs2501290	1	22,353,986	C	G	0.327	0.322	0.528	1.023	0.036	0.953	1.098	0.521	Imputed
rs2501289	1	22,353,989	C	G	0.327	0.322	0.528	1.023	0.036	0.953	1.098	0.521	Imputed
rs41307810	1	22,354,045	T	C	0.046	0.038	5.29E-03	1.257	0.082	1.070	1.476	0.155	Imputed
rs3768574	1	22,354,110	C	T	0.055	0.055	0.876	1.012	0.074	0.875	1.169	0.095	Genotyped
rs116674939	1	22,354,237	C	T	0.013	0.019	7.58E-03	0.679	0.145	0.511	0.903	0.078	Imputed
rs2473300	1	22,354,246	A	T	0.246	0.248	0.777	0.989	0.039	0.916	1.068	0.947	Imputed
rs2473299	1	22,354,274	G	C	0.073	0.070	0.596	1.035	0.065	0.911	1.176	0.215	Imputed
rs76114061	1	22,354,277	C	T	0.254	0.255	0.789	0.990	0.039	0.917	1.068	0.918	Imputed
rs3768571	1	22,354,327	C	T	0.172	0.177	0.447	0.967	0.045	0.885	1.055	0.329	Imputed
rs2473297	1	22,354,537	A	G	0.246	0.248	0.780	0.989	0.039	0.916	1.068	0.949	Imputed
rs80173514	1	22,354,538	A	C	0.190	0.161	1.55E-06	1.232	0.043	1.131	1.342	0.807	Imputed
rs2473296	1	22,354,587	C	T	0.255	0.255	0.950	0.998	0.039	0.925	1.076	0.854	Imputed
chr1:22354659:I	1	22,354,659	C	CA	0.499	0.472	8.33E-04	1.120	0.034	1.048	1.197	0.321	Imputed
rs2473295	1	22,354,866	T	C	0.247	0.249	0.767	0.988	0.039	0.915	1.067	0.785	Genotyped
rs4654780	1	22,355,044	A	G	0.175	0.178	0.667	0.981	0.045	0.899	1.071	0.975	Imputed
rs7523572	1	22,355,289	C	G	0.246	0.247	0.832	0.992	0.039	0.918	1.071	0.954	Imputed
rs10917121	1	22,355,458	A	G	0.078	0.075	0.605	1.033	0.063	0.913	1.169	0.193	Imputed
rs12116952	1	22,355,578	G	A	0.263	0.268	0.480	0.973	0.038	0.903	1.049	0.711	Imputed
rs61778044	1	22,355,631	T	C	0.171	0.176	0.471	0.968	0.045	0.887	1.057	0.291	Imputed
rs10917122	1	22,355,724	C	A	0.266	0.265	0.988	0.999	0.038	0.927	1.077	0.596	Imputed
rs150153349	1	22,355,890	G	A	0.029	0.032	0.227	0.885	0.101	0.727	1.078	0.064	Imputed
rs10917123	1	22,355,978	A	G	0.244	0.216	2.33E-05	1.182	0.040	1.094	1.277	0.248	Imputed
rs2473294	1	22,356,098	T	C	0.247	0.248	0.868	0.994	0.039	0.920	1.073	0.923	Imputed
rs2473293	1	22,356,138	T	C	0.246	0.248	0.798	0.990	0.039	0.917	1.069	0.943	Imputed
rs2473292	1	22,356,155	G	A	0.246	0.248	0.798	0.990	0.039	0.917	1.069	0.943	Imputed
rs1883421	1	22,356,640	C	G	0.246	0.248	0.808	0.991	0.039	0.917	1.070	0.932	Imputed
rs1063116	1	22,356,809	A	T	0.246	0.247	0.821	0.991	0.039	0.918	1.070	0.915	Imputed
rs1063117	1	22,356,888	G	A	0.246	0.247	0.852	0.993	0.039	0.919	1.072	0.932	Imputed
rs1063118	1	22,356,905	G	A	0.246	0.247	0.824	0.991	0.039	0.918	1.071	0.917	Imputed
rs760923	1	22,357,217	G	T	0.246	0.247	0.834	0.992	0.039	0.918	1.071	0.922	Imputed

rs3820687	1	22,357,229	T	A	0.244	0.216	2.68E-05	1.181	0.040	1.092	1.276	0.226	Imputed
rs35536968	1	22,357,488	A	C	0.040	0.038	0.743	1.029	0.087	0.868	1.219	0.909	Imputed
rs2473291	1	22,357,549	G	T	0.253	0.253	0.923	0.996	0.039	0.923	1.075	0.762	Imputed
rs760922	1	22,358,055	T	C	0.073	0.070	0.566	1.038	0.065	0.914	1.179	0.177	Imputed
rs28431812	1	22,358,401	T	C	0.054	0.054	0.992	0.999	0.074	0.864	1.156	0.090	Imputed
rs2473290	1	22,358,457	T	C	0.264	0.232	7.93E-06	1.188	0.039	1.101	1.281	0.300	Imputed
rs150073000	1	22,358,560	T	C	0.052	0.052	0.941	0.994	0.076	0.857	1.154	0.060	Imputed
rs2473289	1	22,358,573	T	C	0.441	0.411	2.53E-04	1.133	0.034	1.060	1.211	0.853	Imputed
rs61778045	1	22,358,595	A	G	0.190	0.162	2.11E-06	1.229	0.043	1.128	1.338	0.833	Imputed
rs4492583	1	22,358,663	A	G	0.054	0.054	0.985	0.999	0.074	0.863	1.156	0.091	Imputed
chr1:22358743:D	1	22,358,743	A	AT	0.054	0.054	0.993	0.999	0.074	0.864	1.156	0.090	Imputed
rs2473288	1	22,358,747	T	C	0.429	0.399	1.85E-04	1.136	0.034	1.063	1.215	0.858	Imputed
rs61778046	1	22,358,789	T	G	0.190	0.161	2.27E-06	1.228	0.044	1.128	1.337	0.838	Imputed
rs6679085	1	22,358,832	T	C	0.086	0.080	0.319	1.062	0.061	0.943	1.196	0.231	Imputed
rs12027753	1	22,358,836	T	C	0.193	0.165	3.81E-06	1.221	0.043	1.122	1.329	0.693	Imputed
rs11589377	1	22,358,906	T	C	0.445	0.414	1.70E-04	1.137	0.034	1.063	1.215	0.898	Imputed
rs7541107	1	22,359,082	G	C	0.430	0.399	1.81E-04	1.137	0.034	1.063	1.215	0.828	Imputed
chr1:22359133:D	1	22,359,133	ACAGT	A	0.429	0.399	1.77E-04	1.137	0.034	1.063	1.216	0.853	Imputed
rs2009020	1	22,359,392	A	G	0.443	0.409	4.50E-05	1.149	0.034	1.075	1.229	0.698	Imputed
rs12757748	1	22,359,557	T	C	0.040	0.038	0.788	1.024	0.087	0.863	1.214	0.810	Imputed
rs2473287	1	22,359,698	T	C	0.430	0.399	1.57E-04	1.138	0.034	1.064	1.217	0.841	Imputed
chr1:22359735:D	1	22,359,735	C	CA	0.024	0.024	0.863	0.981	0.111	0.790	1.219	0.335	Imputed
rs72665313	1	22,359,839	C	T	0.190	0.162	2.06E-06	1.229	0.043	1.129	1.338	0.831	Imputed
rs2473286	1	22,359,922	T	C	0.443	0.412	1.42E-04	1.138	0.034	1.065	1.217	0.726	Imputed
rs10917124	1	22,359,983	T	C	0.053	0.053	0.989	0.999	0.075	0.862	1.158	0.131	Imputed
rs2501287	1	22,359,994	A	G	0.445	0.413	1.34E-04	1.139	0.034	1.065	1.218	0.877	Imputed
rs10917125	1	22,360,049	T	C	0.442	0.409	6.27E-05	1.146	0.034	1.072	1.225	0.784	Imputed
rs56408422	1	22,360,138	T	C	0.166	0.167	0.942	0.997	0.046	0.912	1.090	0.362	Imputed
rs12021606	1	22,360,260	G	T	0.430	0.399	1.60E-04	1.138	0.034	1.064	1.217	0.826	Imputed
rs12021637	1	22,360,383	G	A	0.443	0.410	5.91E-05	1.147	0.034	1.073	1.226	0.774	Imputed
rs67111920	1	22,360,461	G	A	0.431	0.399	9.23E-05	1.143	0.034	1.069	1.222	0.870	Imputed
rs2501286	1	22,360,581	T	G	0.443	0.410	6.78E-05	1.145	0.034	1.071	1.225	0.772	Imputed
rs2473285	1	22,360,582	T	C	0.443	0.411	7.11E-05	1.145	0.034	1.071	1.224	0.776	Imputed
rs2473284	1	22,360,689	T	C	0.443	0.410	6.70E-05	1.146	0.034	1.071	1.225	0.771	Imputed
rs34879787	1	22,360,777	G	A	0.053	0.053	0.977	1.002	0.075	0.865	1.161	0.058	Imputed
rs2473283	1	22,360,800	G	A	0.430	0.400	1.72E-04	1.137	0.034	1.063	1.216	0.870	Imputed
rs2473282	1	22,360,855	C	T	0.432	0.402	2.56E-04	1.133	0.034	1.060	1.212	0.757	Imputed
rs7525440	1	22,360,869	C	T	0.443	0.412	1.30E-04	1.139	0.034	1.066	1.218	0.678	Imputed
rs7525444	1	22,360,876	C	T	0.432	0.402	2.25E-04	1.134	0.034	1.061	1.213	0.744	Imputed
rs4655015	1	22,360,911	A	G	0.430	0.399	1.44E-04	1.139	0.034	1.065	1.218	0.815	Imputed

rs4655016	1	22,360,970	C	T	0.430	0.399	1.44E-04	1.139	0.034	1.065	1.218	0.815	Imputed
rs4654781	1	22,361,062	T	C	0.437	0.407	3.04E-04	1.131	0.034	1.058	1.210	0.810	Imputed
rs2473281	1	22,361,129	C	T	0.430	0.399	1.44E-04	1.139	0.034	1.065	1.218	0.815	Imputed
rs111992780	1	22,361,229	T	C	0.192	0.163	2.19E-06	1.227	0.043	1.127	1.336	0.766	Imputed
rs140820597	1	22,361,313	C	G	0.182	0.199	9.35E-03	0.892	0.044	0.819	0.972	0.372	Imputed
rs2473280	1	22,361,356	G	C	0.441	0.407	5.04E-05	1.148	0.034	1.074	1.228	0.663	Imputed
rs2473279	1	22,361,472	C	T	0.430	0.399	1.29E-04	1.140	0.034	1.066	1.219	0.804	Imputed
rs2473278	1	22,361,504	G	A	0.430	0.399	1.49E-04	1.139	0.034	1.065	1.218	0.833	Imputed
rs2473277	1	22,361,845	G	A	0.511	0.473	6.00E-06	1.165	0.034	1.091	1.245	0.691	Genotyped
rs2501285	1	22,362,044	A	G	0.036	0.033	0.341	1.090	0.091	0.912	1.302	0.044	Imputed
rs2473276	1	22,362,194	T	C	0.442	0.407	2.45E-05	1.155	0.034	1.080	1.235	0.924	Imputed
rs2501284	1	22,362,197	A	G	0.450	0.414	1.26E-05	1.160	0.034	1.085	1.240	0.969	Imputed
rs3117046	1	22,362,426	C	T	0.430	0.399	1.29E-04	1.140	0.034	1.066	1.219	0.804	Imputed
rs2473275	1	22,362,717	C	T	0.437	0.405	7.22E-05	1.145	0.034	1.071	1.224	0.650	Imputed
rs144426966	1	22,362,769	T	C	0.040	0.041	0.928	0.992	0.086	0.838	1.175	0.277	Imputed
rs2038109	1	22,362,805	T	G	0.454	0.419	2.60E-05	1.154	0.034	1.079	1.233	0.677	Imputed
rs2038108	1	22,362,813	A	G	0.053	0.053	0.824	0.983	0.076	0.848	1.140	0.077	Imputed
rs2038107	1	22,362,869	A	G	0.053	0.054	0.900	0.991	0.075	0.855	1.148	0.081	Imputed
rs2038106	1	22,362,940	G	T	0.436	0.407	4.65E-04	1.127	0.034	1.054	1.205	0.784	Imputed
rs2038105	1	22,363,039	A	G	0.431	0.400	1.29E-04	1.140	0.034	1.066	1.219	0.804	Imputed
rs3117045	1	22,363,507	A	G	0.430	0.399	1.29E-04	1.140	0.034	1.066	1.219	0.804	Imputed
rs3123459	1	22,363,646	A	C	0.432	0.401	1.77E-04	1.137	0.034	1.063	1.216	0.917	Imputed
rs3117044	1	22,363,918	G	A	0.430	0.399	1.29E-04	1.140	0.034	1.066	1.219	0.804	Imputed
rs34231820	1	22,364,193	T	C	0.054	0.055	0.988	0.999	0.074	0.863	1.155	0.084	Imputed
rs79223209	1	22,364,283	A	G	0.054	0.055	0.988	0.999	0.074	0.863	1.155	0.084	Imputed
rs11582320	1	22,364,527	T	C	0.030	0.031	0.882	0.986	0.098	0.814	1.194	0.119	Imputed
rs77121533	1	22,364,827	T	G	0.187	0.190	0.624	0.979	0.043	0.899	1.066	0.798	Imputed
chr1:22364950:I	1	22,364,950	CTTTA	C	0.054	0.054	0.910	0.992	0.075	0.856	1.148	0.079	Imputed
rs10917126	1	22,364,954	A	C	0.054	0.055	0.986	0.999	0.074	0.863	1.155	0.090	Imputed
rs2501281	1	22,364,958	A	C	0.335	0.302	2.41E-05	1.164	0.036	1.085	1.249	0.157	Imputed
rs10917127	1	22,365,085	T	C	0.053	0.053	0.961	0.996	0.075	0.860	1.155	0.068	Imputed
rs12405695	1	22,365,689	T	C	0.193	0.164	1.17E-06	1.233	0.043	1.133	1.343	0.802	Imputed
rs2038103	1	22,365,792	A	G	0.055	0.055	0.999	1.000	0.074	0.864	1.157	0.088	Imputed
rs10799731	1	22,365,829	C	T	0.194	0.164	8.27E-07	1.237	0.043	1.136	1.346	0.799	Imputed
rs182244345	1	22,365,831	T	C	0.019	0.023	0.103	0.818	0.123	0.643	1.042	0.758	Imputed
rs2038102	1	22,365,937	T	C	0.081	0.078	0.569	1.036	0.062	0.917	1.170	0.081	Imputed
rs10917128	1	22,366,102	C	T	0.192	0.162	9.88E-07	1.236	0.043	1.135	1.346	0.974	Imputed
rs116825850	1	22,366,426	T	C	0.023	0.024	0.445	0.917	0.113	0.735	1.145	0.280	Imputed
rs74821829	1	22,366,675	A	T	0.026	0.026	0.919	1.011	0.107	0.819	1.248	0.064	Imputed
rs72665317	1	22,367,073	G	T	0.194	0.164	9.45E-07	1.235	0.043	1.135	1.344	0.850	Imputed

rs2473273	1	22,367,222	T	C	0.073	0.070	0.595	1.035	0.065	0.911	1.176	0.196	Imputed
rs2501280	1	22,367,292	C	G	0.073	0.070	0.595	1.035	0.065	0.911	1.176	0.196	Imputed
rs11590939	1	22,367,572	T	C	0.073	0.070	0.609	1.034	0.065	0.910	1.174	0.189	Imputed
rs11590519	1	22,367,622	A	G	0.075	0.072	0.722	1.023	0.064	0.902	1.161	0.193	Imputed
rs10917129	1	22,367,836	T	C	0.084	0.080	0.555	1.037	0.061	0.920	1.169	0.196	Imputed
rs2501279	1	22,368,342	C	T	0.434	0.402	5.79E-05	1.147	0.034	1.073	1.227	0.757	Imputed
rs2473332	1	22,369,117	C	T	0.433	0.400	7.14E-05	1.145	0.034	1.071	1.225	0.770	Imputed
rs12748456	1	22,370,157	A	G	0.165	0.167	0.837	0.991	0.046	0.906	1.083	0.274	Imputed
chr1:22370234:D	1	22,370,234	T	TTTAA	0.190	0.161	9.59E-07	1.237	0.044	1.136	1.347	0.766	Imputed
chr1:22370240:D	1	22,370,240	T	TAA	0.183	0.154	7.16E-07	1.244	0.044	1.141	1.357	0.930	Imputed
rs2473331	1	22,370,330	A	C	0.081	0.078	0.774	1.018	0.062	0.901	1.150	0.230	Imputed
rs2473330	1	22,370,421	A	G	0.434	0.401	5.62E-05	1.148	0.034	1.073	1.227	0.782	Imputed
chr1:22370457:D	1	22,370,457	C	CT	0.192	0.165	6.03E-06	1.216	0.043	1.117	1.324	0.820	Imputed
rs10917130	1	22,371,065	G	T	0.191	0.162	1.00E-06	1.236	0.043	1.135	1.346	0.734	Imputed
rs12024016	1	22,371,380	G	A	0.053	0.053	0.954	0.996	0.075	0.859	1.154	0.063	Imputed
rs2501278	1	22,371,711	G	T	0.126	0.123	0.692	1.020	0.051	0.923	1.127	0.023	Imputed
rs2473329	1	22,372,681	A	C	0.033	0.032	0.909	1.011	0.095	0.840	1.217	0.539	Imputed
rs2501277	1	22,372,683	A	C	0.277	0.267	0.178	1.052	0.038	0.977	1.134	0.700	Imputed
rs143252229	1	22,372,789	A	G	0.015	0.015	0.951	1.009	0.141	0.765	1.329	0.799	Imputed
rs2473328	1	22,373,406	A	G	0.070	0.068	0.726	1.024	0.066	0.899	1.166	0.319	Imputed
rs2501276	1	22,373,624	T	C	0.080	0.096	9.81E-04	0.816	0.062	0.723	0.921	0.898	Genotyped
rs6661161	1	22,373,671	C	T	0.161	0.165	0.518	0.971	0.046	0.887	1.062	0.583	Imputed
rs71638823	1	22,374,032	T	C	0.145	0.143	0.695	1.019	0.048	0.927	1.120	0.736	Imputed
rs72665319	1	22,374,400	A	G	0.071	0.074	0.348	0.940	0.066	0.826	1.070	0.182	Imputed
rs78715691	1	22,374,646	C	T	0.015	0.020	0.019	0.724	0.138	0.553	0.949	0.198	Imputed
rs11579182	1	22,374,693	G	A	0.145	0.143	0.671	1.021	0.048	0.929	1.121	0.749	Imputed
rs2501275	1	22,375,065	C	T	0.235	0.236	0.888	0.994	0.040	0.920	1.075	0.940	Genotyped
rs11580039	1	22,375,190	C	T	0.145	0.143	0.680	1.020	0.048	0.928	1.121	0.744	Imputed
rs12127684	1	22,375,896	A	G	0.038	0.039	0.744	0.971	0.089	0.816	1.156	0.467	Imputed
rs10917131	1	22,376,188	A	G	0.161	0.164	0.674	0.981	0.046	0.896	1.073	0.481	Imputed
rs35139465	1	22,376,452	T	C	0.162	0.164	0.736	0.985	0.046	0.900	1.077	0.456	Imputed
rs113706674	1	22,376,490	A	G	0.183	0.187	0.455	0.968	0.044	0.888	1.054	0.771	Imputed
rs2473327	1	22,376,947	A	G	0.073	0.071	0.765	1.020	0.065	0.898	1.158	0.183	Imputed
rs74941957	1	22,377,142	T	C	0.054	0.054	0.980	1.002	0.075	0.865	1.160	0.068	Imputed
rs111343946	1	22,377,338	A	G	0.053	0.053	0.911	0.992	0.075	0.856	1.149	0.058	Imputed
rs7554578	1	22,377,349	A	G	0.081	0.081	0.720	0.978	0.062	0.866	1.104	0.248	Imputed
rs3754501	1	22,377,543	A	T	0.145	0.143	0.671	1.021	0.048	0.929	1.121	0.749	Imputed
rs3754500	1	22,377,718	A	G	0.145	0.143	0.680	1.020	0.048	0.928	1.121	0.744	Imputed
rs725158	1	22,378,280	A	T	0.202	0.168	5.89E-08	1.259	0.043	1.158	1.369	0.708	Imputed
rs16826209	1	22,378,469	A	G	0.035	0.036	0.930	0.992	0.092	0.829	1.187	0.433	Imputed

rs3754497	1	22,378,670	A	G	0.146	0.144	0.633	1.023	0.048	0.931	1.124	0.810	Imputed
rs3754496	1	22,378,880	A	C	0.202	0.168	5.89E-08	1.259	0.043	1.158	1.369	0.708	Imputed
rs2473325	1	22,379,088	G	A	0.313	0.312	0.947	1.002	0.036	0.933	1.077	0.509	Imputed
rs16860621	1	22,379,360	A	G	0.155	0.153	0.698	1.018	0.047	0.929	1.116	0.993	Imputed
rs12133104	1	22,379,528	C	G	0.072	0.070	0.805	1.016	0.066	0.894	1.156	0.187	Imputed
rs16860629	1	22,379,648	T	C	0.072	0.071	0.736	1.022	0.065	0.900	1.162	0.067	Imputed
rs13375412	1	22,379,669	C	G	0.089	0.089	0.868	0.990	0.059	0.882	1.112	0.183	Imputed
rs13374393	1	22,379,671	T	G	0.079	0.078	0.916	1.007	0.063	0.890	1.139	0.273	Imputed
rs182451497	1	22,379,863	G	C	0.022	0.026	0.151	0.850	0.114	0.680	1.062	0.131	Imputed
rs2501274	1	22,380,012	T	C	0.079	0.075	0.462	1.047	0.063	0.926	1.185	0.310	Imputed
rs1980475	1	22,380,237	T	C	0.073	0.071	0.775	1.019	0.065	0.897	1.157	0.186	Imputed
rs17837945	1	22,380,309	T	C	0.053	0.053	0.909	0.991	0.075	0.855	1.149	0.058	Imputed
rs114307407	1	22,380,772	A	G	0.032	0.031	0.655	1.044	0.096	0.865	1.259	0.665	Imputed
rs56382348	1	22,380,905	T	C	0.018	0.023	0.035	0.766	0.126	0.598	0.982	0.141	Imputed
rs11802628	1	22,381,386	G	A	0.016	0.017	0.684	0.947	0.134	0.729	1.231	0.585	Imputed
chr1:22381397:D	1	22,381,397	T	TATATATATATATATATA	0.130	0.113	8.52E-04	1.184	0.051	1.072	1.308	0.304	Imputed
chr1:22381399:D	1	22,381,399	T	TATATATATATATATA	0.237	0.202	1.39E-07	1.235	0.040	1.142	1.337	0.722	Imputed
rs2501272	1	22,381,501	C	T	0.169	0.164	0.547	1.028	0.046	0.940	1.124	0.254	Imputed
rs2501271	1	22,381,561	C	T	0.084	0.082	0.876	1.010	0.061	0.896	1.138	0.250	Imputed
rs12729825	1	22,382,739	G	A	0.145	0.142	0.634	1.023	0.048	0.931	1.125	0.675	Imputed
rs16826243	1	22,382,942	A	G	0.017	0.023	0.018	0.738	0.129	0.573	0.950	0.175	Imputed
rs2143104	1	22,383,222	C	T	0.073	0.071	0.852	1.012	0.065	0.891	1.150	0.172	Imputed
rs16826249	1	22,383,521	T	C	0.071	0.075	0.372	0.943	0.066	0.829	1.073	0.187	Imputed
chr1:22384077:D	1	22,384,077	A	ATG	0.144	0.142	0.651	1.022	0.048	0.930	1.123	0.675	Imputed
rs10917135	1	22,384,097	C	G	0.077	0.076	0.934	1.005	0.063	0.888	1.138	0.254	Imputed
rs2501270	1	22,384,279	T	C	0.081	0.083	0.539	0.963	0.062	0.853	1.087	0.168	Imputed
rs145801681	1	22,384,358	T	A	0.013	0.013	0.817	0.966	0.151	0.719	1.297	0.058	Imputed
rs148963741	1	22,384,471	T	C	0.019	0.022	0.229	0.863	0.123	0.679	1.098	0.423	Imputed
rs12035094	1	22,384,713	T	G	0.034	0.027	9.46E-03	1.277	0.095	1.061	1.537	0.460	Imputed
chr1:22385504:D	1	22,385,504	GA	G	0.090	0.085	0.456	1.045	0.059	0.930	1.174	0.078	Imputed
chr1:22386063:D	1	22,386,063	AT	A	0.079	0.078	0.981	0.999	0.063	0.883	1.129	0.114	Imputed
rs41266025	1	22,386,150	T	C	0.014	0.020	0.013	0.704	0.141	0.534	0.929	0.125	Imputed
chr1:22386391:D	1	22,386,391	TC	T	0.073	0.071	0.857	1.012	0.065	0.891	1.149	0.173	Imputed
rs16826278	1	22,386,722	A	T	0.160	0.163	0.659	0.980	0.046	0.895	1.073	0.429	Imputed
rs16826279	1	22,386,859	G	A	0.145	0.142	0.671	1.021	0.048	0.929	1.122	0.673	Imputed
rs909821	1	22,386,871	A	T	0.073	0.071	0.864	1.011	0.065	0.890	1.149	0.174	Imputed
rs16826283	1	22,386,930	C	G	0.015	0.020	0.017	0.721	0.138	0.550	0.944	0.192	Imputed
rs12029550	1	22,387,377	T	A	0.053	0.053	0.884	0.989	0.075	0.853	1.147	0.060	Imputed
rs16826293	1	22,387,525	G	A	0.186	0.188	0.658	0.981	0.044	0.901	1.068	0.875	Imputed
rs16826296	1	22,387,622	A	G	0.014	0.020	0.013	0.703	0.141	0.533	0.928	0.124	Imputed

rs12129231	1	22,387,996	A	G	0.037	0.039	0.453	0.935	0.090	0.784	1.114	0.579	Imputed
chr1:22388017:D	1	22,388,017	GTTTC	G	0.237	0.237	0.925	0.996	0.040	0.922	1.077	0.898	Imputed
rs16826302	1	22,388,297	A	G	0.191	0.204	0.039	0.915	0.043	0.842	0.996	0.399	Imputed
rs17837951	1	22,388,872	T	C	0.203	0.169	2.49E-08	1.267	0.042	1.165	1.377	0.803	Imputed
rs2473324	1	22,388,891	G	C	0.073	0.071	0.864	1.011	0.065	0.890	1.149	0.174	Imputed
rs2473323	1	22,389,327	G	T	0.073	0.071	0.891	1.009	0.065	0.888	1.146	0.181	Genotyped
rs16826309	1	22,389,388	G	A	0.144	0.142	0.669	1.021	0.048	0.929	1.122	0.656	Imputed
rs78986575	1	22,389,407	T	G	0.008	0.011	0.196	0.789	0.184	0.550	1.132	0.383	Imputed
rs2473322	1	22,390,072	G	T	0.073	0.071	0.851	1.012	0.065	0.891	1.150	0.172	Genotyped
rs11583790	1	22,390,078	A	G	0.144	0.142	0.669	1.021	0.048	0.929	1.122	0.656	Imputed
chr1:22390559:D	1	22,390,559	T	TA	0.022	0.027	0.070	0.812	0.115	0.649	1.017	0.026	Imputed
rs6691650	1	22,390,667	G	A	0.073	0.071	0.858	1.012	0.065	0.891	1.149	0.173	Imputed
chr1:22390814:D	1	22,390,814	TCG	T	0.094	0.093	0.907	1.007	0.058	0.899	1.128	0.046	Imputed
chr1:22390816:D	1	22,390,816	GCT	G	0.074	0.072	0.887	1.009	0.065	0.889	1.146	0.177	Imputed
rs2473320	1	22,391,262	G	C	0.073	0.071	0.858	1.012	0.065	0.891	1.149	0.173	Imputed
rs17837957	1	22,391,389	T	C	0.035	0.036	0.772	0.974	0.092	0.813	1.166	0.598	Imputed
rs17837958	1	22,391,399	T	C	0.021	0.022	0.868	0.981	0.117	0.779	1.234	0.303	Imputed
chr1:22391477:D	1	22,391,477	T	TTGCC	0.013	0.013	0.817	0.966	0.151	0.719	1.297	0.058	Imputed
rs12406669	1	22,391,563	C	G	0.144	0.142	0.669	1.021	0.048	0.929	1.122	0.656	Imputed
rs2501269	1	22,391,600	A	G	0.073	0.071	0.858	1.012	0.065	0.891	1.149	0.173	Imputed
rs16826354	1	22,391,767	A	G	0.032	0.031	0.693	1.039	0.096	0.861	1.254	0.693	Imputed
rs12037582	1	22,391,784	C	G	0.053	0.053	0.884	0.989	0.075	0.853	1.147	0.060	Imputed
rs6689660	1	22,392,262	G	T	0.016	0.016	0.916	0.986	0.135	0.757	1.283	0.813	Imputed
chr1:22393192:I	1	22,393,192	A	AT	0.222	0.219	0.705	1.016	0.041	0.938	1.100	0.843	Imputed
rs2473319	1	22,393,449	A	G	0.075	0.072	0.724	1.023	0.064	0.902	1.161	0.246	Imputed
rs12740791	1	22,393,524	T	C	0.100	0.101	0.797	0.986	0.057	0.882	1.101	0.302	Imputed
rs17425695	1	22,393,980	A	G	0.078	0.076	0.545	1.039	0.063	0.918	1.176	0.073	Imputed
rs2473318	1	22,394,575	A	G	0.073	0.071	0.851	1.012	0.065	0.891	1.150	0.172	Imputed
rs17837965	1	22,394,625	G	A	0.050	0.048	0.517	1.052	0.078	0.903	1.225	0.356	Imputed
rs2473317	1	22,395,251	G	C	0.118	0.136	1.94E-03	0.851	0.052	0.769	0.943	0.904	Imputed
rs3123460	1	22,395,636	C	A	0.073	0.071	0.858	1.012	0.065	0.891	1.149	0.173	Imputed
chr1:22396104:D	1	22,396,104	C	CA	0.340	0.377	3.35E-06	0.846	0.036	0.788	0.908	0.828	Imputed
chr1:22396288:D	1	22,396,288	T	TTTG	0.203	0.168	2.30E-08	1.268	0.043	1.166	1.378	0.811	Imputed
rs75223442	1	22,396,359	G	T	0.053	0.053	0.833	0.984	0.075	0.849	1.141	0.055	Imputed
rs56319427	1	22,396,998	C	T	0.210	0.178	2.41E-07	1.241	0.042	1.144	1.348	0.660	Imputed
rs2501256	1	22,397,199	C	T	0.356	0.322	1.97E-05	1.163	0.035	1.085	1.247	0.365	Imputed
rs11807562	1	22,397,248	T	C	0.144	0.142	0.666	1.021	0.048	0.929	1.122	0.657	Imputed
rs190076702	1	22,397,661	T	C	0.011	0.013	0.415	0.876	0.163	0.637	1.205	0.539	Imputed
rs34896897	1	22,398,905	G	A	0.144	0.142	0.666	1.021	0.048	0.929	1.122	0.657	Imputed
rs16826413	1	22,400,378	G	A	0.013	0.014	0.573	0.920	0.149	0.688	1.231	0.328	Imputed

rs10799732	1	22,400,771	A	G	0.053	0.053	0.884	0.989	0.075	0.853	1.147	0.060	Imputed
rs11582456	1	22,400,992	G	A	0.026	0.025	0.763	1.033	0.107	0.838	1.273	0.302	Imputed
rs16826425	1	22,401,024	A	G	0.145	0.142	0.645	1.022	0.048	0.930	1.124	0.650	Imputed
rs10917139	1	22,401,538	A	G	0.144	0.142	0.646	1.022	0.048	0.930	1.124	0.631	Imputed
rs115044388	1	22,401,784	T	A	0.014	0.020	0.013	0.703	0.141	0.533	0.928	0.124	Imputed
rs2038101	1	22,402,113	A	G	0.073	0.071	0.853	1.012	0.065	0.891	1.150	0.172	Imputed
rs16826450	1	22,403,017	G	T	0.015	0.021	0.021	0.732	0.136	0.561	0.954	0.216	Genotyped
rs16826452	1	22,403,107	C	T	0.037	0.035	0.576	1.052	0.090	0.881	1.255	0.662	Imputed
rs12038474	1	22,403,357	A	G	0.205	0.170	7.32E-09	1.277	0.042	1.175	1.387	0.740	Imputed
rs16826458	1	22,403,714	A	G	0.015	0.020	0.017	0.720	0.138	0.549	0.943	0.190	Imputed
rs1016494	1	22,404,139	C	G	0.073	0.071	0.853	1.012	0.065	0.891	1.150	0.172	Imputed
rs1016493	1	22,404,178	A	T	0.073	0.071	0.853	1.012	0.065	0.891	1.150	0.172	Imputed
rs75284716	1	22,405,202	A	T	0.010	0.011	0.428	0.874	0.170	0.626	1.220	0.481	Imputed
rs2072921	1	22,405,398	A	G	0.145	0.142	0.645	1.022	0.048	0.930	1.124	0.650	Imputed
rs2501311	1	22,405,590	G	A	0.073	0.071	0.853	1.012	0.065	0.891	1.150	0.172	Imputed
rs78091109	1	22,405,667	A	G	0.015	0.020	0.017	0.721	0.138	0.550	0.944	0.192	Imputed
chr1:22407102:D	1	22,407,102	C	CT	0.203	0.168	2.30E-08	1.268	0.043	1.166	1.378	0.811	Imputed
rs2501282	1	22,408,106	G	A	0.073	0.071	0.853	1.012	0.065	0.891	1.150	0.172	Imputed
rs2056976	1	22,408,465	C	T	0.073	0.071	0.853	1.012	0.065	0.891	1.150	0.172	Imputed
rs2056975	1	22,408,527	A	G	0.073	0.071	0.845	1.013	0.065	0.892	1.150	0.171	Imputed
rs767399	1	22,408,777	A	G	0.073	0.071	0.853	1.012	0.065	0.891	1.150	0.172	Imputed
rs2056974	1	22,409,094	T	A	0.235	0.236	0.831	0.992	0.040	0.917	1.072	0.995	Imputed
rs16826506	1	22,409,290	C	A	0.015	0.020	0.017	0.721	0.138	0.550	0.944	0.192	Imputed
chr1:22409372:D	1	22,409,372	TTA	T	0.081	0.082	0.748	0.980	0.062	0.868	1.107	0.473	Imputed
chr1:22409373:D	1	22,409,373	TA	T	0.073	0.071	0.853	1.012	0.065	0.891	1.150	0.172	Imputed
rs12566130	1	22,410,416	G	T	0.053	0.053	0.825	0.984	0.076	0.848	1.140	0.065	Imputed
rs17425702	1	22,410,998	G	T	0.015	0.020	0.017	0.721	0.138	0.550	0.944	0.192	Imputed
rs2473316	1	22,411,019	T	G	0.247	0.249	0.797	0.990	0.039	0.917	1.069	0.810	Imputed
rs2473315	1	22,411,135	T	C	0.079	0.076	0.647	1.029	0.063	0.910	1.164	0.237	Imputed
rs2501262	1	22,411,159	G	A	0.073	0.071	0.858	1.012	0.065	0.891	1.149	0.173	Imputed
rs2501261	1	22,411,177	A	G	0.236	0.238	0.742	0.987	0.040	0.913	1.067	0.939	Imputed
rs2473314	1	22,411,351	T	G	0.074	0.072	0.743	1.021	0.065	0.900	1.160	0.141	Imputed
chr1:22411715:D	1	22,411,715	ACT	A	0.482	0.452	2.54E-04	1.132	0.034	1.059	1.210	0.873	Imputed
rs16826532	1	22,412,570	T	C	0.015	0.020	0.017	0.721	0.138	0.550	0.944	0.192	Imputed
rs10917145	1	22,412,645	G	A	0.144	0.142	0.626	1.024	0.048	0.932	1.125	0.642	Imputed
rs6696317	1	22,413,874	C	T	0.073	0.071	0.846	1.013	0.065	0.892	1.150	0.161	Imputed
rs11800014	1	22,414,070	T	C	0.160	0.163	0.688	0.982	0.046	0.897	1.075	0.414	Genotyped
rs2268179	1	22,414,785	T	G	0.203	0.168	1.77E-08	1.270	0.042	1.168	1.380	0.808	Imputed
rs2268177	1	22,415,410	T	A	0.203	0.168	1.77E-08	1.270	0.042	1.168	1.380	0.808	Imputed
rs10917148	1	22,415,680	A	G	0.073	0.071	0.862	1.011	0.065	0.890	1.149	0.174	Genotyped

rs763442	1	22,415,941	T	C	0.073	0.071	0.858	1.012	0.065	0.891	1.149	0.173	Imputed
rs74901847	1	22,417,418	A	G	0.019	0.017	0.468	1.096	0.126	0.857	1.402	0.045	Imputed
rs7519109	1	22,417,543	G	A	0.073	0.071	0.856	1.012	0.065	0.891	1.149	0.173	Imputed
chr1:22418260:I	1	22,418,260	CAA	C	0.199	0.165	2.51E-08	1.269	0.043	1.167	1.380	0.785	Imputed
rs1803269	1	22,418,721	A	G	0.010	0.012	0.276	0.831	0.170	0.595	1.161	0.320	Imputed
rs722697	1	22,419,759	A	G	0.073	0.071	0.858	1.012	0.065	0.891	1.149	0.173	Imputed
rs16826571	1	22,420,440	A	C	0.081	0.077	0.327	1.063	0.062	0.941	1.201	0.094	Imputed
rs11577378	1	22,420,449	T	C	0.145	0.142	0.595	1.026	0.048	0.934	1.127	0.678	Imputed
rs116591804	1	22,420,786	T	G	0.074	0.074	0.821	1.015	0.065	0.894	1.152	0.395	Imputed
rs16826582	1	22,420,852	A	G	0.102	0.090	0.012	1.152	0.057	1.031	1.287	0.868	Imputed
rs6702176	1	22,420,932	C	A	0.212	0.213	0.854	0.992	0.042	0.915	1.077	0.299	Imputed
rs12740705	1	22,421,113	T	C	0.145	0.142	0.603	1.025	0.048	0.933	1.127	0.673	Imputed
rs28462103	1	22,421,399	T	C	0.053	0.054	0.867	0.988	0.075	0.852	1.145	0.114	Imputed
rs10799733	1	22,421,831	C	T	0.231	0.230	0.915	1.004	0.040	0.928	1.086	0.388	Imputed
rs112366217	1	22,422,041	T	C	0.015	0.020	0.017	0.720	0.138	0.549	0.943	0.190	Imputed
rs909820	1	22,422,554	A	G	0.074	0.072	0.931	1.006	0.065	0.886	1.142	0.170	Imputed
rs10917151	1	22,422,721	A	G	0.193	0.156	5.63E-10	1.308	0.043	1.201	1.424	0.540	Imputed
chr1:22422766:I	1	22,422,766	TA	T	0.015	0.020	0.020	0.725	0.138	0.554	0.950	0.162	Imputed
chr1:22422795:D	1	22,422,795	GT	G	0.082	0.083	0.785	0.984	0.061	0.872	1.109	0.051	Imputed
rs4076750	1	22,423,543	G	A	0.161	0.165	0.601	0.976	0.046	0.892	1.068	0.462	Genotyped
rs2056973	1	22,423,968	C	T	0.093	0.092	0.992	1.001	0.058	0.893	1.122	0.061	Imputed
rs76739921	1	22,424,075	G	C	0.027	0.023	0.100	1.190	0.106	0.967	1.463	0.673	Imputed
rs80288086	1	22,424,076	G	T	0.027	0.023	0.100	1.190	0.106	0.967	1.463	0.673	Imputed
rs16826588	1	22,424,113	G	C	0.016	0.023	7.72E-03	0.704	0.132	0.543	0.912	0.322	Imputed
rs78923644	1	22,424,264	C	A	0.035	0.042	0.040	0.830	0.091	0.694	0.992	0.379	Imputed
rs6688182	1	22,424,456	C	G	0.117	0.136	1.18E-03	0.844	0.052	0.762	0.935	0.736	Imputed
rs116017034	1	22,425,377	A	G	0.034	0.036	0.510	0.940	0.094	0.783	1.129	0.313	Imputed
rs10917152	1	22,425,642	T	C	0.134	0.134	0.935	1.004	0.050	0.911	1.107	0.657	Imputed
rs12563981	1	22,425,988	T	C	0.053	0.054	0.867	0.988	0.075	0.852	1.145	0.114	Imputed
rs2865179	1	22,426,009	T	C	0.082	0.083	0.737	0.980	0.061	0.869	1.105	0.063	Imputed
rs1534949	1	22,426,187	C	A	0.442	0.408	2.92E-05	1.153	0.034	1.079	1.233	0.976	Genotyped
chr1:22426386:D	1	22,426,386	GCTTGTCCAAGAATTCAGA	A	0.089	0.088	0.912	1.007	0.059	0.896	1.130	0.056	Imputed
chr1:22426504:D	1	22,426,504	TTTC	T	0.149	0.150	0.781	0.987	0.048	0.899	1.083	0.095	Imputed
chr1:22426520:D	1	22,426,520	TTC	T	0.088	0.089	0.631	0.972	0.060	0.865	1.092	0.055	Imputed
chr1:22426521:D	1	22,426,521	TC	T	0.089	0.089	0.708	0.978	0.059	0.871	1.099	0.063	Imputed
rs74059867	1	22,427,894	A	C	0.015	0.020	0.017	0.720	0.138	0.549	0.943	0.190	Imputed
rs55991506	1	22,428,133	G	C	0.012	0.012	0.939	0.988	0.155	0.729	1.339	0.323	Imputed
rs10799735	1	22,428,446	A	C	0.082	0.083	0.752	0.981	0.061	0.870	1.106	0.062	Imputed
rs6672792	1	22,428,701	A	G	0.246	0.250	0.505	0.974	0.039	0.902	1.052	0.644	Imputed
chr1:22428775:D	1	22,428,775	TAC	T	0.442	0.408	3.51E-05	1.152	0.034	1.077	1.231	0.941	Imputed

chr1:22428778:D	1	22,428,778	A	AC	0.411	0.428	0.023	0.924	0.035	0.864	0.989	0.443	Imputed
chr1:22429532:D	1	22,429,532	GGGA	G	0.119	0.120	0.785	0.986	0.052	0.890	1.092	0.314	Imputed
rs2143103	1	22,430,069	A	G	0.137	0.137	0.892	1.007	0.049	0.914	1.109	0.513	Imputed
chr1:22430118:I	1	22,430,118	C	CTT	0.083	0.083	0.754	0.981	0.061	0.870	1.106	0.057	Imputed
rs72665339	1	22,430,920	A	G	0.196	0.199	0.607	0.978	0.043	0.900	1.064	0.971	Imputed
rs66766977	1	22,431,092	T	C	0.494	0.460	4.17E-05	1.149	0.034	1.075	1.228	0.532	Imputed
rs11580451	1	22,431,337	T	C	0.133	0.133	0.987	0.999	0.050	0.906	1.102	0.619	Imputed
rs115384208	1	22,431,471	C	T	0.015	0.015	0.816	1.033	0.139	0.787	1.355	0.522	Imputed
rs72647403	1	22,431,610	T	G	0.037	0.041	0.124	0.871	0.089	0.731	1.038	0.064	Imputed
rs115860811	1	22,432,154	C	G	0.014	0.020	0.013	0.701	0.143	0.530	0.928	0.106	Imputed
rs4655021	1	22,432,282	G	A	0.083	0.083	0.784	0.983	0.061	0.872	1.109	0.055	Imputed
rs4655022	1	22,432,419	T	C	0.232	0.238	0.367	0.965	0.040	0.892	1.043	0.594	Imputed
rs10489437	1	22,432,587	C	T	0.052	0.054	0.724	0.974	0.076	0.840	1.129	0.064	Imputed
rs2865177	1	22,432,771	A	G	0.082	0.083	0.742	0.980	0.061	0.869	1.105	0.058	Imputed
rs34974651	1	22,433,129	C	A	0.099	0.101	0.776	0.984	0.057	0.881	1.099	0.331	Imputed
rs6426737	1	22,434,019	G	A	0.082	0.083	0.742	0.980	0.061	0.869	1.105	0.058	Imputed
rs6426738	1	22,434,241	T	C	0.082	0.083	0.782	0.983	0.061	0.872	1.109	0.059	Imputed
rs6426739	1	22,434,386	T	C	0.082	0.083	0.782	0.983	0.061	0.872	1.109	0.059	Imputed
rs1883423	1	22,434,705	G	A	0.082	0.083	0.742	0.980	0.061	0.869	1.105	0.058	Imputed
rs760924	1	22,435,550	C	T	0.082	0.083	0.747	0.981	0.061	0.869	1.106	0.058	Imputed
rs2092322	1	22,435,723	G	C	0.495	0.461	4.26E-05	1.149	0.034	1.075	1.227	0.573	Imputed
rs7412010	1	22,436,446	C	G	0.191	0.156	2.44E-09	1.296	0.044	1.190	1.411	0.570	Imputed
chr1:22436820:D	1	22,436,820	G	GC	0.057	0.057	0.981	0.998	0.073	0.865	1.152	0.072	Imputed
rs10917154	1	22,437,016	T	C	0.053	0.054	0.815	0.983	0.075	0.848	1.139	0.056	Imputed
rs149446242	1	22,437,591	A	G	0.016	0.014	0.384	1.128	0.138	0.860	1.478	0.789	Imputed
rs143040561	1	22,438,421	T	G	0.009	0.011	0.153	0.778	0.177	0.551	1.100	0.218	Imputed
rs16826640	1	22,438,744	G	A	0.015	0.021	0.014	0.713	0.138	0.544	0.934	0.197	Imputed
chr1:22439394:D	1	22,439,394	GGGA	G	0.082	0.083	0.782	0.983	0.061	0.872	1.109	0.059	Imputed
rs4654783	1	22,439,520	T	C	0.331	0.295	2.01E-06	1.187	0.036	1.106	1.274	0.099	Genotyped
rs115805722	1	22,439,610	T	C	0.009	0.011	0.153	0.778	0.177	0.551	1.100	0.218	Imputed
rs114397905	1	22,439,702	C	T	0.014	0.020	0.013	0.704	0.141	0.534	0.929	0.125	Imputed
rs16826645	1	22,440,339	T	C	0.053	0.054	0.873	0.988	0.075	0.853	1.145	0.052	Imputed
rs7553377	1	22,440,925	T	C	0.101	0.103	0.665	0.976	0.056	0.875	1.089	0.008	Imputed
chr1:22441093:D	1	22,441,093	A	AG	0.043	0.052	0.019	0.823	0.083	0.701	0.968	0.541	Imputed
rs1474647	1	22,441,865	C	T	0.472	0.444	5.36E-04	1.125	0.034	1.052	1.202	0.357	Imputed
chr1:22441879:D	1	22,441,879	A	AC	0.059	0.059	0.913	1.008	0.071	0.876	1.159	0.090	Imputed
rs76439356	1	22,441,982	T	C	0.016	0.012	0.042	1.319	0.137	1.009	1.724	0.343	Imputed
rs1474646	1	22,442,025	A	G	0.224	0.232	0.235	0.953	0.040	0.880	1.032	0.565	Genotyped
rs34745135	1	22,442,171	C	G	0.125	0.127	0.784	0.986	0.051	0.892	1.090	0.647	Imputed
rs74059875	1	22,442,735	G	A	0.015	0.020	0.017	0.721	0.138	0.550	0.944	0.192	Imputed

rs7522784	1	22,442,983	G	T	0.082	0.083	0.782	0.983	0.061	0.872	1.109	0.059	Imputed
rs12131920	1	22,442,987	T	C	0.016	0.022	0.011	0.713	0.133	0.549	0.926	0.245	Imputed
chr1:22443318:I	1	22,443,318	T	TC	0.082	0.083	0.782	0.983	0.061	0.872	1.109	0.059	Imputed
rs72879291	1	22,443,482	T	A	0.035	0.037	0.447	0.932	0.092	0.778	1.117	0.279	Imputed
rs2235530	1	22,443,760	T	C	0.053	0.054	0.873	0.988	0.075	0.853	1.145	0.052	Imputed
rs1046310	1	22,443,887	G	T	0.493	0.460	7.07E-05	1.144	0.034	1.070	1.222	0.405	Genotyped
rs78716180	1	22,444,040	T	A	0.016	0.015	0.630	1.068	0.135	0.819	1.392	0.298	Imputed
rs147420415	1	22,444,486	C	T	0.014	0.013	0.469	1.111	0.145	0.836	1.476	0.381	Imputed
rs4655023	1	22,444,490	A	C	0.082	0.083	0.782	0.983	0.061	0.872	1.109	0.059	Imputed
rs7552004	1	22,444,810	C	A	0.082	0.083	0.782	0.983	0.061	0.872	1.109	0.059	Imputed
rs10753528	1	22,444,859	T	G	0.225	0.233	0.215	0.951	0.040	0.879	1.030	0.628	Imputed
rs10737462	1	22,444,975	T	C	0.248	0.211	4.41E-08	1.241	0.039	1.148	1.340	0.645	Imputed
rs3765351	1	22,445,991	C	T	0.486	0.455	1.26E-04	1.139	0.034	1.065	1.217	0.399	Genotyped
chr1:22446108:I	1	22,446,108	C	CT	0.474	0.438	1.32E-05	1.159	0.034	1.085	1.239	0.197	Imputed
rs34228276	1	22,446,768	A	G	0.015	0.020	0.018	0.723	0.138	0.552	0.947	0.196	Imputed
rs12756110	1	22,447,148	G	C	0.082	0.083	0.740	0.980	0.061	0.869	1.105	0.063	Imputed
rs3765350	1	22,447,316	G	A	0.248	0.211	5.04E-08	1.240	0.039	1.147	1.339	0.663	Imputed
rs114834853	1	22,447,478	T	G	0.014	0.020	0.013	0.704	0.141	0.534	0.929	0.125	Imputed
rs10753529	1	22,447,523	G	A	0.082	0.083	0.751	0.981	0.061	0.870	1.106	0.062	Imputed
chr1:22447646:I	1	22,447,646	T	TGG	0.235	0.242	0.342	0.963	0.040	0.891	1.041	0.586	Imputed
rs12131703	1	22,448,217	T	C	0.117	0.136	1.15E-03	0.844	0.052	0.761	0.935	0.385	Genotyped
rs35923353	1	22,448,448	A	C	0.125	0.127	0.776	0.986	0.051	0.892	1.089	0.643	Imputed
rs74059877	1	22,448,877	A	G	0.015	0.020	0.017	0.721	0.138	0.550	0.944	0.192	Imputed
rs11584458	1	22,448,932	T	C	0.150	0.156	0.357	0.957	0.047	0.873	1.050	0.353	Imputed
rs12037005	1	22,449,239	T	C	0.053	0.054	0.854	0.986	0.075	0.851	1.143	0.068	Imputed
rs59709264	1	22,449,325	G	A	0.491	0.459	9.58E-05	1.141	0.034	1.068	1.219	0.469	Imputed
rs72647409	1	22,449,360	T	C	0.025	0.024	0.614	1.057	0.109	0.853	1.309	0.117	Imputed
rs10737463	1	22,449,452	T	C	0.082	0.083	0.786	0.984	0.061	0.872	1.109	0.058	Imputed
rs10799736	1	22,449,952	T	C	0.082	0.083	0.785	0.984	0.061	0.872	1.109	0.062	Imputed
rs9919225	1	22,450,471	T	C	0.082	0.083	0.740	0.980	0.061	0.869	1.105	0.067	Imputed
rs2235529	1	22,450,487	T	C	0.187	0.151	2.37E-09	1.300	0.044	1.192	1.416	0.609	Genotyped
rs149730061	1	22,451,715	A	G	0.014	0.020	0.013	0.704	0.141	0.534	0.929	0.125	Imputed
rs7526484	1	22,451,845	T	C	0.235	0.240	0.399	0.967	0.040	0.894	1.046	0.811	Genotyped
rs10917155	1	22,451,966	A	G	0.054	0.054	0.892	0.990	0.075	0.855	1.146	0.064	Imputed
rs56172502	1	22,452,263	A	G	0.020	0.015	0.039	1.290	0.124	1.012	1.644	0.223	Imputed
rs11580864	1	22,452,374	C	T	0.161	0.164	0.620	0.977	0.046	0.893	1.070	0.255	Imputed
rs4655024	1	22,452,714	C	T	0.488	0.457	1.37E-04	1.138	0.034	1.065	1.216	0.391	Imputed
rs10917157	1	22,453,324	C	T	0.053	0.054	0.802	0.981	0.075	0.847	1.138	0.073	Imputed
rs41266029	1	22,453,619	T	C	0.034	0.032	0.433	1.076	0.093	0.896	1.291	0.568	Imputed
rs6676241	1	22,453,626	C	T	0.159	0.162	0.664	0.980	0.046	0.895	1.073	0.253	Imputed

rs2235528	1	22,453,640	T	C	0.028	0.028	0.951	0.994	0.102	0.813	1.215	0.026	Imputed
rs6678992	1	22,453,842	C	T	0.159	0.162	0.664	0.980	0.046	0.895	1.073	0.253	Imputed
rs2235527	1	22,453,975	G	C	0.159	0.162	0.664	0.980	0.046	0.895	1.073	0.253	Imputed
rs2235526	1	22,454,001	C	T	0.159	0.162	0.668	0.980	0.046	0.895	1.073	0.254	Genotyped
rs6679479	1	22,454,261	A	T	0.159	0.162	0.664	0.980	0.046	0.895	1.073	0.253	Imputed
rs2235525	1	22,454,325	A	G	0.159	0.162	0.664	0.980	0.046	0.895	1.073	0.253	Imputed
rs10917158	1	22,454,373	G	C	0.159	0.162	0.664	0.980	0.046	0.895	1.073	0.253	Imputed
rs79345128	1	22,454,375	A	G	0.033	0.035	0.437	0.929	0.095	0.771	1.119	0.257	Imputed
rs10917159	1	22,454,791	A	C	0.159	0.162	0.661	0.980	0.046	0.895	1.073	0.252	Imputed
rs7536301	1	22,454,979	T	C	0.082	0.083	0.788	0.984	0.061	0.872	1.109	0.071	Imputed
rs78804914	1	22,455,092	A	C	0.014	0.020	0.020	0.721	0.140	0.548	0.949	0.152	Imputed
rs7544210	1	22,455,142	G	A	0.462	0.430	9.00E-05	1.142	0.034	1.069	1.221	0.541	Genotyped
rs116736185	1	22,455,190	T	C	0.014	0.015	0.792	0.963	0.142	0.730	1.271	0.791	Imputed
rs10753530	1	22,455,314	G	A	0.163	0.164	0.856	0.992	0.046	0.907	1.085	0.282	Imputed
rs111655786	1	22,455,434	T	C	0.013	0.013	0.991	0.998	0.151	0.743	1.341	0.135	Imputed
rs11582100	1	22,455,449	G	A	0.161	0.163	0.675	0.981	0.046	0.896	1.074	0.252	Imputed
rs10737464	1	22,455,499	T	C	0.082	0.083	0.787	0.984	0.061	0.872	1.109	0.071	Imputed
rs11582542	1	22,455,588	C	T	0.160	0.163	0.663	0.980	0.046	0.895	1.073	0.248	Imputed
rs56673898	1	22,455,728	C	T	0.466	0.434	8.91E-05	1.142	0.034	1.069	1.221	0.507	Imputed
rs115547783	1	22,456,050	C	G	0.010	0.012	0.369	0.860	0.169	0.618	1.197	0.308	Imputed
rs877629	1	22,456,895	G	A	0.160	0.163	0.663	0.980	0.046	0.895	1.073	0.248	Imputed
rs909816	1	22,456,970	T	C	0.161	0.163	0.833	0.990	0.046	0.905	1.084	0.243	Imputed
rs877628	1	22,457,027	C	T	0.026	0.028	0.246	0.883	0.107	0.716	1.089	0.110	Imputed
rs34087879	1	22,457,611	A	G	0.159	0.162	0.664	0.980	0.046	0.895	1.073	0.253	Imputed
rs1076680	1	22,458,384	T	C	0.159	0.161	0.681	0.981	0.046	0.896	1.074	0.248	Imputed
chr1:22458637:I	1	22,458,637	TG	T	0.134	0.135	0.942	0.996	0.050	0.904	1.098	0.543	Imputed
rs12404660	1	22,458,794	G	A	0.222	0.189	3.80E-07	1.231	0.041	1.136	1.335	0.693	Imputed
rs742358	1	22,459,170	G	A	0.462	0.430	8.95E-05	1.142	0.034	1.069	1.221	0.576	Imputed
rs141702740	1	22,459,254	A	G	0.010	0.011	0.497	0.888	0.173	0.633	1.247	0.008	Imputed
rs76996541	1	22,459,299	A	G	0.014	0.020	0.017	0.715	0.140	0.543	0.941	0.160	Imputed
rs116422505	1	22,459,578	A	G	0.015	0.017	0.397	0.891	0.137	0.682	1.165	0.044	Imputed
rs4655025	1	22,459,754	A	G	0.460	0.428	1.13E-04	1.140	0.034	1.067	1.219	0.532	Imputed
rs10917161	1	22,460,208	C	T	0.026	0.027	0.835	0.978	0.105	0.796	1.202	0.049	Imputed
rs1040456	1	22,460,515	A	G	0.082	0.083	0.623	0.970	0.062	0.860	1.095	0.045	Imputed
rs12139609	1	22,460,991	A	C	0.036	0.038	0.571	0.950	0.091	0.795	1.135	0.153	Imputed
rs12727309	1	22,461,627	A	G	0.134	0.133	0.821	1.011	0.050	0.917	1.115	0.637	Imputed
rs12037376	1	22,462,111	A	G	0.186	0.153	4.22E-08	1.272	0.044	1.167	1.387	0.619	Imputed
rs10799737	1	22,462,131	T	G	0.372	0.338	3.18E-05	1.157	0.035	1.080	1.240	0.909	Imputed
rs11584713	1	22,462,143	A	G	0.134	0.133	0.825	1.011	0.050	0.917	1.115	0.635	Imputed
rs12091003	1	22,462,609	A	C	0.026	0.027	0.714	0.962	0.107	0.780	1.185	0.136	Imputed

chr1:22462703:D	1	22,462,703	C	CCT	0.169	0.174	0.413	0.964	0.045	0.882	1.053	0.292	Imputed
rs11805891	1	22,463,457	G	T	0.026	0.029	0.209	0.875	0.106	0.710	1.077	0.123	Imputed
rs116278795	1	22,464,564	T	C	0.010	0.012	0.361	0.858	0.169	0.617	1.193	0.279	Imputed
rs909818	1	22,464,940	C	G	0.020	0.017	0.192	1.172	0.122	0.924	1.487	0.692	Imputed
rs909817	1	22,464,943	T	G	0.020	0.017	0.202	1.168	0.122	0.920	1.481	0.680	Imputed
rs2865174	1	22,465,160	C	T	0.082	0.083	0.708	0.977	0.061	0.867	1.102	0.062	Genotyped
rs57423947	1	22,465,629	A	G	0.026	0.029	0.191	0.870	0.106	0.706	1.072	0.115	Imputed
rs61768001	1	22,465,820	C	T	0.187	0.153	2.48E-08	1.277	0.044	1.172	1.392	0.505	Imputed
rs59633390	1	22,465,944	C	G	0.026	0.029	0.184	0.868	0.106	0.705	1.069	0.113	Imputed
rs75676035	1	22,465,945	C	T	0.013	0.013	0.840	0.970	0.151	0.722	1.303	0.074	Imputed
rs74059911	1	22,467,452	T	C	0.014	0.020	7.50E-03	0.684	0.142	0.517	0.904	0.109	Imputed
rs3820282	1	22,468,215	T	C	0.185	0.151	2.66E-08	1.278	0.044	1.172	1.393	0.544	Imputed
rs60039305	1	22,469,069	C	A	0.029	0.033	0.154	0.866	0.101	0.711	1.055	0.170	Imputed
rs183472573	1	22,469,072	A	C	0.042	0.049	0.042	0.843	0.084	0.716	0.993	0.164	Imputed
rs187711342	1	22,469,763	G	C	0.087	0.092	0.312	0.941	0.060	0.837	1.059	0.474	Imputed
rs12135916	1	22,470,115	G	A	0.256	0.262	0.385	0.967	0.039	0.896	1.043	0.877	Imputed
rs56318008	1	22,470,407	T	C	0.179	0.146	2.93E-08	1.281	0.045	1.173	1.398	0.617	Imputed
rs55938609	1	22,470,451	C	G	0.179	0.146	2.93E-08	1.281	0.045	1.173	1.398	0.617	Imputed
rs12038516	1	22,470,882	C	T	0.024	0.025	0.641	0.950	0.110	0.767	1.178	0.059	Imputed
rs78927282	1	22,471,231	G	T	0.023	0.023	0.734	0.962	0.113	0.771	1.201	0.499	Imputed
rs113155445	1	22,472,435	T	C	0.090	0.108	4.90E-04	0.815	0.059	0.727	0.915	0.404	Imputed
rs7543136	1	22,472,451	C	T	0.314	0.287	5.96E-04	1.134	0.037	1.055	1.218	0.360	Imputed
rs7519889	1	22,472,506	A	G	0.227	0.199	2.64E-05	1.186	0.041	1.095	1.285	0.745	Imputed
rs7554742	1	22,472,573	A	G	0.089	0.088	0.841	1.012	0.059	0.901	1.136	0.014	Imputed
rs12042083	1	22,472,732	A	G	0.227	0.200	3.84E-05	1.182	0.041	1.091	1.280	0.730	Genotyped
rs11809073	1	22,473,075	A	G	0.014	0.020	7.50E-03	0.684	0.142	0.517	0.904	0.109	Imputed
rs7515106	1	22,473,410	C	T	0.235	0.206	2.36E-05	1.185	0.040	1.095	1.282	0.712	Imputed
rs4655026	1	22,473,658	T	C	0.454	0.425	4.24E-04	1.127	0.034	1.055	1.205	0.738	Imputed
rs4655027	1	22,473,791	C	T	0.453	0.425	5.47E-04	1.125	0.034	1.052	1.202	0.766	Imputed
rs7529389	1	22,475,425	G	T	0.461	0.431	3.15E-04	1.130	0.034	1.057	1.208	0.815	Imputed
rs138407808	1	22,475,546	T	C	0.015	0.018	0.180	0.832	0.137	0.636	1.089	0.942	Imputed
rs7521775	1	22,475,649	T	C	0.453	0.424	4.87E-04	1.126	0.034	1.053	1.204	0.732	Imputed
rs57837158	1	22,475,709	C	G	0.012	0.010	0.163	1.243	0.156	0.916	1.687	0.681	Imputed
rs7524615	1	22,476,205	T	C	0.077	0.077	0.994	1.000	0.063	0.884	1.133	0.088	Imputed
rs113126541	1	22,476,490	G	C	0.025	0.026	0.682	0.957	0.109	0.773	1.183	0.044	Imputed
rs7518336	1	22,476,954	G	C	0.075	0.074	0.960	0.997	0.064	0.879	1.131	0.064	Imputed
rs12134434	1	22,477,206	G	A	0.025	0.026	0.737	0.965	0.108	0.781	1.192	0.046	Imputed
rs147627901	1	22,477,466	T	C	0.021	0.024	0.195	0.857	0.119	0.679	1.082	0.113	Imputed
rs7542242	1	22,477,493	T	C	0.316	0.330	0.092	0.941	0.036	0.876	1.010	0.610	Genotyped
rs7519103	1	22,477,701	T	G	0.012	0.010	0.152	1.250	0.156	0.921	1.697	0.842	Imputed

rs7544807	1	22,477,926	T	C	0.012	0.010	0.182	1.230	0.156	0.907	1.669	0.850	Imputed
rs151161614	1	22,478,350	A	G	0.012	0.013	0.447	0.890	0.154	0.657	1.204	0.343	Imputed
rs12084780	1	22,478,517	C	T	0.013	0.012	0.590	1.085	0.150	0.808	1.455	0.278	Imputed
rs58111733	1	22,479,067	T	G	0.095	0.103	0.075	0.903	0.058	0.806	1.010	0.637	Imputed
rs7548239	1	22,479,193	A	C	0.014	0.012	0.355	1.144	0.146	0.859	1.524	0.580	Imputed
rs72881075	1	22,479,378	T	C	0.043	0.050	0.059	0.855	0.083	0.726	1.006	0.474	Imputed
rs7517829	1	22,480,159	G	A	0.433	0.416	0.026	1.079	0.034	1.009	1.154	0.836	Imputed
rs10917162	1	22,480,219	C	T	0.353	0.326	2.61E-04	1.138	0.035	1.062	1.220	0.364	Imputed
rs10917163	1	22,480,241	C	T	0.351	0.322	1.39E-04	1.145	0.036	1.068	1.228	0.498	Imputed
rs4623666	1	22,480,312	G	A	0.438	0.407	1.47E-04	1.138	0.034	1.065	1.217	0.794	Genotyped
rs78492083	1	22,480,729	T	C	0.044	0.050	0.103	0.875	0.082	0.746	1.027	0.273	Imputed
rs4356020	1	22,481,906	A	G	0.025	0.021	0.120	1.188	0.111	0.956	1.475	0.568	Imputed
rs735475	1	22,482,230	G	A	0.022	0.020	0.553	1.071	0.116	0.853	1.344	0.087	Imputed
rs76941962	1	22,482,250	A	T	0.012	0.012	0.919	0.984	0.157	0.723	1.340	0.812	Imputed
rs34654575	1	22,482,530	G	A	0.083	0.095	0.016	0.863	0.061	0.766	0.973	0.460	Imputed
rs34870854	1	22,482,625	A	C	0.150	0.163	0.028	0.902	0.047	0.822	0.989	0.074	Imputed
rs12410251	1	22,482,629	T	G	0.238	0.201	1.06E-07	1.237	0.040	1.144	1.338	0.775	Imputed
rs2744701	1	22,483,584	A	G	0.356	0.372	0.036	0.929	0.035	0.867	0.995	0.635	Imputed
rs2744702	1	22,483,630	T	C	0.356	0.372	0.036	0.929	0.035	0.867	0.995	0.635	Imputed
chr1:22483649:I	1	22,483,649	TG	T	0.271	0.246	3.36E-04	1.147	0.038	1.064	1.236	0.159	Imputed
rs2807366	1	22,483,912	A	G	0.073	0.069	0.442	1.051	0.065	0.925	1.195	0.096	Imputed
rs17558605	1	22,484,545	A	G	0.152	0.165	0.033	0.905	0.047	0.825	0.992	0.111	Imputed
rs3971300	1	22,484,575	C	T	0.294	0.266	1.57E-04	1.151	0.037	1.070	1.239	0.459	Imputed
rs139121331	1	22,484,815	T	C	0.014	0.012	0.324	1.156	0.147	0.866	1.543	0.821	Imputed
rs11591214	1	22,485,138	A	G	0.134	0.138	0.577	0.973	0.049	0.883	1.072	0.052	Imputed
chr1:22485141:D	1	22,485,141	T	TCAAA	0.013	0.012	0.654	1.070	0.152	0.795	1.441	0.254	Imputed
rs2744717	1	22,485,337	G	A	0.020	0.016	0.074	1.243	0.122	0.979	1.579	0.622	Imputed
rs2807365	1	22,485,467	G	A	0.356	0.322	2.31E-05	1.162	0.035	1.084	1.245	0.978	Imputed
rs76274239	1	22,485,602	A	G	0.013	0.012	0.829	1.032	0.149	0.772	1.381	0.153	Imputed
rs12093861	1	22,485,677	G	A	0.426	0.409	0.045	1.071	0.034	1.002	1.145	0.037	Imputed
rs2807364	1	22,485,843	C	G	0.073	0.069	0.420	1.054	0.065	0.928	1.197	0.093	Imputed
rs16826658	1	22,485,871	G	T	0.412	0.396	0.050	1.070	0.034	1.000	1.145	0.014	Imputed
rs2807363	1	22,485,954	T	C	0.074	0.070	0.382	1.058	0.065	0.932	1.202	0.141	Imputed
chr1:22486012:D	1	22,486,012	T	TA	0.211	0.185	9.33E-05	1.177	0.042	1.085	1.278	0.553	Imputed
rs12094994	1	22,486,013	T	A	0.223	0.196	7.88E-05	1.175	0.041	1.085	1.273	0.771	Imputed
chr1:22486016:I	1	22,486,016	TA	T	0.049	0.057	0.038	0.851	0.078	0.731	0.991	0.257	Imputed
rs56104760	1	22,486,029	G	A	0.215	0.181	3.62E-07	1.235	0.042	1.139	1.340	0.664	Imputed
rs140531268	1	22,486,032	A	C	0.021	0.021	0.873	0.981	0.118	0.778	1.237	0.719	Imputed
rs61318332	1	22,486,091	A	G	0.047	0.052	0.117	0.882	0.080	0.754	1.032	0.112	Imputed
rs12729516	1	22,486,397	T	C	0.195	0.213	6.61E-03	0.891	0.043	0.819	0.968	0.034	Imputed

rs16826659	1	22,486,501	A	C	0.047	0.052	0.136	0.888	0.080	0.759	1.038	0.154	Genotyped
rs72865212	1	22,486,580	T	C	0.123	0.124	0.921	0.995	0.051	0.900	1.100	0.055	Imputed
rs2744721	1	22,487,036	T	C	0.369	0.386	0.032	0.928	0.035	0.866	0.994	0.974	Genotyped
rs112249410	1	22,487,054	T	C	0.013	0.012	0.737	1.052	0.150	0.784	1.411	0.119	Imputed
rs2744722	1	22,487,062	T	C	0.074	0.070	0.406	1.055	0.065	0.929	1.198	0.147	Imputed
rs1569580	1	22,487,500	T	C	0.154	0.166	0.038	0.907	0.047	0.828	0.995	0.097	Genotyped
rs149324491	1	22,488,062	A	C	0.011	0.013	0.366	0.865	0.161	0.631	1.186	0.510	Imputed
rs2807362	1	22,488,940	T	C	0.073	0.069	0.429	1.053	0.065	0.927	1.196	0.108	Genotyped
rs78651132	1	22,489,057	G	A	0.013	0.012	0.842	1.030	0.149	0.770	1.378	0.155	Imputed
rs34379276	1	22,489,200	A	G	0.139	0.155	5.92E-03	0.874	0.049	0.795	0.962	0.113	Imputed
rs2807361	1	22,489,213	C	G	0.368	0.380	0.171	0.953	0.035	0.890	1.021	0.149	Imputed
chr1:22489246:I	1	22,489,246	AG	A	0.362	0.373	0.174	0.953	0.035	0.890	1.021	0.303	Imputed
rs2744739	1	22,489,417	A	T	0.073	0.069	0.399	1.056	0.065	0.930	1.200	0.089	Imputed
rs72478520	1	22,489,567	T	C	0.212	0.178	1.32E-07	1.246	0.042	1.148	1.352	0.585	Imputed
rs149000738	1	22,490,100	A	G	0.010	0.014	0.041	0.712	0.167	0.513	0.988	0.611	Imputed
rs2807360	1	22,490,171	C	G	0.368	0.380	0.170	0.953	0.035	0.890	1.021	0.145	Imputed
rs2807359	1	22,490,209	T	C	0.073	0.069	0.399	1.056	0.065	0.930	1.200	0.089	Imputed
rs7521902	1	22,490,724	A	C	0.260	0.232	6.48E-05	1.168	0.039	1.082	1.260	0.166	Imputed
rs117881761	1	22,491,183	G	A	0.047	0.053	0.111	0.881	0.080	0.753	1.029	0.115	Imputed
rs2744755	1	22,491,330	C	T	0.355	0.364	0.260	0.961	0.035	0.897	1.030	0.117	Imputed
rs2807358	1	22,491,365	T	C	0.357	0.376	0.018	0.920	0.035	0.859	0.986	0.781	Imputed
rs35454568	1	22,491,665	T	G	0.141	0.156	0.010	0.883	0.048	0.803	0.971	0.242	Imputed
rs4654784	1	22,491,757	A	G	0.253	0.272	8.05E-03	0.902	0.039	0.836	0.974	0.785	Imputed
rs4654785	1	22,491,843	G	A	0.266	0.237	5.08E-05	1.169	0.039	1.084	1.261	0.126	Imputed
rs2807357	1	22,491,984	G	A	0.360	0.372	0.162	0.952	0.035	0.889	1.020	0.193	Genotyped
rs2223480	1	22,492,522	C	T	0.386	0.404	0.023	0.924	0.035	0.863	0.989	0.611	Imputed
rs58670122	1	22,492,613	G	A	0.144	0.147	0.725	0.983	0.048	0.895	1.080	0.146	Imputed
rs2206301	1	22,492,738	T	C	0.352	0.368	0.042	0.931	0.035	0.868	0.997	0.917	Imputed
rs3920498	1	22,492,887	C	G	0.224	0.193	1.77E-06	1.216	0.041	1.122	1.317	0.625	Imputed
rs60999980	1	22,493,090	A	G	0.084	0.085	0.953	0.997	0.061	0.885	1.122	0.022	Imputed
rs2807356	1	22,493,672	T	C	0.228	0.241	0.055	0.926	0.040	0.855	1.001	0.077	Genotyped
rs12405138	1	22,493,824	T	C	0.136	0.142	0.231	0.943	0.049	0.856	1.038	0.959	Imputed
rs4655028	1	22,493,825	C	G	0.392	0.406	0.105	0.945	0.035	0.883	1.012	0.681	Imputed
rs2807355	1	22,493,977	A	C	0.115	0.117	0.823	0.988	0.053	0.891	1.096	0.148	Genotyped
rs35142741	1	22,494,153	T	C	0.171	0.176	0.359	0.960	0.045	0.879	1.048	0.987	Imputed
rs12117004	1	22,494,258	G	A	0.122	0.127	0.401	0.958	0.052	0.866	1.059	0.869	Imputed
rs2807354	1	22,494,312	G	A	0.115	0.117	0.751	0.983	0.053	0.886	1.091	0.127	Imputed
rs2807353	1	22,494,349	T	C	0.169	0.173	0.600	0.977	0.045	0.894	1.067	0.108	Imputed
rs7534624	1	22,494,627	G	C	0.353	0.367	0.097	0.943	0.035	0.880	1.011	0.690	Imputed
rs2056972	1	22,494,652	G	A	0.115	0.117	0.766	0.984	0.053	0.887	1.092	0.124	Imputed

rs12747516	1	22,494,791	A	G	0.157	0.166	0.107	0.928	0.046	0.847	1.016	0.593	Genotyped
rs17360017	1	22,495,215	A	G	0.352	0.367	0.072	0.938	0.035	0.875	1.006	0.684	Genotyped
rs2807352	1	22,495,261	C	G	0.455	0.441	0.122	1.054	0.034	0.986	1.127	0.918	Imputed
rs2807351	1	22,495,437	A	G	0.099	0.104	0.250	0.937	0.056	0.839	1.047	0.160	Imputed
chr1:22495441:D	1	22,495,441	A	AG	0.024	0.029	0.040	0.798	0.110	0.643	0.990	0.548	Imputed
chr1:22495442:D	1	22,495,442	G	GA	0.024	0.029	0.049	0.806	0.110	0.650	1.000	0.462	Imputed
rs3960204	1	22,495,591	T	C	0.024	0.029	0.052	0.808	0.110	0.652	1.002	0.469	Imputed
rs2744698	1	22,495,601	T	C	0.158	0.155	0.685	1.019	0.046	0.930	1.116	0.268	Imputed
rs909813	1	22,496,703	G	A	0.208	0.203	0.421	1.034	0.042	0.953	1.122	0.899	Genotyped
rs79988234	1	22,496,841	T	C	0.016	0.014	0.288	1.156	0.136	0.885	1.511	0.524	Imputed
rs116592100	1	22,497,857	T	C	0.024	0.028	0.058	0.812	0.110	0.655	1.007	0.488	Imputed
rs77814031	1	22,497,907	T	C	0.083	0.082	0.859	1.011	0.061	0.896	1.140	0.917	Imputed
rs78534006	1	22,497,945	T	C	0.081	0.079	0.718	1.023	0.062	0.906	1.155	0.715	Imputed
rs4655030	1	22,498,103	T	C	0.433	0.447	0.112	0.947	0.034	0.886	1.013	0.866	Genotyped
rs2744699	1	22,498,170	T	C	0.076	0.075	0.924	1.006	0.064	0.888	1.140	0.043	Imputed
rs2744700	1	22,498,374	T	C	0.446	0.436	0.336	1.033	0.034	0.967	1.105	0.899	Imputed
rs2807369	1	22,498,451	A	G	0.075	0.074	0.877	1.010	0.064	0.891	1.145	0.050	Imputed
rs2807368	1	22,498,599	A	G	0.209	0.203	0.355	1.039	0.042	0.958	1.128	0.906	Imputed
rs2179378	1	22,498,765	A	C	0.259	0.247	0.108	1.064	0.039	0.987	1.148	0.282	Genotyped
rs1014987	1	22,498,824	G	C	0.260	0.247	0.100	1.066	0.039	0.988	1.150	0.291	Imputed
rs113932113	1	22,498,883	T	C	0.081	0.079	0.677	1.026	0.062	0.909	1.159	0.788	Imputed
rs1014986	1	22,499,222	A	G	0.157	0.154	0.651	1.021	0.047	0.932	1.119	0.261	Genotyped
rs1014985	1	22,499,255	G	T	0.491	0.478	0.135	1.052	0.034	0.984	1.124	0.252	Imputed
rs17360053	1	22,499,530	C	T	0.355	0.370	0.085	0.941	0.035	0.878	1.008	0.679	Genotyped
rs57540864	1	22,499,749	T	C	0.016	0.018	0.254	0.856	0.136	0.656	1.117	0.354	Imputed
rs80052366	1	22,499,908	T	G	0.016	0.012	0.037	1.328	0.137	1.015	1.738	0.463	Imputed
rs150103370	1	22,500,043	T	A	0.016	0.012	0.052	1.308	0.139	0.996	1.717	0.243	Imputed
rs12568557	1	22,500,228	C	T	0.046	0.044	0.707	1.031	0.081	0.880	1.208	0.548	Genotyped
rs10489158	1	22,500,396	C	T	0.046	0.044	0.769	1.024	0.081	0.874	1.200	0.551	Imputed
rs34108012	1	22,500,688	A	G	0.325	0.345	0.014	0.915	0.036	0.852	0.982	0.806	Imputed
chr1:22501357:D	1	22,501,357	A	AC	0.024	0.027	0.124	0.844	0.110	0.680	1.048	0.638	Imputed
rs77361455	1	22,501,421	A	G	0.085	0.083	0.687	1.025	0.061	0.910	1.154	0.882	Imputed
rs10917165	1	22,501,628	C	T	0.368	0.358	0.267	1.040	0.035	0.971	1.114	0.292	Genotyped
rs742356	1	22,501,846	G	A	0.259	0.247	0.116	1.063	0.039	0.985	1.147	0.331	Genotyped
rs11799918	1	22,502,000	A	G	0.109	0.111	0.654	0.976	0.054	0.878	1.086	0.769	Imputed
chr1:22502143:I	1	22,502,143	TG	T	0.361	0.349	0.189	1.047	0.035	0.977	1.122	0.329	Imputed
rs2744703	1	22,502,829	G	A	0.070	0.068	0.578	1.037	0.066	0.911	1.181	0.047	Imputed
rs114328394	1	22,502,885	C	A	0.013	0.013	0.901	0.981	0.151	0.730	1.320	0.845	Imputed
rs2807367	1	22,503,282	G	C	0.325	0.324	0.930	1.003	0.036	0.935	1.077	0.668	Imputed
rs2744704	1	22,503,550	T	A	0.442	0.458	0.044	0.934	0.034	0.874	0.998	0.264	Imputed

rs78395981	1	22,503,561	T	G	0.024	0.027	0.124	0.844	0.110	0.680	1.048	0.638	Imputed
rs35849962	1	22,503,649	G	A	0.347	0.362	0.073	0.938	0.035	0.875	1.006	0.893	Imputed
rs2744705	1	22,503,667	T	C	0.215	0.211	0.542	1.025	0.041	0.946	1.112	0.881	Imputed
rs10737465	1	22,503,776	T	C	0.309	0.306	0.757	1.011	0.037	0.941	1.087	0.378	Imputed
rs2744706	1	22,503,841	T	C	0.215	0.212	0.560	1.024	0.041	0.945	1.111	0.887	Imputed
rs2744707	1	22,503,842	T	G	0.070	0.068	0.533	1.042	0.066	0.915	1.186	0.043	Imputed
rs34026022	1	22,503,904	A	G	0.347	0.362	0.070	0.938	0.035	0.875	1.005	0.872	Imputed
rs35431795	1	22,504,017	T	C	0.024	0.027	0.138	0.849	0.111	0.684	1.054	0.593	Imputed
rs10917167	1	22,504,091	A	G	0.256	0.246	0.149	1.058	0.039	0.980	1.141	0.352	Imputed
rs2982282	1	22,504,272	A	C	0.070	0.068	0.558	1.039	0.066	0.913	1.184	0.049	Imputed
rs116332992	1	22,504,423	T	C	0.087	0.084	0.512	1.040	0.060	0.925	1.171	0.811	Imputed
rs2998278	1	22,504,514	T	C	0.071	0.070	0.690	1.026	0.066	0.903	1.167	0.061	Imputed
rs2982283	1	22,504,527	T	C	0.238	0.238	0.986	0.999	0.040	0.924	1.080	0.762	Imputed
rs12032458	1	22,504,565	G	C	0.257	0.246	0.146	1.058	0.039	0.981	1.142	0.366	Imputed
rs6695779	1	22,505,609	G	A	0.281	0.273	0.397	1.032	0.038	0.959	1.112	0.334	Imputed
rs2982284	1	22,505,742	G	C	0.214	0.211	0.586	1.023	0.041	0.943	1.109	0.827	Imputed
rs926434	1	22,506,159	A	G	0.101	0.099	0.541	1.035	0.056	0.927	1.155	0.502	Imputed
rs2982285	1	22,506,253	T	C	0.495	0.485	0.241	1.041	0.034	0.974	1.112	0.286	Genotyped
rs12023766	1	22,506,437	G	A	0.281	0.273	0.395	1.033	0.038	0.959	1.112	0.335	Imputed
rs733662	1	22,506,709	A	G	0.024	0.027	0.132	0.847	0.111	0.682	1.051	0.584	Imputed
rs2982286	1	22,506,729	G	A	0.497	0.486	0.225	1.042	0.034	0.975	1.113	0.291	Imputed
rs2998277	1	22,506,778	A	C	0.496	0.485	0.232	1.041	0.034	0.975	1.113	0.279	Imputed
rs2744708	1	22,506,851	A	G	0.494	0.484	0.267	1.038	0.034	0.972	1.109	0.221	Genotyped
rs72647429	1	22,507,293	G	C	0.070	0.068	0.610	1.034	0.066	0.908	1.178	0.039	Imputed
rs9700998	1	22,507,321	T	C	0.347	0.363	0.059	0.935	0.035	0.873	1.003	0.974	Imputed
rs2744710	1	22,507,330	G	A	0.495	0.485	0.250	1.040	0.034	0.973	1.111	0.318	Imputed
rs2744711	1	22,507,355	A	G	0.215	0.212	0.619	1.021	0.041	0.942	1.107	0.824	Imputed
rs2807346	1	22,507,423	C	T	0.495	0.485	0.250	1.040	0.034	0.973	1.111	0.318	Imputed
rs114515688	1	22,507,458	T	C	0.012	0.011	0.652	1.072	0.155	0.792	1.453	0.717	Imputed
rs11803232	1	22,507,576	A	C	0.024	0.027	0.130	0.846	0.110	0.681	1.050	0.580	Imputed
rs4535973	1	22,507,655	T	C	0.347	0.363	0.059	0.935	0.035	0.872	1.003	0.976	Imputed
rs2092315	1	22,507,684	T	C	0.257	0.246	0.155	1.057	0.039	0.979	1.140	0.425	Imputed
chr1:22507753:D	1	22,507,753	C	CAGCTAATT	0.233	0.227	0.416	1.033	0.040	0.955	1.118	0.432	Imputed
rs2092316	1	22,507,901	T	C	0.275	0.269	0.484	1.027	0.038	0.953	1.106	0.228	Imputed
rs2870451	1	22,507,942	T	C	0.222	0.219	0.580	1.023	0.041	0.944	1.108	0.839	Imputed
rs2092317	1	22,507,949	T	C	0.274	0.268	0.506	1.026	0.038	0.952	1.105	0.232	Imputed
rs3971296	1	22,507,980	T	C	0.274	0.268	0.513	1.025	0.038	0.952	1.104	0.250	Imputed
rs192885220	1	22,508,084	A	C	0.016	0.018	0.384	0.890	0.134	0.684	1.158	0.318	Imputed
rs2092318	1	22,508,127	G	A	0.215	0.212	0.565	1.024	0.041	0.945	1.110	0.840	Imputed
rs6678085	1	22,508,799	T	C	0.349	0.365	0.048	0.932	0.035	0.870	1.000	0.881	Imputed

rs116190975	1	22,508,862	C	T	0.092	0.090	0.594	1.032	0.059	0.920	1.157	0.832	Imputed
rs2807370	1	22,508,922	C	T	0.495	0.485	0.235	1.041	0.034	0.974	1.112	0.319	Imputed
rs2807371	1	22,509,206	T	C	0.495	0.485	0.235	1.041	0.034	0.974	1.112	0.319	Imputed
rs2744712	1	22,509,251	A	G	0.215	0.212	0.565	1.024	0.041	0.945	1.110	0.840	Genotyped
rs2807372	1	22,509,890	A	G	0.213	0.209	0.597	1.022	0.041	0.943	1.108	0.672	Imputed
rs34918796	1	22,509,906	T	G	0.349	0.365	0.050	0.933	0.035	0.870	1.000	0.847	Imputed
chr1:22510054:I	1	22,510,054	TTA	T	0.257	0.246	0.142	1.059	0.039	0.981	1.142	0.427	Imputed
rs36014107	1	22,510,101	G	T	0.046	0.055	0.016	0.825	0.080	0.705	0.965	0.644	Imputed
rs2807373	1	22,510,784	G	T	0.495	0.485	0.247	1.040	0.034	0.973	1.111	0.311	Imputed
rs2807374	1	22,510,809	C	G	0.500	0.490	0.272	1.038	0.034	0.971	1.109	0.438	Imputed
rs2744713	1	22,511,050	G	T	0.495	0.485	0.245	1.040	0.034	0.973	1.111	0.312	Imputed
rs79503176	1	22,511,059	C	T	0.087	0.084	0.516	1.040	0.060	0.924	1.170	0.834	Imputed
rs2807375	1	22,511,418	C	T	0.495	0.485	0.245	1.040	0.034	0.973	1.111	0.312	Imputed
rs12119429	1	22,511,594	C	T	0.257	0.246	0.148	1.058	0.039	0.980	1.141	0.433	Imputed
rs2744714	1	22,511,922	A	G	0.071	0.068	0.532	1.042	0.066	0.916	1.186	0.029	Imputed
rs2744715	1	22,512,194	A	G	0.215	0.212	0.581	1.023	0.041	0.944	1.109	0.787	Imputed
rs2998276	1	22,512,470	T	C	0.217	0.215	0.630	1.020	0.041	0.941	1.105	0.840	Imputed
rs140767127	1	22,512,667	A	G	0.010	0.012	0.282	0.833	0.170	0.597	1.161	0.230	Imputed
rs77773110	1	22,512,818	T	C	0.089	0.086	0.486	1.042	0.059	0.928	1.171	0.742	Imputed
rs2807376	1	22,512,847	C	T	0.236	0.234	0.735	1.014	0.040	0.937	1.096	0.985	Genotyped
rs12743883	1	22,513,011	A	G	0.406	0.399	0.328	1.034	0.034	0.967	1.107	0.448	Imputed
rs61766864	1	22,513,090	C	G	0.253	0.246	0.358	1.037	0.039	0.960	1.119	0.113	Imputed
rs2998275	1	22,513,275	T	C	0.069	0.068	0.771	1.020	0.067	0.895	1.162	0.034	Imputed
rs7545266	1	22,513,284	C	T	0.306	0.304	0.774	1.011	0.037	0.940	1.086	0.117	Imputed
rs7522122	1	22,513,315	A	G	0.084	0.082	0.436	1.049	0.061	0.931	1.182	0.385	Imputed
rs2998274	1	22,513,384	T	C	0.069	0.067	0.720	1.024	0.067	0.899	1.167	0.043	Imputed
rs116674867	1	22,513,385	A	G	0.018	0.019	0.706	0.953	0.127	0.744	1.221	0.309	Imputed
rs10917168	1	22,513,890	T	A	0.259	0.255	0.684	1.016	0.039	0.942	1.096	0.067	Imputed
rs2744716	1	22,514,070	T	G	0.158	0.159	0.898	0.994	0.046	0.908	1.089	0.215	Imputed
rs34143150	1	22,514,071	T	C	0.044	0.050	0.068	0.862	0.082	0.734	1.011	0.570	Imputed
rs12132705	1	22,514,137	A	C	0.017	0.017	0.986	1.002	0.132	0.773	1.299	0.930	Imputed
rs10753531	1	22,514,236	T	C	0.273	0.275	0.796	0.990	0.038	0.919	1.067	0.069	Genotyped
rs6660027	1	22,514,676	G	A	0.338	0.330	0.317	1.037	0.036	0.966	1.112	0.014	Imputed
rs4654786	1	22,515,032	T	C	0.310	0.304	0.380	1.033	0.037	0.961	1.109	0.124	Imputed
rs72647433	1	22,515,497	A	G	0.015	0.016	0.810	0.967	0.138	0.739	1.267	0.459	Imputed
chr1:22515627:D	1	22,515,627	T	TG	0.084	0.085	0.992	1.001	0.061	0.888	1.127	0.936	Imputed
rs7531383	1	22,515,992	A	G	0.078	0.074	0.344	1.062	0.063	0.938	1.203	0.206	Genotyped
rs2807377	1	22,516,420	T	C	0.069	0.066	0.466	1.050	0.067	0.921	1.196	0.080	Imputed
rs12066045	1	22,517,508	A	G	0.079	0.075	0.294	1.068	0.063	0.944	1.209	0.240	Imputed
rs72647436	1	22,517,559	G	T	0.019	0.020	0.644	0.945	0.124	0.741	1.203	0.311	Imputed

rs114895449	1	22,518,187	T	G	0.084	0.085	0.996	1.000	0.061	0.888	1.127	0.990	Imputed
rs2998273	1	22,518,382	A	G	0.068	0.066	0.601	1.036	0.067	0.908	1.181	0.057	Genotyped
rs11579887	1	22,518,578	A	G	0.078	0.074	0.336	1.063	0.063	0.939	1.204	0.255	Genotyped
rs10917169	1	22,518,750	G	T	0.077	0.074	0.389	1.056	0.064	0.933	1.196	0.284	Imputed
rs10917170	1	22,518,817	T	C	0.090	0.084	0.147	1.090	0.059	0.970	1.225	0.496	Imputed
rs36095869	1	22,519,321	G	A	0.259	0.255	0.598	1.021	0.039	0.946	1.101	0.054	Imputed
rs10799738	1	22,519,423	T	C	0.078	0.075	0.325	1.064	0.063	0.940	1.205	0.313	Imputed
rs74636997	1	22,519,492	T	C	0.091	0.093	0.591	0.969	0.059	0.863	1.087	0.924	Imputed
rs2998272	1	22,519,648	T	C	0.237	0.234	0.630	1.019	0.040	0.943	1.102	0.598	Imputed
rs72647438	1	22,520,321	G	A	0.078	0.075	0.366	1.059	0.063	0.935	1.199	0.198	Imputed
chr1:22520815:D	1	22,520,815	A	AG	0.077	0.074	0.386	1.057	0.064	0.933	1.197	0.285	Imputed
chr1:22521272:I	1	22,521,272	GTC	G	0.346	0.357	0.144	0.950	0.036	0.886	1.018	0.427	Imputed
rs4655031	1	22,521,275	T	A	0.282	0.298	0.033	0.923	0.038	0.857	0.994	0.771	Imputed
rs2982288	1	22,521,570	T	A	0.068	0.066	0.585	1.037	0.067	0.909	1.183	0.044	Imputed
rs2998271	1	22,521,603	A	G	0.068	0.066	0.585	1.037	0.067	0.909	1.183	0.044	Imputed
rs12123590	1	22,521,622	G	A	0.079	0.075	0.301	1.068	0.063	0.943	1.208	0.237	Imputed
rs2998270	1	22,522,007	C	T	0.068	0.066	0.591	1.037	0.067	0.909	1.182	0.045	Imputed
rs7511788	1	22,522,053	T	C	0.259	0.254	0.596	1.021	0.039	0.946	1.101	0.046	Imputed
rs7533740	1	22,522,062	A	G	0.259	0.254	0.549	1.023	0.039	0.949	1.104	0.051	Imputed
rs2807313	1	22,522,200	T	G	0.068	0.066	0.581	1.038	0.067	0.910	1.183	0.044	Imputed
rs11585923	1	22,522,204	T	C	0.077	0.074	0.397	1.055	0.064	0.932	1.195	0.297	Imputed
rs10917171	1	22,522,336	C	T	0.079	0.075	0.301	1.067	0.063	0.943	1.208	0.236	Imputed
rs10799739	1	22,522,352	A	C	0.079	0.075	0.304	1.067	0.063	0.943	1.207	0.235	Imputed
rs12734979	1	22,522,927	C	T	0.259	0.254	0.586	1.021	0.039	0.947	1.102	0.047	Genotyped
rs145953753	1	22,523,189	A	G	0.022	0.021	0.717	1.043	0.117	0.830	1.311	0.322	Imputed
rs2744718	1	22,523,727	T	C	0.157	0.153	0.529	1.030	0.047	0.940	1.128	0.521	Imputed
rs10917173	1	22,524,136	G	A	0.078	0.074	0.322	1.065	0.063	0.941	1.206	0.228	Imputed
chr1:22524854:D	1	22,524,854	G	GC	0.019	0.016	0.228	1.164	0.126	0.909	1.491	0.400	Imputed
rs2982289	1	22,525,126	T	C	0.068	0.066	0.628	1.033	0.067	0.906	1.178	0.047	Imputed
rs2982290	1	22,525,218	C	G	0.068	0.066	0.628	1.033	0.067	0.906	1.178	0.047	Imputed
rs75139787	1	22,525,462	G	A	0.092	0.095	0.565	0.967	0.059	0.862	1.084	0.932	Imputed
rs114702719	1	22,525,865	C	G	0.092	0.095	0.565	0.967	0.059	0.862	1.084	0.932	Imputed
rs2998269	1	22,525,966	G	A	0.068	0.066	0.628	1.033	0.067	0.906	1.178	0.047	Imputed
rs12036482	1	22,526,327	A	G	0.258	0.253	0.566	1.023	0.039	0.948	1.103	0.041	Imputed
rs2870447	1	22,526,730	T	C	0.032	0.035	0.372	0.918	0.095	0.762	1.107	0.363	Imputed
rs2870446	1	22,526,814	T	G	0.235	0.228	0.267	1.045	0.040	0.967	1.131	0.883	Imputed
rs10799740	1	22,527,931	A	G	0.078	0.074	0.319	1.065	0.063	0.941	1.206	0.244	Imputed
rs139827460	1	22,528,074	T	C	0.015	0.017	0.324	0.872	0.139	0.664	1.144	0.157	Imputed
rs142072330	1	22,528,108	T	C	0.089	0.091	0.593	0.969	0.059	0.862	1.088	0.843	Imputed
rs150709382	1	22,528,145	G	A	0.018	0.015	0.174	1.193	0.129	0.926	1.538	0.056	Imputed

rs77364469	1	22,528,531	A	G	0.084	0.085	0.950	0.996	0.061	0.884	1.122	0.891	Imputed
rs2744719	1	22,529,767	C	T	0.234	0.228	0.303	1.042	0.040	0.964	1.127	0.759	Imputed
rs2744720	1	22,529,868	C	T	0.233	0.228	0.336	1.039	0.040	0.961	1.124	0.805	Genotyped
rs17356038	1	22,530,581	G	A	0.034	0.033	0.724	1.034	0.094	0.860	1.243	0.766	Imputed
rs115635878	1	22,531,044	A	G	0.021	0.021	0.767	0.966	0.117	0.768	1.216	0.131	Imputed
rs75526932	1	22,531,051	T	C	0.035	0.029	0.038	1.212	0.093	1.010	1.454	0.830	Imputed
rs926435	1	22,531,134	T	C	0.091	0.094	0.559	0.966	0.059	0.861	1.084	0.857	Imputed
rs115167425	1	22,531,419	T	C	0.025	0.019	3.94E-03	1.369	0.110	1.104	1.697	0.257	Imputed
rs79364962	1	22,531,542	A	G	0.065	0.062	0.533	1.044	0.069	0.912	1.196	0.023	Imputed
rs17356059	1	22,531,553	T	C	0.347	0.359	0.129	0.948	0.036	0.884	1.016	0.348	Genotyped
rs2807350	1	22,531,906	A	G	0.234	0.228	0.280	1.044	0.040	0.966	1.129	0.837	Imputed
rs114268629	1	22,532,007	C	T	0.027	0.028	0.625	0.950	0.105	0.773	1.167	0.612	Imputed
rs35810629	1	22,532,057	C	T	0.034	0.033	0.662	1.042	0.094	0.867	1.252	0.733	Imputed
rs2807349	1	22,532,190	T	G	0.068	0.066	0.633	1.033	0.067	0.905	1.178	0.048	Imputed
rs193057269	1	22,532,848	G	T	0.013	0.014	0.544	0.913	0.150	0.681	1.224	0.990	Imputed
rs2807348	1	22,533,106	A	G	0.235	0.228	0.258	1.046	0.040	0.967	1.132	0.766	Genotyped
rs67504988	1	22,533,161	T	G	0.354	0.366	0.109	0.945	0.035	0.882	1.013	0.324	Imputed
rs56365040	1	22,533,564	A	G	0.040	0.040	0.843	1.017	0.086	0.859	1.205	0.998	Imputed
rs2807347	1	22,533,608	A	C	0.144	0.144	0.974	1.002	0.048	0.911	1.101	0.781	Imputed
rs3765340	1	22,534,242	T	C	0.314	0.308	0.594	1.020	0.036	0.949	1.095	0.822	Genotyped
rs3765341	1	22,534,333	A	G	0.029	0.026	0.332	1.104	0.101	0.905	1.346	0.168	Imputed
rs3765342	1	22,534,551	C	G	0.247	0.250	0.761	0.988	0.039	0.915	1.067	0.540	Imputed
rs72647442	1	22,534,751	A	G	0.111	0.113	0.602	0.972	0.054	0.875	1.081	0.320	Imputed
rs115963111	1	22,534,928	T	C	0.019	0.021	0.524	0.925	0.123	0.727	1.176	0.385	Imputed
rs2744723	1	22,535,288	T	C	0.075	0.078	0.600	0.967	0.064	0.853	1.096	0.138	Imputed
rs2473246	1	22,535,399	T	C	0.156	0.152	0.619	1.024	0.047	0.934	1.122	0.541	Imputed
rs7513455	1	22,535,413	A	G	0.153	0.151	0.823	1.011	0.047	0.922	1.108	0.590	Genotyped
rs2473247	1	22,535,811	T	C	0.317	0.320	0.706	0.986	0.036	0.919	1.059	0.773	Imputed
rs115633394	1	22,536,139	A	G	0.013	0.013	0.674	1.065	0.149	0.796	1.424	0.285	Imputed
rs17356087	1	22,536,534	C	T	0.116	0.118	0.706	0.980	0.053	0.884	1.087	0.066	Imputed
rs2473248	1	22,536,643	T	C	0.126	0.125	0.837	1.010	0.051	0.915	1.117	0.369	Imputed
rs4654788	1	22,536,646	A	G	0.458	0.461	0.765	0.990	0.034	0.926	1.058	0.671	Imputed
rs4655032	1	22,536,899	C	T	0.292	0.296	0.688	0.985	0.037	0.916	1.060	0.714	Imputed
rs2473249	1	22,537,071	T	C	0.460	0.461	0.876	0.995	0.034	0.931	1.063	0.755	Genotyped
rs76142969	1	22,537,373	A	G	0.024	0.025	0.509	0.930	0.110	0.749	1.154	0.281	Imputed
rs114484970	1	22,537,425	C	G	0.034	0.030	0.088	1.173	0.094	0.976	1.409	0.748	Imputed
rs67895977	1	22,537,513	A	G	0.191	0.196	0.543	0.974	0.043	0.895	1.060	0.681	Imputed
rs2473250	1	22,537,649	G	A	0.168	0.166	0.831	1.010	0.045	0.924	1.103	0.377	Genotyped
rs141115036	1	22,537,992	C	A	0.023	0.025	0.312	0.892	0.113	0.716	1.113	0.866	Imputed
rs2473251	1	22,538,028	A	G	0.376	0.366	0.337	1.034	0.035	0.966	1.107	0.548	Imputed

rs2473252	1	22,538,597	G	A	0.376	0.366	0.346	1.033	0.035	0.965	1.107	0.572	Genotyped
rs6426742	1	22,538,629	A	C	0.307	0.313	0.566	0.979	0.037	0.911	1.052	0.688	Imputed
rs2473253	1	22,538,787	T	C	0.155	0.150	0.577	1.027	0.047	0.936	1.125	0.271	Imputed
rs2744724	1	22,538,901	A	G	0.126	0.126	0.881	1.008	0.051	0.912	1.113	0.385	Imputed
rs2473254	1	22,539,052	G	A	0.461	0.462	0.858	0.994	0.034	0.930	1.062	0.664	Imputed
rs2473255	1	22,539,081	T	C	0.153	0.149	0.694	1.019	0.047	0.929	1.117	0.337	Imputed
rs35778318	1	22,539,257	T	C	0.308	0.313	0.613	0.982	0.037	0.914	1.055	0.750	Imputed
rs2744725	1	22,539,323	T	C	0.127	0.126	0.849	1.010	0.051	0.914	1.116	0.373	Imputed
rs2473256	1	22,539,529	A	G	0.461	0.462	0.882	0.995	0.034	0.931	1.063	0.676	Imputed
rs2744726	1	22,539,571	C	A	0.126	0.125	0.869	1.008	0.051	0.913	1.114	0.394	Imputed
chr1:22539601:I	1	22,539,601	CG	C	0.016	0.018	0.421	0.898	0.134	0.691	1.167	0.670	Imputed
chr1:22539602:I	1	22,539,602	CG	C	0.457	0.458	0.861	0.994	0.034	0.930	1.063	0.905	Imputed
rs2473257	1	22,539,641	G	C	0.462	0.463	0.931	0.997	0.034	0.933	1.066	0.614	Imputed
rs4655033	1	22,539,689	C	A	0.308	0.313	0.621	0.982	0.037	0.914	1.055	0.760	Imputed
rs12080095	1	22,539,813	G	T	0.224	0.218	0.427	1.033	0.041	0.954	1.118	0.845	Genotyped
rs2473258	1	22,539,876	T	A	0.152	0.149	0.761	1.014	0.047	0.925	1.113	0.292	Imputed
rs12746131	1	22,540,117	T	C	0.224	0.218	0.419	1.033	0.041	0.954	1.119	0.856	Imputed
rs1883418	1	22,540,161	A	G	0.461	0.462	0.873	0.995	0.034	0.931	1.063	0.672	Imputed
chr1:22540240:D	1	22,540,240	C	CAT	0.454	0.456	0.732	0.988	0.034	0.925	1.057	0.704	Imputed
chr1:22540592:D	1	22,540,592	C	CAT	0.460	0.461	0.847	0.994	0.034	0.930	1.062	0.633	Imputed
rs2744728	1	22,540,918	T	C	0.125	0.126	0.818	0.988	0.051	0.894	1.092	0.371	Imputed
chr1:22541009:D	1	22,541,009	C	CAG	0.153	0.149	0.683	1.019	0.047	0.929	1.118	0.332	Imputed
chr1:22541076:I	1	22,541,076	CATTT	C	0.307	0.313	0.595	0.981	0.037	0.913	1.054	0.723	Imputed
rs71638832	1	22,541,278	T	C	0.266	0.268	0.926	0.996	0.038	0.924	1.074	0.993	Imputed
rs10917174	1	22,541,336	A	G	0.223	0.217	0.411	1.034	0.041	0.955	1.120	0.900	Imputed
rs114321214	1	22,541,518	C	G	0.017	0.017	0.893	0.982	0.132	0.759	1.272	0.544	Imputed
rs2473259	1	22,541,978	A	G	0.456	0.458	0.826	0.993	0.034	0.929	1.061	0.733	Imputed
rs2505720	1	22,542,009	C	T	0.462	0.464	0.852	0.994	0.034	0.930	1.062	0.680	Imputed
rs2473260	1	22,542,184	T	C	0.348	0.343	0.537	1.022	0.036	0.953	1.096	0.411	Imputed
rs3004222	1	22,542,339	C	A	0.384	0.380	0.854	1.006	0.035	0.940	1.078	0.928	Imputed
rs2473261	1	22,542,340	C	A	0.197	0.195	0.794	1.011	0.043	0.930	1.100	0.712	Imputed
rs2505722	1	22,542,445	G	T	0.485	0.482	0.826	1.007	0.034	0.943	1.077	0.888	Genotyped
chr1:22542822:D	1	22,542,822	T	TGAGGGACTCTA	0.013	0.012	0.613	1.079	0.152	0.802	1.453	0.403	Imputed
rs2473263	1	22,543,364	A	G	0.465	0.461	0.714	1.012	0.034	0.947	1.082	0.739	Imputed
rs78416576	1	22,543,368	T	C	0.111	0.102	0.104	1.092	0.054	0.982	1.215	0.989	Imputed
rs2473264	1	22,543,417	C	G	0.153	0.149	0.760	1.015	0.047	0.925	1.113	0.355	Imputed
rs10917175	1	22,543,483	A	G	0.224	0.218	0.444	1.032	0.041	0.953	1.117	0.874	Imputed
rs2473265	1	22,543,492	G	A	0.464	0.461	0.738	1.011	0.034	0.946	1.081	0.727	Imputed
rs11576292	1	22,543,589	G	C	0.151	0.149	0.849	1.009	0.047	0.920	1.107	0.311	Imputed
rs6658912	1	22,543,789	C	A	0.340	0.336	0.658	1.016	0.036	0.947	1.090	0.316	Imputed

chr1:22543985:D	1	22,543,985	AATCT	A	0.478	0.476	0.890	1.005	0.034	0.940	1.074	0.725	Imputed
rs113571562	1	22,544,199	G	A	0.116	0.118	0.742	0.983	0.053	0.886	1.090	0.092	Imputed
rs116191825	1	22,544,452	T	C	0.116	0.118	0.706	0.980	0.053	0.884	1.087	0.087	Imputed
rs115275552	1	22,544,993	T	C	0.036	0.039	0.279	0.907	0.091	0.759	1.083	0.612	Imputed
rs760917	1	22,545,088	A	T	0.459	0.461	0.868	0.994	0.034	0.930	1.063	0.677	Imputed
rs2807345	1	22,545,138	A	G	0.074	0.077	0.585	0.966	0.064	0.851	1.095	0.133	Imputed
rs10917176	1	22,545,297	T	C	0.224	0.218	0.422	1.033	0.041	0.954	1.119	0.875	Imputed
rs12735643	1	22,545,546	G	C	0.224	0.218	0.422	1.033	0.041	0.954	1.119	0.875	Imputed
rs760918	1	22,545,737	G	C	0.307	0.311	0.681	0.985	0.037	0.917	1.058	0.770	Imputed
rs2807344	1	22,546,066	A	C	0.074	0.077	0.575	0.965	0.064	0.851	1.094	0.134	Imputed
rs2744729	1	22,546,110	T	C	0.125	0.125	0.995	1.000	0.051	0.904	1.105	0.391	Imputed
rs2998265	1	22,546,753	T	C	0.028	0.029	0.835	0.979	0.102	0.802	1.195	0.543	Imputed
rs7552560	1	22,547,290	C	A	0.307	0.311	0.657	0.984	0.037	0.916	1.057	0.731	Genotyped
rs1569583	1	22,547,352	C	T	0.342	0.337	0.594	1.019	0.036	0.950	1.093	0.335	Genotyped
rs2505709	1	22,547,479	T	C	0.460	0.461	0.934	0.997	0.034	0.933	1.066	0.723	Imputed
rs2807343	1	22,547,679	G	T	0.074	0.077	0.585	0.966	0.064	0.851	1.095	0.133	Imputed
rs2744730	1	22,547,685	G	A	0.074	0.077	0.585	0.966	0.064	0.851	1.095	0.133	Imputed
rs2473267	1	22,547,689	A	G	0.460	0.461	0.937	0.997	0.034	0.933	1.066	0.725	Imputed
rs7520763	1	22,547,980	A	G	0.223	0.217	0.404	1.035	0.041	0.955	1.120	0.972	Imputed
rs7544280	1	22,548,252	T	C	0.224	0.218	0.390	1.036	0.041	0.956	1.121	0.884	Imputed
rs190680037	1	22,548,333	C	A	0.023	0.021	0.467	1.085	0.112	0.870	1.352	0.167	Imputed
rs7513223	1	22,548,342	C	T	0.307	0.311	0.766	0.989	0.037	0.921	1.063	0.949	Imputed
chr1:22548598:D	1	22,548,598	T	TG	0.016	0.013	0.109	1.245	0.138	0.951	1.630	0.073	Imputed
rs2744731	1	22,548,819	C	T	0.301	0.295	0.499	1.025	0.037	0.954	1.102	0.292	Imputed
rs2744732	1	22,548,994	T	C	0.075	0.077	0.783	0.983	0.064	0.867	1.114	0.138	Imputed
rs71638833	1	22,549,094	A	G	0.278	0.287	0.312	0.963	0.038	0.894	1.036	0.956	Imputed
rs2744733	1	22,549,135	T	C	0.099	0.104	0.328	0.946	0.056	0.847	1.057	0.663	Genotyped
rs2473268	1	22,549,172	A	G	0.430	0.437	0.426	0.973	0.034	0.910	1.041	0.575	Imputed
rs2491213	1	22,549,397	A	G	0.431	0.437	0.462	0.975	0.034	0.912	1.043	0.513	Imputed
rs2807336	1	22,549,666	A	G	0.233	0.239	0.440	0.970	0.040	0.897	1.049	0.635	Imputed
rs2744734	1	22,549,729	T	C	0.233	0.239	0.438	0.969	0.040	0.896	1.048	0.648	Genotyped
rs17356177	1	22,549,764	T	C	0.050	0.056	0.105	0.883	0.077	0.759	1.027	0.906	Imputed
rs2103637	1	22,549,843	A	G	0.233	0.239	0.440	0.970	0.040	0.897	1.049	0.635	Imputed
rs2744735	1	22,550,049	T	C	0.076	0.077	0.893	0.992	0.064	0.875	1.123	0.152	Imputed
rs1883419	1	22,550,050	A	G	0.233	0.239	0.448	0.970	0.040	0.897	1.049	0.640	Imputed
rs12408254	1	22,550,357	G	A	0.258	0.247	0.126	1.061	0.039	0.984	1.145	0.694	Imputed
rs732268	1	22,550,427	T	C	0.233	0.239	0.440	0.970	0.040	0.897	1.049	0.635	Imputed
rs732266	1	22,550,624	T	C	0.431	0.437	0.479	0.976	0.034	0.913	1.044	0.470	Imputed
rs2010042	1	22,550,659	A	G	0.233	0.239	0.440	0.970	0.040	0.897	1.049	0.635	Imputed
rs2010062	1	22,550,958	G	T	0.234	0.240	0.457	0.971	0.040	0.898	1.050	0.691	Imputed

rs2505710	1	22,551,281	T	C	0.155	0.154	0.963	0.998	0.047	0.910	1.094	0.214	Imputed
rs765131	1	22,551,371	G	A	0.258	0.247	0.128	1.061	0.039	0.983	1.144	0.682	Imputed
rs2744736	1	22,551,506	T	C	0.076	0.077	0.887	0.991	0.064	0.875	1.123	0.153	Imputed
rs10917177	1	22,551,518	G	C	0.017	0.014	0.123	1.223	0.131	0.946	1.581	0.375	Imputed
rs2744737	1	22,551,582	T	C	0.076	0.077	0.887	0.991	0.064	0.875	1.123	0.153	Imputed
rs76332980	1	22,551,583	A	G	0.016	0.013	0.105	1.248	0.138	0.953	1.634	0.071	Imputed
rs2473270	1	22,551,743	C	T	0.155	0.154	0.957	1.003	0.047	0.915	1.099	0.236	Imputed
rs2807314	1	22,551,749	G	A	0.493	0.486	0.432	1.027	0.034	0.961	1.097	0.980	Imputed
rs7525517	1	22,551,779	A	T	0.275	0.283	0.380	0.967	0.038	0.898	1.042	0.824	Imputed
rs2744738	1	22,551,813	T	C	0.492	0.486	0.499	1.023	0.034	0.958	1.093	0.933	Genotyped
rs2505711	1	22,551,925	A	G	0.431	0.437	0.476	0.976	0.034	0.913	1.044	0.469	Imputed
rs72867070	1	22,552,188	T	C	0.116	0.120	0.541	0.968	0.053	0.873	1.074	0.140	Imputed
rs1124793	1	22,552,441	T	C	0.234	0.239	0.529	0.975	0.040	0.902	1.055	0.637	Imputed
rs1569584	1	22,552,466	A	G	0.155	0.154	0.990	1.001	0.047	0.913	1.097	0.195	Imputed
rs1806422	1	22,552,736	A	G	0.155	0.154	0.963	0.998	0.047	0.910	1.094	0.214	Imputed
rs2807378	1	22,552,937	T	C	0.076	0.077	0.887	0.991	0.064	0.875	1.123	0.153	Imputed
chr1:22552948:I	1	22,552,948	T	TGA	0.390	0.396	0.421	0.973	0.035	0.909	1.041	0.220	Imputed
rs139029704	1	22,552,964	G	A	0.025	0.026	0.721	0.962	0.109	0.777	1.191	0.411	Imputed
rs3856181	1	22,552,969	G	C	0.276	0.283	0.410	0.969	0.038	0.900	1.044	0.857	Imputed
rs1006940	1	22,553,138	G	A	0.233	0.239	0.443	0.970	0.040	0.897	1.049	0.637	Imputed
chr1:22553160:D	1	22,553,160	A	AAAC	0.055	0.060	0.214	0.912	0.074	0.788	1.055	0.142	Imputed
rs12749758	1	22,553,181	A	C	0.273	0.282	0.313	0.962	0.038	0.894	1.037	0.962	Imputed
rs2807315	1	22,553,338	A	G	0.076	0.077	0.887	0.991	0.064	0.875	1.123	0.153	Imputed
rs75572164	1	22,553,390	C	A	0.022	0.017	0.025	1.302	0.119	1.032	1.642	0.562	Imputed
rs12749135	1	22,553,478	T	G	0.276	0.283	0.399	0.969	0.038	0.899	1.043	0.825	Genotyped
rs116617703	1	22,553,555	T	C	0.024	0.024	0.783	0.970	0.111	0.781	1.205	0.258	Imputed
rs2807316	1	22,553,578	A	C	0.076	0.077	0.893	0.992	0.064	0.875	1.123	0.152	Imputed
rs3004221	1	22,553,837	A	G	0.076	0.077	0.887	0.991	0.064	0.875	1.123	0.153	Imputed
chr1:22554000:D	1	22,554,000	TTTCC	T	0.410	0.414	0.540	0.979	0.034	0.915	1.047	0.083	Imputed
chr1:22554006:D	1	22,554,006	T	TCC	0.350	0.355	0.674	0.985	0.035	0.919	1.056	0.375	Imputed
rs12065981	1	22,554,015	T	C	0.263	0.250	0.091	1.067	0.039	0.990	1.151	0.506	Imputed
rs7517955	1	22,554,088	C	T	0.484	0.494	0.192	0.957	0.034	0.895	1.023	0.431	Imputed
rs2807319	1	22,554,176	A	G	0.122	0.125	0.541	0.969	0.052	0.876	1.072	0.533	Imputed
rs2807320	1	22,554,181	A	C	0.235	0.243	0.270	0.957	0.040	0.885	1.035	0.617	Imputed
rs112191583	1	22,554,378	C	T	0.020	0.019	0.696	1.049	0.121	0.827	1.330	0.495	Imputed
rs1033455	1	22,554,625	A	G	0.388	0.392	0.516	0.978	0.035	0.913	1.047	0.189	Imputed
rs2807321	1	22,554,891	A	C	0.076	0.077	0.842	0.987	0.064	0.872	1.119	0.143	Imputed
rs10917178	1	22,555,288	T	G	0.495	0.499	0.514	0.978	0.034	0.915	1.045	0.992	Imputed
chr1:22555504:D	1	22,555,504	C	CT	0.022	0.025	0.273	0.882	0.114	0.705	1.103	0.051	Imputed
rs115913437	1	22,555,505	C	T	0.139	0.146	0.206	0.940	0.049	0.854	1.035	0.457	Imputed

rs143158078	1	22,555,507	A	G	0.016	0.014	0.485	1.099	0.136	0.842	1.435	0.439	Imputed
rs148244922	1	22,555,545	T	C	0.009	0.011	0.358	0.852	0.174	0.606	1.199	0.518	Imputed
rs2807322	1	22,555,597	A	G	0.078	0.077	0.876	1.010	0.063	0.892	1.143	0.162	Imputed
rs2491210	1	22,555,993	T	G	0.150	0.149	0.944	0.997	0.047	0.908	1.094	0.198	Genotyped
rs12751606	1	22,556,224	C	T	0.040	0.040	0.822	1.020	0.086	0.861	1.207	0.292	Imputed
chr1:22556238:I	1	22,556,238	T	TCA	0.414	0.419	0.491	0.977	0.034	0.913	1.045	0.444	Imputed
rs79006239	1	22,556,398	C	G	0.012	0.011	0.755	1.050	0.157	0.772	1.428	0.462	Imputed
rs10917179	1	22,556,442	A	T	0.491	0.496	0.564	0.981	0.034	0.918	1.048	0.904	Imputed
rs6670439	1	22,556,706	T	C	0.230	0.240	0.172	0.947	0.040	0.875	1.024	0.286	Imputed
rs10917180	1	22,556,740	C	T	0.038	0.033	0.138	1.143	0.090	0.959	1.362	0.135	Imputed
rs10917181	1	22,556,744	T	A	0.278	0.264	0.055	1.075	0.038	0.998	1.158	0.374	Imputed
rs72647459	1	22,556,776	A	G	0.229	0.240	0.162	0.945	0.040	0.874	1.023	0.322	Imputed
rs72647460	1	22,557,354	T	C	0.076	0.075	0.945	1.004	0.064	0.886	1.139	0.279	Imputed
rs2998264	1	22,557,479	A	G	0.201	0.205	0.577	0.977	0.042	0.899	1.061	0.126	Imputed
rs12747857	1	22,558,027	T	C	0.229	0.240	0.151	0.944	0.040	0.872	1.021	0.307	Imputed
rs77554754	1	22,558,048	A	G	0.029	0.029	0.817	0.977	0.101	0.802	1.190	0.140	Imputed
rs114268961	1	22,558,062	T	C	0.027	0.020	1.47E-03	1.398	0.106	1.135	1.723	0.132	Imputed
rs2223482	1	22,558,074	T	C	0.458	0.467	0.332	0.968	0.034	0.905	1.034	0.403	Imputed
rs11578591	1	22,558,081	A	C	0.151	0.150	0.974	1.002	0.047	0.913	1.099	0.239	Imputed
chr1:22558699:I	1	22,558,699	GA	G	0.278	0.264	0.065	1.072	0.038	0.996	1.155	0.447	Imputed
rs16826809	1	22,559,364	C	G	0.277	0.263	0.066	1.072	0.038	0.995	1.155	0.499	Imputed
rs7517342	1	22,559,439	T	C	0.229	0.240	0.151	0.944	0.040	0.872	1.021	0.317	Imputed
rs7517348	1	22,559,445	T	C	0.230	0.240	0.158	0.945	0.040	0.873	1.022	0.289	Imputed
rs10917182	1	22,559,647	T	C	0.150	0.150	0.942	0.997	0.047	0.908	1.094	0.225	Imputed
rs7517609	1	22,559,654	T	C	0.230	0.240	0.177	0.947	0.040	0.876	1.025	0.331	Imputed
rs12048960	1	22,559,813	C	G	0.278	0.264	0.063	1.073	0.038	0.996	1.156	0.454	Imputed
rs2807323	1	22,559,930	C	T	0.354	0.340	0.066	1.067	0.035	0.996	1.144	0.102	Imputed
rs115967141	1	22,560,444	T	C	0.022	0.025	0.322	0.893	0.114	0.714	1.117	0.553	Imputed
rs12124404	1	22,560,791	A	C	0.053	0.052	0.994	1.001	0.076	0.862	1.161	0.744	Imputed
rs35502211	1	22,561,066	T	C	0.278	0.264	0.059	1.074	0.038	0.997	1.157	0.416	Imputed
rs16826818	1	22,561,285	C	A	0.278	0.264	0.060	1.074	0.038	0.997	1.156	0.449	Imputed
rs2807324	1	22,561,733	T	C	0.038	0.036	0.470	1.066	0.089	0.896	1.268	0.157	Imputed
rs10753532	1	22,561,909	T	A	0.277	0.263	0.064	1.073	0.038	0.996	1.155	0.493	Imputed
rs112042234	1	22,562,016	T	C	0.118	0.120	0.733	0.982	0.053	0.886	1.089	0.242	Imputed
rs760919	1	22,562,603	A	G	0.276	0.262	0.071	1.071	0.038	0.994	1.153	0.522	Imputed
rs79887431	1	22,562,604	A	C	0.276	0.262	0.071	1.071	0.038	0.994	1.153	0.522	Imputed
rs2744740	1	22,563,105	T	C	0.354	0.340	0.063	1.068	0.035	0.996	1.145	0.121	Imputed
rs2807325	1	22,563,153	A	G	0.339	0.323	0.045	1.074	0.036	1.001	1.152	0.079	Imputed
rs72867082	1	22,563,234	G	T	0.117	0.119	0.655	0.977	0.053	0.881	1.083	0.199	Imputed
rs926436	1	22,563,429	G	A	0.148	0.150	0.648	0.979	0.048	0.891	1.074	0.151	Imputed

rs2807326	1	22,563,493	A	G	0.354	0.340	0.069	1.066	0.035	0.995	1.143	0.127	Imputed
rs2223483	1	22,563,981	C	A	0.354	0.340	0.065	1.068	0.035	0.996	1.144	0.122	Imputed
rs2807327	1	22,564,190	G	A	0.355	0.340	0.061	1.069	0.035	0.997	1.145	0.119	Imputed
rs2807328	1	22,564,305	C	T	0.354	0.340	0.065	1.068	0.035	0.996	1.144	0.122	Imputed
rs12128603	1	22,564,787	T	C	0.054	0.053	0.957	1.004	0.075	0.867	1.163	0.764	Genotyped
rs72867085	1	22,564,899	T	C	0.117	0.120	0.708	0.981	0.053	0.885	1.087	0.224	Imputed
rs2807329	1	22,565,060	A	G	0.077	0.077	0.925	1.006	0.064	0.888	1.139	0.122	Imputed
rs2807330	1	22,565,074	C	T	0.354	0.340	0.060	1.069	0.035	0.997	1.146	0.123	Imputed
rs72867086	1	22,565,150	A	G	0.118	0.120	0.730	0.982	0.053	0.886	1.088	0.241	Imputed
rs114575622	1	22,565,347	A	G	0.010	0.014	0.018	0.674	0.167	0.486	0.936	0.804	Imputed
rs2744741	1	22,565,371	T	C	0.408	0.393	0.059	1.067	0.034	0.997	1.142	0.228	Genotyped
rs76043706	1	22,565,719	T	C	0.016	0.012	0.072	1.279	0.138	0.976	1.676	0.071	Imputed
rs7536921	1	22,565,734	A	C	0.118	0.120	0.695	0.980	0.053	0.884	1.086	0.231	Imputed
rs2807331	1	22,565,967	T	C	0.475	0.487	0.127	0.950	0.034	0.889	1.015	0.569	Imputed
rs2870445	1	22,566,579	T	C	0.278	0.264	0.068	1.071	0.038	0.995	1.154	0.466	Imputed
rs141985353	1	22,566,765	T	G	0.013	0.011	0.151	1.238	0.149	0.924	1.659	0.766	Imputed
rs2807332	1	22,566,847	T	C	0.079	0.080	0.927	1.006	0.062	0.890	1.137	0.246	Imputed
rs4655034	1	22,566,919	A	T	0.230	0.240	0.176	0.947	0.040	0.876	1.025	0.294	Imputed
rs2744742	1	22,566,927	A	T	0.077	0.077	0.939	1.005	0.063	0.887	1.138	0.117	Imputed
rs148169495	1	22,567,044	A	G	0.020	0.018	0.303	1.133	0.121	0.893	1.438	0.725	Imputed
rs76834423	1	22,567,168	G	A	0.053	0.052	0.942	0.995	0.076	0.857	1.154	0.710	Imputed
rs2807333	1	22,567,381	T	G	0.131	0.130	0.872	1.008	0.050	0.914	1.112	0.292	Imputed
rs2744743	1	22,567,471	C	T	0.505	0.490	0.102	1.057	0.034	0.989	1.129	0.446	Imputed
rs12030840	1	22,567,886	A	C	0.278	0.263	0.057	1.075	0.038	0.998	1.158	0.399	Imputed
rs2807334	1	22,568,696	C	G	0.077	0.077	0.945	1.004	0.063	0.887	1.137	0.117	Imputed
rs72867093	1	22,568,709	T	C	0.116	0.119	0.658	0.977	0.053	0.881	1.083	0.180	Imputed
rs2744744	1	22,568,747	T	C	0.363	0.367	0.557	0.980	0.035	0.914	1.049	0.503	Genotyped
rs932370	1	22,569,036	A	G	0.277	0.263	0.063	1.073	0.038	0.996	1.156	0.424	Imputed
rs932371	1	22,569,226	T	C	0.277	0.263	0.061	1.073	0.038	0.997	1.156	0.409	Genotyped
rs10917184	1	22,569,504	C	G	0.278	0.263	0.057	1.075	0.038	0.998	1.158	0.399	Imputed
rs142021785	1	22,569,656	T	G	0.016	0.023	2.63E-03	0.672	0.133	0.517	0.872	0.607	Imputed
chr1:22569814:D	1	22,569,814	T	TA	0.229	0.240	0.152	0.944	0.040	0.873	1.021	0.309	Imputed
rs72867095	1	22,569,847	A	G	0.117	0.119	0.723	0.981	0.053	0.885	1.088	0.207	Imputed
chr1:22569919:I	1	22,569,919	C	CA	0.109	0.114	0.278	0.943	0.054	0.848	1.049	0.556	Imputed
chr1:22569937:D	1	22,569,937	A	AG	0.458	0.448	0.162	1.049	0.034	0.981	1.122	0.917	Imputed
rs77946829	1	22,570,121	A	G	0.025	0.025	0.684	0.956	0.110	0.771	1.185	0.075	Imputed
rs72867099	1	22,570,364	A	G	0.117	0.118	0.757	0.984	0.053	0.887	1.091	0.196	Imputed
rs61254978	1	22,570,725	A	G	0.052	0.052	0.835	0.984	0.076	0.848	1.142	0.785	Imputed
rs2744746	1	22,571,151	T	A	0.389	0.397	0.417	0.972	0.035	0.908	1.041	0.451	Imputed
rs17360394	1	22,571,211	C	A	0.229	0.240	0.166	0.946	0.040	0.874	1.023	0.367	Imputed

rs10917185	1	22,571,385	T	C	0.331	0.316	0.069	1.068	0.036	0.995	1.146	0.534	Imputed
rs16826833	1	22,571,426	A	T	0.278	0.263	0.050	1.077	0.038	1.000	1.160	0.464	Imputed
rs2744747	1	22,571,462	T	C	0.246	0.249	0.630	0.981	0.039	0.909	1.060	0.814	Imputed
rs35107757	1	22,571,481	T	C	0.277	0.262	0.039	1.081	0.038	1.004	1.164	0.432	Imputed
rs139686547	1	22,571,485	T	C	0.013	0.011	0.187	1.217	0.149	0.909	1.631	0.745	Imputed
rs7553227	1	22,572,123	G	A	0.400	0.411	0.165	0.953	0.035	0.891	1.020	0.085	Genotyped
rs12757104	1	22,572,180	A	C	0.278	0.263	0.046	1.078	0.038	1.001	1.161	0.453	Imputed
rs12755933	1	22,572,185	C	G	0.278	0.263	0.046	1.079	0.038	1.001	1.162	0.452	Imputed
rs72868607	1	22,572,235	T	C	0.117	0.118	0.798	0.987	0.053	0.890	1.094	0.165	Imputed
rs79052526	1	22,572,707	A	G	0.020	0.019	0.865	1.021	0.123	0.803	1.298	0.421	Imputed
rs10917186	1	22,573,147	A	C	0.280	0.264	0.035	1.083	0.038	1.006	1.166	0.353	Imputed
rs2744748	1	22,573,163	G	A	0.075	0.076	0.883	0.991	0.064	0.874	1.123	0.206	Imputed
rs2744749	1	22,573,226	T	A	0.407	0.392	0.075	1.063	0.034	0.994	1.137	0.168	Imputed
rs10489160	1	22,573,259	A	G	0.228	0.239	0.148	0.943	0.040	0.872	1.021	0.317	Genotyped
rs2807335	1	22,573,764	T	C	0.075	0.076	0.907	0.993	0.064	0.876	1.125	0.160	Imputed
rs909814	1	22,573,942	T	C	0.377	0.382	0.483	0.976	0.035	0.911	1.045	0.607	Imputed
rs55642049	1	22,574,175	A	G	0.097	0.097	0.881	0.992	0.057	0.886	1.109	0.218	Imputed
chr1:22574332:D	1	22,574,332	C	CT	0.268	0.261	0.343	1.037	0.038	0.962	1.118	0.459	Imputed
chr1:22574334:D	1	22,574,334	T	TC	0.391	0.376	0.065	1.066	0.035	0.996	1.141	0.168	Imputed
rs2744750	1	22,574,426	A	G	0.393	0.377	0.057	1.068	0.035	0.998	1.143	0.182	Genotyped
rs2744751	1	22,574,682	A	C	0.394	0.379	0.073	1.064	0.035	0.994	1.139	0.141	Imputed
rs143125204	1	22,575,176	T	C	0.010	0.013	0.139	0.784	0.165	0.568	1.082	0.936	Imputed
rs2744752	1	22,575,306	A	G	0.079	0.079	0.992	0.999	0.063	0.884	1.130	0.222	Genotyped
rs875977	1	22,575,918	T	C	0.260	0.245	0.039	1.083	0.039	1.004	1.168	0.401	Imputed
rs875975	1	22,575,940	G	C	0.227	0.238	0.129	0.941	0.040	0.869	1.018	0.480	Imputed
rs2744753	1	22,576,327	C	G	0.077	0.075	0.638	1.030	0.063	0.910	1.167	0.179	Imputed
rs875976	1	22,576,398	G	C	0.389	0.370	0.018	1.086	0.035	1.014	1.162	0.060	Imputed
rs2744754	1	22,576,467	A	T	0.077	0.075	0.588	1.035	0.063	0.914	1.172	0.168	Imputed
rs2744756	1	22,577,197	T	C	0.370	0.375	0.446	0.974	0.035	0.909	1.043	0.319	Imputed
rs12563325	1	22,577,246	A	G	0.049	0.049	0.775	0.978	0.079	0.838	1.141	0.913	Imputed
rs12239548	1	22,577,290	T	C	0.228	0.239	0.123	0.940	0.040	0.868	1.017	0.371	Genotyped
rs2807337	1	22,577,371	T	C	0.370	0.376	0.444	0.974	0.035	0.909	1.043	0.300	Imputed
rs2807338	1	22,577,425	T	C	0.370	0.375	0.444	0.974	0.035	0.909	1.043	0.365	Genotyped
rs75042835	1	22,577,449	G	A	0.023	0.026	0.396	0.910	0.111	0.732	1.132	0.406	Imputed
rs79008139	1	22,577,620	G	A	0.271	0.258	0.063	1.073	0.038	0.996	1.157	0.266	Imputed
rs115558877	1	22,577,709	G	A	0.012	0.013	0.885	0.978	0.152	0.726	1.318	0.611	Imputed
rs2744757	1	22,577,937	C	G	0.268	0.270	0.664	0.984	0.038	0.913	1.060	0.929	Imputed
rs2807339	1	22,578,063	T	C	0.253	0.254	0.820	0.991	0.039	0.918	1.070	0.756	Genotyped
rs76532116	1	22,578,224	A	C	0.016	0.024	2.02E-03	0.669	0.131	0.518	0.865	0.774	Imputed
rs112792715	1	22,578,404	A	G	0.130	0.136	0.388	0.958	0.050	0.868	1.057	0.080	Imputed

rs2143101	1	22,578,436	A	G	0.238	0.245	0.288	0.959	0.040	0.887	1.036	0.547	Imputed
chr1:22578580:D	1	22,578,580	C	CA	0.268	0.250	5.24E-03	1.113	0.038	1.032	1.200	0.012	Imputed
rs2744758	1	22,578,619	A	G	0.076	0.074	0.564	1.038	0.064	0.915	1.176	0.148	Imputed
rs10917188	1	22,579,006	T	C	0.237	0.245	0.257	0.956	0.040	0.884	1.033	0.521	Imputed
rs10799741	1	22,579,114	T	C	0.237	0.245	0.247	0.955	0.040	0.883	1.032	0.562	Imputed
rs74355262	1	22,579,159	A	G	0.051	0.057	0.122	0.889	0.076	0.765	1.032	0.445	Imputed
rs115087026	1	22,579,415	T	C	0.013	0.014	0.440	0.890	0.151	0.663	1.196	0.756	Imputed
rs7547619	1	22,579,630	T	C	0.237	0.245	0.270	0.957	0.040	0.885	1.035	0.582	Imputed
rs2744759	1	22,579,798	G	T	0.446	0.438	0.327	1.034	0.034	0.967	1.105	0.289	Genotyped
rs79679347	1	22,579,802	C	T	0.051	0.057	0.122	0.889	0.076	0.765	1.032	0.445	Imputed
rs185441988	1	22,579,916	G	A	0.011	0.013	0.525	0.903	0.160	0.660	1.236	0.828	Imputed
rs2807340	1	22,580,473	A	G	0.076	0.074	0.574	1.037	0.064	0.914	1.175	0.140	Imputed
rs2744760	1	22,580,752	C	G	0.363	0.363	0.969	1.001	0.035	0.935	1.073	0.582	Imputed
rs111501233	1	22,580,798	T	G	0.131	0.136	0.438	0.962	0.050	0.872	1.061	0.089	Imputed
rs12739469	1	22,581,573	T	C	0.270	0.258	0.099	1.065	0.038	0.988	1.148	0.413	Imputed
rs79398707	1	22,581,580	A	G	0.056	0.062	0.122	0.894	0.073	0.775	1.031	0.889	Imputed
rs16826854	1	22,581,646	A	G	0.236	0.245	0.227	0.953	0.040	0.882	1.030	0.542	Imputed
chr1:22581910:I	1	22,581,910	GA	G	0.328	0.329	0.708	0.987	0.036	0.919	1.059	0.443	Imputed
rs2807342	1	22,581,961	A	G	0.186	0.181	0.495	1.030	0.044	0.946	1.122	0.422	Imputed
rs143574067	1	22,583,049	G	C	0.016	0.016	0.946	1.009	0.134	0.777	1.311	0.729	Imputed
rs10159108	1	22,583,066	A	G	0.101	0.106	0.223	0.934	0.056	0.836	1.042	0.379	Imputed
rs2744697	1	22,583,655	A	G	0.076	0.074	0.584	1.036	0.064	0.914	1.174	0.142	Imputed
rs75965181	1	22,584,002	A	T	0.016	0.016	0.994	1.001	0.135	0.769	1.303	0.622	Imputed
rs141542432	1	22,584,048	T	C	0.014	0.018	0.124	0.805	0.141	0.611	1.061	0.135	Imputed
rs12137525	1	22,584,118	T	C	0.101	0.106	0.221	0.933	0.056	0.836	1.042	0.377	Imputed
rs10753533	1	22,584,165	T	C	0.468	0.485	0.029	0.929	0.034	0.869	0.993	0.025	Imputed
rs115024239	1	22,584,175	T	C	0.028	0.029	0.870	0.984	0.101	0.806	1.200	0.219	Imputed
rs113445070	1	22,584,656	T	G	0.127	0.131	0.516	0.968	0.051	0.876	1.069	0.069	Imputed
rs116357658	1	22,584,685	A	G	0.015	0.015	0.909	0.984	0.138	0.751	1.291	0.534	Imputed
rs72647481	1	22,584,718	T	C	0.076	0.074	0.584	1.036	0.064	0.914	1.174	0.142	Imputed
rs3856182	1	22,585,139	A	G	0.269	0.258	0.150	1.057	0.038	0.980	1.139	0.430	Imputed
rs3845519	1	22,585,236	A	G	0.344	0.332	0.106	1.059	0.036	0.988	1.136	0.131	Genotyped
rs76843148	1	22,585,468	A	G	0.052	0.057	0.131	0.891	0.076	0.768	1.035	0.377	Imputed
rs142386770	1	22,585,889	T	C	0.015	0.015	0.909	0.984	0.138	0.751	1.291	0.534	Imputed
rs60293366	1	22,586,202	A	G	0.101	0.108	0.136	0.920	0.056	0.824	1.027	0.376	Imputed
rs28617726	1	22,586,280	A	G	0.076	0.074	0.563	1.038	0.064	0.915	1.176	0.148	Imputed
rs6682646	1	22,586,432	A	G	0.187	0.182	0.477	1.031	0.043	0.947	1.123	0.334	Imputed
chr1:22586862:D	1	22,586,862	C	CCCTCCCTCCCTCCCTT	0.063	0.068	0.226	0.919	0.069	0.802	1.053	0.172	Imputed
rs144734989	1	22,586,878	C	T	0.046	0.048	0.375	0.931	0.081	0.794	1.091	0.440	Imputed
rs72647483	1	22,587,009	C	T	0.076	0.074	0.583	1.036	0.064	0.914	1.174	0.152	Imputed

rs115906406	1	22,587,475	T	G	0.030	0.021	1.56E-04	1.466	0.102	1.200	1.790	0.331	Imputed
rs35550345	1	22,587,493	C	A	0.271	0.260	0.124	1.060	0.038	0.984	1.143	0.443	Imputed
rs148399402	1	22,587,626	A	G	0.014	0.014	0.968	0.994	0.146	0.747	1.322	0.661	Imputed
rs72647484	1	22,587,728	C	T	0.093	0.091	0.489	1.041	0.058	0.929	1.167	0.683	Imputed
rs4655036	1	22,587,878	A	G	0.489	0.487	0.774	1.010	0.034	0.945	1.079	0.550	Genotyped
rs2473236	1	22,587,949	C	T	0.224	0.223	0.744	1.013	0.041	0.936	1.097	0.570	Imputed
rs4654790	1	22,588,355	A	G	0.310	0.318	0.429	0.972	0.037	0.904	1.044	0.835	Genotyped
rs2473237	1	22,588,993	T	C	0.205	0.200	0.575	1.024	0.042	0.943	1.112	0.296	Genotyped
rs10917192	1	22,589,020	G	C	0.218	0.228	0.186	0.947	0.041	0.874	1.026	0.673	Imputed
rs140863272	1	22,589,068	A	G	0.019	0.019	0.865	0.979	0.123	0.769	1.247	0.249	Imputed
rs116386356	1	22,589,124	C	T	0.056	0.064	0.062	0.872	0.073	0.756	1.006	0.034	Imputed
rs12752183	1	22,589,136	C	G	0.218	0.228	0.183	0.947	0.041	0.874	1.026	0.742	Imputed
rs72647487	1	22,589,836	C	T	0.244	0.244	0.969	1.002	0.039	0.927	1.082	0.686	Imputed
rs72647488	1	22,590,009	A	G	0.082	0.082	0.849	1.012	0.062	0.897	1.142	0.996	Imputed
rs72647489	1	22,590,125	C	T	0.083	0.082	0.775	1.018	0.062	0.902	1.148	0.848	Imputed
rs2473238	1	22,590,331	C	G	0.309	0.320	0.155	0.949	0.037	0.884	1.020	0.050	Imputed
rs2473239	1	22,590,487	T	C	0.191	0.190	0.901	1.005	0.043	0.924	1.094	0.085	Imputed
chr1:22590525:D	1	22,590,525	T	TG	0.010	0.011	0.707	0.939	0.167	0.677	1.304	0.101	Imputed
rs78682722	1	22,590,933	T	C	0.071	0.075	0.267	0.929	0.066	0.817	1.057	0.004	Imputed
rs75897478	1	22,591,299	A	G	0.116	0.124	0.198	0.935	0.053	0.843	1.036	0.906	Imputed
rs2505714	1	22,591,456	G	A	0.241	0.241	0.988	0.999	0.040	0.925	1.080	0.922	Genotyped
rs2505715	1	22,591,814	T	G	0.248	0.248	0.929	1.004	0.039	0.929	1.084	0.688	Imputed
chr1:22591869:D	1	22,591,869	T	TGTGC	0.288	0.275	0.091	1.065	0.037	0.990	1.146	0.367	Imputed
rs34824550	1	22,592,191	A	C	0.243	0.232	0.112	1.065	0.040	0.985	1.151	0.514	Imputed
rs115549715	1	22,592,304	A	C	0.032	0.022	3.58E-05	1.497	0.098	1.234	1.815	0.596	Imputed
rs76727629	1	22,592,949	G	A	0.068	0.069	0.684	0.973	0.067	0.853	1.110	0.005	Imputed
rs79167464	1	22,593,270	A	G	0.122	0.129	0.258	0.943	0.052	0.853	1.044	0.852	Imputed
rs4655037	1	22,593,275	C	T	0.209	0.217	0.325	0.960	0.042	0.885	1.041	0.774	Imputed
rs736473	1	22,593,385	T	C	0.498	0.489	0.265	1.038	0.034	0.972	1.110	0.388	Genotyped
rs115731004	1	22,593,654	T	G	0.014	0.017	0.139	0.813	0.140	0.618	1.070	0.437	Imputed
rs140353641	1	22,593,805	A	C	0.013	0.013	0.825	1.034	0.149	0.772	1.384	0.992	Imputed
rs78890520	1	22,594,027	T	C	0.018	0.020	0.316	0.879	0.128	0.685	1.129	0.115	Imputed
rs114666561	1	22,594,084	C	T	0.092	0.095	0.494	0.961	0.058	0.857	1.077	0.448	Imputed
chr1:22594126:I	1	22,594,126	TGC	T	0.121	0.127	0.345	0.952	0.052	0.861	1.054	0.924	Imputed
rs2505716	1	22,594,675	T	C	0.256	0.259	0.670	0.984	0.039	0.912	1.061	0.937	Imputed
rs75868741	1	22,594,676	A	G	0.130	0.136	0.360	0.955	0.050	0.866	1.054	0.898	Imputed
rs12741884	1	22,594,695	A	G	0.241	0.232	0.170	1.056	0.040	0.977	1.141	0.458	Genotyped
rs6682404	1	22,594,956	T	C	0.115	0.120	0.387	0.955	0.053	0.861	1.060	0.819	Genotyped
rs962770	1	22,595,026	G	A	0.342	0.345	0.685	0.986	0.036	0.919	1.057	0.228	Genotyped
rs76457762	1	22,595,111	T	C	0.017	0.020	0.293	0.874	0.128	0.680	1.124	0.480	Imputed

rs2505717	1	22,595,199	A	G	0.332	0.327	0.648	1.017	0.036	0.947	1.091	0.189	Imputed
rs2473240	1	22,595,303	T	A	0.348	0.342	0.523	1.023	0.036	0.954	1.097	0.045	Imputed
chr1:22595445:D	1	22,595,445	GATTGTGGTC	G	0.388	0.395	0.385	0.970	0.035	0.907	1.039	0.262	Imputed
rs112699915	1	22,595,798	T	C	0.089	0.099	0.051	0.892	0.059	0.794	1.001	0.656	Imputed
rs111828678	1	22,595,876	A	G	0.088	0.098	0.042	0.887	0.059	0.790	0.996	0.755	Imputed
rs2505718	1	22,595,969	C	A	0.447	0.433	0.077	1.062	0.034	0.994	1.135	0.527	Imputed
rs60882330	1	22,596,533	C	T	0.289	0.298	0.279	0.960	0.037	0.893	1.033	0.345	Imputed
rs2473241	1	22,596,600	T	C	0.155	0.136	4.39E-04	1.179	0.047	1.076	1.293	0.157	Genotyped
rs183806152	1	22,597,053	C	T	0.010	0.011	0.667	0.930	0.167	0.671	1.290	0.142	Imputed
rs186847900	1	22,597,073	G	A	0.012	0.012	0.925	0.986	0.155	0.728	1.334	0.777	Imputed
rs113510702	1	22,597,087	T	C	0.089	0.099	0.057	0.894	0.059	0.796	1.004	0.679	Imputed
rs4655039	1	22,597,746	T	A	0.198	0.198	0.967	1.002	0.042	0.922	1.089	0.161	Imputed
rs79716104	1	22,597,763	G	A	0.089	0.099	0.054	0.893	0.059	0.795	1.002	0.688	Imputed
rs2473242	1	22,598,057	G	A	0.152	0.132	2.03E-04	1.192	0.047	1.086	1.308	0.138	Imputed
rs80236058	1	22,598,229	A	G	0.009	0.011	0.377	0.857	0.175	0.608	1.209	0.244	Imputed
rs79899939	1	22,598,429	A	G	0.090	0.100	0.052	0.892	0.059	0.795	1.001	0.692	Imputed
rs74319208	1	22,598,480	A	C	0.089	0.099	0.057	0.894	0.059	0.796	1.003	0.681	Imputed
rs6681709	1	22,598,534	A	G	0.198	0.198	0.994	1.000	0.043	0.920	1.086	0.159	Genotyped
chr1:22598573:I	1	22,598,573	CTCAT	C	0.198	0.198	0.970	1.002	0.042	0.922	1.089	0.160	Imputed
chr1:22598755:D	1	22,598,755	C	CACTT	0.237	0.241	0.527	0.975	0.040	0.902	1.054	0.033	Imputed
chr1:22598819:I	1	22,598,819	AC	A	0.089	0.099	0.059	0.895	0.059	0.797	1.004	0.674	Imputed
rs113634627	1	22,598,903	C	A	0.089	0.099	0.057	0.894	0.059	0.796	1.003	0.681	Imputed
rs113660054	1	22,599,198	G	A	0.089	0.099	0.063	0.896	0.059	0.798	1.006	0.746	Imputed
rs2473243	1	22,599,301	A	G	0.336	0.333	0.713	1.013	0.036	0.945	1.087	0.914	Imputed
rs2473244	1	22,599,404	C	G	0.336	0.333	0.714	1.013	0.036	0.945	1.087	0.913	Imputed
rs78389094	1	22,599,444	A	G	0.089	0.099	0.064	0.897	0.059	0.799	1.006	0.744	Imputed
rs76527629	1	22,599,503	A	C	0.089	0.099	0.064	0.897	0.059	0.799	1.006	0.744	Imputed
rs78812443	1	22,599,715	A	G	0.090	0.099	0.070	0.899	0.059	0.801	1.009	0.725	Imputed
rs2143100	1	22,599,878	C	T	0.328	0.341	0.088	0.940	0.036	0.876	1.009	0.097	Genotyped
rs78848376	1	22,599,947	C	T	0.089	0.099	0.064	0.897	0.059	0.799	1.007	0.742	Imputed
rs150992064	1	22,600,027	A	C	0.012	0.015	0.202	0.824	0.152	0.611	1.111	0.395	Imputed
rs2179376	1	22,600,073	C	T	0.328	0.341	0.083	0.939	0.036	0.875	1.008	0.094	Imputed
rs1018393	1	22,600,167	T	G	0.277	0.279	0.870	0.994	0.038	0.923	1.070	0.439	Imputed
rs79344400	1	22,600,462	C	T	0.090	0.099	0.077	0.901	0.059	0.803	1.011	0.751	Imputed
rs76328390	1	22,600,470	T	C	0.090	0.099	0.077	0.901	0.059	0.803	1.011	0.751	Imputed
rs76813209	1	22,600,661	A	C	0.090	0.099	0.077	0.901	0.059	0.803	1.011	0.751	Imputed
rs4654792	1	22,600,672	C	G	0.237	0.242	0.448	0.970	0.040	0.898	1.049	0.042	Imputed
rs79443958	1	22,600,822	T	G	0.090	0.099	0.077	0.901	0.059	0.803	1.011	0.751	Imputed
rs77585595	1	22,600,876	G	A	0.090	0.100	0.056	0.894	0.059	0.797	1.003	0.601	Imputed
rs74826842	1	22,601,010	G	A	0.090	0.099	0.075	0.901	0.059	0.803	1.011	0.732	Imputed

rs34063726	1	22,601,011	G	A	0.328	0.341	0.083	0.939	0.036	0.875	1.008	0.094	Imputed
rs78140413	1	22,601,171	A	C	0.089	0.099	0.072	0.899	0.059	0.801	1.010	0.714	Imputed
rs34451690	1	22,601,580	T	A	0.328	0.341	0.079	0.939	0.036	0.875	1.007	0.079	Imputed
rs12406679	1	22,602,254	A	G	0.179	0.191	0.074	0.925	0.044	0.848	1.008	0.537	Genotyped
chr1:22602413:D	1	22,602,413	G	GGA	0.090	0.099	0.074	0.900	0.059	0.802	1.010	0.737	Imputed
rs149016001	1	22,602,455	T	C	0.012	0.015	0.202	0.824	0.152	0.611	1.111	0.395	Imputed
rs66593554	1	22,602,556	A	G	0.238	0.241	0.518	0.975	0.040	0.902	1.054	0.033	Imputed
rs909812	1	22,603,366	T	C	0.237	0.241	0.515	0.974	0.040	0.901	1.053	0.020	Imputed
rs760915	1	22,603,552	A	G	0.335	0.332	0.733	1.012	0.036	0.944	1.086	0.941	Genotyped
chr1:22603605:D	1	22,603,605	A	AC	0.093	0.102	0.090	0.907	0.058	0.809	1.016	0.876	Imputed
rs1076678	1	22,603,661	T	C	0.237	0.241	0.474	0.972	0.040	0.899	1.051	0.032	Imputed
rs79175541	1	22,603,668	A	T	0.091	0.100	0.097	0.908	0.059	0.809	1.018	0.642	Imputed
rs12045854	1	22,603,801	G	C	0.179	0.190	0.109	0.932	0.044	0.855	1.016	0.477	Imputed
rs76192896	1	22,604,928	A	G	0.059	0.055	0.325	1.074	0.072	0.932	1.238	0.125	Imputed
rs35324933	1	22,605,024	A	G	0.237	0.241	0.514	0.974	0.040	0.901	1.053	0.029	Imputed
rs76152957	1	22,605,050	A	C	0.091	0.100	0.085	0.904	0.059	0.806	1.014	0.751	Imputed
rs58231124	1	22,605,547	A	G	0.238	0.242	0.538	0.976	0.040	0.903	1.055	0.026	Imputed
rs35616249	1	22,605,641	C	G	0.237	0.242	0.482	0.972	0.040	0.900	1.051	0.035	Imputed
rs114956039	1	22,605,693	A	G	0.022	0.024	0.341	0.896	0.115	0.715	1.123	0.904	Imputed
rs80052895	1	22,605,694	T	G	0.091	0.099	0.110	0.911	0.059	0.812	1.022	0.659	Imputed
rs189229641	1	22,606,421	G	A	0.082	0.091	0.079	0.898	0.061	0.797	1.013	0.648	Imputed
rs116326800	1	22,606,708	T	C	0.023	0.021	0.509	1.079	0.115	0.861	1.353	0.060	Imputed
rs145413709	1	22,606,786	C	T	0.017	0.015	0.380	1.124	0.132	0.867	1.455	0.120	Imputed
rs2505719	1	22,607,109	T	C	0.477	0.467	0.242	1.040	0.034	0.974	1.112	0.992	Imputed
rs190382268	1	22,607,208	T	G	0.012	0.012	0.701	1.060	0.153	0.786	1.431	0.436	Imputed
chr1:22607212:I	1	22,607,212	TGAA	T	0.099	0.109	0.066	0.902	0.056	0.807	1.007	0.513	Imputed
rs140627583	1	22,607,368	T	A	0.012	0.014	0.537	0.910	0.153	0.675	1.227	0.262	Imputed
rs12753092	1	22,607,557	C	T	0.178	0.189	0.108	0.932	0.044	0.855	1.016	0.496	Genotyped
rs2473245	1	22,607,762	A	G	0.159	0.138	1.46E-04	1.193	0.047	1.089	1.307	0.186	Imputed
chr1:22607874:D	1	22,607,874	A	AG	0.262	0.252	0.131	1.060	0.039	0.983	1.143	0.231	Imputed
rs2860379	1	22,607,875	A	G	0.270	0.257	0.052	1.077	0.038	0.999	1.161	0.101	Imputed
rs61766934	1	22,608,214	A	G	0.178	0.188	0.090	0.928	0.044	0.851	1.012	0.526	Imputed
rs76871355	1	22,608,378	A	G	0.083	0.091	0.045	0.884	0.061	0.784	0.997	0.029	Imputed
rs79962271	1	22,608,535	G	A	0.093	0.101	0.122	0.914	0.058	0.816	1.024	0.575	Imputed
rs114271979	1	22,608,608	T	C	0.031	0.026	0.087	1.182	0.098	0.975	1.434	0.176	Imputed
rs77449406	1	22,609,467	T	C	0.090	0.099	0.081	0.903	0.059	0.805	1.013	0.697	Imputed
rs2505721	1	22,609,534	T	C	0.159	0.138	1.68E-04	1.191	0.047	1.087	1.305	0.184	Imputed
rs80145220	1	22,609,643	C	T	0.092	0.100	0.099	0.908	0.058	0.810	1.018	0.596	Imputed
rs151142850	1	22,610,141	T	C	0.009	0.012	0.039	0.690	0.180	0.485	0.982	0.295	Imputed
rs80143516	1	22,610,835	T	G	0.479	0.498	0.020	0.924	0.034	0.865	0.988	0.545	Imputed

rs77691009	1	22,610,839	T	G	0.409	0.424	0.053	0.936	0.034	0.875	1.001	0.505	Imputed
rs148295191	1	22,610,843	T	G	0.063	0.068	0.199	0.915	0.070	0.798	1.048	0.504	Imputed
rs76326464	1	22,610,985	T	C	0.012	0.014	0.214	0.823	0.156	0.606	1.119	0.854	Imputed
rs75753259	1	22,611,485	T	G	0.089	0.099	0.067	0.898	0.059	0.800	1.008	0.757	Imputed
rs115209034	1	22,611,787	T	C	0.013	0.014	0.616	0.927	0.151	0.689	1.247	0.182	Imputed
rs12756188	1	22,612,493	G	C	0.217	0.221	0.502	0.973	0.041	0.898	1.054	0.126	Imputed
rs112028467	1	22,612,534	G	C	0.089	0.099	0.068	0.898	0.059	0.800	1.008	0.754	Imputed
rs143642587	1	22,612,676	C	T	0.010	0.010	0.807	1.041	0.166	0.752	1.442	0.176	Imputed
rs12737805	1	22,612,690	G	A	0.216	0.221	0.437	0.969	0.041	0.894	1.050	0.125	Genotyped
rs75287406	1	22,613,570	T	A	0.089	0.098	0.077	0.901	0.059	0.803	1.011	0.775	Imputed
rs79714427	1	22,613,610	T	A	0.015	0.017	0.403	0.892	0.136	0.683	1.166	0.612	Imputed
rs78307617	1	22,613,987	T	A	0.089	0.098	0.081	0.902	0.059	0.803	1.013	0.833	Imputed
rs78573989	1	22,614,092	G	A	0.091	0.099	0.097	0.907	0.059	0.809	1.018	0.790	Imputed
rs17356374	1	22,614,646	A	G	0.204	0.207	0.578	0.977	0.042	0.900	1.061	0.266	Genotyped
rs1007243	1	22,614,839	T	C	0.292	0.294	0.803	0.991	0.037	0.921	1.066	0.526	Imputed
rs12118633	1	22,615,392	T	C	0.033	0.035	0.652	0.959	0.094	0.797	1.153	0.346	Imputed
rs76328685	1	22,615,393	A	G	0.089	0.098	0.069	0.898	0.059	0.800	1.009	0.952	Imputed
rs12734427	1	22,615,740	A	C	0.215	0.221	0.319	0.960	0.041	0.885	1.040	0.157	Genotyped
rs17360519	1	22,615,804	G	A	0.303	0.319	0.044	0.929	0.037	0.864	0.998	0.196	Imputed
chr1:22616086:D	1	22,616,086	T	TTTG	0.089	0.098	0.077	0.901	0.059	0.803	1.012	0.981	Imputed
rs760916	1	22,616,286	G	T	0.055	0.042	9.35E-05	1.340	0.075	1.156	1.554	0.174	Imputed
rs1980473	1	22,616,583	A	G	0.317	0.333	0.034	0.926	0.036	0.862	0.994	0.094	Imputed
rs880468	1	22,616,901	T	A	0.227	0.235	0.229	0.953	0.040	0.880	1.031	0.074	Imputed
rs880467	1	22,616,978	A	G	0.227	0.235	0.224	0.952	0.040	0.880	1.030	0.073	Genotyped
rs880466	1	22,617,031	A	G	0.317	0.333	0.034	0.926	0.036	0.862	0.994	0.094	Imputed
rs72649403	1	22,617,251	T	G	0.219	0.226	0.254	0.954	0.041	0.881	1.034	0.076	Imputed
rs880469	1	22,617,298	A	G	0.220	0.227	0.311	0.960	0.041	0.886	1.039	0.062	Imputed
rs72649404	1	22,617,345	T	A	0.314	0.331	0.030	0.924	0.036	0.861	0.993	0.078	Imputed
rs72868692	1	22,617,500	G	A	0.294	0.308	0.079	0.937	0.037	0.871	1.008	0.100	Imputed
rs116113465	1	22,617,536	C	T	0.292	0.311	0.013	0.911	0.037	0.847	0.980	0.456	Imputed
rs4291473	1	22,617,572	G	A	0.345	0.342	0.694	1.014	0.036	0.946	1.087	0.752	Imputed
rs79337105	1	22,617,854	C	T	0.037	0.040	0.253	0.902	0.090	0.757	1.076	0.259	Imputed
rs75812208	1	22,618,457	A	G	0.088	0.098	0.046	0.889	0.059	0.791	0.998	0.835	Imputed
rs115230645	1	22,618,736	T	C	0.062	0.060	0.782	1.020	0.071	0.888	1.171	0.075	Imputed
rs7515541	1	22,619,422	C	T	0.311	0.328	0.032	0.925	0.037	0.861	0.993	0.152	Imputed
rs7513263	1	22,619,545	T	A	0.087	0.097	0.050	0.890	0.060	0.792	1.000	0.777	Imputed
rs12059598	1	22,619,878	T	C	0.345	0.341	0.696	1.014	0.036	0.946	1.087	0.567	Imputed
rs10917195	1	22,619,946	T	C	0.164	0.142	1.05E-04	1.195	0.046	1.092	1.308	0.094	Imputed
rs71638836	1	22,619,947	A	G	0.215	0.224	0.193	0.948	0.041	0.874	1.027	0.027	Imputed
rs78895676	1	22,621,020	T	C	0.089	0.099	0.055	0.893	0.059	0.795	1.002	0.713	Imputed

rs79927124	1	22,621,071	G	C	0.061	0.061	0.911	0.992	0.071	0.864	1.140	0.100	Imputed
chr1:22621137:I	1	22,621,137	TAATG	T	0.014	0.015	0.848	0.973	0.143	0.735	1.288	0.022	Imputed
rs79425245	1	22,621,137	G	T	0.061	0.061	0.911	0.992	0.071	0.864	1.140	0.100	Imputed
rs115212331	1	22,621,151	C	T	0.012	0.012	0.823	1.036	0.156	0.763	1.406	0.587	Imputed
rs111311274	1	22,621,280	T	C	0.089	0.099	0.051	0.891	0.059	0.793	1.000	0.723	Imputed
rs67161961	1	22,621,303	C	T	0.216	0.224	0.256	0.954	0.041	0.881	1.034	0.026	Imputed
rs115905527	1	22,621,420	C	T	0.089	0.099	0.055	0.893	0.059	0.795	1.003	0.715	Imputed
rs116302964	1	22,621,421	A	G	0.089	0.099	0.055	0.893	0.059	0.795	1.003	0.715	Imputed
rs112612527	1	22,621,519	G	A	0.061	0.061	0.922	0.993	0.071	0.864	1.141	0.102	Imputed
rs12568411	1	22,622,329	A	G	0.169	0.169	0.917	0.995	0.045	0.911	1.087	0.324	Imputed
rs138014213	1	22,622,386	G	T	0.012	0.013	0.788	0.960	0.154	0.709	1.298	0.885	Imputed
rs12139039	1	22,622,437	C	A	0.112	0.103	0.111	1.090	0.054	0.980	1.212	0.157	Imputed
rs10917196	1	22,622,513	T	C	0.416	0.410	0.577	1.019	0.034	0.953	1.090	0.048	Genotyped
rs12120413	1	22,622,554	T	C	0.381	0.393	0.200	0.956	0.035	0.893	1.024	0.086	Imputed
rs35720166	1	22,622,565	T	C	0.117	0.133	6.44E-03	0.867	0.052	0.783	0.961	0.828	Imputed
chr1:22622745:I	1	22,622,745	TC	T	0.145	0.147	0.749	0.985	0.048	0.896	1.082	0.379	Imputed
rs12121212	1	22,622,756	T	C	0.236	0.246	0.186	0.949	0.040	0.878	1.026	0.234	Imputed
rs12140773	1	22,622,882	C	T	0.255	0.260	0.483	0.973	0.039	0.902	1.050	0.199	Imputed
rs12143398	1	22,623,000	A	G	0.252	0.258	0.418	0.969	0.039	0.898	1.046	0.193	Imputed
rs10799743	1	22,623,239	T	C	0.163	0.164	0.921	0.995	0.046	0.910	1.089	0.495	Imputed
rs7536060	1	22,623,248	A	G	0.126	0.118	0.142	1.078	0.051	0.975	1.192	0.167	Imputed
chr1:22623332:I	1	22,623,332	TG	T	0.163	0.163	0.943	0.997	0.046	0.911	1.090	0.434	Imputed
rs7536219	1	22,623,380	C	G	0.247	0.256	0.296	0.960	0.039	0.889	1.037	0.240	Imputed
rs72649408	1	22,623,542	T	C	0.163	0.163	0.898	0.994	0.046	0.909	1.087	0.476	Imputed
rs12043950	1	22,623,641	C	A	0.247	0.256	0.285	0.959	0.039	0.888	1.035	0.266	Imputed
rs12043958	1	22,623,651	G	A	0.247	0.256	0.285	0.959	0.039	0.888	1.035	0.266	Imputed
rs10799744	1	22,623,863	A	G	0.163	0.162	0.952	1.003	0.046	0.917	1.097	0.433	Imputed
rs10799745	1	22,623,933	A	G	0.163	0.162	0.996	1.000	0.046	0.914	1.094	0.404	Imputed
rs4457550	1	22,624,235	C	T	0.165	0.165	0.846	0.991	0.046	0.907	1.084	0.351	Imputed
chr1:22624466:I	1	22,624,466	AT	A	0.125	0.118	0.204	1.067	0.051	0.965	1.180	0.112	Imputed
rs6426743	1	22,624,511	A	G	0.163	0.163	0.949	0.997	0.046	0.912	1.091	0.455	Imputed
rs6426744	1	22,624,557	G	A	0.341	0.332	0.280	1.039	0.036	0.969	1.115	0.974	Imputed
rs145522054	1	22,624,636	T	C	0.024	0.020	0.104	1.201	0.113	0.962	1.498	0.585	Imputed
rs6675040	1	22,624,702	C	A	0.165	0.165	0.843	0.991	0.046	0.906	1.084	0.352	Imputed
rs6426745	1	22,624,756	A	G	0.165	0.165	0.843	0.991	0.046	0.906	1.084	0.352	Imputed
rs7354906	1	22,625,074	A	C	0.164	0.165	0.822	0.990	0.046	0.905	1.082	0.360	Imputed
rs10799746	1	22,625,246	C	A	0.165	0.166	0.821	0.990	0.046	0.905	1.082	0.470	Genotyped
rs10753534	1	22,625,311	G	A	0.163	0.163	0.929	0.996	0.046	0.911	1.089	0.449	Imputed
rs12143066	1	22,625,403	C	T	0.126	0.118	0.188	1.070	0.051	0.968	1.182	0.122	Imputed
chr1:22625581:I	1	22,625,581	AT	A	0.037	0.035	0.479	1.065	0.089	0.894	1.269	0.219	Imputed

rs12049211	1	22,625,643	A	G	0.163	0.163	0.964	0.998	0.046	0.912	1.092	0.412	Imputed
rs10917197	1	22,625,770	G	A	0.165	0.165	0.846	0.991	0.046	0.907	1.084	0.351	Imputed
rs12021835	1	22,625,826	T	G	0.162	0.161	0.965	1.002	0.046	0.916	1.096	0.402	Imputed
rs10917198	1	22,625,952	C	T	0.165	0.165	0.849	0.991	0.046	0.907	1.084	0.350	Imputed
rs10917199	1	22,626,271	C	G	0.126	0.118	0.186	1.070	0.051	0.968	1.183	0.128	Imputed
rs10917200	1	22,626,370	T	G	0.165	0.165	0.846	0.991	0.046	0.907	1.084	0.351	Imputed
rs12029355	1	22,626,638	T	C	0.165	0.165	0.850	0.991	0.046	0.907	1.084	0.349	Imputed
rs113619481	1	22,626,687	A	C	0.165	0.165	0.850	0.991	0.046	0.907	1.084	0.349	Imputed
rs116532258	1	22,626,765	T	C	0.163	0.162	0.983	1.001	0.046	0.915	1.095	0.334	Imputed
rs10917201	1	22,626,927	G	A	0.165	0.165	0.850	0.991	0.046	0.907	1.084	0.349	Imputed
rs10917202	1	22,627,034	A	G	0.165	0.165	0.850	0.991	0.046	0.907	1.084	0.349	Imputed
rs10917203	1	22,627,266	A	C	0.340	0.331	0.266	1.041	0.036	0.970	1.116	0.967	Imputed
rs10917204	1	22,627,313	C	A	0.165	0.165	0.878	0.993	0.046	0.908	1.086	0.340	Imputed
rs12088199	1	22,627,395	G	A	0.165	0.165	0.878	0.993	0.046	0.908	1.086	0.340	Imputed
rs12088205	1	22,627,445	C	A	0.165	0.165	0.876	0.993	0.046	0.908	1.086	0.348	Imputed
rs12088248	1	22,627,517	G	A	0.165	0.165	0.871	0.993	0.046	0.908	1.085	0.350	Imputed
rs13374200	1	22,627,760	T	C	0.164	0.165	0.816	0.990	0.046	0.905	1.082	0.326	Imputed
rs4633259	1	22,627,871	T	A	0.166	0.167	0.793	0.988	0.045	0.904	1.080	0.351	Imputed
rs7524862	1	22,628,172	T	A	0.247	0.256	0.301	0.960	0.039	0.889	1.037	0.250	Imputed
rs7513169	1	22,628,207	A	C	0.247	0.256	0.298	0.960	0.039	0.889	1.037	0.251	Imputed
chr1:22628270:I	1	22,628,270	T	TC	0.185	0.180	0.486	1.031	0.044	0.946	1.123	0.574	Imputed
rs4477249	1	22,628,301	A	T	0.164	0.164	0.946	0.997	0.046	0.912	1.090	0.437	Imputed
rs10917205	1	22,628,317	C	G	0.164	0.164	0.946	0.997	0.046	0.912	1.090	0.437	Imputed
rs72649419	1	22,628,493	G	C	0.311	0.321	0.229	0.957	0.037	0.891	1.028	0.104	Imputed
rs10917206	1	22,628,496	A	G	0.347	0.341	0.561	1.021	0.036	0.952	1.095	0.338	Imputed
rs7516084	1	22,628,954	T	C	0.247	0.256	0.295	0.960	0.039	0.889	1.036	0.245	Imputed
chr1:22629019:I	1	22,629,019	C	CT	0.183	0.177	0.424	1.036	0.044	0.950	1.129	0.687	Imputed
chr1:22629038:I	1	22,629,038	TAGAG	T	0.222	0.228	0.474	0.971	0.041	0.897	1.052	0.170	Imputed
rs6426746	1	22,629,057	A	G	0.183	0.177	0.399	1.038	0.044	0.952	1.131	0.653	Genotyped
rs7527884	1	22,629,121	T	A	0.247	0.256	0.295	0.960	0.039	0.889	1.036	0.245	Imputed
chr1:22629154:D	1	22,629,154	T	TAAG	0.164	0.164	0.967	0.998	0.046	0.913	1.092	0.500	Imputed
rs7538267	1	22,629,188	A	G	0.247	0.256	0.293	0.960	0.039	0.889	1.036	0.246	Imputed
rs12026987	1	22,629,355	G	C	0.126	0.118	0.170	1.073	0.051	0.970	1.186	0.134	Imputed
rs4654794	1	22,629,387	C	T	0.186	0.181	0.476	1.032	0.044	0.947	1.123	0.676	Imputed
rs60897542	1	22,630,645	T	A	0.305	0.316	0.185	0.952	0.037	0.886	1.024	0.119	Imputed
rs56741055	1	22,630,654	C	T	0.305	0.316	0.180	0.952	0.037	0.886	1.023	0.117	Imputed
rs76699248	1	22,631,166	C	T	0.017	0.016	0.792	1.036	0.133	0.798	1.345	0.117	Imputed
rs34121098	1	22,631,588	T	G	0.118	0.135	5.77E-03	0.866	0.052	0.782	0.959	0.831	Imputed
rs112985112	1	22,632,223	A	C	0.305	0.315	0.209	0.955	0.037	0.889	1.026	0.121	Imputed
rs10917207	1	22,632,310	T	C	0.125	0.118	0.191	1.069	0.051	0.967	1.182	0.124	Imputed

chr1:22632515:D	1	22,632,515	A	AATG	0.247	0.256	0.286	0.959	0.039	0.888	1.036	0.253	Imputed
rs67692625	1	22,632,550	G	A	0.305	0.316	0.194	0.953	0.037	0.887	1.025	0.117	Imputed
rs6684438	1	22,632,699	A	G	0.186	0.181	0.479	1.031	0.044	0.947	1.123	0.678	Imputed
rs55654754	1	22,633,113	G	A	0.337	0.327	0.238	1.043	0.036	0.972	1.119	0.959	Imputed
rs56020422	1	22,634,131	C	T	0.170	0.163	0.241	1.054	0.045	0.965	1.152	0.597	Imputed
rs116238047	1	22,635,450	T	C	0.012	0.012	0.991	1.002	0.155	0.740	1.356	0.614	Imputed
rs10917208	1	22,636,103	C	T	0.120	0.114	0.281	1.058	0.052	0.955	1.171	0.210	Imputed
rs4589079	1	22,636,659	T	C	0.181	0.176	0.520	1.029	0.044	0.944	1.121	0.415	Imputed
rs4589080	1	22,636,808	T	C	0.465	0.476	0.224	0.960	0.034	0.898	1.026	0.017	Genotyped
rs181287420	1	22,636,873	T	A	0.013	0.012	0.635	1.074	0.151	0.800	1.443	0.718	Imputed
rs76599916	1	22,637,589	C	G	0.066	0.067	0.944	0.995	0.068	0.871	1.137	0.609	Imputed
rs111864242	1	22,637,713	T	C	0.074	0.074	0.773	1.019	0.065	0.898	1.156	0.657	Imputed
rs4655040	1	22,638,050	G	A	0.344	0.350	0.447	0.973	0.036	0.908	1.044	0.060	Genotyped
rs111859922	1	22,638,080	A	G	0.045	0.043	0.524	1.054	0.082	0.897	1.238	0.593	Imputed
rs113634428	1	22,638,195	A	G	0.037	0.039	0.682	0.964	0.089	0.809	1.149	0.540	Imputed
rs4655041	1	22,638,819	T	C	0.225	0.222	0.707	1.015	0.041	0.938	1.099	0.265	Imputed
rs7531369	1	22,639,161	G	C	0.331	0.335	0.582	0.980	0.036	0.914	1.052	0.128	Imputed
rs6426747	1	22,639,190	T	A	0.222	0.220	0.882	1.006	0.041	0.929	1.090	0.252	Imputed
rs16826892	1	22,639,964	T	C	0.038	0.040	0.658	0.962	0.088	0.810	1.143	0.595	Imputed
rs4598465	1	22,640,192	C	T	0.311	0.303	0.282	1.040	0.037	0.968	1.118	0.843	Imputed
rs111753426	1	22,640,970	A	G	0.038	0.040	0.640	0.960	0.088	0.808	1.140	0.771	Imputed
rs113124992	1	22,640,977	A	G	0.038	0.040	0.640	0.960	0.088	0.808	1.140	0.771	Imputed
rs113785832	1	22,641,007	T	C	0.038	0.040	0.640	0.960	0.088	0.808	1.140	0.771	Imputed
rs74816778	1	22,641,134	A	G	0.030	0.031	0.766	0.971	0.100	0.799	1.180	0.470	Imputed
rs79986280	1	22,641,959	A	G	0.014	0.013	0.591	1.080	0.143	0.816	1.428	0.784	Imputed
rs115215555	1	22,642,044	A	C	0.011	0.011	0.876	0.975	0.162	0.709	1.340	0.614	Imputed
chr1:22642167:I	1	22,642,167	TA	T	0.038	0.040	0.574	0.952	0.088	0.801	1.131	0.732	Imputed
rs114651452	1	22,642,797	A	G	0.013	0.015	0.254	0.844	0.149	0.630	1.130	0.560	Imputed
chr1:22642909:I	1	22,642,909	A	AC	0.310	0.313	0.734	0.988	0.037	0.919	1.061	0.036	Imputed
rs116724501	1	22,644,991	T	C	0.013	0.012	0.677	1.065	0.152	0.791	1.434	0.413	Imputed
rs79773108	1	22,645,147	T	C	0.083	0.083	0.990	1.001	0.061	0.887	1.129	0.962	Imputed
rs112943651	1	22,645,195	T	C	0.075	0.075	0.986	1.001	0.064	0.883	1.136	0.864	Imputed
rs113814907	1	22,645,511	C	T	0.083	0.083	0.910	1.007	0.061	0.893	1.135	0.895	Imputed
rs8179370	1	22,645,889	A	G	0.102	0.106	0.304	0.944	0.056	0.846	1.053	0.537	Genotyped
rs148082431	1	22,646,126	A	G	0.027	0.025	0.773	1.031	0.106	0.838	1.268	0.872	Imputed
rs76767825	1	22,646,446	G	T	0.014	0.012	0.382	1.134	0.144	0.855	1.506	0.685	Imputed
rs7516288	1	22,646,788	A	G	0.392	0.393	0.956	1.002	0.035	0.936	1.072	0.699	Imputed
rs6690148	1	22,648,411	T	C	0.267	0.266	0.948	1.002	0.038	0.930	1.081	0.660	Genotyped
rs113784679	1	22,648,479	T	G	0.026	0.028	0.435	0.920	0.107	0.746	1.134	0.657	Imputed
rs72649429	1	22,649,018	A	G	0.162	0.156	0.284	1.051	0.046	0.960	1.150	0.963	Imputed

rs7546088	1	22,649,037	T	C	0.294	0.296	0.827	0.992	0.037	0.922	1.067	0.832	Imputed
rs11580218	1	22,649,487	T	G	0.109	0.111	0.814	0.987	0.054	0.888	1.098	0.783	Genotyped
rs10917209	1	22,649,994	G	A	0.270	0.267	0.682	1.016	0.038	0.943	1.095	0.540	Genotyped
rs16826920	1	22,650,546	T	C	0.070	0.071	0.838	0.987	0.066	0.866	1.123	0.818	Genotyped
rs142430518	1	22,651,090	C	A	0.013	0.012	0.711	1.057	0.149	0.789	1.416	0.945	Imputed
rs76247373	1	22,651,414	T	C	0.020	0.019	0.756	1.038	0.120	0.820	1.314	0.576	Imputed
rs10753535	1	22,651,579	C	G	0.492	0.482	0.138	1.052	0.034	0.984	1.124	0.330	Imputed
rs10917211	1	22,651,603	T	G	0.183	0.186	0.894	0.994	0.044	0.912	1.084	0.506	Imputed
rs147872811	1	22,651,604	C	G	0.040	0.038	0.382	1.078	0.086	0.911	1.277	0.777	Imputed
rs10917212	1	22,651,606	G	A	0.230	0.230	0.736	1.014	0.041	0.936	1.098	0.666	Imputed
rs10917213	1	22,651,682	C	A	0.039	0.037	0.309	1.092	0.087	0.921	1.296	0.286	Imputed
rs60329625	1	22,651,922	A	G	0.028	0.030	0.794	0.974	0.102	0.798	1.189	0.978	Imputed
rs10917214	1	22,652,501	A	G	0.471	0.469	0.905	1.004	0.034	0.940	1.073	0.503	Imputed
rs72870421	1	22,652,911	T	C	0.058	0.054	0.133	1.115	0.072	0.967	1.285	0.552	Imputed
rs7525483	1	22,652,997	G	A	0.029	0.031	0.554	0.943	0.100	0.776	1.146	0.703	Genotyped
rs7513808	1	22,653,005	T	C	0.029	0.031	0.493	0.934	0.100	0.767	1.136	0.779	Imputed
rs58438761	1	22,653,079	T	C	0.065	0.059	0.057	1.140	0.069	0.996	1.304	0.339	Imputed
rs7535940	1	22,653,158	A	G	0.029	0.031	0.493	0.934	0.100	0.767	1.136	0.779	Imputed
rs4233284	1	22,653,424	G	C	0.325	0.335	0.158	0.950	0.036	0.885	1.020	0.742	Imputed
rs4433361	1	22,653,550	T	C	0.326	0.336	0.164	0.951	0.036	0.886	1.021	0.700	Imputed
rs76225863	1	22,653,595	A	G	0.041	0.036	0.075	1.164	0.086	0.984	1.377	0.607	Imputed
rs12137132	1	22,653,884	T	C	0.326	0.336	0.160	0.951	0.036	0.886	1.020	0.773	Genotyped
rs139388853	1	22,653,992	T	C	0.026	0.023	0.375	1.100	0.107	0.891	1.356	0.979	Imputed
rs4367765	1	22,654,111	A	G	0.360	0.373	0.085	0.941	0.035	0.878	1.008	0.893	Imputed
rs4655043	1	22,654,254	A	G	0.383	0.387	0.590	0.981	0.035	0.917	1.051	0.896	Genotyped
rs4654795	1	22,655,630	A	G	0.324	0.335	0.148	0.949	0.036	0.884	1.019	0.713	Imputed
rs6668462	1	22,655,757	T	C	0.029	0.031	0.452	0.927	0.101	0.761	1.129	0.846	Imputed
rs111745895	1	22,656,725	T	C	0.058	0.053	0.130	1.116	0.073	0.968	1.287	0.695	Imputed
rs12082601	1	22,656,796	T	C	0.288	0.274	0.040	1.080	0.037	1.003	1.162	0.984	Imputed
rs11580249	1	22,656,863	T	G	0.165	0.163	0.802	1.011	0.046	0.925	1.106	0.707	Genotyped
rs11585537	1	22,656,868	G	C	0.166	0.162	0.614	1.023	0.046	0.936	1.119	0.541	Imputed
rs78042793	1	22,656,936	T	C	0.029	0.031	0.433	0.924	0.101	0.759	1.126	0.821	Imputed
rs75940334	1	22,657,091	A	G	0.029	0.031	0.416	0.921	0.101	0.756	1.122	0.833	Imputed
rs35274107	1	22,657,120	T	C	0.363	0.379	0.035	0.928	0.035	0.867	0.995	0.253	Imputed
rs78252507	1	22,657,679	G	A	0.121	0.117	0.488	1.037	0.052	0.936	1.148	0.134	Imputed
rs143172994	1	22,657,777	A	G	0.016	0.016	0.868	0.978	0.135	0.750	1.275	0.492	Imputed
rs12128312	1	22,658,301	A	G	0.111	0.115	0.412	0.957	0.054	0.861	1.063	0.888	Genotyped
rs12128356	1	22,658,485	A	G	0.365	0.381	0.038	0.930	0.035	0.868	0.996	0.287	Imputed
rs113589630	1	22,658,682	T	C	0.029	0.031	0.410	0.920	0.101	0.756	1.121	0.800	Imputed
rs116126380	1	22,659,203	A	T	0.047	0.040	0.020	1.207	0.081	1.030	1.415	0.334	Imputed

rs6660965	1	22,659,243	T	C	0.029	0.031	0.455	0.928	0.100	0.762	1.129	0.767	Imputed
rs4655044	1	22,659,375	C	T	0.343	0.340	0.857	1.006	0.036	0.939	1.079	0.364	Genotyped
rs6676262	1	22,659,397	A	T	0.029	0.031	0.437	0.925	0.100	0.760	1.126	0.779	Imputed
rs12034809	1	22,660,414	T	G	0.190	0.180	0.090	1.076	0.043	0.989	1.171	0.562	Imputed
rs113246313	1	22,662,887	C	T	0.028	0.031	0.367	0.913	0.101	0.749	1.113	0.834	Imputed
rs114118053	1	22,663,720	A	G	0.048	0.053	0.130	0.887	0.079	0.760	1.036	0.323	Imputed
rs12407338	1	22,664,003	A	G	0.284	0.269	0.026	1.088	0.038	1.010	1.171	0.890	Genotyped
rs1934477	1	22,664,774	T	C	0.343	0.340	0.862	1.006	0.036	0.938	1.079	0.430	Imputed
rs114382075	1	22,664,835	A	G	0.010	0.011	0.803	0.959	0.168	0.690	1.333	0.709	Imputed
rs1934478	1	22,665,210	G	A	0.409	0.402	0.386	1.030	0.034	0.963	1.102	0.193	Imputed
rs10917216	1	22,665,400	C	A	0.412	0.404	0.317	1.035	0.034	0.968	1.107	0.140	Genotyped
rs111676509	1	22,667,791	A	G	0.053	0.051	0.337	1.075	0.076	0.927	1.247	0.962	Imputed
rs4655047	1	22,668,372	T	C	0.418	0.432	0.109	0.947	0.034	0.885	1.012	0.443	Imputed
rs4655048	1	22,668,771	T	C	0.224	0.218	0.327	1.041	0.041	0.961	1.127	0.775	Imputed
rs34663201	1	22,668,830	A	G	0.020	0.016	0.120	1.210	0.123	0.950	1.541	0.102	Imputed
rs10917217	1	22,669,472	A	C	0.446	0.437	0.311	1.035	0.034	0.968	1.107	0.409	Imputed
rs59422211	1	22,669,653	A	G	0.055	0.052	0.306	1.079	0.075	0.933	1.249	0.886	Imputed
rs7553482	1	22,669,656	G	A	0.366	0.364	0.919	1.004	0.035	0.937	1.075	0.431	Genotyped
rs34634004	1	22,670,583	A	G	0.339	0.352	0.111	0.945	0.036	0.881	1.013	0.544	Imputed
rs12078899	1	22,670,664	G	A	0.419	0.434	0.092	0.944	0.034	0.883	1.009	0.696	Genotyped
rs79483286	1	22,670,695	G	A	0.019	0.021	0.269	0.872	0.123	0.685	1.111	0.174	Imputed
rs191270678	1	22,671,455	T	C	0.027	0.024	0.212	1.140	0.105	0.928	1.400	0.672	Imputed
rs74061832	1	22,671,671	T	C	0.008	0.012	0.015	0.636	0.189	0.440	0.920	0.289	Imputed
rs12048660	1	22,672,363	G	C	0.195	0.188	0.214	1.055	0.043	0.970	1.147	0.876	Imputed
rs79953792	1	22,672,371	A	C	0.043	0.043	0.812	1.020	0.084	0.866	1.202	0.949	Imputed
rs4655050	1	22,673,644	A	G	0.384	0.396	0.148	0.951	0.035	0.888	1.018	0.582	Imputed
rs184840896	1	22,673,732	G	T	0.010	0.010	0.956	0.991	0.171	0.709	1.385	0.540	Imputed
chr1:22673891:D	1	22,673,891	A	AAAAG	0.200	0.195	0.445	1.033	0.042	0.951	1.123	0.945	Imputed
chr1:22673892:D	1	22,673,892	A	AAAG	0.220	0.212	0.255	1.048	0.041	0.967	1.135	0.705	Imputed
rs4655051	1	22,674,007	A	G	0.224	0.219	0.435	1.032	0.041	0.953	1.118	0.844	Imputed
rs149857527	1	22,674,115	A	G	0.011	0.010	0.749	1.054	0.165	0.763	1.456	0.525	Imputed
rs10917219	1	22,674,370	T	C	0.341	0.353	0.100	0.943	0.036	0.879	1.011	0.572	Genotyped
rs12137135	1	22,675,221	G	A	0.146	0.152	0.245	0.946	0.048	0.861	1.039	0.695	Imputed
chr1:22675708:D	1	22,675,708	A	AAG	0.048	0.049	0.919	1.008	0.079	0.864	1.176	0.525	Imputed
rs3856183	1	22,675,846	A	G	0.341	0.353	0.105	0.944	0.036	0.880	1.012	0.581	Imputed
rs4233285	1	22,676,292	T	C	0.384	0.376	0.433	1.028	0.035	0.960	1.100	0.576	Imputed
rs112843613	1	22,677,646	C	G	0.046	0.048	0.556	0.954	0.081	0.814	1.117	0.840	Imputed
rs17430940	1	22,677,703	C	T	0.028	0.030	0.362	0.911	0.102	0.745	1.113	0.843	Imputed
rs61769163	1	22,678,805	T	C	0.059	0.063	0.318	0.931	0.072	0.809	1.071	0.576	Imputed
rs12088685	1	22,679,088	A	G	0.021	0.026	0.043	0.789	0.117	0.627	0.993	0.341	Imputed

rs78477485	1	22,679,125	T	C	0.050	0.052	0.852	0.986	0.077	0.847	1.147	0.582	Imputed
rs1343987	1	22,679,309	T	C	0.196	0.188	0.180	1.059	0.043	0.974	1.151	0.693	Imputed
chr1:22679435:D	1	22,679,435	C	CT	0.027	0.030	0.336	0.906	0.103	0.741	1.108	0.678	Imputed
rs111980782	1	22,679,476	C	T	0.017	0.019	0.464	0.910	0.129	0.707	1.172	0.619	Imputed
rs76972837	1	22,679,970	A	G	0.010	0.012	0.413	0.870	0.169	0.626	1.211	0.035	Imputed
rs7526047	1	22,679,996	A	G	0.027	0.030	0.303	0.899	0.103	0.735	1.101	0.741	Imputed
rs6660548	1	22,680,079	G	A	0.138	0.138	0.992	1.000	0.049	0.908	1.100	0.675	Imputed
rs1316342	1	22,680,517	C	A	0.145	0.140	0.379	1.043	0.048	0.949	1.146	0.657	Genotyped
rs56232568	1	22,681,201	T	G	0.413	0.406	0.401	1.029	0.034	0.962	1.101	0.464	Imputed
rs34761529	1	22,681,214	T	C	0.200	0.201	0.986	0.999	0.042	0.920	1.086	0.883	Imputed
rs12045139	1	22,681,615	A	G	0.146	0.140	0.313	1.050	0.048	0.955	1.153	0.815	Imputed
rs11578020	1	22,682,133	T	C	0.248	0.246	0.758	1.012	0.039	0.937	1.093	0.935	Imputed
chr1:22682306:D	1	22,682,306	CT	C	0.401	0.409	0.303	0.965	0.035	0.902	1.033	0.479	Imputed
chr1:22682307:D	1	22,682,307	TTA	T	0.401	0.409	0.303	0.965	0.035	0.902	1.033	0.479	Imputed
rs12742784	1	22,682,366	T	C	0.204	0.204	0.954	0.998	0.042	0.919	1.083	0.525	Genotyped
chr1:22682541:D	1	22,682,541	G	GA	0.024	0.026	0.391	0.909	0.111	0.731	1.130	0.528	Imputed
rs56088118	1	22,682,544	T	A	0.048	0.048	0.865	0.987	0.079	0.845	1.153	0.563	Imputed
rs76917597	1	22,682,821	T	A	0.070	0.075	0.446	0.951	0.066	0.836	1.082	0.335	Imputed
rs12089677	1	22,683,030	G	A	0.249	0.246	0.685	1.016	0.039	0.941	1.097	0.918	Imputed
rs10917220	1	22,683,405	T	C	0.401	0.409	0.328	0.967	0.034	0.904	1.034	0.483	Genotyped
rs77582331	1	22,684,517	A	G	0.048	0.045	0.442	1.063	0.080	0.909	1.243	0.988	Imputed
chr1:22684576:I	1	22,684,576	AG	A	0.146	0.140	0.315	1.049	0.048	0.955	1.153	0.883	Imputed
rs12756978	1	22,684,755	C	G	0.149	0.147	0.805	1.012	0.048	0.922	1.111	0.375	Imputed
rs12097230	1	22,684,919	A	G	0.451	0.450	0.826	1.008	0.034	0.943	1.077	0.536	Imputed
rs12092590	1	22,684,927	G	A	0.247	0.245	0.802	1.010	0.039	0.935	1.091	0.821	Imputed
rs10753536	1	22,685,251	A	G	0.396	0.392	0.682	1.014	0.035	0.948	1.085	0.347	Genotyped
rs148018251	1	22,685,447	C	G	0.029	0.026	0.152	1.156	0.101	0.948	1.410	0.749	Imputed
rs76883087	1	22,686,447	C	T	0.054	0.057	0.599	0.962	0.075	0.831	1.113	0.534	Imputed
rs12029258	1	22,686,493	A	C	0.147	0.140	0.271	1.054	0.048	0.960	1.158	0.756	Imputed
rs61769165	1	22,686,594	A	G	0.262	0.264	0.817	0.991	0.038	0.919	1.069	0.728	Imputed
rs12029320	1	22,686,692	T	C	0.145	0.140	0.375	1.044	0.048	0.950	1.147	0.794	Imputed
rs113633002	1	22,686,887	C	G	0.054	0.057	0.645	0.966	0.074	0.835	1.118	0.512	Imputed
rs4439332	1	22,686,949	G	A	0.347	0.342	0.513	1.024	0.036	0.955	1.097	0.532	Imputed
rs78746042	1	22,686,987	A	G	0.273	0.274	0.964	0.998	0.038	0.926	1.076	0.911	Imputed
rs78486914	1	22,686,991	G	A	0.283	0.283	0.934	1.003	0.038	0.932	1.080	0.586	Imputed
rs111830527	1	22,687,173	A	G	0.054	0.057	0.645	0.966	0.074	0.835	1.118	0.512	Imputed
rs11810369	1	22,687,651	A	G	0.149	0.147	0.865	1.008	0.048	0.918	1.107	0.353	Imputed
rs12089212	1	22,687,701	C	T	0.408	0.404	0.596	1.018	0.034	0.952	1.090	0.687	Imputed
rs55936687	1	22,687,882	T	G	0.145	0.140	0.375	1.044	0.048	0.950	1.147	0.794	Imputed
rs55739621	1	22,688,018	G	A	0.147	0.140	0.248	1.057	0.048	0.962	1.161	0.867	Imputed

rs4655052	1	22,688,484	G	A	0.201	0.197	0.494	1.029	0.042	0.948	1.118	0.799	Genotyped
rs4233286	1	22,688,589	C	T	0.350	0.345	0.435	1.028	0.035	0.959	1.102	0.564	Imputed
rs12750721	1	22,689,273	A	G	0.149	0.147	0.849	1.009	0.048	0.919	1.108	0.373	Imputed
rs72649450	1	22,689,344	T	G	0.146	0.140	0.324	1.049	0.048	0.954	1.152	0.876	Imputed

Table S2b RND3-RMB43 region on chromosome 2

SNP	CHR	BP	Allele 1	Allele 2	F_A	F_U	P cmh test	OR cmh	SE	L95	U95	P het	Method
rs10497061	2	151,375,161	G	A	0.095	0.095	0.838	0.988	0.058	0.883	1.107	0.602	Genotyped
rs7605020	2	151,375,298	C	T	0.473	0.466	0.351	1.032	0.034	0.966	1.103	0.357	Imputed
rs72865991	2	151,375,349	C	T	0.101	0.110	0.060	0.900	0.056	0.807	1.004	0.341	Imputed
rs11681459	2	151,375,551	A	G	0.269	0.263	0.325	1.038	0.038	0.963	1.119	0.931	Genotyped
chr2:151376449:I	2	151,376,449	TTG	T	0.012	0.010	0.286	1.183	0.157	0.869	1.610	0.889	Imputed
rs1919142	2	151,376,543	C	T	0.473	0.466	0.350	1.032	0.034	0.966	1.103	0.358	Imputed
rs78538345	2	151,377,525	T	C	0.095	0.095	0.817	0.987	0.058	0.881	1.105	0.612	Imputed
rs79231981	2	151,378,204	T	C	0.066	0.065	0.758	1.021	0.069	0.893	1.168	0.043	Imputed
rs16828305	2	151,378,867	G	A	0.095	0.095	0.817	0.987	0.058	0.881	1.105	0.612	Imputed
rs139984487	2	151,379,206	A	G	0.016	0.019	0.235	0.854	0.133	0.659	1.108	0.746	Imputed
rs142322820	2	151,379,994	A	C	0.028	0.029	0.587	0.946	0.103	0.773	1.157	0.182	Imputed
rs12692747	2	151,380,108	A	C	0.475	0.471	0.518	1.022	0.034	0.957	1.092	0.407	Imputed
rs13401636	2	151,380,685	A	G	0.101	0.101	1.000	1.000	0.056	0.896	1.116	0.423	Imputed
rs2340852	2	151,380,968	A	T	0.471	0.464	0.393	1.029	0.034	0.963	1.100	0.315	Imputed
rs12692748	2	151,381,624	A	C	0.382	0.386	0.659	0.985	0.035	0.920	1.054	0.803	Imputed
rs62169697	2	151,382,047	A	C	0.045	0.051	0.109	0.879	0.081	0.750	1.029	0.510	Imputed
rs140050953	2	151,382,232	G	T	0.087	0.092	0.346	0.945	0.060	0.841	1.063	0.541	Imputed
rs114790858	2	151,382,483	A	G	0.069	0.071	0.468	0.953	0.067	0.836	1.086	0.891	Imputed
rs115566268	2	151,382,515	T	G	0.026	0.027	0.493	0.929	0.107	0.754	1.145	0.232	Imputed
rs145700377	2	151,382,950	T	G	0.024	0.025	0.389	0.909	0.111	0.731	1.130	0.931	Imputed
rs17197723	2	151,383,662	T	C	0.083	0.086	0.560	0.965	0.061	0.856	1.088	0.254	Imputed
chr2:151384929:I	2	151,384,929	TTA	T	0.146	0.143	0.601	1.026	0.048	0.933	1.127	0.151	Imputed
rs116198341	2	151,384,940	G	T	0.026	0.026	0.866	1.018	0.105	0.828	1.251	0.045	Imputed
rs112657180	2	151,384,944	T	G	0.010	0.010	0.708	0.937	0.173	0.668	1.315	0.308	Imputed
rs11898136	2	151,385,324	C	G	0.023	0.025	0.404	0.911	0.112	0.732	1.134	0.801	Imputed
chr2:151385515:I	2	151,385,515	TCC	T	0.053	0.053	0.989	1.001	0.075	0.864	1.160	0.040	Imputed
rs17269899	2	151,388,966	G	A	0.077	0.074	0.555	1.038	0.064	0.917	1.176	0.977	Imputed
chr2:151389565:D	2	151,389,565	C	CTT	0.051	0.049	0.501	1.053	0.077	0.906	1.225	0.623	Imputed
rs56114110	2	151,390,744	C	G	0.107	0.112	0.428	0.958	0.055	0.860	1.066	0.648	Imputed
rs148534875	2	151,391,305	C	T	0.093	0.088	0.319	1.060	0.058	0.945	1.189	0.465	Imputed

chr2:151391489:I	2	151,391,489	ATG	A	0.028	0.029	0.671	0.957	0.103	0.783	1.171	0.325	Imputed
chr2:151391503:D	2	151,391,503	G	GTA	0.014	0.014	0.826	1.032	0.142	0.781	1.364	0.449	Imputed
rs59986005	2	151,391,505	G	A	0.061	0.065	0.263	0.924	0.071	0.805	1.061	0.166	Imputed
rs6743187	2	151,392,059	C	T	0.279	0.280	0.984	0.999	0.038	0.928	1.076	0.980	Genotyped
rs16828317	2	151,392,542	A	G	0.080	0.078	0.619	1.032	0.062	0.913	1.166	0.772	Imputed
rs6729200	2	151,392,702	T	C	0.276	0.276	0.964	0.998	0.038	0.927	1.075	0.910	Imputed
rs59491196	2	151,392,704	T	G	0.115	0.113	0.690	1.021	0.053	0.921	1.133	0.411	Imputed
rs6752046	2	151,394,840	A	G	0.408	0.404	0.647	1.016	0.034	0.950	1.087	0.973	Imputed
rs181849767	2	151,394,863	A	G	0.019	0.022	0.182	0.849	0.123	0.668	1.080	0.803	Imputed
rs186103263	2	151,394,883	T	C	0.011	0.012	0.634	0.927	0.159	0.678	1.266	0.324	Imputed
rs141966899	2	151,394,977	C	T	0.022	0.024	0.462	0.918	0.116	0.732	1.152	0.049	Imputed
rs147592359	2	151,396,144	T	C	0.019	0.022	0.182	0.849	0.123	0.668	1.080	0.803	Imputed
rs149004581	2	151,396,729	C	T	0.018	0.019	0.398	0.898	0.128	0.699	1.153	0.282	Imputed
rs113859176	2	151,398,379	T	C	0.043	0.037	0.038	1.191	0.084	1.010	1.404	0.775	Imputed
rs144629681	2	151,398,442	A	T	0.091	0.091	0.998	1.000	0.059	0.891	1.122	0.513	Imputed
rs139627657	2	151,398,522	G	A	0.091	0.091	0.993	1.000	0.059	0.891	1.121	0.516	Imputed
rs12474629	2	151,398,669	C	A	0.280	0.278	0.767	1.011	0.038	0.939	1.089	0.747	Imputed
rs115857831	2	151,399,362	A	C	0.020	0.023	0.119	0.829	0.120	0.655	1.050	0.947	Imputed
rs140397859	2	151,399,541	T	C	0.091	0.091	0.993	1.000	0.059	0.891	1.121	0.516	Imputed
rs76217809	2	151,399,946	C	G	0.121	0.123	0.656	0.977	0.052	0.883	1.082	0.262	Imputed
rs148951689	2	151,400,822	C	T	0.019	0.022	0.171	0.846	0.123	0.665	1.076	0.778	Imputed
rs143726548	2	151,401,089	A	G	0.090	0.091	0.845	0.989	0.059	0.881	1.110	0.458	Imputed
rs10460349	2	151,401,133	T	A	0.418	0.421	0.766	0.990	0.034	0.925	1.059	0.746	Imputed
rs61452036	2	151,401,193	G	A	0.122	0.124	0.666	0.978	0.052	0.884	1.082	0.256	Imputed
rs114992768	2	151,401,630	T	C	0.014	0.017	0.083	0.782	0.143	0.591	1.034	0.721	Imputed
rs147419993	2	151,401,663	C	A	0.011	0.012	0.571	0.913	0.161	0.666	1.251	0.451	Imputed
rs6759010	2	151,401,771	G	A	0.284	0.284	0.996	1.000	0.038	0.929	1.076	0.885	Genotyped
rs76194710	2	151,402,631	C	T	0.091	0.091	0.982	0.999	0.059	0.890	1.121	0.501	Imputed
rs77386958	2	151,404,875	C	T	0.091	0.091	0.982	0.999	0.059	0.890	1.121	0.501	Imputed
rs138223236	2	151,406,071	A	G	0.012	0.013	0.627	0.928	0.154	0.686	1.255	0.995	Imputed
rs141264331	2	151,407,469	C	G	0.019	0.022	0.154	0.840	0.123	0.661	1.068	0.803	Imputed
rs139092324	2	151,407,689	A	G	0.019	0.022	0.154	0.840	0.123	0.661	1.068	0.803	Imputed
rs145935860	2	151,407,989	A	G	0.019	0.022	0.154	0.840	0.123	0.661	1.068	0.803	Imputed
chr2:151408367:D	2	151,408,367	C	CAT	0.093	0.094	0.912	0.994	0.058	0.887	1.113	0.533	Imputed
rs10497062	2	151,408,827	C	T	0.092	0.092	0.916	1.006	0.058	0.897	1.128	0.509	Imputed
rs80152063	2	151,409,398	A	G	0.100	0.098	0.744	1.019	0.056	0.912	1.138	0.270	Imputed
rs7592790	2	151,409,434	A	G	0.123	0.124	0.796	0.987	0.052	0.892	1.092	0.203	Genotyped
rs17269913	2	151,409,801	C	T	0.092	0.092	0.926	1.005	0.058	0.897	1.128	0.513	Imputed
rs56269515	2	151,411,010	T	C	0.037	0.041	0.262	0.905	0.089	0.761	1.078	0.328	Imputed
rs114286693	2	151,411,283	T	C	0.012	0.014	0.320	0.856	0.155	0.631	1.161	0.058	Imputed

rs140985687	2	151,411,898	A	G	0.087	0.088	0.738	0.980	0.060	0.871	1.102	0.364	Imputed
rs7571510	2	151,411,955	C	A	0.020	0.023	0.157	0.843	0.121	0.665	1.068	0.637	Imputed
rs75540734	2	151,412,034	C	T	0.091	0.091	0.961	1.003	0.059	0.894	1.125	0.412	Imputed
rs1406242	2	151,413,170	T	C	0.392	0.395	0.743	0.989	0.035	0.924	1.058	0.542	Genotyped
rs79906955	2	151,414,185	A	G	0.091	0.091	0.881	0.991	0.059	0.883	1.112	0.438	Imputed
rs149336132	2	151,414,637	A	G	0.013	0.017	0.097	0.787	0.145	0.592	1.045	0.589	Imputed
rs191349070	2	151,415,160	T	C	0.015	0.017	0.463	0.904	0.137	0.692	1.183	0.832	Imputed
rs116022144	2	151,415,478	T	C	0.091	0.091	0.950	0.996	0.059	0.888	1.118	0.446	Imputed
rs189174205	2	151,416,028	A	T	0.019	0.022	0.154	0.840	0.123	0.661	1.068	0.803	Imputed
rs112580855	2	151,417,384	T	C	0.091	0.091	0.955	0.997	0.059	0.888	1.118	0.444	Imputed
rs148778157	2	151,418,278	T	G	0.092	0.091	0.897	1.008	0.058	0.899	1.130	0.500	Imputed
rs75212287	2	151,420,583	G	T	0.027	0.023	0.129	1.171	0.105	0.954	1.438	0.155	Imputed
chr2:151420599:D	2	151,420,599	T	TG	0.019	0.017	0.483	1.092	0.125	0.854	1.396	0.291	Imputed
rs7605119	2	151,420,609	G	T	0.099	0.101	0.687	0.978	0.056	0.875	1.092	0.268	Imputed
rs11693304	2	151,420,663	C	T	0.398	0.400	0.770	0.990	0.035	0.925	1.059	0.377	Imputed
rs112747600	2	151,420,855	C	T	0.123	0.124	0.787	0.986	0.052	0.892	1.091	0.187	Imputed
rs78014793	2	151,422,351	C	A	0.092	0.092	0.916	1.006	0.058	0.897	1.128	0.509	Imputed
rs11680193	2	151,423,345	C	A	0.275	0.276	0.960	0.998	0.038	0.927	1.075	0.975	Imputed
rs2340855	2	151,423,578	G	T	0.030	0.026	0.116	1.168	0.099	0.962	1.419	0.591	Imputed
rs184388311	2	151,423,849	T	C	0.021	0.026	0.041	0.790	0.116	0.629	0.992	0.492	Imputed
rs17269927	2	151,424,784	C	T	0.092	0.091	0.908	1.007	0.058	0.898	1.129	0.502	Imputed
rs10497063	2	151,425,052	T	C	0.100	0.098	0.744	1.019	0.056	0.912	1.138	0.270	Imputed
rs16828329	2	151,425,550	A	C	0.022	0.025	0.226	0.871	0.114	0.696	1.090	0.732	Imputed
chr2:151425600:I	2	151,425,600	GT	G	0.095	0.098	0.424	0.955	0.058	0.853	1.069	0.989	Imputed
rs55806653	2	151,426,804	A	T	0.260	0.259	0.844	1.008	0.039	0.934	1.087	0.979	Imputed
rs75806486	2	151,426,843	C	T	0.087	0.088	0.682	0.976	0.060	0.867	1.098	0.283	Imputed
chr2:151427098:D	2	151,427,098	C	CGT	0.024	0.027	0.195	0.868	0.110	0.699	1.076	0.270	Imputed
rs75213735	2	151,427,203	G	A	0.022	0.024	0.349	0.897	0.116	0.714	1.126	0.122	Imputed
rs12692762	2	151,427,260	A	C	0.248	0.251	0.769	0.989	0.039	0.915	1.068	0.943	Imputed
chr2:151428474:D	2	151,428,474	GA	G	0.012	0.012	0.905	0.982	0.156	0.723	1.332	0.529	Imputed
rs146719876	2	151,428,543	C	T	0.020	0.021	0.357	0.894	0.121	0.705	1.135	0.867	Imputed
rs716730	2	151,429,031	T	C	0.107	0.111	0.457	0.960	0.055	0.863	1.069	0.261	Imputed
rs114067806	2	151,429,172	A	G	0.047	0.049	0.567	0.955	0.080	0.817	1.117	0.785	Imputed
rs4635532	2	151,429,183	T	C	0.243	0.245	0.789	0.990	0.039	0.916	1.069	0.985	Genotyped
rs72867408	2	151,430,498	T	C	0.032	0.029	0.200	1.132	0.097	0.937	1.368	0.391	Imputed
rs2340857	2	151,430,525	C	T	0.445	0.436	0.324	1.034	0.034	0.967	1.106	0.014	Genotyped
rs79226093	2	151,431,021	T	C	0.063	0.066	0.460	0.950	0.069	0.829	1.088	0.227	Imputed
rs11685634	2	151,431,607	A	G	0.306	0.306	0.987	1.001	0.037	0.931	1.075	0.464	Imputed
rs34064007	2	151,433,554	A	G	0.010	0.012	0.257	0.825	0.170	0.592	1.151	0.652	Imputed
rs142034547	2	151,433,649	A	G	0.033	0.030	0.176	1.138	0.096	0.944	1.373	0.409	Imputed

rs112020773	2	151,433,701	T	C	0.071	0.074	0.530	0.960	0.066	0.843	1.091	0.381	Imputed
rs112761460	2	151,433,708	A	G	0.065	0.068	0.487	0.954	0.068	0.834	1.090	0.181	Imputed
rs34849534	2	151,434,279	A	C	0.492	0.497	0.541	0.980	0.034	0.917	1.047	0.002	Imputed
rs112077418	2	151,434,333	C	G	0.149	0.156	0.143	0.933	0.047	0.850	1.024	0.200	Imputed
rs10930161	2	151,434,434	T	C	0.439	0.430	0.280	1.038	0.034	0.970	1.109	0.016	Imputed
rs142726282	2	151,434,473	A	G	0.014	0.013	0.576	1.084	0.144	0.817	1.439	0.420	Imputed
rs13034102	2	151,434,697	A	G	0.016	0.015	0.578	1.079	0.137	0.826	1.411	0.214	Imputed
rs6757157	2	151,435,489	A	C	0.491	0.496	0.570	0.981	0.034	0.918	1.048	0.002	Imputed
rs10469691	2	151,436,367	G	A	0.493	0.496	0.676	0.986	0.034	0.923	1.054	0.002	Genotyped
rs10469692	2	151,436,719	C	G	0.489	0.495	0.482	0.977	0.034	0.914	1.043	0.001	Imputed
rs142894171	2	151,438,271	T	G	0.011	0.011	0.834	1.034	0.162	0.754	1.419	0.567	Imputed
rs116386362	2	151,439,595	A	T	0.017	0.013	0.061	1.281	0.133	0.988	1.661	0.669	Imputed
rs2340858	2	151,440,176	A	C	0.442	0.433	0.303	1.036	0.034	0.969	1.107	0.017	Imputed
rs4426529	2	151,441,164	T	C	0.086	0.090	0.440	0.955	0.060	0.849	1.074	0.162	Imputed
rs28617484	2	151,443,503	C	T	0.113	0.107	0.322	1.055	0.054	0.949	1.172	0.018	Imputed
rs13005097	2	151,443,505	T	G	0.021	0.020	0.494	1.084	0.118	0.861	1.366	0.303	Imputed
rs4438475	2	151,443,571	C	G	0.448	0.439	0.280	1.037	0.034	0.971	1.109	0.016	Imputed
rs16828344	2	151,443,640	A	T	0.066	0.070	0.387	0.943	0.068	0.825	1.077	0.168	Imputed
rs4233661	2	151,444,333	G	C	0.324	0.323	0.817	1.008	0.036	0.939	1.082	0.368	Imputed
rs142063051	2	151,445,158	C	T	0.020	0.018	0.391	1.111	0.122	0.874	1.411	0.072	Imputed
rs56767144	2	151,445,206	A	C	0.122	0.115	0.195	1.070	0.052	0.966	1.184	0.016	Imputed
chr2:151445902:I	2	151,445,902	TG	T	0.014	0.013	0.589	1.082	0.146	0.813	1.441	0.241	Imputed
chr2:151445935:D	2	151,445,935	AT	A	0.324	0.319	0.572	1.021	0.036	0.951	1.096	0.467	Imputed
rs6720165	2	151,445,936	T	A	0.304	0.305	0.978	0.999	0.037	0.930	1.074	0.577	Imputed
rs16828351	2	151,446,187	C	G	0.066	0.070	0.390	0.943	0.068	0.826	1.078	0.169	Imputed
rs74749104	2	151,446,247	T	C	0.064	0.067	0.444	0.949	0.069	0.829	1.086	0.156	Imputed
rs77009579	2	151,447,010	C	T	0.013	0.011	0.240	1.198	0.154	0.886	1.620	0.462	Imputed
rs17197737	2	151,447,063	G	T	0.148	0.157	0.078	0.920	0.047	0.838	1.009	0.216	Imputed
rs114928248	2	151,447,264	T	C	0.064	0.067	0.448	0.949	0.069	0.829	1.086	0.157	Imputed
chr2:151447508:D	2	151,447,508	C	CG	0.113	0.107	0.319	1.055	0.054	0.950	1.172	0.018	Imputed
chr2:151447541:D	2	151,447,541	A	AT	0.128	0.135	0.167	0.932	0.051	0.843	1.030	0.789	Imputed
rs148966634	2	151,447,771	A	G	0.016	0.013	0.054	1.297	0.136	0.994	1.692	0.470	Imputed
rs2340859	2	151,448,583	A	T	0.486	0.490	0.583	0.982	0.034	0.919	1.049	0.002	Imputed
rs142400617	2	151,451,055	A	G	0.027	0.028	0.795	0.973	0.104	0.794	1.194	0.968	Imputed
rs148261929	2	151,451,982	T	C	0.012	0.014	0.437	0.890	0.151	0.661	1.197	0.152	Imputed
rs6720091	2	151,452,042	A	T	0.448	0.438	0.224	1.042	0.034	0.975	1.114	0.023	Imputed
rs16828361	2	151,452,910	G	C	0.066	0.069	0.469	0.952	0.068	0.833	1.088	0.109	Imputed
rs4664659	2	151,454,525	A	G	0.312	0.312	0.937	1.003	0.037	0.934	1.077	0.370	Imputed
rs2340860	2	151,455,164	G	A	0.309	0.310	0.982	0.999	0.037	0.930	1.073	0.637	Imputed
rs10198421	2	151,455,782	A	G	0.066	0.069	0.562	0.961	0.068	0.841	1.098	0.079	Imputed

rs61151072	2	151,455,784	T	C	0.021	0.020	0.651	1.055	0.118	0.837	1.331	0.346	Imputed
rs115392640	2	151,456,843	C	T	0.064	0.067	0.493	0.954	0.069	0.833	1.092	0.070	Imputed
rs12615315	2	151,456,951	T	C	0.479	0.483	0.577	0.981	0.034	0.918	1.049	0.002	Imputed
rs2340861	2	151,457,605	A	G	0.309	0.310	0.934	0.997	0.037	0.928	1.071	0.627	Genotyped
rs13029435	2	151,459,014	A	G	0.021	0.020	0.523	1.079	0.119	0.855	1.361	0.292	Imputed
rs113725234	2	151,459,072	C	A	0.066	0.068	0.579	0.963	0.068	0.843	1.100	0.081	Imputed
rs2340862	2	151,459,575	C	T	0.276	0.280	0.602	0.980	0.038	0.910	1.056	0.742	Imputed
rs16828368	2	151,459,630	C	T	0.116	0.110	0.300	1.057	0.053	0.952	1.172	0.031	Imputed
rs35817225	2	151,459,829	A	G	0.021	0.020	0.670	1.052	0.118	0.834	1.327	0.339	Imputed
rs62169720	2	151,460,462	C	T	0.038	0.033	0.132	1.144	0.090	0.960	1.364	0.848	Imputed
rs7571312	2	151,461,398	T	C	0.479	0.483	0.571	0.981	0.034	0.918	1.048	0.001	Imputed
rs35694757	2	151,461,770	G	A	0.021	0.020	0.523	1.079	0.119	0.855	1.361	0.292	Imputed
rs34683308	2	151,461,887	T	A	0.021	0.020	0.523	1.079	0.119	0.855	1.361	0.292	Imputed
rs116243630	2	151,462,640	G	T	0.064	0.067	0.493	0.954	0.069	0.833	1.092	0.070	Imputed
chr2:151462839:D	2	151,462,839	T	TTAA	0.047	0.052	0.131	0.887	0.079	0.760	1.037	0.113	Imputed
rs16828372	2	151,462,867	T	C	0.145	0.137	0.152	1.072	0.048	0.975	1.178	0.007	Genotyped
rs72867428	2	151,462,909	G	C	0.034	0.031	0.246	1.116	0.094	0.927	1.342	0.606	Imputed
rs7604619	2	151,463,554	T	G	0.483	0.488	0.523	0.979	0.034	0.916	1.046	0.001	Imputed
rs78448596	2	151,464,012	A	G	0.020	0.020	0.989	0.998	0.121	0.788	1.265	0.211	Imputed
rs2879878	2	151,464,695	C	T	0.143	0.150	0.153	0.934	0.048	0.849	1.026	0.217	Genotyped
rs10930176	2	151,465,458	T	C	0.114	0.105	0.089	1.095	0.053	0.986	1.215	0.081	Genotyped
rs143073275	2	151,466,331	A	G	0.023	0.021	0.387	1.103	0.113	0.883	1.377	0.969	Imputed
rs79490326	2	151,466,451	C	T	0.141	0.149	0.113	0.926	0.048	0.843	1.018	0.234	Imputed
rs11676530	2	151,467,243	A	G	0.141	0.149	0.119	0.928	0.048	0.844	1.020	0.244	Imputed
rs79301607	2	151,467,314	A	G	0.053	0.041	2.15E-03	1.268	0.077	1.090	1.476	0.082	Imputed
rs2340863	2	151,467,617	G	A	0.310	0.311	0.949	0.998	0.037	0.929	1.072	0.641	Imputed
rs4664678	2	151,468,414	C	T	0.111	0.102	0.091	1.096	0.054	0.985	1.218	0.113	Imputed
rs4664679	2	151,468,476	A	G	0.479	0.483	0.591	0.982	0.034	0.919	1.049	0.002	Imputed
rs71413699	2	151,468,918	T	G	0.021	0.020	0.523	1.079	0.119	0.855	1.361	0.292	Imputed
rs2340864	2	151,469,069	T	C	0.479	0.484	0.548	0.980	0.034	0.917	1.047	0.002	Genotyped
rs10202222	2	151,470,729	A	C	0.240	0.242	0.764	0.988	0.040	0.914	1.068	0.890	Genotyped
rs62169721	2	151,471,941	T	G	0.026	0.026	0.905	0.987	0.107	0.801	1.217	0.085	Imputed
rs6758119	2	151,471,993	A	G	0.309	0.310	0.971	0.999	0.037	0.930	1.073	0.659	Imputed
rs75015450	2	151,472,002	A	G	0.064	0.067	0.484	0.953	0.069	0.832	1.091	0.075	Imputed
chr2:151472384:I	2	151,472,384	AT	A	0.064	0.067	0.484	0.953	0.069	0.832	1.091	0.075	Imputed
rs12052383	2	151,472,497	C	G	0.144	0.136	0.201	1.064	0.048	0.968	1.170	0.007	Imputed
rs78776788	2	151,472,902	A	G	0.064	0.067	0.484	0.953	0.069	0.832	1.091	0.075	Imputed
rs35767020	2	151,473,374	T	C	0.230	0.233	0.589	0.979	0.040	0.904	1.059	0.959	Imputed
rs2879927	2	151,473,402	G	A	0.311	0.312	0.897	0.995	0.037	0.926	1.069	0.679	Imputed
rs144295405	2	151,474,089	C	T	0.012	0.011	0.313	1.169	0.155	0.863	1.584	0.717	Imputed

rs74432168	2	151,474,278	C	G	0.013	0.012	0.444	1.121	0.149	0.837	1.501	0.532	Imputed
rs76966238	2	151,474,362	T	C	0.011	0.011	0.776	0.954	0.165	0.690	1.319	0.076	Imputed
rs16828387	2	151,474,999	A	G	0.031	0.028	0.255	1.119	0.098	0.923	1.356	0.539	Imputed
rs74877856	2	151,475,537	C	A	0.018	0.024	0.034	0.768	0.124	0.602	0.980	0.837	Imputed
rs17269955	2	151,476,720	G	A	0.020	0.021	0.809	0.971	0.122	0.764	1.233	0.169	Imputed
rs4334493	2	151,477,785	T	C	0.110	0.105	0.371	1.050	0.054	0.944	1.168	0.034	Imputed
rs2341059	2	151,478,268	C	T	0.111	0.105	0.380	1.049	0.054	0.943	1.167	0.041	Imputed
rs62169724	2	151,478,463	A	G	0.026	0.027	0.871	0.983	0.105	0.800	1.208	0.283	Imputed
rs72867442	2	151,478,990	G	A	0.030	0.027	0.200	1.136	0.100	0.935	1.382	0.365	Imputed
rs2341060	2	151,479,273	T	C	0.109	0.104	0.420	1.045	0.055	0.939	1.163	0.035	Imputed
rs4297879	2	151,479,571	G	A	0.109	0.105	0.451	1.042	0.055	0.937	1.160	0.044	Imputed
rs140385447	2	151,481,213	T	C	0.014	0.010	0.030	1.375	0.148	1.029	1.836	0.353	Imputed
rs2341061	2	151,482,152	G	C	0.109	0.104	0.420	1.045	0.055	0.939	1.163	0.035	Imputed
rs4664689	2	151,482,490	T	C	0.109	0.104	0.420	1.045	0.055	0.939	1.163	0.035	Imputed
rs35733313	2	151,482,607	T	C	0.014	0.015	0.461	0.901	0.142	0.682	1.189	0.587	Imputed
rs10930186	2	151,483,006	T	C	0.456	0.449	0.527	1.022	0.034	0.956	1.092	0.023	Imputed
rs34463872	2	151,483,065	C	G	0.346	0.345	0.901	1.004	0.036	0.937	1.077	0.327	Imputed
rs2341062	2	151,484,625	T	C	0.109	0.104	0.429	1.044	0.055	0.938	1.162	0.043	Imputed
rs2341063	2	151,484,903	T	G	0.109	0.104	0.429	1.044	0.055	0.938	1.162	0.043	Imputed
rs2341064	2	151,484,929	A	G	0.110	0.105	0.425	1.044	0.054	0.939	1.162	0.043	Imputed
rs2341065	2	151,484,942	G	A	0.110	0.105	0.425	1.044	0.054	0.939	1.162	0.043	Imputed
rs13028473	2	151,485,109	C	A	0.314	0.316	0.724	0.987	0.036	0.919	1.060	0.473	Imputed
chr2:151485177:D	2	151,485,177	AT	A	0.455	0.449	0.565	1.020	0.034	0.954	1.090	0.022	Imputed
rs12692786	2	151,485,293	A	G	0.346	0.345	0.893	1.005	0.036	0.937	1.077	0.333	Imputed
rs72867459	2	151,485,314	A	G	0.031	0.028	0.162	1.147	0.098	0.947	1.389	0.394	Imputed
rs2341066	2	151,485,402	G	C	0.110	0.105	0.425	1.044	0.054	0.939	1.162	0.043	Imputed
rs2341067	2	151,485,406	C	T	0.110	0.105	0.425	1.044	0.054	0.939	1.162	0.043	Imputed
rs2341068	2	151,485,519	A	G	0.110	0.105	0.404	1.047	0.054	0.941	1.164	0.046	Genotyped
rs2247416	2	151,485,649	C	T	0.110	0.105	0.425	1.044	0.054	0.939	1.162	0.043	Imputed
rs2290817	2	151,485,672	G	T	0.118	0.112	0.395	1.046	0.053	0.943	1.160	0.065	Imputed
rs2290816	2	151,485,701	T	A	0.110	0.105	0.425	1.044	0.054	0.939	1.162	0.043	Imputed
rs150205106	2	151,485,718	A	C	0.008	0.010	0.167	0.771	0.188	0.533	1.115	0.328	Imputed
rs10497064	2	151,485,720	A	G	0.112	0.108	0.526	1.035	0.054	0.931	1.150	0.039	Imputed
rs13409790	2	151,485,825	C	T	0.054	0.043	2.84E-03	1.256	0.076	1.082	1.460	0.147	Imputed
rs2290815	2	151,485,881	G	A	0.110	0.105	0.426	1.044	0.054	0.939	1.162	0.043	Imputed
rs4664692	2	151,486,025	C	T	0.110	0.105	0.402	1.047	0.054	0.941	1.165	0.036	Imputed
rs147186292	2	151,486,207	A	G	0.012	0.015	0.096	0.776	0.153	0.575	1.046	0.406	Imputed
rs2290814	2	151,486,321	C	T	0.346	0.345	0.886	1.005	0.036	0.937	1.078	0.303	Imputed
rs2290813	2	151,486,385	C	G	0.346	0.345	0.886	1.005	0.036	0.937	1.078	0.303	Imputed
rs13419254	2	151,486,410	A	G	0.346	0.345	0.886	1.005	0.036	0.937	1.078	0.303	Imputed

rs13413391	2	151,486,803	C	T	0.346	0.345	0.886	1.005	0.036	0.937	1.078	0.303	Imputed
rs4355108	2	151,487,613	A	G	0.455	0.450	0.554	1.020	0.034	0.955	1.091	0.023	Genotyped
rs2341070	2	151,487,921	G	T	0.456	0.450	0.509	1.023	0.034	0.957	1.093	0.023	Imputed
chr2:151488204:D	2	151,488,204	T	TC	0.011	0.012	0.769	0.955	0.159	0.699	1.303	0.504	Imputed
rs2341071	2	151,488,351	T	A	0.110	0.105	0.397	1.047	0.054	0.941	1.165	0.043	Imputed
rs2341072	2	151,488,396	T	A	0.111	0.107	0.554	1.033	0.054	0.929	1.148	0.039	Imputed
rs2341073	2	151,489,012	A	C	0.110	0.105	0.397	1.047	0.054	0.941	1.165	0.043	Imputed
rs751614	2	151,489,116	T	C	0.456	0.449	0.509	1.023	0.034	0.957	1.093	0.023	Imputed
rs2341074	2	151,489,339	C	T	0.110	0.105	0.397	1.047	0.054	0.941	1.165	0.043	Imputed
rs2341075	2	151,489,419	A	G	0.113	0.109	0.576	1.031	0.054	0.928	1.145	0.046	Imputed
rs13031659	2	151,489,452	C	T	0.459	0.453	0.538	1.021	0.034	0.955	1.091	0.026	Imputed
rs143229390	2	151,489,623	T	C	0.030	0.027	0.166	1.148	0.099	0.945	1.394	0.393	Imputed
chr2:151489656:D	2	151,489,656	AG	A	0.115	0.111	0.654	1.024	0.053	0.923	1.137	0.048	Imputed
rs71413701	2	151,489,681	C	T	0.014	0.015	0.453	0.899	0.142	0.681	1.187	0.581	Imputed
rs62167793	2	151,489,682	C	T	0.022	0.020	0.425	1.097	0.117	0.873	1.379	0.436	Imputed
rs2341077	2	151,489,796	A	C	0.455	0.449	0.506	1.023	0.034	0.957	1.093	0.025	Imputed
rs2341078	2	151,489,909	C	T	0.109	0.104	0.398	1.047	0.055	0.941	1.165	0.047	Imputed
rs34871167	2	151,490,081	G	A	0.346	0.345	0.918	1.004	0.036	0.936	1.076	0.324	Imputed
chr2:151490360:I	2	151,490,360	G	GC	0.120	0.117	0.609	1.027	0.052	0.927	1.138	0.048	Imputed
rs7600949	2	151,490,361	A	C	0.121	0.118	0.642	1.025	0.052	0.925	1.135	0.046	Imputed
rs7559963	2	151,490,362	G	A	0.120	0.116	0.556	1.031	0.052	0.931	1.143	0.034	Imputed
rs7603890	2	151,490,551	T	C	0.110	0.104	0.368	1.050	0.054	0.944	1.169	0.040	Imputed
rs13390664	2	151,490,593	C	T	0.346	0.345	0.924	1.003	0.036	0.936	1.076	0.332	Imputed
rs72867486	2	151,490,614	G	A	0.030	0.027	0.200	1.136	0.100	0.935	1.382	0.365	Imputed
rs13020543	2	151,490,702	A	G	0.314	0.316	0.729	0.987	0.036	0.919	1.061	0.498	Imputed
rs4380232	2	151,490,718	T	C	0.109	0.104	0.388	1.048	0.055	0.942	1.166	0.048	Imputed
rs4514872	2	151,490,766	C	T	0.109	0.104	0.388	1.048	0.055	0.942	1.166	0.048	Imputed
rs4664694	2	151,491,400	A	G	0.347	0.345	0.886	1.005	0.036	0.938	1.078	0.321	Imputed
rs707021	2	151,491,805	G	A	0.455	0.449	0.511	1.023	0.034	0.957	1.093	0.027	Imputed
rs4664150	2	151,491,950	G	A	0.109	0.104	0.388	1.048	0.055	0.942	1.166	0.048	Imputed
rs7591161	2	151,492,110	T	C	0.455	0.449	0.511	1.023	0.034	0.957	1.093	0.027	Imputed
rs12692788	2	151,492,472	C	T	0.346	0.345	0.896	1.005	0.036	0.937	1.077	0.318	Imputed
rs12692789	2	151,492,597	C	T	0.346	0.345	0.896	1.005	0.036	0.937	1.077	0.318	Imputed
rs72867488	2	151,492,608	A	C	0.022	0.026	0.049	0.797	0.115	0.636	0.999	0.588	Imputed
rs13012442	2	151,492,842	T	A	0.346	0.345	0.905	1.004	0.036	0.937	1.077	0.315	Imputed
chr2:151493164:I	2	151,493,164	TTTTG	T	0.327	0.331	0.601	0.981	0.036	0.914	1.053	0.460	Imputed
chr2:151493165:I	2	151,493,165	TTTG	T	0.332	0.335	0.721	0.987	0.036	0.920	1.059	0.506	Imputed
chr2:151493166:I	2	151,493,166	TTG	T	0.332	0.335	0.715	0.987	0.036	0.920	1.059	0.515	Imputed
rs11889316	2	151,493,193	A	G	0.133	0.125	0.206	1.065	0.050	0.966	1.175	0.120	Imputed
rs35926151	2	151,493,302	C	T	0.346	0.345	0.921	1.004	0.036	0.936	1.076	0.342	Imputed

rs77403773	2	151,493,395	C	T	0.314	0.316	0.713	0.987	0.036	0.919	1.060	0.535	Imputed
rs11883782	2	151,493,452	C	T	0.456	0.450	0.576	1.019	0.034	0.954	1.089	0.040	Imputed
rs11894221	2	151,493,463	G	T	0.456	0.451	0.568	1.020	0.034	0.954	1.090	0.028	Imputed
rs16828469	2	151,493,632	A	G	0.346	0.345	0.921	1.004	0.036	0.936	1.076	0.342	Imputed
rs72867495	2	151,493,693	A	G	0.346	0.345	0.921	1.004	0.036	0.936	1.076	0.342	Imputed
rs71413702	2	151,493,786	C	A	0.346	0.345	0.921	1.004	0.036	0.936	1.076	0.342	Imputed
rs2341079	2	151,494,079	C	T	0.346	0.345	0.921	1.004	0.036	0.936	1.076	0.342	Imputed
rs2341080	2	151,494,170	C	T	0.456	0.450	0.566	1.020	0.034	0.954	1.090	0.030	Imputed
rs4271766	2	151,494,381	A	G	0.346	0.345	0.911	1.004	0.036	0.936	1.076	0.355	Genotyped
rs10497065	2	151,494,540	G	A	0.346	0.345	0.940	1.003	0.036	0.935	1.075	0.370	Imputed
rs10497066	2	151,494,809	A	G	0.346	0.345	0.965	1.002	0.036	0.934	1.074	0.397	Genotyped
rs7602198	2	151,494,981	T	G	0.109	0.103	0.388	1.048	0.055	0.942	1.167	0.042	Imputed
rs4638794	2	151,495,574	G	T	0.463	0.455	0.378	1.030	0.034	0.964	1.101	0.035	Imputed
rs4664698	2	151,495,631	C	T	0.366	0.364	0.793	1.009	0.035	0.942	1.081	0.377	Imputed
rs4664699	2	151,495,654	G	T	0.353	0.350	0.751	1.011	0.035	0.944	1.084	0.382	Imputed
rs2341081	2	151,495,697	C	T	0.109	0.104	0.392	1.048	0.055	0.942	1.166	0.051	Imputed
rs4627563	2	151,495,976	G	T	0.463	0.455	0.384	1.030	0.034	0.964	1.101	0.034	Imputed
rs16828502	2	151,496,151	A	G	0.031	0.029	0.220	1.128	0.098	0.931	1.365	0.346	Genotyped
rs7562436	2	151,496,161	G	A	0.110	0.104	0.330	1.055	0.054	0.948	1.173	0.048	Genotyped
rs74924751	2	151,496,338	T	A	0.014	0.012	0.296	1.163	0.144	0.877	1.543	0.332	Imputed
rs7589990	2	151,496,454	C	T	0.463	0.454	0.359	1.032	0.034	0.965	1.103	0.032	Imputed
rs7603668	2	151,496,524	A	G	0.455	0.448	0.527	1.022	0.034	0.956	1.092	0.033	Genotyped
rs2341082	2	151,496,784	G	A	0.110	0.104	0.318	1.056	0.054	0.949	1.175	0.049	Imputed
rs7593427	2	151,497,120	C	A	0.109	0.104	0.420	1.045	0.055	0.939	1.163	0.049	Imputed
rs12473120	2	151,497,342	T	A	0.109	0.103	0.336	1.054	0.055	0.947	1.173	0.045	Imputed
rs2341083	2	151,497,386	C	A	0.109	0.103	0.336	1.054	0.055	0.947	1.173	0.045	Imputed
rs2341084	2	151,497,466	T	G	0.109	0.103	0.336	1.054	0.055	0.947	1.173	0.045	Imputed
rs7569499	2	151,497,781	G	A	0.109	0.103	0.336	1.054	0.055	0.947	1.173	0.045	Imputed
rs7560326	2	151,497,939	T	A	0.109	0.103	0.336	1.054	0.055	0.947	1.173	0.045	Imputed
rs2341085	2	151,498,098	T	C	0.109	0.103	0.336	1.054	0.055	0.947	1.173	0.045	Imputed
rs7557930	2	151,498,170	A	G	0.109	0.103	0.336	1.054	0.055	0.947	1.173	0.045	Imputed
rs7597424	2	151,498,386	C	G	0.109	0.103	0.336	1.054	0.055	0.947	1.173	0.045	Imputed
rs4365460	2	151,498,722	G	C	0.109	0.103	0.336	1.054	0.055	0.947	1.173	0.045	Imputed
rs4408725	2	151,498,770	C	T	0.109	0.103	0.325	1.055	0.055	0.948	1.174	0.049	Genotyped
chr2:151498896:D	2	151,498,896	C	CA	0.105	0.099	0.362	1.052	0.056	0.944	1.173	0.100	Imputed
rs4530366	2	151,498,953	A	G	0.109	0.103	0.336	1.054	0.055	0.947	1.173	0.045	Imputed
rs4600650	2	151,499,059	T	A	0.109	0.103	0.336	1.054	0.055	0.947	1.173	0.045	Imputed
rs9287861	2	151,499,283	T	C	0.109	0.103	0.336	1.054	0.055	0.947	1.173	0.045	Imputed
rs2879931	2	151,499,332	C	T	0.109	0.103	0.336	1.054	0.055	0.947	1.173	0.045	Imputed
rs2341086	2	151,499,500	A	C	0.109	0.103	0.336	1.054	0.055	0.947	1.173	0.045	Imputed

rs4530365	2	151,499,771	T	C	0.109	0.103	0.336	1.054	0.055	0.947	1.173	0.045	Imputed
rs4233665	2	151,499,850	A	G	0.109	0.103	0.336	1.054	0.055	0.947	1.173	0.045	Imputed
rs4233666	2	151,500,116	T	A	0.109	0.103	0.336	1.054	0.055	0.947	1.173	0.045	Imputed
rs17197765	2	151,500,131	G	A	0.030	0.033	0.259	0.894	0.099	0.737	1.085	0.794	Imputed
rs4233667	2	151,500,148	C	G	0.109	0.103	0.336	1.054	0.055	0.947	1.173	0.045	Imputed
rs9287862	2	151,500,518	C	T	0.094	0.088	0.320	1.060	0.058	0.945	1.188	0.127	Imputed
rs10188741	2	151,500,544	C	T	0.094	0.088	0.320	1.060	0.058	0.945	1.188	0.127	Imputed
rs9287863	2	151,500,658	T	C	0.448	0.439	0.368	1.031	0.034	0.965	1.102	0.072	Imputed
rs2341087	2	151,501,020	G	A	0.109	0.103	0.336	1.054	0.055	0.947	1.173	0.045	Imputed
rs2341088	2	151,501,033	T	A	0.095	0.089	0.291	1.063	0.058	0.949	1.191	0.162	Imputed
rs6710003	2	151,501,328	G	C	0.094	0.088	0.320	1.060	0.058	0.945	1.188	0.127	Imputed
rs12614085	2	151,501,545	C	T	0.094	0.088	0.320	1.060	0.058	0.945	1.188	0.127	Imputed
chr2:151501862:D	2	151,501,862	CGT	C	0.129	0.122	0.263	1.058	0.051	0.958	1.169	0.470	Imputed
rs10930197	2	151,501,863	G	A	0.109	0.102	0.197	1.073	0.055	0.964	1.194	0.202	Imputed
rs10930198	2	151,501,864	T	C	0.100	0.093	0.237	1.070	0.057	0.957	1.195	0.098	Imputed
rs10930199	2	151,501,903	T	C	0.441	0.434	0.519	1.022	0.034	0.956	1.093	0.083	Imputed
chr2:151502093:D	2	151,502,093	CA	C	0.094	0.089	0.346	1.057	0.058	0.943	1.184	0.135	Imputed
rs11894862	2	151,502,122	A	G	0.094	0.088	0.317	1.060	0.058	0.946	1.188	0.128	Imputed
rs11899711	2	151,502,215	G	C	0.094	0.088	0.317	1.060	0.058	0.946	1.188	0.128	Imputed
rs10497067	2	151,502,303	T	C	0.094	0.088	0.317	1.060	0.058	0.946	1.188	0.128	Imputed
rs10497068	2	151,502,539	C	A	0.094	0.088	0.317	1.060	0.058	0.946	1.188	0.128	Imputed
rs13019271	2	151,502,666	C	G	0.094	0.088	0.317	1.060	0.058	0.946	1.188	0.128	Imputed
rs2341089	2	151,502,890	A	G	0.094	0.088	0.315	1.060	0.058	0.946	1.189	0.128	Imputed
rs80142141	2	151,502,903	C	G	0.121	0.125	0.577	0.972	0.052	0.878	1.075	0.335	Imputed
rs2341090	2	151,503,163	T	C	0.094	0.089	0.354	1.056	0.058	0.942	1.183	0.133	Imputed
rs10432416	2	151,503,343	T	C	0.090	0.084	0.344	1.058	0.059	0.942	1.189	0.257	Imputed
rs2341091	2	151,503,453	T	C	0.094	0.088	0.312	1.061	0.058	0.946	1.189	0.129	Imputed
rs2879932	2	151,503,700	A	G	0.094	0.088	0.302	1.062	0.058	0.948	1.191	0.171	Genotyped
rs10930203	2	151,503,926	G	A	0.095	0.089	0.268	1.066	0.058	0.952	1.195	0.171	Imputed
rs10497069	2	151,504,004	G	C	0.109	0.103	0.314	1.057	0.055	0.949	1.176	0.056	Imputed
rs13428302	2	151,504,581	A	G	0.080	0.073	0.198	1.085	0.063	0.959	1.227	0.115	Imputed
rs10803803	2	151,504,694	T	C	0.093	0.087	0.366	1.055	0.059	0.940	1.183	0.143	Genotyped
rs72869138	2	151,505,104	A	C	0.031	0.028	0.270	1.115	0.099	0.919	1.353	0.450	Imputed
rs74350099	2	151,505,277	T	A	0.089	0.083	0.340	1.059	0.060	0.942	1.191	0.221	Imputed
rs17197779	2	151,505,948	T	C	0.050	0.048	0.605	1.041	0.078	0.894	1.212	0.228	Imputed
rs68181431	2	151,506,367	G	C	0.316	0.318	0.730	0.988	0.036	0.920	1.061	0.528	Imputed
rs2341092	2	151,506,626	T	C	0.125	0.116	0.124	1.082	0.051	0.979	1.197	0.093	Genotyped
rs72869142	2	151,507,014	T	A	0.031	0.028	0.215	1.130	0.098	0.932	1.371	0.492	Imputed
rs2341093	2	151,508,361	T	C	0.450	0.440	0.290	1.037	0.034	0.970	1.108	0.077	Imputed
rs79170387	2	151,508,450	G	A	0.031	0.028	0.253	1.119	0.099	0.923	1.358	0.709	Imputed

rs6731965	2	151,508,849	C	T	0.110	0.119	0.078	0.910	0.054	0.819	1.011	0.155	Imputed
rs79433433	2	151,508,883	T	A	0.031	0.029	0.251	1.118	0.098	0.924	1.354	0.622	Imputed
rs77575901	2	151,509,005	C	T	0.031	0.028	0.250	1.120	0.099	0.923	1.360	0.585	Imputed
rs187580709	2	151,509,205	A	G	0.010	0.013	0.153	0.792	0.164	0.574	1.092	0.328	Imputed
rs12463905	2	151,509,486	G	A	0.327	0.326	0.960	1.002	0.036	0.934	1.075	0.690	Genotyped
rs115670836	2	151,509,870	C	A	0.015	0.011	0.024	1.375	0.142	1.041	1.814	0.447	Imputed
rs13018941	2	151,510,404	G	C	0.452	0.442	0.294	1.036	0.034	0.970	1.108	0.117	Imputed
rs10201751	2	151,511,111	A	G	0.093	0.087	0.314	1.061	0.059	0.946	1.190	0.106	Imputed
rs12988553	2	151,511,325	G	A	0.140	0.133	0.209	1.064	0.049	0.966	1.171	0.046	Imputed
rs142230824	2	151,512,375	T	C	0.013	0.013	0.808	1.037	0.148	0.776	1.385	0.971	Imputed
rs13416697	2	151,513,311	T	C	0.089	0.083	0.320	1.061	0.060	0.944	1.194	0.191	Imputed
rs16828562	2	151,514,970	C	T	0.031	0.028	0.195	1.136	0.098	0.937	1.376	0.635	Imputed
rs11675535	2	151,515,541	A	G	0.130	0.121	0.164	1.073	0.051	0.972	1.185	0.125	Imputed
rs74769554	2	151,516,515	C	G	0.010	0.011	0.224	0.810	0.173	0.578	1.136	0.131	Imputed
rs13430342	2	151,516,631	A	C	0.089	0.083	0.323	1.061	0.060	0.944	1.193	0.190	Imputed
rs72869146	2	151,516,717	A	C	0.030	0.028	0.263	1.118	0.099	0.920	1.358	0.808	Imputed
rs6739921	2	151,516,978	C	A	0.030	0.028	0.249	1.121	0.099	0.923	1.361	0.820	Imputed
rs73970508	2	151,517,184	T	C	0.038	0.040	0.492	0.941	0.089	0.791	1.119	0.905	Imputed
rs72869148	2	151,517,268	T	C	0.030	0.028	0.263	1.118	0.099	0.920	1.358	0.808	Imputed
rs72869150	2	151,517,440	T	C	0.031	0.029	0.270	1.114	0.098	0.920	1.349	0.981	Imputed
rs72869151	2	151,517,979	G	A	0.029	0.027	0.293	1.112	0.101	0.913	1.354	0.696	Imputed
rs4260239	2	151,518,176	A	G	0.458	0.451	0.443	1.026	0.034	0.960	1.097	0.048	Imputed
rs115159009	2	151,518,880	T	C	0.015	0.011	0.024	1.375	0.142	1.041	1.814	0.447	Imputed
rs6751079	2	151,519,008	G	T	0.032	0.029	0.221	1.126	0.097	0.931	1.361	0.778	Imputed
rs117446015	2	151,519,174	A	G	0.016	0.013	0.088	1.263	0.137	0.965	1.653	0.533	Imputed
rs17269969	2	151,519,918	A	G	0.109	0.117	0.075	0.908	0.054	0.817	1.010	0.369	Imputed
rs72869154	2	151,520,512	C	T	0.030	0.027	0.235	1.126	0.100	0.926	1.368	0.519	Imputed
rs10803808	2	151,522,391	G	A	0.153	0.142	0.085	1.085	0.047	0.989	1.190	0.025	Imputed
rs6724645	2	151,522,526	C	T	0.111	0.104	0.220	1.069	0.054	0.961	1.188	0.046	Imputed
rs114873241	2	151,524,268	A	T	0.030	0.028	0.284	1.112	0.099	0.916	1.350	0.558	Imputed
rs4627564	2	151,524,286	G	C	0.108	0.102	0.326	1.056	0.055	0.948	1.176	0.037	Imputed
rs2879933	2	151,524,333	G	A	0.139	0.131	0.138	1.076	0.049	0.977	1.184	0.045	Imputed
rs78845383	2	151,524,635	C	A	0.030	0.028	0.284	1.112	0.099	0.916	1.350	0.558	Imputed
rs13389284	2	151,524,705	A	C	0.304	0.304	0.992	1.000	0.037	0.931	1.075	0.657	Imputed
rs150468669	2	151,525,344	T	G	0.014	0.014	0.977	1.004	0.145	0.756	1.334	0.407	Imputed
rs10186504	2	151,525,406	T	C	0.093	0.087	0.314	1.061	0.059	0.946	1.190	0.106	Imputed
rs13032678	2	151,525,457	G	T	0.318	0.320	0.786	0.990	0.036	0.922	1.063	0.612	Imputed
rs13027515	2	151,525,573	G	A	0.318	0.320	0.786	0.990	0.036	0.922	1.063	0.612	Imputed
rs35238738	2	151,526,725	C	T	0.049	0.054	0.197	0.904	0.078	0.776	1.054	0.791	Imputed
rs13421850	2	151,527,396	T	G	0.088	0.083	0.320	1.062	0.060	0.944	1.194	0.180	Imputed

rs13020496	2	151,528,062	G	A	0.458	0.451	0.445	1.026	0.034	0.960	1.097	0.052	Imputed
rs72869167	2	151,528,256	T	C	0.030	0.028	0.279	1.113	0.099	0.917	1.352	0.562	Imputed
chr2:151528362:I	2	151,528,362	AT	A	0.032	0.030	0.319	1.101	0.096	0.912	1.330	0.726	Imputed
chr2:151528370:D	2	151,528,370	T	TC	0.022	0.029	6.19E-03	0.734	0.113	0.588	0.917	0.526	Imputed
rs78151259	2	151,528,371	T	C	0.108	0.117	0.062	0.904	0.054	0.813	1.005	0.209	Imputed
rs74656181	2	151,528,818	A	G	0.108	0.118	0.047	0.898	0.054	0.807	0.999	0.206	Imputed
rs182190920	2	151,528,935	T	G	0.011	0.013	0.239	0.826	0.162	0.601	1.134	0.204	Imputed
rs72869169	2	151,529,642	T	C	0.030	0.028	0.279	1.113	0.099	0.917	1.352	0.562	Imputed
rs72869170	2	151,531,025	A	G	0.312	0.310	0.736	1.012	0.037	0.943	1.088	0.710	Imputed
rs11678115	2	151,531,154	C	T	0.454	0.443	0.212	1.043	0.034	0.976	1.115	0.091	Imputed
rs72869174	2	151,531,299	A	T	0.313	0.310	0.705	1.014	0.037	0.944	1.089	0.726	Imputed
rs72869176	2	151,533,203	A	G	0.032	0.030	0.293	1.106	0.096	0.917	1.336	0.695	Imputed
rs79778841	2	151,533,326	A	C	0.014	0.012	0.226	1.195	0.147	0.896	1.594	0.428	Imputed
rs1519782	2	151,533,780	A	G	0.092	0.086	0.301	1.063	0.059	0.947	1.193	0.127	Imputed
rs72869179	2	151,534,445	T	C	0.030	0.028	0.295	1.109	0.099	0.914	1.347	0.551	Imputed
rs2176746	2	151,534,813	A	G	0.421	0.417	0.791	1.009	0.034	0.944	1.079	0.293	Imputed
rs77845516	2	151,535,100	C	T	0.030	0.027	0.235	1.126	0.100	0.926	1.368	0.519	Imputed
rs10210296	2	151,535,427	T	G	0.433	0.428	0.631	1.017	0.034	0.951	1.087	0.203	Imputed
rs72869182	2	151,535,852	T	C	0.030	0.028	0.409	1.086	0.100	0.893	1.320	0.442	Imputed
rs4664722	2	151,535,966	C	T	0.442	0.435	0.446	1.026	0.034	0.960	1.097	0.205	Imputed
rs4664723	2	151,536,032	T	G	0.323	0.323	0.914	0.996	0.036	0.928	1.069	0.798	Imputed
rs72869188	2	151,536,034	A	T	0.030	0.028	0.419	1.084	0.100	0.892	1.318	0.437	Imputed
rs72869189	2	151,537,185	T	G	0.029	0.028	0.452	1.079	0.101	0.886	1.314	0.354	Imputed
rs10196763	2	151,537,652	T	C	0.088	0.082	0.355	1.057	0.060	0.940	1.189	0.192	Imputed
rs16828577	2	151,538,472	A	G	0.088	0.083	0.409	1.051	0.060	0.934	1.182	0.142	Genotyped
rs16828581	2	151,539,186	C	A	0.026	0.025	0.675	1.046	0.108	0.847	1.292	0.287	Imputed
rs72869196	2	151,539,581	A	G	0.025	0.025	0.784	1.030	0.108	0.833	1.274	0.645	Imputed
chr2:151539797:D	2	151,539,797	T	TC	0.077	0.066	0.025	1.156	0.064	1.019	1.311	0.136	Imputed
rs13428203	2	151,541,778	C	T	0.083	0.068	1.48E-03	1.219	0.062	1.079	1.378	0.155	Genotyped
rs13412670	2	151,541,987	T	C	0.082	0.067	1.97E-03	1.214	0.063	1.074	1.372	0.175	Imputed
rs151061563	2	151,542,901	C	T	0.009	0.011	0.423	0.868	0.176	0.615	1.226	0.532	Imputed
rs13028819	2	151,543,074	G	A	0.035	0.032	0.219	1.121	0.093	0.934	1.344	0.951	Imputed
rs142241793	2	151,544,094	C	T	0.009	0.011	0.423	0.868	0.176	0.615	1.226	0.532	Imputed
rs17269982	2	151,544,436	C	T	0.014	0.012	0.340	1.148	0.144	0.866	1.522	0.552	Imputed
rs11678094	2	151,546,220	T	A	0.133	0.119	0.020	1.125	0.050	1.019	1.242	0.107	Imputed
chr2:151546409:D	2	151,546,409	G	GA	0.082	0.068	2.36E-03	1.210	0.063	1.070	1.367	0.165	Imputed
rs1519786	2	151,546,612	G	T	0.082	0.068	2.56E-03	1.207	0.062	1.068	1.364	0.183	Genotyped
rs75086901	2	151,547,155	T	G	0.011	0.012	0.658	0.933	0.158	0.684	1.272	0.267	Imputed
rs13383661	2	151,548,310	T	C	0.083	0.068	2.22E-03	1.210	0.062	1.071	1.368	0.137	Imputed
rs1533664	2	151,548,341	A	G	0.114	0.099	3.86E-03	1.168	0.054	1.051	1.298	0.070	Genotyped

rs75180090	2	151,548,888	A	G	0.012	0.015	0.094	0.777	0.152	0.577	1.046	0.092	Imputed
rs77158359	2	151,549,071	C	G	0.083	0.069	3.20E-03	1.201	0.062	1.063	1.357	0.169	Imputed
rs80258907	2	151,549,355	G	A	0.083	0.069	2.58E-03	1.206	0.062	1.068	1.362	0.169	Imputed
rs6731904	2	151,549,650	T	C	0.029	0.029	0.720	1.037	0.100	0.852	1.262	0.204	Imputed
chr2:151549854:I	2	151,549,854	TC	T	0.082	0.068	3.03E-03	1.204	0.063	1.065	1.361	0.152	Imputed
rs79567008	2	151,549,880	G	C	0.082	0.068	2.30E-03	1.210	0.063	1.071	1.368	0.167	Imputed
rs9973727	2	151,550,148	A	G	0.085	0.071	2.87E-03	1.202	0.062	1.065	1.356	0.072	Imputed
rs78281835	2	151,550,585	A	G	0.082	0.068	3.03E-03	1.204	0.063	1.065	1.361	0.152	Imputed
rs17197786	2	151,551,289	G	T	0.082	0.068	2.96E-03	1.204	0.063	1.065	1.361	0.153	Imputed
rs9973439	2	151,552,330	T	A	0.082	0.068	3.31E-03	1.202	0.063	1.063	1.359	0.148	Imputed
chr2:151552389:I	2	151,552,389	AG	A	0.083	0.068	2.11E-03	1.212	0.062	1.072	1.369	0.139	Imputed
rs183211079	2	151,553,344	G	A	0.030	0.028	0.461	1.076	0.099	0.886	1.307	0.435	Imputed
rs143398155	2	151,553,520	T	A	0.078	0.063	1.29E-03	1.230	0.064	1.084	1.394	0.128	Imputed
rs10497070	2	151,553,998	C	T	0.082	0.068	3.31E-03	1.202	0.063	1.063	1.359	0.148	Imputed
rs28366677	2	151,554,230	T	G	0.082	0.068	2.52E-03	1.208	0.063	1.069	1.366	0.162	Imputed
rs13389304	2	151,554,838	T	C	0.083	0.069	3.85E-03	1.197	0.062	1.060	1.353	0.130	Imputed
rs13392657	2	151,555,634	G	C	0.082	0.068	3.31E-03	1.202	0.063	1.063	1.359	0.148	Imputed
rs13417351	2	151,555,672	C	G	0.082	0.068	3.31E-03	1.202	0.063	1.063	1.359	0.148	Imputed
rs13408495	2	151,555,677	A	T	0.082	0.068	3.31E-03	1.202	0.063	1.063	1.359	0.148	Imputed
rs6719875	2	151,555,697	T	C	0.099	0.106	0.153	0.923	0.056	0.826	1.030	0.261	Imputed
rs13405881	2	151,555,776	G	A	0.082	0.068	2.59E-03	1.208	0.063	1.068	1.366	0.161	Imputed
rs13393062	2	151,555,990	A	C	0.082	0.068	2.59E-03	1.208	0.063	1.068	1.366	0.161	Imputed
rs13393170	2	151,556,108	T	C	0.082	0.068	2.59E-03	1.208	0.063	1.068	1.366	0.161	Imputed
rs13412348	2	151,556,877	C	T	0.083	0.069	2.34E-03	1.208	0.062	1.070	1.365	0.199	Genotyped
rs6752015	2	151,557,098	A	G	0.083	0.068	2.10E-03	1.211	0.062	1.072	1.368	0.182	Imputed
rs13424938	2	151,557,992	C	G	0.083	0.068	2.10E-03	1.211	0.062	1.072	1.368	0.182	Imputed
rs13416663	2	151,558,520	C	T	0.083	0.068	2.10E-03	1.211	0.062	1.072	1.368	0.182	Imputed
rs13400803	2	151,558,526	T	C	0.082	0.068	2.59E-03	1.208	0.063	1.068	1.366	0.161	Imputed
rs13428240	2	151,558,689	T	G	0.082	0.068	2.59E-03	1.208	0.063	1.068	1.366	0.161	Imputed
rs13428359	2	151,558,803	T	G	0.082	0.068	2.59E-03	1.208	0.063	1.068	1.366	0.161	Imputed
rs13404038	2	151,559,187	G	C	0.082	0.068	2.59E-03	1.208	0.063	1.068	1.366	0.161	Imputed
rs113855767	2	151,559,834	G	A	0.082	0.068	2.36E-03	1.210	0.063	1.070	1.367	0.165	Imputed
rs13423164	2	151,559,925	C	T	0.082	0.068	2.18E-03	1.211	0.063	1.072	1.369	0.169	Imputed
rs192947668	2	151,560,166	A	G	0.011	0.012	0.658	0.933	0.158	0.684	1.272	0.267	Imputed
rs77027834	2	151,561,323	C	T	0.082	0.068	2.42E-03	1.209	0.063	1.070	1.367	0.164	Imputed
rs114576841	2	151,561,358	A	C	0.015	0.017	0.263	0.854	0.140	0.649	1.125	0.306	Imputed
chr2:151561735:I	2	151,561,735	CG	C	0.082	0.068	2.42E-03	1.209	0.063	1.070	1.367	0.164	Imputed
rs111381381	2	151,561,852	G	T	0.082	0.068	2.42E-03	1.209	0.063	1.070	1.367	0.164	Imputed
rs111652273	2	151,561,862	A	G	0.082	0.068	2.42E-03	1.209	0.063	1.070	1.367	0.164	Imputed
rs10189732	2	151,562,517	T	A	0.084	0.069	1.96E-03	1.212	0.062	1.073	1.369	0.185	Imputed

rs10192227	2	151,562,559	G	T	0.082	0.068	2.42E-03	1.209	0.063	1.070	1.367	0.164	Imputed
rs13412013	2	151,562,755	T	A	0.082	0.068	2.42E-03	1.209	0.063	1.070	1.367	0.164	Imputed
rs12104957	2	151,563,053	C	T	0.030	0.030	0.699	1.039	0.099	0.856	1.262	0.373	Imputed
rs75662477	2	151,563,140	C	A	0.032	0.037	0.096	0.853	0.096	0.707	1.029	0.887	Imputed
rs13423981	2	151,563,163	T	G	0.082	0.068	2.42E-03	1.209	0.063	1.070	1.367	0.164	Imputed
rs4664735	2	151,563,298	T	C	0.115	0.099	3.47E-03	1.170	0.054	1.053	1.300	0.083	Imputed
rs79325680	2	151,563,676	T	G	0.082	0.068	2.42E-03	1.209	0.063	1.070	1.367	0.164	Imputed
rs10196033	2	151,564,209	G	A	0.083	0.068	1.87E-03	1.214	0.062	1.074	1.372	0.165	Genotyped
rs10206835	2	151,564,372	T	G	0.082	0.068	2.30E-03	1.210	0.063	1.071	1.368	0.167	Imputed
rs147762970	2	151,564,395	T	C	0.021	0.021	0.888	1.017	0.117	0.808	1.280	0.071	Imputed
rs13430876	2	151,564,804	A	G	0.082	0.068	2.65E-03	1.207	0.063	1.068	1.365	0.159	Imputed
rs145406943	2	151,565,004	A	G	0.010	0.011	0.814	0.961	0.170	0.688	1.341	0.741	Imputed
rs114660867	2	151,565,008	A	G	0.021	0.018	0.196	1.166	0.120	0.923	1.474	0.497	Imputed
rs186157863	2	151,565,155	C	T	0.013	0.010	0.119	1.264	0.151	0.940	1.698	0.464	Imputed
rs72869202	2	151,565,880	C	G	0.028	0.031	0.400	0.919	0.101	0.754	1.120	0.161	Imputed
rs80039764	2	151,566,553	G	A	0.082	0.068	2.42E-03	1.209	0.063	1.070	1.367	0.164	Imputed
rs75456517	2	151,567,508	A	G	0.082	0.068	2.42E-03	1.209	0.063	1.070	1.367	0.164	Imputed
rs74541455	2	151,567,554	G	C	0.082	0.068	2.42E-03	1.209	0.063	1.070	1.367	0.164	Imputed
chr2:151567641:D	2	151,567,641	A	ACT	0.052	0.056	0.282	0.922	0.075	0.796	1.069	0.041	Imputed
rs75887023	2	151,567,818	G	A	0.082	0.068	2.30E-03	1.210	0.063	1.071	1.368	0.167	Imputed
rs185813798	2	151,567,910	G	C	0.011	0.012	0.658	0.933	0.158	0.684	1.272	0.267	Imputed
rs78425019	2	151,567,966	A	C	0.082	0.068	2.42E-03	1.209	0.063	1.070	1.367	0.164	Imputed
rs76781073	2	151,568,001	C	T	0.099	0.107	0.122	0.917	0.056	0.821	1.024	0.302	Imputed
rs4664737	2	151,569,097	T	G	0.030	0.030	0.775	1.029	0.099	0.847	1.250	0.346	Imputed
rs74942152	2	151,569,383	C	A	0.082	0.068	2.30E-03	1.210	0.063	1.071	1.368	0.156	Imputed
rs10170967	2	151,569,849	G	T	0.083	0.068	1.67E-03	1.217	0.062	1.077	1.375	0.140	Imputed
rs10171149	2	151,570,004	C	T	0.081	0.066	2.09E-03	1.214	0.063	1.073	1.374	0.152	Imputed
rs10168596	2	151,570,075	G	A	0.081	0.066	2.09E-03	1.214	0.063	1.073	1.374	0.152	Imputed
rs10179438	2	151,570,086	A	G	0.085	0.071	4.60E-03	1.191	0.062	1.055	1.343	0.168	Imputed
rs10179443	2	151,570,097	A	G	0.081	0.066	2.09E-03	1.214	0.063	1.073	1.374	0.152	Imputed
rs10203897	2	151,570,279	T	C	0.082	0.067	1.64E-03	1.218	0.063	1.077	1.377	0.173	Imputed
rs72870904	2	151,570,932	A	T	0.025	0.025	0.764	1.033	0.108	0.836	1.276	0.512	Imputed
rs148304943	2	151,570,981	A	G	0.012	0.012	0.920	0.984	0.156	0.725	1.337	0.916	Imputed
rs4531926	2	151,571,109	G	C	0.083	0.068	1.76E-03	1.215	0.062	1.076	1.373	0.246	Imputed
rs4531927	2	151,571,137	T	C	0.083	0.068	1.76E-03	1.215	0.062	1.076	1.373	0.246	Imputed
rs77182635	2	151,571,329	C	T	0.083	0.068	1.76E-03	1.215	0.062	1.076	1.373	0.246	Imputed
rs10201861	2	151,571,420	G	A	0.083	0.068	1.76E-03	1.215	0.062	1.076	1.373	0.246	Imputed
rs10165365	2	151,571,431	A	G	0.083	0.068	1.76E-03	1.215	0.062	1.076	1.373	0.246	Imputed
rs10207733	2	151,572,439	C	T	0.084	0.069	1.84E-03	1.213	0.062	1.074	1.369	0.255	Imputed
rs10207836	2	151,572,529	C	T	0.084	0.069	1.84E-03	1.213	0.062	1.074	1.369	0.255	Imputed

rs10205298	2	151,572,562	C	A	0.084	0.069	1.84E-03	1.213	0.062	1.074	1.369	0.255	Imputed
rs1519787	2	151,572,662	T	A	0.030	0.026	0.118	1.169	0.100	0.961	1.421	0.870	Imputed
rs10210226	2	151,572,677	C	T	0.084	0.069	1.41E-03	1.219	0.062	1.080	1.377	0.274	Imputed
rs10171399	2	151,572,717	A	G	0.084	0.069	1.84E-03	1.213	0.062	1.074	1.369	0.255	Imputed
rs10171405	2	151,572,748	A	G	0.084	0.069	1.89E-03	1.212	0.062	1.074	1.369	0.253	Imputed
rs1519788	2	151,572,877	G	A	0.030	0.026	0.118	1.169	0.100	0.961	1.421	0.870	Imputed
rs10171712	2	151,573,072	A	G	0.084	0.069	1.84E-03	1.213	0.062	1.074	1.369	0.255	Imputed
rs10171791	2	151,573,105	A	G	0.084	0.069	1.84E-03	1.213	0.062	1.074	1.369	0.255	Imputed
rs10196249	2	151,573,195	T	C	0.084	0.069	1.84E-03	1.213	0.062	1.074	1.369	0.255	Imputed
rs10198986	2	151,573,657	T	C	0.084	0.069	1.84E-03	1.213	0.062	1.074	1.369	0.255	Imputed
rs116544557	2	151,573,840	A	G	0.034	0.038	0.202	0.888	0.093	0.740	1.065	0.287	Imputed
rs10174975	2	151,573,886	A	G	0.084	0.069	1.84E-03	1.213	0.062	1.074	1.369	0.255	Imputed
rs10199258	2	151,573,928	T	C	0.067	0.055	6.93E-03	1.204	0.069	1.052	1.378	0.188	Imputed
rs4109146	2	151,573,944	G	A	0.068	0.056	7.25E-03	1.202	0.068	1.051	1.374	0.226	Imputed
rs979072	2	151,574,168	T	G	0.084	0.069	1.84E-03	1.213	0.062	1.074	1.369	0.255	Imputed
rs979071	2	151,574,207	A	C	0.084	0.069	1.84E-03	1.213	0.062	1.074	1.369	0.255	Imputed
rs6721317	2	151,574,522	A	G	0.099	0.107	0.102	0.912	0.056	0.817	1.018	0.276	Genotyped
rs77236751	2	151,574,637	C	T	0.084	0.069	1.84E-03	1.213	0.062	1.074	1.369	0.255	Imputed
rs10202449	2	151,574,792	T	C	0.084	0.069	1.84E-03	1.213	0.062	1.074	1.369	0.255	Imputed
rs10167330	2	151,574,797	C	A	0.084	0.069	1.84E-03	1.213	0.062	1.074	1.369	0.255	Imputed
rs10167359	2	151,574,863	C	A	0.084	0.069	1.84E-03	1.213	0.062	1.074	1.369	0.255	Imputed
rs7565938	2	151,575,062	T	G	0.116	0.100	3.75E-03	1.168	0.053	1.052	1.297	0.142	Imputed
rs13397197	2	151,575,102	A	G	0.084	0.069	1.84E-03	1.213	0.062	1.074	1.369	0.255	Imputed
rs13388399	2	151,575,144	C	T	0.084	0.069	1.84E-03	1.213	0.062	1.074	1.369	0.255	Imputed
rs10181281	2	151,575,619	T	G	0.084	0.069	2.13E-03	1.210	0.062	1.071	1.366	0.245	Imputed
rs13427145	2	151,576,280	T	C	0.084	0.070	2.46E-03	1.206	0.062	1.068	1.361	0.279	Imputed
rs13395042	2	151,576,623	C	T	0.085	0.070	1.97E-03	1.210	0.061	1.072	1.365	0.253	Imputed
rs13430341	2	151,576,800	A	C	0.085	0.070	1.91E-03	1.210	0.061	1.073	1.365	0.255	Imputed
rs13392640	2	151,576,892	G	A	0.085	0.070	1.75E-03	1.212	0.061	1.075	1.367	0.226	Imputed
rs10177278	2	151,577,399	C	T	0.086	0.071	1.42E-03	1.216	0.061	1.078	1.371	0.199	Imputed
rs10188346	2	151,577,898	T	G	0.086	0.071	1.45E-03	1.216	0.061	1.078	1.371	0.197	Imputed
rs10177779	2	151,578,083	G	A	0.086	0.071	1.45E-03	1.216	0.061	1.078	1.371	0.197	Imputed
rs10178005	2	151,578,352	G	A	0.086	0.071	1.74E-03	1.212	0.061	1.075	1.366	0.199	Imputed
rs13408465	2	151,578,503	A	G	0.086	0.071	1.74E-03	1.212	0.061	1.075	1.366	0.199	Imputed
chr2:151579204:I	2	151,579,204	GC	G	0.086	0.071	1.78E-03	1.211	0.061	1.074	1.366	0.198	Imputed
rs7578593	2	151,579,482	A	G	0.029	0.030	0.932	1.009	0.100	0.829	1.227	0.423	Imputed
rs13387439	2	151,579,541	T	C	0.086	0.071	1.78E-03	1.211	0.061	1.074	1.366	0.198	Imputed
rs13390009	2	151,579,657	T	C	0.086	0.071	1.78E-03	1.211	0.061	1.074	1.366	0.198	Imputed
rs13390635	2	151,580,246	T	C	0.087	0.072	2.10E-03	1.206	0.061	1.070	1.359	0.210	Imputed
rs10195471	2	151,580,445	T	G	0.086	0.071	1.78E-03	1.211	0.061	1.074	1.366	0.198	Imputed

rs144664707	2	151,580,533	A	C	0.019	0.020	0.698	0.953	0.124	0.747	1.216	0.108	Imputed
rs10180303	2	151,580,628	A	G	0.086	0.071	1.78E-03	1.211	0.061	1.074	1.366	0.198	Imputed
chr2:151581398:I	2	151,581,398	AAAGG	A	0.042	0.040	0.513	1.057	0.085	0.896	1.248	0.328	Imputed
rs13400098	2	151,581,419	A	G	0.095	0.079	1.53E-03	1.204	0.059	1.073	1.351	0.213	Imputed
rs10208473	2	151,582,136	A	C	0.086	0.071	1.83E-03	1.211	0.061	1.074	1.365	0.196	Imputed
rs7572547	2	151,582,323	C	T	0.086	0.071	1.72E-03	1.211	0.061	1.075	1.365	0.175	Imputed
rs7581925	2	151,582,400	A	G	0.086	0.071	1.72E-03	1.211	0.061	1.075	1.365	0.175	Imputed
rs187295546	2	151,583,418	T	C	0.012	0.012	0.946	0.990	0.156	0.729	1.344	0.930	Imputed
rs71415105	2	151,583,556	C	G	0.038	0.034	0.162	1.132	0.089	0.951	1.347	0.997	Imputed
rs13401296	2	151,583,697	C	T	0.086	0.071	1.81E-03	1.210	0.061	1.074	1.364	0.172	Imputed
rs2139425	2	151,584,332	C	A	0.388	0.389	0.879	1.005	0.035	0.939	1.076	0.894	Genotyped
rs148683203	2	151,585,335	T	C	0.017	0.013	0.143	1.216	0.134	0.935	1.583	0.595	Imputed
rs144122621	2	151,586,302	C	T	0.012	0.012	0.975	1.005	0.156	0.740	1.365	0.975	Imputed
rs13396101	2	151,586,663	T	C	0.086	0.071	1.69E-03	1.212	0.061	1.075	1.367	0.188	Imputed
rs13409620	2	151,586,932	T	A	0.086	0.071	1.72E-03	1.211	0.061	1.075	1.365	0.164	Imputed
rs79917138	2	151,587,943	T	C	0.036	0.039	0.317	0.913	0.091	0.764	1.091	0.167	Imputed
rs72870913	2	151,588,066	C	T	0.025	0.025	0.993	0.999	0.108	0.808	1.235	0.529	Imputed
rs13400288	2	151,588,181	T	C	0.087	0.072	2.48E-03	1.203	0.061	1.067	1.355	0.188	Genotyped
rs115503998	2	151,588,661	G	C	0.012	0.012	0.935	0.987	0.158	0.725	1.345	0.357	Imputed
rs6732513	2	151,589,173	T	C	0.086	0.071	1.72E-03	1.211	0.061	1.075	1.365	0.164	Imputed
rs13431837	2	151,589,876	A	G	0.087	0.071	1.31E-03	1.217	0.061	1.080	1.372	0.178	Imputed
rs79135668	2	151,590,670	G	A	0.013	0.016	0.176	0.823	0.145	0.619	1.094	0.165	Imputed
rs7594930	2	151,590,735	C	G	0.086	0.071	1.62E-03	1.213	0.061	1.076	1.367	0.157	Imputed
chr2:151591408:D	2	151,591,408	C	CTTTA	0.086	0.071	1.68E-03	1.212	0.061	1.075	1.366	0.165	Imputed
rs13402125	2	151,591,772	A	G	0.086	0.071	1.68E-03	1.212	0.061	1.075	1.366	0.165	Imputed
rs10207475	2	151,591,927	T	C	0.086	0.071	1.68E-03	1.212	0.061	1.075	1.366	0.165	Imputed
rs16828597	2	151,592,314	A	G	0.086	0.071	1.68E-03	1.212	0.061	1.075	1.366	0.165	Imputed
chr2:151592785:D	2	151,592,785	G	GGAA	0.335	0.346	0.138	0.948	0.036	0.884	1.017	0.976	Imputed
chr2:151592787:D	2	151,592,787	A	AAGG	0.335	0.346	0.138	0.948	0.036	0.884	1.017	0.976	Imputed
rs10210737	2	151,592,895	T	C	0.086	0.071	1.68E-03	1.212	0.061	1.075	1.366	0.165	Imputed
rs13398492	2	151,594,475	T	A	0.086	0.071	1.68E-03	1.212	0.061	1.075	1.366	0.165	Imputed
rs12328511	2	151,595,551	G	A	0.086	0.071	1.68E-03	1.212	0.061	1.075	1.366	0.165	Imputed
rs7601669	2	151,595,776	A	G	0.027	0.028	0.959	0.995	0.103	0.812	1.218	0.509	Imputed
chr2:151596017:D	2	151,596,017	A	AAC	0.086	0.071	1.64E-03	1.212	0.061	1.076	1.367	0.167	Imputed
chr2:151596426:D	2	151,596,426	G	GC	0.086	0.071	2.04E-03	1.208	0.061	1.071	1.362	0.189	Imputed
rs181801601	2	151,596,427	T	C	0.086	0.071	1.71E-03	1.212	0.061	1.075	1.366	0.200	Imputed
rs10176716	2	151,596,596	T	C	0.086	0.071	1.64E-03	1.212	0.061	1.076	1.367	0.167	Imputed
rs75267023	2	151,598,274	T	C	0.051	0.057	0.159	0.899	0.076	0.774	1.043	0.096	Imputed
rs13418645	2	151,598,934	C	T	0.080	0.067	6.69E-03	1.187	0.063	1.049	1.344	0.202	Imputed
rs13403254	2	151,599,332	T	C	0.080	0.067	4.60E-03	1.196	0.063	1.057	1.354	0.228	Imputed

rs13403450	2	151,599,509	T	C	0.085	0.071	1.99E-03	1.209	0.061	1.072	1.364	0.260	Imputed
rs149597024	2	151,599,920	C	T	0.012	0.011	0.883	1.024	0.158	0.751	1.396	0.899	Imputed
rs1114489	2	151,602,113	C	T	0.173	0.160	0.073	1.084	0.045	0.993	1.185	0.517	Imputed
rs1519789	2	151,603,418	C	G	0.024	0.024	0.864	0.981	0.111	0.790	1.219	0.906	Imputed
rs71415107	2	151,603,585	A	C	0.036	0.031	0.096	1.164	0.092	0.973	1.393	0.809	Imputed
rs138908055	2	151,604,023	A	G	0.081	0.067	3.00E-03	1.205	0.063	1.065	1.364	0.421	Imputed
rs2176747	2	151,604,112	G	T	0.032	0.034	0.662	0.959	0.096	0.795	1.157	0.913	Imputed
rs71415108	2	151,604,147	G	T	0.039	0.033	0.042	1.197	0.088	1.007	1.423	0.984	Imputed
rs12477523	2	151,604,422	G	A	0.024	0.025	0.845	0.979	0.111	0.788	1.216	0.943	Imputed
rs62169262	2	151,604,888	T	C	0.105	0.109	0.512	0.965	0.055	0.866	1.074	0.774	Imputed
rs77563301	2	151,605,076	C	T	0.045	0.049	0.299	0.919	0.081	0.784	1.078	0.458	Imputed
chr2:151606618:D	2	151,606,618	ATG	A	0.028	0.027	0.677	1.043	0.102	0.854	1.274	0.238	Imputed
rs112279402	2	151,606,792	G	A	0.026	0.026	0.963	1.005	0.107	0.815	1.239	0.970	Imputed
rs7582337	2	151,606,913	G	A	0.101	0.092	0.130	1.090	0.057	0.975	1.218	0.705	Imputed
rs12464731	2	151,606,958	A	G	0.232	0.209	1.00E-03	1.142	0.040	1.055	1.236	0.590	Imputed
rs12477164	2	151,607,040	A	G	0.128	0.119	0.188	1.069	0.051	0.968	1.182	0.767	Genotyped
rs62169263	2	151,607,252	C	T	0.105	0.109	0.435	0.958	0.055	0.860	1.067	0.804	Imputed
rs1401802	2	151,607,557	A	T	0.101	0.091	0.117	1.093	0.057	0.978	1.221	0.727	Imputed
rs1401804	2	151,607,683	C	T	0.101	0.092	0.133	1.089	0.057	0.974	1.217	0.703	Imputed
rs10803820	2	151,608,579	T	C	0.101	0.091	0.106	1.096	0.057	0.981	1.225	0.680	Imputed
rs10497071	2	151,608,765	G	A	0.315	0.317	0.861	0.994	0.036	0.925	1.067	0.707	Genotyped
rs1028218	2	151,609,009	C	G	0.101	0.091	0.113	1.094	0.057	0.979	1.223	0.735	Imputed
rs1028217	2	151,609,160	G	A	0.101	0.091	0.108	1.095	0.057	0.980	1.224	0.676	Imputed
rs79696081	2	151,609,368	A	T	0.078	0.066	8.60E-03	1.183	0.064	1.044	1.340	0.215	Imputed
rs1028216	2	151,609,422	T	C	0.101	0.091	0.109	1.095	0.057	0.980	1.224	0.673	Imputed
rs72870937	2	151,609,552	G	C	0.013	0.014	0.572	0.919	0.150	0.685	1.233	0.166	Imputed
rs2013081	2	151,609,681	G	A	0.101	0.091	0.111	1.095	0.057	0.980	1.223	0.738	Imputed
rs7568086	2	151,610,086	C	G	0.101	0.091	0.096	1.099	0.057	0.983	1.228	0.703	Imputed
rs1401805	2	151,610,824	C	T	0.101	0.091	0.110	1.095	0.057	0.980	1.223	0.718	Imputed
rs13036128	2	151,611,297	A	G	0.023	0.021	0.483	1.083	0.113	0.867	1.353	0.869	Imputed
rs1519790	2	151,611,392	G	A	0.101	0.092	0.130	1.089	0.057	0.975	1.217	0.736	Genotyped
rs1519791	2	151,611,523	A	G	0.101	0.091	0.109	1.095	0.057	0.980	1.224	0.715	Imputed
rs10191762	2	151,611,945	T	C	0.100	0.090	0.098	1.099	0.057	0.983	1.228	0.604	Imputed
rs4664744	2	151,612,462	C	T	0.126	0.118	0.192	1.069	0.051	0.967	1.182	0.727	Imputed
rs78324558	2	151,612,661	G	A	0.025	0.025	0.876	0.983	0.109	0.794	1.218	0.776	Imputed
rs13000349	2	151,613,331	G	A	0.101	0.091	0.124	1.091	0.057	0.976	1.219	0.756	Imputed
rs72870941	2	151,613,526	A	G	0.025	0.025	0.876	0.983	0.109	0.794	1.218	0.776	Imputed
rs925986	2	151,613,569	A	G	0.101	0.091	0.124	1.091	0.057	0.976	1.219	0.756	Imputed
rs969912	2	151,614,091	C	T	0.101	0.092	0.137	1.088	0.057	0.974	1.216	0.733	Imputed
rs6727272	2	151,614,470	A	G	0.101	0.091	0.124	1.091	0.057	0.976	1.219	0.756	Imputed

rs6730489	2	151,614,603	C	T	0.309	0.312	0.700	0.986	0.037	0.918	1.059	0.746	Imputed
rs4664745	2	151,614,824	A	G	0.122	0.115	0.321	1.053	0.052	0.951	1.166	0.527	Imputed
rs78515346	2	151,614,870	G	T	0.099	0.090	0.123	1.092	0.057	0.977	1.221	0.677	Imputed
rs12477261	2	151,616,147	C	G	0.097	0.085	0.029	1.134	0.058	1.013	1.270	0.827	Imputed
rs114082831	2	151,616,375	T	C	0.056	0.061	0.305	0.928	0.073	0.804	1.070	0.558	Imputed
rs1401797	2	151,617,259	C	T	0.450	0.406	1.01E-07	1.199	0.034	1.121	1.281	0.942	Imputed
rs11893757	2	151,617,608	G	A	0.450	0.405	1.28E-07	1.197	0.034	1.120	1.280	0.981	Imputed
rs62169264	2	151,618,234	A	G	0.103	0.110	0.246	0.938	0.055	0.841	1.045	0.977	Imputed
rs12987270	2	151,618,286	C	G	0.120	0.110	0.097	1.091	0.052	0.984	1.208	0.746	Imputed
rs79379780	2	151,618,346	G	A	0.013	0.015	0.188	0.823	0.147	0.617	1.098	0.099	Imputed
rs970309	2	151,619,040	C	T	0.451	0.405	7.40E-08	1.201	0.034	1.123	1.284	0.912	Imputed
rs970308	2	151,619,179	G	A	0.230	0.249	6.86E-03	0.897	0.040	0.829	0.971	0.429	Imputed
rs115897797	2	151,619,206	T	A	0.021	0.021	0.969	1.005	0.118	0.797	1.266	0.657	Imputed
rs970307	2	151,619,345	G	A	0.120	0.111	0.109	1.088	0.052	0.982	1.205	0.788	Genotyped
rs142360909	2	151,619,390	A	C	0.027	0.025	0.291	1.115	0.104	0.910	1.367	0.169	Imputed
rs1519754	2	151,619,693	G	T	0.447	0.403	1.15E-07	1.198	0.034	1.120	1.281	0.918	Genotyped
rs2203895	2	151,620,069	G	A	0.451	0.406	1.19E-07	1.197	0.034	1.120	1.280	0.988	Imputed
rs2203896	2	151,620,073	A	C	0.452	0.407	1.06E-07	1.198	0.034	1.121	1.281	0.995	Imputed
chr2:151620651:D	2	151,620,651	AC	A	0.023	0.023	0.906	1.013	0.112	0.813	1.263	0.721	Imputed
rs11693960	2	151,621,737	T	C	0.097	0.087	0.074	1.108	0.058	0.990	1.241	0.905	Imputed
rs6740164	2	151,621,741	T	A	0.024	0.023	0.866	1.019	0.112	0.819	1.269	0.788	Imputed
rs11678576	2	151,621,827	A	T	0.097	0.087	0.077	1.107	0.058	0.989	1.239	0.945	Imputed
rs115979775	2	151,621,986	T	C	0.013	0.011	0.130	1.254	0.149	0.936	1.680	0.509	Imputed
rs59304757	2	151,622,296	T	G	0.014	0.014	0.904	0.983	0.146	0.738	1.308	0.558	Imputed
rs1519756	2	151,622,783	T	C	0.350	0.315	4.07E-06	1.178	0.036	1.099	1.263	0.892	Imputed
rs1519757	2	151,622,877	G	A	0.098	0.088	0.073	1.108	0.057	0.991	1.241	0.898	Genotyped
rs1028213	2	151,623,322	G	A	0.451	0.406	6.09E-08	1.202	0.034	1.125	1.285	0.933	Imputed
rs6734792	2	151,624,882	C	T	0.449	0.404	5.19E-08	1.204	0.034	1.126	1.287	0.986	Genotyped
rs1356738	2	151,626,425	A	T	0.325	0.290	4.39E-06	1.181	0.036	1.100	1.268	0.958	Imputed
rs78700746	2	151,626,705	C	A	0.022	0.022	0.980	1.003	0.116	0.799	1.259	0.446	Imputed
rs9789673	2	151,627,106	T	G	0.325	0.291	3.70E-06	1.183	0.036	1.101	1.270	0.988	Genotyped
rs1519758	2	151,627,175	C	T	0.350	0.314	3.72E-06	1.179	0.036	1.099	1.264	0.886	Imputed
rs955766	2	151,627,578	G	A	0.350	0.314	3.72E-06	1.179	0.036	1.099	1.264	0.886	Imputed
rs1519759	2	151,627,785	C	A	0.024	0.024	0.738	1.038	0.110	0.836	1.288	0.697	Imputed
rs1554370	2	151,628,055	G	A	0.349	0.314	4.10E-06	1.178	0.036	1.099	1.263	0.930	Imputed
rs1554371	2	151,628,292	A	G	0.349	0.314	4.10E-06	1.178	0.036	1.099	1.263	0.930	Imputed
rs1554372	2	151,628,355	G	A	0.349	0.314	4.10E-06	1.178	0.036	1.099	1.263	0.930	Imputed
rs1356739	2	151,628,393	G	A	0.349	0.314	4.10E-06	1.178	0.036	1.099	1.263	0.930	Imputed
rs1356740	2	151,628,411	A	T	0.349	0.314	4.10E-06	1.178	0.036	1.099	1.263	0.930	Imputed
rs6705092	2	151,628,594	A	T	0.446	0.401	7.75E-08	1.201	0.034	1.123	1.284	0.863	Imputed

rs74699282	2	151,628,836	G	A	0.014	0.014	0.958	0.993	0.145	0.748	1.318	0.667	Imputed
rs6751028	2	151,629,004	A	C	0.325	0.291	3.71E-06	1.183	0.036	1.101	1.270	0.988	Imputed
rs143397748	2	151,629,071	C	T	0.013	0.015	0.188	0.823	0.147	0.617	1.098	0.099	Imputed
rs6711779	2	151,629,101	T	A	0.024	0.024	0.786	1.031	0.111	0.830	1.280	0.830	Imputed
rs13033404	2	151,629,804	A	C	0.326	0.291	3.52E-06	1.183	0.036	1.102	1.270	0.992	Imputed
rs77687468	2	151,630,168	C	T	0.013	0.011	0.135	1.250	0.149	0.933	1.675	0.503	Imputed
rs1519760	2	151,630,293	G	T	0.097	0.087	0.082	1.105	0.058	0.988	1.238	0.909	Imputed
rs9789693	2	151,630,325	C	T	0.326	0.291	3.45E-06	1.183	0.036	1.102	1.270	0.990	Imputed
rs79878469	2	151,630,554	C	A	0.013	0.013	0.980	1.004	0.151	0.747	1.349	0.067	Imputed
rs1607618	2	151,631,355	C	T	0.447	0.401	5.99E-08	1.203	0.034	1.125	1.286	0.897	Imputed
rs75090794	2	151,632,031	A	G	0.025	0.028	0.362	0.907	0.108	0.734	1.119	0.801	Imputed
rs6716544	2	151,633,079	G	A	0.453	0.407	8.17E-08	1.200	0.034	1.123	1.283	0.814	Imputed
rs1850878	2	151,633,093	T	C	0.450	0.405	6.17E-08	1.202	0.034	1.125	1.285	0.956	Imputed
chr2:151633111:D	2	151,633,111	AAC	A	0.465	0.420	1.02E-07	1.198	0.034	1.121	1.281	0.959	Imputed
rs1519761	2	151,633,204	G	A	0.447	0.401	7.25E-08	1.201	0.034	1.124	1.284	0.868	Genotyped
rs11900824	2	151,633,520	G	A	0.447	0.401	6.67E-08	1.202	0.034	1.124	1.285	0.874	Imputed
rs11900828	2	151,633,543	G	A	0.449	0.404	6.97E-08	1.201	0.034	1.124	1.284	0.965	Imputed
rs7561985	2	151,634,130	T	C	0.448	0.403	6.26E-08	1.202	0.034	1.125	1.285	0.999	Imputed
rs1519762	2	151,634,419	G	A	0.449	0.404	7.27E-08	1.201	0.034	1.124	1.284	0.968	Imputed
rs1519763	2	151,634,517	T	G	0.445	0.401	1.56E-07	1.196	0.034	1.118	1.278	0.960	Imputed
rs11899817	2	151,634,830	A	T	0.326	0.291	3.59E-06	1.183	0.036	1.102	1.270	0.994	Imputed
rs1879147	2	151,634,982	G	A	0.326	0.291	3.67E-06	1.183	0.036	1.101	1.270	0.995	Imputed
rs1879148	2	151,635,022	C	T	0.121	0.111	0.086	1.094	0.052	0.987	1.212	0.813	Imputed
rs6757804	2	151,635,832	C	T	0.447	0.401	4.53E-08	1.205	0.034	1.127	1.288	0.903	Genotyped
rs55996080	2	151,638,114	A	C	0.013	0.014	0.512	0.907	0.150	0.676	1.216	0.090	Imputed
rs6726969	2	151,638,164	C	T	0.272	0.247	4.47E-04	1.143	0.038	1.061	1.232	0.940	Imputed
rs12475182	2	151,638,802	C	T	0.143	0.133	0.095	1.084	0.049	0.986	1.193	0.444	Imputed
rs13384483	2	151,638,827	C	T	0.270	0.240	3.40E-05	1.172	0.038	1.087	1.263	0.934	Imputed
rs1519764	2	151,639,218	G	C	0.267	0.238	2.86E-05	1.174	0.038	1.089	1.266	0.864	Imputed
chr2:151639732:D	2	151,639,732	ATG	A	0.420	0.377	3.16E-07	1.192	0.034	1.114	1.275	0.749	Imputed
rs1519765	2	151,640,403	A	C	0.024	0.023	0.603	1.059	0.110	0.853	1.314	1.000	Imputed
rs142662237	2	151,640,897	G	A	0.011	0.015	0.052	0.738	0.157	0.543	1.004	0.897	Imputed
rs56245823	2	151,641,329	C	T	0.417	0.377	1.45E-06	1.180	0.034	1.103	1.262	0.726	Imputed
rs141212334	2	151,641,727	T	G	0.010	0.012	0.108	0.767	0.166	0.554	1.062	0.319	Imputed
rs145015566	2	151,641,728	T	A	0.010	0.012	0.108	0.767	0.166	0.554	1.062	0.319	Imputed
rs143991243	2	151,642,307	A	G	0.125	0.113	0.051	1.106	0.052	0.999	1.223	0.733	Imputed
rs148249839	2	151,642,504	T	C	0.009	0.011	0.302	0.836	0.175	0.593	1.177	0.247	Imputed
rs16828685	2	151,643,016	A	T	0.268	0.240	8.60E-05	1.163	0.038	1.078	1.253	0.968	Imputed
rs12465523	2	151,643,201	G	A	0.268	0.240	8.60E-05	1.163	0.038	1.078	1.253	0.968	Imputed
chr2:151643226:D	2	151,643,226	T	TACTCTA	0.022	0.021	0.602	1.062	0.116	0.847	1.333	0.728	Imputed

rs962052	2	151,644,203	C	T	0.287	0.260	1.48E-04	1.153	0.038	1.071	1.241	0.897	Imputed
rs12990101	2	151,644,407	C	T	0.266	0.237	7.84E-05	1.164	0.039	1.080	1.255	0.803	Imputed
rs115581255	2	151,644,454	A	G	0.012	0.012	0.962	1.007	0.154	0.745	1.362	0.100	Imputed
rs12990371	2	151,644,573	G	T	0.266	0.238	8.28E-05	1.164	0.039	1.079	1.255	0.797	Imputed
rs726578	2	151,644,711	T	G	0.417	0.374	2.85E-07	1.193	0.034	1.115	1.276	0.642	Imputed
rs60978265	2	151,644,717	A	G	0.022	0.021	0.602	1.062	0.116	0.847	1.333	0.728	Imputed
rs61090401	2	151,644,724	A	G	0.022	0.021	0.602	1.062	0.116	0.847	1.333	0.728	Imputed
rs5011637	2	151,645,050	T	G	0.290	0.261	6.34E-05	1.161	0.037	1.079	1.250	0.869	Imputed
rs76791701	2	151,645,120	C	A	0.022	0.021	0.602	1.062	0.116	0.847	1.333	0.728	Imputed
rs78177577	2	151,645,276	T	C	0.022	0.021	0.548	1.072	0.115	0.855	1.343	0.601	Imputed
chr2:151645415:I	2	151,645,415	AAATACCAGGGGGT	A	0.233	0.209	4.41E-04	1.152	0.040	1.065	1.247	0.738	Imputed
chr2:151645428:I	2	151,645,428	TA	T	0.116	0.107	0.072	1.101	0.053	0.992	1.222	0.685	Imputed
rs138675266	2	151,645,786	C	A	0.011	0.015	0.054	0.740	0.157	0.544	1.006	0.891	Imputed
rs10515926	2	151,645,865	T	C	0.414	0.374	1.11E-06	1.182	0.034	1.105	1.265	0.701	Genotyped
rs13022563	2	151,646,273	G	C	0.269	0.242	1.64E-04	1.155	0.038	1.072	1.246	0.810	Imputed
rs76953575	2	151,646,287	C	G	0.033	0.039	0.041	0.826	0.094	0.687	0.992	0.717	Imputed
rs10489978	2	151,646,494	T	G	0.125	0.113	0.053	1.105	0.052	0.999	1.222	0.728	Imputed
rs11677724	2	151,646,578	C	T	0.027	0.029	0.562	0.942	0.103	0.769	1.153	0.361	Imputed
rs71415110	2	151,646,825	T	A	0.066	0.055	7.08E-03	1.204	0.069	1.052	1.379	0.759	Imputed
rs1010330	2	151,646,872	G	C	0.275	0.245	4.19E-05	1.169	0.038	1.085	1.260	0.477	Imputed
rs1519766	2	151,647,211	A	C	0.415	0.374	9.03E-07	1.184	0.034	1.107	1.267	0.683	Imputed
rs1519767	2	151,647,402	C	G	0.106	0.094	0.048	1.116	0.055	1.001	1.244	0.997	Imputed
rs2880093	2	151,647,420	T	G	0.266	0.238	8.13E-05	1.164	0.038	1.079	1.255	0.831	Imputed
rs2158818	2	151,647,791	C	G	0.266	0.238	6.90E-05	1.165	0.038	1.081	1.257	0.792	Imputed
rs2158819	2	151,647,796	G	A	0.266	0.238	6.90E-05	1.165	0.038	1.081	1.257	0.792	Imputed
chr2:151647855:D	2	151,647,855	G	GTA	0.414	0.374	1.13E-06	1.182	0.034	1.105	1.265	0.640	Imputed
rs1519768	2	151,648,276	T	C	0.414	0.374	1.39E-06	1.181	0.034	1.104	1.263	0.793	Genotyped
rs1607619	2	151,648,499	A	G	0.415	0.374	1.04E-06	1.183	0.034	1.106	1.266	0.706	Imputed
rs995300	2	151,648,506	T	C	0.147	0.134	0.047	1.100	0.048	1.001	1.209	0.661	Imputed
rs995299	2	151,648,536	T	A	0.266	0.238	7.70E-05	1.164	0.038	1.080	1.255	0.837	Imputed
rs79202081	2	151,648,984	A	C	0.147	0.134	0.047	1.100	0.048	1.001	1.209	0.661	Imputed
rs79995340	2	151,648,993	T	C	0.147	0.134	0.047	1.100	0.048	1.001	1.209	0.661	Imputed
chr2:151649135:I	2	151,649,135	CG	C	0.022	0.021	0.638	1.056	0.116	0.842	1.324	0.757	Imputed
rs12616378	2	151,649,227	C	T	0.147	0.134	0.047	1.100	0.048	1.001	1.209	0.661	Imputed
rs6715800	2	151,649,791	C	T	0.424	0.382	4.50E-07	1.189	0.034	1.112	1.272	0.422	Imputed
rs6743419	2	151,649,843	G	A	0.275	0.245	4.09E-05	1.169	0.038	1.085	1.260	0.479	Imputed
rs2190375	2	151,650,314	G	A	0.148	0.135	0.054	1.097	0.048	0.999	1.205	0.752	Genotyped
chr2:151651579:I	2	151,651,579	GA	G	0.141	0.130	0.084	1.088	0.049	0.989	1.197	0.631	Imputed
rs7607449	2	151,651,763	C	T	0.415	0.374	8.01E-07	1.185	0.034	1.108	1.268	0.632	Imputed
chr2:151651890:I	2	151,651,890	C	CA	0.426	0.385	8.95E-07	1.183	0.034	1.106	1.266	0.381	Imputed

rs115793259	2	151,652,167	T	G	0.266	0.238	8.52E-05	1.163	0.039	1.079	1.254	0.740	Imputed
rs4449130	2	151,652,187	A	C	0.019	0.019	0.929	0.989	0.126	0.773	1.266	0.134	Imputed
rs146810369	2	151,652,520	C	A	0.147	0.134	0.046	1.101	0.048	1.002	1.210	0.666	Imputed
rs80006513	2	151,652,556	G	A	0.274	0.244	3.92E-05	1.170	0.038	1.085	1.261	0.460	Imputed
rs144614313	2	151,652,596	C	A	0.281	0.252	6.42E-05	1.163	0.038	1.080	1.253	0.454	Imputed
rs12470796	2	151,652,790	A	G	0.125	0.113	0.053	1.105	0.052	0.999	1.223	0.749	Imputed
rs7574816	2	151,652,945	T	G	0.266	0.238	7.64E-05	1.164	0.038	1.080	1.255	0.728	Imputed
rs7571986	2	151,653,018	A	G	0.268	0.240	8.22E-05	1.163	0.038	1.079	1.254	0.899	Imputed
chr2:151653372:I	2	151,653,372	ATTTTC	A	0.129	0.114	0.019	1.127	0.051	1.020	1.245	0.733	Imputed
rs6728560	2	151,653,870	T	C	0.277	0.248	3.91E-05	1.169	0.038	1.085	1.259	0.507	Genotyped
chr2:151654105:I	2	151,654,105	CT	C	0.228	0.251	1.07E-03	0.877	0.040	0.810	0.949	0.378	Imputed
rs57657407	2	151,654,157	G	A	0.422	0.379	4.16E-07	1.190	0.034	1.112	1.273	0.293	Imputed
rs12468370	2	151,654,287	T	A	0.125	0.112	0.043	1.110	0.052	1.003	1.228	0.742	Imputed
rs115419104	2	151,655,493	G	A	0.017	0.015	0.425	1.111	0.132	0.857	1.440	0.514	Imputed
rs7580162	2	151,655,513	G	A	0.278	0.248	3.96E-05	1.169	0.038	1.085	1.259	0.949	Genotyped
rs7569269	2	151,655,699	T	C	0.087	0.078	0.033	1.138	0.060	1.011	1.281	0.848	Imputed
rs12466447	2	151,656,140	T	C	0.135	0.119	3.60E-03	1.156	0.050	1.049	1.275	0.489	Imputed
rs12466545	2	151,656,386	T	C	0.103	0.091	0.010	1.155	0.056	1.035	1.289	0.557	Imputed
rs80023863	2	151,657,080	C	T	0.033	0.034	0.780	0.974	0.094	0.809	1.172	0.381	Imputed
rs147635575	2	151,657,569	T	C	0.017	0.015	0.430	1.110	0.132	0.857	1.439	0.700	Imputed
rs6744277	2	151,657,704	T	C	0.156	0.142	0.011	1.127	0.047	1.028	1.235	0.739	Imputed
rs6726932	2	151,657,955	C	T	0.166	0.148	2.12E-03	1.151	0.046	1.052	1.259	0.820	Imputed
rs10180526	2	151,658,348	G	C	0.130	0.116	9.55E-03	1.140	0.051	1.033	1.259	0.419	Imputed
rs6758550	2	151,658,789	G	A	0.134	0.119	5.68E-03	1.149	0.050	1.041	1.267	0.595	Imputed
rs60649510	2	151,659,983	C	G	0.253	0.272	9.71E-03	0.904	0.039	0.838	0.976	0.176	Imputed
rs7584633	2	151,659,991	C	A	0.135	0.119	4.12E-03	1.154	0.050	1.046	1.273	0.588	Imputed
rs138278180	2	151,660,043	A	G	0.028	0.025	0.164	1.153	0.103	0.943	1.411	0.274	Imputed
rs13034292	2	151,660,356	T	C	0.270	0.246	8.26E-04	1.137	0.038	1.054	1.225	0.923	Imputed
rs1155617	2	151,660,436	C	T	0.135	0.119	4.36E-03	1.153	0.050	1.046	1.272	0.601	Imputed
rs13033752	2	151,660,727	T	C	0.135	0.119	4.36E-03	1.153	0.050	1.046	1.272	0.601	Imputed
rs6717780	2	151,661,037	C	G	0.135	0.119	4.28E-03	1.153	0.050	1.046	1.272	0.602	Imputed
rs13029146	2	151,661,103	A	G	0.135	0.119	4.28E-03	1.153	0.050	1.046	1.272	0.602	Imputed
rs1519769	2	151,661,321	T	A	0.135	0.119	4.36E-03	1.153	0.050	1.046	1.272	0.601	Imputed
rs957744	2	151,662,271	G	C	0.132	0.116	4.24E-03	1.155	0.050	1.046	1.275	0.412	Imputed
rs1519770	2	151,662,590	A	G	0.135	0.120	6.92E-03	1.144	0.050	1.038	1.262	0.400	Imputed
rs6432985	2	151,663,009	C	T	0.132	0.116	4.24E-03	1.155	0.050	1.046	1.275	0.412	Imputed
rs4141070	2	151,663,105	T	C	0.135	0.119	4.96E-03	1.151	0.050	1.043	1.269	0.625	Genotyped
rs4141071	2	151,663,340	C	G	0.135	0.119	4.20E-03	1.154	0.050	1.046	1.272	0.605	Imputed
rs13411622	2	151,663,394	G	A	0.104	0.092	0.014	1.148	0.056	1.029	1.281	0.555	Imputed
rs12473533	2	151,663,457	T	C	0.135	0.119	4.61E-03	1.152	0.050	1.045	1.270	0.613	Imputed

chr2:151663682:D	2	151,663,682	T	TA	0.223	0.239	0.020	0.910	0.041	0.841	0.986	0.110	Imputed
rs6747106	2	151,663,736	T	C	0.132	0.116	4.26E-03	1.155	0.050	1.046	1.275	0.411	Imputed
rs1949524	2	151,664,530	G	A	0.134	0.119	4.28E-03	1.154	0.050	1.046	1.272	0.628	Imputed
rs77781660	2	151,665,485	A	G	0.025	0.029	0.176	0.864	0.107	0.700	1.067	0.013	Imputed
rs886769	2	151,667,121	A	G	0.136	0.120	5.76E-03	1.147	0.050	1.041	1.265	0.798	Imputed
rs1519772	2	151,667,708	T	C	0.242	0.263	3.50E-03	0.891	0.039	0.825	0.963	0.348	Imputed
rs1519773	2	151,667,905	G	T	0.133	0.118	4.53E-03	1.153	0.050	1.045	1.273	0.633	Imputed
rs1519774	2	151,668,609	T	C	0.136	0.120	5.56E-03	1.148	0.050	1.041	1.266	0.803	Imputed
rs75166170	2	151,669,182	C	T	0.031	0.029	0.331	1.099	0.097	0.908	1.330	0.292	Imputed
rs1519775	2	151,669,375	G	A	0.135	0.120	7.60E-03	1.142	0.050	1.036	1.260	0.762	Genotyped
rs6753674	2	151,669,661	C	T	0.135	0.120	5.75E-03	1.147	0.050	1.041	1.265	0.848	Genotyped
rs2190374	2	151,669,844	G	A	0.136	0.121	6.42E-03	1.145	0.050	1.039	1.262	0.919	Imputed
rs1949525	2	151,669,892	T	G	0.133	0.118	4.78E-03	1.152	0.050	1.044	1.271	0.647	Imputed
rs1519776	2	151,670,883	C	G	0.133	0.118	5.27E-03	1.150	0.050	1.043	1.269	0.659	Imputed
rs79024333	2	151,671,888	C	T	0.084	0.088	0.447	0.955	0.061	0.848	1.076	0.430	Imputed
rs4664762	2	151,672,484	G	A	0.133	0.118	5.46E-03	1.150	0.050	1.042	1.269	0.654	Imputed
rs62169310	2	151,673,048	A	G	0.044	0.048	0.281	0.916	0.082	0.780	1.075	0.181	Imputed
rs13031920	2	151,673,143	T	C	0.133	0.118	4.72E-03	1.152	0.050	1.044	1.272	0.631	Imputed
rs13000995	2	151,673,158	C	G	0.133	0.118	4.72E-03	1.152	0.050	1.044	1.272	0.631	Imputed
rs6705775	2	151,673,530	T	C	0.133	0.117	4.36E-03	1.154	0.050	1.046	1.273	0.638	Imputed
rs6716109	2	151,673,599	G	A	0.131	0.116	4.55E-03	1.154	0.050	1.045	1.274	0.560	Imputed
rs1401798	2	151,673,871	G	T	0.136	0.121	4.92E-03	1.150	0.050	1.043	1.268	0.796	Imputed
rs1401799	2	151,673,939	C	T	0.136	0.120	5.08E-03	1.150	0.050	1.043	1.268	0.771	Imputed
rs1401800	2	151,673,960	C	T	0.133	0.118	4.61E-03	1.153	0.050	1.045	1.272	0.631	Genotyped
rs10173993	2	151,674,078	A	G	0.131	0.116	4.46E-03	1.154	0.050	1.046	1.274	0.562	Imputed
rs1160182	2	151,674,593	A	C	0.133	0.119	8.76E-03	1.140	0.050	1.034	1.258	0.626	Imputed
rs1356741	2	151,674,876	G	A	0.154	0.140	0.016	1.121	0.047	1.022	1.229	0.679	Imputed
chr2:151674877:I	2	151,674,877	A	AC	0.174	0.159	8.55E-03	1.125	0.045	1.031	1.229	0.592	Imputed
rs1356742	2	151,674,877	A	C	0.153	0.139	0.017	1.120	0.047	1.021	1.229	0.654	Imputed
rs1554373	2	151,675,301	G	A	0.136	0.120	5.08E-03	1.150	0.050	1.043	1.268	0.771	Imputed
rs6432991	2	151,675,367	G	A	0.137	0.121	4.70E-03	1.150	0.050	1.044	1.268	0.838	Imputed
rs2880094	2	151,675,474	C	T	0.150	0.138	0.039	1.103	0.048	1.005	1.211	0.767	Imputed
rs4444521	2	151,675,475	A	G	0.141	0.128	0.015	1.126	0.049	1.023	1.239	0.527	Imputed
rs2341592	2	151,675,653	G	A	0.136	0.120	4.99E-03	1.150	0.050	1.043	1.268	0.773	Imputed
rs4664768	2	151,675,991	T	A	0.136	0.120	4.99E-03	1.150	0.050	1.043	1.268	0.773	Imputed
rs12998083	2	151,676,174	A	G	0.132	0.116	4.31E-03	1.155	0.050	1.046	1.275	0.505	Genotyped
rs12998238	2	151,676,199	A	G	0.136	0.120	4.99E-03	1.150	0.050	1.043	1.268	0.773	Imputed
rs6757455	2	151,676,395	C	G	0.141	0.125	3.02E-03	1.156	0.049	1.050	1.272	0.888	Imputed
rs6432992	2	151,676,774	T	C	0.136	0.121	4.74E-03	1.151	0.050	1.044	1.268	0.800	Imputed
rs1519777	2	151,677,109	C	A	0.133	0.117	4.36E-03	1.154	0.050	1.046	1.273	0.638	Imputed

rs4664176	2	151,677,329	T	C	0.137	0.122	8.35E-03	1.140	0.050	1.034	1.256	0.804	Imputed
rs11679193	2	151,677,425	T	A	0.141	0.125	3.02E-03	1.156	0.049	1.050	1.272	0.888	Imputed
rs11683080	2	151,677,478	G	A	0.136	0.121	5.87E-03	1.147	0.050	1.040	1.264	0.816	Genotyped
rs12463537	2	151,678,529	T	C	0.133	0.118	4.97E-03	1.152	0.050	1.044	1.271	0.394	Imputed
rs1519778	2	151,678,791	A	G	0.137	0.122	5.52E-03	1.147	0.050	1.041	1.264	0.578	Imputed
rs4664177	2	151,679,055	T	G	0.146	0.130	2.47E-03	1.157	0.048	1.053	1.272	0.496	Imputed
rs4664178	2	151,679,068	T	C	0.135	0.119	4.42E-03	1.153	0.050	1.045	1.271	0.467	Imputed
rs4664179	2	151,679,150	C	T	0.137	0.122	5.05E-03	1.149	0.050	1.043	1.266	0.588	Imputed
rs10803827	2	151,680,209	T	G	0.137	0.122	5.05E-03	1.149	0.050	1.043	1.266	0.588	Imputed
chr2:151680230:I	2	151,680,230	GAT	G	0.022	0.021	0.619	1.059	0.115	0.845	1.328	0.490	Imputed
rs7576195	2	151,680,753	G	A	0.138	0.123	5.24E-03	1.148	0.049	1.042	1.265	0.601	Imputed
rs6432993	2	151,680,884	A	C	0.135	0.119	4.35E-03	1.153	0.050	1.046	1.272	0.430	Imputed
rs7567248	2	151,680,982	T	C	0.139	0.125	9.19E-03	1.137	0.049	1.032	1.252	0.518	Imputed
rs7564415	2	151,681,039	A	G	0.138	0.122	4.23E-03	1.152	0.049	1.046	1.269	0.607	Genotyped
rs7576640	2	151,681,074	G	A	0.138	0.122	4.80E-03	1.150	0.049	1.043	1.267	0.612	Imputed
rs10930290	2	151,681,578	A	G	0.137	0.122	5.30E-03	1.148	0.050	1.042	1.265	0.693	Imputed
rs10930291	2	151,681,786	T	C	0.136	0.120	4.99E-03	1.150	0.050	1.043	1.268	0.773	Imputed
rs10930292	2	151,681,932	A	T	0.135	0.119	4.30E-03	1.153	0.050	1.046	1.271	0.751	Imputed
rs10489979	2	151,682,012	A	G	0.104	0.092	0.014	1.148	0.056	1.029	1.281	0.747	Imputed
rs6730701	2	151,682,212	G	A	0.133	0.118	4.29E-03	1.154	0.050	1.046	1.273	0.594	Imputed
rs1519779	2	151,682,536	C	T	0.131	0.117	9.29E-03	1.140	0.050	1.033	1.259	0.718	Imputed
rs1519780	2	151,682,606	G	A	0.136	0.120	3.60E-03	1.156	0.050	1.048	1.274	0.815	Imputed
rs6706282	2	151,682,924	C	T	0.410	0.370	7.13E-07	1.187	0.035	1.109	1.270	0.953	Imputed
rs6432994	2	151,683,177	G	A	0.136	0.121	4.11E-03	1.153	0.050	1.046	1.271	0.819	Imputed
rs968878	2	151,683,461	T	G	0.136	0.120	4.24E-03	1.153	0.050	1.046	1.271	0.794	Imputed
rs79020158	2	151,683,596	T	C	0.013	0.013	0.750	0.953	0.151	0.709	1.281	0.707	Imputed
rs6432995	2	151,684,419	A	G	0.146	0.130	3.45E-03	1.152	0.048	1.048	1.266	0.432	Imputed
rs74903963	2	151,684,838	C	T	0.104	0.092	0.013	1.149	0.056	1.030	1.282	0.731	Imputed
rs6729783	2	151,685,251	A	G	0.138	0.122	3.59E-03	1.155	0.049	1.048	1.272	0.626	Genotyped
rs6715480	2	151,685,652	C	T	0.138	0.122	4.38E-03	1.151	0.049	1.045	1.269	0.583	Imputed
rs1541436	2	151,685,743	A	C	0.134	0.118	3.85E-03	1.156	0.050	1.048	1.275	0.320	Imputed
rs79768769	2	151,685,896	C	T	0.013	0.013	0.750	0.953	0.151	0.709	1.281	0.825	Imputed
rs78288514	2	151,686,109	A	G	0.011	0.011	0.561	1.098	0.161	0.802	1.504	0.378	Imputed
rs72858553	2	151,686,998	T	C	0.036	0.038	0.305	0.911	0.091	0.763	1.089	0.638	Imputed
rs6738258	2	151,687,598	A	G	0.136	0.121	5.66E-03	1.147	0.050	1.041	1.265	0.291	Imputed
rs2097935	2	151,691,144	A	G	0.134	0.120	6.56E-03	1.146	0.050	1.039	1.264	0.423	Imputed
rs10170910	2	151,691,270	C	T	0.134	0.120	6.22E-03	1.147	0.050	1.040	1.265	0.484	Imputed
rs1829481	2	151,692,837	G	A	0.140	0.124	5.33E-03	1.147	0.049	1.041	1.263	0.621	Imputed
rs1519781	2	151,693,284	A	C	0.136	0.120	2.66E-03	1.161	0.050	1.053	1.280	0.494	Genotyped
rs115336604	2	151,694,518	G	T	0.022	0.020	0.489	1.083	0.116	0.864	1.358	0.562	Imputed

rs1914989	2	151,695,847	C	A	0.140	0.125	6.05E-03	1.144	0.049	1.039	1.260	0.561	Imputed
rs13410272	2	151,696,789	C	T	0.107	0.095	0.019	1.138	0.055	1.021	1.267	0.642	Imputed
rs72858556	2	151,697,042	A	T	0.014	0.011	0.104	1.269	0.147	0.952	1.692	0.685	Imputed
rs1850880	2	151,697,611	T	A	0.135	0.120	6.46E-03	1.146	0.050	1.039	1.264	0.459	Imputed
rs1850879	2	151,698,157	A	G	0.135	0.120	5.05E-03	1.150	0.050	1.043	1.268	0.412	Imputed
rs4368330	2	151,698,431	C	G	0.135	0.120	5.23E-03	1.149	0.050	1.042	1.268	0.409	Imputed
rs1985540	2	151,699,151	A	T	0.135	0.120	5.82E-03	1.148	0.050	1.041	1.266	0.399	Imputed
chr2:151699502:I	2	151,699,502	T	TG	0.134	0.119	5.98E-03	1.148	0.050	1.040	1.266	0.352	Imputed
rs7421622	2	151,699,727	G	A	0.138	0.123	5.65E-03	1.147	0.049	1.041	1.263	0.607	Imputed
rs1813728	2	151,699,892	C	T	0.134	0.120	6.56E-03	1.146	0.050	1.039	1.264	0.424	Imputed
rs76016313	2	151,700,571	G	A	0.065	0.067	0.695	0.974	0.068	0.851	1.113	0.742	Imputed
rs1519755	2	151,701,392	C	T	0.135	0.120	5.71E-03	1.148	0.050	1.041	1.266	0.337	Imputed
chr2:151701581:D	2	151,701,581	C	CA	0.010	0.015	0.028	0.699	0.164	0.507	0.963	0.939	Imputed
rs55841613	2	151,702,435	A	G	0.134	0.119	6.12E-03	1.147	0.050	1.040	1.265	0.395	Imputed
rs62167060	2	151,703,616	C	G	0.116	0.121	0.375	0.954	0.053	0.861	1.058	0.654	Imputed
rs2214896	2	151,703,660	G	A	0.135	0.120	5.62E-03	1.148	0.050	1.041	1.267	0.439	Imputed
rs72858574	2	151,703,828	C	T	0.036	0.038	0.314	0.913	0.091	0.764	1.090	0.631	Imputed
rs917239	2	151,704,211	G	C	0.134	0.120	6.95E-03	1.144	0.050	1.038	1.262	0.451	Imputed
chr2:151704330:D	2	151,704,330	T	TAAG	0.134	0.119	7.92E-03	1.142	0.050	1.035	1.260	0.459	Imputed
rs929388	2	151,704,756	C	T	0.222	0.235	0.088	0.933	0.041	0.862	1.011	0.089	Genotyped
rs72858579	2	151,706,272	G	T	0.228	0.247	7.56E-03	0.898	0.040	0.830	0.972	0.684	Imputed
rs72858582	2	151,706,273	T	G	0.229	0.248	7.29E-03	0.898	0.040	0.830	0.971	0.725	Imputed
rs2190377	2	151,706,704	T	C	0.135	0.120	5.55E-03	1.148	0.050	1.041	1.266	0.492	Imputed
rs2190378	2	151,706,822	A	G	0.172	0.151	4.15E-04	1.173	0.045	1.074	1.282	0.739	Imputed
rs76035740	2	151,706,858	G	C	0.028	0.027	0.676	1.044	0.102	0.854	1.275	0.444	Imputed
rs144033056	2	151,707,709	C	T	0.027	0.023	0.110	1.182	0.105	0.962	1.452	0.908	Imputed
rs74892176	2	151,708,529	C	T	0.009	0.013	0.022	0.671	0.176	0.475	0.947	0.953	Imputed
rs10210373	2	151,708,928	A	G	0.135	0.120	5.62E-03	1.148	0.050	1.041	1.266	0.491	Imputed
rs58900699	2	151,709,837	T	C	0.123	0.117	0.277	1.058	0.052	0.956	1.170	0.931	Imputed
rs72858591	2	151,711,452	C	T	0.082	0.085	0.350	0.944	0.062	0.837	1.065	0.138	Imputed
rs12991056	2	151,711,610	C	T	0.185	0.175	0.121	1.070	0.044	0.982	1.166	0.727	Imputed
rs7579851	2	151,711,700	G	T	0.186	0.175	0.106	1.073	0.044	0.985	1.169	0.702	Imputed
rs7567736	2	151,711,749	A	G	0.166	0.150	0.012	1.122	0.046	1.026	1.227	0.859	Imputed
rs58234421	2	151,711,874	T	C	0.162	0.147	0.014	1.121	0.046	1.024	1.227	0.707	Imputed
rs12472398	2	151,712,240	A	C	0.162	0.147	0.012	1.123	0.046	1.025	1.229	0.722	Imputed
rs12476555	2	151,712,243	T	A	0.162	0.147	0.012	1.123	0.046	1.025	1.229	0.722	Imputed
rs12464150	2	151,712,272	A	G	0.162	0.147	0.012	1.123	0.046	1.025	1.229	0.722	Imputed
rs12472472	2	151,712,393	G	C	0.164	0.147	5.57E-03	1.136	0.046	1.038	1.243	0.681	Imputed
rs12472499	2	151,712,494	A	C	0.162	0.146	0.011	1.124	0.046	1.027	1.231	0.715	Imputed
rs56375806	2	151,713,161	A	G	0.079	0.083	0.236	0.929	0.063	0.822	1.050	0.190	Imputed

rs6433007	2	151,713,358	T	G	0.166	0.150	0.010	1.124	0.046	1.028	1.230	0.877	Imputed
rs59626949	2	151,713,479	C	T	0.079	0.084	0.225	0.927	0.062	0.820	1.048	0.185	Imputed
rs61409252	2	151,713,887	C	T	0.082	0.085	0.346	0.944	0.061	0.837	1.065	0.164	Imputed
rs976722	2	151,714,098	A	T	0.169	0.152	8.24E-03	1.128	0.045	1.031	1.233	0.978	Imputed
rs7578824	2	151,714,350	T	G	0.162	0.147	0.014	1.120	0.046	1.023	1.226	0.737	Imputed
rs7587992	2	151,714,356	G	A	0.162	0.147	0.014	1.120	0.046	1.023	1.226	0.737	Imputed
rs56009465	2	151,714,617	T	C	0.059	0.063	0.350	0.935	0.071	0.813	1.076	0.828	Imputed
rs767118	2	151,714,618	A	G	0.168	0.151	8.54E-03	1.127	0.046	1.031	1.233	0.916	Imputed
rs4442997	2	151,714,861	C	T	0.167	0.151	0.012	1.121	0.046	1.025	1.226	0.911	Imputed
rs1114978	2	151,714,880	T	C	0.246	0.235	0.185	1.054	0.039	0.975	1.138	0.369	Genotyped
rs75219194	2	151,715,065	T	C	0.024	0.018	0.010	1.341	0.114	1.072	1.677	0.603	Imputed
rs2190379	2	151,715,322	C	T	0.163	0.148	0.016	1.117	0.046	1.021	1.223	0.718	Imputed
rs4664779	2	151,715,691	A	G	0.186	0.200	0.045	0.916	0.044	0.841	0.998	0.133	Imputed
rs4664780	2	151,716,876	T	G	0.237	0.228	0.253	1.047	0.040	0.968	1.132	0.797	Imputed
rs6722970	2	151,717,238	C	A	0.329	0.305	2.23E-03	1.117	0.036	1.041	1.200	0.399	Imputed
rs138659599	2	151,717,357	A	G	0.012	0.011	0.510	1.107	0.154	0.818	1.497	0.686	Imputed
rs6433009	2	151,717,998	C	A	0.135	0.134	0.956	0.997	0.050	0.905	1.099	0.963	Imputed
rs6433010	2	151,718,133	C	T	0.135	0.134	0.970	0.998	0.050	0.906	1.100	0.991	Imputed
rs13405938	2	151,718,134	A	G	0.041	0.036	0.113	1.145	0.086	0.968	1.354	0.762	Imputed
rs78964611	2	151,718,714	G	A	0.023	0.025	0.356	0.901	0.112	0.723	1.123	0.030	Imputed
rs981893	2	151,719,004	T	A	0.135	0.134	0.957	0.997	0.050	0.905	1.099	0.996	Imputed
rs2079456	2	151,719,268	A	G	0.044	0.038	0.061	1.169	0.083	0.993	1.376	0.503	Imputed
rs78991766	2	151,719,593	G	A	0.021	0.023	0.328	0.891	0.117	0.708	1.121	0.006	Imputed
rs11896420	2	151,719,671	A	G	0.281	0.263	0.017	1.094	0.038	1.016	1.178	0.715	Imputed
rs35278637	2	151,720,041	G	C	0.023	0.025	0.356	0.901	0.112	0.723	1.123	0.030	Imputed
rs34122850	2	151,720,083	C	A	0.023	0.025	0.356	0.901	0.112	0.723	1.123	0.030	Imputed
rs62167085	2	151,720,206	G	C	0.134	0.134	0.842	0.990	0.050	0.898	1.091	0.920	Imputed
rs61613689	2	151,720,469	G	A	0.043	0.037	0.081	1.158	0.084	0.982	1.366	0.573	Imputed
rs12621936	2	151,721,040	T	C	0.024	0.026	0.349	0.900	0.112	0.723	1.120	0.020	Genotyped
rs12469219	2	151,721,642	G	A	0.045	0.038	0.036	1.188	0.083	1.011	1.397	0.475	Imputed
rs13392322	2	151,722,060	C	T	0.024	0.026	0.345	0.901	0.111	0.725	1.118	0.052	Imputed
rs139419045	2	151,722,182	T	A	0.010	0.011	0.906	0.981	0.166	0.708	1.358	0.549	Imputed
rs6433014	2	151,722,220	A	T	0.043	0.038	0.124	1.138	0.084	0.965	1.341	0.489	Imputed
rs58350940	2	151,722,436	T	C	0.183	0.193	0.159	0.940	0.044	0.863	1.024	0.016	Imputed
rs7586245	2	151,722,437	A	G	0.021	0.024	0.222	0.866	0.118	0.688	1.092	0.340	Imputed
rs6743453	2	151,723,171	T	G	0.024	0.026	0.436	0.917	0.111	0.738	1.139	0.058	Imputed
rs10803831	2	151,723,184	A	G	0.024	0.026	0.423	0.915	0.111	0.737	1.136	0.056	Imputed
rs55657971	2	151,724,494	A	G	0.037	0.039	0.384	0.925	0.090	0.776	1.103	0.908	Imputed
rs6433015	2	151,725,236	T	A	0.024	0.026	0.394	0.909	0.111	0.731	1.130	0.051	Imputed
chr2:151725249:I	2	151,725,249	C	CA	0.137	0.135	0.982	1.001	0.049	0.909	1.103	0.926	Imputed

rs6761863	2	151,725,382	G	A	0.137	0.135	0.982	1.001	0.049	0.909	1.103	0.926	Imputed
rs6748877	2	151,725,498	A	G	0.026	0.027	0.512	0.932	0.107	0.756	1.149	0.042	Imputed
rs62167088	2	151,725,684	A	G	0.036	0.037	0.711	0.967	0.091	0.809	1.155	0.286	Imputed
rs142679602	2	151,725,724	T	G	0.031	0.029	0.581	1.056	0.098	0.871	1.280	0.524	Imputed
rs6737290	2	151,725,741	C	T	0.026	0.027	0.580	0.942	0.108	0.763	1.163	0.037	Imputed
rs10195263	2	151,725,792	G	A	0.024	0.026	0.347	0.900	0.111	0.724	1.119	0.045	Genotyped
rs13004017	2	151,726,326	T	C	0.282	0.266	0.033	1.084	0.038	1.007	1.167	0.760	Genotyped
rs17177861	2	151,726,388	A	G	0.068	0.057	1.84E-03	1.235	0.068	1.081	1.411	0.742	Imputed
rs12619871	2	151,726,402	A	G	0.023	0.025	0.279	0.884	0.113	0.708	1.104	0.022	Imputed
rs4664193	2	151,726,615	G	A	0.025	0.026	0.513	0.931	0.109	0.753	1.152	0.023	Imputed
rs12614419	2	151,726,724	T	C	0.023	0.025	0.279	0.884	0.113	0.708	1.104	0.022	Imputed
rs115657032	2	151,726,957	T	G	0.018	0.019	0.625	0.940	0.126	0.734	1.204	0.341	Imputed
rs12467192	2	151,726,959	C	T	0.136	0.136	0.872	0.992	0.049	0.901	1.093	0.894	Genotyped
rs62167089	2	151,727,589	G	A	0.116	0.124	0.148	0.927	0.053	0.836	1.028	0.986	Imputed
rs4423587	2	151,727,616	G	C	0.025	0.026	0.528	0.933	0.109	0.754	1.155	0.024	Imputed
rs59741702	2	151,727,971	C	T	0.045	0.038	0.062	1.167	0.083	0.992	1.372	0.697	Imputed
rs12621706	2	151,728,115	A	G	0.023	0.025	0.279	0.884	0.113	0.708	1.104	0.022	Imputed
rs12621710	2	151,728,153	A	G	0.023	0.025	0.279	0.884	0.113	0.708	1.104	0.022	Imputed
rs2016391	2	151,728,209	A	G	0.104	0.108	0.382	0.953	0.055	0.855	1.062	0.943	Imputed
rs2016389	2	151,728,241	T	C	0.091	0.096	0.235	0.933	0.059	0.832	1.046	0.847	Imputed
rs1005274	2	151,728,377	G	A	0.093	0.097	0.283	0.940	0.058	0.838	1.053	0.779	Genotyped
rs34650710	2	151,728,543	A	C	0.038	0.039	0.644	0.960	0.088	0.808	1.141	0.213	Imputed
rs2158820	2	151,728,549	G	C	0.134	0.134	0.888	0.993	0.050	0.901	1.095	0.953	Imputed
rs6433017	2	151,728,587	T	C	0.117	0.127	0.076	0.911	0.052	0.822	1.010	0.860	Genotyped
rs60338791	2	151,728,830	C	G	0.067	0.070	0.398	0.945	0.067	0.828	1.078	0.286	Imputed
rs2190380	2	151,728,859	G	A	0.093	0.097	0.308	0.942	0.058	0.841	1.056	0.803	Imputed
rs2024036	2	151,729,225	C	G	0.093	0.098	0.264	0.937	0.058	0.836	1.050	0.744	Imputed
rs6433018	2	151,729,571	A	G	0.068	0.070	0.468	0.953	0.067	0.835	1.087	0.549	Genotyped
rs6753573	2	151,729,818	C	G	0.095	0.097	0.472	0.959	0.058	0.857	1.074	0.765	Imputed
rs60703324	2	151,729,827	G	C	0.042	0.038	0.135	1.135	0.085	0.961	1.339	0.593	Imputed
rs13421269	2	151,730,175	C	T	0.043	0.039	0.142	1.130	0.084	0.959	1.332	0.556	Imputed
rs10189619	2	151,731,930	C	T	0.507	0.491	0.076	1.062	0.034	0.994	1.135	0.926	Genotyped
rs1990149	2	151,731,950	C	T	0.115	0.114	0.807	1.013	0.053	0.913	1.124	0.963	Genotyped
rs78853258	2	151,732,265	C	T	0.041	0.039	0.603	1.045	0.085	0.884	1.236	0.951	Imputed
rs62167090	2	151,732,718	T	C	0.018	0.020	0.545	0.926	0.127	0.722	1.188	0.532	Imputed
rs12618428	2	151,732,979	G	A	0.129	0.125	0.407	1.043	0.051	0.945	1.152	0.263	Imputed
rs116321269	2	151,732,998	C	G	0.016	0.014	0.233	1.176	0.135	0.902	1.533	0.377	Imputed
rs960615	2	151,733,201	T	C	0.197	0.206	0.130	0.938	0.042	0.863	1.019	0.998	Genotyped
rs77060483	2	151,733,381	T	C	0.018	0.017	0.766	1.039	0.129	0.807	1.338	0.342	Imputed
rs2341594	2	151,733,441	A	G	0.124	0.127	0.584	0.972	0.051	0.879	1.075	0.505	Imputed

rs12469061	2	151,733,659	T	C	0.197	0.180	6.00E-03	1.125	0.043	1.034	1.223	0.880	Imputed
rs77388016	2	151,733,840	T	C	0.081	0.079	0.700	1.024	0.062	0.907	1.157	0.915	Imputed
chr2:151734104:D	2	151,734,104	A	AT	0.176	0.186	0.095	0.929	0.044	0.851	1.013	0.789	Imputed
rs10469635	2	151,734,630	T	C	0.435	0.422	0.081	1.061	0.034	0.993	1.135	0.457	Imputed
rs6706651	2	151,734,631	A	G	0.125	0.127	0.728	0.982	0.051	0.889	1.086	0.486	Imputed
rs6709687	2	151,734,679	A	G	0.159	0.160	0.904	0.994	0.046	0.908	1.089	0.876	Imputed
rs12151724	2	151,734,715	A	T	0.081	0.079	0.684	1.026	0.062	0.908	1.158	0.996	Imputed
rs2880095	2	151,734,818	T	C	0.124	0.127	0.689	0.980	0.051	0.886	1.083	0.494	Imputed
rs6723323	2	151,735,155	G	A	0.254	0.252	0.756	1.012	0.039	0.938	1.092	0.158	Genotyped
rs10199904	2	151,735,348	T	C	0.125	0.128	0.658	0.978	0.051	0.884	1.081	0.597	Imputed
chr2:151735362:D	2	151,735,362	GA	G	0.253	0.253	0.796	1.010	0.039	0.936	1.090	0.153	Imputed
rs726503	2	151,735,805	G	T	0.253	0.252	0.740	1.013	0.039	0.939	1.093	0.152	Imputed
rs75667030	2	151,735,866	C	T	0.040	0.038	0.752	1.028	0.087	0.867	1.220	0.299	Imputed
rs10203161	2	151,736,256	C	T	0.128	0.124	0.489	1.036	0.051	0.938	1.144	0.243	Imputed
rs6760354	2	151,736,705	C	T	0.428	0.437	0.276	0.963	0.034	0.901	1.030	0.306	Imputed
rs10206083	2	151,736,804	T	C	0.299	0.312	0.081	0.938	0.037	0.872	1.008	0.914	Imputed
rs10206093	2	151,736,844	T	C	0.299	0.312	0.081	0.938	0.037	0.872	1.008	0.927	Imputed
rs16828815	2	151,736,937	G	T	0.172	0.183	0.076	0.924	0.045	0.847	1.008	0.733	Imputed
rs79308412	2	151,737,087	A	G	0.082	0.080	0.728	1.022	0.062	0.905	1.153	0.919	Imputed
rs10191781	2	151,737,127	C	T	0.299	0.312	0.099	0.941	0.037	0.875	1.012	0.925	Imputed
rs62167091	2	151,737,238	A	G	0.039	0.039	0.762	1.027	0.087	0.865	1.218	0.738	Imputed
chr2:151737434:D	2	151,737,434	CAGT	C	0.433	0.444	0.181	0.955	0.034	0.893	1.021	0.333	Imputed
rs4664199	2	151,738,287	C	A	0.174	0.185	0.078	0.925	0.044	0.847	1.009	0.835	Imputed
rs6707835	2	151,738,382	C	T	0.425	0.436	0.225	0.959	0.034	0.897	1.026	0.369	Imputed
rs77861697	2	151,738,487	G	T	0.017	0.018	0.855	0.977	0.130	0.758	1.259	0.403	Imputed
rs757921	2	151,738,700	A	G	0.124	0.127	0.719	0.982	0.051	0.888	1.085	0.620	Imputed
rs75482237	2	151,738,987	T	C	0.123	0.112	0.041	1.112	0.052	1.005	1.231	0.699	Imputed
rs72860188	2	151,739,275	A	C	0.175	0.185	0.100	0.930	0.044	0.852	1.014	0.889	Imputed
rs17178183	2	151,739,348	T	C	0.175	0.185	0.100	0.930	0.044	0.852	1.014	0.889	Imputed
rs10166214	2	151,739,560	T	C	0.302	0.313	0.135	0.947	0.037	0.881	1.017	0.718	Imputed
rs10210872	2	151,739,579	C	A	0.214	0.207	0.323	1.042	0.041	0.961	1.130	0.468	Genotyped
rs2214897	2	151,739,748	G	A	0.302	0.313	0.135	0.947	0.037	0.881	1.017	0.718	Imputed
rs77834577	2	151,740,507	G	A	0.123	0.113	0.049	1.107	0.052	1.000	1.225	0.659	Imputed
rs6433020	2	151,740,770	C	T	0.125	0.127	0.792	0.987	0.051	0.892	1.091	0.489	Imputed
rs16828818	2	151,741,204	C	T	0.090	0.095	0.389	0.951	0.059	0.847	1.067	0.573	Imputed
rs7585613	2	151,741,365	T	C	0.074	0.080	0.184	0.918	0.064	0.810	1.041	0.715	Genotyped
rs7558214	2	151,741,406	A	G	0.075	0.081	0.199	0.921	0.064	0.813	1.044	0.628	Genotyped
rs17178319	2	151,741,581	C	G	0.174	0.184	0.087	0.927	0.044	0.849	1.011	0.836	Imputed
rs12472402	2	151,742,339	G	C	0.410	0.399	0.169	1.048	0.034	0.980	1.122	0.451	Imputed
rs1859723	2	151,742,387	A	G	0.125	0.127	0.809	0.988	0.051	0.893	1.092	0.480	Imputed

rs1859724	2	151,742,522	C	T	0.301	0.313	0.112	0.943	0.037	0.877	1.014	0.797	Genotyped
rs2190381	2	151,743,024	G	T	0.124	0.112	0.043	1.111	0.052	1.004	1.229	0.576	Imputed
rs4664201	2	151,743,342	C	A	0.043	0.051	0.018	0.823	0.083	0.700	0.967	0.957	Imputed
rs2214898	2	151,743,382	A	T	0.124	0.112	0.043	1.111	0.052	1.003	1.229	0.575	Imputed
rs978741	2	151,743,392	T	C	0.090	0.094	0.395	0.951	0.059	0.847	1.068	0.535	Imputed
rs16828833	2	151,743,498	T	G	0.074	0.080	0.166	0.915	0.064	0.806	1.038	0.715	Imputed
rs12623693	2	151,743,900	G	A	0.090	0.094	0.395	0.951	0.059	0.847	1.068	0.516	Imputed
rs17178545	2	151,744,095	T	C	0.175	0.185	0.091	0.928	0.044	0.850	1.012	0.697	Imputed
rs10209774	2	151,744,426	G	A	0.090	0.094	0.381	0.950	0.059	0.846	1.066	0.507	Genotyped
rs16828835	2	151,744,452	A	T	0.074	0.080	0.162	0.914	0.064	0.806	1.037	0.720	Imputed
rs112218868	2	151,744,698	T	C	0.011	0.011	0.585	1.090	0.159	0.799	1.488	0.086	Imputed
rs62167093	2	151,744,699	A	G	0.175	0.185	0.084	0.926	0.044	0.849	1.010	0.712	Imputed
rs72862916	2	151,744,761	G	A	0.074	0.080	0.175	0.917	0.064	0.808	1.040	0.702	Imputed
rs78904109	2	151,744,806	T	C	0.124	0.113	0.037	1.114	0.052	1.006	1.232	0.616	Imputed
chr2:151744892:D	2	151,744,892	G	GAGA	0.174	0.185	0.073	0.924	0.044	0.846	1.008	0.755	Imputed
rs10168022	2	151,744,908	C	T	0.090	0.095	0.374	0.949	0.059	0.845	1.065	0.522	Imputed
rs4664202	2	151,745,211	C	T	0.409	0.399	0.194	1.046	0.034	0.978	1.119	0.596	Imputed
rs62167109	2	151,745,882	C	T	0.175	0.185	0.094	0.928	0.044	0.851	1.013	0.690	Imputed
rs72862921	2	151,746,048	T	C	0.014	0.013	0.353	1.144	0.145	0.862	1.519	0.731	Imputed
rs17178664	2	151,746,348	T	C	0.409	0.399	0.192	1.046	0.034	0.978	1.119	0.593	Imputed
rs10489980	2	151,746,396	C	G	0.409	0.399	0.192	1.046	0.034	0.978	1.119	0.593	Imputed
rs2214891	2	151,746,846	A	G	0.125	0.127	0.821	0.989	0.051	0.894	1.093	0.566	Imputed
rs2214892	2	151,746,876	A	G	0.258	0.245	0.062	1.075	0.039	0.996	1.160	0.925	Genotyped
rs62167110	2	151,747,296	T	C	0.175	0.185	0.090	0.927	0.044	0.850	1.012	0.699	Imputed
rs114781367	2	151,747,340	A	C	0.019	0.015	0.048	1.284	0.127	1.001	1.646	0.223	Imputed
rs1990147	2	151,747,915	C	T	0.125	0.127	0.829	0.989	0.051	0.895	1.093	0.570	Imputed
chr2:151748101:D	2	151,748,101	G	GAAGA	0.475	0.471	0.660	1.015	0.034	0.950	1.085	0.457	Imputed
rs10181266	2	151,748,836	G	T	0.090	0.095	0.368	0.948	0.059	0.845	1.065	0.518	Imputed
rs77142918	2	151,749,662	T	C	0.124	0.113	0.036	1.115	0.052	1.007	1.233	0.602	Imputed
rs2190367	2	151,749,834	T	C	0.074	0.081	0.124	0.906	0.064	0.799	1.027	0.637	Imputed
rs2214893	2	151,749,973	G	A	0.125	0.127	0.790	0.987	0.051	0.892	1.091	0.430	Imputed
rs6730185	2	151,750,506	G	A	0.092	0.096	0.437	0.956	0.058	0.852	1.071	0.532	Genotyped
rs10930331	2	151,750,639	A	G	0.407	0.396	0.195	1.046	0.034	0.977	1.119	0.508	Genotyped
chr2:151750883:D	2	151,750,883	A	AC	0.011	0.011	0.585	1.090	0.159	0.799	1.488	0.086	Imputed
rs10167831	2	151,750,892	A	C	0.125	0.127	0.849	0.990	0.051	0.896	1.095	0.438	Imputed
rs62167111	2	151,750,934	G	C	0.174	0.185	0.076	0.924	0.044	0.847	1.008	0.732	Imputed
rs10167843	2	151,750,936	A	T	0.125	0.127	0.812	0.988	0.051	0.894	1.092	0.439	Imputed
rs77950715	2	151,751,328	G	A	0.014	0.011	0.101	1.273	0.147	0.954	1.699	0.815	Imputed
rs1859718	2	151,751,396	G	A	0.499	0.492	0.370	1.031	0.034	0.965	1.101	0.789	Imputed
rs1990148	2	151,751,702	G	A	0.090	0.095	0.366	0.948	0.059	0.845	1.064	0.537	Imputed

rs111303643	2	151,752,296	C	G	0.038	0.044	0.099	0.866	0.087	0.730	1.028	0.815	Imputed
rs7570251	2	151,752,329	C	T	0.075	0.081	0.152	0.912	0.064	0.805	1.034	0.596	Imputed
rs6716692	2	151,752,439	T	A	0.174	0.184	0.076	0.924	0.045	0.847	1.008	0.748	Imputed
rs2190368	2	151,752,775	G	A	0.466	0.490	5.59E-03	0.910	0.034	0.852	0.973	0.752	Imputed
rs16828857	2	151,753,548	A	C	0.075	0.081	0.152	0.912	0.064	0.805	1.034	0.596	Imputed
rs6727152	2	151,753,832	C	T	0.090	0.095	0.400	0.952	0.059	0.848	1.068	0.539	Imputed
rs78009712	2	151,753,877	G	A	0.124	0.113	0.042	1.111	0.052	1.004	1.229	0.598	Imputed
rs10188669	2	151,753,903	G	A	0.466	0.489	5.57E-03	0.910	0.034	0.852	0.973	0.738	Genotyped
chr2:151754338:I	2	151,754,338	A	AG	0.127	0.129	0.882	0.993	0.051	0.898	1.096	0.389	Imputed
rs4664809	2	151,754,368	C	A	0.409	0.398	0.154	1.050	0.034	0.982	1.124	0.524	Imputed
rs72862941	2	151,754,400	T	C	0.090	0.094	0.381	0.950	0.059	0.846	1.066	0.527	Imputed
rs6724697	2	151,754,448	A	G	0.125	0.127	0.828	0.989	0.051	0.895	1.093	0.414	Imputed
rs16828865	2	151,754,602	C	T	0.075	0.081	0.152	0.912	0.064	0.805	1.034	0.596	Imputed
rs12994538	2	151,754,956	A	G	0.038	0.038	0.951	0.995	0.089	0.836	1.183	0.386	Imputed
rs12996110	2	151,754,978	T	C	0.472	0.469	0.724	1.012	0.034	0.947	1.082	0.189	Genotyped
rs75229806	2	151,755,090	A	C	0.124	0.113	0.042	1.111	0.052	1.004	1.229	0.598	Imputed
rs10489981	2	151,755,117	T	C	0.090	0.094	0.388	0.950	0.059	0.847	1.067	0.531	Imputed
rs79953851	2	151,755,522	C	A	0.124	0.113	0.042	1.111	0.052	1.004	1.229	0.598	Imputed
chr2:151755727:D	2	151,755,727	C	CACACACACAT	0.408	0.398	0.232	1.042	0.034	0.974	1.115	0.967	Imputed
rs72862945	2	151,755,779	T	C	0.170	0.181	0.064	0.920	0.045	0.843	1.005	0.746	Imputed
rs2190369	2	151,756,019	G	A	0.090	0.094	0.381	0.950	0.059	0.846	1.066	0.527	Imputed
rs2190370	2	151,756,087	G	A	0.090	0.094	0.381	0.950	0.059	0.846	1.066	0.527	Imputed
rs74568159	2	151,756,126	C	T	0.124	0.113	0.042	1.111	0.052	1.004	1.229	0.598	Imputed
rs12470304	2	151,756,279	G	A	0.410	0.398	0.148	1.051	0.034	0.983	1.124	0.516	Imputed
rs12471226	2	151,756,314	A	T	0.410	0.398	0.153	1.050	0.034	0.982	1.124	0.523	Imputed
rs72862947	2	151,756,346	G	T	0.014	0.013	0.353	1.144	0.145	0.862	1.519	0.731	Imputed
rs12474028	2	151,756,445	A	G	0.410	0.398	0.148	1.051	0.034	0.983	1.124	0.516	Imputed
rs12471262	2	151,756,466	A	T	0.410	0.398	0.148	1.051	0.034	0.983	1.124	0.516	Imputed
rs72862948	2	151,756,705	C	T	0.021	0.020	0.604	1.064	0.119	0.842	1.344	0.494	Imputed
rs16828883	2	151,756,711	T	C	0.075	0.081	0.150	0.912	0.064	0.805	1.034	0.599	Imputed
rs10198677	2	151,756,935	A	G	0.485	0.480	0.502	1.023	0.034	0.957	1.093	0.339	Imputed
rs57691554	2	151,757,107	A	G	0.075	0.081	0.150	0.912	0.064	0.805	1.034	0.599	Imputed
rs12692884	2	151,757,190	C	A	0.090	0.095	0.375	0.949	0.059	0.845	1.065	0.522	Imputed
rs10198969	2	151,757,260	A	G	0.404	0.394	0.204	1.045	0.035	0.976	1.118	0.370	Imputed
chr2:151757465:I	2	151,757,465	AG	A	0.410	0.398	0.135	1.053	0.034	0.984	1.126	0.523	Imputed
rs72862957	2	151,757,520	T	C	0.075	0.081	0.150	0.912	0.064	0.805	1.034	0.599	Imputed
rs7601783	2	151,758,242	A	G	0.076	0.082	0.167	0.916	0.064	0.808	1.037	0.555	Imputed
rs60216830	2	151,758,374	A	G	0.090	0.094	0.376	0.949	0.059	0.845	1.066	0.523	Imputed
rs7602001	2	151,758,422	A	G	0.076	0.082	0.180	0.918	0.064	0.811	1.040	0.539	Imputed
rs11687663	2	151,758,445	C	T	0.410	0.398	0.133	1.053	0.034	0.984	1.127	0.486	Imputed

rs1859719	2	151,759,180	A	T	0.410	0.398	0.149	1.051	0.034	0.982	1.124	0.517	Imputed
rs17794030	2	151,759,490	T	C	0.174	0.185	0.064	0.921	0.045	0.844	1.005	0.781	Imputed
rs13418188	2	151,759,975	G	A	0.090	0.094	0.381	0.950	0.059	0.846	1.066	0.527	Imputed
rs1076309	2	151,760,053	T	C	0.331	0.326	0.506	1.024	0.036	0.954	1.099	0.736	Imputed
rs1079089	2	151,760,165	A	G	0.197	0.192	0.529	1.027	0.043	0.945	1.117	0.939	Imputed
rs13032164	2	151,760,452	G	C	0.137	0.149	0.040	0.904	0.049	0.822	0.996	0.921	Imputed
rs76518694	2	151,760,617	T	C	0.074	0.072	0.828	1.014	0.065	0.893	1.151	0.698	Imputed
rs35302780	2	151,760,756	T	C	0.202	0.187	0.030	1.097	0.042	1.009	1.192	0.873	Imputed
rs740274	2	151,760,808	T	G	0.443	0.420	6.15E-03	1.098	0.034	1.027	1.174	0.345	Genotyped
rs80081269	2	151,760,925	A	C	0.125	0.116	0.116	1.084	0.051	0.980	1.199	0.761	Imputed
rs78507847	2	151,761,128	A	G	0.103	0.097	0.292	1.061	0.056	0.951	1.184	0.611	Imputed
rs72862962	2	151,761,254	G	A	0.144	0.144	0.855	1.009	0.048	0.918	1.109	0.715	Imputed
rs62167112	2	151,761,448	T	A	0.334	0.331	0.714	1.013	0.036	0.944	1.087	0.914	Imputed
rs78591232	2	151,761,540	T	C	0.127	0.120	0.185	1.070	0.051	0.968	1.182	0.441	Imputed
rs115426504	2	151,761,579	G	A	0.019	0.017	0.449	1.100	0.126	0.860	1.406	0.969	Imputed
rs75981941	2	151,761,609	C	T	0.145	0.136	0.180	1.067	0.048	0.971	1.173	0.550	Imputed
rs62167113	2	151,761,668	A	G	0.252	0.243	0.252	1.046	0.039	0.969	1.129	0.732	Imputed
rs142219736	2	151,761,905	C	T	0.023	0.020	0.312	1.123	0.115	0.897	1.407	0.461	Imputed
chr2:151761953:D	2	151,761,953	G	GC	0.022	0.024	0.749	0.964	0.114	0.771	1.206	0.973	Imputed
chr2:151761955:D	2	151,761,955	C	CT	0.186	0.180	0.244	1.052	0.044	0.966	1.146	0.626	Imputed
rs79383822	2	151,762,194	A	G	0.016	0.013	0.147	1.221	0.138	0.931	1.601	0.688	Imputed
rs6735645	2	151,762,436	T	A	0.109	0.113	0.466	0.961	0.054	0.865	1.069	0.640	Imputed
rs75158222	2	151,762,639	G	A	0.102	0.095	0.228	1.070	0.056	0.959	1.194	0.770	Imputed
rs10200704	2	151,762,741	A	G	0.342	0.340	0.820	1.008	0.036	0.940	1.081	0.937	Genotyped
rs4664204	2	151,762,917	A	G	0.194	0.187	0.228	1.053	0.043	0.968	1.145	0.543	Imputed
rs116667811	2	151,763,044	T	C	0.030	0.028	0.300	1.109	0.099	0.913	1.347	0.245	Imputed
rs4664205	2	151,763,050	A	C	0.326	0.342	0.046	0.931	0.036	0.867	0.999	0.787	Imputed
rs13020081	2	151,763,686	A	G	0.194	0.187	0.186	1.058	0.043	0.973	1.151	0.609	Imputed
rs72862974	2	151,763,775	A	G	0.016	0.013	0.138	1.227	0.138	0.936	1.609	0.802	Imputed
rs917237	2	151,764,512	G	A	0.209	0.208	0.953	0.998	0.042	0.919	1.082	0.626	Genotyped
rs28399772	2	151,764,912	C	T	0.208	0.207	0.942	0.997	0.042	0.919	1.082	0.592	Imputed
rs13010870	2	151,765,163	C	T	0.216	0.205	0.088	1.073	0.041	0.990	1.163	0.642	Genotyped
rs12470461	2	151,765,447	T	C	0.207	0.206	0.965	0.998	0.042	0.920	1.083	0.688	Imputed
rs74369587	2	151,766,978	G	A	0.094	0.092	0.757	1.018	0.058	0.909	1.141	0.723	Imputed
rs60076148	2	151,767,049	T	G	0.485	0.463	9.69E-03	1.092	0.034	1.021	1.167	0.618	Imputed
rs58545632	2	151,767,050	C	T	0.393	0.418	2.66E-03	0.901	0.035	0.842	0.965	0.398	Imputed
rs16828944	2	151,767,424	C	T	0.419	0.439	0.017	0.922	0.034	0.862	0.986	0.425	Imputed
rs35819210	2	151,767,443	C	T	0.393	0.416	5.36E-03	0.908	0.035	0.848	0.972	0.396	Imputed
rs16828946	2	151,767,786	A	G	0.105	0.107	0.676	0.977	0.055	0.877	1.089	0.557	Imputed
rs2214894	2	151,767,919	A	T	0.020	0.025	0.083	0.813	0.119	0.644	1.028	0.557	Imputed

rs2190371	2	151,767,977	T	A	0.144	0.148	0.573	0.973	0.048	0.886	1.070	0.748	Imputed
chr2:151768433:I	2	151,768,433	GAA	G	0.249	0.230	8.64E-03	1.109	0.039	1.027	1.198	0.199	Imputed
rs62167115	2	151,769,380	G	T	0.088	0.081	0.174	1.085	0.060	0.965	1.220	0.521	Imputed
rs7421119	2	151,769,518	A	G	0.132	0.131	0.832	1.011	0.050	0.916	1.115	0.779	Genotyped
rs77556452	2	151,770,404	T	C	0.014	0.013	0.374	1.139	0.147	0.855	1.519	0.652	Imputed
rs7601653	2	151,770,630	T	C	0.121	0.118	0.530	1.033	0.052	0.933	1.144	0.498	Imputed
rs7601656	2	151,770,636	A	C	0.119	0.117	0.600	1.028	0.052	0.928	1.139	0.478	Imputed
rs57632653	2	151,771,402	G	A	0.290	0.285	0.632	1.018	0.037	0.946	1.095	0.603	Imputed
rs62167118	2	151,772,157	A	T	0.074	0.070	0.514	1.043	0.065	0.919	1.185	0.594	Imputed
rs62167119	2	151,772,158	A	C	0.074	0.070	0.514	1.043	0.065	0.919	1.185	0.594	Imputed
rs80322972	2	151,772,223	G	T	0.094	0.094	0.972	0.998	0.058	0.891	1.118	0.785	Imputed
rs76432123	2	151,772,384	G	A	0.095	0.095	0.920	1.006	0.058	0.898	1.126	0.635	Imputed
rs34030124	2	151,772,494	T	C	0.146	0.151	0.471	0.966	0.048	0.880	1.061	0.618	Imputed
rs2214895	2	151,772,816	C	T	0.025	0.022	0.199	1.151	0.110	0.929	1.428	0.693	Imputed
rs78606425	2	151,773,226	T	C	0.202	0.203	0.826	0.991	0.042	0.912	1.076	0.787	Imputed
rs16828958	2	151,773,304	C	G	0.352	0.359	0.458	0.974	0.035	0.909	1.044	0.364	Imputed
rs917238	2	151,773,679	G	C	0.147	0.152	0.476	0.967	0.048	0.880	1.061	0.564	Imputed
rs1859720	2	151,773,732	C	T	0.333	0.350	0.034	0.927	0.036	0.864	0.994	0.785	Genotyped
rs13003656	2	151,773,871	A	G	0.146	0.152	0.457	0.965	0.048	0.879	1.060	0.639	Imputed
rs2190373	2	151,774,093	G	A	0.420	0.424	0.641	0.984	0.034	0.920	1.052	0.328	Genotyped
rs11673872	2	151,774,124	C	T	0.255	0.264	0.288	0.960	0.039	0.890	1.035	0.351	Genotyped
rs80216420	2	151,774,233	A	G	0.048	0.055	0.044	0.853	0.079	0.731	0.996	0.725	Imputed
rs79977377	2	151,774,234	A	C	0.049	0.056	0.050	0.858	0.078	0.737	1.000	0.677	Imputed
chr2:151774510:D	2	151,774,510	T	TA	0.276	0.293	0.019	0.915	0.038	0.850	0.986	0.177	Imputed
chr2:151774520:I	2	151,774,520	AG	A	0.055	0.048	0.040	1.166	0.075	1.007	1.351	0.751	Imputed
chr2:151774521:D	2	151,774,521	G	GT	0.273	0.293	5.83E-03	0.901	0.038	0.836	0.970	0.108	Imputed
rs34788394	2	151,774,522	G	T	0.289	0.311	1.98E-03	0.891	0.037	0.829	0.959	0.093	Imputed
rs12328269	2	151,774,643	A	G	0.443	0.418	1.98E-03	1.111	0.034	1.039	1.188	0.092	Genotyped
rs13383157	2	151,774,981	G	A	0.292	0.270	2.06E-03	1.122	0.037	1.043	1.207	0.062	Imputed
rs66739021	2	151,775,409	A	G	0.284	0.309	4.98E-04	0.878	0.037	0.816	0.945	0.192	Imputed
rs11690942	2	151,775,556	C	T	0.433	0.409	2.77E-03	1.108	0.034	1.036	1.185	0.328	Imputed
chr2:151775761:D	2	151,775,761	GA	G	0.406	0.383	3.99E-03	1.104	0.035	1.032	1.182	0.078	Imputed
rs114937118	2	151,775,790	A	G	0.094	0.093	0.835	1.012	0.058	0.904	1.134	0.644	Imputed
rs72862991	2	151,776,114	T	C	0.021	0.019	0.433	1.097	0.119	0.870	1.385	0.946	Imputed
rs1548632	2	151,776,216	C	T	0.414	0.390	2.26E-03	1.111	0.034	1.038	1.188	0.147	Genotyped
chr2:151776748:I	2	151,776,748	TG	T	0.299	0.277	2.32E-03	1.120	0.037	1.041	1.204	0.076	Imputed
rs79381202	2	151,776,988	A	G	0.018	0.019	0.862	0.978	0.127	0.763	1.255	0.378	Imputed
rs13031323	2	151,777,621	A	G	0.288	0.314	4.06E-04	0.877	0.037	0.815	0.943	0.101	Imputed
rs10177702	2	151,777,665	T	C	0.074	0.075	0.877	0.990	0.064	0.873	1.123	0.517	Imputed
rs16828979	2	151,777,756	T	C	0.046	0.042	0.277	1.093	0.082	0.931	1.283	0.661	Imputed

rs10210491	2	151,778,014	A	C	0.298	0.277	2.48E-03	1.119	0.037	1.040	1.203	0.083	Imputed
rs7571912	2	151,778,154	A	G	0.419	0.394	1.72E-03	1.114	0.034	1.041	1.191	0.132	Imputed
rs75399158	2	151,778,466	C	A	0.120	0.117	0.529	1.033	0.052	0.933	1.144	0.869	Imputed
rs13016055	2	151,778,653	T	A	0.289	0.315	5.17E-04	0.879	0.037	0.817	0.946	0.090	Imputed
rs114974957	2	151,778,947	T	G	0.038	0.039	0.793	0.977	0.088	0.823	1.161	0.652	Imputed
chr2:151779368:D	2	151,779,368	T	TTA	0.198	0.198	0.975	1.001	0.042	0.921	1.088	0.830	Imputed
rs12465152	2	151,779,777	G	C	0.094	0.093	0.904	1.007	0.058	0.899	1.129	0.621	Imputed
rs10184706	2	151,780,028	C	T	0.299	0.277	2.07E-03	1.121	0.037	1.042	1.206	0.088	Imputed
rs62167120	2	151,780,795	T	C	0.046	0.042	0.298	1.089	0.082	0.928	1.278	0.643	Imputed
rs12692888	2	151,781,053	T	C	0.120	0.117	0.636	1.025	0.052	0.925	1.135	0.880	Genotyped
rs17179752	2	151,781,156	A	T	0.299	0.277	2.30E-03	1.120	0.037	1.041	1.204	0.085	Imputed
rs964645	2	151,781,642	A	G	0.101	0.101	0.948	0.996	0.056	0.893	1.112	0.731	Imputed
rs6433059	2	151,781,982	T	A	0.197	0.198	0.918	1.004	0.042	0.924	1.092	0.790	Imputed
rs6433060	2	151,782,003	A	G	0.126	0.125	0.860	1.009	0.051	0.913	1.115	0.798	Imputed
rs116618154	2	151,782,052	T	C	0.011	0.013	0.232	0.823	0.163	0.598	1.133	0.953	Imputed
rs7567635	2	151,782,570	G	A	0.052	0.047	0.240	1.095	0.077	0.941	1.274	0.397	Imputed
rs190754833	2	151,782,787	A	T	0.017	0.017	0.846	1.026	0.131	0.794	1.326	0.462	Imputed
rs13404308	2	151,782,825	T	G	0.295	0.272	1.61E-03	1.125	0.037	1.046	1.210	0.169	Imputed
rs79812684	2	151,782,922	T	A	0.059	0.054	0.210	1.095	0.073	0.950	1.263	0.127	Imputed
chr2:151782944:D	2	151,782,944	AT	A	0.074	0.075	0.836	0.987	0.065	0.869	1.120	0.557	Imputed
rs16828997	2	151,783,005	G	A	0.050	0.047	0.331	1.079	0.078	0.926	1.257	0.321	Genotyped
rs2341926	2	151,783,928	G	A	0.295	0.272	1.56E-03	1.125	0.037	1.046	1.210	0.164	Genotyped
rs12618117	2	151,784,547	T	G	0.050	0.047	0.411	1.066	0.078	0.915	1.243	0.302	Genotyped
rs1548633	2	151,785,107	T	G	0.074	0.075	0.842	0.987	0.065	0.870	1.120	0.577	Imputed
rs62167121	2	151,785,119	C	G	0.048	0.044	0.253	1.095	0.080	0.937	1.280	0.271	Imputed
rs12612667	2	151,785,153	G	C	0.050	0.047	0.367	1.073	0.078	0.921	1.251	0.303	Imputed
rs62168565	2	151,785,173	T	C	0.050	0.047	0.391	1.069	0.078	0.918	1.246	0.291	Imputed
rs139844921	2	151,785,203	T	C	0.015	0.015	0.877	0.979	0.141	0.743	1.289	0.695	Imputed
rs1465234	2	151,785,370	A	C	0.074	0.075	0.865	0.989	0.064	0.872	1.122	0.544	Genotyped
rs1465235	2	151,785,424	C	G	0.295	0.272	1.57E-03	1.125	0.037	1.046	1.210	0.150	Imputed
rs57833369	2	151,785,426	C	T	0.102	0.098	0.604	1.030	0.056	0.922	1.149	0.661	Imputed
chr2:151785489:D	2	151,785,489	G	GA	0.050	0.047	0.391	1.069	0.078	0.918	1.246	0.291	Imputed
rs12465911	2	151,785,742	T	C	0.295	0.272	1.51E-03	1.125	0.037	1.046	1.211	0.138	Genotyped
rs114461029	2	151,785,940	T	A	0.014	0.014	0.753	1.046	0.143	0.791	1.383	0.966	Imputed
rs12613613	2	151,786,131	T	C	0.050	0.047	0.367	1.073	0.078	0.921	1.251	0.303	Imputed
rs7568567	2	151,786,203	C	G	0.520	0.492	7.72E-04	1.121	0.034	1.049	1.197	0.093	Imputed
rs10187523	2	151,786,649	C	A	0.295	0.272	1.57E-03	1.125	0.037	1.046	1.210	0.150	Imputed
chr2:151786670:I	2	151,786,670	C	CA	0.074	0.075	0.831	0.986	0.065	0.869	1.119	0.582	Imputed
rs7588329	2	151,786,791	C	T	0.113	0.108	0.381	1.048	0.054	0.944	1.164	0.806	Imputed
rs62168566	2	151,786,921	A	C	0.050	0.047	0.396	1.069	0.078	0.917	1.246	0.289	Imputed

rs59981233	2	151,786,930	C	G	0.087	0.087	0.835	1.013	0.060	0.901	1.139	0.507	Imputed
rs62168568	2	151,787,063	G	A	0.050	0.047	0.436	1.063	0.078	0.912	1.239	0.271	Imputed
rs13035974	2	151,787,101	G	A	0.273	0.299	3.64E-04	0.874	0.038	0.811	0.941	0.216	Imputed
rs192627409	2	151,787,195	A	T	0.078	0.074	0.470	1.047	0.063	0.925	1.186	0.880	Imputed
rs62168569	2	151,787,408	G	A	0.050	0.047	0.396	1.069	0.078	0.917	1.246	0.289	Imputed
rs60795199	2	151,787,666	A	T	0.101	0.098	0.631	1.027	0.056	0.920	1.147	0.646	Imputed
rs62168570	2	151,787,830	G	T	0.050	0.047	0.415	1.066	0.078	0.915	1.241	0.202	Imputed
rs7592542	2	151,788,206	C	T	0.198	0.197	0.816	1.010	0.042	0.929	1.098	0.765	Imputed
rs10204953	2	151,788,701	A	G	0.295	0.272	1.55E-03	1.125	0.037	1.046	1.210	0.150	Imputed
chr2:151788762:D	2	151,788,762	C	CA	0.101	0.098	0.614	1.029	0.056	0.922	1.149	0.634	Imputed
rs13005612	2	151,789,125	C	T	0.282	0.310	1.51E-04	0.868	0.037	0.807	0.934	0.134	Imputed
rs62168571	2	151,789,371	G	T	0.050	0.047	0.426	1.064	0.078	0.913	1.241	0.276	Imputed
rs72864611	2	151,789,681	C	T	0.057	0.053	0.203	1.098	0.073	0.951	1.268	0.992	Imputed
rs4452138	2	151,790,156	G	T	0.101	0.099	0.662	1.025	0.056	0.918	1.144	0.650	Imputed
rs116493102	2	151,790,167	G	C	0.028	0.030	0.543	0.940	0.102	0.769	1.148	0.375	Imputed
rs12621310	2	151,790,497	C	T	0.100	0.098	0.731	1.020	0.056	0.913	1.139	0.603	Imputed
rs56287485	2	151,791,220	T	C	0.176	0.178	0.772	0.987	0.044	0.905	1.077	0.620	Imputed
rs62168572	2	151,791,257	T	G	0.050	0.047	0.396	1.069	0.078	0.917	1.246	0.289	Imputed
rs76493088	2	151,791,565	T	A	0.050	0.047	0.426	1.064	0.078	0.913	1.241	0.276	Imputed
rs74738399	2	151,792,333	C	T	0.050	0.047	0.396	1.069	0.078	0.917	1.246	0.289	Imputed
rs58829155	2	151,792,602	A	G	0.101	0.099	0.662	1.025	0.056	0.918	1.144	0.650	Imputed
rs78552905	2	151,792,625	A	G	0.092	0.089	0.633	1.029	0.059	0.917	1.154	0.501	Imputed
rs74388543	2	151,793,106	C	T	0.104	0.104	0.998	1.000	0.055	0.897	1.115	0.634	Imputed
rs41478047	2	151,793,335	A	C	0.050	0.047	0.396	1.069	0.078	0.917	1.246	0.289	Imputed
rs77886955	2	151,793,449	C	G	0.050	0.047	0.426	1.064	0.078	0.913	1.241	0.276	Imputed
rs10930342	2	151,793,627	T	C	0.151	0.146	0.408	1.040	0.047	0.948	1.141	0.292	Genotyped
rs75894524	2	151,794,451	T	G	0.046	0.052	0.124	0.884	0.080	0.756	1.034	0.751	Imputed
rs62168573	2	151,794,619	C	T	0.048	0.044	0.269	1.092	0.080	0.934	1.277	0.262	Imputed
rs61394001	2	151,794,928	C	A	0.151	0.145	0.397	1.041	0.047	0.949	1.142	0.298	Imputed
rs77286775	2	151,795,295	C	T	0.100	0.098	0.722	1.020	0.056	0.913	1.140	0.608	Imputed
chr2:151795949:I	2	151,795,949	AC	A	0.153	0.145	0.224	1.059	0.047	0.966	1.162	0.375	Imputed
rs13031138	2	151,796,283	C	T	0.283	0.310	1.92E-04	0.870	0.037	0.808	0.936	0.130	Imputed
chr2:151797099:D	2	151,797,099	T	TG	0.197	0.195	0.708	1.016	0.043	0.935	1.104	0.755	Imputed
chr2:151797100:D	2	151,797,100	G	GC	0.198	0.197	0.764	1.013	0.042	0.932	1.101	0.727	Imputed
rs76993339	2	151,797,579	T	A	0.153	0.145	0.210	1.061	0.047	0.967	1.164	0.386	Imputed
rs6759103	2	151,797,838	T	A	0.489	0.469	9.12E-03	1.092	0.034	1.022	1.167	0.251	Imputed
rs78579421	2	151,797,873	C	T	0.050	0.047	0.396	1.069	0.078	0.917	1.246	0.289	Imputed
rs75749028	2	151,797,914	A	C	0.050	0.047	0.396	1.069	0.078	0.917	1.246	0.289	Imputed
rs10201074	2	151,798,095	C	T	0.074	0.075	0.832	0.987	0.064	0.869	1.119	0.494	Genotyped
rs62168576	2	151,798,878	T	C	0.153	0.145	0.190	1.064	0.047	0.970	1.167	0.390	Imputed

chr2:151799152:D	2	151,799,152	T	TG	0.104	0.104	0.956	1.003	0.055	0.900	1.118	0.632	Imputed
rs6433074	2	151,799,393	A	G	0.489	0.468	8.71E-03	1.093	0.034	1.023	1.168	0.222	Imputed
rs151281982	2	151,799,590	G	C	0.013	0.016	0.088	0.775	0.150	0.578	1.039	0.778	Imputed
rs62168577	2	151,800,498	G	A	0.155	0.147	0.169	1.067	0.047	0.973	1.169	0.516	Imputed
rs12996797	2	151,800,673	T	C	0.283	0.311	2.09E-04	0.871	0.037	0.809	0.937	0.110	Imputed
rs10168747	2	151,800,685	A	G	0.489	0.467	6.28E-03	1.097	0.034	1.026	1.172	0.211	Imputed
chr2:151800699:I	2	151,800,699	CA	C	0.013	0.010	0.079	1.306	0.153	0.968	1.761	0.706	Imputed
rs62168578	2	151,801,252	G	C	0.153	0.146	0.212	1.061	0.047	0.967	1.163	0.399	Imputed
rs4664214	2	151,801,350	C	A	0.282	0.310	1.73E-04	0.869	0.037	0.808	0.935	0.117	Imputed
chr2:151801743:D	2	151,801,743	T	TGTCA	0.050	0.047	0.421	1.065	0.078	0.914	1.242	0.278	Imputed
rs77764596	2	151,801,747	T	A	0.103	0.099	0.402	1.048	0.056	0.939	1.169	0.798	Imputed
rs62168579	2	151,801,868	T	G	0.050	0.047	0.391	1.070	0.078	0.917	1.247	0.291	Imputed
rs62168580	2	151,802,020	C	G	0.153	0.146	0.206	1.061	0.047	0.968	1.164	0.404	Imputed
chr2:151802047:I	2	151,802,047	CGT	C	0.153	0.146	0.206	1.061	0.047	0.968	1.164	0.404	Imputed
chr2:151802082:D	2	151,802,082	GTA	G	0.165	0.164	0.932	1.004	0.046	0.918	1.098	0.524	Imputed
rs1019034	2	151,802,403	C	T	0.074	0.075	0.828	0.986	0.064	0.869	1.119	0.539	Imputed
rs74790846	2	151,802,462	C	T	0.011	0.011	0.863	1.029	0.164	0.747	1.417	0.739	Imputed
rs1385430	2	151,802,790	C	T	0.198	0.197	0.783	1.012	0.042	0.931	1.100	0.747	Genotyped
rs1019035	2	151,802,844	T	C	0.437	0.457	0.011	0.917	0.034	0.858	0.980	0.311	Genotyped
rs1962787	2	151,803,061	T	C	0.103	0.099	0.413	1.047	0.056	0.938	1.168	0.790	Imputed
rs1962788	2	151,803,201	C	T	0.436	0.456	0.011	0.917	0.034	0.858	0.980	0.320	Imputed
rs2341927	2	151,803,207	T	C	0.283	0.311	2.06E-04	0.871	0.037	0.809	0.937	0.085	Imputed
rs62168581	2	151,803,211	A	G	0.061	0.056	0.167	1.104	0.071	0.960	1.269	0.340	Imputed
rs1385429	2	151,803,908	G	T	0.283	0.312	1.52E-04	0.868	0.037	0.807	0.934	0.120	Imputed
rs1385428	2	151,803,951	T	C	0.437	0.456	0.016	0.921	0.034	0.862	0.985	0.341	Imputed
rs114453373	2	151,804,679	T	C	0.072	0.075	0.643	0.970	0.065	0.854	1.102	0.676	Imputed
rs115568833	2	151,804,719	C	T	0.015	0.013	0.230	1.180	0.139	0.898	1.551	0.032	Imputed
chr2:151805028:I	2	151,805,028	AAATAT	A	0.134	0.121	0.012	1.134	0.050	1.029	1.251	0.292	Imputed
chr2:151805030:D	2	151,805,030	A	ATTT	0.162	0.150	0.031	1.104	0.046	1.009	1.209	0.711	Imputed
rs1905749	2	151,805,117	C	G	0.157	0.147	0.130	1.073	0.047	0.979	1.176	0.371	Imputed
rs1905748	2	151,805,334	C	G	0.156	0.146	0.101	1.080	0.047	0.985	1.184	0.346	Imputed
rs146544019	2	151,805,375	T	C	0.013	0.016	0.156	0.813	0.146	0.611	1.082	0.707	Imputed
rs1905747	2	151,805,376	A	G	0.156	0.146	0.105	1.079	0.047	0.984	1.183	0.353	Imputed
rs1905746	2	151,805,415	G	T	0.157	0.147	0.091	1.082	0.047	0.988	1.186	0.368	Imputed
rs62168583	2	151,805,764	C	G	0.053	0.048	0.207	1.101	0.076	0.949	1.278	0.321	Imputed
chr2:151805933:I	2	151,805,933	AAC	A	0.153	0.144	0.125	1.075	0.047	0.980	1.179	0.274	Imputed
chr2:151805934:I	2	151,805,934	AC	A	0.149	0.141	0.186	1.065	0.048	0.970	1.169	0.328	Imputed
chr2:151805940:D	2	151,805,940	C	CAA	0.141	0.131	0.103	1.083	0.049	0.984	1.192	0.241	Imputed
chr2:151805942:I	2	151,805,942	AAC	A	0.144	0.135	0.110	1.080	0.048	0.983	1.188	0.319	Imputed
chr2:151805945:D	2	151,805,945	A	AC	0.157	0.151	0.340	1.046	0.047	0.954	1.146	0.239	Imputed

chr2:151805946:D	2	151,805,946	C	CA	0.142	0.132	0.085	1.088	0.049	0.989	1.197	0.264	Imputed
chr2:151805955:D	2	151,805,955	C	CA	0.129	0.120	0.103	1.086	0.051	0.983	1.200	0.493	Imputed
rs74785527	2	151,805,969	C	A	0.145	0.141	0.550	1.029	0.049	0.936	1.132	0.655	Imputed
rs62168584	2	151,806,054	G	T	0.157	0.148	0.151	1.069	0.047	0.976	1.172	0.390	Imputed
chr2:151806167:D	2	151,806,167	C	CACACACAG	0.157	0.147	0.133	1.073	0.047	0.979	1.176	0.382	Imputed
rs62168585	2	151,806,281	T	C	0.157	0.147	0.134	1.073	0.047	0.979	1.176	0.367	Imputed
chr2:151806369:D	2	151,806,369	G	GA	0.157	0.148	0.159	1.068	0.047	0.975	1.171	0.355	Imputed
rs75926049	2	151,806,679	A	G	0.157	0.147	0.134	1.073	0.047	0.979	1.176	0.367	Imputed
rs62168586	2	151,806,876	G	A	0.157	0.147	0.134	1.073	0.047	0.979	1.176	0.367	Imputed
rs62168587	2	151,807,072	G	A	0.157	0.147	0.134	1.073	0.047	0.979	1.176	0.367	Imputed
rs67547706	2	151,807,078	A	G	0.281	0.310	1.02E-04	0.865	0.037	0.804	0.931	0.087	Imputed
rs12988725	2	151,807,119	A	G	0.156	0.147	0.141	1.071	0.047	0.977	1.174	0.364	Imputed
rs12468125	2	151,807,299	G	A	0.157	0.147	0.134	1.073	0.047	0.979	1.176	0.367	Imputed
rs35967096	2	151,807,347	C	T	0.281	0.310	1.02E-04	0.865	0.037	0.804	0.931	0.087	Imputed
rs12464141	2	151,807,469	T	C	0.157	0.148	0.146	1.070	0.047	0.977	1.173	0.348	Imputed
rs62168588	2	151,807,659	C	T	0.157	0.147	0.137	1.072	0.047	0.978	1.175	0.377	Imputed
rs1117292	2	151,807,843	A	G	0.195	0.195	0.915	1.005	0.043	0.924	1.092	0.714	Imputed
rs12995446	2	151,807,975	T	G	0.282	0.311	1.50E-04	0.868	0.037	0.807	0.934	0.094	Imputed
rs7574935	2	151,808,308	C	T	0.157	0.147	0.136	1.072	0.047	0.978	1.175	0.378	Imputed
rs7575057	2	151,808,405	C	T	0.436	0.455	0.020	0.924	0.034	0.864	0.987	0.277	Imputed
rs7584238	2	151,808,406	A	G	0.154	0.144	0.113	1.078	0.047	0.983	1.182	0.441	Imputed
chr2:151808689:I	2	151,808,689	TA	T	0.161	0.152	0.125	1.073	0.046	0.981	1.175	0.410	Imputed
rs1601363	2	151,808,935	G	T	0.157	0.148	0.141	1.071	0.047	0.977	1.174	0.372	Imputed
rs1601362	2	151,809,043	T	C	0.157	0.147	0.135	1.073	0.047	0.979	1.175	0.380	Imputed
rs1601361	2	151,809,106	C	T	0.157	0.147	0.126	1.074	0.047	0.980	1.177	0.389	Imputed
rs78165640	2	151,809,144	C	G	0.021	0.018	0.157	1.187	0.121	0.937	1.504	0.313	Imputed
rs62168589	2	151,809,445	T	C	0.157	0.147	0.135	1.073	0.047	0.979	1.175	0.380	Imputed
rs62168590	2	151,809,534	T	G	0.157	0.147	0.135	1.073	0.047	0.979	1.175	0.380	Imputed
rs76357556	2	151,809,545	G	A	0.157	0.147	0.133	1.073	0.047	0.979	1.176	0.381	Imputed
rs12620637	2	151,809,638	C	G	0.157	0.147	0.133	1.073	0.047	0.979	1.176	0.381	Imputed
rs974619	2	151,809,689	T	G	0.195	0.195	0.945	1.003	0.043	0.922	1.091	0.696	Imputed
rs12618502	2	151,809,781	C	T	0.157	0.147	0.133	1.073	0.047	0.979	1.176	0.381	Imputed
rs12618522	2	151,809,804	C	T	0.182	0.173	0.160	1.064	0.044	0.976	1.160	0.546	Imputed
rs12617868	2	151,809,828	T	A	0.157	0.147	0.135	1.073	0.047	0.979	1.175	0.380	Imputed
rs10176466	2	151,810,248	T	C	0.283	0.262	4.00E-03	1.115	0.038	1.035	1.200	0.065	Imputed
rs6742534	2	151,810,276	A	G	0.153	0.146	0.243	1.057	0.047	0.963	1.159	0.338	Imputed
rs62168591	2	151,810,325	T	C	0.053	0.049	0.198	1.103	0.076	0.951	1.279	0.283	Imputed
rs13010850	2	151,810,577	A	G	0.281	0.310	1.03E-04	0.865	0.037	0.804	0.931	0.105	Imputed
rs188116282	2	151,810,700	T	C	0.009	0.012	0.110	0.755	0.177	0.534	1.067	0.819	Imputed
chr2:151810748:D	2	151,810,748	C	CCA	0.052	0.051	0.706	1.029	0.076	0.886	1.196	0.364	Imputed

rs62168592	2	151,810,937	A	G	0.157	0.148	0.144	1.071	0.047	0.977	1.173	0.447	Imputed
rs12478652	2	151,811,339	A	C	0.103	0.099	0.410	1.047	0.056	0.939	1.168	0.863	Imputed
rs12466694	2	151,811,350	C	A	0.157	0.147	0.137	1.072	0.047	0.978	1.175	0.456	Imputed
rs62168593	2	151,811,418	C	T	0.053	0.048	0.200	1.102	0.076	0.950	1.279	0.325	Imputed
chr2:151811558:D	2	151,811,558	A	AG	0.282	0.311	1.13E-04	0.866	0.037	0.804	0.932	0.113	Imputed
rs146099113	2	151,811,605	T	C	0.195	0.195	0.889	1.006	0.043	0.925	1.094	0.762	Imputed
rs62168594	2	151,811,638	A	G	0.156	0.147	0.140	1.072	0.047	0.978	1.174	0.490	Imputed
rs62168595	2	151,811,640	T	C	0.156	0.147	0.140	1.072	0.047	0.978	1.174	0.490	Imputed
rs62168596	2	151,811,773	T	C	0.053	0.048	0.194	1.104	0.076	0.951	1.281	0.330	Imputed
rs13035894	2	151,811,868	G	A	0.436	0.428	0.323	1.034	0.034	0.967	1.106	0.862	Imputed
rs145066188	2	151,811,881	T	C	0.026	0.023	0.159	1.164	0.108	0.943	1.436	0.540	Imputed
rs12692891	2	151,812,012	T	C	0.281	0.309	1.89E-04	0.870	0.037	0.808	0.936	0.115	Imputed
rs78390458	2	151,812,013	A	G	0.106	0.101	0.270	1.063	0.055	0.954	1.184	0.654	Imputed
rs62168621	2	151,812,101	A	T	0.161	0.151	0.100	1.079	0.046	0.986	1.181	0.519	Imputed
rs62168622	2	151,812,192	A	T	0.156	0.147	0.140	1.072	0.047	0.978	1.174	0.437	Imputed
rs62168623	2	151,812,210	T	C	0.053	0.048	0.207	1.101	0.076	0.949	1.278	0.321	Imputed
rs62168624	2	151,812,243	A	T	0.157	0.147	0.137	1.072	0.047	0.978	1.175	0.456	Imputed
rs62168625	2	151,812,550	G	C	0.053	0.048	0.194	1.104	0.076	0.951	1.281	0.330	Imputed
rs74357710	2	151,812,565	A	G	0.053	0.049	0.216	1.099	0.076	0.947	1.275	0.313	Imputed
rs77098704	2	151,812,819	T	C	0.157	0.147	0.137	1.072	0.047	0.978	1.175	0.456	Imputed
rs62168626	2	151,812,900	T	C	0.156	0.147	0.144	1.071	0.047	0.977	1.174	0.453	Imputed
rs141390915	2	151,813,192	G	C	0.025	0.022	0.220	1.143	0.109	0.923	1.415	0.603	Imputed
rs116629669	2	151,813,194	T	A	0.011	0.011	0.920	1.017	0.163	0.738	1.400	0.842	Imputed
rs62168627	2	151,813,333	A	G	0.157	0.148	0.122	1.075	0.047	0.981	1.178	0.485	Imputed
rs62168628	2	151,813,421	G	A	0.157	0.148	0.122	1.075	0.047	0.981	1.178	0.485	Imputed
rs79744562	2	151,813,730	T	C	0.158	0.148	0.117	1.076	0.047	0.982	1.179	0.508	Imputed
rs62168629	2	151,813,788	G	C	0.157	0.148	0.122	1.075	0.047	0.981	1.178	0.485	Imputed
rs4664216	2	151,813,988	T	C	0.074	0.075	0.767	0.981	0.065	0.865	1.113	0.566	Imputed
rs185448817	2	151,813,989	A	G	0.053	0.048	0.206	1.102	0.076	0.948	1.280	0.304	Imputed
rs116152396	2	151,813,999	T	A	0.157	0.148	0.122	1.075	0.047	0.981	1.178	0.485	Imputed
rs145252607	2	151,814,060	C	T	0.157	0.148	0.120	1.075	0.047	0.981	1.178	0.488	Imputed
rs147581596	2	151,814,073	C	T	0.157	0.147	0.124	1.075	0.047	0.981	1.178	0.473	Imputed
rs149061531	2	151,814,075	A	G	0.157	0.147	0.124	1.075	0.047	0.981	1.178	0.473	Imputed
rs142134892	2	151,814,207	A	G	0.195	0.195	0.891	1.006	0.043	0.925	1.094	0.713	Imputed
rs13028601	2	151,814,523	T	A	0.282	0.310	1.12E-04	0.866	0.037	0.804	0.931	0.110	Imputed
rs12620371	2	151,814,636	C	G	0.053	0.048	0.183	1.107	0.076	0.954	1.284	0.339	Imputed
rs77065590	2	151,814,652	A	C	0.104	0.099	0.364	1.052	0.056	0.943	1.173	0.911	Imputed
rs4664841	2	151,814,776	T	C	0.205	0.203	0.693	1.017	0.042	0.937	1.104	0.875	Imputed
rs12614295	2	151,814,911	T	C	0.157	0.147	0.105	1.079	0.047	0.984	1.182	0.478	Imputed
rs4664217	2	151,814,912	A	G	0.195	0.194	0.897	1.006	0.043	0.925	1.093	0.776	Imputed

rs12614304	2	151,814,974	T	C	0.157	0.147	0.105	1.079	0.047	0.984	1.182	0.478	Imputed
rs2108198	2	151,815,243	A	C	0.282	0.311	1.09E-04	0.865	0.037	0.804	0.931	0.111	Imputed
rs78114223	2	151,815,346	G	A	0.158	0.147	0.097	1.081	0.047	0.986	1.184	0.492	Imputed
rs2108199	2	151,815,388	T	C	0.281	0.310	1.22E-04	0.866	0.037	0.805	0.932	0.100	Imputed
rs62168630	2	151,815,447	T	C	0.160	0.150	0.100	1.079	0.046	0.986	1.182	0.431	Imputed
rs62168631	2	151,815,546	G	A	0.053	0.048	0.163	1.112	0.076	0.958	1.290	0.313	Imputed
rs57189116	2	151,815,660	A	C	0.158	0.147	0.097	1.081	0.047	0.986	1.184	0.492	Imputed
rs57031532	2	151,815,771	G	A	0.158	0.147	0.097	1.081	0.047	0.986	1.184	0.492	Imputed
rs56985368	2	151,815,899	T	C	0.157	0.147	0.107	1.078	0.047	0.984	1.182	0.498	Imputed
rs59769602	2	151,815,932	G	A	0.157	0.147	0.118	1.076	0.047	0.982	1.179	0.514	Imputed
rs13016337	2	151,816,062	A	C	0.282	0.311	1.09E-04	0.865	0.037	0.804	0.931	0.111	Imputed
rs62168636	2	151,816,086	T	C	0.053	0.048	0.148	1.116	0.076	0.962	1.295	0.327	Imputed
rs61658188	2	151,816,133	A	G	0.158	0.147	0.097	1.081	0.047	0.986	1.184	0.492	Imputed
rs11680426	2	151,816,175	G	C	0.198	0.198	0.940	1.003	0.042	0.923	1.090	0.627	Imputed
rs13016725	2	151,816,192	T	C	0.282	0.311	1.20E-04	0.866	0.037	0.805	0.932	0.108	Imputed
rs12468172	2	151,816,279	T	C	0.158	0.147	0.097	1.081	0.047	0.986	1.184	0.492	Imputed
rs12473256	2	151,816,285	C	T	0.158	0.147	0.098	1.080	0.047	0.986	1.184	0.490	Imputed
rs6717928	2	151,816,429	T	C	0.194	0.194	0.822	1.010	0.043	0.929	1.098	0.830	Imputed
rs12472474	2	151,816,432	G	A	0.158	0.147	0.097	1.081	0.047	0.986	1.184	0.492	Imputed
rs12473325	2	151,816,493	C	T	0.158	0.147	0.098	1.080	0.047	0.986	1.184	0.490	Imputed
rs13003635	2	151,816,753	C	T	0.291	0.319	1.03E-04	0.866	0.037	0.805	0.931	0.114	Genotyped
rs12998436	2	151,816,764	G	A	0.291	0.319	1.02E-04	0.866	0.037	0.805	0.931	0.115	Genotyped
rs62168640	2	151,816,783	T	C	0.283	0.263	5.33E-03	1.111	0.038	1.032	1.196	0.081	Imputed
rs6735966	2	151,816,873	G	A	0.194	0.194	0.888	1.006	0.043	0.925	1.094	0.778	Imputed
rs62168641	2	151,816,900	T	A	0.053	0.048	0.163	1.112	0.076	0.958	1.290	0.313	Imputed
rs13022902	2	151,816,919	A	C	0.290	0.318	1.60E-04	0.869	0.037	0.808	0.935	0.114	Imputed
rs13022926	2	151,816,956	G	C	0.282	0.311	1.24E-04	0.866	0.037	0.805	0.932	0.107	Imputed
rs13023089	2	151,817,016	G	C	0.282	0.311	1.22E-04	0.866	0.037	0.805	0.932	0.107	Imputed
rs2341928	2	151,817,160	A	G	0.281	0.309	1.62E-04	0.868	0.038	0.807	0.935	0.086	Imputed
rs2341929	2	151,817,179	C	A	0.281	0.309	1.55E-04	0.868	0.037	0.806	0.934	0.117	Imputed
rs2341930	2	151,817,189	T	A	0.281	0.309	1.67E-04	0.869	0.037	0.807	0.935	0.115	Imputed
rs2341931	2	151,817,207	C	T	0.289	0.318	1.09E-04	0.866	0.037	0.805	0.932	0.118	Imputed
rs149555449	2	151,817,448	T	C	0.025	0.023	0.570	1.065	0.110	0.858	1.322	0.017	Imputed
rs6739724	2	151,817,463	C	T	0.352	0.341	0.147	1.053	0.035	0.982	1.129	0.823	Imputed
rs6752773	2	151,817,664	A	G	0.348	0.338	0.181	1.049	0.036	0.978	1.124	0.786	Imputed
rs10185069	2	151,817,875	T	C	0.283	0.263	5.47E-03	1.110	0.038	1.031	1.195	0.084	Imputed
rs13404975	2	151,818,420	T	C	0.129	0.119	0.033	1.115	0.051	1.009	1.231	0.609	Imputed
rs1843275	2	151,818,629	C	T	0.352	0.342	0.158	1.051	0.035	0.981	1.127	0.784	Imputed
rs1843274	2	151,818,743	G	C	0.191	0.191	0.854	1.008	0.043	0.927	1.097	0.816	Imputed
rs12471652	2	151,819,095	C	T	0.362	0.343	0.011	1.094	0.035	1.021	1.173	0.202	Imputed

rs12475783	2	151,819,115	C	A	0.352	0.342	0.159	1.051	0.035	0.981	1.127	0.783	Imputed
rs34018967	2	151,819,233	G	T	0.282	0.310	1.07E-04	0.865	0.037	0.804	0.931	0.115	Imputed
rs10188901	2	151,819,277	T	C	0.459	0.443	0.056	1.067	0.034	0.999	1.141	0.959	Imputed
rs12986591	2	151,819,319	A	G	0.282	0.310	1.17E-04	0.866	0.037	0.805	0.932	0.113	Imputed
chr2:151819342:D	2	151,819,342	T	TTA	0.104	0.099	0.382	1.050	0.056	0.941	1.171	0.827	Imputed
rs111364945	2	151,819,406	C	T	0.103	0.099	0.396	1.048	0.056	0.940	1.170	0.793	Imputed
rs6744706	2	151,819,530	G	A	0.346	0.339	0.311	1.037	0.036	0.967	1.112	0.949	Imputed
rs62168642	2	151,819,596	G	A	0.046	0.044	0.453	1.063	0.081	0.907	1.245	0.438	Imputed
rs6747878	2	151,819,617	C	T	0.346	0.337	0.261	1.041	0.036	0.971	1.116	0.863	Imputed
rs6760882	2	151,819,700	T	G	0.352	0.341	0.152	1.052	0.035	0.982	1.128	0.765	Imputed
rs6733259	2	151,819,756	T	C	0.354	0.342	0.104	1.059	0.035	0.988	1.135	0.829	Imputed
chr2:151820095:D	2	151,820,095	A	AC	0.103	0.099	0.389	1.049	0.056	0.941	1.170	0.798	Imputed
rs6733693	2	151,820,098	C	A	0.358	0.339	0.013	1.092	0.035	1.019	1.170	0.210	Imputed
rs62168643	2	151,820,253	A	G	0.250	0.244	0.387	1.034	0.039	0.958	1.117	0.813	Imputed
rs66919845	2	151,820,282	G	A	0.290	0.319	8.54E-05	0.864	0.037	0.804	0.930	0.125	Imputed
rs72485577	2	151,820,540	T	G	0.195	0.194	0.818	1.010	0.043	0.929	1.098	0.810	Imputed
rs62168644	2	151,820,660	C	T	0.358	0.339	0.015	1.090	0.035	1.017	1.168	0.219	Imputed
rs10200264	2	151,820,858	C	G	0.284	0.264	5.18E-03	1.111	0.038	1.032	1.196	0.089	Imputed
rs6716456	2	151,821,242	T	C	0.352	0.341	0.171	1.050	0.035	0.979	1.125	0.752	Genotyped
rs13396805	2	151,821,512	T	C	0.284	0.264	5.11E-03	1.111	0.038	1.032	1.196	0.086	Genotyped
rs72864637	2	151,821,583	A	G	0.067	0.058	0.024	1.167	0.068	1.021	1.335	0.985	Imputed
rs1859722	2	151,821,832	A	G	0.290	0.319	8.28E-05	0.864	0.037	0.804	0.929	0.126	Imputed
rs60771511	2	151,822,080	A	C	0.352	0.341	0.169	1.050	0.035	0.980	1.126	0.741	Imputed
rs74879888	2	151,822,161	C	A	0.104	0.099	0.368	1.051	0.056	0.943	1.173	0.814	Imputed
rs61024762	2	151,822,200	T	C	0.344	0.334	0.219	1.045	0.036	0.974	1.120	0.996	Imputed
rs75351679	2	151,822,217	T	C	0.197	0.197	0.873	1.007	0.043	0.926	1.094	0.774	Imputed
rs57381291	2	151,822,241	G	A	0.352	0.341	0.169	1.050	0.035	0.980	1.126	0.741	Imputed
rs13003276	2	151,822,544	C	T	0.275	0.304	8.91E-05	0.863	0.038	0.801	0.929	0.210	Imputed
rs72995957	2	151,822,677	T	G	0.352	0.341	0.166	1.050	0.035	0.980	1.126	0.696	Imputed
chr2:151822691:D	2	151,822,691	T	TACCC	0.276	0.304	2.19E-04	0.870	0.038	0.808	0.937	0.123	Imputed
rs56992483	2	151,822,692	T	A	0.282	0.310	1.24E-04	0.866	0.037	0.805	0.932	0.146	Imputed
rs58825049	2	151,822,693	T	C	0.280	0.308	1.37E-04	0.867	0.038	0.805	0.933	0.176	Imputed
rs114838140	2	151,822,694	T	C	0.280	0.308	1.46E-04	0.867	0.038	0.806	0.933	0.179	Imputed
rs56233126	2	151,822,888	G	A	0.352	0.342	0.180	1.049	0.035	0.978	1.124	0.727	Imputed
rs13026292	2	151,822,914	G	A	0.358	0.339	0.014	1.090	0.035	1.017	1.169	0.224	Imputed
rs55769614	2	151,823,147	T	G	0.352	0.341	0.178	1.049	0.035	0.979	1.124	0.780	Imputed
rs16829051	2	151,823,389	T	C	0.193	0.194	0.982	1.001	0.043	0.920	1.089	0.771	Imputed
rs116388321	2	151,823,542	T	G	0.039	0.039	0.994	0.999	0.088	0.842	1.186	0.606	Imputed
rs1485104	2	151,823,799	C	T	0.358	0.339	0.014	1.090	0.035	1.017	1.169	0.224	Imputed
rs62168649	2	151,824,144	G	T	0.352	0.341	0.178	1.049	0.035	0.979	1.124	0.780	Imputed

rs1485105	2	151,824,604	G	A	0.358	0.339	0.014	1.090	0.035	1.017	1.169	0.224	Imputed
rs10930349	2	151,824,774	T	A	0.363	0.346	0.022	1.084	0.035	1.011	1.161	0.265	Imputed
rs74418962	2	151,824,998	A	G	0.083	0.087	0.379	0.948	0.061	0.841	1.068	0.243	Imputed
chr2:151825028:D	2	151,825,028	TATC	T	0.394	0.379	0.065	1.066	0.035	0.996	1.142	0.324	Imputed
rs12992519	2	151,825,093	C	T	0.358	0.339	0.014	1.090	0.035	1.017	1.169	0.224	Imputed
rs12991256	2	151,825,201	A	G	0.290	0.319	9.33E-05	0.865	0.037	0.804	0.930	0.122	Imputed
rs12992982	2	151,825,296	T	C	0.281	0.310	1.19E-04	0.866	0.037	0.805	0.932	0.121	Genotyped
rs13024197	2	151,825,473	T	C	0.358	0.339	0.014	1.090	0.035	1.017	1.169	0.224	Imputed
rs13024552	2	151,825,812	A	G	0.358	0.339	0.014	1.090	0.035	1.017	1.169	0.224	Imputed
rs12999071	2	151,825,870	C	T	0.358	0.339	0.014	1.090	0.035	1.017	1.169	0.224	Imputed
chr2:151826299:I	2	151,826,299	TA	T	0.281	0.309	1.82E-04	0.869	0.038	0.808	0.936	0.118	Imputed
rs34113888	2	151,826,300	A	T	0.280	0.308	1.66E-04	0.868	0.038	0.807	0.935	0.118	Imputed
rs10930350	2	151,826,543	C	G	0.368	0.349	0.011	1.094	0.035	1.021	1.172	0.161	Imputed
rs4664845	2	151,826,705	T	C	0.358	0.340	0.016	1.089	0.035	1.016	1.167	0.238	Imputed
rs4664846	2	151,826,857	A	G	0.358	0.340	0.016	1.089	0.035	1.016	1.167	0.237	Imputed
rs1586257	2	151,827,171	G	T	0.358	0.340	0.016	1.089	0.035	1.016	1.167	0.238	Imputed
rs1586258	2	151,827,330	A	G	0.370	0.356	0.088	1.062	0.035	0.991	1.137	0.702	Imputed
rs1586259	2	151,827,457	C	T	0.358	0.340	0.016	1.089	0.035	1.016	1.167	0.238	Imputed
rs2079453	2	151,827,583	A	G	0.074	0.076	0.763	0.981	0.064	0.864	1.113	0.462	Imputed
rs1601354	2	151,827,644	A	G	0.358	0.340	0.016	1.089	0.035	1.016	1.167	0.238	Imputed
rs10200786	2	151,827,909	T	C	0.044	0.044	0.856	1.015	0.083	0.863	1.194	0.576	Imputed
rs1601355	2	151,827,918	C	A	0.358	0.340	0.016	1.089	0.035	1.016	1.167	0.238	Imputed
rs1601356	2	151,828,009	A	G	0.290	0.319	9.64E-05	0.865	0.037	0.805	0.931	0.130	Imputed
rs1601357	2	151,828,034	C	A	0.358	0.340	0.016	1.089	0.035	1.016	1.167	0.238	Imputed
rs62169389	2	151,828,195	T	C	0.053	0.048	0.158	1.113	0.076	0.959	1.292	0.340	Imputed
rs12990001	2	151,828,296	A	G	0.358	0.340	0.016	1.089	0.035	1.016	1.167	0.238	Imputed
rs12995627	2	151,828,436	T	C	0.358	0.340	0.016	1.089	0.035	1.016	1.167	0.245	Genotyped
rs13018464	2	151,828,708	A	G	0.291	0.318	2.72E-04	0.874	0.037	0.812	0.940	0.219	Imputed
rs62169390	2	151,828,757	A	G	0.053	0.048	0.158	1.113	0.076	0.959	1.292	0.340	Imputed
rs150389192	2	151,829,167	T	A	0.014	0.013	0.584	1.082	0.144	0.816	1.437	0.624	Imputed
rs1843270	2	151,829,332	C	T	0.358	0.340	0.016	1.089	0.035	1.016	1.167	0.238	Imputed
chr2:151829743:D	2	151,829,743	T	TA	0.282	0.310	1.83E-04	0.869	0.037	0.808	0.936	0.152	Imputed
rs1485106	2	151,829,773	C	T	0.369	0.352	0.027	1.081	0.035	1.009	1.158	0.186	Imputed
rs6752241	2	151,829,779	T	C	0.105	0.099	0.260	1.064	0.055	0.955	1.187	0.885	Imputed
rs6752383	2	151,829,891	C	T	0.358	0.340	0.016	1.089	0.035	1.016	1.167	0.238	Imputed
rs6709868	2	151,829,956	A	G	0.358	0.340	0.016	1.089	0.035	1.016	1.167	0.238	Imputed
rs6713084	2	151,830,040	A	T	0.352	0.341	0.157	1.051	0.035	0.981	1.127	0.742	Imputed
rs13009887	2	151,830,488	T	G	0.359	0.341	0.022	1.084	0.035	1.012	1.162	0.265	Imputed
rs1024553	2	151,830,515	A	G	0.074	0.076	0.753	0.980	0.064	0.864	1.112	0.447	Imputed
rs62169391	2	151,830,594	T	C	0.053	0.048	0.158	1.113	0.076	0.959	1.292	0.340	Imputed

rs12473058	2	151,830,676	G	C	0.352	0.341	0.152	1.052	0.035	0.982	1.128	0.751	Imputed
rs12473114	2	151,830,834	C	T	0.358	0.340	0.016	1.089	0.035	1.016	1.167	0.236	Imputed
rs2108200	2	151,830,864	A	G	0.363	0.344	0.012	1.092	0.035	1.019	1.170	0.249	Imputed
rs1024554	2	151,831,081	A	C	0.358	0.340	0.016	1.089	0.035	1.016	1.167	0.236	Imputed
chr2:151831223:D	2	151,831,223	ATCTT	A	0.361	0.343	0.018	1.087	0.035	1.014	1.165	0.278	Imputed
rs13023613	2	151,831,360	G	A	0.290	0.319	9.95E-05	0.866	0.037	0.805	0.931	0.129	Imputed
rs13001965	2	151,831,642	A	G	0.292	0.321	1.25E-04	0.868	0.037	0.807	0.933	0.128	Imputed
rs16829081	2	151,831,643	C	T	0.053	0.048	0.158	1.113	0.076	0.959	1.292	0.340	Imputed
rs12478344	2	151,831,748	G	A	0.352	0.341	0.156	1.052	0.035	0.981	1.127	0.744	Imputed
rs1872388	2	151,831,926	A	G	0.358	0.340	0.016	1.089	0.035	1.016	1.167	0.235	Imputed
rs74324250	2	151,832,138	G	C	0.016	0.013	0.223	1.182	0.137	0.903	1.547	0.643	Imputed
rs13030344	2	151,832,187	C	A	0.282	0.310	1.86E-04	0.870	0.037	0.808	0.936	0.146	Imputed
rs13030392	2	151,832,252	G	A	0.281	0.310	1.10E-04	0.865	0.037	0.804	0.931	0.167	Imputed
chr2:151832346:D	2	151,832,346	C	CT	0.352	0.342	0.160	1.051	0.035	0.981	1.127	0.703	Imputed
rs141483103	2	151,832,407	T	C	0.027	0.028	0.862	0.982	0.104	0.802	1.203	0.572	Imputed
rs16829095	2	151,832,511	G	A	0.290	0.319	1.01E-04	0.866	0.037	0.805	0.931	0.128	Genotyped
rs6433094	2	151,832,608	C	T	0.358	0.340	0.016	1.089	0.035	1.016	1.167	0.235	Imputed
rs13009161	2	151,832,653	A	G	0.290	0.319	9.95E-05	0.866	0.037	0.805	0.931	0.129	Imputed
rs13009389	2	151,832,764	A	G	0.290	0.319	9.95E-05	0.866	0.037	0.805	0.931	0.129	Imputed
rs62169392	2	151,832,796	T	C	0.053	0.048	0.158	1.113	0.076	0.959	1.292	0.340	Imputed
rs6433095	2	151,832,922	A	G	0.358	0.340	0.016	1.089	0.035	1.016	1.167	0.235	Imputed
chr2:151833037:D	2	151,833,037	CATTA	C	0.359	0.342	0.022	1.084	0.035	1.012	1.162	0.237	Imputed
rs116541173	2	151,833,093	A	G	0.039	0.039	0.946	1.006	0.088	0.847	1.194	0.601	Imputed
rs12612286	2	151,833,423	T	C	0.359	0.341	0.018	1.087	0.035	1.014	1.165	0.254	Imputed
rs1485107	2	151,833,507	T	A	0.358	0.340	0.016	1.089	0.035	1.016	1.167	0.243	Imputed
rs1485108	2	151,833,729	T	C	0.358	0.340	0.016	1.089	0.035	1.016	1.167	0.243	Imputed
rs72995984	2	151,834,158	C	G	0.352	0.341	0.168	1.050	0.035	0.980	1.126	0.793	Imputed
rs1905743	2	151,834,211	A	G	0.358	0.340	0.016	1.089	0.035	1.016	1.167	0.243	Imputed
rs6708093	2	151,834,334	T	C	0.358	0.340	0.016	1.089	0.035	1.016	1.167	0.244	Genotyped
rs13019109	2	151,834,355	T	C	0.278	0.305	2.44E-04	0.871	0.038	0.809	0.938	0.244	Imputed
chr2:151834355:D	2	151,834,355	C	CT	0.306	0.329	2.02E-03	0.893	0.037	0.831	0.960	0.395	Imputed
rs13000291	2	151,834,356	T	C	0.361	0.345	0.036	1.077	0.035	1.005	1.154	0.265	Imputed
rs13019269	2	151,834,384	T	C	0.290	0.319	1.18E-04	0.867	0.037	0.806	0.932	0.151	Imputed
rs6718591	2	151,834,542	G	T	0.358	0.340	0.016	1.089	0.035	1.016	1.167	0.243	Imputed
rs1948021	2	151,835,309	G	T	0.359	0.341	0.017	1.088	0.035	1.016	1.166	0.265	Imputed
rs1948022	2	151,835,317	C	T	0.358	0.340	0.016	1.089	0.035	1.016	1.167	0.243	Imputed
rs1948023	2	151,835,393	G	A	0.358	0.340	0.016	1.089	0.035	1.016	1.167	0.243	Imputed
rs7563865	2	151,835,794	A	G	0.358	0.340	0.016	1.089	0.035	1.016	1.167	0.243	Imputed
rs116249011	2	151,835,865	G	T	0.022	0.021	0.595	1.063	0.115	0.848	1.332	0.635	Imputed
rs7563970	2	151,835,883	A	G	0.358	0.340	0.016	1.089	0.035	1.016	1.167	0.243	Imputed

rs2062365	2	151,835,990	T	C	0.358	0.340	0.016	1.089	0.035	1.016	1.167	0.243	Imputed
rs1905744	2	151,836,205	T	G	0.358	0.340	0.018	1.087	0.035	1.015	1.165	0.236	Imputed
rs6713372	2	151,836,383	G	A	0.105	0.099	0.239	1.067	0.055	0.958	1.190	0.906	Genotyped
rs6433098	2	151,836,651	A	G	0.358	0.340	0.017	1.088	0.035	1.015	1.166	0.238	Imputed
rs6433099	2	151,836,787	G	A	0.358	0.340	0.017	1.088	0.035	1.015	1.166	0.241	Genotyped
rs6433101	2	151,836,963	C	G	0.358	0.340	0.018	1.087	0.035	1.015	1.165	0.236	Imputed
rs6730077	2	151,836,980	A	G	0.105	0.099	0.255	1.065	0.055	0.956	1.187	0.890	Imputed
rs6720115	2	151,837,014	T	C	0.358	0.340	0.021	1.085	0.035	1.013	1.163	0.232	Imputed
chr2:151837280:D	2	151,837,280	G	GCTAC	0.290	0.319	1.30E-04	0.868	0.037	0.807	0.933	0.142	Imputed
chr2:151837284:D	2	151,837,284	C	CCTA	0.291	0.319	1.33E-04	0.868	0.037	0.807	0.933	0.141	Imputed
rs12989382	2	151,837,519	A	G	0.290	0.319	1.14E-04	0.867	0.037	0.806	0.932	0.147	Imputed
rs76701413	2	151,837,825	A	C	0.021	0.019	0.434	1.097	0.118	0.870	1.382	0.827	Imputed
rs13027517	2	151,837,961	C	T	0.291	0.320	1.19E-04	0.867	0.037	0.806	0.933	0.154	Imputed
rs13412460	2	151,838,205	A	G	0.282	0.263	7.03E-03	1.107	0.038	1.028	1.192	0.078	Imputed
rs62169393	2	151,838,421	G	A	0.282	0.263	7.03E-03	1.107	0.038	1.028	1.192	0.078	Imputed
rs34398830	2	151,838,656	G	C	0.283	0.311	1.57E-04	0.868	0.037	0.807	0.934	0.197	Imputed
rs13028980	2	151,838,855	T	A	0.283	0.311	1.97E-04	0.870	0.037	0.809	0.936	0.180	Imputed
rs13034876	2	151,838,995	C	T	0.283	0.311	1.97E-04	0.870	0.037	0.809	0.936	0.180	Imputed
rs34372555	2	151,839,088	A	G	0.284	0.313	1.36E-04	0.867	0.037	0.806	0.933	0.220	Imputed
rs34462888	2	151,839,100	C	T	0.284	0.313	1.36E-04	0.867	0.037	0.806	0.933	0.220	Imputed
rs187717422	2	151,839,104	T	G	0.021	0.018	0.148	1.191	0.121	0.940	1.510	0.284	Imputed
chr2:151839113:D	2	151,839,113	T	TTTGTA	0.348	0.338	0.200	1.047	0.036	0.976	1.122	0.896	Imputed
rs12619845	2	151,839,136	A	G	0.053	0.047	0.145	1.118	0.076	0.963	1.298	0.380	Imputed
rs990127	2	151,839,240	C	T	0.283	0.311	1.65E-04	0.869	0.037	0.807	0.935	0.195	Imputed
rs62169394	2	151,839,332	T	C	0.357	0.347	0.183	1.048	0.035	0.978	1.123	0.900	Imputed
rs1118980	2	151,839,884	G	T	0.369	0.351	0.025	1.082	0.035	1.010	1.159	0.195	Genotyped
rs62169395	2	151,839,922	C	G	0.053	0.048	0.153	1.115	0.076	0.961	1.294	0.345	Imputed
rs11679370	2	151,840,385	T	C	0.193	0.194	0.936	1.003	0.043	0.923	1.091	0.792	Imputed
rs1476667	2	151,840,526	T	C	0.102	0.096	0.238	1.069	0.056	0.957	1.193	0.918	Imputed
rs75634404	2	151,840,717	G	A	0.053	0.048	0.161	1.113	0.076	0.959	1.291	0.359	Imputed
rs1476668	2	151,840,729	A	G	0.102	0.096	0.240	1.068	0.056	0.957	1.192	0.940	Imputed
rs1476669	2	151,840,744	C	T	0.102	0.096	0.238	1.069	0.056	0.957	1.193	0.918	Imputed
rs141613741	2	151,841,000	T	G	0.039	0.039	0.982	1.002	0.088	0.844	1.189	0.702	Imputed
rs12623677	2	151,841,295	G	C	0.053	0.048	0.153	1.115	0.076	0.961	1.294	0.345	Imputed
chr2:151841369:D	2	151,841,369	G	GAT	0.102	0.096	0.218	1.072	0.056	0.960	1.196	0.858	Imputed
rs76579452	2	151,841,939	T	C	0.102	0.096	0.226	1.070	0.056	0.959	1.195	0.932	Imputed
rs62169396	2	151,842,536	C	G	0.051	0.045	0.108	1.133	0.078	0.973	1.320	0.185	Imputed
rs12994178	2	151,842,743	T	C	0.301	0.327	4.84E-04	0.880	0.037	0.819	0.945	0.290	Imputed
rs757919	2	151,842,851	T	C	0.194	0.194	0.902	1.005	0.043	0.924	1.093	0.855	Imputed
rs757920	2	151,843,023	G	A	0.194	0.194	0.909	1.005	0.043	0.924	1.093	0.841	Imputed

rs78705460	2	151,843,207	A	G	0.102	0.096	0.240	1.068	0.056	0.957	1.192	0.940	Imputed
rs1005086	2	151,843,217	T	C	0.384	0.407	4.18E-03	0.905	0.035	0.846	0.969	0.227	Imputed
rs57676538	2	151,843,725	A	G	0.250	0.244	0.379	1.035	0.039	0.959	1.118	0.787	Imputed
rs989414	2	151,843,978	T	C	0.078	0.079	0.770	0.982	0.063	0.868	1.111	0.427	Imputed
chr2:151844222:D	2	151,844,222	T	TAAA	0.282	0.311	1.49E-04	0.868	0.037	0.806	0.934	0.176	Imputed
rs148885277	2	151,844,612	G	C	0.015	0.014	0.484	1.103	0.140	0.839	1.451	0.676	Imputed
chr2:151844739:D	2	151,844,739	A	AT	0.012	0.013	0.811	0.963	0.156	0.710	1.307	0.417	Imputed
rs72866517	2	151,845,395	T	C	0.044	0.042	0.466	1.062	0.083	0.903	1.250	0.647	Imputed
rs2880193	2	151,845,617	C	T	0.102	0.096	0.264	1.065	0.056	0.954	1.189	0.916	Imputed
rs58808803	2	151,846,085	G	A	0.102	0.096	0.264	1.065	0.056	0.954	1.189	0.916	Imputed
rs1905745	2	151,846,133	G	A	0.248	0.242	0.402	1.033	0.039	0.957	1.116	0.819	Imputed
rs58558650	2	151,846,619	C	T	0.102	0.096	0.268	1.064	0.056	0.953	1.188	0.869	Imputed
rs79518869	2	151,846,749	A	G	0.102	0.096	0.260	1.065	0.056	0.954	1.190	0.877	Imputed
rs76440132	2	151,846,886	A	G	0.048	0.054	0.115	0.884	0.079	0.757	1.031	0.414	Imputed
rs149618885	2	151,846,914	C	T	0.012	0.016	0.063	0.756	0.151	0.563	1.016	0.707	Imputed
rs1601360	2	151,847,222	C	T	0.350	0.338	0.139	1.054	0.036	0.983	1.130	0.792	Imputed
rs72997773	2	151,847,727	T	C	0.102	0.096	0.313	1.058	0.056	0.948	1.182	0.893	Imputed
rs13006345	2	151,848,271	T	A	0.282	0.310	1.63E-04	0.869	0.037	0.807	0.935	0.172	Imputed
chr2:151848931:I	2	151,848,931	CA	C	0.254	0.251	0.642	1.018	0.039	0.943	1.099	0.686	Imputed
rs16829166	2	151,850,740	A	C	0.101	0.096	0.332	1.056	0.056	0.946	1.179	0.877	Imputed
rs6433104	2	151,850,866	T	C	0.078	0.079	0.793	0.984	0.063	0.869	1.113	0.418	Imputed
chr2:151851182:D	2	151,851,182	C	CATAA	0.053	0.048	0.146	1.117	0.076	0.963	1.297	0.330	Imputed
chr2:151851435:I	2	151,851,435	CA	C	0.107	0.102	0.266	1.063	0.055	0.955	1.184	0.791	Imputed
rs10178050	2	151,851,490	C	T	0.078	0.079	0.775	0.982	0.063	0.868	1.112	0.425	Imputed
rs72997777	2	151,851,879	G	A	0.193	0.194	0.974	1.001	0.043	0.921	1.089	0.910	Imputed
rs4664864	2	151,852,128	G	A	0.247	0.242	0.448	1.030	0.039	0.954	1.113	0.770	Imputed
rs75591172	2	151,852,385	A	G	0.101	0.096	0.332	1.056	0.056	0.946	1.179	0.877	Imputed
rs13026509	2	151,853,097	G	C	0.470	0.448	6.51E-03	1.097	0.034	1.026	1.172	0.282	Imputed
rs2341844	2	151,854,711	A	T	0.078	0.079	0.793	0.984	0.063	0.869	1.113	0.418	Imputed
rs114097755	2	151,854,956	G	A	0.025	0.026	0.686	0.957	0.108	0.775	1.183	0.827	Imputed
rs16829172	2	151,855,587	C	G	0.193	0.194	0.954	1.002	0.043	0.922	1.090	0.900	Imputed
chr2:151856162:I	2	151,856,162	GA	G	0.101	0.096	0.335	1.056	0.056	0.946	1.179	0.874	Imputed
rs1485101	2	151,856,579	T	C	0.026	0.025	0.597	1.058	0.107	0.858	1.306	0.347	Imputed
rs114931896	2	151,856,929	T	C	0.022	0.020	0.508	1.079	0.116	0.860	1.354	0.523	Imputed
rs1385418	2	151,857,138	T	A	0.101	0.096	0.317	1.058	0.056	0.948	1.181	0.890	Imputed
rs1385419	2	151,857,442	A	G	0.426	0.417	0.256	1.040	0.034	0.972	1.112	0.955	Imputed
rs1385421	2	151,857,541	G	A	0.180	0.176	0.520	1.029	0.044	0.944	1.122	0.615	Imputed
rs6709096	2	151,857,799	T	A	0.077	0.079	0.692	0.975	0.063	0.861	1.104	0.541	Imputed
chr2:151858101:D	2	151,858,101	T	TTC	0.290	0.317	3.60E-04	0.876	0.037	0.814	0.942	0.306	Imputed
rs77136799	2	151,858,431	T	A	0.026	0.022	0.208	1.146	0.108	0.927	1.418	0.491	Imputed

rs56748051	2	151,858,862	T	C	0.102	0.097	0.340	1.055	0.056	0.945	1.178	0.894	Imputed
rs6433108	2	151,858,868	G	A	0.180	0.176	0.521	1.029	0.044	0.944	1.121	0.642	Imputed
rs13390448	2	151,859,530	C	T	0.290	0.271	8.39E-03	1.104	0.037	1.026	1.188	0.104	Imputed
rs10175171	2	151,860,141	T	G	0.078	0.079	0.775	0.982	0.063	0.868	1.112	0.425	Imputed
rs59864395	2	151,860,805	A	T	0.102	0.096	0.294	1.061	0.056	0.950	1.184	0.910	Imputed
rs1485102	2	151,860,903	T	C	0.078	0.079	0.777	0.982	0.063	0.868	1.112	0.406	Imputed
chr2:151861572:I	2	151,861,572	A	AC	0.086	0.088	0.689	0.976	0.060	0.867	1.099	0.349	Imputed
rs76441237	2	151,861,716	T	A	0.102	0.096	0.294	1.061	0.056	0.950	1.184	0.910	Imputed
rs1385422	2	151,861,760	G	A	0.078	0.079	0.788	0.983	0.063	0.869	1.112	0.420	Imputed
rs138954829	2	151,862,004	A	G	0.102	0.096	0.294	1.061	0.056	0.950	1.184	0.910	Imputed
rs4664869	2	151,862,034	T	C	0.242	0.267	6.32E-04	0.874	0.039	0.809	0.944	0.517	Imputed
rs12465957	2	151,862,035	A	G	0.017	0.013	0.070	1.277	0.135	0.980	1.665	0.538	Imputed
rs4664871	2	151,862,070	G	A	0.393	0.380	0.142	1.053	0.035	0.983	1.127	0.624	Imputed
rs10211364	2	151,862,198	C	T	0.290	0.271	8.27E-03	1.104	0.037	1.026	1.188	0.091	Genotyped
rs62167165	2	151,863,059	G	A	0.051	0.046	0.110	1.132	0.077	0.973	1.317	0.336	Imputed
rs2220180	2	151,863,244	G	A	0.102	0.096	0.294	1.061	0.056	0.950	1.184	0.910	Imputed
rs78733166	2	151,863,430	C	T	0.103	0.097	0.279	1.062	0.056	0.952	1.186	0.913	Imputed
rs1485103	2	151,863,595	C	T	0.180	0.176	0.541	1.027	0.044	0.942	1.120	0.628	Imputed
rs62167166	2	151,863,658	C	T	0.053	0.048	0.150	1.116	0.076	0.961	1.295	0.325	Imputed
rs936767	2	151,864,071	A	C	0.176	0.173	0.582	1.025	0.044	0.939	1.118	0.570	Imputed
chr2:151864491:D	2	151,864,491	T	TA	0.283	0.311	1.78E-04	0.869	0.037	0.808	0.935	0.147	Imputed
rs79769149	2	151,864,913	A	G	0.153	0.144	0.198	1.063	0.047	0.969	1.166	0.055	Imputed
rs10206535	2	151,865,022	C	A	0.180	0.176	0.535	1.028	0.044	0.943	1.121	0.624	Genotyped
rs74342069	2	151,865,415	C	T	0.102	0.096	0.294	1.061	0.056	0.950	1.184	0.910	Imputed
rs10206941	2	151,865,465	C	T	0.180	0.176	0.545	1.027	0.044	0.942	1.120	0.630	Imputed
rs6705136	2	151,865,709	C	T	0.180	0.176	0.533	1.028	0.044	0.943	1.121	0.639	Imputed
chr2:151865717:I	2	151,865,717	G	GT	0.310	0.315	0.527	0.977	0.037	0.909	1.050	0.730	Imputed
chr2:151865754:I	2	151,865,754	C	CTGTT	0.091	0.096	0.379	0.950	0.059	0.847	1.065	0.512	Imputed
rs79910557	2	151,865,882	G	A	0.102	0.096	0.303	1.060	0.056	0.949	1.183	0.878	Imputed
rs4664873	2	151,866,453	G	A	0.180	0.176	0.533	1.028	0.044	0.943	1.121	0.639	Imputed
rs185346572	2	151,866,687	A	G	0.012	0.015	0.277	0.849	0.151	0.631	1.142	0.799	Imputed
rs12151382	2	151,866,725	T	C	0.283	0.311	2.36E-04	0.872	0.037	0.810	0.938	0.146	Imputed
rs936765	2	151,867,138	T	G	0.426	0.417	0.287	1.037	0.034	0.970	1.109	0.935	Imputed
rs62167167	2	151,867,387	C	T	0.053	0.048	0.148	1.116	0.076	0.962	1.296	0.327	Imputed
rs62167168	2	151,867,554	G	A	0.051	0.046	0.104	1.134	0.077	0.975	1.320	0.321	Imputed
rs111795027	2	151,867,678	A	T	0.011	0.011	0.718	1.059	0.159	0.775	1.446	0.167	Imputed
rs57201384	2	151,867,743	C	A	0.282	0.310	1.64E-04	0.869	0.037	0.807	0.935	0.127	Imputed
rs61433863	2	151,867,764	C	T	0.446	0.439	0.352	1.032	0.034	0.966	1.104	0.497	Imputed
rs7581655	2	151,868,167	T	G	0.078	0.079	0.772	0.982	0.063	0.868	1.111	0.408	Imputed
rs12470183	2	151,868,658	G	A	0.101	0.097	0.445	1.044	0.056	0.935	1.166	0.838	Imputed

rs12470267	2	151,868,894	C	A	0.101	0.097	0.472	1.041	0.056	0.933	1.163	0.819	Imputed
rs1485095	2	151,868,925	C	T	0.179	0.176	0.719	1.016	0.044	0.932	1.108	0.674	Imputed
rs1485094	2	151,868,975	G	T	0.078	0.079	0.746	0.980	0.063	0.866	1.109	0.400	Imputed
rs58129209	2	151,869,140	C	G	0.101	0.097	0.448	1.044	0.056	0.935	1.165	0.835	Imputed
rs56856850	2	151,869,171	A	G	0.078	0.079	0.746	0.980	0.063	0.866	1.109	0.400	Imputed
rs10187633	2	151,869,226	T	C	0.078	0.079	0.746	0.980	0.063	0.866	1.109	0.400	Imputed
rs10172940	2	151,869,231	C	G	0.078	0.079	0.746	0.980	0.063	0.866	1.109	0.400	Imputed
rs11684685	2	151,869,511	C	T	0.246	0.242	0.506	1.027	0.039	0.950	1.109	0.756	Imputed
rs10187955	2	151,869,515	T	A	0.078	0.080	0.726	0.978	0.063	0.865	1.107	0.408	Imputed
chr2:151869641:I	2	151,869,641	A	AT	0.079	0.081	0.632	0.971	0.063	0.858	1.097	0.443	Imputed
rs7585828	2	151,869,754	A	G	0.078	0.079	0.746	0.980	0.063	0.866	1.109	0.400	Imputed
rs7597842	2	151,869,808	G	C	0.078	0.079	0.746	0.980	0.063	0.866	1.109	0.400	Imputed
rs7572164	2	151,869,823	C	T	0.078	0.079	0.746	0.980	0.063	0.866	1.109	0.400	Imputed
rs7572404	2	151,870,040	C	T	0.078	0.079	0.746	0.980	0.063	0.866	1.109	0.400	Imputed
rs7598311	2	151,870,187	A	G	0.193	0.194	0.949	0.997	0.043	0.917	1.085	0.765	Genotyped
rs17270016	2	151,870,214	G	A	0.192	0.193	0.957	0.998	0.043	0.917	1.085	0.937	Imputed
rs1485093	2	151,870,612	G	A	0.078	0.079	0.755	0.981	0.063	0.866	1.110	0.414	Imputed
rs6433111	2	151,870,985	C	T	0.425	0.418	0.372	1.031	0.034	0.964	1.103	0.938	Genotyped
rs4664874	2	151,871,409	C	T	0.078	0.079	0.751	0.980	0.063	0.866	1.109	0.398	Imputed
rs16829215	2	151,871,445	T	C	0.053	0.048	0.158	1.113	0.076	0.959	1.292	0.339	Imputed
rs35680581	2	151,871,616	T	A	0.185	0.184	0.905	1.005	0.044	0.923	1.095	0.680	Imputed
rs62167171	2	151,871,619	G	A	0.185	0.185	0.962	1.002	0.044	0.920	1.091	0.685	Imputed
rs62167172	2	151,871,620	C	A	0.185	0.185	0.954	1.003	0.044	0.920	1.092	0.715	Imputed
rs34175226	2	151,871,624	C	T	0.185	0.185	0.946	1.003	0.044	0.921	1.092	0.711	Imputed
rs4664875	2	151,871,888	T	C	0.232	0.224	0.274	1.045	0.040	0.966	1.130	0.895	Genotyped
rs4664876	2	151,871,926	C	T	0.179	0.177	0.721	1.016	0.044	0.932	1.108	0.658	Imputed
rs4664877	2	151,871,929	T	A	0.173	0.172	0.834	1.009	0.045	0.925	1.102	0.947	Imputed
rs74843597	2	151,871,937	T	C	0.101	0.097	0.507	1.038	0.056	0.930	1.159	0.819	Imputed
rs4664878	2	151,871,940	G	A	0.179	0.177	0.721	1.016	0.044	0.932	1.108	0.658	Imputed
rs12987549	2	151,871,998	C	T	0.180	0.178	0.820	1.010	0.044	0.927	1.101	0.594	Imputed
rs13013454	2	151,872,059	A	G	0.180	0.178	0.820	1.010	0.044	0.927	1.101	0.594	Imputed
rs12987585	2	151,872,066	C	T	0.180	0.178	0.820	1.010	0.044	0.927	1.101	0.594	Imputed
rs12987768	2	151,872,127	C	T	0.472	0.450	8.05E-03	1.094	0.034	1.024	1.169	0.367	Imputed
rs12986468	2	151,872,205	G	C	0.179	0.178	0.843	1.009	0.044	0.925	1.100	0.605	Imputed
rs7560466	2	151,872,407	C	G	0.182	0.181	0.901	1.005	0.044	0.923	1.096	0.630	Imputed
rs7577063	2	151,872,432	T	C	0.180	0.178	0.827	1.010	0.044	0.926	1.101	0.597	Imputed
rs1485092	2	151,872,584	G	A	0.181	0.180	0.878	1.007	0.044	0.924	1.097	0.675	Imputed
rs16829234	2	151,872,685	G	A	0.053	0.048	0.183	1.107	0.076	0.954	1.285	0.318	Imputed
rs12464767	2	151,873,098	T	C	0.102	0.099	0.534	1.035	0.056	0.928	1.156	0.965	Genotyped
rs11895825	2	151,873,259	G	A	0.179	0.178	0.862	1.008	0.044	0.924	1.099	0.518	Imputed

rs11890981	2	151,873,378	A	G	0.179	0.178	0.876	1.007	0.044	0.924	1.098	0.539	Imputed
rs10930374	2	151,873,461	G	A	0.179	0.178	0.862	1.008	0.044	0.924	1.099	0.533	Imputed
rs10497072	2	151,873,535	A	G	0.077	0.079	0.665	0.973	0.063	0.859	1.102	0.337	Imputed
rs10497073	2	151,873,561	A	G	0.179	0.178	0.862	1.008	0.044	0.924	1.099	0.533	Imputed
rs78782607	2	151,873,651	G	A	0.102	0.099	0.534	1.035	0.056	0.928	1.156	0.942	Imputed
rs9287920	2	151,873,685	T	C	0.179	0.178	0.862	1.008	0.044	0.924	1.099	0.533	Imputed
rs10186209	2	151,873,828	T	C	0.371	0.369	0.722	1.013	0.035	0.945	1.084	0.592	Genotyped
rs10183711	2	151,873,884	A	T	0.179	0.178	0.862	1.008	0.044	0.924	1.099	0.533	Imputed
rs2341841	2	151,874,159	G	C	0.179	0.178	0.862	1.008	0.044	0.924	1.099	0.533	Imputed
rs2341842	2	151,874,232	A	C	0.283	0.308	4.53E-04	0.877	0.037	0.815	0.944	0.185	Imputed
rs2341843	2	151,874,265	A	G	0.179	0.178	0.862	1.008	0.044	0.924	1.099	0.533	Imputed
rs77664875	2	151,874,288	T	C	0.016	0.016	0.949	0.992	0.135	0.761	1.291	0.378	Imputed
rs6735085	2	151,874,500	T	C	0.179	0.178	0.862	1.008	0.044	0.924	1.099	0.533	Imputed
rs6717342	2	151,874,544	C	G	0.179	0.178	0.866	1.007	0.044	0.924	1.098	0.535	Imputed
rs4664879	2	151,874,718	T	C	0.179	0.178	0.862	1.008	0.044	0.924	1.099	0.533	Imputed
rs4664880	2	151,874,723	G	C	0.179	0.178	0.862	1.008	0.044	0.924	1.099	0.533	Imputed
rs4664881	2	151,874,855	C	T	0.179	0.178	0.862	1.008	0.044	0.924	1.099	0.533	Imputed

Table S2c RNF144B-ID4 region on chromosome 6

SNP	CHR	BP	Allele 1	Allele 2	F_A	F_U	P cmh test	OR cmh	SE	L95	U95	P het	Method
rs10484719	6	19,553,937	G	T	0.165	0.160	0.374	1.041	0.046	0.952	1.139	0.880	Imputed
rs9350162	6	19,554,284	T	C	0.468	0.456	0.185	1.046	0.034	0.979	1.118	0.957	Imputed
rs77903790	6	19,554,908	A	G	0.012	0.012	0.793	0.960	0.157	0.706	1.305	0.712	Imputed
rs2743594	6	19,555,819	T	C	0.406	0.387	0.054	1.069	0.034	0.999	1.143	0.393	Imputed
rs13197725	6	19,555,838	A	G	0.173	0.162	0.134	1.069	0.045	0.979	1.168	0.321	Imputed
rs2743595	6	19,556,168	A	G	0.408	0.391	0.073	1.064	0.034	0.994	1.138	0.636	Genotyped
rs9465436	6	19,556,807	A	T	0.172	0.161	0.116	1.073	0.045	0.983	1.172	0.317	Imputed
rs140760275	6	19,557,122	T	C	0.037	0.039	0.479	0.939	0.090	0.787	1.119	0.890	Imputed
rs116232405	6	19,558,294	A	G	0.011	0.013	0.375	0.870	0.158	0.638	1.185	0.412	Imputed
rs9465437	6	19,558,313	C	T	0.073	0.067	0.164	1.096	0.066	0.964	1.246	0.403	Imputed
chr6:19558328:D	6	19,558,328	C	CA	0.397	0.380	0.065	1.066	0.035	0.996	1.142	0.755	Imputed
rs9465438	6	19,558,904	A	G	0.051	0.048	0.433	1.062	0.077	0.913	1.236	0.178	Genotyped
rs62402573	6	19,559,129	C	T	0.137	0.135	0.594	1.027	0.050	0.932	1.131	0.141	Imputed
rs9460381	6	19,559,488	C	T	0.062	0.058	0.290	1.078	0.071	0.939	1.238	0.089	Imputed
rs2064105	6	19,559,996	T	C	0.012	0.012	0.835	0.968	0.156	0.713	1.315	0.030	Imputed
rs10484720	6	19,560,024	C	T	0.258	0.270	0.100	0.939	0.039	0.870	1.012	0.569	Genotyped
rs2743596	6	19,560,177	A	C	0.200	0.195	0.514	1.028	0.043	0.946	1.118	0.708	Imputed
rs11754765	6	19,560,183	C	G	0.021	0.021	0.746	0.962	0.119	0.762	1.215	0.371	Imputed

rs7751362	6	19,560,246	T	G	0.109	0.119	0.048	0.899	0.054	0.808	0.999	0.034	Imputed
rs760669	6	19,561,987	T	C	0.270	0.279	0.313	0.962	0.038	0.893	1.037	0.842	Genotyped
rs116146936	6	19,562,393	G	A	0.015	0.013	0.229	1.183	0.140	0.900	1.557	0.966	Imputed
rs2743597	6	19,563,321	C	T	0.280	0.288	0.418	0.970	0.038	0.901	1.044	0.508	Genotyped
rs9465443	6	19,563,666	C	T	0.037	0.031	0.047	1.198	0.091	1.002	1.432	0.634	Imputed
rs9460382	6	19,564,461	G	A	0.100	0.089	0.016	1.147	0.057	1.026	1.282	0.462	Genotyped
rs2743598	6	19,564,477	C	A	0.312	0.319	0.464	0.974	0.037	0.906	1.046	0.840	Imputed
rs2205863	6	19,564,975	A	G	0.285	0.292	0.449	0.972	0.037	0.903	1.046	0.857	Imputed
rs1883233	6	19,566,284	C	T	0.222	0.219	0.513	1.027	0.041	0.948	1.112	0.438	Genotyped
rs7745130	6	19,567,942	A	G	0.501	0.499	0.878	1.005	0.034	0.941	1.074	0.999	Genotyped
rs115337948	6	19,568,902	C	T	0.015	0.014	0.434	1.116	0.140	0.848	1.468	0.657	Imputed
rs9350163	6	19,568,937	G	A	0.318	0.320	0.784	0.990	0.036	0.922	1.063	0.574	Imputed
rs2743602	6	19,569,044	C	T	0.082	0.077	0.228	1.078	0.062	0.954	1.217	0.778	Genotyped
rs2743604	6	19,569,095	G	A	0.083	0.078	0.270	1.070	0.062	0.949	1.208	0.700	Imputed
rs113719642	6	19,569,186	A	G	0.051	0.049	0.594	1.042	0.077	0.896	1.212	0.469	Imputed
rs9368091	6	19,569,753	C	T	0.499	0.498	0.955	1.002	0.034	0.938	1.071	0.942	Imputed
rs9368092	6	19,570,107	C	G	0.499	0.498	0.935	1.003	0.034	0.938	1.072	0.931	Imputed
rs2179176	6	19,570,634	A	G	0.415	0.419	0.605	0.982	0.034	0.919	1.051	0.754	Genotyped
rs80231250	6	19,571,400	C	G	0.082	0.076	0.210	1.081	0.062	0.957	1.221	0.695	Imputed
rs34908874	6	19,571,542	G	A	0.074	0.067	0.141	1.101	0.065	0.969	1.252	0.036	Imputed
rs2743605	6	19,571,592	A	G	0.084	0.078	0.221	1.078	0.061	0.956	1.216	0.808	Imputed
rs2817280	6	19,572,439	T	C	0.082	0.077	0.239	1.076	0.062	0.953	1.214	0.767	Imputed
rs2817279	6	19,573,444	G	C	0.084	0.078	0.219	1.078	0.061	0.956	1.216	0.811	Imputed
rs2817276	6	19,575,082	C	T	0.082	0.077	0.239	1.076	0.062	0.953	1.214	0.767	Imputed
rs6456252	6	19,575,378	G	C	0.495	0.499	0.681	0.986	0.034	0.923	1.054	0.845	Imputed
rs79342135	6	19,575,485	A	G	0.078	0.083	0.352	0.943	0.063	0.834	1.067	0.120	Imputed
rs7755592	6	19,575,578	G	C	0.062	0.057	0.285	1.079	0.071	0.939	1.240	0.097	Imputed
rs2493218	6	19,575,837	A	T	0.084	0.078	0.197	1.082	0.061	0.960	1.221	0.828	Imputed
rs6456253	6	19,575,853	C	T	0.498	0.500	0.821	0.992	0.034	0.929	1.060	0.816	Imputed
rs148440460	6	19,576,634	A	G	0.012	0.014	0.233	0.834	0.153	0.619	1.125	0.550	Imputed
rs7747426	6	19,576,950	T	C	0.421	0.425	0.590	0.982	0.034	0.918	1.050	0.890	Imputed
rs12179765	6	19,576,987	G	A	0.083	0.078	0.258	1.072	0.062	0.950	1.210	0.711	Imputed
rs77615679	6	19,578,160	C	T	0.081	0.076	0.229	1.078	0.062	0.954	1.218	0.578	Imputed
rs113787203	6	19,578,406	C	T	0.089	0.088	0.684	1.025	0.059	0.912	1.151	0.444	Imputed
chr6:19578428:D	6	19,578,428	C	CAT	0.080	0.075	0.238	1.077	0.063	0.952	1.217	0.791	Imputed
rs34445009	6	19,578,933	A	T	0.115	0.110	0.301	1.057	0.053	0.952	1.172	0.491	Imputed
rs2817273	6	19,579,138	T	A	0.082	0.077	0.228	1.078	0.062	0.954	1.217	0.778	Imputed
rs28825503	6	19,579,843	C	T	0.082	0.076	0.168	1.089	0.062	0.965	1.230	0.720	Imputed
rs28841064	6	19,580,053	T	C	0.082	0.076	0.210	1.081	0.062	0.957	1.221	0.695	Imputed
rs114854862	6	19,580,116	A	C	0.016	0.016	0.830	1.029	0.133	0.792	1.336	0.186	Imputed

rs2817271	6	19,580,222	G	A	0.084	0.078	0.196	1.082	0.061	0.960	1.221	0.836	Imputed
chr6:19581291:D	6	19,581,291	T	TC	0.082	0.076	0.159	1.091	0.062	0.966	1.232	0.757	Imputed
rs2743607	6	19,582,396	C	T	0.082	0.077	0.215	1.080	0.062	0.956	1.219	0.819	Imputed
rs6909115	6	19,582,625	T	A	0.502	0.500	0.801	1.009	0.034	0.944	1.078	0.811	Imputed
rs116470514	6	19,582,815	A	T	0.031	0.026	0.088	1.183	0.099	0.975	1.436	0.653	Imputed
rs2817270	6	19,583,011	T	C	0.082	0.076	0.218	1.079	0.062	0.956	1.219	0.817	Imputed
rs2817269	6	19,583,447	T	C	0.084	0.078	0.180	1.086	0.061	0.963	1.224	0.883	Imputed
rs9460384	6	19,584,041	C	T	0.082	0.076	0.153	1.093	0.062	0.968	1.234	0.766	Imputed
rs13362587	6	19,584,784	T	C	0.082	0.076	0.193	1.084	0.062	0.960	1.224	0.740	Imputed
chr6:19585044:I	6	19,585,044	GA	G	0.024	0.029	0.075	0.822	0.110	0.663	1.020	0.864	Imputed
rs12332955	6	19,585,459	C	T	0.082	0.076	0.193	1.084	0.062	0.960	1.224	0.740	Imputed
rs112457841	6	19,586,282	G	T	0.082	0.076	0.193	1.084	0.062	0.960	1.224	0.740	Imputed
rs9350164	6	19,586,721	C	G	0.424	0.430	0.368	0.970	0.034	0.907	1.037	0.865	Imputed
rs114223366	6	19,586,951	T	C	0.064	0.067	0.448	0.949	0.069	0.829	1.086	0.737	Imputed
rs2817268	6	19,586,992	A	G	0.425	0.431	0.392	0.971	0.034	0.908	1.038	0.904	Imputed
rs72836014	6	19,587,285	A	G	0.103	0.095	0.094	1.098	0.056	0.984	1.226	0.368	Imputed
rs68074779	6	19,587,286	T	C	0.103	0.095	0.094	1.098	0.056	0.984	1.226	0.368	Imputed
rs2817265	6	19,588,359	A	G	0.084	0.078	0.175	1.086	0.061	0.964	1.225	0.889	Imputed
rs67145622	6	19,588,752	T	C	0.115	0.110	0.290	1.058	0.053	0.953	1.174	0.421	Imputed
rs2817264	6	19,589,291	T	C	0.084	0.078	0.180	1.086	0.061	0.963	1.224	0.883	Imputed
rs2743610	6	19,589,595	C	T	0.084	0.079	0.196	1.082	0.061	0.960	1.220	0.863	Genotyped
chr6:19589809:D	6	19,589,809	T	TAG	0.082	0.077	0.218	1.079	0.062	0.956	1.218	0.816	Imputed
rs2743611	6	19,590,455	C	T	0.417	0.421	0.526	0.979	0.034	0.915	1.047	0.797	Imputed
rs2473028	6	19,591,953	A	T	0.084	0.079	0.178	1.086	0.061	0.963	1.224	0.939	Imputed
chr6:19592432:D	6	19,592,432	A	AG	0.082	0.076	0.188	1.085	0.062	0.961	1.225	0.746	Imputed
chr6:19592509:I	6	19,592,509	CAT	C	0.010	0.011	0.816	0.961	0.169	0.690	1.339	0.121	Imputed
rs2743613	6	19,592,510	T	A	0.088	0.080	0.083	1.110	0.060	0.987	1.249	0.804	Imputed
rs9460385	6	19,592,564	G	T	0.088	0.080	0.092	1.107	0.060	0.984	1.245	0.769	Imputed
rs78409950	6	19,592,794	T	C	0.084	0.078	0.162	1.089	0.061	0.966	1.228	0.933	Imputed
rs2817263	6	19,593,634	C	G	0.416	0.420	0.536	0.979	0.034	0.915	1.047	0.647	Imputed
rs9465455	6	19,593,807	T	A	0.082	0.076	0.188	1.085	0.062	0.961	1.225	0.746	Imputed
chr6:19594107:D	6	19,594,107	T	GCTTTGTATTAGGCACTA	0.492	0.489	0.721	1.012	0.034	0.947	1.082	0.813	Imputed
rs72836019	6	19,595,760	A	G	0.034	0.036	0.578	0.950	0.093	0.792	1.139	0.666	Imputed
rs72836020	6	19,595,849	A	G	0.061	0.057	0.232	1.089	0.071	0.947	1.251	0.087	Imputed
rs9465459	6	19,596,431	C	A	0.048	0.043	0.128	1.129	0.080	0.966	1.319	0.804	Imputed
chr6:19596867:I	6	19,596,867	GA	G	0.069	0.072	0.486	0.955	0.067	0.838	1.088	0.647	Imputed
rs140483486	6	19,597,088	A	G	0.014	0.013	0.372	1.139	0.145	0.857	1.514	0.211	Imputed
rs2743615	6	19,597,131	T	C	0.084	0.079	0.173	1.087	0.061	0.964	1.225	0.891	Imputed
rs10484721	6	19,597,229	C	T	0.082	0.076	0.195	1.084	0.062	0.960	1.224	0.737	Imputed
rs2743616	6	19,597,331	T	A	0.082	0.077	0.210	1.081	0.062	0.957	1.220	0.825	Imputed

rs144649198	6	19,598,334	C	A	0.013	0.016	0.258	0.847	0.147	0.635	1.130	0.842	Imputed
rs74322648	6	19,598,450	A	G	0.028	0.029	0.693	0.960	0.103	0.785	1.175	0.683	Imputed
rs2473029	6	19,598,474	G	C	0.416	0.420	0.529	0.979	0.034	0.915	1.047	0.651	Imputed
rs146704671	6	19,598,789	A	C	0.032	0.027	0.065	1.196	0.097	0.989	1.448	0.657	Imputed
rs115495583	6	19,599,033	T	C	0.030	0.031	0.985	1.002	0.098	0.826	1.214	0.327	Imputed
rs78216582	6	19,599,473	T	C	0.082	0.076	0.188	1.085	0.062	0.961	1.225	0.746	Imputed
rs2817297	6	19,599,795	A	G	0.415	0.420	0.538	0.979	0.034	0.915	1.047	0.646	Genotyped
rs35770510	6	19,599,978	T	C	0.015	0.011	0.056	1.311	0.142	0.992	1.733	0.494	Imputed
rs2817296	6	19,600,214	G	T	0.082	0.077	0.210	1.081	0.062	0.957	1.220	0.825	Imputed
rs2743617	6	19,600,328	C	T	0.084	0.079	0.173	1.087	0.061	0.964	1.225	0.891	Imputed
rs2743618	6	19,600,521	A	C	0.406	0.413	0.382	0.970	0.034	0.907	1.038	0.669	Imputed
rs11758567	6	19,601,623	T	C	0.051	0.049	0.711	1.029	0.077	0.885	1.197	0.393	Imputed
rs9465461	6	19,602,406	G	T	0.082	0.076	0.188	1.085	0.062	0.961	1.225	0.746	Imputed
rs2743570	6	19,602,860	C	A	0.082	0.077	0.210	1.081	0.062	0.957	1.220	0.825	Imputed
rs2743571	6	19,602,950	G	C	0.084	0.079	0.171	1.087	0.061	0.965	1.226	0.921	Imputed
rs10806896	6	19,602,973	C	G	0.501	0.500	0.924	1.003	0.034	0.939	1.072	0.841	Imputed
rs6911692	6	19,604,199	C	A	0.059	0.055	0.199	1.097	0.072	0.953	1.264	0.030	Imputed
rs2817283	6	19,604,412	G	A	0.424	0.427	0.664	0.985	0.034	0.921	1.054	0.744	Genotyped
rs6912367	6	19,604,466	T	C	0.493	0.495	0.811	0.992	0.034	0.928	1.060	0.915	Imputed
rs1936902	6	19,604,724	G	A	0.420	0.424	0.543	0.979	0.034	0.916	1.047	0.868	Imputed
rs1936903	6	19,604,924	T	C	0.085	0.078	0.130	1.097	0.061	0.973	1.236	0.803	Genotyped
rs1936904	6	19,605,126	A	G	0.088	0.080	0.086	1.108	0.060	0.985	1.247	0.942	Imputed
rs11758252	6	19,606,057	T	C	0.090	0.082	0.069	1.114	0.059	0.991	1.252	0.701	Imputed
rs28569373	6	19,606,930	G	A	0.103	0.113	0.066	0.903	0.056	0.809	1.007	0.099	Imputed
rs28854627	6	19,607,013	G	C	0.069	0.066	0.577	1.038	0.067	0.910	1.184	0.128	Imputed
rs144353449	6	19,607,025	C	G	0.333	0.332	0.954	1.002	0.036	0.933	1.076	0.826	Imputed
rs113210285	6	19,607,028	T	A	0.047	0.044	0.325	1.082	0.080	0.924	1.267	0.361	Imputed
rs141280669	6	19,607,040	T	A	0.299	0.299	0.954	1.002	0.037	0.932	1.078	0.438	Imputed
chr6:19607514:I	6	19,607,514	ATAACT	A	0.469	0.464	0.527	1.022	0.034	0.956	1.092	0.399	Imputed
rs10946329	6	19,607,690	T	C	0.493	0.494	0.946	0.998	0.034	0.934	1.066	0.683	Imputed
rs10946330	6	19,607,696	T	C	0.494	0.494	0.989	1.000	0.034	0.935	1.068	0.613	Imputed
chr6:19608532:D	6	19,608,532	G	GGT	0.015	0.016	0.468	0.903	0.140	0.686	1.189	0.615	Imputed
rs11965154	6	19,608,592	T	A	0.495	0.495	0.995	1.000	0.034	0.936	1.069	0.671	Imputed
rs2817292	6	19,608,760	G	C	0.412	0.419	0.330	0.967	0.034	0.904	1.034	0.712	Imputed
chr6:19608843:I	6	19,608,843	GC	G	0.494	0.494	0.960	1.002	0.034	0.937	1.070	0.744	Imputed
rs2743576	6	19,609,364	A	G	0.024	0.023	0.865	1.019	0.111	0.820	1.267	0.976	Genotyped
rs9766473	6	19,609,834	C	T	0.496	0.497	0.991	1.000	0.034	0.936	1.068	0.612	Imputed
rs9465463	6	19,610,117	A	G	0.091	0.083	0.076	1.111	0.059	0.989	1.247	0.705	Imputed
rs6905926	6	19,610,320	A	G	0.496	0.497	0.997	1.000	0.034	0.936	1.068	0.615	Imputed
rs114033357	6	19,610,388	T	C	0.007	0.011	0.038	0.674	0.192	0.462	0.982	0.392	Imputed

rs13214665	6	19,610,766	G	T	0.091	0.083	0.076	1.111	0.059	0.989	1.247	0.705	Imputed
rs6910563	6	19,610,835	C	A	0.497	0.498	0.967	0.999	0.034	0.935	1.067	0.606	Imputed
rs6933602	6	19,610,858	T	C	0.412	0.419	0.347	0.968	0.034	0.905	1.036	0.712	Genotyped
rs6934188	6	19,611,159	C	T	0.497	0.498	0.973	0.999	0.034	0.935	1.067	0.608	Imputed
rs76962271	6	19,611,195	A	T	0.048	0.046	0.825	1.018	0.080	0.871	1.189	0.363	Imputed
rs9350166	6	19,611,231	T	C	0.497	0.497	0.999	1.000	0.034	0.936	1.069	0.585	Imputed
rs9350167	6	19,611,284	T	G	0.495	0.495	0.995	1.000	0.034	0.936	1.069	0.671	Imputed
rs9295444	6	19,611,375	G	A	0.497	0.497	0.999	1.000	0.034	0.936	1.069	0.585	Imputed
rs9368098	6	19,611,426	C	T	0.495	0.495	0.995	1.000	0.034	0.936	1.069	0.671	Imputed
rs9358248	6	19,611,739	A	G	0.497	0.497	0.999	1.000	0.034	0.936	1.069	0.585	Imputed
rs9358249	6	19,611,913	A	C	0.497	0.497	0.994	1.000	0.034	0.936	1.069	0.588	Imputed
rs9350168	6	19,612,034	G	A	0.497	0.497	0.988	1.001	0.034	0.936	1.069	0.578	Imputed
chr6:19612966:I	6	19,612,966	TC	T	0.491	0.491	0.919	1.003	0.034	0.939	1.072	0.695	Imputed
rs1936905	6	19,613,078	C	T	0.497	0.497	0.973	1.001	0.034	0.937	1.070	0.549	Genotyped
rs1936906	6	19,613,088	A	G	0.405	0.412	0.310	0.966	0.034	0.903	1.033	0.686	Imputed
rs1936907	6	19,613,120	G	A	0.497	0.498	0.997	1.000	0.034	0.936	1.069	0.555	Imputed
rs9348387	6	19,613,709	T	C	0.497	0.497	0.991	1.000	0.034	0.936	1.069	0.577	Imputed
rs9366302	6	19,613,858	A	C	0.494	0.495	0.994	1.000	0.034	0.936	1.069	0.634	Imputed
rs142142795	6	19,614,359	A	T	0.344	0.343	0.987	0.999	0.036	0.932	1.072	0.625	Imputed
rs183711860	6	19,614,384	A	G	0.019	0.022	0.230	0.862	0.124	0.677	1.099	0.859	Imputed
rs72836028	6	19,614,926	G	A	0.481	0.483	0.905	0.996	0.034	0.932	1.064	0.562	Imputed
rs67321156	6	19,614,940	C	T	0.481	0.483	0.905	0.996	0.034	0.932	1.064	0.562	Imputed
rs9465464	6	19,615,363	C	G	0.090	0.083	0.086	1.107	0.059	0.986	1.244	0.750	Imputed
rs1936908	6	19,616,036	G	A	0.478	0.480	0.895	0.996	0.034	0.932	1.064	0.622	Imputed
rs1936909	6	19,616,157	G	A	0.481	0.483	0.890	0.995	0.034	0.931	1.064	0.543	Imputed
chr6:19616174:I	6	19,616,174	GA	G	0.481	0.483	0.890	0.995	0.034	0.931	1.064	0.543	Imputed
rs55779808	6	19,616,733	A	G	0.481	0.483	0.892	0.995	0.034	0.932	1.064	0.544	Imputed
rs7753163	6	19,617,303	A	G	0.481	0.483	0.884	0.995	0.034	0.931	1.063	0.552	Imputed
rs7773374	6	19,617,378	G	T	0.481	0.483	0.892	0.995	0.034	0.932	1.064	0.544	Imputed
rs7753244	6	19,617,525	T	C	0.480	0.482	0.891	0.995	0.034	0.932	1.064	0.532	Imputed
chr6:19617536:I	6	19,617,536	TA	T	0.480	0.481	0.953	0.998	0.034	0.934	1.066	0.510	Imputed
rs1120461	6	19,617,721	T	C	0.478	0.480	0.901	0.996	0.034	0.932	1.064	0.612	Imputed
rs1120462	6	19,617,841	G	A	0.481	0.483	0.912	0.996	0.034	0.932	1.065	0.628	Genotyped
rs1120463	6	19,617,885	G	A	0.481	0.483	0.889	0.995	0.034	0.931	1.064	0.543	Imputed
rs1120464	6	19,617,968	A	C	0.419	0.424	0.418	0.973	0.034	0.909	1.040	0.495	Imputed
rs143017509	6	19,618,112	A	C	0.017	0.012	0.024	1.354	0.135	1.039	1.765	0.707	Imputed
rs1954541	6	19,618,388	C	G	0.480	0.482	0.873	0.995	0.034	0.931	1.063	0.554	Imputed
chr6:19618673:D	6	19,618,673	T	TA	0.468	0.471	0.789	0.991	0.034	0.927	1.059	0.313	Imputed
rs56188615	6	19,618,940	C	A	0.134	0.140	0.390	0.958	0.050	0.870	1.056	0.446	Imputed
rs55679921	6	19,619,036	A	G	0.477	0.479	0.916	0.996	0.034	0.932	1.065	0.508	Imputed

rs11969049	6	19,619,060	G	A	0.478	0.480	0.882	0.995	0.034	0.931	1.063	0.520	Imputed
rs11964162	6	19,619,143	A	G	0.478	0.479	0.867	0.994	0.034	0.931	1.063	0.654	Imputed
rs2876559	6	19,619,414	G	A	0.429	0.435	0.428	0.973	0.034	0.910	1.041	0.708	Imputed
rs9368101	6	19,619,700	A	G	0.480	0.482	0.873	0.995	0.034	0.931	1.063	0.573	Imputed
rs71535508	6	19,619,705	A	G	0.022	0.022	0.954	1.007	0.116	0.803	1.263	0.282	Imputed
rs9348388	6	19,619,773	C	G	0.479	0.481	0.891	0.995	0.034	0.931	1.064	0.613	Imputed
rs112811949	6	19,619,829	C	T	0.091	0.083	0.076	1.111	0.059	0.989	1.247	0.705	Imputed
rs9356700	6	19,619,924	C	T	0.494	0.495	0.934	0.997	0.034	0.933	1.066	0.644	Imputed
chr6:19619933:D	6	19,619,933	A	AT	0.494	0.495	0.934	0.997	0.034	0.933	1.066	0.644	Imputed
rs9356701	6	19,619,957	C	G	0.495	0.496	0.928	0.997	0.034	0.933	1.065	0.621	Imputed
rs35602465	6	19,620,042	G	A	0.091	0.083	0.076	1.111	0.059	0.989	1.247	0.705	Imputed
rs2328484	6	19,620,361	C	G	0.496	0.497	0.938	0.997	0.034	0.933	1.066	0.544	Imputed
rs4610552	6	19,620,452	G	A	0.496	0.497	0.968	0.999	0.034	0.935	1.067	0.546	Imputed
rs2876558	6	19,620,480	C	T	0.496	0.497	0.968	0.999	0.034	0.935	1.067	0.546	Imputed
rs61578935	6	19,620,779	A	G	0.496	0.497	0.971	0.999	0.034	0.935	1.067	0.547	Imputed
rs34740846	6	19,620,870	T	C	0.468	0.471	0.840	0.993	0.034	0.929	1.061	0.167	Imputed
rs7454652	6	19,620,886	T	C	0.487	0.490	0.744	0.989	0.034	0.926	1.057	0.468	Imputed
rs9717730	6	19,620,989	A	G	0.164	0.152	0.058	1.091	0.046	0.997	1.195	0.582	Imputed
rs6456254	6	19,621,098	C	G	0.449	0.455	0.432	0.974	0.034	0.911	1.041	0.612	Imputed
rs7774717	6	19,621,200	A	G	0.479	0.481	0.872	0.995	0.034	0.931	1.063	0.514	Imputed
rs7774619	6	19,621,304	G	C	0.493	0.493	0.971	0.999	0.034	0.935	1.067	0.624	Imputed
rs7774828	6	19,621,521	C	A	0.474	0.476	0.973	0.999	0.034	0.935	1.067	0.258	Imputed
chr6:19621607:D	6	19,621,607	C	CA	0.091	0.083	0.076	1.111	0.059	0.989	1.247	0.705	Imputed
rs9358251	6	19,621,732	C	T	0.496	0.496	0.980	0.999	0.034	0.935	1.068	0.570	Imputed
rs9358252	6	19,621,821	C	T	0.486	0.486	0.857	1.006	0.034	0.942	1.075	0.273	Imputed
rs9465465	6	19,621,824	C	G	0.091	0.083	0.076	1.111	0.059	0.989	1.247	0.705	Imputed
rs9358253	6	19,621,862	C	A	0.434	0.430	0.472	1.025	0.034	0.958	1.096	0.118	Imputed
rs9368102	6	19,622,087	T	A	0.469	0.468	0.889	1.005	0.034	0.940	1.074	0.259	Imputed
rs138780361	6	19,622,708	A	G	0.487	0.485	0.776	1.010	0.034	0.945	1.079	0.610	Imputed
rs141115938	6	19,622,819	G	T	0.454	0.456	0.919	0.997	0.034	0.932	1.065	0.474	Imputed
rs143325247	6	19,622,893	C	T	0.490	0.491	0.976	1.001	0.034	0.937	1.070	0.549	Imputed
rs138151184	6	19,623,012	G	C	0.469	0.470	0.992	1.000	0.034	0.935	1.068	0.268	Imputed
rs182868337	6	19,623,234	C	A	0.016	0.016	0.879	0.980	0.134	0.753	1.274	0.674	Imputed
rs188192201	6	19,623,267	G	A	0.045	0.043	0.415	1.069	0.082	0.911	1.254	0.366	Imputed
rs10434838	6	19,623,494	C	A	0.466	0.473	0.277	0.964	0.034	0.902	1.030	0.382	Imputed
rs113808940	6	19,624,278	C	T	0.496	0.497	0.919	0.997	0.034	0.933	1.065	0.513	Imputed
rs142777486	6	19,624,310	G	A	0.094	0.085	0.051	1.121	0.058	1.000	1.257	0.607	Imputed
rs6456255	6	19,625,142	C	T	0.414	0.421	0.377	0.970	0.034	0.907	1.038	0.671	Imputed
rs10434839	6	19,625,739	A	G	0.483	0.483	0.987	1.000	0.034	0.935	1.068	0.283	Imputed
rs10434828	6	19,625,748	G	A	0.486	0.487	0.967	1.001	0.034	0.937	1.070	0.320	Imputed

rs6938345	6	19,626,242	C	T	0.414	0.421	0.353	0.969	0.034	0.906	1.036	0.715	Imputed
rs138906148	6	19,626,443	C	A	0.021	0.020	0.743	1.040	0.118	0.825	1.311	0.899	Imputed
rs6900462	6	19,626,762	G	A	0.491	0.492	0.946	0.998	0.034	0.934	1.066	0.460	Imputed
rs11966061	6	19,627,064	A	C	0.327	0.324	0.575	1.020	0.036	0.951	1.095	0.936	Imputed
rs10946332	6	19,627,155	A	G	0.329	0.325	0.516	1.024	0.036	0.954	1.099	0.839	Imputed
rs11961210	6	19,627,445	A	G	0.418	0.425	0.337	0.968	0.034	0.905	1.035	0.626	Imputed
rs12527655	6	19,627,807	A	G	0.326	0.322	0.591	1.020	0.036	0.950	1.094	0.851	Imputed
rs77999185	6	19,628,019	G	A	0.162	0.166	0.468	0.967	0.046	0.884	1.058	0.537	Imputed
rs2328483	6	19,628,340	G	A	0.329	0.326	0.579	1.020	0.036	0.951	1.095	0.766	Imputed
rs6930954	6	19,628,633	G	T	0.326	0.323	0.619	1.018	0.036	0.949	1.093	0.811	Genotyped
chr6:19629067:D	6	19,629,067	T	TC	0.151	0.148	0.639	1.022	0.047	0.932	1.122	0.213	Imputed
rs4452635	6	19,629,346	C	T	0.329	0.326	0.604	1.019	0.036	0.949	1.093	0.819	Imputed
rs9366303	6	19,629,491	C	G	0.327	0.323	0.609	1.019	0.036	0.949	1.093	0.915	Imputed
rs9465467	6	19,629,554	G	T	0.099	0.090	0.042	1.123	0.057	1.004	1.255	0.649	Genotyped
rs2096373	6	19,629,844	G	A	0.481	0.483	0.910	0.996	0.034	0.932	1.065	0.497	Imputed
rs10946333	6	19,629,907	A	T	0.326	0.322	0.610	1.019	0.036	0.949	1.093	0.908	Imputed
rs9460387	6	19,629,921	G	A	0.099	0.090	0.044	1.121	0.057	1.003	1.254	0.582	Genotyped
rs10806904	6	19,630,197	C	T	0.325	0.322	0.625	1.018	0.036	0.948	1.092	0.953	Imputed
rs79234575	6	19,630,321	T	G	0.155	0.159	0.438	0.964	0.047	0.880	1.057	0.376	Imputed
chr6:19630471:D	6	19,630,471	A	AT	0.329	0.324	0.484	1.026	0.036	0.956	1.101	0.672	Imputed
rs10946334	6	19,630,500	T	C	0.481	0.483	0.906	0.996	0.034	0.932	1.064	0.495	Imputed
rs9358254	6	19,630,657	G	T	0.479	0.478	0.931	1.003	0.034	0.939	1.072	0.467	Imputed
rs9465468	6	19,630,854	A	G	0.099	0.090	0.053	1.117	0.057	0.999	1.249	0.609	Imputed
rs140641453	6	19,630,926	A	G	0.032	0.033	0.594	0.950	0.096	0.788	1.147	0.476	Imputed
rs75822785	6	19,631,032	A	C	0.155	0.159	0.443	0.965	0.047	0.880	1.057	0.378	Imputed
rs9358255	6	19,631,149	C	T	0.325	0.322	0.630	1.018	0.036	0.948	1.092	0.949	Imputed
rs9460388	6	19,631,328	G	A	0.099	0.090	0.053	1.117	0.057	0.999	1.249	0.609	Imputed
rs9460389	6	19,632,073	T	A	0.091	0.083	0.067	1.114	0.059	0.993	1.251	0.658	Imputed
rs138695951	6	19,632,102	A	G	0.021	0.020	0.743	1.040	0.118	0.825	1.311	0.899	Imputed
rs6900491	6	19,632,668	G	C	0.106	0.102	0.453	1.042	0.055	0.936	1.161	0.458	Imputed
chr6:19632693:I	6	19,632,693	TAG	T	0.488	0.489	0.972	0.999	0.034	0.935	1.067	0.490	Imputed
rs74635050	6	19,632,865	A	T	0.013	0.010	0.138	1.255	0.154	0.929	1.696	0.593	Imputed
rs75291050	6	19,633,015	T	C	0.043	0.041	0.561	1.050	0.084	0.891	1.238	0.257	Imputed
rs75257779	6	19,633,927	T	C	0.162	0.166	0.517	0.971	0.046	0.887	1.062	0.405	Imputed
rs78033174	6	19,633,975	C	T	0.162	0.166	0.517	0.971	0.046	0.887	1.062	0.405	Imputed
rs6912530	6	19,634,533	T	G	0.326	0.322	0.619	1.018	0.036	0.949	1.093	0.987	Imputed
rs77805393	6	19,634,654	G	A	0.162	0.166	0.501	0.970	0.046	0.886	1.061	0.411	Imputed
rs7774949	6	19,635,232	C	T	0.163	0.167	0.493	0.969	0.046	0.886	1.060	0.378	Imputed
rs60461667	6	19,635,778	A	C	0.154	0.159	0.383	0.960	0.047	0.876	1.052	0.338	Imputed
rs80210536	6	19,636,065	G	A	0.162	0.166	0.496	0.969	0.046	0.886	1.061	0.386	Imputed

rs77671200	6	19,636,240	G	A	0.162	0.166	0.512	0.970	0.046	0.887	1.062	0.381	Imputed
rs17546053	6	19,636,344	C	T	0.023	0.019	0.083	1.220	0.115	0.974	1.527	0.641	Imputed
rs9295445	6	19,636,541	A	G	0.091	0.082	0.049	1.123	0.059	1.000	1.261	0.632	Imputed
rs2328469	6	19,636,800	C	T	0.162	0.166	0.546	0.973	0.046	0.889	1.064	0.338	Imputed
rs2186048	6	19,636,958	A	G	0.341	0.338	0.644	1.017	0.036	0.948	1.090	0.911	Imputed
chr6:19637106:I	6	19,637,106	GA	G	0.394	0.403	0.257	0.962	0.035	0.899	1.029	0.404	Imputed
chr6:19637107:D	6	19,637,107	A	AAAG	0.394	0.403	0.257	0.962	0.035	0.898	1.029	0.415	Imputed
chr6:19637113:I	6	19,637,113	AG	A	0.394	0.402	0.286	0.964	0.035	0.901	1.031	0.474	Imputed
rs149903278	6	19,637,143	G	A	0.214	0.208	0.322	1.042	0.041	0.961	1.130	0.736	Imputed
rs138385718	6	19,637,328	G	A	0.012	0.010	0.166	1.241	0.156	0.913	1.686	0.379	Imputed
rs1936897	6	19,639,273	A	G	0.331	0.330	0.851	1.007	0.036	0.938	1.080	0.887	Imputed
rs58963316	6	19,639,552	T	C	0.162	0.166	0.513	0.970	0.046	0.887	1.062	0.303	Imputed
rs143242091	6	19,639,757	T	G	0.021	0.020	0.694	1.048	0.118	0.831	1.321	0.870	Imputed
chr6:19639962:I	6	19,639,962	CAT	C	0.407	0.411	0.532	0.979	0.034	0.915	1.047	0.319	Imputed
rs72836035	6	19,641,154	T	C	0.042	0.044	0.573	0.954	0.084	0.808	1.125	0.370	Imputed
rs73731526	6	19,642,250	C	T	0.162	0.166	0.503	0.970	0.046	0.886	1.061	0.295	Imputed
rs9460391	6	19,642,528	T	G	0.322	0.320	0.747	1.012	0.036	0.942	1.086	0.784	Genotyped
rs75244316	6	19,643,497	T	C	0.151	0.156	0.395	0.961	0.047	0.876	1.054	0.320	Imputed
rs7763387	6	19,643,628	T	C	0.319	0.316	0.703	1.014	0.036	0.944	1.089	0.887	Imputed
rs7743224	6	19,643,688	A	G	0.159	0.159	0.968	1.002	0.046	0.915	1.097	0.350	Genotyped
rs72836038	6	19,643,912	A	G	0.031	0.036	0.113	0.858	0.097	0.709	1.037	0.795	Imputed
rs1936896	6	19,644,251	G	A	0.162	0.165	0.578	0.975	0.046	0.891	1.067	0.302	Genotyped
rs35416132	6	19,644,906	C	T	0.106	0.097	0.061	1.109	0.055	0.995	1.235	0.603	Imputed
rs9358257	6	19,646,689	T	C	0.298	0.309	0.158	0.949	0.037	0.883	1.020	0.915	Imputed
rs62402577	6	19,646,872	T	C	0.029	0.031	0.432	0.924	0.101	0.758	1.126	0.396	Imputed
rs58401732	6	19,647,071	A	G	0.050	0.040	5.16E-03	1.247	0.079	1.068	1.456	0.744	Imputed
rs142579743	6	19,647,850	T	C	0.013	0.015	0.590	0.924	0.146	0.694	1.231	0.917	Imputed
rs9348389	6	19,647,981	A	G	0.320	0.330	0.189	0.954	0.036	0.888	1.024	0.778	Genotyped
rs138761612	6	19,648,223	T	C	0.050	0.040	5.32E-03	1.246	0.079	1.067	1.455	0.748	Imputed
rs16882416	6	19,649,678	A	T	0.050	0.041	7.70E-03	1.232	0.078	1.056	1.437	0.881	Imputed
rs2147042	6	19,649,733	A	G	0.372	0.376	0.600	0.982	0.035	0.917	1.052	0.670	Imputed
rs12191054	6	19,650,233	G	C	0.032	0.029	0.348	1.096	0.097	0.906	1.326	0.357	Imputed
rs10946335	6	19,650,426	G	A	0.319	0.330	0.153	0.950	0.036	0.884	1.019	0.831	Imputed
rs1892187	6	19,650,665	G	A	0.255	0.275	6.68E-03	0.900	0.039	0.835	0.971	0.889	Genotyped
rs2031254	6	19,651,916	G	T	0.489	0.482	0.475	1.024	0.034	0.959	1.095	0.885	Imputed
rs4710913	6	19,652,352	C	T	0.247	0.267	6.40E-03	0.899	0.039	0.833	0.971	0.920	Imputed
rs9356702	6	19,653,090	A	C	0.247	0.267	6.68E-03	0.899	0.039	0.833	0.971	0.868	Imputed
chr6:19653225:I	6	19,653,225	T	TA	0.370	0.372	0.753	0.989	0.035	0.923	1.059	0.798	Imputed
rs77659945	6	19,653,253	A	G	0.050	0.041	8.94E-03	1.228	0.079	1.052	1.434	0.787	Imputed
rs78005791	6	19,653,259	A	G	0.050	0.041	6.85E-03	1.236	0.078	1.060	1.441	0.756	Imputed

rs9465471	6	19,653,652	C	T	0.169	0.175	0.347	0.959	0.045	0.878	1.047	0.822	Imputed
rs2876556	6	19,653,687	T	G	0.309	0.319	0.184	0.953	0.037	0.887	1.023	0.929	Imputed
rs75678129	6	19,653,695	A	G	0.053	0.046	0.070	1.148	0.076	0.989	1.333	0.850	Imputed
rs56976565	6	19,653,781	A	G	0.051	0.041	4.83E-03	1.245	0.078	1.069	1.450	0.616	Imputed
rs2876555	6	19,653,999	C	T	0.298	0.309	0.150	0.948	0.037	0.882	1.019	0.745	Genotyped
rs74458379	6	19,654,596	C	G	0.050	0.041	8.69E-03	1.229	0.079	1.053	1.435	0.783	Imputed
rs4710914	6	19,654,713	T	G	0.302	0.313	0.133	0.946	0.037	0.880	1.017	0.857	Imputed
rs4710915	6	19,654,831	T	C	0.290	0.303	0.093	0.939	0.037	0.873	1.010	0.984	Imputed
rs9358258	6	19,655,292	C	A	0.291	0.303	0.106	0.942	0.037	0.876	1.013	0.958	Genotyped
rs4710916	6	19,655,643	C	T	0.357	0.367	0.187	0.955	0.035	0.891	1.023	0.679	Imputed
chr6:19656798:D	6	19,656,798	G	GT	0.052	0.041	1.89E-03	1.270	0.077	1.092	1.477	0.553	Imputed
rs142803040	6	19,656,932	A	G	0.039	0.031	9.88E-03	1.258	0.089	1.056	1.499	0.915	Imputed
rs9366304	6	19,657,361	T	G	0.241	0.263	2.55E-03	0.888	0.039	0.822	0.959	0.939	Imputed
rs74321027	6	19,658,778	C	G	0.017	0.015	0.305	1.147	0.133	0.883	1.489	0.410	Imputed
rs7761669	6	19,658,912	C	G	0.248	0.268	5.76E-03	0.898	0.039	0.832	0.969	0.807	Imputed
rs9348390	6	19,659,057	C	T	0.498	0.491	0.502	1.023	0.034	0.957	1.093	0.966	Genotyped
rs6932331	6	19,660,020	G	A	0.355	0.362	0.381	0.970	0.035	0.905	1.039	0.627	Imputed
rs17546547	6	19,660,584	C	T	0.018	0.017	0.855	1.024	0.128	0.797	1.315	0.098	Imputed
rs4712423	6	19,660,872	C	T	0.371	0.376	0.517	0.978	0.035	0.913	1.047	0.728	Genotyped
rs9465472	6	19,662,837	G	A	0.041	0.035	0.040	1.193	0.086	1.008	1.411	0.412	Imputed
rs9465473	6	19,662,852	T	C	0.041	0.035	0.041	1.192	0.086	1.007	1.410	0.415	Imputed
rs12197609	6	19,664,481	T	C	0.144	0.143	0.686	1.020	0.048	0.928	1.121	0.250	Genotyped
rs16882440	6	19,665,547	G	T	0.061	0.055	0.086	1.129	0.071	0.983	1.298	0.509	Imputed
rs9356703	6	19,665,871	T	C	0.419	0.425	0.490	0.977	0.034	0.913	1.045	0.583	Imputed
rs9460394	6	19,665,957	A	C	0.080	0.069	0.011	1.172	0.063	1.036	1.326	0.539	Genotyped
rs75860415	6	19,666,688	T	C	0.057	0.051	0.070	1.141	0.073	0.989	1.318	0.353	Imputed
rs9465474	6	19,666,716	C	T	0.092	0.080	8.82E-03	1.166	0.059	1.039	1.309	0.412	Imputed
chr6:19666794:I	6	19,666,794	T	TATAC	0.358	0.374	0.052	0.934	0.035	0.871	1.001	0.577	Imputed
rs11968417	6	19,666,800	T	C	0.417	0.430	0.116	0.947	0.034	0.886	1.014	0.654	Imputed
rs58969544	6	19,666,802	T	C	0.400	0.414	0.116	0.947	0.035	0.885	1.014	0.385	Imputed
rs12184086	6	19,666,815	T	A	0.017	0.014	0.114	1.229	0.132	0.949	1.591	0.023	Imputed
rs190439647	6	19,666,835	T	A	0.028	0.032	0.212	0.882	0.101	0.724	1.075	0.038	Imputed
rs114735690	6	19,666,971	A	G	0.049	0.043	0.063	1.158	0.079	0.992	1.351	0.272	Imputed
rs9366306	6	19,667,184	C	T	0.333	0.352	0.019	0.919	0.036	0.857	0.986	0.912	Imputed
rs9358259	6	19,667,419	A	G	0.343	0.363	0.014	0.916	0.036	0.855	0.982	0.873	Genotyped
chr6:19668011:D	6	19,668,011	A	AG	0.019	0.017	0.586	1.071	0.126	0.837	1.369	0.247	Imputed
rs11966592	6	19,668,012	A	G	0.017	0.015	0.372	1.125	0.132	0.868	1.459	0.763	Imputed
rs9358260	6	19,668,213	A	G	0.440	0.446	0.529	0.979	0.034	0.916	1.046	0.777	Genotyped
rs12664955	6	19,668,459	C	T	0.097	0.084	5.88E-03	1.172	0.058	1.047	1.312	0.418	Imputed
rs77158610	6	19,668,596	T	A	0.043	0.038	0.123	1.138	0.084	0.965	1.342	0.482	Imputed

rs4712424	6	19,668,671	C	T	0.290	0.316	1.28E-03	0.887	0.037	0.825	0.954	0.850	Imputed
rs12213997	6	19,668,969	C	T	0.142	0.140	0.665	1.021	0.049	0.929	1.123	0.272	Imputed
rs994750	6	19,669,083	T	C	0.305	0.323	0.021	0.919	0.037	0.855	0.987	0.933	Imputed
rs9368104	6	19,669,842	G	A	0.367	0.379	0.160	0.952	0.035	0.889	1.020	0.397	Genotyped
rs2876554	6	19,670,018	A	G	0.274	0.298	1.99E-03	0.890	0.038	0.826	0.958	0.758	Imputed
rs72836047	6	19,670,343	T	C	0.071	0.067	0.376	1.061	0.066	0.931	1.208	0.063	Imputed
rs73370862	6	19,670,701	A	G	0.010	0.011	0.784	0.955	0.170	0.685	1.331	0.347	Imputed
rs58144019	6	19,670,762	C	T	0.031	0.026	0.068	1.197	0.099	0.986	1.454	0.347	Imputed
rs2031255	6	19,670,986	G	A	0.437	0.441	0.651	0.985	0.034	0.921	1.053	0.209	Genotyped
rs970652	6	19,671,292	G	A	0.188	0.196	0.276	0.954	0.043	0.876	1.038	0.647	Imputed
rs2031256	6	19,671,401	G	C	0.405	0.414	0.299	0.965	0.034	0.902	1.032	0.396	Imputed
chr6:19671987:I	6	19,671,987	CA	C	0.011	0.010	0.463	1.127	0.163	0.819	1.551	0.271	Imputed
rs4712425	6	19,671,995	C	A	0.379	0.385	0.473	0.975	0.035	0.911	1.044	0.561	Imputed
rs9465476	6	19,672,138	T	C	0.153	0.152	0.960	1.002	0.047	0.914	1.099	0.210	Genotyped
rs73731531	6	19,672,376	C	T	0.009	0.012	0.136	0.776	0.172	0.555	1.086	0.103	Imputed
rs150450255	6	19,672,630	T	C	0.009	0.012	0.130	0.771	0.174	0.548	1.083	0.106	Imputed
rs4486001	6	19,672,632	C	G	0.214	0.225	0.140	0.941	0.041	0.868	1.020	0.426	Imputed
rs2031257	6	19,672,655	C	T	0.241	0.250	0.274	0.958	0.040	0.886	1.035	0.203	Imputed
rs62402603	6	19,672,715	T	A	0.053	0.053	0.760	0.977	0.076	0.842	1.134	0.281	Imputed
rs9356704	6	19,672,799	A	G	0.386	0.392	0.399	0.971	0.035	0.907	1.040	0.635	Genotyped
rs16882448	6	19,672,913	C	T	0.071	0.073	0.593	0.966	0.066	0.849	1.098	0.880	Genotyped
rs1936895	6	19,673,257	C	T	0.285	0.297	0.081	0.937	0.037	0.871	1.008	0.822	Genotyped
rs1936894	6	19,673,412	G	A	0.219	0.228	0.220	0.951	0.041	0.877	1.031	0.425	Imputed
rs41363444	6	19,673,475	G	T	0.084	0.074	0.023	1.151	0.062	1.020	1.298	0.472	Imputed
rs1033872	6	19,673,765	A	G	0.267	0.261	0.471	1.028	0.038	0.954	1.108	0.214	Genotyped
rs2031258	6	19,674,026	A	T	0.035	0.032	0.403	1.080	0.093	0.901	1.296	0.127	Imputed
rs139548842	6	19,674,137	C	A	0.014	0.019	0.021	0.720	0.143	0.545	0.953	0.427	Imputed
rs2031259	6	19,674,143	A	G	0.281	0.281	0.879	1.006	0.038	0.934	1.083	0.629	Imputed
rs55723014	6	19,674,443	T	C	0.451	0.462	0.202	0.957	0.034	0.895	1.024	0.442	Imputed
rs9465478	6	19,674,813	T	C	0.282	0.282	0.990	1.000	0.038	0.929	1.077	0.458	Imputed
rs17468433	6	19,675,239	A	C	0.088	0.077	0.015	1.158	0.060	1.029	1.303	0.649	Imputed
rs10456001	6	19,676,003	T	G	0.451	0.463	0.159	0.953	0.034	0.892	1.019	0.558	Imputed
MERGED_DEL_2_34677	6	19,676,316	A	;TCAGGAGTTTGAGACCAC	0.104	0.099	0.243	1.067	0.056	0.957	1.190	0.878	Imputed
rs56039346	6	19,676,329	G	C	0.091	0.083	0.068	1.114	0.059	0.992	1.251	0.734	Imputed
rs55726573	6	19,676,349	C	T	0.075	0.073	0.554	1.039	0.064	0.916	1.179	0.818	Imputed
rs55881281	6	19,676,357	T	G	0.071	0.069	0.635	1.032	0.066	0.907	1.174	0.949	Imputed
rs55713215	6	19,676,375	T	C	0.014	0.014	0.714	1.054	0.143	0.796	1.394	0.850	Imputed
rs2328439	6	19,676,778	C	A	0.132	0.133	0.611	0.975	0.050	0.884	1.075	0.514	Imputed
rs2328438	6	19,676,779	C	G	0.132	0.133	0.608	0.975	0.050	0.884	1.075	0.516	Imputed
rs71535509	6	19,677,058	T	C	0.132	0.134	0.586	0.973	0.050	0.882	1.073	0.528	Imputed

rs9465480	6	19,677,114	T	C	0.132	0.134	0.577	0.973	0.050	0.882	1.073	0.516	Imputed
rs9465481	6	19,677,224	A	G	0.295	0.300	0.467	0.973	0.037	0.905	1.047	0.247	Imputed
rs111769369	6	19,677,340	G	A	0.124	0.131	0.295	0.948	0.051	0.858	1.048	0.912	Imputed
rs9366307	6	19,677,496	A	G	0.037	0.034	0.326	1.092	0.090	0.916	1.303	0.161	Imputed
rs9368106	6	19,677,560	G	A	0.387	0.382	0.558	1.021	0.035	0.953	1.093	0.401	Imputed
rs9366308	6	19,677,593	T	C	0.036	0.033	0.258	1.108	0.091	0.927	1.323	0.124	Imputed
rs9358261	6	19,677,633	G	T	0.036	0.032	0.314	1.096	0.092	0.916	1.312	0.152	Imputed
rs9366309	6	19,677,824	T	C	0.397	0.395	0.860	1.006	0.035	0.940	1.077	0.416	Genotyped
rs9356705	6	19,677,864	T	C	0.036	0.033	0.241	1.112	0.091	0.931	1.329	0.220	Genotyped
rs17547028	6	19,677,912	G	A	0.124	0.131	0.272	0.945	0.051	0.855	1.045	0.911	Genotyped
rs12662463	6	19,678,604	G	A	0.124	0.131	0.281	0.946	0.051	0.856	1.046	0.920	Imputed
rs73370883	6	19,678,823	A	T	0.088	0.077	0.011	1.165	0.060	1.035	1.311	0.687	Imputed
rs140935428	6	19,679,105	C	T	0.015	0.015	0.742	1.046	0.138	0.799	1.371	0.731	Imputed
rs62402606	6	19,679,222	C	T	0.124	0.131	0.279	0.946	0.051	0.856	1.046	0.938	Imputed
chr6:19679770:D	6	19,679,770	G	GCA	0.197	0.192	0.402	1.037	0.043	0.953	1.127	0.959	Imputed
rs9358262	6	19,679,772	G	A	0.396	0.395	0.931	1.003	0.035	0.937	1.073	0.347	Genotyped
rs62402607	6	19,680,802	A	G	0.124	0.131	0.279	0.946	0.051	0.856	1.046	0.938	Imputed
rs62402608	6	19,680,885	G	A	0.124	0.131	0.279	0.946	0.051	0.856	1.046	0.938	Imputed
rs7767164	6	19,680,929	A	T	0.017	0.023	0.027	0.756	0.127	0.589	0.970	0.520	Imputed
rs9460398	6	19,681,366	C	T	0.088	0.077	0.013	1.161	0.060	1.032	1.307	0.777	Imputed
rs9465485	6	19,682,348	A	T	0.106	0.100	0.223	1.070	0.055	0.960	1.192	0.994	Imputed
rs12198138	6	19,682,385	G	A	0.035	0.032	0.356	1.089	0.092	0.908	1.305	0.094	Imputed
rs9465487	6	19,682,599	C	T	0.106	0.100	0.220	1.070	0.055	0.960	1.192	0.997	Imputed
rs62402609	6	19,682,968	T	C	0.124	0.131	0.285	0.947	0.051	0.857	1.047	0.928	Imputed
chr6:19683583:I	6	19,683,583	CT	C	0.270	0.264	0.458	1.029	0.038	0.955	1.109	0.274	Imputed
chr6:19683589:I	6	19,683,589	TC	T	0.162	0.161	0.858	1.008	0.046	0.921	1.103	0.224	Imputed
rs6917754	6	19,683,823	C	T	0.129	0.131	0.605	0.974	0.050	0.883	1.075	0.662	Imputed
rs62402610	6	19,683,916	A	G	0.124	0.131	0.285	0.947	0.051	0.857	1.047	0.928	Imputed
rs79946077	6	19,684,010	A	C	0.017	0.023	0.029	0.755	0.129	0.586	0.972	0.628	Imputed
rs62402611	6	19,684,035	T	C	0.124	0.131	0.285	0.947	0.051	0.857	1.047	0.928	Imputed
rs9348392	6	19,684,268	G	T	0.036	0.033	0.348	1.089	0.092	0.910	1.303	0.186	Imputed
rs7773280	6	19,684,306	G	A	0.397	0.396	0.915	1.004	0.035	0.938	1.074	0.417	Imputed
rs13204316	6	19,684,414	A	T	0.129	0.131	0.663	0.978	0.050	0.886	1.080	0.630	Imputed
chr6:19684513:I	6	19,684,513	CA	C	0.396	0.396	0.959	1.002	0.035	0.936	1.072	0.414	Imputed
rs7773944	6	19,684,596	C	A	0.379	0.391	0.132	0.949	0.035	0.886	1.016	0.308	Imputed
rs7774228	6	19,684,623	A	G	0.271	0.264	0.372	1.035	0.038	0.960	1.115	0.328	Imputed
chr6:19684753:D	6	19,684,753	G	GC	0.278	0.269	0.241	1.045	0.038	0.971	1.126	0.273	Imputed
rs7739541	6	19,684,794	G	A	0.271	0.264	0.362	1.035	0.038	0.961	1.116	0.323	Imputed
rs34629412	6	19,684,811	A	G	0.129	0.131	0.632	0.976	0.050	0.884	1.077	0.628	Imputed
rs62402612	6	19,685,095	C	T	0.382	0.387	0.488	0.976	0.035	0.912	1.045	0.498	Imputed

rs62402613	6	19,685,154	G	A	0.226	0.225	0.712	1.015	0.040	0.938	1.099	0.960	Imputed
rs71535510	6	19,685,227	T	C	0.129	0.131	0.667	0.979	0.050	0.887	1.080	0.628	Imputed
rs183448533	6	19,685,233	G	T	0.008	0.014	3.66E-03	0.592	0.183	0.413	0.846	0.914	Imputed
rs73370895	6	19,685,404	G	C	0.106	0.101	0.211	1.071	0.055	0.962	1.193	0.928	Imputed
chr6:19685460:I	6	19,685,460	TAAGG	T	0.106	0.100	0.226	1.069	0.055	0.960	1.191	0.996	Imputed
rs75492896	6	19,685,483	G	A	0.106	0.100	0.245	1.066	0.055	0.957	1.188	0.975	Imputed
MERGED_DEL_2_34682	6	19,685,576	A	AGAAGCGTCAAGTAAGAG	0.129	0.131	0.621	0.975	0.050	0.884	1.077	0.635	Imputed
rs76767869	6	19,685,675	G	A	0.017	0.023	0.026	0.754	0.127	0.588	0.968	0.556	Imputed
rs9350170	6	19,685,924	T	A	0.036	0.033	0.305	1.098	0.091	0.918	1.313	0.187	Imputed
rs6907630	6	19,686,038	A	C	0.019	0.024	0.042	0.780	0.122	0.614	0.992	0.504	Imputed
rs115378788	6	19,686,123	A	G	0.017	0.023	0.027	0.753	0.129	0.584	0.969	0.635	Imputed
rs9460399	6	19,686,212	T	C	0.017	0.023	0.025	0.752	0.127	0.586	0.965	0.529	Imputed
chr6:19686236:D	6	19,686,236	T	TTAA	0.017	0.022	0.084	0.803	0.128	0.625	1.031	0.575	Imputed
chr6:19686237:D	6	19,686,237	T	TAATA	0.017	0.023	0.025	0.752	0.127	0.586	0.965	0.529	Imputed
rs78695121	6	19,686,281	C	T	0.126	0.132	0.316	0.950	0.051	0.860	1.050	0.826	Imputed
rs16882470	6	19,687,502	A	G	0.088	0.077	0.014	1.158	0.060	1.030	1.303	0.787	Genotyped
rs9356706	6	19,688,317	T	A	0.165	0.164	0.993	1.000	0.046	0.915	1.094	0.286	Imputed
rs9366310	6	19,688,331	G	A	0.167	0.164	0.818	1.011	0.045	0.925	1.105	0.260	Genotyped
rs78087607	6	19,688,448	C	T	0.017	0.023	0.023	0.750	0.127	0.585	0.963	0.472	Imputed
rs62402614	6	19,688,752	A	G	0.124	0.131	0.288	0.947	0.051	0.857	1.047	0.926	Imputed
rs191478493	6	19,689,198	A	G	0.026	0.027	0.407	0.915	0.107	0.742	1.129	0.563	Imputed
rs9350171	6	19,689,211	A	G	0.035	0.032	0.426	1.076	0.092	0.898	1.290	0.133	Imputed
rs7762425	6	19,689,293	A	G	0.088	0.077	0.013	1.162	0.060	1.033	1.308	0.755	Imputed
rs150201742	6	19,689,693	G	C	0.020	0.019	0.864	1.021	0.122	0.805	1.296	0.862	Imputed
rs6932342	6	19,690,131	A	G	0.174	0.170	0.682	1.018	0.045	0.933	1.111	0.203	Imputed
rs111419279	6	19,691,268	T	G	0.022	0.016	0.014	1.337	0.119	1.060	1.687	0.853	Imputed
rs13218434	6	19,691,473	T	C	0.126	0.128	0.634	0.976	0.051	0.884	1.078	0.484	Genotyped
rs72657608	6	19,691,522	T	C	0.042	0.038	0.336	1.085	0.085	0.919	1.281	0.134	Imputed
rs9358263	6	19,692,306	A	C	0.045	0.040	0.176	1.118	0.082	0.951	1.314	0.140	Genotyped
rs62402615	6	19,692,354	T	C	0.124	0.131	0.260	0.944	0.051	0.854	1.044	0.878	Imputed
rs9368108	6	19,692,570	T	A	0.042	0.038	0.340	1.084	0.085	0.918	1.280	0.135	Imputed
rs6917874	6	19,693,101	T	A	0.088	0.078	0.014	1.159	0.060	1.030	1.303	0.760	Imputed
rs6919172	6	19,693,584	C	G	0.128	0.129	0.669	0.979	0.051	0.886	1.081	0.567	Imputed
rs6923485	6	19,693,784	A	C	0.128	0.129	0.673	0.979	0.051	0.886	1.081	0.565	Imputed
rs147518981	6	19,694,955	T	C	0.033	0.038	0.134	0.869	0.094	0.723	1.044	0.525	Imputed
chr6:19695367:I	6	19,695,367	TC	T	0.157	0.154	0.851	1.009	0.047	0.921	1.105	0.342	Imputed
chr6:19696097:I	6	19,696,097	CAG	C	0.009	0.014	9.50E-03	0.633	0.178	0.447	0.897	0.805	Imputed
rs10946337	6	19,696,395	C	T	0.405	0.402	0.734	1.012	0.034	0.946	1.082	0.358	Imputed
rs6921521	6	19,696,826	T	C	0.022	0.027	0.099	0.830	0.113	0.665	1.036	0.742	Imputed
rs9368109	6	19,697,189	C	T	0.023	0.028	0.123	0.842	0.111	0.677	1.048	0.620	Genotyped

rs13207071	6	19,697,374	G	T	0.124	0.124	0.881	0.992	0.051	0.897	1.097	0.740	Imputed
rs192470098	6	19,697,635	T	C	0.018	0.016	0.378	1.119	0.128	0.871	1.439	0.988	Imputed
rs7743576	6	19,697,898	T	G	0.168	0.164	0.762	1.014	0.045	0.928	1.108	0.217	Imputed
rs79098780	6	19,698,049	G	A	0.013	0.014	0.622	0.929	0.150	0.693	1.245	0.469	Imputed
rs59378765	6	19,698,207	A	G	0.040	0.035	0.181	1.123	0.087	0.947	1.332	0.138	Imputed
rs1936899	6	19,698,464	G	T	0.113	0.106	0.154	1.079	0.054	0.972	1.199	0.991	Genotyped
rs9348393	6	19,699,091	A	G	0.039	0.035	0.220	1.113	0.088	0.937	1.322	0.166	Imputed
rs11969832	6	19,699,096	A	T	0.040	0.035	0.199	1.118	0.087	0.943	1.326	0.146	Imputed
rs13199763	6	19,699,372	C	G	0.129	0.129	0.818	0.988	0.050	0.895	1.091	0.430	Imputed
rs6940685	6	19,700,166	C	T	0.446	0.431	0.055	1.067	0.034	0.999	1.141	0.795	Genotyped
chr6:19701618:I	6	19,701,618	CATAA	C	0.122	0.130	0.233	0.940	0.052	0.850	1.040	0.965	Imputed
chr6:19701688:D	6	19,701,688	A	AAT	0.168	0.162	0.361	1.042	0.045	0.954	1.139	0.296	Imputed
chr6:19701691:D	6	19,701,691	A	ATG	0.168	0.162	0.378	1.041	0.045	0.952	1.138	0.338	Imputed
rs4712427	6	19,701,912	C	T	0.168	0.163	0.426	1.037	0.045	0.949	1.133	0.416	Imputed
rs144257781	6	19,702,750	A	T	0.168	0.162	0.342	1.044	0.045	0.955	1.141	0.375	Imputed
rs12211390	6	19,702,884	C	T	0.366	0.354	0.106	1.059	0.035	0.988	1.135	0.953	Imputed
rs62402634	6	19,703,480	A	G	0.117	0.124	0.234	0.939	0.053	0.848	1.041	0.842	Imputed
rs78347727	6	19,703,552	C	T	0.050	0.055	0.140	0.892	0.078	0.766	1.038	0.733	Imputed
rs9465496	6	19,703,618	C	T	0.287	0.288	0.863	0.994	0.037	0.923	1.069	0.271	Genotyped
rs17481528	6	19,703,994	G	C	0.117	0.124	0.244	0.941	0.052	0.849	1.043	0.832	Imputed
rs4557503	6	19,704,477	A	G	0.343	0.321	3.30E-03	1.111	0.036	1.036	1.191	0.612	Imputed
rs12524518	6	19,704,545	C	A	0.152	0.130	1.03E-04	1.203	0.048	1.096	1.320	0.869	Imputed
rs9368110	6	19,704,592	A	G	0.071	0.064	0.115	1.111	0.067	0.975	1.266	0.132	Imputed
rs12664056	6	19,705,185	T	C	0.117	0.124	0.234	0.939	0.053	0.848	1.041	0.842	Imputed
rs17547439	6	19,705,389	G	T	0.117	0.124	0.236	0.940	0.053	0.848	1.042	0.840	Imputed
chr6:19705472:I	6	19,705,472	C	CA	0.035	0.039	0.268	0.904	0.091	0.756	1.081	0.776	Imputed
rs7740281	6	19,706,122	C	T	0.511	0.484	9.63E-04	1.118	0.034	1.046	1.195	0.883	Imputed
rs9358264	6	19,706,271	G	C	0.225	0.197	1.45E-05	1.193	0.041	1.102	1.293	0.417	Imputed
rs9358265	6	19,706,407	T	C	0.343	0.321	3.22E-03	1.111	0.036	1.036	1.192	0.602	Genotyped
rs9465498	6	19,706,553	C	T	0.117	0.124	0.240	0.940	0.052	0.849	1.042	0.794	Imputed
rs1016250	6	19,706,623	C	A	0.343	0.321	3.25E-03	1.111	0.036	1.036	1.191	0.614	Imputed
rs1016251	6	19,706,761	G	A	0.510	0.484	1.14E-03	1.116	0.034	1.045	1.193	0.917	Imputed
rs1016252	6	19,706,799	C	T	0.151	0.129	9.23E-05	1.205	0.048	1.097	1.323	0.865	Genotyped
chr6:19706830:D	6	19,706,830	G	GTCTT	0.117	0.124	0.230	0.939	0.053	0.847	1.041	0.847	Imputed
rs17547516	6	19,706,996	T	C	0.117	0.124	0.236	0.940	0.053	0.848	1.042	0.840	Imputed
chr6:19707218:D	6	19,707,218	G	GTC	0.012	0.017	0.025	0.712	0.152	0.529	0.959	0.508	Imputed
rs62402635	6	19,707,523	G	T	0.117	0.124	0.236	0.940	0.053	0.848	1.042	0.840	Imputed
rs144562069	6	19,707,526	C	G	0.117	0.124	0.236	0.940	0.053	0.848	1.042	0.840	Imputed
rs7764424	6	19,707,715	G	A	0.225	0.196	1.27E-05	1.195	0.041	1.103	1.294	0.400	Imputed
rs9460403	6	19,708,095	A	G	0.510	0.484	9.70E-04	1.118	0.034	1.046	1.195	0.912	Imputed

rs17547572	6	19,708,229	G	A	0.168	0.163	0.445	1.035	0.045	0.947	1.131	0.391	Genotyped
rs9366312	6	19,708,481	A	G	0.511	0.484	8.08E-04	1.120	0.034	1.048	1.197	0.890	Imputed
chr6:19709205:D	6	19,709,205	A	AAAAG	0.103	0.106	0.430	0.957	0.056	0.858	1.067	0.469	Imputed
rs2224813	6	19,709,354	A	G	0.071	0.064	0.112	1.112	0.066	0.976	1.266	0.144	Imputed
rs73729711	6	19,710,055	T	C	0.151	0.129	8.81E-05	1.205	0.048	1.098	1.323	0.869	Imputed
rs62402636	6	19,710,304	C	T	0.117	0.124	0.245	0.941	0.052	0.849	1.043	0.749	Imputed
rs62402637	6	19,710,427	A	G	0.117	0.124	0.242	0.941	0.052	0.849	1.042	0.751	Imputed
rs6926312	6	19,712,293	G	A	0.510	0.484	1.41E-03	1.114	0.034	1.043	1.190	0.860	Genotyped
rs12661949	6	19,712,746	C	A	0.073	0.066	0.109	1.111	0.066	0.977	1.264	0.233	Imputed
rs12660887	6	19,712,959	C	T	0.071	0.064	0.108	1.113	0.066	0.977	1.268	0.136	Imputed
rs12665187	6	19,713,335	T	C	0.242	0.230	0.097	1.068	0.040	0.988	1.154	0.909	Imputed
chr6:19713558:D	6	19,713,558	A	[illegible]ATATATATATATATATTTTT	0.023	0.020	0.162	1.171	0.113	0.938	1.461	0.396	Imputed
rs148600868	6	19,713,816	A	G	0.010	0.013	0.139	0.784	0.165	0.568	1.083	0.881	Imputed
rs12661747	6	19,715,107	G	T	0.116	0.124	0.177	0.931	0.053	0.840	1.033	0.927	Imputed
rs151318327	6	19,715,318	G	A	0.011	0.013	0.179	0.804	0.163	0.584	1.106	0.947	Imputed
chr6:19715366:D	6	19,715,366	C	CTTTGTT	0.244	0.231	0.071	1.074	0.040	0.994	1.160	0.784	Imputed
rs148126012	6	19,716,300	C	G	0.010	0.011	0.467	0.886	0.167	0.639	1.229	0.229	Imputed
chr6:19716347:I	6	19,716,347	GC	G	0.015	0.011	0.047	1.328	0.144	1.001	1.760	0.462	Imputed
chr6:19716884:I	6	19,716,884	CA	C	0.169	0.163	0.333	1.045	0.045	0.956	1.141	0.366	Imputed
chr6:19716885:I	6	19,716,885	AG	A	0.079	0.081	0.543	0.963	0.063	0.851	1.089	0.571	Imputed
chr6:19716890:I	6	19,716,890	AG	A	0.083	0.087	0.350	0.944	0.061	0.837	1.065	0.385	Imputed
rs2147044	6	19,717,442	A	G	0.245	0.232	0.059	1.077	0.039	0.997	1.164	0.744	Genotyped
rs7776268	6	19,717,876	C	T	0.243	0.229	0.057	1.078	0.040	0.998	1.165	0.883	Imputed
rs9350173	6	19,717,932	T	C	0.332	0.352	0.010	0.912	0.036	0.850	0.978	0.777	Imputed
rs9465502	6	19,718,072	C	A	0.113	0.117	0.504	0.965	0.053	0.869	1.071	0.774	Imputed
rs9465503	6	19,718,141	T	C	0.073	0.065	0.073	1.125	0.066	0.989	1.280	0.215	Imputed
rs9368112	6	19,718,157	C	T	0.498	0.474	3.08E-03	1.105	0.034	1.034	1.181	0.918	Imputed
rs9356707	6	19,718,349	C	G	0.513	0.485	8.00E-04	1.120	0.034	1.048	1.197	0.765	Imputed
rs74921218	6	19,718,698	T	C	0.021	0.021	0.759	1.037	0.118	0.823	1.306	0.184	Imputed
rs116430053	6	19,719,752	T	C	0.024	0.026	0.394	0.911	0.109	0.735	1.129	0.580	Imputed
rs140444135	6	19,719,965	C	G	0.014	0.016	0.544	0.917	0.142	0.694	1.212	0.614	Imputed
chr6:19720691:I	6	19,720,691	ACT	A	0.392	0.357	1.10E-05	1.165	0.035	1.088	1.247	0.632	Imputed
chr6:19720952:I	6	19,720,952	ACTT	A	0.397	0.362	1.29E-05	1.163	0.035	1.087	1.245	0.823	Imputed
chr6:19720954:I	6	19,720,954	TTC	T	0.397	0.362	1.34E-05	1.163	0.035	1.086	1.244	0.812	Imputed
chr6:19720955:I	6	19,720,955	TC	T	0.393	0.358	2.19E-05	1.159	0.035	1.082	1.240	0.922	Imputed
rs12208708	6	19,720,956	G	A	0.397	0.361	1.33E-05	1.163	0.035	1.086	1.245	0.782	Genotyped
rs6918737	6	19,721,681	A	T	0.242	0.229	0.065	1.076	0.040	0.996	1.163	0.973	Imputed
chr6:19721884:I	6	19,721,884	AG	A	0.125	0.118	0.206	1.068	0.052	0.965	1.181	0.026	Imputed
rs6911293	6	19,721,889	G	A	0.242	0.229	0.064	1.076	0.040	0.996	1.163	0.971	Imputed
rs192632029	6	19,722,043	T	C	0.034	0.037	0.254	0.899	0.093	0.750	1.079	0.861	Imputed

rs74792647	6	19,723,164	G	T	0.011	0.012	0.632	0.925	0.162	0.673	1.272	0.968	Imputed
rs6941684	6	19,723,606	A	T	0.071	0.064	0.076	1.125	0.066	0.988	1.282	0.129	Imputed
rs142347518	6	19,723,991	A	G	0.011	0.012	0.632	0.925	0.162	0.673	1.272	0.968	Imputed
rs6909668	6	19,724,989	C	T	0.431	0.397	7.61E-05	1.145	0.034	1.071	1.225	0.698	Imputed
rs113331178	6	19,725,034	G	A	0.153	0.131	8.94E-05	1.204	0.047	1.097	1.321	0.946	Imputed
rs149529723	6	19,725,035	T	C	0.212	0.208	0.614	1.021	0.041	0.942	1.107	0.187	Imputed
rs76656244	6	19,725,384	G	A	0.153	0.132	1.65E-04	1.195	0.047	1.089	1.311	0.943	Imputed
rs12528349	6	19,725,973	C	T	0.154	0.132	1.29E-04	1.198	0.047	1.092	1.315	0.968	Imputed
rs9465505	6	19,726,083	G	A	0.443	0.469	2.43E-03	0.902	0.034	0.844	0.964	0.411	Imputed
rs9366313	6	19,726,320	C	A	0.071	0.064	0.082	1.122	0.066	0.986	1.278	0.124	Genotyped
rs72836058	6	19,726,325	T	A	0.212	0.208	0.618	1.021	0.041	0.941	1.107	0.188	Imputed
rs10456002	6	19,726,558	C	A	0.071	0.064	0.080	1.124	0.066	0.987	1.280	0.126	Imputed
rs12527122	6	19,727,378	T	C	0.153	0.131	7.14E-05	1.207	0.047	1.100	1.324	0.945	Imputed
rs80315459	6	19,727,384	G	C	0.115	0.124	0.161	0.929	0.053	0.838	1.030	0.661	Imputed
rs6905308	6	19,728,081	T	C	0.230	0.200	9.28E-06	1.197	0.041	1.105	1.296	0.548	Imputed
rs6928321	6	19,728,283	C	T	0.230	0.200	9.48E-06	1.196	0.041	1.105	1.295	0.546	Imputed
rs12529365	6	19,728,548	G	T	0.154	0.132	1.16E-04	1.200	0.047	1.094	1.316	0.956	Imputed
chr6:19728565:D	6	19,728,565	T	TGAGA	0.155	0.133	1.23E-04	1.199	0.047	1.093	1.315	0.878	Imputed
rs9460406	6	19,728,835	C	G	0.230	0.200	9.90E-06	1.196	0.041	1.105	1.295	0.543	Imputed
rs9356708	6	19,729,003	G	T	0.343	0.322	5.55E-03	1.104	0.036	1.029	1.184	0.840	Genotyped
rs7769632	6	19,729,381	C	T	0.159	0.136	9.65E-05	1.200	0.047	1.095	1.315	0.694	Genotyped
rs61681081	6	19,729,747	C	T	0.071	0.064	0.077	1.125	0.066	0.988	1.281	0.118	Imputed
rs12176555	6	19,730,522	G	T	0.071	0.064	0.077	1.125	0.066	0.988	1.281	0.118	Imputed
rs11753051	6	19,730,659	T	A	0.206	0.202	0.678	1.018	0.042	0.937	1.105	0.222	Imputed
rs114548458	6	19,731,328	T	C	0.015	0.017	0.394	0.888	0.139	0.676	1.166	0.233	Imputed
rs12524009	6	19,731,384	C	T	0.154	0.132	1.51E-04	1.196	0.047	1.090	1.312	0.961	Imputed
rs9460407	6	19,731,834	G	A	0.229	0.200	1.18E-05	1.194	0.041	1.103	1.293	0.545	Imputed
rs17547906	6	19,732,380	C	T	0.154	0.132	1.51E-04	1.196	0.047	1.090	1.312	0.961	Imputed
rs6920825	6	19,732,492	C	T	0.227	0.198	7.72E-06	1.199	0.041	1.108	1.299	0.384	Genotyped
rs111424117	6	19,733,035	G	A	0.154	0.132	1.51E-04	1.196	0.047	1.090	1.312	0.961	Imputed
rs17547948	6	19,733,321	A	T	0.154	0.132	1.51E-04	1.196	0.047	1.090	1.312	0.961	Imputed
rs9368114	6	19,733,538	C	T	0.230	0.200	9.90E-06	1.196	0.041	1.105	1.295	0.543	Imputed
rs6904484	6	19,733,694	G	A	0.230	0.200	1.08E-05	1.195	0.041	1.104	1.294	0.551	Imputed
rs62402640	6	19,733,783	G	A	0.115	0.124	0.159	0.928	0.053	0.837	1.030	0.644	Imputed
rs6932015	6	19,733,894	C	T	0.230	0.200	9.90E-06	1.196	0.041	1.105	1.295	0.543	Imputed
rs6932208	6	19,734,011	C	T	0.230	0.200	9.90E-06	1.196	0.041	1.105	1.295	0.543	Imputed
rs6909973	6	19,734,274	C	A	0.449	0.474	2.86E-03	0.904	0.034	0.845	0.966	0.415	Imputed
rs74865905	6	19,734,853	A	G	0.115	0.124	0.146	0.926	0.053	0.835	1.027	0.662	Imputed
rs77956947	6	19,735,065	C	T	0.115	0.124	0.146	0.926	0.053	0.835	1.027	0.662	Imputed
rs2328437	6	19,735,805	T	C	0.154	0.132	1.73E-04	1.194	0.047	1.088	1.310	0.975	Imputed

rs714876	6	19,736,085	T	C	0.012	0.014	0.317	0.858	0.153	0.635	1.158	0.604	Imputed
chr6:19736267:D	6	19,736,267	A	ATCTTT	0.154	0.132	1.73E-04	1.194	0.047	1.088	1.310	0.975	Imputed
rs12525475	6	19,736,351	G	A	0.154	0.132	1.73E-04	1.194	0.047	1.088	1.310	0.975	Imputed
rs6922513	6	19,736,405	C	G	0.230	0.200	1.01E-05	1.196	0.041	1.104	1.295	0.541	Imputed
rs2328436	6	19,736,727	C	T	0.450	0.474	3.51E-03	0.906	0.034	0.847	0.968	0.414	Imputed
rs115462120	6	19,737,048	G	T	0.012	0.011	0.702	1.061	0.156	0.782	1.441	0.448	Imputed
rs9350174	6	19,737,117	G	A	0.230	0.200	1.15E-05	1.194	0.041	1.103	1.293	0.562	Imputed
chr6:19737128:D	6	19,737,128	A	AAAG	0.230	0.200	1.13E-05	1.195	0.041	1.103	1.293	0.564	Imputed
chr6:19737132:D	6	19,737,132	A	AAGC	0.230	0.200	1.13E-05	1.195	0.041	1.103	1.293	0.564	Imputed
rs9358266	6	19,737,175	T	A	0.227	0.197	8.42E-06	1.199	0.041	1.107	1.298	0.379	Imputed
chr6:19737600:D	6	19,737,600	T	TTTTG	0.074	0.067	0.081	1.121	0.065	0.986	1.273	0.296	Imputed
rs62402641	6	19,737,870	A	G	0.115	0.124	0.146	0.926	0.053	0.835	1.027	0.662	Imputed
rs1209817	6	19,738,241	C	A	0.033	0.035	0.563	0.947	0.095	0.786	1.140	0.323	Imputed
rs62402642	6	19,738,302	C	A	0.115	0.124	0.146	0.926	0.053	0.835	1.027	0.662	Imputed
rs12200680	6	19,738,691	G	A	0.036	0.032	0.134	1.147	0.092	0.959	1.373	0.024	Imputed
rs149713408	6	19,739,377	C	A	0.033	0.036	0.334	0.914	0.094	0.761	1.098	0.566	Imputed
rs6920225	6	19,739,378	G	T	0.200	0.197	0.783	1.012	0.042	0.931	1.099	0.220	Imputed
rs9637961	6	19,739,481	T	C	0.072	0.064	0.057	1.134	0.066	0.997	1.291	0.158	Imputed
rs9460408	6	19,739,943	C	T	0.231	0.203	1.87E-05	1.189	0.040	1.098	1.287	0.563	Genotyped
chr6:19740601:D	6	19,740,601	T	TGCTTGCTTGCTG	0.225	0.196	1.84E-05	1.191	0.041	1.099	1.290	0.326	Imputed
rs12662702	6	19,741,952	T	C	0.122	0.131	0.160	0.930	0.051	0.841	1.029	0.365	Imputed
rs1209816	6	19,742,243	T	C	0.037	0.038	0.893	0.988	0.089	0.829	1.177	0.611	Genotyped
rs9460409	6	19,742,264	C	T	0.072	0.064	0.060	1.133	0.066	0.995	1.289	0.154	Imputed
chr6:19742417:D	6	19,742,417	T	TTTTTC	0.036	0.032	0.126	1.151	0.092	0.962	1.377	0.022	Imputed
rs6939745	6	19,742,526	G	A	0.230	0.200	1.21E-05	1.194	0.041	1.103	1.292	0.558	Imputed
rs78937950	6	19,742,680	A	G	0.075	0.064	6.47E-03	1.193	0.065	1.051	1.355	0.964	Imputed
rs9356709	6	19,742,814	C	T	0.451	0.476	2.40E-03	0.902	0.034	0.844	0.964	0.494	Imputed
rs9366314	6	19,742,863	C	G	0.450	0.474	3.31E-03	0.905	0.034	0.847	0.967	0.419	Imputed
rs111648257	6	19,742,926	A	C	0.154	0.132	1.73E-04	1.194	0.047	1.088	1.310	0.975	Imputed
chr6:19742984:I	6	19,742,984	TC	T	0.226	0.197	8.24E-06	1.199	0.041	1.107	1.299	0.440	Imputed
rs67389033	6	19,743,070	T	C	0.206	0.202	0.641	1.020	0.042	0.939	1.107	0.212	Imputed
rs9358267	6	19,743,486	G	A	0.072	0.065	0.069	1.128	0.066	0.991	1.284	0.168	Genotyped
rs72836061	6	19,743,835	T	C	0.206	0.202	0.643	1.020	0.042	0.939	1.107	0.213	Imputed
rs9368116	6	19,743,894	T	C	0.227	0.197	8.23E-06	1.199	0.041	1.107	1.298	0.368	Imputed
rs9465509	6	19,744,129	A	G	0.452	0.477	2.96E-03	0.904	0.034	0.846	0.966	0.567	Imputed
rs145444299	6	19,744,168	A	G	0.011	0.013	0.309	0.850	0.160	0.621	1.163	0.478	Imputed
chr6:19744464:D	6	19,744,464	C	CTTTA	0.206	0.202	0.636	1.020	0.042	0.940	1.107	0.211	Imputed
chr6:19744858:D	6	19,744,858	G	GTC	0.073	0.065	0.042	1.142	0.066	1.005	1.299	0.103	Imputed
rs141986645	6	19,745,083	G	T	0.206	0.202	0.649	1.019	0.042	0.939	1.106	0.207	Imputed
rs139643115	6	19,745,853	C	A	0.154	0.132	1.77E-04	1.194	0.047	1.088	1.310	0.978	Imputed

rs6920339	6	19,745,951	C	G	0.337	0.320	0.026	1.083	0.036	1.010	1.162	0.986	Imputed
rs11757933	6	19,746,113	T	G	0.200	0.196	0.543	1.026	0.042	0.944	1.115	0.277	Imputed
rs11757897	6	19,746,115	G	C	0.200	0.196	0.562	1.025	0.042	0.943	1.113	0.264	Imputed
rs6900502	6	19,746,253	A	T	0.230	0.200	1.23E-05	1.194	0.041	1.103	1.292	0.541	Imputed
rs9465510	6	19,746,551	G	T	0.226	0.197	1.20E-05	1.195	0.041	1.103	1.295	0.407	Imputed
rs145893044	6	19,746,592	G	T	0.205	0.203	0.812	1.010	0.042	0.930	1.096	0.202	Imputed
rs138252395	6	19,746,634	T	C	0.115	0.124	0.144	0.926	0.053	0.835	1.027	0.664	Imputed
chr6:19746737:D	6	19,746,737	A	AT	0.329	0.312	0.017	1.090	0.036	1.015	1.169	0.644	Imputed
chr6:19746740:D	6	19,746,740	T	TTA	0.281	0.251	4.07E-05	1.168	0.038	1.085	1.258	0.354	Imputed
chr6:19746759:D	6	19,746,759	T	TC	0.233	0.205	2.77E-05	1.184	0.040	1.094	1.281	0.485	Imputed
rs141541811	6	19,746,760	T	C	0.217	0.193	4.37E-04	1.157	0.041	1.067	1.255	0.458	Imputed
rs7743832	6	19,746,848	C	T	0.230	0.200	1.20E-05	1.194	0.041	1.103	1.293	0.543	Imputed
rs72836063	6	19,747,045	A	C	0.206	0.202	0.691	1.017	0.042	0.937	1.104	0.190	Imputed
rs6456258	6	19,748,048	G	T	0.450	0.473	4.80E-03	0.909	0.034	0.850	0.971	0.370	Imputed
rs11759345	6	19,748,549	A	G	0.206	0.202	0.667	1.018	0.042	0.938	1.105	0.207	Genotyped
rs9465511	6	19,748,695	G	C	0.230	0.201	1.38E-05	1.192	0.040	1.101	1.291	0.513	Imputed
rs9460410	6	19,748,716	A	G	0.230	0.200	1.23E-05	1.194	0.041	1.103	1.292	0.557	Imputed
rs4141748	6	19,748,726	G	C	0.072	0.064	0.054	1.136	0.066	0.998	1.293	0.150	Imputed
chr6:19749139:I	6	19,749,139	TA	T	0.115	0.124	0.109	0.919	0.053	0.829	1.019	0.736	Imputed
rs9465512	6	19,750,184	T	C	0.230	0.200	1.13E-05	1.195	0.041	1.103	1.293	0.548	Imputed
rs9465513	6	19,750,222	A	T	0.230	0.200	1.13E-05	1.195	0.041	1.103	1.293	0.548	Imputed
rs74657954	6	19,751,487	G	A	0.116	0.124	0.159	0.929	0.053	0.838	1.030	0.814	Imputed
rs10806906	6	19,751,516	C	T	0.451	0.476	2.47E-03	0.902	0.034	0.844	0.964	0.507	Imputed
rs12192729	6	19,751,668	A	G	0.226	0.198	1.84E-05	1.190	0.041	1.099	1.289	0.605	Genotyped
rs12197522	6	19,752,263	G	A	0.075	0.069	0.112	1.108	0.065	0.976	1.258	0.198	Genotyped
rs55949111	6	19,752,648	T	C	0.101	0.091	0.056	1.114	0.057	0.997	1.245	0.632	Imputed
rs75026206	6	19,752,656	T	C	0.051	0.040	2.22E-03	1.271	0.078	1.090	1.482	0.710	Imputed
rs74892329	6	19,752,736	C	T	0.027	0.022	0.045	1.235	0.106	1.003	1.519	0.306	Imputed
rs7766034	6	19,753,061	T	C	0.464	0.428	2.41E-05	1.154	0.034	1.080	1.234	0.810	Genotyped
rs71558200	6	19,753,363	C	T	0.066	0.072	0.124	0.901	0.068	0.789	1.029	0.670	Imputed
rs12194904	6	19,753,932	C	T	0.076	0.070	0.202	1.086	0.064	0.957	1.232	0.281	Imputed
rs35185749	6	19,754,511	T	C	0.101	0.091	0.056	1.115	0.057	0.997	1.245	0.635	Imputed
rs10434840	6	19,755,734	A	G	0.074	0.069	0.163	1.095	0.065	0.964	1.244	0.219	Imputed
rs9350175	6	19,756,008	G	T	0.075	0.069	0.170	1.093	0.065	0.963	1.241	0.226	Genotyped
rs79963847	6	19,756,203	T	C	0.101	0.091	0.058	1.113	0.057	0.996	1.244	0.649	Imputed
rs11756360	6	19,756,295	C	A	0.033	0.036	0.247	0.897	0.094	0.746	1.079	0.346	Imputed
rs78296116	6	19,756,305	A	C	0.101	0.091	0.058	1.113	0.057	0.996	1.244	0.649	Imputed
rs9350176	6	19,757,013	A	G	0.075	0.069	0.163	1.095	0.065	0.964	1.243	0.230	Imputed
rs9368117	6	19,757,028	C	T	0.465	0.429	1.84E-05	1.156	0.034	1.082	1.236	0.751	Imputed
rs62402643	6	19,757,581	G	T	0.082	0.097	4.71E-03	0.842	0.061	0.747	0.949	0.660	Imputed

rs142467799	6	19,757,847	A	G	0.052	0.042	2.28E-03	1.266	0.077	1.088	1.473	0.658	Imputed
rs7755728	6	19,758,569	G	C	0.074	0.068	0.151	1.098	0.065	0.967	1.247	0.313	Imputed
rs7776015	6	19,758,598	G	T	0.160	0.140	7.11E-04	1.171	0.047	1.069	1.283	0.497	Imputed
chr6:19758810:D	6	19,758,810	C	CCA	0.074	0.068	0.154	1.097	0.065	0.966	1.247	0.258	Imputed
rs75778554	6	19,759,086	T	C	0.058	0.049	0.012	1.203	0.073	1.042	1.389	0.488	Imputed
rs61300526	6	19,759,087	A	G	0.200	0.194	0.378	1.038	0.042	0.955	1.128	0.154	Imputed
rs76327413	6	19,759,296	G	A	0.009	0.011	0.458	0.878	0.176	0.622	1.239	0.816	Imputed
rs9295447	6	19,759,362	C	T	0.135	0.138	0.477	0.965	0.050	0.876	1.064	0.767	Genotyped
rs9295448	6	19,759,587	C	T	0.277	0.265	0.101	1.064	0.038	0.988	1.146	0.471	Genotyped
rs10484640	6	19,759,897	G	T	0.277	0.264	0.087	1.067	0.038	0.991	1.149	0.454	Imputed
rs11758935	6	19,760,008	G	A	0.200	0.193	0.366	1.039	0.042	0.956	1.129	0.141	Genotyped
chr6:19760594:D	6	19,760,594	A	AT	0.084	0.099	4.72E-03	0.843	0.060	0.749	0.949	0.764	Imputed
rs74457659	6	19,760,725	T	C	0.098	0.089	0.079	1.106	0.057	0.989	1.237	0.950	Imputed
rs6936425	6	19,761,141	C	A	0.451	0.475	4.99E-03	0.909	0.034	0.850	0.972	0.530	Imputed
rs6916251	6	19,761,215	C	T	0.464	0.427	8.92E-06	1.163	0.034	1.088	1.243	0.614	Genotyped
rs6916589	6	19,761,363	G	T	0.128	0.114	8.42E-03	1.144	0.051	1.035	1.264	0.922	Imputed
rs6456259	6	19,761,718	G	A	0.181	0.156	8.22E-05	1.190	0.044	1.091	1.299	0.877	Genotyped
rs9368118	6	19,762,352	A	G	0.450	0.473	5.92E-03	0.911	0.034	0.852	0.974	0.552	Imputed
rs9368119	6	19,762,411	C	T	0.278	0.266	0.102	1.064	0.038	0.988	1.145	0.474	Imputed
rs62402645	6	19,762,854	G	A	0.031	0.034	0.431	0.927	0.097	0.766	1.121	0.537	Imputed
rs6456260	6	19,762,952	T	G	0.460	0.423	1.01E-05	1.162	0.034	1.087	1.242	0.621	Imputed
rs9350178	6	19,763,653	G	A	0.278	0.266	0.102	1.064	0.038	0.988	1.145	0.474	Imputed
rs9350179	6	19,764,095	A	G	0.458	0.421	7.50E-06	1.164	0.034	1.089	1.245	0.596	Imputed
rs115810513	6	19,764,251	G	A	0.032	0.030	0.486	1.070	0.097	0.885	1.295	0.077	Imputed
rs72836068	6	19,764,677	A	C	0.018	0.018	0.997	1.000	0.128	0.778	1.284	0.756	Imputed
rs113181793	6	19,771,435	C	T	0.081	0.065	1.03E-04	1.276	0.063	1.128	1.443	0.622	Imputed
rs148757596	6	19,771,460	C	T	0.024	0.027	0.261	0.885	0.109	0.715	1.096	0.203	Imputed
rs1121384	6	19,771,507	G	A	0.458	0.421	9.63E-06	1.162	0.034	1.087	1.242	0.573	Imputed
rs3900464	6	19,771,601	A	G	0.278	0.265	0.089	1.066	0.038	0.990	1.148	0.446	Imputed
rs139428266	6	19,771,613	T	C	0.082	0.066	1.10E-04	1.273	0.063	1.126	1.440	0.698	Imputed
rs12201836	6	19,771,789	A	G	0.286	0.304	0.021	0.918	0.037	0.853	0.987	0.380	Imputed
rs9366315	6	19,771,902	C	T	0.078	0.071	0.133	1.101	0.064	0.972	1.247	0.351	Imputed
rs9465516	6	19,771,998	C	A	0.464	0.427	7.45E-06	1.164	0.034	1.089	1.244	0.644	Imputed
rs9368120	6	19,772,112	A	G	0.078	0.071	0.133	1.101	0.064	0.972	1.247	0.351	Imputed
rs76807328	6	19,772,322	A	G	0.030	0.025	0.033	1.238	0.100	1.017	1.506	0.925	Imputed
chr6:19772947:I	6	19,772,947	TAAA	T	0.130	0.127	0.727	1.018	0.050	0.922	1.124	0.408	Imputed
rs61082225	6	19,772,954	A	T	0.042	0.049	0.063	0.856	0.084	0.726	1.008	0.685	Imputed
rs6901949	6	19,773,156	G	A	0.051	0.040	1.17E-03	1.289	0.078	1.106	1.502	0.485	Imputed
rs6925511	6	19,773,553	C	T	0.465	0.427	6.89E-06	1.165	0.034	1.090	1.245	0.625	Imputed
rs6908034	6	19,773,930	A	G	0.181	0.157	6.80E-05	1.193	0.044	1.094	1.301	0.831	Imputed

rs6907875	6	19,773,953	A	C	0.200	0.193	0.290	1.046	0.042	0.963	1.136	0.184	Imputed
rs9348394	6	19,774,484	C	T	0.279	0.264	0.049	1.077	0.038	1.000	1.160	0.518	Imputed
rs9465519	6	19,775,615	T	A	0.271	0.261	0.148	1.057	0.038	0.981	1.139	0.961	Imputed
rs112743661	6	19,776,346	T	C	0.030	0.025	0.033	1.238	0.100	1.017	1.506	0.925	Imputed
rs6901079	6	19,776,659	C	T	0.262	0.249	0.067	1.073	0.039	0.995	1.158	0.848	Genotyped
rs6925962	6	19,777,055	A	G	0.459	0.488	7.22E-04	0.892	0.034	0.834	0.953	0.486	Imputed
rs76450105	6	19,777,209	C	A	0.023	0.022	0.600	1.061	0.113	0.851	1.322	0.644	Imputed
rs9350181	6	19,777,376	T	C	0.278	0.264	0.051	1.077	0.038	1.000	1.160	0.505	Imputed
rs75395558	6	19,777,385	C	T	0.017	0.014	0.112	1.234	0.134	0.950	1.604	0.108	Imputed
rs11758644	6	19,777,468	A	G	0.200	0.192	0.278	1.047	0.042	0.964	1.138	0.168	Imputed
rs9465521	6	19,777,786	A	G	0.256	0.243	0.058	1.076	0.039	0.998	1.162	0.852	Imputed
rs143331957	6	19,777,997	A	G	0.009	0.013	0.056	0.718	0.175	0.510	1.011	0.905	Imputed
rs2223362	6	19,778,171	A	C	0.459	0.420	3.60E-06	1.170	0.034	1.095	1.251	0.620	Imputed
rs2206036	6	19,778,399	C	T	0.078	0.071	0.093	1.112	0.063	0.982	1.260	0.320	Imputed
rs6933068	6	19,778,618	T	A	0.200	0.192	0.282	1.047	0.042	0.963	1.137	0.163	Imputed
rs142980251	6	19,778,783	A	G	0.012	0.014	0.395	0.878	0.153	0.650	1.185	0.770	Imputed
rs6456263	6	19,778,863	T	C	0.153	0.160	0.292	0.952	0.047	0.868	1.043	0.858	Imputed
rs139916517	6	19,779,238	T	C	0.030	0.025	0.033	1.238	0.100	1.017	1.506	0.925	Imputed
chr6:19779701:I	6	19,779,701	A	ACT	0.439	0.465	2.23E-03	0.901	0.034	0.843	0.963	0.549	Imputed
chr6:19779702:I	6	19,779,702	C	CTT	0.439	0.464	3.35E-03	0.905	0.034	0.847	0.967	0.512	Imputed
rs76250300	6	19,780,205	C	T	0.024	0.022	0.547	1.070	0.112	0.859	1.332	0.617	Imputed
rs9358270	6	19,780,584	C	G	0.451	0.476	2.84E-03	0.904	0.034	0.845	0.966	0.513	Imputed
rs9350182	6	19,780,879	G	A	0.278	0.264	0.048	1.078	0.038	1.001	1.161	0.483	Imputed
rs9465523	6	19,780,956	A	G	0.137	0.141	0.582	0.973	0.049	0.884	1.072	0.877	Genotyped
rs113986290	6	19,781,009	T	C	0.030	0.025	0.034	1.236	0.100	1.016	1.502	0.787	Imputed
rs62402649	6	19,781,550	T	C	0.084	0.098	4.81E-03	0.844	0.060	0.749	0.950	0.697	Imputed
rs62402650	6	19,781,563	T	C	0.084	0.098	4.81E-03	0.844	0.060	0.749	0.950	0.697	Imputed
rs73374867	6	19,781,802	T	C	0.030	0.025	0.028	1.245	0.100	1.024	1.514	0.851	Imputed
rs9350183	6	19,781,834	G	A	0.079	0.072	0.072	1.121	0.063	0.990	1.268	0.259	Imputed
chr6:19781906:D	6	19,781,906	G	GT	0.330	0.306	1.32E-03	1.123	0.036	1.046	1.205	0.651	Imputed
rs2078462	6	19,782,173	T	C	0.278	0.264	0.047	1.078	0.038	1.001	1.161	0.482	Imputed
rs2328435	6	19,782,196	C	G	0.199	0.192	0.307	1.044	0.042	0.961	1.135	0.127	Imputed
rs74795718	6	19,782,299	A	G	0.098	0.089	0.094	1.101	0.057	0.984	1.231	0.953	Imputed
rs115759209	6	19,782,370	C	G	0.014	0.017	0.091	0.785	0.144	0.592	1.040	0.865	Imputed
rs143351225	6	19,782,599	A	G	0.024	0.022	0.547	1.070	0.112	0.859	1.332	0.617	Imputed
rs79028848	6	19,783,153	A	G	0.097	0.089	0.114	1.095	0.057	0.979	1.225	0.908	Imputed
rs9350184	6	19,783,157	T	C	0.285	0.268	0.027	1.087	0.038	1.010	1.170	0.568	Imputed
rs12212949	6	19,783,325	T	C	0.029	0.027	0.382	1.093	0.102	0.896	1.335	0.073	Imputed
rs12189759	6	19,785,137	A	G	0.029	0.027	0.376	1.095	0.102	0.897	1.336	0.074	Imputed
rs62402651	6	19,785,359	G	T	0.085	0.100	2.37E-03	0.833	0.060	0.740	0.937	0.945	Imputed

rs7739264	6	19,785,588	C	T	0.450	0.474	3.11E-03	0.904	0.034	0.846	0.967	0.457	Genotyped
chr6:19786591:D	6	19,786,591	C	CATT	0.027	0.024	0.348	1.103	0.105	0.898	1.354	0.537	Imputed
chr6:19786668:I	6	19,786,668	G	GTA	0.457	0.482	2.29E-03	0.902	0.034	0.844	0.964	0.368	Imputed
chr6:19786670:I	6	19,786,670	ATG	A	0.520	0.488	1.75E-04	1.136	0.034	1.063	1.214	0.307	Imputed
rs6456264	6	19,786,682	A	T	0.445	0.428	0.031	1.076	0.034	1.007	1.150	0.396	Imputed
rs7745218	6	19,786,692	A	G	0.084	0.097	0.012	0.860	0.060	0.764	0.968	0.534	Imputed
rs116283561	6	19,786,801	A	G	0.014	0.017	0.116	0.798	0.144	0.601	1.058	0.765	Imputed
rs6456265	6	19,786,950	T	C	0.450	0.474	3.15E-03	0.905	0.034	0.846	0.967	0.447	Imputed
rs72657611	6	19,787,544	G	T	0.198	0.191	0.373	1.039	0.043	0.956	1.129	0.116	Imputed
chr6:19787653:D	6	19,787,653	ATT	A	0.467	0.490	4.98E-03	0.909	0.034	0.851	0.972	0.598	Imputed
rs181635615	6	19,787,743	A	G	0.030	0.025	0.038	1.231	0.101	1.011	1.500	0.849	Imputed
rs9348395	6	19,787,928	A	G	0.079	0.072	0.068	1.122	0.063	0.992	1.270	0.204	Imputed
rs112545594	6	19,788,182	T	G	0.018	0.018	0.894	0.983	0.128	0.766	1.262	0.287	Imputed
rs75464772	6	19,788,226	T	C	0.015	0.011	0.020	1.379	0.140	1.048	1.815	0.101	Imputed
rs72836073	6	19,788,415	G	A	0.015	0.018	0.180	0.832	0.137	0.635	1.089	0.849	Imputed
rs6918235	6	19,788,427	T	A	0.099	0.091	0.101	1.098	0.057	0.982	1.227	0.987	Imputed
chr6:19788949:D	6	19,788,949	TA	T	0.136	0.140	0.380	0.958	0.049	0.870	1.055	0.614	Imputed
rs9350185	6	19,789,173	A	G	0.134	0.116	8.91E-04	1.182	0.050	1.071	1.304	0.164	Imputed
rs2328434	6	19,789,374	T	A	0.446	0.432	0.096	1.059	0.034	0.990	1.133	0.395	Imputed
rs2328433	6	19,789,507	T	C	0.200	0.192	0.288	1.046	0.042	0.963	1.136	0.097	Imputed
rs55983166	6	19,789,676	T	G	0.010	0.010	0.786	0.954	0.173	0.681	1.338	0.964	Imputed
rs7747609	6	19,790,092	G	T	0.052	0.041	1.83E-03	1.274	0.078	1.094	1.484	0.468	Imputed
rs760796	6	19,790,387	T	C	0.468	0.429	3.34E-06	1.171	0.034	1.095	1.251	0.571	Imputed
rs760795	6	19,790,487	A	G	0.135	0.116	3.12E-04	1.198	0.050	1.086	1.321	0.173	Genotyped
rs760794	6	19,790,560	T	C	0.468	0.429	3.34E-06	1.171	0.034	1.095	1.251	0.571	Genotyped
rs17483277	6	19,790,571	A	G	0.334	0.313	0.011	1.096	0.036	1.021	1.176	0.143	Genotyped
chr6:19790659:I	6	19,790,659	AT	A	0.468	0.429	3.87E-06	1.170	0.034	1.094	1.250	0.582	Imputed
rs2223361	6	19,790,809	T	C	0.468	0.429	3.53E-06	1.170	0.034	1.095	1.251	0.575	Genotyped
rs2206035	6	19,790,979	T	A	0.199	0.192	0.283	1.047	0.042	0.963	1.137	0.115	Imputed
rs6456266	6	19,791,284	T	C	0.468	0.429	3.53E-06	1.170	0.034	1.095	1.251	0.575	Imputed
rs75871619	6	19,791,978	T	C	0.029	0.025	0.051	1.218	0.101	0.998	1.485	0.895	Imputed
rs1997690	6	19,792,254	C	T	0.259	0.256	0.789	1.010	0.039	0.937	1.090	0.085	Imputed
rs1997689	6	19,792,295	C	G	0.259	0.256	0.789	1.010	0.039	0.937	1.090	0.085	Imputed
rs12660828	6	19,792,498	A	G	0.084	0.098	4.82E-03	0.844	0.060	0.749	0.950	0.696	Imputed
rs1997688	6	19,792,518	C	A	0.259	0.256	0.796	1.010	0.039	0.936	1.089	0.086	Genotyped
rs1997687	6	19,792,526	A	G	0.252	0.250	0.864	1.007	0.039	0.933	1.087	0.069	Imputed
rs6456267	6	19,792,830	G	A	0.386	0.370	0.082	1.062	0.035	0.992	1.137	0.074	Imputed
rs11752817	6	19,793,162	T	C	0.251	0.249	0.865	1.007	0.039	0.933	1.087	0.073	Genotyped
rs62404176	6	19,793,524	T	G	0.084	0.099	4.55E-03	0.843	0.060	0.749	0.949	0.748	Imputed
rs62404177	6	19,793,566	C	G	0.084	0.099	4.98E-03	0.844	0.060	0.750	0.950	0.736	Imputed

rs9350186	6	19,793,575	A	G	0.133	0.115	6.75E-04	1.187	0.050	1.075	1.310	0.152	Imputed
rs76617443	6	19,793,634	G	A	0.037	0.041	0.256	0.904	0.089	0.758	1.077	0.678	Imputed
rs6921866	6	19,793,829	A	G	0.245	0.235	0.185	1.053	0.039	0.975	1.138	0.093	Genotyped
rs6921786	6	19,794,016	G	A	0.520	0.486	9.28E-05	1.141	0.034	1.068	1.220	0.377	Imputed
rs17549422	6	19,794,044	T	G	0.085	0.099	6.14E-03	0.848	0.060	0.754	0.954	0.686	Imputed
rs12525506	6	19,794,234	A	C	0.098	0.090	0.131	1.090	0.057	0.975	1.220	0.900	Imputed
rs58381549	6	19,794,374	G	C	0.085	0.099	6.14E-03	0.848	0.060	0.754	0.954	0.686	Imputed
rs9350187	6	19,794,487	T	C	0.081	0.074	0.069	1.120	0.062	0.991	1.266	0.239	Imputed
rs7759616	6	19,794,585	A	G	0.520	0.486	9.28E-05	1.141	0.034	1.068	1.220	0.377	Genotyped
rs75398766	6	19,795,015	C	T	0.051	0.041	2.27E-03	1.268	0.078	1.089	1.478	0.449	Imputed
rs75091868	6	19,795,274	A	G	0.014	0.017	0.094	0.786	0.144	0.593	1.042	0.821	Imputed
rs7764632	6	19,795,585	G	C	0.425	0.382	4.65E-07	1.189	0.034	1.111	1.271	0.712	Imputed
rs7769163	6	19,795,968	A	C	0.157	0.147	0.112	1.077	0.047	0.983	1.180	0.134	Genotyped
rs75356149	6	19,796,332	T	G	0.030	0.025	0.049	1.219	0.101	1.000	1.485	0.890	Imputed
chr6:19796580:I	6	19,796,580	CA	C	0.051	0.041	2.27E-03	1.268	0.078	1.089	1.478	0.449	Imputed
rs2206034	6	19,796,863	T	C	0.477	0.440	1.93E-05	1.156	0.034	1.081	1.235	0.490	Genotyped
chr6:19796876:I	6	19,796,876	AC	A	0.024	0.022	0.481	1.081	0.111	0.870	1.342	0.605	Imputed
rs2206033	6	19,796,878	C	A	0.522	0.488	7.80E-05	1.143	0.034	1.070	1.221	0.342	Imputed
rs9348397	6	19,797,320	T	C	0.134	0.116	9.50E-04	1.181	0.050	1.070	1.303	0.201	Imputed
rs9465527	6	19,797,394	A	G	0.245	0.234	0.156	1.057	0.039	0.979	1.142	0.083	Imputed
rs77944944	6	19,797,489	A	G	0.062	0.071	0.036	0.864	0.070	0.754	0.990	0.543	Imputed
rs6456268	6	19,797,654	T	A	0.343	0.324	0.025	1.083	0.036	1.010	1.162	0.088	Imputed
rs6456269	6	19,797,837	G	A	0.343	0.324	0.029	1.081	0.036	1.008	1.159	0.115	Genotyped
chr6:19797988:D	6	19,797,988	G	GA	0.478	0.440	1.61E-05	1.157	0.034	1.083	1.237	0.476	Imputed
chr6:19797992:D	6	19,797,992	A	AG	0.350	0.334	0.061	1.070	0.036	0.997	1.147	0.779	Imputed
rs9358271	6	19,798,075	T	A	0.134	0.116	9.50E-04	1.181	0.050	1.070	1.303	0.201	Imputed
rs6903595	6	19,798,141	T	C	0.425	0.383	5.63E-07	1.187	0.034	1.110	1.270	0.673	Genotyped
rs6926381	6	19,798,169	G	T	0.164	0.152	0.075	1.085	0.046	0.992	1.187	0.159	Genotyped
rs6926751	6	19,798,358	T	C	0.445	0.471	2.26E-03	0.901	0.034	0.843	0.964	0.395	Imputed
rs111509549	6	19,798,370	G	A	0.098	0.090	0.123	1.092	0.057	0.976	1.222	0.862	Imputed
chr6:19798443:D	6	19,798,443	A	AAAGG	0.134	0.116	9.50E-04	1.181	0.050	1.070	1.303	0.201	Imputed
rs6904600	6	19,798,493	A	G	0.164	0.152	0.074	1.085	0.046	0.992	1.187	0.158	Imputed
rs9465531	6	19,798,649	T	A	0.292	0.267	2.03E-03	1.122	0.037	1.043	1.207	0.176	Imputed
rs9368123	6	19,798,682	A	G	0.133	0.115	7.21E-04	1.186	0.050	1.074	1.309	0.149	Genotyped
rs6904518	6	19,798,704	G	A	0.425	0.382	2.73E-07	1.193	0.034	1.115	1.276	0.832	Genotyped
chr6:19798991:D	6	19,798,991	G	GCTAA	0.158	0.147	0.096	1.080	0.047	0.986	1.184	0.130	Imputed
chr6:19799169:D	6	19,799,169	A	AAAGAT	0.292	0.267	1.97E-03	1.122	0.037	1.043	1.208	0.174	Imputed
rs7747860	6	19,799,405	T	C	0.158	0.147	0.096	1.080	0.047	0.986	1.184	0.130	Imputed
rs2223360	6	19,799,441	C	A	0.231	0.205	1.58E-04	1.165	0.040	1.076	1.261	0.294	Genotyped
chr6:19799495:I	6	19,799,495	CAGAAAA	C	0.424	0.382	4.77E-07	1.188	0.034	1.111	1.271	0.812	Imputed

chr6:19799497:I	6	19,799,497	GAAAAAA	G	0.422	0.380	7.08E-07	1.185	0.034	1.108	1.268	0.850	Imputed
chr6:19799521:D	6	19,799,521	T	TG	0.426	0.383	3.75E-07	1.190	0.034	1.113	1.273	0.703	Imputed
rs79733895	6	19,799,541	G	A	0.098	0.090	0.123	1.092	0.057	0.976	1.222	0.862	Imputed
chr6:19800273:D	6	19,800,273	C	CA	0.292	0.267	2.03E-03	1.122	0.037	1.043	1.207	0.176	Imputed
rs6456270	6	19,800,491	G	C	0.343	0.324	0.026	1.083	0.036	1.009	1.161	0.108	Imputed
rs6921766	6	19,800,972	A	G	0.426	0.383	2.83E-07	1.192	0.034	1.115	1.275	0.736	Imputed
rs6921778	6	19,800,988	A	G	0.426	0.383	4.68E-07	1.188	0.034	1.111	1.271	0.720	Imputed
rs34388787	6	19,801,078	C	A	0.254	0.251	0.814	1.009	0.039	0.935	1.089	0.049	Imputed
rs76322282	6	19,801,660	A	G	0.098	0.089	0.105	1.097	0.057	0.981	1.228	0.877	Imputed
rs9465532	6	19,801,701	C	G	0.292	0.267	1.74E-03	1.124	0.037	1.045	1.209	0.181	Imputed
rs9465533	6	19,801,708	A	C	0.292	0.267	1.74E-03	1.124	0.037	1.045	1.209	0.181	Imputed
rs9465534	6	19,801,794	A	G	0.292	0.267	1.76E-03	1.124	0.037	1.044	1.209	0.181	Imputed
rs6456271	6	19,801,996	T	A	0.292	0.267	1.69E-03	1.124	0.037	1.045	1.210	0.186	Imputed
rs6938760	6	19,802,104	A	C	0.426	0.383	4.07E-07	1.190	0.034	1.112	1.272	0.737	Imputed
rs111231540	6	19,802,226	C	A	0.098	0.089	0.105	1.097	0.057	0.981	1.228	0.877	Imputed
rs6939160	6	19,802,317	G	C	0.292	0.267	1.57E-03	1.125	0.037	1.046	1.211	0.189	Imputed
chr6:19802495:D	6	19,802,495	C	CTG	0.277	0.255	3.97E-03	1.115	0.038	1.035	1.201	0.141	Imputed
rs73376633	6	19,802,678	T	A	0.029	0.025	0.108	1.177	0.102	0.964	1.437	0.696	Imputed
chr6:19802820:D	6	19,802,820	A	AATG	0.144	0.136	0.185	1.066	0.048	0.970	1.172	0.104	Imputed
rs16882710	6	19,803,155	T	C	0.052	0.041	1.74E-03	1.275	0.078	1.095	1.484	0.471	Genotyped
rs111533721	6	19,803,745	G	C	0.098	0.089	0.105	1.097	0.057	0.981	1.228	0.877	Imputed
rs6907340	6	19,803,768	T	C	0.413	0.372	1.29E-06	1.181	0.034	1.104	1.264	0.727	Genotyped
rs6907367	6	19,803,831	G	C	0.426	0.383	2.94E-07	1.192	0.034	1.115	1.275	0.753	Imputed
rs77486465	6	19,803,862	G	T	0.098	0.089	0.105	1.097	0.057	0.981	1.228	0.877	Imputed
rs78332113	6	19,804,007	C	G	0.029	0.024	0.061	1.210	0.102	0.991	1.477	0.920	Imputed
rs1155102	6	19,804,014	T	C	0.124	0.110	6.62E-03	1.151	0.052	1.040	1.274	0.142	Imputed
rs1155101	6	19,804,188	T	C	0.199	0.182	9.92E-03	1.116	0.043	1.027	1.213	0.601	Imputed
rs76709285	6	19,804,330	G	C	0.098	0.090	0.096	1.100	0.057	0.983	1.230	0.786	Imputed
rs114990132	6	19,804,359	C	A	0.052	0.042	2.43E-03	1.265	0.078	1.086	1.473	0.532	Imputed
rs1155100	6	19,804,365	T	C	0.458	0.421	9.16E-06	1.163	0.034	1.088	1.243	0.481	Imputed
chr6:19804369:I	6	19,804,369	TTACACTGC	T	0.115	0.103	0.025	1.127	0.053	1.015	1.251	0.628	Imputed
rs78895934	6	19,804,458	T	G	0.052	0.042	2.43E-03	1.265	0.078	1.086	1.473	0.532	Imputed
rs138618611	6	19,804,481	A	G	0.011	0.015	0.034	0.712	0.160	0.520	0.975	0.709	Imputed
rs79419305	6	19,804,510	C	T	0.099	0.090	0.091	1.101	0.057	0.985	1.232	0.857	Imputed
rs7746588	6	19,804,640	T	C	0.128	0.115	0.019	1.127	0.051	1.020	1.246	0.699	Imputed
rs112824001	6	19,804,691	T	C	0.098	0.090	0.094	1.100	0.057	0.984	1.231	0.783	Imputed
rs7750533	6	19,804,796	G	A	0.468	0.428	1.71E-06	1.176	0.034	1.100	1.257	0.546	Imputed
rs7750703	6	19,804,814	G	C	0.128	0.114	0.018	1.129	0.051	1.021	1.247	0.710	Imputed
rs7750716	6	19,804,839	T	C	0.128	0.115	0.019	1.127	0.051	1.020	1.246	0.699	Imputed
rs7750725	6	19,804,897	G	A	0.469	0.428	1.50E-06	1.177	0.034	1.101	1.258	0.536	Imputed

chr6:19805034:I	6	19,805,034	AG	A	0.021	0.016	0.011	1.362	0.121	1.074	1.727	0.065	Imputed
chr6:19805035:D	6	19,805,035	G	GC	0.162	0.141	6.98E-04	1.170	0.046	1.069	1.282	0.675	Imputed
rs116293473	6	19,805,125	A	G	0.050	0.040	3.40E-03	1.261	0.079	1.080	1.473	0.286	Imputed
rs139745567	6	19,805,216	T	G	0.094	0.085	0.067	1.112	0.058	0.992	1.247	0.481	Imputed
rs112465943	6	19,805,234	G	A	0.100	0.093	0.169	1.081	0.057	0.967	1.208	0.781	Imputed
chr6:19805361:D	6	19,805,361	T	TG	0.096	0.112	4.07E-03	0.850	0.057	0.760	0.950	0.872	Imputed
rs73376650	6	19,805,506	G	T	0.099	0.090	0.111	1.095	0.057	0.979	1.225	0.766	Imputed
rs79499891	6	19,805,516	T	G	0.028	0.024	0.098	1.186	0.103	0.969	1.452	0.949	Imputed
rs112599867	6	19,805,532	G	A	0.099	0.090	0.111	1.095	0.057	0.979	1.225	0.766	Imputed
rs961130	6	19,805,541	G	A	0.130	0.146	0.013	0.883	0.050	0.801	0.974	0.477	Imputed
rs73376651	6	19,805,559	G	C	0.099	0.090	0.111	1.095	0.057	0.979	1.225	0.766	Imputed
rs116566162	6	19,805,655	C	A	0.014	0.016	0.313	0.864	0.145	0.651	1.148	0.735	Imputed
rs73376655	6	19,805,664	T	C	0.099	0.090	0.111	1.095	0.057	0.979	1.225	0.766	Imputed
rs116315805	6	19,805,702	G	T	0.031	0.030	0.844	1.020	0.098	0.842	1.235	0.852	Imputed
rs9368125	6	19,805,876	A	G	0.073	0.069	0.317	1.068	0.066	0.939	1.214	0.279	Imputed
rs73376657	6	19,805,960	T	C	0.099	0.090	0.114	1.094	0.057	0.979	1.224	0.771	Imputed
rs74573060	6	19,806,032	A	T	0.097	0.089	0.127	1.092	0.058	0.975	1.222	0.925	Imputed
rs113592672	6	19,806,063	C	G	0.097	0.089	0.127	1.092	0.058	0.975	1.222	0.925	Imputed
rs113894708	6	19,806,066	T	G	0.097	0.089	0.127	1.092	0.058	0.975	1.222	0.925	Imputed
chr6:19806081:D	6	19,806,081	C	CT	0.097	0.089	0.127	1.092	0.058	0.975	1.222	0.925	Imputed
rs9358276	6	19,806,259	C	T	0.123	0.109	7.38E-03	1.150	0.052	1.038	1.273	0.206	Imputed
rs62404179	6	19,806,272	C	T	0.099	0.090	0.108	1.096	0.057	0.980	1.226	0.784	Imputed
rs10946342	6	19,806,298	A	G	0.123	0.109	7.12E-03	1.150	0.052	1.039	1.274	0.208	Imputed
rs73376662	6	19,806,340	A	G	0.099	0.090	0.114	1.094	0.057	0.979	1.224	0.771	Imputed
rs73376665	6	19,806,343	T	G	0.030	0.026	0.096	1.181	0.100	0.971	1.438	0.735	Imputed
rs113896292	6	19,806,466	A	G	0.099	0.090	0.116	1.094	0.057	0.978	1.223	0.775	Imputed
chr6:19806486:I	6	19,806,486	CTTCTGATG	C	0.192	0.187	0.477	1.031	0.044	0.947	1.123	0.892	Imputed
rs12529148	6	19,806,797	C	G	0.099	0.091	0.104	1.097	0.057	0.981	1.226	0.730	Imputed
rs12191540	6	19,806,810	C	T	0.121	0.108	9.75E-03	1.145	0.052	1.033	1.268	0.161	Imputed
rs12529121	6	19,806,823	T	C	0.099	0.091	0.095	1.100	0.057	0.984	1.229	0.713	Imputed
rs12529140	6	19,806,912	T	C	0.099	0.091	0.097	1.099	0.057	0.983	1.228	0.718	Imputed
rs35292732	6	19,806,946	T	G	0.069	0.061	0.064	1.133	0.067	0.993	1.293	0.767	Imputed
chr6:19806947:D	6	19,806,947	A	AT	0.069	0.061	0.064	1.133	0.067	0.993	1.293	0.767	Imputed
rs6906104	6	19,807,226	T	C	0.408	0.427	0.029	0.928	0.034	0.867	0.992	0.100	Imputed
rs114146042	6	19,807,229	A	G	0.099	0.090	0.108	1.096	0.057	0.980	1.225	0.682	Imputed
rs12197936	6	19,807,242	G	A	0.072	0.068	0.305	1.070	0.066	0.941	1.217	0.271	Imputed
rs6906292	6	19,807,296	C	T	0.496	0.485	0.202	1.044	0.034	0.977	1.116	0.183	Imputed
rs112659166	6	19,807,409	T	C	0.099	0.091	0.099	1.098	0.057	0.983	1.228	0.721	Imputed
rs6927315	6	19,807,683	A	G	0.202	0.196	0.462	1.031	0.042	0.950	1.120	0.054	Imputed
chr6:19807981:D	6	19,807,981	A	AAAGG	0.104	0.094	0.058	1.111	0.056	0.996	1.240	0.647	Imputed

chr6:19807992:D	6	19,807,992	G	GGAA	0.100	0.092	0.104	1.096	0.057	0.981	1.225	0.511	Imputed
rs34109983	6	19,808,042	A	G	0.099	0.091	0.100	1.098	0.057	0.982	1.227	0.723	Imputed
rs73376680	6	19,808,099	A	G	0.099	0.091	0.100	1.098	0.057	0.982	1.227	0.723	Imputed
rs73376681	6	19,808,175	G	A	0.099	0.091	0.100	1.098	0.057	0.982	1.227	0.723	Imputed
rs760793	6	19,808,270	G	A	0.143	0.156	0.040	0.906	0.048	0.824	0.996	0.421	Genotyped
rs80355837	6	19,808,343	G	C	0.099	0.091	0.100	1.098	0.057	0.982	1.227	0.723	Imputed
rs760792	6	19,808,347	C	T	0.132	0.148	0.011	0.882	0.050	0.800	0.972	0.260	Imputed
rs9368126	6	19,808,479	C	G	0.119	0.105	4.03E-03	1.164	0.053	1.050	1.291	0.175	Imputed
chr6:19808483:I	6	19,808,483	ATGAGT	A	0.093	0.086	0.137	1.091	0.058	0.973	1.223	0.827	Imputed
rs58242516	6	19,808,674	A	G	0.030	0.026	0.093	1.183	0.100	0.972	1.439	0.775	Imputed
rs76413455	6	19,808,964	G	T	0.099	0.090	0.097	1.099	0.057	0.983	1.229	0.767	Imputed
rs6938418	6	19,809,033	T	G	0.259	0.237	4.20E-03	1.117	0.039	1.035	1.205	0.149	Genotyped
rs76525593	6	19,809,170	T	G	0.099	0.090	0.097	1.099	0.057	0.983	1.229	0.767	Imputed
rs12525152	6	19,809,424	A	T	0.099	0.090	0.097	1.099	0.057	0.983	1.229	0.767	Imputed
chr6:19809493:D	6	19,809,493	T	TA	0.333	0.321	0.095	1.062	0.036	0.990	1.139	0.914	Imputed
chr6:19809500:D	6	19,809,500	A	AT	0.012	0.016	0.103	0.780	0.152	0.579	1.052	0.898	Imputed
rs11961118	6	19,809,777	G	C	0.030	0.026	0.091	1.184	0.100	0.973	1.441	0.770	Imputed
rs34254500	6	19,810,353	G	A	0.260	0.254	0.483	1.027	0.039	0.953	1.108	0.037	Imputed
rs74514942	6	19,810,520	A	G	0.099	0.090	0.098	1.099	0.057	0.983	1.228	0.770	Imputed
rs74561613	6	19,810,672	G	A	0.036	0.042	0.051	0.837	0.091	0.701	1.000	0.089	Imputed
rs16882727	6	19,810,832	T	C	0.030	0.026	0.109	1.174	0.100	0.965	1.429	0.715	Imputed
rs113071446	6	19,810,835	C	A	0.099	0.091	0.092	1.101	0.057	0.985	1.230	0.734	Imputed
rs6907446	6	19,811,074	T	C	0.195	0.187	0.225	1.053	0.043	0.969	1.145	0.044	Imputed
chr6:19811115:I	6	19,811,115	TTTATTA	T	0.084	0.075	0.048	1.129	0.061	1.001	1.273	0.956	Imputed
chr6:19811132:D	6	19,811,132	TA	T	0.267	0.269	0.712	0.986	0.038	0.915	1.063	0.861	Imputed
rs6456273	6	19,811,133	A	T	0.250	0.249	0.892	1.005	0.039	0.931	1.085	0.843	Imputed
chr6:19811135:I	6	19,811,135	TA	T	0.084	0.075	0.048	1.129	0.061	1.001	1.273	0.956	Imputed
rs9350190	6	19,811,660	G	A	0.207	0.208	0.964	1.002	0.042	0.923	1.087	0.469	Imputed
rs78402429	6	19,811,690	A	C	0.099	0.091	0.093	1.100	0.057	0.984	1.230	0.713	Imputed
rs16882731	6	19,811,887	T	C	0.030	0.026	0.119	1.169	0.101	0.960	1.424	0.730	Imputed
rs9368127	6	19,811,991	A	G	0.207	0.208	0.990	1.001	0.042	0.922	1.086	0.412	Genotyped
chr6:19812095:D	6	19,812,095	A	ATTT	0.100	0.091	0.101	1.098	0.057	0.982	1.227	0.739	Imputed
rs6925380	6	19,812,579	C	G	0.343	0.327	0.046	1.074	0.036	1.001	1.152	0.602	Imputed
rs77791009	6	19,812,602	G	A	0.099	0.090	0.121	1.092	0.057	0.977	1.222	0.687	Imputed
rs6924929	6	19,812,612	C	A	0.205	0.204	0.846	1.008	0.042	0.929	1.095	0.492	Imputed
rs66668421	6	19,812,659	T	C	0.207	0.195	0.096	1.072	0.042	0.988	1.164	0.050	Imputed
chr6:19812731:D	6	19,812,731	T	TATG	0.014	0.012	0.318	1.156	0.146	0.869	1.539	0.523	Imputed
chr6:19812732:D	6	19,812,732	A	ATGAT	0.030	0.027	0.201	1.136	0.100	0.934	1.382	0.671	Imputed
rs75060331	6	19,812,956	T	C	0.099	0.090	0.121	1.092	0.057	0.977	1.222	0.687	Imputed
rs77753186	6	19,812,980	C	G	0.099	0.090	0.121	1.092	0.057	0.977	1.222	0.687	Imputed

rs75359632	6	19,813,030	G	T	0.029	0.024	0.062	1.208	0.102	0.990	1.475	0.926	Imputed
rs79162793	6	19,813,065	G	A	0.099	0.090	0.121	1.092	0.057	0.977	1.222	0.687	Imputed
rs60290480	6	19,813,120	A	C	0.163	0.149	0.032	1.103	0.046	1.008	1.208	0.098	Imputed
rs76245225	6	19,813,188	G	T	0.099	0.090	0.121	1.092	0.057	0.977	1.222	0.687	Imputed
rs725316	6	19,813,619	A	G	0.030	0.026	0.101	1.178	0.100	0.968	1.434	0.701	Imputed
rs9348398	6	19,814,369	C	G	0.397	0.420	6.29E-03	0.910	0.035	0.850	0.974	0.179	Imputed
rs75110577	6	19,814,523	T	C	0.099	0.090	0.109	1.095	0.057	0.980	1.225	0.666	Imputed
chr6:19814567:D	6	19,814,567	T	TC	0.099	0.090	0.109	1.095	0.057	0.980	1.225	0.666	Imputed
rs77386860	6	19,814,755	C	A	0.099	0.091	0.115	1.094	0.057	0.978	1.223	0.677	Imputed
rs77933553	6	19,814,969	T	C	0.042	0.045	0.467	0.941	0.084	0.797	1.109	0.579	Imputed
rs9465536	6	19,816,177	A	G	0.216	0.207	0.254	1.048	0.041	0.967	1.136	0.056	Imputed
rs79406203	6	19,816,221	G	T	0.099	0.090	0.111	1.095	0.057	0.979	1.224	0.669	Imputed
rs7744395	6	19,816,475	T	C	0.099	0.090	0.111	1.095	0.057	0.979	1.224	0.669	Imputed
rs6456274	6	19,816,514	C	T	0.099	0.091	0.120	1.093	0.057	0.977	1.222	0.685	Imputed
rs111825079	6	19,816,524	T	C	0.030	0.026	0.091	1.184	0.100	0.973	1.441	0.725	Imputed
rs9358277	6	19,816,629	A	G	0.242	0.243	0.985	0.999	0.040	0.925	1.080	0.698	Imputed
rs9460417	6	19,816,857	T	C	0.164	0.150	0.033	1.103	0.046	1.008	1.207	0.073	Imputed
rs111514271	6	19,817,429	A	G	0.140	0.134	0.281	1.054	0.049	0.958	1.160	0.719	Imputed
rs113458950	6	19,817,550	C	T	0.099	0.090	0.097	1.099	0.057	0.983	1.229	0.644	Imputed
rs6917179	6	19,817,801	G	C	0.248	0.250	0.935	0.997	0.039	0.923	1.076	0.769	Imputed
rs4140625	6	19,818,340	C	T	0.248	0.250	0.922	0.996	0.039	0.923	1.076	0.762	Imputed
rs4140624	6	19,818,560	T	C	0.204	0.203	0.815	1.010	0.042	0.930	1.097	0.580	Imputed
rs4712433	6	19,818,707	G	A	0.285	0.305	0.012	0.910	0.037	0.846	0.980	0.271	Genotyped
rs74559916	6	19,818,717	T	G	0.099	0.090	0.111	1.095	0.057	0.979	1.224	0.669	Imputed
rs76391302	6	19,819,116	A	G	0.099	0.090	0.111	1.095	0.057	0.979	1.224	0.669	Imputed
rs77880623	6	19,819,454	C	T	0.099	0.090	0.111	1.095	0.057	0.979	1.224	0.669	Imputed
rs12197664	6	19,819,649	A	G	0.203	0.202	0.826	1.009	0.042	0.929	1.096	0.583	Imputed
rs111565547	6	19,819,666	G	A	0.099	0.090	0.111	1.095	0.057	0.979	1.224	0.669	Imputed
rs111529353	6	19,819,793	G	A	0.030	0.026	0.147	1.157	0.101	0.950	1.408	0.773	Imputed
rs112606701	6	19,819,827	T	C	0.099	0.090	0.111	1.095	0.057	0.979	1.224	0.669	Imputed
rs112388826	6	19,819,858	G	A	0.099	0.090	0.111	1.095	0.057	0.979	1.224	0.669	Imputed
rs12206194	6	19,819,908	C	T	0.204	0.204	0.909	1.005	0.042	0.925	1.091	0.514	Imputed
rs73378506	6	19,819,987	C	T	0.129	0.116	0.025	1.121	0.051	1.014	1.238	0.577	Imputed
rs12526109	6	19,820,089	A	C	0.099	0.090	0.111	1.095	0.057	0.979	1.224	0.669	Imputed
rs6910773	6	19,820,658	C	T	0.256	0.261	0.540	0.977	0.039	0.905	1.054	0.769	Genotyped
rs78774173	6	19,820,686	C	T	0.099	0.090	0.111	1.095	0.057	0.979	1.224	0.669	Imputed
rs116427286	6	19,821,131	T	A	0.012	0.014	0.436	0.888	0.153	0.657	1.199	0.858	Imputed
rs7771384	6	19,821,270	A	G	0.246	0.228	0.012	1.104	0.039	1.022	1.192	0.077	Genotyped
rs79842087	6	19,821,316	A	G	0.099	0.091	0.112	1.095	0.057	0.979	1.224	0.672	Imputed
rs12527834	6	19,821,781	G	A	0.099	0.091	0.112	1.095	0.057	0.979	1.224	0.672	Imputed

rs12527843	6	19,821,861	G	A	0.099	0.091	0.121	1.092	0.057	0.977	1.221	0.687	Imputed
rs7747295	6	19,821,910	G	C	0.094	0.087	0.209	1.076	0.058	0.960	1.206	0.610	Imputed
chr6:19822357:D	6	19,822,357	T	TAAAG	0.099	0.090	0.107	1.096	0.057	0.980	1.226	0.632	Imputed
rs7773456	6	19,823,238	G	T	0.313	0.317	0.693	0.986	0.036	0.918	1.059	0.831	Genotyped
rs7753263	6	19,823,252	C	T	0.397	0.419	8.32E-03	0.913	0.035	0.853	0.977	0.112	Genotyped
rs7773816	6	19,823,432	A	T	0.360	0.330	2.77E-04	1.137	0.035	1.061	1.218	0.162	Imputed
rs9465539	6	19,823,890	A	G	0.187	0.194	0.295	0.956	0.043	0.878	1.040	0.740	Imputed
rs7740073	6	19,823,943	C	T	0.233	0.242	0.247	0.955	0.040	0.883	1.033	0.477	Imputed
rs6921758	6	19,824,025	T	C	0.362	0.334	5.79E-04	1.129	0.035	1.054	1.210	0.184	Imputed
rs6456275	6	19,824,186	A	G	0.498	0.487	0.194	1.045	0.034	0.978	1.117	0.119	Imputed
rs6456276	6	19,824,198	G	T	0.507	0.494	0.105	1.056	0.034	0.989	1.129	0.197	Imputed
rs9358278	6	19,824,971	A	C	0.228	0.211	0.017	1.102	0.040	1.018	1.192	0.339	Imputed
rs6908376	6	19,825,305	C	T	0.506	0.492	0.106	1.056	0.034	0.989	1.129	0.188	Imputed
rs78920271	6	19,825,523	A	G	0.015	0.015	0.933	0.988	0.141	0.750	1.303	0.898	Imputed
rs78408834	6	19,825,913	C	T	0.027	0.023	0.154	1.162	0.106	0.945	1.430	0.717	Imputed
rs111587319	6	19,825,962	G	A	0.017	0.018	0.567	0.928	0.132	0.717	1.200	0.152	Imputed
rs7774679	6	19,827,152	C	A	0.061	0.063	0.695	0.973	0.071	0.846	1.118	0.029	Imputed
rs7756998	6	19,827,278	C	T	0.110	0.108	0.553	1.033	0.054	0.929	1.149	0.095	Imputed
rs6456277	6	19,827,389	C	G	0.374	0.393	0.034	0.928	0.035	0.866	0.994	0.343	Imputed
rs9368128	6	19,827,421	C	T	0.028	0.031	0.453	0.927	0.102	0.759	1.131	0.596	Imputed
rs9348399	6	19,827,905	T	C	0.148	0.151	0.646	0.978	0.048	0.891	1.074	0.458	Genotyped
rs971144	6	19,827,919	G	A	0.380	0.383	0.728	0.988	0.035	0.923	1.058	0.367	Genotyped
rs6926229	6	19,828,213	T	C	0.470	0.457	0.120	1.054	0.034	0.986	1.127	0.859	Genotyped
chr6:19828597:D	6	19,828,597	T	TG	0.281	0.284	0.697	0.985	0.038	0.915	1.061	0.538	Imputed
rs141585469	6	19,829,164	C	G	0.018	0.022	0.177	0.843	0.126	0.659	1.079	0.298	Imputed
rs66837488	6	19,830,310	C	T	0.279	0.275	0.598	1.020	0.038	0.947	1.098	0.815	Imputed
rs7757599	6	19,830,846	C	T	0.317	0.302	0.069	1.068	0.036	0.995	1.147	0.996	Imputed
rs7757706	6	19,830,882	G	C	0.276	0.279	0.732	0.987	0.038	0.917	1.063	0.460	Imputed
rs35293914	6	19,831,177	A	G	0.162	0.162	0.977	0.999	0.046	0.913	1.093	0.566	Imputed
rs6456278	6	19,831,215	A	T	0.470	0.478	0.335	0.968	0.034	0.906	1.034	0.861	Imputed
rs72836083	6	19,831,263	A	T	0.102	0.102	0.937	0.996	0.056	0.892	1.111	0.927	Imputed
rs3777741	6	19,831,435	G	C	0.034	0.037	0.449	0.932	0.093	0.776	1.119	0.926	Imputed
rs6922714	6	19,831,560	A	G	0.316	0.321	0.568	0.980	0.036	0.912	1.052	0.417	Genotyped
rs17550540	6	19,831,598	C	T	0.034	0.037	0.443	0.931	0.093	0.776	1.118	0.930	Imputed
rs12528525	6	19,831,818	G	T	0.284	0.287	0.742	0.988	0.038	0.918	1.063	0.313	Imputed
rs60752518	6	19,832,130	A	G	0.278	0.283	0.571	0.979	0.038	0.909	1.054	0.311	Imputed
chr6:19832382:I	6	19,832,382	GC	G	0.033	0.037	0.359	0.917	0.094	0.763	1.103	0.994	Imputed
rs7773538	6	19,832,384	G	C	0.044	0.036	0.012	1.233	0.084	1.046	1.454	0.442	Imputed
rs76175662	6	19,832,445	G	T	0.278	0.282	0.634	0.982	0.038	0.912	1.058	0.237	Imputed
rs13218929	6	19,832,460	T	G	0.473	0.482	0.310	0.966	0.034	0.904	1.033	0.598	Imputed

rs6902000	6	19,833,413	G	A	0.475	0.484	0.287	0.965	0.034	0.903	1.031	0.696	Imputed
rs6456279	6	19,834,560	G	T	0.470	0.459	0.222	1.042	0.034	0.975	1.114	0.517	Imputed
rs1980461	6	19,835,009	C	G	0.471	0.480	0.297	0.965	0.034	0.903	1.032	0.808	Imputed
rs11967589	6	19,835,389	A	G	0.310	0.311	0.945	0.998	0.037	0.928	1.072	0.864	Imputed
rs11962599	6	19,835,446	G	A	0.033	0.036	0.299	0.907	0.095	0.753	1.091	0.954	Imputed
rs55907705	6	19,835,915	C	A	0.015	0.016	0.496	0.910	0.139	0.694	1.194	0.352	Imputed
rs6919858	6	19,836,015	A	G	0.472	0.480	0.343	0.968	0.034	0.906	1.035	0.856	Imputed
rs34836545	6	19,836,370	G	A	0.033	0.036	0.317	0.910	0.095	0.756	1.095	0.932	Imputed
chr6:19836798:I	6	19,836,798	AC	A	0.012	0.011	0.381	1.147	0.157	0.843	1.560	0.517	Imputed
chr6:19836868:I	6	19,836,868	C	CCTT	0.245	0.239	0.497	1.027	0.039	0.951	1.110	0.917	Imputed
rs140754978	6	19,837,062	A	G	0.029	0.034	0.097	0.848	0.099	0.698	1.030	0.503	Imputed
rs13210018	6	19,837,457	C	G	0.271	0.268	0.715	1.014	0.038	0.941	1.093	0.787	Imputed
rs149625702	6	19,837,524	T	G	0.072	0.072	0.930	0.994	0.066	0.874	1.131	0.608	Imputed
rs926368	6	19,837,548	C	T	0.363	0.365	0.735	0.988	0.035	0.922	1.059	0.327	Imputed
rs76835657	6	19,837,557	G	C	0.066	0.064	0.734	1.024	0.069	0.895	1.171	0.381	Imputed
chr6:19837754:D	6	19,837,754	C	CT	0.344	0.346	0.790	0.991	0.036	0.924	1.062	0.219	Imputed
rs11759102	6	19,837,774	T	C	0.128	0.135	0.281	0.947	0.050	0.858	1.046	0.197	Imputed
rs192039062	6	19,837,964	T	C	0.014	0.014	0.940	0.989	0.143	0.748	1.308	0.339	Imputed
rs1047316	6	19,837,966	C	T	0.274	0.276	0.677	0.984	0.038	0.913	1.061	0.979	Imputed
rs12662234	6	19,837,969	G	A	0.271	0.273	0.647	0.983	0.038	0.912	1.059	0.940	Imputed
rs147448400	6	19,838,447	A	C	0.046	0.045	0.779	1.023	0.081	0.873	1.199	0.343	Imputed
rs113135736	6	19,838,516	T	C	0.229	0.229	0.895	0.995	0.040	0.919	1.076	0.820	Imputed
rs77115435	6	19,838,957	A	T	0.015	0.017	0.216	0.842	0.139	0.641	1.107	0.411	Imputed
rs9460423	6	19,838,990	G	C	0.056	0.057	0.762	0.978	0.074	0.847	1.130	0.675	Imputed
rs41271299	6	19,839,415	T	C	0.037	0.035	0.424	1.075	0.090	0.901	1.281	0.447	Imputed
rs8214	6	19,839,779	T	C	0.224	0.228	0.578	0.978	0.041	0.903	1.059	0.141	Imputed
chr6:19839847:D	6	19,839,847	A	AT	0.203	0.210	0.370	0.963	0.042	0.887	1.046	0.604	Imputed
chr6:19840060:D	6	19,840,060	A	ATG	0.067	0.067	0.776	1.020	0.068	0.892	1.165	0.023	Imputed
rs12663067	6	19,840,120	T	A	0.063	0.066	0.367	0.939	0.070	0.819	1.076	0.734	Imputed
rs9465545	6	19,840,342	G	A	0.259	0.260	0.795	0.990	0.039	0.918	1.068	0.381	Imputed
rs1047033	6	19,840,492	G	A	0.319	0.316	0.700	1.014	0.036	0.944	1.089	0.883	Genotyped
chr6:19840952:D	6	19,840,952	C	CTT	0.261	0.262	0.859	0.993	0.039	0.921	1.071	0.544	Imputed
rs3798339	6	19,840,995	G	A	0.063	0.065	0.558	0.960	0.070	0.838	1.100	0.829	Imputed
rs7765893	6	19,841,388	C	T	0.362	0.351	0.174	1.049	0.035	0.979	1.124	0.520	Imputed
rs1047014	6	19,841,493	C	T	0.254	0.255	0.861	0.993	0.039	0.920	1.072	0.397	Imputed
rs11545617	6	19,841,966	T	C	0.180	0.186	0.375	0.962	0.044	0.882	1.048	0.443	Imputed
rs6917605	6	19,842,497	T	C	0.456	0.463	0.460	0.975	0.034	0.912	1.042	0.899	Genotyped
rs12526762	6	19,842,556	A	G	0.262	0.261	0.884	1.006	0.039	0.933	1.085	0.383	Imputed
rs72836088	6	19,842,560	A	T	0.034	0.037	0.528	0.943	0.092	0.787	1.131	0.830	Imputed
rs9350191	6	19,842,661	C	T	0.126	0.129	0.663	0.978	0.051	0.885	1.081	0.298	Imputed

rs72836089	6	19,842,959	G	A	0.021	0.023	0.468	0.919	0.117	0.730	1.156	0.462	Imputed
rs13191574	6	19,843,400	G	A	0.015	0.014	0.483	1.103	0.140	0.838	1.451	0.318	Imputed
rs6905736	6	19,843,767	C	A	0.120	0.126	0.330	0.951	0.052	0.859	1.053	0.254	Imputed
chr6:19844116:D	6	19,844,116	C	CA	0.029	0.030	0.515	0.936	0.102	0.767	1.142	0.242	Imputed
chr6:19844125:D	6	19,844,125	T	TG	0.048	0.049	0.722	0.972	0.079	0.833	1.135	0.767	Imputed
rs6906699	6	19,844,355	G	A	0.239	0.236	0.727	1.014	0.040	0.938	1.096	0.464	Genotyped
rs142599545	6	19,844,384	G	A	0.014	0.012	0.156	1.226	0.144	0.925	1.626	0.698	Imputed
rs62404182	6	19,844,533	G	C	0.242	0.238	0.563	1.023	0.040	0.947	1.106	0.551	Imputed
rs7765485	6	19,844,713	A	T	0.299	0.292	0.374	1.034	0.037	0.961	1.112	0.803	Imputed
rs77102978	6	19,844,743	G	T	0.055	0.061	0.144	0.898	0.074	0.776	1.038	0.930	Imputed
rs6456280	6	19,845,014	A	G	0.324	0.328	0.648	0.984	0.036	0.916	1.056	0.969	Imputed
chr6:19845133:D	6	19,845,133	G	GTC	0.052	0.055	0.302	0.925	0.076	0.796	1.073	0.909	Imputed
rs1009501	6	19,845,305	G	C	0.021	0.025	0.137	0.842	0.116	0.670	1.057	0.897	Imputed
rs139040822	6	19,845,569	G	C	0.011	0.011	0.765	0.952	0.163	0.691	1.312	0.290	Imputed
rs75933196	6	19,845,741	G	A	0.035	0.034	0.717	1.034	0.092	0.864	1.238	0.349	Imputed
rs9465547	6	19,845,911	C	G	0.198	0.191	0.269	1.048	0.043	0.964	1.140	0.717	Imputed
rs115769260	6	19,845,934	A	G	0.021	0.020	0.549	1.073	0.118	0.852	1.353	0.902	Imputed
rs145360871	6	19,845,980	T	G	0.009	0.012	0.119	0.764	0.173	0.544	1.072	0.646	Imputed
rs9368129	6	19,846,079	A	G	0.081	0.079	0.853	1.012	0.062	0.895	1.143	0.259	Imputed
rs9350192	6	19,846,336	G	A	0.073	0.071	0.711	1.025	0.065	0.901	1.165	0.280	Imputed
rs35003014	6	19,846,389	G	A	0.142	0.141	0.828	1.011	0.049	0.919	1.112	0.065	Imputed
rs150460023	6	19,847,177	T	C	0.011	0.011	0.834	0.966	0.164	0.701	1.331	0.125	Imputed
rs185531555	6	19,847,719	A	G	0.027	0.031	0.089	0.837	0.105	0.682	1.027	0.218	Imputed
rs11754400	6	19,848,545	G	A	0.105	0.102	0.516	1.037	0.055	0.930	1.155	0.758	Imputed
rs7749020	6	19,848,673	C	T	0.220	0.221	0.823	0.991	0.041	0.915	1.074	0.083	Genotyped
rs12195033	6	19,849,242	A	T	0.029	0.030	0.709	0.963	0.102	0.789	1.175	0.089	Imputed
rs6937263	6	19,849,403	T	C	0.028	0.029	0.770	0.971	0.102	0.795	1.185	0.068	Imputed
rs62404193	6	19,849,653	C	T	0.192	0.192	0.980	0.999	0.043	0.918	1.087	0.229	Imputed
rs13191070	6	19,850,036	A	G	0.106	0.104	0.683	1.023	0.055	0.918	1.139	0.965	Imputed
rs112175294	6	19,850,181	G	T	0.015	0.013	0.698	1.056	0.141	0.802	1.391	0.559	Imputed
rs9350193	6	19,850,245	T	C	0.176	0.174	0.782	1.012	0.044	0.928	1.105	0.738	Imputed
rs12190051	6	19,850,707	T	C	0.029	0.030	0.781	0.972	0.101	0.798	1.184	0.112	Genotyped
rs79392399	6	19,850,718	T	A	0.106	0.103	0.433	1.044	0.055	0.937	1.163	0.876	Imputed
rs10456226	6	19,850,819	G	A	0.151	0.158	0.289	0.951	0.047	0.867	1.043	0.957	Imputed
rs6905466	6	19,850,875	T	C	0.324	0.323	0.917	1.004	0.036	0.935	1.078	0.180	Genotyped
rs1010284	6	19,851,608	T	C	0.029	0.030	0.695	0.961	0.102	0.788	1.172	0.097	Imputed
rs11961818	6	19,851,729	A	G	0.190	0.189	0.987	1.001	0.043	0.920	1.089	0.236	Imputed
rs9366319	6	19,852,106	G	C	0.028	0.029	0.771	0.971	0.102	0.795	1.185	0.068	Imputed
rs79709578	6	19,852,772	T	C	0.043	0.044	0.813	0.981	0.083	0.833	1.154	0.546	Imputed
rs6908308	6	19,852,941	G	T	0.190	0.189	0.929	1.004	0.043	0.923	1.092	0.221	Imputed

rs9356714	6	19,853,320	G	A	0.218	0.216	0.745	1.013	0.041	0.935	1.098	0.298	Imputed
rs1474612	6	19,853,484	A	G	0.181	0.176	0.378	1.040	0.044	0.954	1.133	0.627	Imputed
rs1474611	6	19,853,562	T	A	0.219	0.218	0.972	0.999	0.041	0.922	1.082	0.080	Imputed
rs3950182	6	19,854,105	T	G	0.181	0.176	0.388	1.039	0.044	0.953	1.132	0.618	Imputed
rs2142902	6	19,854,292	G	C	0.029	0.030	0.638	0.953	0.101	0.782	1.162	0.089	Imputed
rs2179272	6	19,854,551	G	C	0.221	0.214	0.272	1.046	0.041	0.965	1.133	0.495	Imputed
rs4504464	6	19,854,651	T	C	0.189	0.188	0.898	1.006	0.043	0.924	1.094	0.266	Imputed
rs13216637	6	19,854,713	A	G	0.106	0.102	0.462	1.041	0.055	0.935	1.160	0.919	Imputed
rs4276485	6	19,854,898	A	G	0.192	0.191	0.950	1.003	0.043	0.922	1.091	0.184	Imputed
rs146534140	6	19,854,920	T	C	0.029	0.030	0.734	0.966	0.101	0.793	1.177	0.063	Imputed
rs181217173	6	19,854,944	A	G	0.016	0.016	0.604	0.932	0.136	0.714	1.217	0.787	Imputed
chr6:19854958:I	6	19,854,958	GC	G	0.273	0.269	0.741	1.013	0.038	0.940	1.091	0.347	Imputed
rs185461650	6	19,855,008	A	G	0.009	0.010	0.441	0.873	0.176	0.618	1.234	0.698	Imputed
rs4431418	6	19,855,017	C	T	0.221	0.220	0.974	1.001	0.041	0.924	1.085	0.060	Imputed
rs760801	6	19,855,165	A	G	0.191	0.187	0.489	1.030	0.043	0.947	1.121	0.713	Imputed
rs13220066	6	19,855,290	G	A	0.106	0.103	0.521	1.036	0.055	0.930	1.154	0.925	Imputed
rs742229	6	19,855,461	G	A	0.191	0.189	0.898	1.006	0.043	0.924	1.094	0.174	Imputed
rs2328460	6	19,855,613	C	T	0.107	0.103	0.430	1.044	0.055	0.938	1.163	0.980	Imputed
rs2294430	6	19,855,921	T	C	0.029	0.030	0.726	0.965	0.102	0.791	1.177	0.081	Imputed
rs742228	6	19,855,933	A	G	0.220	0.220	0.986	1.001	0.041	0.924	1.084	0.056	Genotyped
rs742227	6	19,856,058	G	A	0.164	0.163	0.913	1.005	0.046	0.919	1.099	0.914	Genotyped
rs9460424	6	19,856,701	G	T	0.191	0.189	0.823	1.010	0.043	0.928	1.099	0.183	Imputed
rs74323750	6	19,856,836	T	C	0.032	0.033	0.948	0.994	0.096	0.823	1.200	0.410	Imputed
rs141168133	6	19,856,909	G	A	0.012	0.010	0.193	1.222	0.154	0.903	1.653	0.510	Imputed
chr6:19856948:D	6	19,856,948	T	TA	0.328	0.323	0.585	1.020	0.036	0.950	1.095	0.100	Imputed
rs13202453	6	19,858,176	G	A	0.111	0.107	0.408	1.046	0.054	0.941	1.162	0.886	Imputed
rs13203119	6	19,858,468	A	G	0.112	0.108	0.420	1.044	0.054	0.940	1.161	0.991	Genotyped
rs6939816	6	19,858,516	C	T	0.027	0.028	0.911	0.988	0.104	0.806	1.211	0.089	Imputed
chr6:19858603:I	6	19,858,603	TG	T	0.018	0.022	0.098	0.813	0.125	0.637	1.039	0.574	Imputed
rs116803082	6	19,859,255	A	G	0.012	0.011	0.530	1.102	0.155	0.813	1.493	0.407	Imputed
rs4710918	6	19,859,339	A	G	0.336	0.338	0.862	0.994	0.036	0.927	1.066	0.570	Imputed
rs6923535	6	19,859,654	A	G	0.448	0.446	0.832	1.007	0.034	0.942	1.077	0.241	Imputed
rs9366320	6	19,859,897	A	C	0.335	0.337	0.869	0.994	0.036	0.927	1.066	0.546	Genotyped
rs4618518	6	19,860,298	T	C	0.209	0.210	0.883	0.994	0.042	0.916	1.078	0.378	Genotyped
rs932346	6	19,860,447	T	C	0.183	0.178	0.415	1.036	0.044	0.951	1.129	0.518	Genotyped
rs7769138	6	19,860,788	G	C	0.197	0.190	0.223	1.053	0.043	0.969	1.145	0.412	Imputed
rs4712434	6	19,861,776	G	A	0.199	0.192	0.231	1.052	0.042	0.968	1.143	0.461	Imputed
rs146118920	6	19,861,886	C	G	0.010	0.012	0.143	0.779	0.171	0.557	1.089	0.750	Imputed
rs2056953	6	19,862,203	C	T	0.027	0.028	0.736	0.965	0.104	0.787	1.185	0.170	Imputed
rs57661677	6	19,862,244	G	A	0.377	0.379	0.956	0.998	0.035	0.932	1.069	0.749	Imputed

rs71560105	6	19,862,891	G	A	0.152	0.161	0.172	0.938	0.047	0.855	1.028	0.858	Imputed
rs4710919	6	19,863,150	A	T	0.361	0.364	0.800	0.991	0.035	0.925	1.062	0.910	Imputed
rs9356715	6	19,863,232	T	C	0.025	0.026	0.696	0.958	0.110	0.773	1.187	0.113	Imputed
rs1011119	6	19,864,165	G	T	0.350	0.350	0.881	1.005	0.035	0.938	1.078	0.360	Imputed
rs6926989	6	19,865,407	A	C	0.026	0.026	0.869	0.983	0.107	0.797	1.212	0.161	Imputed
rs13210561	6	19,865,689	T	C	0.152	0.160	0.245	0.947	0.047	0.864	1.038	0.811	Imputed
rs73731213	6	19,865,785	A	T	0.026	0.026	0.905	0.987	0.107	0.800	1.218	0.151	Imputed
rs74370952	6	19,866,264	G	T	0.045	0.046	0.645	0.963	0.081	0.821	1.130	0.313	Imputed
rs975673	6	19,866,400	C	G	0.336	0.339	0.842	0.993	0.036	0.926	1.065	0.542	Imputed
rs77694980	6	19,867,244	C	A	0.013	0.014	0.751	0.954	0.148	0.714	1.275	0.610	Imputed
rs11755276	6	19,867,441	T	C	0.014	0.012	0.228	1.192	0.147	0.894	1.590	0.232	Imputed
rs760800	6	19,868,095	A	G	0.187	0.180	0.253	1.051	0.043	0.965	1.144	0.464	Imputed
rs9465548	6	19,868,404	A	T	0.339	0.346	0.321	0.965	0.036	0.900	1.035	0.166	Imputed
rs6902539	6	19,868,502	T	C	0.215	0.218	0.602	0.979	0.041	0.903	1.061	0.053	Genotyped
rs6903044	6	19,868,603	C	G	0.213	0.217	0.512	0.973	0.041	0.898	1.055	0.084	Imputed
rs4710920	6	19,868,768	C	T	0.151	0.159	0.228	0.945	0.047	0.862	1.036	0.808	Imputed
rs62404196	6	19,869,252	T	C	0.197	0.202	0.337	0.960	0.043	0.883	1.043	0.057	Imputed
rs34407656	6	19,869,396	A	G	0.152	0.160	0.261	0.949	0.047	0.865	1.040	0.773	Imputed
rs6913702	6	19,870,068	G	A	0.349	0.362	0.096	0.943	0.035	0.879	1.011	0.069	Imputed
rs73731214	6	19,870,136	T	C	0.026	0.026	0.885	0.985	0.108	0.797	1.216	0.131	Imputed
rs10484639	6	19,870,333	A	G	0.084	0.084	0.960	1.003	0.061	0.890	1.130	0.434	Genotyped
rs6914725	6	19,870,406	A	G	0.348	0.361	0.112	0.945	0.035	0.882	1.013	0.072	Imputed
rs9460426	6	19,870,686	C	T	0.349	0.362	0.096	0.943	0.035	0.879	1.011	0.069	Imputed
rs9465549	6	19,871,076	G	A	0.349	0.362	0.104	0.944	0.035	0.881	1.012	0.054	Genotyped
rs116677711	6	19,871,317	T	A	0.026	0.026	0.894	0.986	0.108	0.798	1.217	0.132	Imputed
rs71560106	6	19,871,393	T	C	0.111	0.107	0.457	1.041	0.054	0.937	1.157	0.830	Imputed
rs62404197	6	19,872,020	A	C	0.170	0.175	0.319	0.956	0.045	0.875	1.044	0.192	Imputed
rs35272702	6	19,872,156	C	A	0.349	0.362	0.096	0.943	0.035	0.879	1.011	0.062	Imputed
rs57861446	6	19,872,344	T	C	0.025	0.025	0.738	0.964	0.110	0.778	1.195	0.120	Imputed
rs12201493	6	19,872,356	T	C	0.349	0.362	0.096	0.943	0.035	0.879	1.011	0.062	Imputed
rs12201508	6	19,872,428	T	C	0.349	0.362	0.098	0.943	0.035	0.880	1.011	0.070	Imputed
rs12201511	6	19,872,440	A	C	0.348	0.360	0.122	0.947	0.036	0.883	1.015	0.047	Imputed
rs145392848	6	19,872,502	C	T	0.015	0.012	0.239	1.181	0.142	0.894	1.560	0.187	Imputed
rs12201697	6	19,872,557	A	G	0.349	0.362	0.096	0.943	0.035	0.879	1.011	0.062	Imputed
rs62404199	6	19,872,850	T	C	0.196	0.202	0.293	0.956	0.043	0.880	1.039	0.054	Imputed
rs4712435	6	19,872,952	C	T	0.349	0.362	0.099	0.943	0.035	0.880	1.011	0.059	Imputed
rs2142901	6	19,873,374	A	G	0.111	0.107	0.472	1.040	0.054	0.935	1.156	0.863	Imputed
rs1534923	6	19,873,584	T	C	0.196	0.202	0.297	0.957	0.043	0.880	1.040	0.054	Genotyped
rs62404200	6	19,873,870	C	T	0.168	0.174	0.283	0.953	0.045	0.872	1.041	0.210	Imputed
rs1534922	6	19,873,878	C	T	0.348	0.362	0.091	0.942	0.035	0.879	1.010	0.063	Imputed

rs1534921	6	19,873,939	A	G	0.348	0.362	0.091	0.942	0.035	0.879	1.010	0.063	Imputed
rs6906457	6	19,874,109	C	G	0.348	0.362	0.091	0.942	0.035	0.879	1.010	0.063	Imputed
rs7745135	6	19,874,475	C	G	0.348	0.362	0.091	0.942	0.035	0.879	1.010	0.063	Imputed
rs7745072	6	19,874,702	C	A	0.350	0.363	0.092	0.942	0.035	0.879	1.010	0.053	Imputed
rs1008655	6	19,875,302	G	A	0.246	0.235	0.118	1.063	0.039	0.985	1.149	0.710	Imputed
rs2142900	6	19,875,396	G	T	0.077	0.081	0.244	0.929	0.063	0.820	1.052	0.380	Imputed
rs2142899	6	19,875,552	A	G	0.348	0.362	0.092	0.942	0.035	0.879	1.010	0.061	Imputed
rs59293334	6	19,875,704	T	C	0.025	0.026	0.762	0.968	0.109	0.782	1.197	0.121	Imputed
rs7451842	6	19,875,756	T	C	0.153	0.160	0.291	0.952	0.047	0.868	1.043	0.734	Imputed
rs185978916	6	19,875,811	G	A	0.010	0.010	0.665	0.928	0.173	0.661	1.302	0.509	Imputed
rs1028330	6	19,875,846	A	G	0.348	0.362	0.092	0.942	0.035	0.879	1.010	0.061	Imputed
rs1016484	6	19,876,149	G	A	0.348	0.362	0.092	0.942	0.035	0.879	1.010	0.061	Imputed
rs1028329	6	19,876,199	T	C	0.348	0.362	0.092	0.942	0.035	0.879	1.010	0.061	Imputed
rs1016483	6	19,876,279	A	G	0.348	0.362	0.092	0.942	0.035	0.879	1.010	0.061	Imputed
rs1016482	6	19,876,323	A	G	0.348	0.362	0.092	0.942	0.035	0.879	1.010	0.061	Imputed
rs7755535	6	19,876,487	C	A	0.348	0.362	0.092	0.942	0.035	0.879	1.010	0.061	Imputed
rs7755971	6	19,876,492	A	G	0.348	0.362	0.092	0.942	0.035	0.879	1.010	0.061	Imputed
rs12528660	6	19,876,533	A	G	0.152	0.160	0.272	0.950	0.047	0.866	1.041	0.717	Genotyped
rs7756018	6	19,876,676	T	C	0.348	0.361	0.080	0.940	0.036	0.877	1.007	0.056	Imputed
rs760799	6	19,877,358	C	T	0.292	0.295	0.726	0.987	0.037	0.918	1.062	0.151	Imputed
rs879836	6	19,877,368	G	A	0.025	0.026	0.780	0.970	0.109	0.784	1.200	0.111	Imputed
rs9358281	6	19,877,392	T	C	0.403	0.401	0.860	1.006	0.034	0.940	1.076	0.152	Imputed
rs12529090	6	19,877,513	T	C	0.153	0.160	0.328	0.955	0.047	0.871	1.047	0.700	Imputed
rs760798	6	19,877,857	C	T	0.024	0.025	0.666	0.954	0.110	0.769	1.183	0.120	Imputed
rs760797	6	19,878,217	T	A	0.401	0.397	0.534	1.022	0.035	0.955	1.093	0.900	Imputed
rs1569515	6	19,878,354	T	C	0.400	0.397	0.545	1.021	0.035	0.954	1.093	0.921	Imputed
chr6:19878435:I	6	19,878,435	CA	C	0.202	0.205	0.485	0.971	0.042	0.894	1.055	0.019	Imputed
rs2328423	6	19,878,461	A	G	0.402	0.401	0.882	1.005	0.035	0.939	1.075	0.151	Genotyped
rs1569514	6	19,878,486	G	T	0.197	0.202	0.375	0.963	0.043	0.886	1.047	0.053	Imputed
rs4712436	6	19,878,487	C	G	0.153	0.160	0.272	0.950	0.047	0.866	1.041	0.809	Imputed
chr6:19878787:D	6	19,878,787	A	AT	0.197	0.202	0.356	0.961	0.043	0.885	1.045	0.059	Imputed
rs146765684	6	19,878,948	G	A	0.077	0.081	0.299	0.936	0.063	0.827	1.060	0.421	Imputed
rs6936631	6	19,878,973	A	G	0.198	0.203	0.361	0.962	0.042	0.885	1.045	0.067	Imputed
rs6936363	6	19,879,093	G	A	0.340	0.348	0.242	0.959	0.036	0.894	1.029	0.355	Imputed
rs73731216	6	19,879,351	A	G	0.024	0.026	0.619	0.947	0.110	0.763	1.174	0.148	Imputed
rs7772056	6	19,879,568	T	C	0.363	0.373	0.174	0.953	0.035	0.890	1.021	0.241	Genotyped
chr6:19879695:I	6	19,879,695	CA	C	0.167	0.170	0.684	0.982	0.045	0.898	1.073	0.923	Imputed
rs62873160	6	19,879,699	C	G	0.365	0.376	0.152	0.951	0.035	0.888	1.019	0.167	Imputed
rs7738643	6	19,880,369	G	A	0.105	0.102	0.470	1.041	0.055	0.934	1.160	0.774	Imputed
rs2876542	6	19,880,730	A	G	0.117	0.114	0.452	1.040	0.053	0.938	1.153	0.814	Genotyped

rs11754039	6	19,880,945	C	T	0.014	0.012	0.235	1.189	0.147	0.892	1.586	0.236	Imputed
rs765331	6	19,881,001	G	A	0.367	0.377	0.203	0.956	0.035	0.893	1.024	0.188	Imputed
rs6456283	6	19,881,259	G	T	0.369	0.379	0.180	0.954	0.035	0.891	1.022	0.166	Imputed
rs9465552	6	19,881,289	T	C	0.172	0.174	0.739	0.985	0.045	0.902	1.076	0.942	Imputed
rs6902310	6	19,882,091	G	T	0.171	0.173	0.689	0.982	0.045	0.899	1.073	0.923	Imputed
rs9460428	6	19,882,303	C	G	0.174	0.180	0.311	0.956	0.045	0.876	1.043	0.193	Imputed
rs6922818	6	19,882,389	G	A	0.024	0.026	0.572	0.940	0.110	0.758	1.165	0.153	Imputed
rs7745794	6	19,882,822	C	T	0.174	0.180	0.322	0.957	0.045	0.877	1.044	0.193	Imputed
rs71560107	6	19,882,956	G	T	0.023	0.021	0.483	1.084	0.115	0.866	1.358	0.189	Imputed
chr6:19883452:I	6	19,883,452	AC	A	0.174	0.180	0.322	0.957	0.045	0.877	1.044	0.193	Imputed
chr6:19883454:I	6	19,883,454	CA	C	0.174	0.180	0.322	0.957	0.045	0.877	1.044	0.193	Imputed
rs62404222	6	19,883,577	G	A	0.172	0.178	0.241	0.949	0.045	0.869	1.036	0.233	Imputed
rs11758945	6	19,883,664	A	G	0.053	0.052	0.934	1.006	0.076	0.868	1.167	0.442	Imputed
rs11758951	6	19,883,716	A	G	0.105	0.102	0.485	1.039	0.055	0.933	1.158	0.762	Imputed
chr6:19883968:D	6	19,883,968	A	AT	0.177	0.185	0.169	0.941	0.044	0.863	1.026	0.222	Imputed
rs7769851	6	19,884,065	C	G	0.173	0.179	0.298	0.955	0.045	0.875	1.042	0.203	Imputed
rs7769600	6	19,884,082	T	C	0.175	0.180	0.371	0.961	0.044	0.881	1.048	0.248	Genotyped
rs10946343	6	19,884,130	G	T	0.170	0.173	0.711	0.984	0.045	0.901	1.074	0.910	Imputed
rs9465553	6	19,884,225	T	C	0.174	0.180	0.322	0.957	0.045	0.877	1.044	0.193	Imputed
rs62404224	6	19,884,529	A	G	0.028	0.031	0.419	0.921	0.102	0.754	1.125	0.315	Imputed
rs9295452	6	19,884,562	A	G	0.174	0.179	0.327	0.957	0.045	0.877	1.045	0.208	Imputed
rs9295453	6	19,884,574	G	T	0.174	0.180	0.335	0.958	0.045	0.878	1.045	0.198	Imputed
rs56219935	6	19,885,192	A	G	0.118	0.114	0.401	1.045	0.053	0.943	1.159	0.854	Imputed
rs12332918	6	19,885,716	C	T	0.168	0.176	0.189	0.942	0.045	0.862	1.030	0.246	Imputed
rs6925952	6	19,885,772	C	T	0.174	0.180	0.338	0.958	0.045	0.878	1.046	0.199	Imputed
rs6903631	6	19,885,823	A	G	0.024	0.026	0.603	0.944	0.110	0.761	1.171	0.161	Imputed
rs6926130	6	19,885,880	C	T	0.178	0.183	0.406	0.964	0.044	0.884	1.051	0.124	Imputed
rs6903527	6	19,885,984	C	A	0.174	0.180	0.304	0.955	0.045	0.875	1.042	0.186	Imputed
rs2142898	6	19,886,068	G	A	0.237	0.225	0.077	1.073	0.040	0.992	1.160	0.574	Imputed
rs62402760	6	19,886,861	G	T	0.171	0.179	0.175	0.941	0.045	0.862	1.027	0.276	Imputed
rs72836099	6	19,887,041	T	C	0.173	0.179	0.290	0.954	0.045	0.874	1.041	0.233	Imputed
rs76689511	6	19,887,256	T	C	0.049	0.054	0.129	0.888	0.078	0.762	1.035	0.671	Imputed
rs10080611	6	19,887,307	G	T	0.173	0.179	0.257	0.951	0.045	0.871	1.038	0.235	Imputed
rs56256808	6	19,890,882	T	G	0.116	0.112	0.370	1.049	0.053	0.945	1.163	0.897	Imputed
rs73731228	6	19,890,884	C	T	0.113	0.112	0.998	1.000	0.054	0.901	1.111	0.559	Imputed
rs34661977	6	19,890,975	G	C	0.126	0.128	0.669	0.978	0.051	0.885	1.081	0.942	Imputed
rs34504929	6	19,891,315	A	G	0.097	0.099	0.572	0.968	0.057	0.866	1.083	0.695	Imputed
rs7769434	6	19,891,340	C	A	0.366	0.373	0.347	0.968	0.035	0.903	1.036	0.325	Genotyped
rs7752124	6	19,891,633	T	A	0.378	0.374	0.537	1.022	0.035	0.954	1.094	0.517	Imputed
rs9295454	6	19,892,077	C	T	0.215	0.215	0.927	1.004	0.041	0.926	1.088	0.877	Imputed

chr6:19892311:D	6	19,892,311	AG	A	0.254	0.256	0.854	0.993	0.039	0.920	1.072	0.840	Imputed
rs2056955	6	19,892,312	G	A	0.237	0.239	0.869	0.994	0.040	0.919	1.074	0.570	Imputed
rs2056954	6	19,892,586	T	C	0.183	0.185	0.922	0.996	0.044	0.914	1.085	0.476	Imputed
rs57677804	6	19,892,938	C	T	0.031	0.029	0.363	1.094	0.098	0.902	1.326	0.664	Imputed
rs926367	6	19,893,110	C	T	0.163	0.162	0.957	1.003	0.046	0.916	1.097	0.686	Genotyped
rs12524887	6	19,893,386	A	G	0.031	0.029	0.375	1.091	0.099	0.900	1.324	0.796	Imputed
chr6:19893693:I	6	19,893,693	T	TGTGCACAC	0.222	0.223	0.985	1.001	0.041	0.924	1.084	0.288	Imputed
chr6:19893698:I	6	19,893,698	A	ACACGTG	0.228	0.231	0.785	0.989	0.040	0.914	1.070	0.325	Imputed
chr6:19893701:I	6	19,893,701	C	CGT	0.285	0.292	0.468	0.973	0.038	0.904	1.048	0.555	Imputed
rs7756427	6	19,893,817	C	T	0.199	0.204	0.631	0.980	0.042	0.902	1.065	0.421	Imputed
rs10946345	6	19,893,852	C	A	0.301	0.302	0.807	0.991	0.037	0.922	1.065	0.946	Imputed
rs12203172	6	19,893,932	C	T	0.129	0.128	0.792	1.013	0.050	0.918	1.119	0.671	Genotyped
rs7756905	6	19,894,142	C	T	0.199	0.204	0.629	0.980	0.042	0.902	1.064	0.397	Imputed
rs7739799	6	19,894,725	T	C	0.199	0.203	0.628	0.980	0.042	0.902	1.064	0.410	Genotyped
rs75084674	6	19,894,752	C	T	0.031	0.029	0.328	1.100	0.098	0.908	1.333	0.688	Imputed
rs2179271	6	19,895,192	G	A	0.200	0.204	0.565	0.976	0.042	0.898	1.060	0.447	Imputed
chr6:19895632:D	6	19,895,632	GA	G	0.200	0.205	0.575	0.977	0.042	0.899	1.061	0.429	Imputed
rs926365	6	19,896,419	A	C	0.199	0.203	0.635	0.980	0.042	0.902	1.065	0.420	Imputed
rs723130	6	19,896,441	C	T	0.200	0.205	0.575	0.977	0.042	0.899	1.061	0.429	Imputed
rs76537555	6	19,897,183	A	G	0.031	0.029	0.317	1.103	0.098	0.910	1.336	0.697	Imputed
rs9356716	6	19,897,400	T	C	0.221	0.225	0.614	0.980	0.041	0.904	1.061	0.957	Imputed
rs7454654	6	19,897,730	A	G	0.213	0.216	0.702	0.984	0.041	0.908	1.067	0.378	Imputed
rs9348402	6	19,898,130	T	G	0.199	0.204	0.514	0.973	0.042	0.895	1.057	0.471	Imputed
rs2328422	6	19,898,475	G	C	0.221	0.225	0.590	0.978	0.041	0.903	1.060	0.989	Imputed
rs2328421	6	19,898,770	A	G	0.199	0.204	0.522	0.973	0.042	0.896	1.057	0.508	Genotyped
rs2328420	6	19,898,791	G	A	0.198	0.203	0.577	0.977	0.042	0.899	1.061	0.472	Imputed
rs28705059	6	19,898,860	C	T	0.199	0.203	0.628	0.980	0.042	0.902	1.064	0.435	Imputed
rs7745228	6	19,898,945	A	T	0.194	0.197	0.737	0.986	0.043	0.907	1.072	0.581	Imputed
rs7745659	6	19,899,172	A	C	0.193	0.196	0.747	0.986	0.043	0.907	1.073	0.670	Imputed
rs7746063	6	19,899,177	G	C	0.193	0.196	0.747	0.986	0.043	0.907	1.073	0.670	Imputed
rs7766026	6	19,899,248	T	C	0.193	0.196	0.747	0.986	0.043	0.907	1.073	0.670	Imputed
rs9368133	6	19,899,386	G	T	0.216	0.219	0.786	0.989	0.041	0.912	1.072	0.741	Imputed
rs9350195	6	19,899,405	T	C	0.193	0.196	0.747	0.986	0.043	0.907	1.073	0.670	Imputed
rs5010585	6	19,899,654	G	T	0.216	0.219	0.819	0.991	0.041	0.914	1.074	0.775	Genotyped
rs9358286	6	19,899,729	A	G	0.193	0.196	0.747	0.986	0.043	0.907	1.073	0.670	Imputed
rs9465562	6	19,899,734	C	G	0.193	0.196	0.747	0.986	0.043	0.907	1.073	0.670	Imputed
rs10946346	6	19,899,785	C	T	0.216	0.219	0.786	0.989	0.041	0.912	1.072	0.741	Imputed
rs9460433	6	19,900,006	G	T	0.214	0.216	0.969	0.998	0.041	0.921	1.082	0.512	Imputed
rs9366322	6	19,900,082	C	G	0.209	0.210	0.945	0.997	0.042	0.919	1.082	0.612	Imputed
chr6:19900392:I	6	19,900,392	T	TG	0.213	0.215	0.917	0.996	0.041	0.918	1.080	0.622	Imputed

rs2092223	6	19,900,441	C	T	0.193	0.196	0.747	0.986	0.043	0.907	1.073	0.670	Imputed
rs12665526	6	19,900,521	C	T	0.350	0.353	0.769	0.990	0.035	0.923	1.061	0.937	Imputed
rs2092222	6	19,900,556	T	C	0.193	0.196	0.747	0.986	0.043	0.907	1.073	0.670	Imputed
rs2092221	6	19,900,574	A	G	0.193	0.196	0.747	0.986	0.043	0.907	1.073	0.670	Imputed
rs2103599	6	19,900,983	C	A	0.193	0.196	0.747	0.986	0.043	0.907	1.073	0.670	Imputed
rs2092224	6	19,901,095	C	T	0.196	0.198	0.799	0.989	0.043	0.910	1.075	0.675	Imputed
rs148730452	6	19,901,267	T	C	0.022	0.021	0.697	1.047	0.116	0.833	1.314	0.048	Imputed
rs2103598	6	19,901,452	G	A	0.223	0.226	0.753	0.987	0.041	0.912	1.069	0.937	Imputed
rs4052745	6	19,901,485	G	A	0.199	0.203	0.661	0.982	0.042	0.904	1.067	0.433	Imputed
rs9368134	6	19,902,096	G	A	0.193	0.196	0.783	0.988	0.043	0.909	1.075	0.652	Imputed
rs9356717	6	19,902,134	A	G	0.193	0.196	0.783	0.988	0.043	0.909	1.075	0.652	Imputed
rs4712439	6	19,902,288	C	T	0.193	0.196	0.783	0.988	0.043	0.909	1.075	0.652	Imputed
rs116259261	6	19,902,303	A	G	0.022	0.021	0.697	1.047	0.116	0.833	1.314	0.048	Imputed
rs16882896	6	19,902,519	A	G	0.022	0.021	0.734	1.040	0.116	0.830	1.304	0.062	Imputed
rs4710921	6	19,902,789	A	G	0.193	0.196	0.782	0.988	0.043	0.909	1.075	0.653	Imputed
rs10946347	6	19,902,810	T	C	0.357	0.360	0.659	0.985	0.035	0.919	1.055	0.893	Genotyped
rs116805050	6	19,902,859	C	T	0.022	0.021	0.697	1.047	0.116	0.833	1.314	0.048	Imputed
rs7738820	6	19,903,134	G	C	0.193	0.196	0.777	0.988	0.043	0.908	1.074	0.655	Imputed
rs11751045	6	19,903,323	C	A	0.097	0.090	0.146	1.087	0.057	0.971	1.216	0.516	Imputed
rs9460435	6	19,903,324	C	G	0.199	0.203	0.655	0.981	0.042	0.903	1.066	0.436	Imputed
rs7739393	6	19,903,672	A	G	0.193	0.196	0.777	0.988	0.043	0.908	1.074	0.655	Imputed
rs7759843	6	19,903,688	T	C	0.193	0.196	0.777	0.988	0.043	0.908	1.074	0.655	Imputed
rs6456284	6	19,903,776	C	T	0.195	0.198	0.761	0.987	0.043	0.908	1.073	0.657	Imputed
rs6456285	6	19,903,838	A	G	0.195	0.198	0.765	0.987	0.043	0.908	1.073	0.656	Imputed
rs2038071	6	19,904,167	T	C	0.200	0.203	0.696	0.984	0.042	0.906	1.069	0.440	Imputed
rs4052759	6	19,904,696	G	A	0.224	0.226	0.838	0.992	0.041	0.916	1.074	0.281	Imputed
rs9465568	6	19,904,911	G	A	0.200	0.203	0.775	0.988	0.042	0.909	1.073	0.459	Imputed
rs9465569	6	19,904,942	T	C	0.201	0.204	0.770	0.988	0.042	0.909	1.073	0.457	Imputed
rs7749216	6	19,905,024	A	G	0.200	0.203	0.775	0.988	0.042	0.909	1.073	0.459	Imputed
chr6:19905166:I	6	19,905,166	C	CG	0.241	0.244	0.749	0.987	0.040	0.914	1.067	0.177	Imputed
rs7749505	6	19,905,167	A	G	0.232	0.235	0.821	0.991	0.040	0.916	1.072	0.203	Imputed
rs7749879	6	19,905,168	G	A	0.229	0.232	0.795	0.990	0.040	0.915	1.071	0.178	Imputed
rs187400838	6	19,905,169	C	T	0.017	0.018	0.438	0.904	0.131	0.699	1.168	0.093	Imputed
rs10946348	6	19,905,263	T	C	0.203	0.206	0.706	0.984	0.042	0.907	1.069	0.379	Imputed
rs7749991	6	19,905,501	A	C	0.200	0.203	0.749	0.987	0.042	0.908	1.072	0.470	Imputed
rs7750232	6	19,905,552	C	T	0.200	0.203	0.749	0.987	0.042	0.908	1.072	0.470	Imputed
rs1548229	6	19,905,747	A	G	0.200	0.203	0.779	0.988	0.042	0.910	1.073	0.508	Imputed
rs1931770	6	19,905,961	G	C	0.201	0.204	0.744	0.986	0.042	0.908	1.071	0.468	Imputed
rs6917838	6	19,906,088	A	G	0.201	0.204	0.744	0.986	0.042	0.908	1.071	0.468	Imputed
rs114581570	6	19,906,546	G	A	0.022	0.024	0.321	0.893	0.115	0.713	1.117	0.784	Imputed

rs1931771	6	19,906,567	T	C	0.201	0.204	0.744	0.986	0.042	0.908	1.071	0.468	Imputed
rs1997691	6	19,906,838	A	G	0.201	0.204	0.744	0.986	0.042	0.908	1.071	0.468	Genotyped
rs2038070	6	19,906,939	T	C	0.201	0.204	0.744	0.986	0.042	0.908	1.071	0.468	Imputed
rs76932818	6	19,907,055	T	C	0.022	0.021	0.613	1.061	0.116	0.845	1.332	0.040	Imputed
rs72837706	6	19,907,263	C	G	0.099	0.097	0.750	1.018	0.057	0.911	1.138	0.477	Imputed
rs114214903	6	19,907,448	G	T	0.022	0.021	0.613	1.061	0.116	0.845	1.332	0.048	Imputed
rs1931772	6	19,907,723	T	C	0.201	0.203	0.882	0.994	0.042	0.915	1.079	0.442	Imputed
chr6:19908426:I	6	19,908,426	GA	G	0.025	0.024	0.956	1.006	0.109	0.812	1.246	0.966	Imputed
rs1883328	6	19,908,495	A	G	0.143	0.142	0.676	1.020	0.048	0.928	1.122	0.674	Genotyped
rs6936399	6	19,909,241	G	A	0.041	0.043	0.620	0.959	0.085	0.811	1.133	0.572	Genotyped
rs62402792	6	19,909,355	A	G	0.017	0.015	0.434	1.110	0.134	0.854	1.443	0.902	Imputed
rs4052758	6	19,909,471	T	C	0.022	0.021	0.783	1.033	0.116	0.823	1.297	0.035	Imputed
rs1931773	6	19,909,736	A	G	0.041	0.043	0.586	0.955	0.085	0.808	1.129	0.619	Imputed
rs2225705	6	19,910,353	C	A	0.227	0.224	0.564	1.024	0.040	0.946	1.108	0.832	Imputed
rs77900408	6	19,910,382	T	G	0.040	0.042	0.627	0.959	0.086	0.810	1.135	0.722	Imputed
rs2179359	6	19,910,862	A	C	0.179	0.179	0.970	0.998	0.044	0.916	1.089	0.959	Imputed
rs75435909	6	19,911,663	G	A	0.040	0.042	0.647	0.961	0.086	0.812	1.138	0.744	Imputed
rs16882922	6	19,911,852	T	C	0.040	0.043	0.524	0.947	0.086	0.801	1.120	0.625	Genotyped
chr6:19911941:D	6	19,911,941	C	CCTGA	0.018	0.019	0.694	0.951	0.128	0.740	1.221	0.466	Imputed
rs76101188	6	19,912,108	G	C	0.040	0.043	0.524	0.947	0.086	0.801	1.120	0.625	Imputed
chr6:19912162:D	6	19,912,162	G	GAT	0.133	0.131	0.769	1.015	0.050	0.920	1.119	0.593	Imputed
chr6:19912166:D	6	19,912,166	G	GAT	0.144	0.145	0.857	0.991	0.048	0.902	1.090	0.988	Imputed
rs77793250	6	19,912,331	A	G	0.062	0.064	0.707	0.974	0.070	0.849	1.117	0.373	Imputed
rs1202287	6	19,912,620	G	A	0.227	0.224	0.583	1.022	0.040	0.945	1.107	0.795	Imputed
rs1202285	6	19,914,179	T	C	0.289	0.288	0.797	1.010	0.037	0.938	1.086	0.441	Imputed
rs4052756	6	19,914,494	T	C	0.494	0.489	0.494	1.024	0.034	0.958	1.094	0.896	Imputed
rs4052755	6	19,914,496	T	C	0.242	0.239	0.595	1.021	0.040	0.945	1.104	0.544	Imputed
rs1202284	6	19,914,725	A	C	0.226	0.224	0.635	1.019	0.040	0.942	1.104	0.765	Imputed
rs12661503	6	19,915,326	A	T	0.040	0.042	0.556	0.951	0.086	0.804	1.125	0.698	Imputed
rs1202312	6	19,916,257	A	G	0.431	0.428	0.739	1.011	0.034	0.946	1.081	0.749	Genotyped
rs79599019	6	19,917,011	C	A	0.022	0.021	0.803	1.030	0.116	0.820	1.293	0.033	Imputed
rs1202311	6	19,917,167	T	C	0.289	0.288	0.772	1.011	0.037	0.940	1.088	0.440	Imputed
chr6:19917285:D	6	19,917,285	A	AAC	0.018	0.016	0.610	1.068	0.130	0.829	1.377	0.988	Imputed
rs4563706	6	19,917,769	G	A	0.062	0.064	0.632	0.967	0.070	0.843	1.109	0.362	Imputed
chr6:19918178:I	6	19,918,178	AG	A	0.231	0.229	0.732	1.014	0.040	0.937	1.097	0.714	Imputed
rs1202310	6	19,918,183	G	A	0.240	0.236	0.500	1.027	0.040	0.950	1.110	0.822	Imputed
rs12110662	6	19,918,799	A	G	0.062	0.064	0.653	0.969	0.070	0.845	1.111	0.371	Imputed
rs112235797	6	19,919,026	A	G	0.040	0.042	0.553	0.950	0.086	0.803	1.125	0.732	Imputed
rs112885256	6	19,919,217	C	T	0.179	0.180	0.932	0.996	0.044	0.914	1.086	0.976	Imputed
rs72837711	6	19,919,882	T	C	0.102	0.100	0.658	1.025	0.056	0.919	1.144	0.683	Imputed

rs1202309	6	19,919,986	A	G	0.289	0.288	0.769	1.011	0.037	0.940	1.088	0.441	Genotyped
rs1202308	6	19,920,339	C	A	0.129	0.124	0.379	1.046	0.051	0.947	1.155	0.596	Genotyped
rs1202307	6	19,920,643	T	A	0.227	0.224	0.573	1.023	0.040	0.945	1.107	0.776	Imputed
rs7768737	6	19,920,904	T	C	0.040	0.042	0.599	0.956	0.086	0.808	1.131	0.706	Genotyped
rs10456227	6	19,921,581	T	C	0.053	0.055	0.533	0.954	0.075	0.823	1.106	0.107	Genotyped
rs1202306	6	19,921,870	A	T	0.227	0.224	0.566	1.023	0.040	0.946	1.108	0.748	Imputed
rs994646	6	19,922,050	T	A	0.040	0.042	0.559	0.951	0.086	0.803	1.126	0.729	Imputed
rs994645	6	19,922,228	A	G	0.132	0.126	0.260	1.058	0.050	0.959	1.167	0.562	Imputed
rs1202305	6	19,922,354	T	C	0.226	0.224	0.637	1.019	0.040	0.942	1.103	0.747	Genotyped
rs113512022	6	19,923,008	A	T	0.018	0.020	0.493	0.917	0.126	0.717	1.174	0.174	Imputed
rs9350196	6	19,923,212	T	C	0.172	0.173	0.895	0.994	0.045	0.911	1.085	0.243	Genotyped
rs149848120	6	19,923,859	T	A	0.012	0.012	0.627	1.078	0.154	0.797	1.459	0.351	Imputed
rs1202304	6	19,924,070	T	C	0.259	0.258	0.810	1.009	0.039	0.936	1.089	0.946	Imputed
rs1931802	6	19,924,318	G	A	0.290	0.293	0.634	0.982	0.037	0.913	1.057	0.340	Genotyped
rs1202303	6	19,925,026	G	A	0.226	0.224	0.628	1.020	0.040	0.942	1.104	0.736	Imputed
rs62402794	6	19,925,084	A	T	0.290	0.293	0.675	0.985	0.037	0.915	1.059	0.364	Imputed
rs1202302	6	19,925,960	G	T	0.226	0.223	0.613	1.021	0.040	0.943	1.105	0.745	Imputed
rs1202301	6	19,926,201	C	T	0.132	0.125	0.292	1.054	0.050	0.956	1.163	0.598	Imputed
rs78248154	6	19,926,974	C	T	0.040	0.042	0.624	0.959	0.086	0.810	1.135	0.692	Imputed
rs1202300	6	19,927,081	T	G	0.266	0.266	0.963	1.002	0.038	0.929	1.080	0.871	Imputed
rs1202299	6	19,927,717	T	G	0.227	0.223	0.530	1.026	0.040	0.948	1.110	0.845	Imputed
chr6:19927813:D	6	19,927,813	G	GAT	0.268	0.267	0.880	1.006	0.038	0.933	1.084	0.929	Imputed
chr6:19927815:D	6	19,927,815	T	TA	0.295	0.296	0.930	0.997	0.037	0.927	1.072	0.892	Imputed
rs75527812	6	19,928,134	T	C	0.028	0.032	0.217	0.883	0.101	0.724	1.076	0.937	Imputed
rs1202298	6	19,928,208	C	T	0.268	0.266	0.713	1.014	0.038	0.941	1.093	0.992	Imputed
rs1202297	6	19,928,775	T	C	0.128	0.122	0.312	1.053	0.051	0.953	1.163	0.617	Genotyped
rs1202296	6	19,929,121	A	G	0.325	0.317	0.313	1.037	0.036	0.966	1.113	0.291	Genotyped
rs1202295	6	19,929,168	C	A	0.405	0.404	0.870	1.006	0.034	0.940	1.076	0.758	Imputed
rs75051506	6	19,929,575	A	G	0.040	0.043	0.466	0.940	0.086	0.795	1.111	0.686	Imputed
rs12205916	6	19,929,577	A	C	0.224	0.222	0.746	1.013	0.041	0.936	1.097	0.697	Imputed
rs12194017	6	19,930,077	G	A	0.219	0.216	0.615	1.021	0.041	0.942	1.106	0.682	Imputed
rs6913980	6	19,930,198	A	G	0.045	0.048	0.350	0.927	0.081	0.790	1.087	0.628	Imputed
chr6:19930236:D	6	19,930,236	T	TG	0.213	0.216	0.561	0.976	0.041	0.900	1.059	0.793	Imputed
rs186460118	6	19,930,788	T	C	0.011	0.012	0.577	0.913	0.163	0.664	1.256	0.209	Imputed
rs114224505	6	19,930,830	A	G	0.018	0.019	0.642	0.943	0.127	0.735	1.209	0.349	Imputed
rs1202294	6	19,930,995	C	A	0.358	0.359	0.961	1.002	0.035	0.935	1.073	0.919	Imputed
rs9465581	6	19,931,360	A	G	0.221	0.217	0.580	1.023	0.041	0.944	1.108	0.767	Imputed
rs1202293	6	19,931,781	C	T	0.330	0.322	0.302	1.038	0.036	0.967	1.114	0.386	Genotyped
rs9358287	6	19,932,477	T	G	0.458	0.451	0.420	1.028	0.034	0.962	1.099	0.277	Imputed
chr6:19932639:D	6	19,932,639	T	TTTC	0.219	0.214	0.470	1.030	0.041	0.951	1.116	0.771	Imputed

rs149487143	6	19,932,706	G	A	0.049	0.051	0.549	0.954	0.078	0.819	1.112	0.540	Imputed
rs1202292	6	19,933,160	C	T	0.092	0.093	0.992	1.001	0.058	0.892	1.122	0.745	Genotyped
rs6899869	6	19,933,273	G	A	0.092	0.092	0.939	1.004	0.059	0.896	1.127	0.764	Imputed
rs1202291	6	19,933,791	G	A	0.092	0.092	0.957	1.003	0.058	0.895	1.125	0.773	Imputed
rs78865640	6	19,934,190	G	A	0.045	0.049	0.340	0.926	0.081	0.790	1.085	0.528	Imputed
rs62402795	6	19,934,553	C	G	0.037	0.037	0.873	0.986	0.090	0.826	1.176	0.548	Imputed
rs6911517	6	19,934,777	A	G	0.220	0.217	0.579	1.023	0.041	0.944	1.108	0.777	Imputed
rs6933991	6	19,934,807	C	T	0.220	0.217	0.579	1.023	0.041	0.944	1.108	0.777	Imputed
rs112520029	6	19,934,963	A	G	0.045	0.049	0.340	0.926	0.081	0.790	1.085	0.528	Imputed
rs1202288	6	19,935,512	C	T	0.266	0.267	0.975	1.001	0.038	0.929	1.079	0.983	Imputed
chr6:19935743:D	6	19,935,743	A	AC	0.045	0.049	0.340	0.926	0.081	0.790	1.085	0.528	Imputed
rs77737518	6	19,936,060	T	A	0.046	0.049	0.423	0.938	0.081	0.801	1.098	0.524	Imputed
rs150544005	6	19,936,318	T	C	0.011	0.011	0.678	1.069	0.161	0.780	1.465	0.211	Imputed
rs79449550	6	19,936,542	C	T	0.046	0.049	0.423	0.938	0.081	0.801	1.098	0.524	Imputed
rs79729833	6	19,936,598	T	C	0.046	0.049	0.419	0.937	0.080	0.800	1.097	0.527	Imputed
rs2782205	6	19,937,023	G	A	0.092	0.092	0.986	0.999	0.059	0.891	1.120	0.802	Imputed
rs13207878	6	19,937,112	G	A	0.219	0.216	0.606	1.021	0.041	0.943	1.107	0.761	Imputed
rs4052754	6	19,937,650	C	T	0.046	0.049	0.419	0.937	0.080	0.800	1.097	0.527	Imputed
rs75159079	6	19,937,949	T	C	0.009	0.011	0.406	0.863	0.177	0.610	1.220	0.149	Imputed
rs2744359	6	19,938,521	C	G	0.353	0.346	0.371	1.032	0.035	0.963	1.106	0.795	Imputed
rs6935955	6	19,938,977	A	C	0.052	0.054	0.563	0.957	0.076	0.825	1.111	0.237	Imputed
rs6935975	6	19,939,002	T	C	0.039	0.037	0.534	1.056	0.088	0.890	1.254	0.747	Imputed
rs187091263	6	19,939,212	C	T	0.014	0.015	0.860	0.975	0.142	0.739	1.288	0.851	Imputed
rs191656077	6	19,939,379	C	T	0.092	0.093	0.932	0.995	0.058	0.887	1.116	0.442	Imputed
rs183798210	6	19,939,567	A	C	0.249	0.248	0.810	1.009	0.039	0.935	1.090	0.902	Imputed
rs193297559	6	19,939,745	A	G	0.047	0.052	0.215	0.907	0.079	0.776	1.059	0.449	Imputed
rs185513192	6	19,939,768	T	C	0.076	0.079	0.535	0.961	0.064	0.849	1.089	0.264	Imputed
rs9350197	6	19,939,830	T	C	0.012	0.014	0.236	0.831	0.156	0.612	1.128	0.794	Imputed
rs140840093	6	19,939,944	A	G	0.290	0.300	0.146	0.947	0.037	0.881	1.019	0.260	Imputed
rs184237303	6	19,940,186	T	C	0.037	0.039	0.488	0.940	0.090	0.788	1.120	0.709	Imputed
rs71535517	6	19,940,340	G	C	0.198	0.201	0.699	0.984	0.043	0.905	1.069	0.580	Imputed
rs71535518	6	19,940,350	T	C	0.180	0.177	0.623	1.022	0.044	0.937	1.114	0.150	Imputed
rs186102839	6	19,940,351	A	G	0.026	0.029	0.214	0.876	0.107	0.711	1.080	0.798	Imputed
rs182570850	6	19,941,184	A	G	0.046	0.049	0.484	0.946	0.080	0.808	1.106	0.490	Imputed
rs12205014	6	19,941,422	G	A	0.219	0.217	0.660	1.018	0.041	0.940	1.103	0.730	Imputed
rs149042512	6	19,941,471	G	A	0.042	0.046	0.382	0.929	0.084	0.789	1.095	0.537	Imputed
rs114633668	6	19,942,295	T	C	0.009	0.012	0.094	0.742	0.179	0.522	1.053	0.804	Imputed
rs2744358	6	19,942,797	G	A	0.047	0.050	0.373	0.931	0.080	0.797	1.089	0.554	Imputed
chr6:19942947:D	6	19,942,947	A	AC	0.220	0.218	0.637	1.019	0.041	0.941	1.104	0.800	Imputed
rs9368136	6	19,942,951	G	C	0.300	0.306	0.344	0.966	0.037	0.898	1.038	0.943	Imputed

chr6:19942952:D	6	19,942,952	C	CT	0.220	0.218	0.637	1.019	0.041	0.941	1.104	0.800	Imputed
rs116225644	6	19,944,399	G	T	0.011	0.011	0.733	0.945	0.165	0.684	1.306	0.178	Imputed
rs2744357	6	19,944,506	G	A	0.266	0.267	0.975	1.001	0.038	0.929	1.079	0.983	Imputed
rs2782196	6	19,944,670	C	T	0.266	0.266	0.972	1.001	0.038	0.929	1.079	0.981	Imputed
rs12205022	6	19,944,698	A	G	0.219	0.216	0.615	1.021	0.041	0.942	1.106	0.789	Imputed
rs10946352	6	19,945,073	T	C	0.219	0.216	0.615	1.021	0.041	0.942	1.106	0.789	Imputed
rs1079801	6	19,945,285	T	C	0.046	0.049	0.479	0.945	0.080	0.808	1.106	0.622	Genotyped
rs12206820	6	19,945,599	T	C	0.219	0.216	0.615	1.021	0.041	0.942	1.106	0.789	Imputed
rs12206899	6	19,945,618	T	G	0.219	0.216	0.615	1.021	0.041	0.942	1.106	0.789	Imputed
rs150603644	6	19,945,663	T	C	0.012	0.012	0.987	0.998	0.156	0.735	1.354	0.087	Imputed
chr6:19945772:D	6	19,945,772	A	AATT	0.270	0.269	0.816	1.009	0.038	0.936	1.087	0.930	Imputed
chr6:19945774:D	6	19,945,774	T	TTA	0.270	0.270	0.954	1.002	0.038	0.930	1.080	0.840	Imputed
rs75822530	6	19,945,949	C	T	0.045	0.048	0.371	0.930	0.081	0.793	1.091	0.558	Imputed
rs2744356	6	19,946,002	A	C	0.047	0.050	0.376	0.932	0.080	0.797	1.090	0.576	Imputed
rs6917226	6	19,946,615	T	A	0.041	0.043	0.553	0.951	0.085	0.806	1.123	0.574	Imputed
rs2184393	6	19,946,730	G	A	0.358	0.359	0.990	1.000	0.035	0.934	1.072	0.773	Genotyped
rs1931794	6	19,947,189	T	C	0.267	0.266	0.914	1.004	0.038	0.932	1.082	0.991	Imputed
rs77436575	6	19,947,331	G	C	0.021	0.021	0.932	1.010	0.118	0.802	1.273	0.091	Imputed
rs1931795	6	19,947,411	C	A	0.266	0.266	0.938	1.003	0.038	0.931	1.081	0.996	Imputed
rs2744355	6	19,947,498	G	C	0.266	0.266	0.953	1.002	0.038	0.930	1.080	0.988	Imputed
rs12212015	6	19,947,777	T	C	0.139	0.137	0.606	1.026	0.049	0.932	1.129	0.889	Imputed
rs2179358	6	19,947,894	G	T	0.266	0.266	0.975	1.001	0.038	0.929	1.079	0.945	Imputed
rs2143046	6	19,948,028	A	G	0.041	0.043	0.439	0.936	0.085	0.792	1.107	0.587	Imputed
rs2143045	6	19,948,041	T	C	0.266	0.266	0.978	1.001	0.038	0.929	1.079	0.943	Imputed
chr6:19948099:D	6	19,948,099	A	AAG	0.266	0.266	0.965	1.002	0.038	0.929	1.080	0.950	Imputed
rs77877536	6	19,948,376	A	G	0.041	0.043	0.547	0.950	0.085	0.805	1.122	0.577	Imputed
rs79612475	6	19,948,386	A	T	0.041	0.043	0.547	0.950	0.085	0.805	1.122	0.577	Imputed
rs2782202	6	19,948,570	C	T	0.341	0.333	0.321	1.036	0.036	0.966	1.111	0.775	Imputed
rs67724988	6	19,948,659	T	C	0.224	0.222	0.721	1.015	0.041	0.937	1.099	0.742	Imputed
rs1931797	6	19,948,926	G	A	0.266	0.266	0.980	1.001	0.038	0.929	1.079	0.942	Imputed
chr6:19949086:I	6	19,949,086	CAT	C	0.269	0.271	0.924	0.996	0.038	0.925	1.074	0.774	Imputed
rs970508	6	19,949,092	T	G	0.035	0.039	0.240	0.899	0.091	0.752	1.074	0.463	Imputed
chr6:19949181:D	6	19,949,181	T	TG	0.267	0.267	0.982	1.001	0.038	0.929	1.079	0.947	Imputed
rs12665363	6	19,949,182	A	G	0.265	0.266	0.959	0.998	0.038	0.926	1.076	0.993	Imputed
rs1931800	6	19,949,417	A	G	0.266	0.266	0.980	1.001	0.038	0.929	1.079	0.942	Imputed
rs1931801	6	19,949,676	G	C	0.266	0.266	0.980	1.001	0.038	0.929	1.079	0.942	Imputed
rs2744353	6	19,949,948	T	C	0.266	0.267	0.961	1.002	0.038	0.930	1.080	0.984	Imputed
rs12202547	6	19,950,182	G	A	0.224	0.222	0.699	1.016	0.041	0.938	1.100	0.820	Imputed
rs9465586	6	19,950,364	A	G	0.266	0.267	0.961	1.002	0.038	0.930	1.080	0.984	Imputed
rs9465587	6	19,950,403	C	T	0.266	0.267	0.961	1.002	0.038	0.930	1.080	0.984	Imputed

rs2744351	6	19,950,508	C	T	0.266	0.267	0.961	1.002	0.038	0.930	1.080	0.984	Imputed
rs2782208	6	19,950,554	C	T	0.266	0.267	0.961	1.002	0.038	0.930	1.080	0.984	Imputed
rs2744350	6	19,950,622	T	C	0.266	0.267	0.961	1.002	0.038	0.930	1.080	0.984	Imputed
rs76628847	6	19,950,704	G	A	0.041	0.043	0.547	0.950	0.085	0.805	1.122	0.577	Imputed
chr6:19950838:D	6	19,950,838	C	CTA	0.223	0.220	0.642	1.019	0.041	0.941	1.104	0.762	Imputed
rs2744349	6	19,950,880	A	T	0.266	0.267	0.961	1.002	0.038	0.930	1.080	0.984	Imputed
rs2744348	6	19,951,023	T	C	0.266	0.267	0.961	1.002	0.038	0.930	1.080	0.984	Imputed
rs7742070	6	19,951,130	T	A	0.266	0.267	0.961	1.002	0.038	0.930	1.080	0.984	Imputed
rs12660402	6	19,951,328	T	C	0.019	0.021	0.424	0.905	0.125	0.709	1.155	0.617	Imputed
rs2744347	6	19,951,367	G	A	0.266	0.266	0.951	1.002	0.038	0.930	1.080	0.989	Imputed
rs4712441	6	19,951,426	A	G	0.224	0.222	0.693	1.016	0.041	0.939	1.100	0.824	Imputed
rs2782166	6	19,951,522	T	A	0.266	0.266	0.899	1.005	0.038	0.932	1.083	0.969	Imputed
chr6:19951560:I	6	19,951,560	GT	G	0.231	0.229	0.680	1.017	0.040	0.940	1.100	0.679	Imputed
chr6:19951564:I	6	19,951,564	TG	T	0.224	0.222	0.706	1.015	0.041	0.938	1.099	0.783	Imputed
rs185414266	6	19,951,565	G	T	0.018	0.019	0.712	0.954	0.127	0.743	1.224	0.203	Imputed
rs4712442	6	19,951,611	A	C	0.224	0.222	0.690	1.016	0.041	0.939	1.100	0.792	Imputed
rs2782167	6	19,951,693	G	A	0.266	0.267	0.961	1.002	0.038	0.930	1.080	0.984	Imputed
rs2782168	6	19,951,825	C	A	0.266	0.267	0.961	1.002	0.038	0.930	1.080	0.984	Imputed
rs12189818	6	19,951,890	C	T	0.224	0.222	0.690	1.016	0.041	0.939	1.100	0.792	Imputed
rs2782169	6	19,952,025	G	T	0.266	0.267	0.961	1.002	0.038	0.930	1.080	0.984	Imputed
rs2782170	6	19,952,073	A	G	0.266	0.267	0.961	1.002	0.038	0.930	1.080	0.984	Imputed
rs2782171	6	19,952,200	T	C	0.266	0.267	0.952	0.998	0.038	0.926	1.075	0.918	Imputed
chr6:19952232:D	6	19,952,232	T	TC	0.230	0.227	0.559	1.024	0.040	0.946	1.108	0.840	Imputed
rs2782173	6	19,952,290	T	G	0.266	0.267	0.961	1.002	0.038	0.930	1.080	0.984	Imputed
rs9465595	6	19,952,336	C	T	0.266	0.267	0.961	1.002	0.038	0.930	1.080	0.984	Imputed
rs13191976	6	19,952,404	C	T	0.224	0.222	0.694	1.016	0.041	0.938	1.100	0.790	Imputed
rs2782174	6	19,952,540	A	C	0.266	0.267	0.961	1.002	0.038	0.930	1.080	0.984	Imputed
rs9465597	6	19,952,857	A	T	0.266	0.267	0.980	0.999	0.038	0.927	1.077	0.984	Imputed
chr6:19952891:I	6	19,952,891	GT	G	0.266	0.267	0.961	1.002	0.038	0.930	1.080	0.984	Imputed
rs2206221	6	19,953,260	A	T	0.266	0.267	0.961	1.002	0.038	0.930	1.080	0.984	Imputed
rs2206220	6	19,953,382	T	C	0.266	0.267	0.961	1.002	0.038	0.930	1.080	0.984	Imputed
rs2206219	6	19,953,459	C	T	0.266	0.267	0.961	1.002	0.038	0.930	1.080	0.984	Imputed
rs10946353	6	19,953,512	C	T	0.224	0.222	0.690	1.016	0.041	0.939	1.100	0.792	Imputed
rs973776	6	19,953,696	A	G	0.266	0.267	0.961	1.002	0.038	0.930	1.080	0.984	Imputed
rs1931775	6	19,954,244	C	T	0.271	0.272	0.960	0.998	0.038	0.926	1.075	0.729	Imputed
rs2744346	6	19,954,334	G	C	0.265	0.265	0.919	1.004	0.038	0.931	1.082	0.984	Imputed
rs2744345	6	19,954,406	C	T	0.266	0.267	0.961	1.002	0.038	0.930	1.080	0.984	Imputed
rs75506772	6	19,954,481	A	C	0.041	0.043	0.541	0.950	0.085	0.805	1.121	0.580	Imputed
chr6:19954550:I	6	19,954,550	AG	A	0.266	0.266	0.938	1.003	0.038	0.931	1.081	0.980	Imputed
chr6:19954552:I	6	19,954,552	GT	G	0.266	0.267	0.961	1.002	0.038	0.930	1.080	0.984	Imputed

rs2744344	6	19,954,698	C	T	0.037	0.040	0.412	0.930	0.089	0.782	1.106	0.378	Imputed
rs2782175	6	19,954,908	G	A	0.266	0.267	0.961	1.002	0.038	0.930	1.080	0.984	Imputed
rs2782176	6	19,954,976	A	G	0.266	0.267	0.961	1.002	0.038	0.930	1.080	0.984	Imputed
rs2782177	6	19,954,987	G	A	0.266	0.267	0.961	1.002	0.038	0.930	1.080	0.984	Imputed
rs2782178	6	19,955,060	C	T	0.266	0.266	0.971	1.001	0.038	0.929	1.079	0.947	Imputed
rs2744343	6	19,955,068	T	C	0.266	0.266	0.971	1.001	0.038	0.929	1.079	0.947	Imputed
rs113434777	6	19,955,166	C	A	0.041	0.044	0.518	0.947	0.085	0.802	1.118	0.593	Imputed
rs9460443	6	19,955,209	A	G	0.266	0.267	0.961	1.002	0.038	0.930	1.080	0.984	Imputed
rs12215674	6	19,955,251	T	G	0.224	0.221	0.670	1.017	0.041	0.940	1.102	0.771	Imputed
rs9460444	6	19,955,260	A	G	0.266	0.265	0.909	1.004	0.038	0.932	1.083	0.948	Imputed
rs12202043	6	19,955,295	G	A	0.266	0.267	0.961	1.002	0.038	0.930	1.080	0.984	Imputed
rs9348403	6	19,955,306	C	T	0.341	0.333	0.343	1.034	0.036	0.965	1.109	0.781	Imputed
rs2782180	6	19,955,514	A	G	0.266	0.267	0.961	1.002	0.038	0.930	1.080	0.984	Imputed
rs2782181	6	19,955,533	A	C	0.266	0.267	0.967	0.998	0.038	0.926	1.076	0.987	Imputed
rs80037688	6	19,955,635	G	C	0.041	0.043	0.551	0.950	0.085	0.804	1.123	0.669	Imputed
chr6:19955653:D	6	19,955,653	T	TG	0.035	0.038	0.286	0.906	0.092	0.757	1.085	0.863	Imputed
rs2744342	6	19,955,755	C	T	0.266	0.266	0.899	1.005	0.038	0.932	1.083	0.969	Imputed
rs2744341	6	19,955,831	T	C	0.266	0.267	0.961	1.002	0.038	0.930	1.080	0.984	Imputed
rs2782182	6	19,956,121	C	G	0.266	0.267	0.961	1.002	0.038	0.930	1.080	0.984	Imputed
rs2782183	6	19,956,135	A	G	0.266	0.267	0.961	1.002	0.038	0.930	1.080	0.984	Imputed
rs2782184	6	19,956,164	A	G	0.266	0.267	0.961	1.002	0.038	0.930	1.080	0.984	Imputed
rs9460445	6	19,956,303	T	C	0.266	0.267	0.961	1.002	0.038	0.930	1.080	0.984	Imputed
rs9465601	6	19,956,368	A	G	0.267	0.267	0.920	1.004	0.038	0.931	1.082	0.949	Imputed
rs12190760	6	19,956,442	A	G	0.224	0.221	0.689	1.016	0.041	0.939	1.101	0.760	Imputed
rs4052748	6	19,956,537	C	T	0.266	0.265	0.840	1.008	0.038	0.935	1.086	0.957	Imputed
rs145936122	6	19,956,607	A	G	0.088	0.089	0.743	0.981	0.060	0.872	1.102	0.879	Imputed
rs2103630	6	19,956,620	A	G	0.252	0.251	0.896	1.005	0.039	0.931	1.085	0.888	Imputed
rs2103629	6	19,956,668	C	T	0.267	0.267	0.960	1.002	0.038	0.930	1.080	0.984	Imputed
rs2103628	6	19,956,670	T	C	0.267	0.267	0.960	1.002	0.038	0.930	1.080	0.984	Imputed
chr6:19956679:D	6	19,956,679	T	TA	0.041	0.043	0.439	0.936	0.085	0.792	1.107	0.587	Imputed
rs2782185	6	19,956,843	G	A	0.267	0.267	0.954	1.002	0.038	0.930	1.080	0.996	Genotyped
rs7766118	6	19,957,038	C	T	0.267	0.267	0.960	1.002	0.038	0.930	1.080	0.984	Imputed
rs2744338	6	19,957,282	A	G	0.042	0.044	0.525	0.948	0.084	0.804	1.118	0.589	Imputed
rs10946356	6	19,958,306	C	T	0.224	0.222	0.686	1.017	0.041	0.939	1.101	0.827	Imputed
rs10946357	6	19,958,392	C	T	0.225	0.223	0.717	1.015	0.041	0.937	1.099	0.843	Imputed
rs12194600	6	19,958,466	C	G	0.224	0.221	0.587	1.022	0.041	0.944	1.107	0.819	Imputed
rs970356	6	19,959,188	A	T	0.266	0.266	0.955	1.002	0.038	0.930	1.080	0.981	Imputed
rs2782187	6	19,959,784	A	G	0.340	0.331	0.340	1.035	0.036	0.965	1.110	0.731	Genotyped
rs12200012	6	19,961,172	G	C	0.224	0.222	0.671	1.017	0.041	0.940	1.102	0.862	Imputed
rs1931778	6	19,961,474	C	G	0.042	0.043	0.591	0.956	0.084	0.810	1.128	0.665	Imputed

rs12193940	6	19,963,459	T	C	0.224	0.222	0.739	1.014	0.041	0.936	1.098	0.874	Imputed
chr6:19963874:D	6	19,963,874	T	TCTC	0.223	0.219	0.509	1.027	0.041	0.949	1.112	0.727	Imputed
rs12195872	6	19,964,422	C	G	0.224	0.222	0.740	1.014	0.041	0.936	1.098	0.840	Imputed
chr6:19964883:D	6	19,964,883	C	CT	0.266	0.266	0.951	1.002	0.038	0.930	1.081	0.995	Imputed
rs12197383	6	19,964,968	T	C	0.224	0.222	0.740	1.014	0.041	0.936	1.098	0.840	Imputed
rs12204575	6	19,965,028	C	T	0.224	0.222	0.740	1.014	0.041	0.936	1.098	0.840	Imputed
rs1931782	6	19,965,482	A	G	0.265	0.266	0.989	1.001	0.038	0.928	1.079	0.958	Imputed
rs1931784	6	19,965,566	T	C	0.265	0.266	0.989	1.001	0.038	0.928	1.079	0.958	Imputed
rs77102136	6	19,965,722	A	G	0.062	0.063	0.913	0.992	0.070	0.866	1.138	0.500	Imputed
rs2782192	6	19,965,966	A	G	0.340	0.332	0.332	1.035	0.036	0.965	1.110	0.724	Imputed
rs2782193	6	19,966,014	C	A	0.266	0.266	0.999	1.000	0.038	0.928	1.078	0.964	Imputed
rs2782194	6	19,966,018	C	G	0.266	0.266	0.999	1.000	0.038	0.928	1.078	0.964	Imputed
rs2782195	6	19,966,110	T	C	0.340	0.332	0.360	1.033	0.036	0.963	1.108	0.744	Genotyped
rs1931786	6	19,966,473	A	G	0.173	0.170	0.713	1.017	0.045	0.931	1.110	0.483	Genotyped
rs1931787	6	19,966,621	A	G	0.266	0.267	0.971	0.999	0.038	0.926	1.076	0.979	Imputed
rs12529614	6	19,966,649	G	A	0.267	0.266	0.928	1.003	0.038	0.931	1.082	0.982	Genotyped
rs78409574	6	19,967,069	A	G	0.040	0.043	0.536	0.948	0.086	0.802	1.122	0.678	Imputed
rs12526695	6	19,967,188	C	G	0.265	0.266	0.992	1.000	0.038	0.928	1.078	0.960	Imputed
rs13217312	6	19,967,345	A	C	0.224	0.222	0.698	1.016	0.041	0.938	1.100	0.856	Imputed
rs56029040	6	19,967,825	A	G	0.093	0.093	0.855	1.011	0.058	0.902	1.133	0.714	Imputed
rs7759100	6	19,968,178	G	T	0.041	0.044	0.477	0.942	0.085	0.797	1.112	0.647	Imputed
chr6:19968182:D	6	19,968,182	A	AC	0.224	0.222	0.744	1.013	0.041	0.936	1.097	0.838	Imputed
rs7738895	6	19,968,313	T	C	0.267	0.267	0.917	1.004	0.038	0.932	1.082	0.794	Imputed
rs13195184	6	19,969,137	G	A	0.224	0.222	0.743	1.013	0.041	0.936	1.097	0.872	Imputed
rs11756396	6	19,969,304	A	G	0.341	0.333	0.407	1.030	0.036	0.960	1.105	0.724	Imputed
rs16883016	6	19,969,480	A	G	0.041	0.044	0.524	0.948	0.085	0.803	1.118	0.590	Genotyped
rs16883019	6	19,969,638	C	T	0.041	0.043	0.493	0.944	0.085	0.799	1.114	0.608	Imputed
rs16883021	6	19,969,712	A	G	0.041	0.043	0.493	0.944	0.085	0.799	1.114	0.608	Imputed
rs77620899	6	19,969,880	A	T	0.041	0.043	0.493	0.944	0.085	0.799	1.114	0.608	Imputed
rs12193640	6	19,970,270	G	A	0.225	0.223	0.753	1.013	0.041	0.936	1.097	0.934	Imputed
rs78101564	6	19,970,488	A	C	0.018	0.015	0.246	1.161	0.129	0.902	1.494	0.354	Imputed
chr6:19970528:I	6	19,970,528	AT	A	0.051	0.053	0.580	0.958	0.077	0.824	1.114	0.778	Imputed
chr6:19970709:D	6	19,970,709	C	CG	0.034	0.034	0.964	1.004	0.093	0.837	1.205	0.202	Imputed
rs7754644	6	19,970,784	G	A	0.276	0.278	0.809	0.991	0.038	0.920	1.067	0.981	Imputed
rs12528537	6	19,970,786	A	G	0.028	0.026	0.334	1.105	0.103	0.902	1.353	0.941	Imputed
rs62402829	6	19,970,837	C	G	0.339	0.333	0.437	1.028	0.036	0.959	1.103	0.662	Imputed
rs727165	6	19,971,817	C	G	0.340	0.334	0.555	1.021	0.036	0.952	1.095	0.762	Imputed
rs509674	6	19,972,222	C	T	0.225	0.223	0.777	1.012	0.041	0.934	1.095	0.894	Imputed
rs1931788	6	19,972,602	G	A	0.268	0.268	0.878	1.006	0.038	0.933	1.084	0.861	Imputed
rs12202520	6	19,972,938	T	C	0.225	0.223	0.784	1.011	0.041	0.934	1.095	0.840	Genotyped

chr6:19973130:D	6	19,973,130	T	TCC	0.033	0.034	0.834	0.980	0.095	0.814	1.180	0.119	Imputed
rs7775344	6	19,973,132	T	C	0.042	0.044	0.594	0.956	0.084	0.811	1.128	0.475	Imputed
rs62402830	6	19,973,248	G	A	0.335	0.329	0.475	1.026	0.036	0.956	1.101	0.606	Imputed
rs116138031	6	19,973,393	T	C	0.016	0.015	0.576	1.079	0.136	0.827	1.407	0.387	Imputed
rs544241	6	19,973,680	T	C	0.260	0.262	0.806	0.991	0.039	0.919	1.068	0.793	Imputed
rs76392898	6	19,974,661	A	G	0.040	0.042	0.518	0.946	0.086	0.799	1.120	0.636	Imputed
rs517374	6	19,975,946	A	G	0.256	0.258	0.747	0.988	0.039	0.915	1.066	0.598	Genotyped
rs17560702	6	19,976,217	T	C	0.468	0.470	0.759	0.990	0.034	0.926	1.058	0.980	Imputed
chr6:19976520:D	6	19,976,520	A	AT	0.187	0.197	0.091	0.929	0.043	0.854	1.012	0.162	Imputed
chr6:19976524:D	6	19,976,524	T	TA	0.285	0.289	0.515	0.976	0.037	0.907	1.050	0.322	Imputed
rs187132332	6	19,977,207	G	A	0.015	0.014	0.799	1.036	0.139	0.788	1.362	0.755	Imputed
chr6:19977932:I	6	19,977,932	TA	T	0.061	0.061	0.657	1.032	0.071	0.899	1.185	0.231	Imputed
rs76190010	6	19,978,200	A	G	0.020	0.020	0.777	1.035	0.121	0.817	1.311	0.666	Imputed
rs76018761	6	19,978,579	C	T	0.012	0.014	0.396	0.879	0.153	0.652	1.185	0.402	Imputed
rs115378303	6	19,979,276	A	G	0.009	0.011	0.212	0.802	0.177	0.567	1.136	0.440	Imputed
rs72837754	6	19,979,602	C	G	0.287	0.294	0.367	0.967	0.037	0.899	1.040	0.430	Imputed
rs16883030	6	19,980,733	A	T	0.284	0.291	0.321	0.964	0.037	0.895	1.037	0.340	Imputed
rs56279616	6	19,980,838	T	G	0.276	0.284	0.264	0.959	0.038	0.890	1.032	0.300	Imputed
rs10806910	6	19,980,846	G	A	0.194	0.195	0.878	0.994	0.043	0.913	1.080	0.345	Imputed
rs9368139	6	19,981,543	C	A	0.194	0.195	0.894	0.994	0.043	0.914	1.081	0.351	Imputed
rs1931789	6	19,981,546	G	A	0.276	0.284	0.274	0.960	0.038	0.891	1.033	0.328	Imputed
rs73730415	6	19,981,566	G	A	0.023	0.022	0.796	1.030	0.114	0.824	1.286	0.886	Imputed
rs62402832	6	19,981,919	C	T	0.033	0.034	0.838	0.981	0.094	0.815	1.180	0.661	Imputed
rs62402833	6	19,982,625	G	C	0.278	0.286	0.284	0.960	0.038	0.892	1.034	0.352	Imputed
rs76069627	6	19,983,114	A	G	0.018	0.019	0.722	0.956	0.128	0.744	1.227	0.344	Imputed
rs72837757	6	19,983,160	T	A	0.014	0.016	0.364	0.879	0.142	0.665	1.162	0.901	Imputed
rs9358290	6	19,983,213	A	T	0.194	0.195	0.901	0.995	0.043	0.915	1.082	0.353	Imputed
rs7768877	6	19,984,669	G	C	0.298	0.306	0.291	0.962	0.037	0.895	1.034	0.275	Imputed
chr6:19984857:D	6	19,984,857	AG	A	0.017	0.013	0.025	1.350	0.135	1.037	1.758	0.763	Imputed
rs11966096	6	19,985,972	G	A	0.098	0.097	0.621	1.029	0.057	0.920	1.150	0.788	Imputed
rs1931791	6	19,986,244	C	G	0.021	0.021	0.830	1.026	0.118	0.814	1.292	0.752	Imputed
rs78453421	6	19,987,136	A	G	0.022	0.021	0.679	1.049	0.117	0.835	1.319	0.675	Imputed
rs16883036	6	19,987,277	T	C	0.277	0.284	0.346	0.965	0.038	0.896	1.039	0.342	Imputed
rs12194143	6	19,987,811	T	G	0.196	0.197	0.861	0.993	0.043	0.913	1.079	0.356	Imputed
rs17560881	6	19,987,971	G	A	0.097	0.097	0.807	1.014	0.057	0.907	1.134	0.828	Imputed
rs74599689	6	19,988,215	T	C	0.021	0.021	0.739	1.040	0.117	0.826	1.309	0.705	Imputed
rs9358291	6	19,988,445	C	T	0.196	0.198	0.877	0.993	0.043	0.914	1.080	0.362	Imputed
rs9348404	6	19,988,653	C	A	0.196	0.198	0.871	0.993	0.043	0.914	1.080	0.403	Genotyped
rs72841030	6	19,988,704	T	C	0.034	0.036	0.531	0.943	0.093	0.785	1.133	0.673	Imputed
rs9465606	6	19,989,673	T	G	0.502	0.496	0.482	1.024	0.034	0.958	1.094	0.727	Genotyped

rs16883052	6	19,989,955	T	G	0.277	0.284	0.329	0.964	0.038	0.895	1.038	0.352	Imputed
chr6:19990190:I	6	19,990,190	GT	G	0.009	0.011	0.457	0.877	0.176	0.621	1.239	0.884	Imputed
rs1555006	6	19,990,299	C	T	0.277	0.284	0.333	0.964	0.038	0.895	1.038	0.349	Imputed
rs1555005	6	19,990,359	A	G	0.406	0.398	0.459	1.026	0.034	0.959	1.098	0.940	Imputed
rs965037	6	19,990,972	G	T	0.404	0.398	0.556	1.020	0.034	0.954	1.092	0.832	Genotyped
rs965036	6	19,991,043	G	T	0.288	0.284	0.658	1.017	0.037	0.945	1.094	0.643	Imputed
rs9295456	6	19,991,446	C	T	0.196	0.198	0.877	0.993	0.043	0.914	1.080	0.451	Imputed
chr6:19991928:I	6	19,991,928	G	GTAT	0.405	0.399	0.614	1.018	0.034	0.951	1.089	0.867	Imputed
chr6:19992164:I	6	19,992,164	TTA	T	0.022	0.021	0.604	1.062	0.116	0.846	1.334	0.798	Imputed
chr6:19992166:I	6	19,992,166	AT	A	0.022	0.021	0.604	1.062	0.116	0.846	1.334	0.798	Imputed
rs520188	6	19,992,265	G	A	0.098	0.094	0.306	1.060	0.057	0.948	1.186	0.353	Genotyped
rs78358355	6	19,992,272	G	A	0.067	0.066	0.648	1.031	0.068	0.903	1.178	0.451	Imputed
rs79297189	6	19,992,691	T	G	0.079	0.085	0.137	0.912	0.062	0.807	1.030	0.204	Imputed
rs7749252	6	19,992,762	T	C	0.239	0.239	0.916	1.004	0.040	0.929	1.085	0.403	Imputed
rs74591073	6	19,993,751	A	C	0.033	0.034	0.728	0.968	0.095	0.804	1.165	0.637	Imputed
rs9358292	6	19,995,731	A	G	0.196	0.198	0.843	0.992	0.043	0.912	1.078	0.424	Imputed
rs75082619	6	19,996,020	G	A	0.022	0.021	0.532	1.075	0.116	0.857	1.348	0.805	Imputed
rs73380643	6	19,996,377	A	G	0.018	0.018	0.798	0.968	0.129	0.752	1.245	0.827	Imputed
rs57321247	6	19,996,401	A	G	0.296	0.304	0.293	0.962	0.037	0.895	1.034	0.201	Imputed
rs9350204	6	19,996,808	C	A	0.196	0.198	0.831	0.991	0.043	0.912	1.077	0.432	Genotyped
rs9465608	6	19,997,176	A	G	0.415	0.421	0.564	0.980	0.034	0.917	1.049	0.253	Genotyped
rs78073769	6	19,997,466	G	A	0.257	0.259	0.791	0.990	0.039	0.918	1.068	0.147	Imputed
rs72841037	6	19,998,039	A	C	0.014	0.016	0.386	0.884	0.142	0.669	1.169	0.883	Imputed
rs190414953	6	19,998,309	A	G	0.018	0.019	0.677	0.949	0.126	0.741	1.216	0.541	Imputed
rs111239943	6	19,998,376	T	G	0.022	0.021	0.550	1.072	0.116	0.854	1.344	0.768	Imputed
rs9348407	6	19,998,422	A	T	0.196	0.198	0.840	0.991	0.043	0.912	1.078	0.423	Imputed
rs4513782	6	19,998,462	T	G	0.387	0.380	0.468	1.026	0.035	0.958	1.098	0.618	Imputed
rs9358294	6	19,999,122	G	A	0.200	0.201	0.950	0.997	0.042	0.918	1.084	0.421	Imputed
rs4712443	6	19,999,724	G	A	0.268	0.264	0.625	1.019	0.038	0.945	1.098	0.970	Imputed
rs9358295	6	20,000,010	T	C	0.196	0.198	0.790	0.989	0.043	0.910	1.075	0.416	Imputed
rs9368141	6	20,000,032	C	T	0.196	0.198	0.790	0.989	0.043	0.910	1.075	0.416	Imputed
rs7749480	6	20,000,048	G	A	0.486	0.479	0.339	1.033	0.034	0.967	1.104	0.525	Imputed
rs9689480	6	20,000,839	G	C	0.296	0.303	0.362	0.967	0.037	0.899	1.040	0.215	Imputed
rs7755724	6	20,001,699	A	C	0.221	0.221	0.937	1.003	0.041	0.926	1.087	0.520	Imputed
rs499899	6	20,001,747	A	G	0.295	0.302	0.335	0.965	0.037	0.897	1.038	0.214	Genotyped
rs10946358	6	20,001,927	T	G	0.197	0.199	0.795	0.989	0.043	0.910	1.075	0.431	Imputed
rs10806911	6	20,002,063	A	G	0.197	0.199	0.795	0.989	0.043	0.910	1.075	0.431	Imputed
rs185767927	6	20,002,577	T	C	0.295	0.301	0.426	0.971	0.037	0.903	1.044	0.245	Imputed
rs6915415	6	20,002,578	T	C	0.386	0.379	0.494	1.024	0.035	0.957	1.096	0.602	Imputed
rs7775315	6	20,003,157	T	C	0.221	0.220	0.921	1.004	0.041	0.927	1.088	0.564	Genotyped

rs9366325	6	20,003,304	A	G	0.197	0.199	0.806	0.990	0.043	0.910	1.076	0.435	Imputed
rs9366326	6	20,003,466	G	A	0.197	0.199	0.806	0.990	0.043	0.910	1.076	0.435	Imputed
rs77562520	6	20,003,564	T	C	0.022	0.021	0.541	1.073	0.116	0.856	1.346	0.762	Imputed
rs76230232	6	20,003,602	G	A	0.063	0.063	0.964	1.003	0.070	0.875	1.150	0.794	Imputed
rs115292823	6	20,003,785	T	C	0.030	0.030	0.856	0.982	0.099	0.808	1.193	0.436	Imputed
rs992893	6	20,004,051	A	G	0.386	0.378	0.423	1.028	0.035	0.961	1.101	0.583	Genotyped
rs140872290	6	20,005,581	C	T	0.019	0.019	0.818	1.029	0.125	0.806	1.314	0.670	Imputed
rs17561468	6	20,005,664	C	T	0.086	0.085	0.556	1.036	0.061	0.920	1.167	0.397	Imputed
rs56266936	6	20,006,182	C	T	0.084	0.083	0.565	1.036	0.061	0.919	1.168	0.532	Imputed
rs76648182	6	20,007,570	T	C	0.046	0.049	0.194	0.901	0.081	0.769	1.055	0.919	Imputed
rs116588059	6	20,009,085	C	T	0.031	0.027	0.091	1.179	0.098	0.974	1.429	0.705	Imputed
rs991447	6	20,009,666	C	A	0.354	0.367	0.157	0.951	0.035	0.888	1.019	0.092	Imputed
rs35007705	6	20,009,979	T	C	0.047	0.044	0.380	1.073	0.081	0.917	1.257	0.401	Imputed
rs591976	6	20,011,949	A	G	0.024	0.024	0.803	0.972	0.112	0.781	1.210	0.378	Imputed
rs35484089	6	20,012,160	C	T	0.020	0.027	0.011	0.742	0.118	0.589	0.934	0.165	Imputed
rs34497575	6	20,012,357	T	C	0.355	0.366	0.176	0.953	0.035	0.890	1.022	0.109	Imputed
rs149641711	6	20,013,004	C	T	0.015	0.018	0.087	0.790	0.138	0.602	1.036	0.850	Imputed
rs684274	6	20,013,085	C	T	0.411	0.422	0.239	0.960	0.034	0.898	1.027	0.300	Imputed
rs77574437	6	20,013,373	A	G	0.014	0.016	0.261	0.852	0.142	0.645	1.126	0.952	Imputed
rs476974	6	20,013,820	T	C	0.024	0.025	0.757	0.966	0.110	0.778	1.200	0.463	Imputed
rs13213659	6	20,013,937	G	A	0.350	0.361	0.192	0.955	0.035	0.891	1.024	0.083	Imputed
rs669307	6	20,014,089	C	T	0.405	0.416	0.210	0.958	0.034	0.895	1.025	0.262	Imputed
rs479905	6	20,014,181	T	C	0.025	0.025	0.890	0.985	0.110	0.795	1.221	0.402	Imputed
rs480696	6	20,014,225	G	T	0.016	0.013	0.068	1.282	0.137	0.981	1.677	0.296	Imputed
rs481747	6	20,014,366	G	A	0.379	0.392	0.161	0.952	0.035	0.890	1.020	0.175	Imputed
rs78152696	6	20,014,449	T	G	0.139	0.141	0.712	0.982	0.049	0.893	1.081	0.880	Imputed
rs667828	6	20,014,469	A	G	0.379	0.392	0.161	0.952	0.035	0.890	1.020	0.175	Imputed
rs114008520	6	20,014,605	T	C	0.026	0.025	0.666	1.048	0.108	0.848	1.294	0.465	Imputed
rs508416	6	20,014,918	T	C	0.358	0.372	0.102	0.944	0.035	0.881	1.011	0.244	Imputed
rs508418	6	20,014,919	G	A	0.358	0.372	0.102	0.944	0.035	0.881	1.011	0.244	Imputed
rs508473	6	20,014,938	T	C	0.358	0.372	0.108	0.945	0.035	0.882	1.013	0.237	Imputed
rs508474	6	20,014,939	G	A	0.358	0.372	0.102	0.944	0.035	0.881	1.011	0.244	Imputed
rs1202269	6	20,014,976	G	T	0.380	0.394	0.132	0.949	0.035	0.886	1.016	0.186	Imputed
rs510257	6	20,015,095	G	A	0.376	0.388	0.156	0.952	0.035	0.889	1.019	0.162	Imputed
rs11753077	6	20,015,205	G	T	0.350	0.362	0.190	0.955	0.035	0.891	1.023	0.084	Genotyped
rs511181	6	20,015,228	A	T	0.379	0.392	0.159	0.952	0.035	0.889	1.019	0.177	Imputed
rs653254	6	20,015,439	C	A	0.380	0.392	0.165	0.953	0.035	0.890	1.020	0.173	Imputed
rs641585	6	20,015,683	C	T	0.380	0.392	0.165	0.953	0.035	0.890	1.020	0.173	Imputed
rs515788	6	20,015,768	G	A	0.380	0.392	0.165	0.953	0.035	0.890	1.020	0.173	Imputed
rs640646	6	20,015,937	A	G	0.380	0.392	0.174	0.954	0.035	0.891	1.021	0.190	Imputed

rs639287	6	20,016,225	G	C	0.380	0.392	0.174	0.954	0.035	0.891	1.021	0.190	Imputed
rs637441	6	20,016,632	G	T	0.380	0.392	0.174	0.954	0.035	0.891	1.021	0.190	Imputed
rs545825	6	20,016,701	T	C	0.024	0.025	0.793	0.971	0.111	0.782	1.206	0.303	Imputed
rs625964	6	20,016,967	C	T	0.380	0.392	0.165	0.953	0.035	0.890	1.020	0.201	Genotyped
rs623329	6	20,017,533	C	T	0.407	0.417	0.263	0.962	0.034	0.899	1.029	0.301	Genotyped
rs574543	6	20,017,571	G	A	0.406	0.417	0.229	0.959	0.034	0.897	1.026	0.340	Imputed
rs622890	6	20,017,642	T	G	0.406	0.417	0.227	0.959	0.034	0.897	1.026	0.341	Genotyped
rs611623	6	20,017,799	A	G	0.380	0.392	0.176	0.954	0.035	0.891	1.021	0.236	Imputed
rs611083	6	20,017,962	G	T	0.406	0.417	0.230	0.960	0.034	0.897	1.027	0.339	Imputed
rs609844	6	20,018,206	A	T	0.406	0.417	0.227	0.959	0.034	0.897	1.026	0.341	Imputed
rs492591	6	20,018,376	G	A	0.029	0.030	0.671	0.958	0.101	0.787	1.167	0.203	Genotyped
rs608524	6	20,018,515	A	G	0.406	0.417	0.227	0.959	0.034	0.897	1.026	0.341	Imputed
rs597361	6	20,018,691	G	A	0.406	0.417	0.230	0.960	0.034	0.897	1.027	0.348	Imputed
rs596893	6	20,018,815	T	A	0.406	0.417	0.229	0.960	0.034	0.897	1.026	0.364	Imputed
rs77237487	6	20,019,036	G	A	0.406	0.417	0.227	0.959	0.034	0.897	1.026	0.341	Imputed
rs594089	6	20,019,443	T	C	0.406	0.417	0.227	0.959	0.034	0.897	1.026	0.341	Imputed
rs582382	6	20,019,738	T	C	0.407	0.418	0.236	0.960	0.034	0.897	1.027	0.306	Imputed
rs2457325	6	20,019,985	G	A	0.406	0.417	0.227	0.959	0.034	0.897	1.026	0.341	Imputed
rs72841063	6	20,020,039	G	A	0.039	0.036	0.204	1.118	0.088	0.941	1.329	0.681	Imputed
rs688580	6	20,020,113	T	A	0.406	0.417	0.263	0.962	0.034	0.899	1.029	0.359	Imputed
rs1202264	6	20,020,167	C	A	0.406	0.417	0.227	0.959	0.034	0.897	1.026	0.341	Imputed
rs1202263	6	20,020,189	C	A	0.406	0.417	0.227	0.959	0.034	0.897	1.026	0.341	Imputed
rs1202262	6	20,020,287	C	A	0.389	0.402	0.136	0.950	0.035	0.887	1.016	0.455	Imputed
rs647679	6	20,020,692	G	A	0.405	0.416	0.258	0.962	0.034	0.899	1.029	0.385	Imputed
rs646305	6	20,021,025	G	A	0.406	0.417	0.226	0.959	0.034	0.897	1.026	0.342	Imputed
rs2472784	6	20,021,671	C	T	0.406	0.417	0.237	0.960	0.034	0.898	1.027	0.316	Imputed
rs7763815	6	20,021,842	C	T	0.406	0.417	0.219	0.959	0.034	0.896	1.025	0.338	Genotyped
rs693897	6	20,021,990	A	C	0.025	0.025	0.809	0.974	0.109	0.786	1.207	0.402	Imputed
rs533323	6	20,022,013	A	C	0.406	0.417	0.225	0.959	0.034	0.897	1.026	0.334	Imputed
chr6:20023389:D	6	20,023,389	T	TTC	0.398	0.410	0.187	0.955	0.035	0.893	1.022	0.293	Imputed
chr6:20023390:D	6	20,023,390	T	TC	0.403	0.415	0.201	0.957	0.034	0.894	1.024	0.218	Imputed
chr6:20023546:D	6	20,023,546	T	TA	0.405	0.416	0.243	0.961	0.034	0.898	1.028	0.299	Imputed
chr6:20023555:D	6	20,023,555	A	AG	0.017	0.015	0.495	1.095	0.133	0.844	1.423	0.273	Imputed
rs6456288	6	20,023,589	A	C	0.377	0.386	0.313	0.965	0.035	0.902	1.034	0.161	Imputed
rs2472783	6	20,023,650	G	A	0.407	0.417	0.312	0.966	0.034	0.903	1.033	0.304	Imputed
rs4052730	6	20,023,748	A	G	0.337	0.347	0.240	0.959	0.036	0.894	1.028	0.097	Imputed
rs62404765	6	20,024,021	C	T	0.015	0.015	0.945	0.990	0.140	0.752	1.304	0.591	Imputed
rs4052731	6	20,024,178	A	G	0.329	0.341	0.196	0.955	0.036	0.890	1.024	0.067	Imputed
rs79992840	6	20,024,709	T	C	0.075	0.075	0.955	0.996	0.064	0.879	1.130	0.073	Imputed
rs7761069	6	20,025,404	T	G	0.328	0.340	0.181	0.953	0.036	0.888	1.023	0.050	Imputed

rs662751	6	20,027,638	G	A	0.329	0.340	0.180	0.953	0.036	0.888	1.023	0.055	Imputed
rs650802	6	20,027,988	A	T	0.257	0.270	0.101	0.938	0.039	0.869	1.012	0.361	Imputed
chr6:20028013:D	6	20,028,013	C	CTT	0.301	0.309	0.398	0.969	0.037	0.902	1.042	0.112	Imputed
rs6921993	6	20,028,015	C	T	0.329	0.341	0.190	0.954	0.036	0.889	1.024	0.095	Imputed
rs9348408	6	20,028,644	T	C	0.245	0.244	0.777	1.011	0.039	0.936	1.092	0.633	Genotyped
rs62404767	6	20,031,028	G	A	0.196	0.201	0.460	0.969	0.043	0.892	1.053	0.328	Imputed
chr6:20031214:I	6	20,031,214	CA	C	0.013	0.012	0.422	1.128	0.151	0.840	1.516	0.574	Imputed
rs588537	6	20,031,350	G	A	0.025	0.025	0.797	0.972	0.110	0.784	1.205	0.224	Imputed
rs564340	6	20,031,359	C	T	0.223	0.228	0.549	0.976	0.041	0.901	1.057	0.531	Imputed
rs683966	6	20,031,798	G	T	0.224	0.229	0.489	0.972	0.041	0.898	1.053	0.518	Imputed
rs683860	6	20,031,862	C	T	0.224	0.229	0.489	0.972	0.041	0.898	1.053	0.518	Imputed
rs683434	6	20,031,953	G	A	0.223	0.228	0.524	0.975	0.041	0.900	1.055	0.568	Imputed
rs682141	6	20,032,189	C	G	0.024	0.025	0.732	0.963	0.110	0.776	1.195	0.285	Imputed
chr6:20032199:I	6	20,032,199	GC	G	0.194	0.201	0.307	0.957	0.043	0.880	1.041	0.285	Imputed
rs142651372	6	20,032,295	C	A	0.011	0.011	0.710	0.941	0.164	0.683	1.297	0.316	Imputed
rs7770176	6	20,032,345	C	G	0.219	0.227	0.289	0.958	0.041	0.884	1.037	0.606	Imputed
rs681593	6	20,032,349	T	G	0.222	0.228	0.425	0.968	0.041	0.894	1.048	0.520	Imputed
rs192524558	6	20,032,498	T	C	0.014	0.016	0.421	0.892	0.143	0.673	1.180	0.312	Imputed
rs510191	6	20,032,912	C	G	0.359	0.371	0.158	0.952	0.035	0.888	1.020	0.195	Imputed
rs510872	6	20,032,947	C	G	0.219	0.226	0.370	0.964	0.041	0.890	1.044	0.530	Imputed
rs668493	6	20,032,949	G	A	0.219	0.226	0.377	0.965	0.041	0.891	1.045	0.539	Imputed
rs144774193	6	20,032,984	A	G	0.029	0.029	0.959	0.995	0.100	0.818	1.210	0.015	Imputed
rs511738	6	20,033,047	A	G	0.223	0.229	0.450	0.970	0.041	0.896	1.050	0.593	Imputed
rs667574	6	20,033,139	C	T	0.223	0.228	0.451	0.970	0.041	0.896	1.050	0.568	Imputed
chr6:20033361:I	6	20,033,361	AC	A	0.018	0.017	0.585	1.072	0.128	0.834	1.378	0.251	Imputed
rs72841073	6	20,033,389	A	G	0.041	0.044	0.448	0.938	0.085	0.795	1.107	0.573	Imputed
rs666247	6	20,033,459	C	T	0.224	0.229	0.484	0.972	0.041	0.898	1.052	0.558	Imputed
rs12202818	6	20,033,640	G	C	0.024	0.025	0.715	0.960	0.110	0.774	1.192	0.200	Imputed
rs186895816	6	20,033,768	G	T	0.013	0.012	0.705	1.060	0.153	0.786	1.429	0.679	Imputed
rs542154	6	20,034,122	T	C	0.222	0.228	0.417	0.968	0.041	0.894	1.048	0.629	Imputed
chr6:20034382:D	6	20,034,382	C	CT	0.227	0.231	0.538	0.975	0.040	0.901	1.056	0.569	Imputed
rs493024	6	20,034,781	T	C	0.363	0.372	0.288	0.963	0.035	0.899	1.032	0.218	Imputed
rs35446194	6	20,034,846	G	C	0.132	0.137	0.467	0.964	0.050	0.875	1.063	0.279	Imputed
rs638490	6	20,035,052	G	C	0.025	0.025	0.762	0.967	0.110	0.780	1.199	0.214	Imputed
rs495768	6	20,035,068	C	T	0.224	0.229	0.528	0.975	0.041	0.900	1.055	0.472	Genotyped
rs2457324	6	20,035,365	A	G	0.136	0.142	0.351	0.955	0.049	0.867	1.052	0.338	Imputed
rs637107	6	20,035,372	G	A	0.224	0.229	0.543	0.976	0.041	0.901	1.056	0.490	Imputed
rs76979774	6	20,036,142	A	G	0.018	0.020	0.392	0.898	0.126	0.701	1.149	0.916	Imputed
rs73380683	6	20,038,354	T	C	0.010	0.010	0.954	0.990	0.169	0.711	1.379	0.012	Imputed
rs687734	6	20,038,974	G	A	0.226	0.233	0.321	0.961	0.040	0.888	1.040	0.403	Genotyped

rs185491104	6	20,039,578	G	T	0.013	0.012	0.441	1.123	0.150	0.837	1.507	0.111	Imputed
rs2472779	6	20,039,645	G	A	0.195	0.204	0.212	0.948	0.043	0.872	1.031	0.226	Imputed
chr6:20040191:D	6	20,040,191	A	AAAAC	0.140	0.148	0.145	0.932	0.049	0.847	1.025	0.024	Imputed
rs17562088	6	20,041,024	C	T	0.023	0.024	0.831	0.976	0.113	0.783	1.217	0.150	Imputed
rs484043	6	20,041,792	T	G	0.027	0.026	0.787	1.029	0.106	0.837	1.265	0.176	Imputed
rs72841077	6	20,042,320	G	A	0.016	0.017	0.806	0.968	0.133	0.746	1.256	0.486	Imputed
rs490655	6	20,042,503	A	G	0.202	0.208	0.402	0.965	0.042	0.889	1.048	0.162	Imputed
rs2294182	6	20,042,648	G	C	0.194	0.204	0.191	0.946	0.043	0.870	1.028	0.216	Imputed
rs11757690	6	20,042,802	C	T	0.194	0.204	0.189	0.946	0.043	0.870	1.028	0.216	Imputed
rs78663358	6	20,042,852	T	C	0.048	0.046	0.577	1.045	0.079	0.895	1.221	0.286	Imputed
rs72841079	6	20,043,072	T	C	0.034	0.031	0.305	1.102	0.095	0.916	1.326	0.395	Imputed
rs62404768	6	20,043,379	A	G	0.194	0.204	0.194	0.946	0.043	0.870	1.029	0.222	Imputed
rs496514	6	20,044,563	C	T	0.224	0.231	0.312	0.960	0.041	0.887	1.039	0.580	Imputed
rs2472777	6	20,044,576	G	T	0.194	0.204	0.189	0.946	0.043	0.870	1.028	0.277	Imputed
rs594259	6	20,044,587	G	A	0.379	0.393	0.054	0.935	0.035	0.874	1.001	0.081	Imputed
rs582469	6	20,044,954	G	A	0.225	0.232	0.366	0.964	0.040	0.891	1.044	0.494	Imputed
chr6:20045089:I	6	20,045,089	CA	C	0.032	0.033	0.574	0.947	0.097	0.784	1.145	0.854	Imputed
rs78155513	6	20,045,375	G	C	0.082	0.085	0.427	0.952	0.061	0.845	1.074	0.766	Imputed
rs78851087	6	20,046,113	G	C	0.083	0.085	0.459	0.956	0.061	0.848	1.078	0.743	Imputed
rs532229	6	20,046,162	T	A	0.225	0.232	0.347	0.963	0.040	0.889	1.042	0.507	Imputed
rs533066	6	20,046,229	A	T	0.224	0.232	0.290	0.958	0.041	0.885	1.037	0.562	Imputed
rs558825	6	20,046,786	C	T	0.380	0.396	0.048	0.934	0.035	0.872	1.000	0.070	Genotyped
rs77046498	6	20,048,238	A	T	0.058	0.062	0.490	0.951	0.072	0.826	1.096	0.033	Imputed
rs2472775	6	20,049,211	T	G	0.266	0.284	0.019	0.915	0.038	0.849	0.986	0.177	Imputed
rs11753036	6	20,049,875	C	T	0.012	0.014	0.187	0.815	0.156	0.601	1.106	0.622	Imputed
rs9350206	6	20,050,161	C	T	0.254	0.251	0.627	1.019	0.039	0.944	1.100	0.957	Genotyped
rs542936	6	20,050,190	G	C	0.317	0.336	0.023	0.921	0.036	0.858	0.989	0.379	Imputed
rs149567003	6	20,050,244	T	C	0.033	0.029	0.189	1.135	0.096	0.940	1.370	0.358	Imputed
rs72841082	6	20,050,634	T	C	0.064	0.062	0.505	1.047	0.069	0.914	1.200	0.479	Imputed
rs72841083	6	20,050,751	C	G	0.069	0.065	0.305	1.071	0.067	0.939	1.222	0.566	Imputed
rs34986217	6	20,050,957	A	C	0.026	0.023	0.260	1.128	0.107	0.915	1.392	0.897	Imputed
rs34487350	6	20,050,984	G	C	0.026	0.023	0.260	1.128	0.107	0.915	1.392	0.897	Imputed
rs675439	6	20,052,552	T	C	0.028	0.025	0.164	1.154	0.103	0.943	1.413	0.967	Imputed
rs116740032	6	20,052,970	A	G	0.012	0.014	0.252	0.839	0.154	0.621	1.134	0.372	Imputed
rs2484058	6	20,053,560	T	C	0.286	0.309	3.60E-03	0.897	0.037	0.834	0.965	0.334	Imputed
rs548770	6	20,053,612	C	T	0.316	0.333	0.032	0.925	0.036	0.862	0.993	0.400	Imputed
rs549837	6	20,053,758	G	A	0.029	0.025	0.136	1.163	0.102	0.953	1.419	0.911	Imputed
rs71560111	6	20,053,768	G	C	0.051	0.056	0.108	0.885	0.076	0.762	1.028	0.068	Imputed

Table S2d IL33 region on chromosome 9

SNP	CHR	BP	Allele 1	Allele 2	F_A	F_U	P cmh test	OR cmh	SE	L95	U95	P het	Method
rs78052166	9	6,004,304	T	C	0.021	0.019	0.263	1.142	0.119	0.905	1.441	0.666	Imputed
rs7875455	9	6,004,618	A	T	0.220	0.218	0.707	1.016	0.041	0.937	1.100	0.572	Imputed
rs10815340	9	6,005,037	G	T	0.218	0.217	0.867	1.007	0.041	0.929	1.091	0.623	Imputed
rs77310481	9	6,007,122	A	G	0.061	0.062	0.762	0.979	0.071	0.852	1.124	0.773	Imputed
rs1061767	9	6,007,500	A	G	0.224	0.228	0.579	0.978	0.041	0.903	1.059	0.314	Imputed
rs2773341	9	6,007,767	A	C	0.011	0.010	0.762	1.051	0.164	0.762	1.450	0.170	Imputed
rs186636115	9	6,007,995	T	G	0.016	0.018	0.492	0.913	0.133	0.704	1.184	0.560	Imputed
rs4740835	9	6,008,571	C	G	0.218	0.217	0.865	1.007	0.041	0.929	1.091	0.615	Imputed
rs74917309	9	6,008,691	T	C	0.057	0.057	0.820	0.984	0.073	0.852	1.135	0.592	Imputed
rs78450583	9	6,009,220	G	A	0.060	0.060	0.857	0.987	0.071	0.858	1.135	0.497	Imputed
rs343477	9	6,009,761	A	G	0.293	0.290	0.680	1.015	0.037	0.944	1.092	0.650	Genotyped
chr9:6009967:I	9	6,009,967	AC	A	0.060	0.060	0.880	0.989	0.071	0.860	1.138	0.464	Imputed
rs79828803	9	6,010,015	C	G	0.044	0.048	0.225	0.905	0.082	0.770	1.064	0.705	Imputed
rs7853734	9	6,010,632	C	A	0.220	0.218	0.763	1.012	0.041	0.934	1.097	0.595	Imputed
rs41281759	9	6,011,134	A	G	0.030	0.029	0.510	1.067	0.099	0.879	1.296	0.756	Imputed
rs117996258	9	6,011,145	C	T	0.015	0.017	0.360	0.881	0.139	0.671	1.157	0.143	Imputed
rs7870774	9	6,011,330	T	G	0.012	0.011	0.813	1.038	0.159	0.761	1.418	0.242	Imputed
rs7425	9	6,011,335	C	T	0.220	0.219	0.821	1.009	0.041	0.932	1.093	0.667	Genotyped
rs10758736	9	6,011,757	A	G	0.223	0.219	0.518	1.027	0.041	0.948	1.112	0.469	Genotyped
rs106033	9	6,012,734	C	A	0.235	0.231	0.506	1.027	0.040	0.950	1.111	0.904	Imputed
rs343500	9	6,015,011	C	T	0.012	0.011	0.526	1.105	0.158	0.811	1.506	0.305	Genotyped
rs7858844	9	6,015,953	A	G	0.205	0.205	0.969	1.002	0.042	0.923	1.087	0.964	Imputed
rs2773340	9	6,016,125	T	C	0.011	0.011	0.761	1.050	0.162	0.764	1.444	0.126	Imputed
chr9:6016834:D	9	6,016,834	TA	T	0.011	0.010	0.763	1.050	0.164	0.762	1.449	0.146	Imputed
rs457313	9	6,016,983	A	G	0.011	0.012	0.826	0.965	0.162	0.704	1.325	0.169	Imputed
rs17580721	9	6,017,252	G	A	0.099	0.098	0.938	0.996	0.057	0.890	1.113	0.461	Imputed
rs2482124	9	6,018,316	G	A	0.334	0.335	0.893	0.995	0.036	0.928	1.068	0.586	Imputed
chr9:6019150:I	9	6,019,150	AG	A	0.010	0.011	0.526	0.898	0.169	0.645	1.252	0.557	Imputed
rs2777017	9	6,019,280	G	A	0.326	0.326	0.957	0.998	0.036	0.930	1.071	0.359	Imputed
rs111476692	9	6,019,624	T	C	0.015	0.019	0.092	0.794	0.136	0.608	1.038	0.203	Imputed
rs115269245	9	6,020,634	T	C	0.057	0.058	0.752	0.977	0.073	0.848	1.127	0.767	Imputed
rs58963098	9	6,020,955	A	C	0.253	0.257	0.571	0.978	0.039	0.906	1.056	0.485	Imputed
chr9:6020995:D	9	6,020,995	T	TG	0.011	0.010	0.750	1.054	0.164	0.764	1.454	0.144	Imputed
rs391401	9	6,021,034	C	T	0.325	0.327	0.756	0.989	0.036	0.921	1.061	0.397	Imputed
rs404267	9	6,021,181	A	C	0.011	0.010	0.600	1.089	0.163	0.791	1.498	0.221	Imputed
rs148813004	9	6,021,398	C	G	0.060	0.058	0.496	1.050	0.072	0.912	1.208	0.398	Imputed
rs376770	9	6,021,400	T	G	0.325	0.327	0.772	0.990	0.036	0.922	1.062	0.401	Imputed

rs146066500	9	6,021,592	T	C	0.058	0.058	0.831	0.985	0.073	0.854	1.135	0.782	Imputed
rs143463230	9	6,021,672	A	G	0.018	0.018	0.742	0.959	0.129	0.745	1.233	0.614	Imputed
rs10975398	9	6,021,979	A	G	0.310	0.314	0.490	0.975	0.037	0.908	1.048	0.466	Imputed
rs2618781	9	6,022,019	G	C	0.323	0.326	0.686	0.986	0.036	0.918	1.058	0.326	Imputed
chr9:6022076:I	9	6,022,076	AG	A	0.058	0.058	0.881	0.989	0.072	0.858	1.140	0.810	Imputed
rs77999730	9	6,022,526	C	G	0.058	0.058	0.876	0.989	0.072	0.858	1.140	0.807	Imputed
rs343480	9	6,023,030	G	A	0.011	0.010	0.525	1.107	0.161	0.808	1.518	0.094	Imputed
rs7859471	9	6,023,626	C	T	0.061	0.059	0.774	1.021	0.071	0.888	1.173	0.722	Imputed
rs10758739	9	6,023,927	C	T	0.252	0.256	0.559	0.978	0.039	0.906	1.055	0.565	Imputed
rs343481	9	6,024,285	G	C	0.324	0.326	0.777	0.990	0.036	0.922	1.062	0.434	Imputed
rs343482	9	6,024,347	G	T	0.011	0.010	0.600	1.089	0.163	0.791	1.497	0.121	Imputed
rs6477028	9	6,024,844	T	G	0.251	0.256	0.490	0.974	0.039	0.902	1.051	0.590	Imputed
rs343483	9	6,024,904	T	A	0.011	0.010	0.736	1.057	0.164	0.766	1.458	0.121	Imputed
rs343484	9	6,025,606	A	G	0.324	0.326	0.812	0.991	0.036	0.924	1.064	0.416	Imputed
chr9:6026007:D	9	6,026,007	C	CA	0.219	0.224	0.361	0.963	0.041	0.889	1.044	0.954	Imputed
chr9:6026008:D	9	6,026,008	A	AT	0.324	0.326	0.842	0.993	0.036	0.925	1.066	0.405	Imputed
rs343485	9	6,026,180	G	T	0.324	0.326	0.841	0.993	0.036	0.925	1.066	0.405	Imputed
rs7872052	9	6,026,757	C	T	0.252	0.256	0.570	0.978	0.039	0.906	1.056	0.661	Imputed
rs7028961	9	6,027,474	C	G	0.251	0.256	0.463	0.972	0.039	0.900	1.049	0.592	Imputed
rs7045750	9	6,027,590	C	A	0.251	0.256	0.438	0.970	0.039	0.899	1.047	0.510	Imputed
rs117324300	9	6,027,860	C	T	0.014	0.016	0.333	0.873	0.141	0.661	1.151	0.155	Imputed
rs6477029	9	6,027,931	C	T	0.310	0.315	0.473	0.974	0.037	0.907	1.046	0.563	Genotyped
rs343486	9	6,028,419	C	T	0.310	0.314	0.615	0.982	0.037	0.914	1.055	0.354	Imputed
rs77622058	9	6,029,494	G	A	0.057	0.058	0.885	0.990	0.073	0.858	1.141	0.979	Imputed
rs1094328	9	6,029,644	T	C	0.071	0.069	0.829	1.014	0.066	0.891	1.155	0.607	Imputed
chr9:6029723:I	9	6,029,723	TA	T	0.018	0.017	0.714	1.048	0.128	0.815	1.348	0.450	Imputed
rs12379805	9	6,030,387	T	C	0.204	0.204	0.954	0.998	0.042	0.919	1.083	0.879	Imputed
rs459718	9	6,030,699	C	T	0.012	0.012	0.701	1.060	0.153	0.786	1.432	0.593	Imputed
rs419595	9	6,030,917	C	T	0.013	0.012	0.444	1.121	0.149	0.837	1.501	0.382	Imputed
rs118031994	9	6,031,638	A	T	0.013	0.013	0.939	0.989	0.150	0.738	1.326	0.002	Imputed
rs192273112	9	6,032,174	A	G	0.014	0.017	0.287	0.861	0.142	0.652	1.136	0.226	Imputed
rs454045	9	6,032,792	A	C	0.266	0.268	0.820	0.991	0.038	0.920	1.069	0.424	Imputed
rs459525	9	6,032,924	T	C	0.266	0.268	0.784	0.990	0.038	0.918	1.067	0.427	Imputed
chr9:6033035:D	9	6,033,035	T	TC	0.026	0.029	0.222	0.879	0.106	0.714	1.082	0.096	Imputed
rs460564	9	6,033,078	C	G	0.012	0.011	0.564	1.093	0.155	0.807	1.480	0.351	Imputed
rs12378118	9	6,033,278	A	G	0.197	0.196	0.975	0.999	0.043	0.919	1.086	0.898	Genotyped
rs150975758	9	6,033,318	T	G	0.011	0.012	0.658	0.931	0.162	0.677	1.279	0.735	Imputed
rs2618780	9	6,033,581	T	A	0.274	0.275	0.831	0.992	0.038	0.921	1.068	0.520	Imputed
rs899381	9	6,034,076	C	T	0.014	0.012	0.358	1.144	0.147	0.858	1.524	0.392	Genotyped
rs76015832	9	6,034,464	A	G	0.057	0.058	0.746	0.977	0.073	0.847	1.126	0.581	Imputed

rs117479513	9	6,035,944	A	G	0.057	0.059	0.550	0.958	0.073	0.830	1.104	0.736	Imputed
rs411087	9	6,036,496	A	G	0.266	0.267	0.907	0.996	0.038	0.924	1.073	0.471	Imputed
chr9:6037856:I	9	6,037,856	GT	G	0.143	0.145	0.748	0.985	0.048	0.896	1.082	0.879	Imputed
rs402872	9	6,038,487	C	T	0.012	0.011	0.663	1.070	0.156	0.788	1.453	0.275	Imputed
rs10815344	9	6,038,695	C	T	0.258	0.261	0.699	0.985	0.039	0.913	1.063	0.674	Imputed
rs4742150	9	6,039,679	A	G	0.272	0.274	0.859	0.993	0.038	0.922	1.070	0.569	Imputed
rs58030850	9	6,040,876	T	C	0.268	0.271	0.687	0.985	0.038	0.914	1.061	0.505	Imputed
rs116881395	9	6,040,979	G	T	0.017	0.013	0.109	1.240	0.135	0.953	1.615	0.986	Imputed
rs670167	9	6,041,414	G	A	0.012	0.011	0.703	1.061	0.156	0.782	1.440	0.220	Imputed
rs629318	9	6,041,473	G	C	0.271	0.273	0.839	0.992	0.038	0.921	1.069	0.526	Imputed
rs10975406	9	6,041,603	A	T	0.259	0.261	0.762	0.988	0.039	0.916	1.066	0.664	Imputed
rs11532876	9	6,042,187	C	T	0.259	0.261	0.750	0.988	0.039	0.916	1.065	0.656	Imputed
rs10815345	9	6,042,708	A	G	0.270	0.272	0.822	0.992	0.038	0.920	1.068	0.552	Genotyped
rs343472	9	6,043,381	T	G	0.270	0.272	0.868	0.994	0.038	0.922	1.071	0.505	Imputed
rs343470	9	6,044,937	C	T	0.470	0.472	0.708	0.987	0.034	0.924	1.055	0.451	Imputed
rs343469	9	6,046,486	G	A	0.012	0.011	0.744	1.053	0.157	0.773	1.433	0.260	Imputed
rs7035152	9	6,046,694	T	G	0.249	0.253	0.559	0.977	0.039	0.905	1.055	0.668	Imputed
rs62557312	9	6,047,765	T	C	0.192	0.192	0.936	1.003	0.043	0.922	1.092	0.746	Imputed
rs10975410	9	6,047,766	A	G	0.461	0.462	0.966	0.999	0.034	0.934	1.067	0.791	Imputed
rs117328369	9	6,048,290	T	A	0.017	0.020	0.282	0.871	0.129	0.676	1.121	0.476	Imputed
rs10975412	9	6,049,547	G	A	0.193	0.191	0.640	1.020	0.043	0.938	1.110	0.508	Genotyped
rs10975413	9	6,049,843	G	A	0.194	0.194	0.980	1.001	0.043	0.921	1.089	0.637	Imputed
rs343478	9	6,051,399	A	G	0.455	0.455	0.945	0.998	0.034	0.933	1.066	0.714	Imputed
rs189349	9	6,051,597	G	A	0.448	0.447	0.938	1.003	0.034	0.938	1.072	0.705	Imputed
rs10975416	9	6,051,924	G	T	0.194	0.195	0.999	1.000	0.043	0.920	1.087	0.671	Genotyped
chr9:6052031:D	9	6,052,031	T	TC	0.043	0.041	0.726	1.030	0.084	0.874	1.213	0.263	Imputed
chr9:6052033:D	9	6,052,033	C	CT	0.460	0.462	0.779	0.991	0.034	0.927	1.059	0.957	Imputed
rs13290235	9	6,052,352	A	G	0.025	0.021	0.131	1.181	0.111	0.951	1.467	0.843	Imputed
rs183294115	9	6,052,933	G	C	0.378	0.373	0.555	1.021	0.035	0.953	1.094	0.677	Imputed
rs1330124	9	6,053,098	C	A	0.193	0.194	0.935	0.997	0.043	0.916	1.084	0.739	Imputed
rs13302000	9	6,053,382	C	T	0.055	0.055	0.904	0.991	0.075	0.856	1.147	0.263	Imputed
rs343479	9	6,054,314	C	T	0.195	0.195	0.993	1.000	0.043	0.920	1.088	0.576	Imputed
rs10758741	9	6,054,645	A	G	0.271	0.273	0.743	0.988	0.038	0.917	1.064	0.726	Imputed
rs72699150	9	6,054,794	T	C	0.034	0.038	0.320	0.912	0.092	0.762	1.093	0.633	Imputed
rs380568	9	6,055,531	C	T	0.193	0.194	0.926	0.996	0.043	0.916	1.083	0.796	Imputed
rs393556	9	6,056,468	G	A	0.193	0.194	0.904	0.995	0.043	0.915	1.082	0.740	Imputed
rs138927099	9	6,056,768	C	T	0.011	0.011	0.652	1.075	0.161	0.784	1.473	0.177	Imputed
rs10975418	9	6,057,011	G	A	0.193	0.195	0.924	0.996	0.043	0.916	1.083	0.751	Imputed
rs343498	9	6,059,145	A	T	0.193	0.195	0.924	0.996	0.043	0.916	1.083	0.751	Imputed
rs343499	9	6,059,157	G	A	0.193	0.195	0.924	0.996	0.043	0.916	1.083	0.751	Imputed

rs149887599	9	6,059,736	T	A	0.064	0.064	0.947	0.995	0.070	0.869	1.141	0.654	Imputed
rs9721567	9	6,060,143	C	T	0.276	0.281	0.521	0.976	0.038	0.906	1.051	0.902	Imputed
rs140433519	9	6,060,376	C	T	0.011	0.011	0.856	1.029	0.160	0.752	1.409	0.104	Imputed
rs182146546	9	6,061,258	C	G	0.022	0.021	0.937	1.009	0.116	0.804	1.267	0.596	Imputed
rs7040215	9	6,061,661	C	A	0.410	0.419	0.328	0.967	0.035	0.904	1.034	0.711	Imputed
rs6477032	9	6,061,779	G	C	0.399	0.401	0.829	0.993	0.035	0.927	1.063	0.395	Imputed
rs343487	9	6,062,269	G	A	0.192	0.193	0.877	0.993	0.043	0.913	1.081	0.960	Imputed
rs148334054	9	6,062,536	C	G	0.021	0.020	0.579	1.068	0.119	0.846	1.350	0.480	Imputed
rs10975420	9	6,062,676	G	C	0.269	0.272	0.712	0.986	0.038	0.915	1.063	0.932	Imputed
rs147128335	9	6,063,483	A	G	0.010	0.011	0.730	0.944	0.166	0.682	1.308	0.995	Imputed
rs343488	9	6,064,071	T	C	0.474	0.476	0.793	0.991	0.034	0.928	1.059	0.980	Imputed
rs10815347	9	6,064,103	A	G	0.215	0.217	0.693	0.984	0.041	0.908	1.067	0.691	Imputed
rs56705560	9	6,064,283	T	G	0.011	0.011	0.870	1.026	0.161	0.749	1.406	0.196	Imputed
rs343489	9	6,064,299	G	C	0.194	0.194	0.978	1.001	0.043	0.921	1.089	0.873	Imputed
rs117540688	9	6,064,428	C	T	0.011	0.011	0.939	1.013	0.164	0.735	1.396	0.347	Imputed
rs343490	9	6,064,575	G	A	0.192	0.193	0.956	0.998	0.043	0.917	1.085	0.967	Imputed
rs343491	9	6,064,640	C	G	0.192	0.193	0.925	0.996	0.043	0.916	1.083	0.950	Imputed
rs10758742	9	6,064,670	G	C	0.269	0.271	0.710	0.986	0.038	0.915	1.062	0.843	Imputed
rs343492	9	6,065,524	A	G	0.463	0.466	0.742	0.989	0.034	0.925	1.057	0.916	Imputed
rs10758743	9	6,066,175	C	T	0.278	0.280	0.721	0.987	0.038	0.916	1.062	0.710	Imputed
rs10739083	9	6,067,122	C	T	0.270	0.273	0.655	0.983	0.038	0.912	1.059	0.756	Genotyped
rs343497	9	6,067,744	T	C	0.466	0.467	0.960	0.998	0.034	0.934	1.067	0.966	Imputed
rs343496	9	6,068,077	T	A	0.188	0.187	0.860	1.008	0.043	0.926	1.097	0.792	Imputed
rs62558390	9	6,068,361	T	C	0.187	0.185	0.783	1.012	0.043	0.929	1.102	0.790	Imputed
chr9:6068879:D	9	6,068,879	A	AG	0.477	0.478	0.942	0.998	0.034	0.934	1.066	0.783	Imputed
rs7852365	9	6,068,910	T	C	0.163	0.159	0.519	1.030	0.046	0.942	1.127	0.957	Imputed
rs986295	9	6,069,435	A	G	0.163	0.159	0.561	1.027	0.046	0.939	1.124	0.978	Imputed
rs343495	9	6,069,445	C	T	0.192	0.193	0.963	0.998	0.043	0.918	1.086	0.988	Imputed
chr9:6069731:I	9	6,069,731	GT	G	0.116	0.120	0.504	0.965	0.053	0.871	1.070	0.691	Imputed
chr9:6069936:D	9	6,069,936	A	ACTGT	0.467	0.467	0.976	0.999	0.034	0.935	1.068	0.929	Imputed
rs17496153	9	6,070,037	C	G	0.067	0.069	0.645	0.969	0.068	0.849	1.107	0.957	Imputed
rs343494	9	6,070,342	T	C	0.477	0.477	0.959	1.002	0.034	0.937	1.070	0.759	Genotyped
rs343493	9	6,071,061	G	A	0.480	0.479	0.939	1.003	0.034	0.938	1.071	0.821	Imputed
chr9:6071623:D	9	6,071,623	A	AC	0.017	0.019	0.589	0.932	0.129	0.724	1.201	0.706	Imputed
rs7872100	9	6,071,843	T	C	0.133	0.135	0.687	0.980	0.050	0.889	1.081	0.506	Genotyped
rs184670	9	6,072,071	G	A	0.490	0.487	0.768	1.010	0.034	0.945	1.079	0.851	Imputed
rs343476	9	6,072,597	T	C	0.189	0.189	0.917	1.005	0.043	0.923	1.093	0.795	Genotyped
rs343475	9	6,073,013	C	G	0.189	0.189	0.953	1.003	0.043	0.921	1.091	0.822	Imputed
rs189348	9	6,073,194	T	C	0.189	0.189	0.937	1.003	0.043	0.922	1.092	0.768	Imputed
rs10975422	9	6,073,817	C	G	0.167	0.165	0.786	1.012	0.045	0.926	1.107	0.878	Imputed

rs343474	9	6,073,843	A	G	0.467	0.470	0.769	0.990	0.034	0.926	1.058	0.945	Imputed
rs411270	9	6,074,494	T	C	0.192	0.191	0.782	1.012	0.043	0.930	1.101	0.624	Imputed
rs404444	9	6,074,495	G	T	0.477	0.477	0.995	1.000	0.034	0.936	1.068	0.859	Imputed
rs343473	9	6,074,936	A	G	0.356	0.354	0.722	1.013	0.035	0.945	1.085	0.814	Genotyped
rs4742151	9	6,076,102	G	A	0.357	0.355	0.741	1.012	0.035	0.944	1.084	0.846	Imputed
rs149080671	9	6,076,641	T	C	0.022	0.021	0.623	1.059	0.117	0.843	1.331	0.645	Imputed
rs404582	9	6,076,777	T	A	0.370	0.369	0.792	1.009	0.035	0.942	1.081	0.902	Imputed
rs397505	9	6,076,823	T	C	0.191	0.191	0.971	1.002	0.043	0.921	1.090	0.721	Imputed
rs451974	9	6,076,844	A	G	0.193	0.193	0.956	1.002	0.043	0.922	1.090	0.618	Imputed
rs34133170	9	6,076,871	T	C	0.165	0.162	0.655	1.021	0.046	0.933	1.116	0.976	Imputed
rs10975424	9	6,077,716	C	T	0.122	0.127	0.398	0.957	0.052	0.865	1.059	0.433	Imputed
rs378952	9	6,078,146	C	T	0.191	0.191	0.976	1.001	0.043	0.920	1.090	0.719	Imputed
rs11793017	9	6,078,204	T	C	0.110	0.115	0.324	0.948	0.054	0.853	1.054	0.661	Imputed
rs371454	9	6,078,614	C	T	0.191	0.191	0.961	1.002	0.043	0.921	1.090	0.726	Imputed
rs76679494	9	6,078,724	A	G	0.021	0.022	0.670	0.951	0.117	0.756	1.197	0.433	Imputed
rs454664	9	6,078,763	A	G	0.356	0.353	0.679	1.015	0.035	0.947	1.088	0.796	Imputed
rs13299380	9	6,078,779	T	C	0.052	0.052	0.874	0.988	0.077	0.850	1.148	0.350	Imputed
rs77238775	9	6,078,936	T	C	0.017	0.018	0.598	0.934	0.129	0.725	1.203	0.711	Imputed
rs10975425	9	6,078,958	A	C	0.108	0.113	0.291	0.944	0.054	0.849	1.051	0.611	Imputed
rs401834	9	6,078,991	T	C	0.191	0.191	0.965	1.002	0.043	0.921	1.090	0.724	Imputed
rs79085296	9	6,079,398	A	G	0.021	0.021	0.853	0.979	0.117	0.778	1.232	0.092	Imputed
rs74194084	9	6,080,277	T	C	0.248	0.253	0.394	0.967	0.039	0.896	1.044	0.497	Imputed
rs10815349	9	6,080,298	A	G	0.110	0.116	0.286	0.944	0.054	0.850	1.049	0.620	Imputed
chr9:6080407:I	9	6,080,407	C	CT	0.194	0.195	0.939	0.997	0.043	0.917	1.084	0.845	Imputed
chr9:6080474:D	9	6,080,474	GC	G	0.198	0.198	0.974	0.999	0.042	0.919	1.085	0.954	Imputed
rs184646	9	6,080,758	C	A	0.489	0.488	0.954	1.002	0.034	0.938	1.071	0.744	Imputed
rs340935	9	6,080,998	C	G	0.194	0.195	1.000	1.000	0.043	0.920	1.087	0.825	Imputed
rs2150968	9	6,081,298	A	G	0.310	0.313	0.721	0.987	0.037	0.919	1.060	0.709	Imputed
rs340934	9	6,081,804	G	T	0.196	0.196	0.977	1.001	0.043	0.921	1.088	0.961	Imputed
rs143985683	9	6,082,627	G	C	0.017	0.019	0.547	0.925	0.129	0.718	1.192	0.728	Imputed
rs13302455	9	6,083,735	C	T	0.174	0.171	0.543	1.028	0.045	0.941	1.122	0.792	Imputed
rs340933	9	6,085,078	T	G	0.186	0.182	0.586	1.024	0.044	0.940	1.115	0.648	Genotyped
rs10975426	9	6,085,344	A	T	0.137	0.145	0.196	0.939	0.049	0.853	1.033	0.650	Imputed
rs7024677	9	6,086,073	T	C	0.128	0.135	0.197	0.937	0.051	0.849	1.034	0.994	Imputed
rs340932	9	6,086,293	T	G	0.502	0.498	0.662	1.015	0.034	0.950	1.085	0.963	Imputed
rs340931	9	6,086,443	G	A	0.324	0.330	0.443	0.973	0.036	0.906	1.044	0.889	Imputed
rs12377511	9	6,086,578	C	G	0.174	0.171	0.655	1.020	0.045	0.935	1.114	0.826	Imputed
rs145508294	9	6,086,680	T	C	0.018	0.019	0.574	0.932	0.127	0.726	1.195	0.081	Imputed
rs168168	9	6,086,695	A	C	0.186	0.184	0.793	1.011	0.044	0.929	1.102	0.805	Imputed
rs340929	9	6,086,714	C	T	0.334	0.338	0.645	0.984	0.036	0.917	1.055	0.983	Imputed

rs340928	9	6,086,913	A	G	0.029	0.029	0.970	0.996	0.102	0.816	1.216	0.039	Imputed
rs340927	9	6,087,178	A	G	0.497	0.500	0.720	0.988	0.034	0.925	1.056	0.843	Imputed
rs340926	9	6,087,252	T	G	0.187	0.185	0.760	1.013	0.043	0.931	1.103	0.640	Imputed
rs340925	9	6,088,398	T	G	0.186	0.184	0.795	1.011	0.044	0.929	1.101	0.724	Imputed
rs10120134	9	6,088,457	A	G	0.233	0.237	0.565	0.977	0.040	0.904	1.057	0.267	Genotyped
rs340924	9	6,088,525	T	C	0.186	0.184	0.781	1.012	0.044	0.929	1.102	0.731	Imputed
rs340923	9	6,088,628	G	A	0.186	0.184	0.805	1.011	0.044	0.928	1.101	0.736	Imputed
rs10975429	9	6,088,778	A	G	0.172	0.169	0.618	1.023	0.045	0.937	1.117	0.739	Imputed
rs340922	9	6,088,815	A	C	0.186	0.184	0.810	1.011	0.044	0.928	1.101	0.669	Imputed
rs12378311	9	6,088,903	C	G	0.172	0.169	0.611	1.023	0.045	0.937	1.117	0.743	Imputed
rs73386610	9	6,089,224	T	C	0.010	0.011	0.636	0.923	0.171	0.661	1.289	0.049	Imputed
rs4740837	9	6,089,527	T	C	0.128	0.135	0.217	0.940	0.050	0.851	1.037	0.961	Genotyped
rs340921	9	6,090,160	G	T	0.186	0.184	0.766	1.013	0.044	0.930	1.103	0.642	Imputed
rs340920	9	6,090,484	T	G	0.487	0.488	0.924	0.997	0.034	0.933	1.065	0.514	Imputed
rs340919	9	6,090,704	A	G	0.187	0.184	0.738	1.015	0.043	0.932	1.105	0.631	Imputed
rs10815350	9	6,090,711	A	G	0.129	0.135	0.249	0.944	0.050	0.855	1.041	0.978	Imputed
rs821164	9	6,090,989	C	G	0.186	0.184	0.757	1.014	0.043	0.931	1.104	0.622	Imputed
rs340918	9	6,091,048	A	C	0.186	0.184	0.757	1.014	0.043	0.931	1.104	0.622	Imputed
chr9:6091302:I	9	6,091,302	G	GA	0.187	0.184	0.732	1.015	0.043	0.932	1.105	0.685	Imputed
rs695013	9	6,091,565	C	T	0.186	0.184	0.775	1.013	0.044	0.930	1.103	0.613	Imputed
rs72699156	9	6,091,952	A	T	0.033	0.035	0.660	0.960	0.094	0.798	1.153	0.815	Imputed
rs531759	9	6,091,996	C	T	0.186	0.184	0.764	1.013	0.044	0.930	1.103	0.618	Imputed
rs639247	9	6,092,089	T	G	0.186	0.184	0.764	1.013	0.044	0.930	1.103	0.618	Imputed
rs420445	9	6,092,154	G	A	0.186	0.184	0.764	1.013	0.044	0.930	1.103	0.618	Imputed
rs79129775	9	6,092,339	C	T	0.015	0.017	0.454	0.903	0.137	0.690	1.181	0.335	Imputed
rs380888	9	6,092,395	T	G	0.186	0.184	0.764	1.013	0.044	0.930	1.103	0.618	Imputed
rs372560	9	6,092,399	T	C	0.186	0.184	0.764	1.013	0.044	0.930	1.103	0.618	Imputed
rs7856231	9	6,092,500	A	C	0.128	0.135	0.199	0.937	0.050	0.849	1.035	0.996	Imputed
rs503507	9	6,092,757	G	A	0.186	0.184	0.764	1.013	0.044	0.930	1.103	0.618	Imputed
rs503384	9	6,092,804	T	A	0.186	0.184	0.764	1.013	0.044	0.930	1.103	0.618	Imputed
rs371165	9	6,092,856	T	G	0.186	0.184	0.764	1.013	0.044	0.930	1.103	0.618	Imputed
chr9:6092989:D	9	6,092,989	TTA	T	0.186	0.184	0.764	1.013	0.044	0.930	1.103	0.618	Imputed
rs398561	9	6,093,132	G	A	0.186	0.184	0.753	1.014	0.044	0.931	1.104	0.608	Imputed
rs369996	9	6,093,192	T	A	0.186	0.184	0.764	1.013	0.044	0.930	1.103	0.618	Imputed
rs397916	9	6,093,345	G	A	0.186	0.184	0.764	1.013	0.044	0.930	1.103	0.618	Imputed
rs57993622	9	6,093,438	C	G	0.128	0.135	0.220	0.940	0.050	0.852	1.038	0.967	Imputed
rs140483043	9	6,093,627	C	G	0.017	0.019	0.354	0.887	0.129	0.689	1.142	0.312	Imputed
rs425489	9	6,093,790	T	C	0.186	0.184	0.735	1.015	0.044	0.932	1.105	0.577	Imputed
rs2150969	9	6,093,990	C	G	0.187	0.184	0.702	1.017	0.043	0.934	1.107	0.593	Imputed
rs399834	9	6,094,454	G	C	0.186	0.182	0.633	1.021	0.044	0.937	1.112	0.527	Imputed

rs390158	9	6,094,463	T	G	0.186	0.183	0.643	1.020	0.044	0.937	1.111	0.537	Imputed
rs1576463	9	6,094,755	T	C	0.172	0.168	0.562	1.026	0.045	0.940	1.121	0.691	Imputed
rs13294420	9	6,095,095	T	C	0.171	0.168	0.584	1.025	0.045	0.939	1.119	0.644	Imputed
chr9:6095223:I	9	6,095,223	GT	G	0.446	0.443	0.813	1.008	0.034	0.943	1.078	0.731	Imputed
rs111833758	9	6,095,477	G	A	0.133	0.142	0.089	0.919	0.050	0.834	1.013	0.607	Imputed
rs13300552	9	6,095,799	C	T	0.060	0.063	0.564	0.960	0.071	0.835	1.103	0.572	Imputed
rs10739084	9	6,096,189	G	C	0.185	0.182	0.654	1.020	0.044	0.936	1.111	0.570	Imputed
rs422499	9	6,096,288	T	A	0.497	0.499	0.825	0.993	0.034	0.929	1.061	0.667	Imputed
rs401731	9	6,096,349	A	C	0.186	0.183	0.673	1.019	0.044	0.935	1.109	0.608	Imputed
rs9408638	9	6,096,931	A	G	0.185	0.181	0.617	1.022	0.044	0.938	1.113	0.528	Imputed
rs10815351	9	6,097,088	G	C	0.497	0.499	0.846	0.993	0.034	0.930	1.062	0.678	Imputed
rs340894	9	6,097,164	T	C	0.185	0.181	0.617	1.022	0.044	0.938	1.113	0.528	Imputed
rs68075537	9	6,097,190	T	C	0.129	0.135	0.249	0.944	0.050	0.855	1.041	0.849	Imputed
rs340893	9	6,097,390	C	T	0.185	0.182	0.651	1.020	0.044	0.936	1.111	0.572	Imputed
rs10975431	9	6,097,430	A	T	0.181	0.178	0.718	1.016	0.044	0.932	1.108	0.910	Imputed
rs10975432	9	6,098,003	G	C	0.174	0.171	0.709	1.017	0.045	0.932	1.110	0.795	Imputed
rs182908	9	6,098,381	A	C	0.498	0.500	0.850	0.994	0.034	0.930	1.062	0.725	Imputed
rs340892	9	6,098,662	T	G	0.497	0.498	0.897	0.996	0.034	0.932	1.064	0.704	Genotyped
rs340891	9	6,098,868	G	C	0.498	0.500	0.839	0.993	0.034	0.929	1.061	0.719	Imputed
rs55817693	9	6,099,180	A	G	0.129	0.136	0.242	0.943	0.050	0.854	1.040	0.862	Imputed
rs437389	9	6,099,531	T	C	0.185	0.183	0.678	1.018	0.044	0.935	1.109	0.558	Imputed
rs340890	9	6,100,169	T	C	0.255	0.252	0.692	1.016	0.039	0.941	1.096	0.326	Imputed
rs10975434	9	6,100,215	A	G	0.042	0.038	0.223	1.109	0.085	0.939	1.309	0.909	Imputed
rs340889	9	6,100,476	G	C	0.332	0.335	0.729	0.988	0.036	0.921	1.060	0.760	Imputed
rs340888	9	6,101,163	C	T	0.336	0.340	0.579	0.980	0.036	0.914	1.052	0.858	Imputed
rs11526367	9	6,101,197	A	C	0.136	0.140	0.441	0.963	0.049	0.874	1.060	0.834	Imputed
rs13299104	9	6,101,432	T	C	0.175	0.171	0.582	1.025	0.045	0.939	1.118	0.850	Imputed
rs340887	9	6,101,481	T	A	0.187	0.184	0.748	1.014	0.043	0.931	1.104	0.570	Imputed
rs117052993	9	6,101,566	C	A	0.017	0.019	0.382	0.891	0.132	0.689	1.154	0.695	Imputed
rs2381384	9	6,101,939	A	C	0.064	0.064	0.937	0.995	0.069	0.869	1.139	0.519	Imputed
rs75916979	9	6,102,013	T	C	0.017	0.017	0.836	0.973	0.131	0.753	1.258	0.334	Imputed
rs2183352	9	6,102,599	A	C	0.138	0.144	0.343	0.955	0.049	0.867	1.051	0.871	Imputed
rs71503800	9	6,102,648	T	C	0.025	0.029	0.095	0.834	0.109	0.674	1.032	0.262	Imputed
rs340896	9	6,102,891	T	C	0.186	0.184	0.742	1.014	0.044	0.932	1.105	0.558	Imputed
rs2104758	9	6,102,947	G	A	0.138	0.143	0.335	0.954	0.049	0.867	1.050	0.844	Imputed
rs2149980	9	6,103,793	C	G	0.128	0.135	0.199	0.937	0.051	0.849	1.035	0.941	Imputed
rs340895	9	6,104,244	C	T	0.190	0.188	0.789	1.012	0.043	0.930	1.101	0.490	Imputed
rs34916503	9	6,105,050	C	T	0.175	0.172	0.570	1.026	0.045	0.940	1.119	0.858	Imputed
rs2890696	9	6,105,514	A	C	0.228	0.231	0.635	0.981	0.040	0.906	1.062	0.491	Imputed
chr9:6105674:I	9	6,105,674	TA	T	0.022	0.022	0.938	1.009	0.115	0.805	1.264	0.906	Imputed

rs340906	9	6,106,086	T	C	0.186	0.184	0.747	1.014	0.044	0.931	1.104	0.610	Imputed
rs340905	9	6,106,169	A	C	0.186	0.184	0.747	1.014	0.044	0.931	1.104	0.610	Imputed
chr9:6106402:D	9	6,106,402	T	TTC	0.011	0.015	0.042	0.722	0.161	0.527	0.989	0.194	Imputed
rs340904	9	6,106,779	C	A	0.186	0.184	0.737	1.015	0.044	0.932	1.105	0.600	Imputed
rs340903	9	6,107,113	C	G	0.186	0.184	0.747	1.014	0.044	0.931	1.104	0.610	Imputed
rs67478282	9	6,107,775	A	G	0.129	0.136	0.249	0.944	0.050	0.855	1.041	0.930	Imputed
rs340902	9	6,108,216	T	G	0.186	0.184	0.737	1.015	0.044	0.932	1.105	0.600	Imputed
rs340901	9	6,108,398	G	C	0.186	0.184	0.747	1.014	0.044	0.931	1.104	0.610	Imputed
rs10975436	9	6,108,729	C	T	0.104	0.101	0.623	1.028	0.056	0.922	1.146	0.714	Imputed
rs340900	9	6,108,921	T	C	0.473	0.466	0.361	1.031	0.034	0.965	1.102	0.631	Imputed
rs340899	9	6,109,080	C	T	0.473	0.466	0.352	1.032	0.034	0.966	1.103	0.593	Imputed
rs340898	9	6,109,516	A	C	0.472	0.464	0.349	1.032	0.034	0.966	1.103	0.597	Imputed
rs7024136	9	6,110,470	C	A	0.116	0.115	0.916	1.006	0.053	0.907	1.115	0.564	Imputed
rs340897	9	6,110,763	C	T	0.290	0.285	0.555	1.022	0.037	0.950	1.100	0.489	Imputed
rs7869888	9	6,111,007	T	C	0.104	0.101	0.644	1.026	0.056	0.920	1.144	0.703	Imputed
rs7869061	9	6,111,071	T	G	0.104	0.101	0.653	1.025	0.056	0.920	1.143	0.720	Imputed
rs7869064	9	6,111,078	T	G	0.104	0.101	0.644	1.026	0.056	0.920	1.144	0.703	Imputed
rs10975437	9	6,111,197	T	C	0.104	0.101	0.644	1.026	0.056	0.920	1.144	0.703	Imputed
rs189309	9	6,111,393	T	C	0.464	0.455	0.304	1.035	0.034	0.969	1.107	0.382	Imputed
rs7043663	9	6,111,603	T	C	0.101	0.099	0.688	1.023	0.056	0.916	1.142	0.755	Imputed
rs974936	9	6,111,703	A	C	0.186	0.184	0.747	1.014	0.044	0.931	1.104	0.610	Imputed
rs436300	9	6,112,044	G	C	0.464	0.458	0.466	1.025	0.034	0.959	1.096	0.620	Imputed
rs405594	9	6,112,261	G	A	0.290	0.285	0.552	1.022	0.037	0.950	1.100	0.490	Imputed
rs396183	9	6,112,501	G	A	0.290	0.285	0.535	1.023	0.037	0.951	1.101	0.499	Imputed
rs414320	9	6,112,532	C	T	0.290	0.285	0.533	1.024	0.037	0.951	1.101	0.500	Imputed
chr9:6113626:I	9	6,113,626	C	CA	0.214	0.214	0.968	1.002	0.041	0.924	1.086	0.919	Imputed
rs7040888	9	6,113,735	C	T	0.115	0.113	0.778	1.015	0.053	0.915	1.126	0.481	Imputed
rs441616	9	6,113,940	T	C	0.186	0.184	0.683	1.018	0.044	0.935	1.109	0.611	Imputed
rs439190	9	6,114,076	C	T	0.301	0.297	0.591	1.020	0.037	0.949	1.097	0.365	Imputed
rs391813	9	6,114,094	C	T	0.301	0.297	0.591	1.020	0.037	0.949	1.097	0.365	Imputed
rs7024235	9	6,114,095	A	G	0.115	0.113	0.796	1.014	0.053	0.914	1.125	0.490	Imputed
rs7024339	9	6,114,101	C	G	0.115	0.113	0.787	1.014	0.053	0.914	1.126	0.494	Imputed
rs7024340	9	6,114,102	T	G	0.115	0.113	0.787	1.014	0.053	0.914	1.126	0.494	Imputed
rs444826	9	6,114,483	G	C	0.484	0.477	0.432	1.027	0.034	0.961	1.097	0.429	Imputed
rs393410	9	6,114,512	A	G	0.290	0.283	0.404	1.032	0.037	0.959	1.110	0.530	Imputed
rs375560	9	6,114,744	T	C	0.186	0.184	0.683	1.018	0.044	0.935	1.109	0.611	Imputed
rs1556470	9	6,115,538	C	T	0.186	0.184	0.680	1.018	0.044	0.935	1.109	0.613	Imputed
rs440366	9	6,115,681	A	T	0.280	0.275	0.522	1.024	0.038	0.952	1.103	0.537	Imputed
rs410649	9	6,115,769	G	T	0.473	0.464	0.267	1.038	0.034	0.972	1.110	0.606	Imputed
rs10975441	9	6,116,053	T	C	0.102	0.098	0.464	1.042	0.056	0.934	1.163	0.907	Imputed

rs6651526	9	6,116,228	A	G	0.234	0.229	0.495	1.028	0.040	0.950	1.112	0.920	Imputed
rs4008366	9	6,116,407	C	T	0.310	0.309	0.969	1.001	0.037	0.932	1.076	0.329	Imputed
rs35829914	9	6,116,496	T	C	0.173	0.170	0.557	1.027	0.045	0.940	1.121	0.685	Imputed
chr9:6116543:I	9	6,116,543	A	AT	0.303	0.299	0.567	1.021	0.037	0.950	1.098	0.453	Imputed
rs2381385	9	6,116,666	C	T	0.301	0.297	0.597	1.020	0.037	0.949	1.096	0.394	Imputed
chr9:6116719:I	9	6,116,719	AC	A	0.103	0.104	0.785	0.985	0.056	0.883	1.098	0.449	Imputed
rs4742156	9	6,116,720	C	T	0.103	0.104	0.780	0.985	0.056	0.883	1.098	0.447	Imputed
rs4740839	9	6,116,721	T	G	0.103	0.104	0.780	0.985	0.056	0.883	1.098	0.447	Imputed
rs10114267	9	6,116,820	G	A	0.301	0.297	0.600	1.020	0.037	0.948	1.096	0.415	Imputed
chr9:6116849:I	9	6,116,849	TA	T	0.114	0.112	0.822	1.012	0.053	0.912	1.124	0.580	Imputed
rs13287430	9	6,116,936	C	A	0.173	0.170	0.565	1.026	0.045	0.940	1.120	0.743	Imputed
rs1591044	9	6,116,948	A	G	0.187	0.184	0.630	1.021	0.043	0.938	1.112	0.656	Imputed
rs182579341	9	6,116,979	G	C	0.016	0.017	0.681	0.947	0.133	0.730	1.228	0.503	Imputed
rs9407312	9	6,116,981	T	C	0.187	0.184	0.630	1.021	0.043	0.938	1.112	0.656	Imputed
rs1591045	9	6,117,094	A	G	0.290	0.283	0.398	1.032	0.037	0.959	1.110	0.600	Imputed
rs13290080	9	6,117,291	A	G	0.173	0.170	0.585	1.025	0.045	0.939	1.119	0.730	Imputed
rs694965	9	6,117,329	C	T	0.290	0.283	0.396	1.032	0.037	0.959	1.111	0.602	Imputed
rs118176494	9	6,117,562	A	G	0.021	0.025	0.106	0.827	0.118	0.657	1.041	0.509	Imputed
rs397542	9	6,118,043	T	C	0.301	0.297	0.577	1.021	0.037	0.950	1.097	0.414	Imputed
rs6477037	9	6,118,174	C	T	0.115	0.114	0.853	1.010	0.053	0.910	1.121	0.575	Genotyped
rs381486	9	6,118,911	G	A	0.301	0.297	0.607	1.019	0.037	0.948	1.096	0.430	Imputed
rs1411341	9	6,118,982	C	T	0.115	0.113	0.864	1.009	0.053	0.909	1.120	0.550	Imputed
rs374672	9	6,119,038	C	T	0.187	0.184	0.624	1.022	0.043	0.938	1.112	0.643	Imputed
rs10975444	9	6,119,329	G	A	0.175	0.172	0.567	1.026	0.045	0.940	1.119	0.635	Genotyped
rs374889	9	6,119,456	T	C	0.298	0.296	0.754	1.012	0.037	0.941	1.088	0.527	Imputed
rs380580	9	6,119,479	C	A	0.487	0.481	0.426	1.027	0.034	0.961	1.098	0.495	Imputed
rs10975445	9	6,119,512	C	T	0.174	0.171	0.584	1.025	0.045	0.939	1.118	0.731	Imputed
rs447778	9	6,119,627	G	T	0.304	0.300	0.636	1.018	0.037	0.947	1.094	0.432	Imputed
chr9:6119721:I	9	6,119,721	GA	G	0.117	0.115	0.674	1.022	0.053	0.922	1.134	0.813	Imputed
rs372482	9	6,119,872	G	A	0.302	0.297	0.577	1.021	0.037	0.950	1.097	0.437	Imputed
rs434521	9	6,120,137	C	A	0.301	0.297	0.600	1.020	0.037	0.948	1.096	0.426	Imputed
rs418014	9	6,120,336	C	T	0.301	0.297	0.600	1.020	0.037	0.948	1.096	0.426	Imputed
chr9:6120513:I	9	6,120,513	A	ATTAATTATATATAATTATA	0.204	0.203	0.873	1.007	0.042	0.927	1.093	0.730	Imputed
rs185272047	9	6,120,536	G	A	0.017	0.017	1.000	1.000	0.129	0.776	1.289	0.405	Imputed
rs1758599	9	6,120,543	T	C	0.331	0.325	0.483	1.026	0.036	0.956	1.101	0.813	Imputed
rs7048296	9	6,120,702	C	T	0.108	0.106	0.784	1.015	0.055	0.912	1.130	0.680	Imputed
rs186052001	9	6,120,917	G	T	0.011	0.013	0.214	0.817	0.163	0.593	1.124	0.803	Imputed
rs1755529	9	6,120,963	G	A	0.302	0.297	0.579	1.021	0.037	0.950	1.097	0.459	Imputed
rs369300	9	6,120,976	G	A	0.483	0.477	0.422	1.028	0.034	0.962	1.098	0.535	Imputed
rs453111	9	6,121,382	G	A	0.290	0.283	0.397	1.032	0.037	0.959	1.110	0.615	Imputed

rs442246	9	6,121,504	G	T	0.301	0.297	0.604	1.019	0.037	0.948	1.096	0.424	Imputed
rs392148	9	6,121,675	C	T	0.290	0.283	0.399	1.032	0.037	0.959	1.110	0.613	Imputed
rs13289987	9	6,121,900	A	T	0.177	0.174	0.672	1.019	0.044	0.934	1.112	0.768	Imputed
rs67217130	9	6,121,922	G	A	0.115	0.113	0.882	1.008	0.053	0.908	1.119	0.561	Imputed
rs381702	9	6,122,331	C	T	0.301	0.297	0.611	1.019	0.037	0.948	1.095	0.421	Imputed
rs693838	9	6,122,556	C	T	0.300	0.297	0.662	1.016	0.037	0.945	1.093	0.388	Genotyped
rs79537590	9	6,123,293	A	G	0.012	0.015	0.209	0.825	0.153	0.612	1.114	0.821	Imputed
rs10975446	9	6,123,309	C	T	0.114	0.113	0.875	1.008	0.053	0.909	1.119	0.545	Imputed
rs443175	9	6,123,556	T	C	0.187	0.184	0.627	1.021	0.043	0.938	1.112	0.641	Imputed
rs442851	9	6,123,782	C	T	0.187	0.184	0.627	1.021	0.043	0.938	1.112	0.641	Imputed
rs1332291	9	6,124,101	T	C	0.195	0.192	0.660	1.019	0.043	0.937	1.108	0.931	Imputed
rs1755531	9	6,124,250	T	G	0.187	0.184	0.627	1.021	0.043	0.938	1.112	0.641	Imputed
rs1970089	9	6,124,359	T	C	0.187	0.184	0.627	1.021	0.043	0.938	1.112	0.641	Imputed
rs1537285	9	6,124,584	T	G	0.187	0.184	0.640	1.021	0.043	0.937	1.111	0.650	Imputed
rs1332292	9	6,124,862	T	C	0.187	0.184	0.627	1.021	0.043	0.938	1.112	0.641	Imputed
rs7035594	9	6,125,353	A	T	0.187	0.184	0.619	1.022	0.043	0.938	1.113	0.645	Imputed
rs75485502	9	6,125,367	A	C	0.037	0.039	0.455	0.936	0.089	0.786	1.114	0.194	Imputed
rs7039066	9	6,125,539	T	C	0.187	0.184	0.627	1.021	0.043	0.938	1.112	0.641	Imputed
rs10975447	9	6,125,902	A	G	0.112	0.106	0.235	1.066	0.054	0.959	1.185	0.878	Imputed
rs340917	9	6,125,912	A	G	0.190	0.186	0.590	1.024	0.043	0.941	1.114	0.754	Imputed
rs340916	9	6,126,372	T	C	0.483	0.476	0.365	1.031	0.034	0.965	1.102	0.474	Imputed
rs77875869	9	6,126,403	T	A	0.011	0.013	0.215	0.820	0.160	0.599	1.122	0.199	Imputed
rs340915	9	6,126,588	A	G	0.187	0.184	0.630	1.021	0.043	0.938	1.112	0.639	Imputed
rs340914	9	6,126,799	A	G	0.187	0.184	0.630	1.021	0.043	0.938	1.112	0.639	Imputed
rs13296527	9	6,127,082	T	G	0.173	0.169	0.557	1.027	0.045	0.940	1.121	0.650	Imputed
rs340913	9	6,127,330	T	C	0.187	0.184	0.648	1.020	0.043	0.937	1.111	0.666	Imputed
chr9:6127338:I	9	6,127,338	TA	T	0.113	0.113	0.947	1.004	0.053	0.904	1.114	0.540	Imputed
rs10975448	9	6,127,514	T	A	0.108	0.107	0.783	1.015	0.055	0.912	1.130	0.681	Imputed
rs340912	9	6,127,851	A	G	0.187	0.184	0.620	1.022	0.043	0.938	1.113	0.587	Imputed
rs6477038	9	6,127,921	C	A	0.119	0.118	0.914	1.006	0.052	0.908	1.114	0.739	Imputed
rs72699172	9	6,128,000	T	C	0.029	0.030	0.791	0.974	0.100	0.800	1.185	0.971	Imputed
rs4742158	9	6,128,137	C	A	0.128	0.127	0.937	1.004	0.051	0.909	1.109	0.973	Imputed
rs340911	9	6,128,446	C	G	0.304	0.298	0.417	1.030	0.037	0.959	1.107	0.912	Imputed
chr9:6128617:I	9	6,128,617	TG	T	0.439	0.446	0.426	0.973	0.034	0.910	1.041	0.829	Imputed
rs340910	9	6,128,620	G	A	0.484	0.491	0.439	0.974	0.034	0.912	1.041	0.923	Imputed
rs74511614	9	6,128,642	A	T	0.017	0.017	0.617	0.937	0.131	0.725	1.211	0.146	Imputed
rs340909	9	6,128,646	G	A	0.304	0.297	0.401	1.031	0.037	0.960	1.109	0.893	Imputed
rs340908	9	6,128,897	T	C	0.187	0.184	0.604	1.023	0.043	0.939	1.114	0.596	Imputed
rs13293742	9	6,129,017	C	T	0.175	0.171	0.542	1.028	0.045	0.942	1.122	0.738	Imputed
rs340907	9	6,129,637	A	C	0.187	0.184	0.615	1.022	0.043	0.939	1.113	0.647	Imputed

rs10739086	9	6,130,822	C	T	0.315	0.312	0.642	1.017	0.036	0.947	1.092	0.667	Imputed
rs10114455	9	6,130,938	A	G	0.187	0.184	0.597	1.023	0.043	0.940	1.114	0.600	Imputed
rs10114457	9	6,130,940	A	G	0.187	0.184	0.601	1.023	0.043	0.940	1.114	0.598	Imputed
chr9:6130960:I	9	6,130,960	T	TAAC	0.188	0.185	0.690	1.017	0.043	0.935	1.108	0.581	Imputed
rs1888908	9	6,131,334	T	A	0.316	0.312	0.629	1.018	0.036	0.948	1.093	0.639	Imputed
rs1888906	9	6,131,460	G	A	0.188	0.184	0.574	1.025	0.043	0.941	1.116	0.557	Imputed
rs4742159	9	6,131,612	A	T	0.188	0.184	0.577	1.025	0.043	0.941	1.115	0.556	Imputed
rs694796	9	6,132,621	A	G	0.188	0.184	0.562	1.026	0.043	0.942	1.117	0.526	Imputed
rs118100385	9	6,132,625	A	G	0.044	0.040	0.260	1.098	0.083	0.933	1.293	0.874	Imputed
rs489464	9	6,132,664	A	T	0.316	0.312	0.614	1.019	0.036	0.948	1.094	0.612	Imputed
rs386412	9	6,132,904	T	A	0.316	0.312	0.614	1.019	0.036	0.948	1.094	0.612	Imputed
rs407153	9	6,133,014	C	T	0.489	0.482	0.385	1.030	0.034	0.964	1.100	0.586	Imputed
rs6477040	9	6,133,074	T	C	0.128	0.128	0.958	1.003	0.051	0.908	1.107	0.964	Genotyped
rs79300279	9	6,133,663	A	C	0.032	0.032	0.847	1.019	0.096	0.844	1.229	0.837	Imputed
rs10975450	9	6,133,672	T	A	0.173	0.170	0.599	1.024	0.045	0.938	1.118	0.851	Imputed
rs2026992	9	6,134,048	G	C	0.305	0.298	0.418	1.030	0.037	0.959	1.107	0.810	Imputed
rs138551099	9	6,134,212	G	A	0.015	0.012	0.215	1.193	0.142	0.903	1.576	0.388	Imputed
rs694294	9	6,134,402	C	T	0.316	0.312	0.616	1.018	0.036	0.948	1.094	0.632	Imputed
rs10975451	9	6,134,466	A	C	0.114	0.113	0.835	1.011	0.053	0.911	1.123	0.758	Imputed
rs10975452	9	6,134,510	A	G	0.172	0.170	0.640	1.021	0.045	0.935	1.115	0.772	Imputed
rs448115	9	6,134,642	G	A	0.316	0.312	0.616	1.018	0.036	0.948	1.094	0.632	Imputed
rs409038	9	6,134,750	A	G	0.316	0.311	0.596	1.020	0.036	0.949	1.095	0.643	Imputed
rs2069264	9	6,134,787	C	T	0.128	0.128	0.978	0.999	0.051	0.904	1.103	0.941	Imputed
rs376690	9	6,134,926	C	T	0.187	0.184	0.633	1.021	0.043	0.938	1.112	0.490	Imputed
rs376382	9	6,134,999	C	T	0.316	0.312	0.597	1.019	0.036	0.949	1.095	0.608	Genotyped
rs10118537	9	6,135,155	T	A	0.038	0.041	0.448	0.936	0.088	0.788	1.111	0.261	Imputed
rs10975454	9	6,135,411	A	C	0.120	0.119	0.882	1.008	0.052	0.910	1.116	0.668	Imputed
rs113396459	9	6,135,481	C	T	0.020	0.021	0.719	0.958	0.120	0.758	1.211	0.713	Imputed
rs77911193	9	6,135,571	T	C	0.007	0.012	2.95E-03	0.556	0.201	0.375	0.824	0.449	Imputed
rs2095043	9	6,135,847	C	T	0.316	0.312	0.651	1.017	0.036	0.947	1.092	0.627	Imputed
chr9:6136099:D	9	6,136,099	CG	C	0.322	0.318	0.575	1.021	0.037	0.950	1.096	0.686	Imputed
rs182220816	9	6,136,130	A	G	0.042	0.043	0.803	0.979	0.084	0.830	1.155	0.856	Imputed
rs6415796	9	6,136,147	T	G	0.026	0.029	0.361	0.908	0.106	0.738	1.117	0.902	Imputed
rs6415797	9	6,136,150	G	A	0.043	0.048	0.229	0.905	0.083	0.768	1.065	0.646	Imputed
rs6477041	9	6,136,219	C	T	0.126	0.124	0.785	1.014	0.051	0.917	1.121	0.880	Imputed
rs10975455	9	6,136,264	C	G	0.223	0.224	0.956	0.998	0.041	0.921	1.081	0.885	Imputed
rs4590488	9	6,136,268	G	A	0.494	0.496	0.895	0.996	0.034	0.931	1.064	0.512	Imputed
rs116880401	9	6,136,822	G	C	0.133	0.134	0.867	0.992	0.050	0.899	1.093	0.938	Imputed
rs147490634	9	6,136,898	C	T	0.107	0.105	0.763	1.017	0.055	0.913	1.132	0.968	Imputed
rs6477047	9	6,138,207	C	G	0.215	0.211	0.570	1.024	0.041	0.944	1.110	0.705	Imputed

rs7850455	9	6,138,327	C	A	0.191	0.187	0.531	1.027	0.043	0.944	1.118	0.616	Imputed
rs141495213	9	6,138,555	G	A	0.214	0.211	0.607	1.022	0.041	0.942	1.108	0.634	Imputed
rs145332328	9	6,138,891	A	G	0.486	0.493	0.390	0.971	0.034	0.909	1.038	0.876	Imputed
rs137963207	9	6,138,915	T	C	0.011	0.013	0.335	0.858	0.159	0.628	1.172	0.626	Imputed
rs146189858	9	6,139,009	G	A	0.322	0.317	0.546	1.022	0.036	0.952	1.098	0.809	Imputed
rs185694818	9	6,141,058	A	T	0.027	0.031	0.142	0.858	0.104	0.699	1.053	0.643	Imputed
rs9407324	9	6,141,823	G	C	0.196	0.194	0.732	1.015	0.043	0.933	1.103	0.671	Imputed
rs544253	9	6,142,157	C	A	0.310	0.315	0.540	0.978	0.037	0.910	1.050	0.972	Genotyped
rs394879	9	6,142,418	T	A	0.183	0.182	0.846	1.009	0.044	0.926	1.099	0.907	Imputed
rs10975460	9	6,142,674	A	G	0.210	0.208	0.717	1.015	0.042	0.936	1.101	0.705	Imputed
rs413382	9	6,142,948	C	A	0.191	0.190	0.932	1.004	0.043	0.923	1.092	0.841	Genotyped
rs10815357	9	6,144,025	G	A	0.209	0.211	0.796	0.989	0.042	0.912	1.073	0.998	Imputed
rs10815358	9	6,144,065	A	G	0.207	0.209	0.794	0.989	0.042	0.912	1.073	0.979	Genotyped
rs386880	9	6,144,333	C	T	0.304	0.304	0.952	0.998	0.037	0.928	1.072	0.855	Imputed
rs387149	9	6,145,022	C	T	0.319	0.326	0.357	0.967	0.036	0.901	1.038	0.893	Genotyped
rs12349559	9	6,145,491	C	T	0.078	0.078	0.952	0.996	0.063	0.880	1.128	0.369	Genotyped
rs406322	9	6,146,121	T	C	0.238	0.252	0.066	0.930	0.040	0.860	1.005	0.416	Imputed
rs369756	9	6,146,441	T	G	0.218	0.229	0.132	0.940	0.041	0.868	1.019	0.791	Imputed
rs384766	9	6,146,800	C	T	0.246	0.259	0.074	0.932	0.039	0.863	1.007	0.576	Imputed
rs10975463	9	6,149,006	G	A	0.090	0.089	0.848	1.011	0.059	0.901	1.136	0.592	Genotyped
rs13298861	9	6,150,279	T	A	0.089	0.087	0.637	1.028	0.059	0.916	1.155	0.638	Imputed
rs111286410	9	6,150,622	C	T	0.022	0.022	0.868	1.019	0.115	0.813	1.278	0.907	Imputed
rs116898132	9	6,151,129	C	T	0.034	0.032	0.412	1.080	0.094	0.899	1.297	0.424	Imputed
rs1012715	9	6,151,320	A	C	0.065	0.066	0.803	0.983	0.069	0.860	1.124	0.475	Imputed
rs10975464	9	6,151,609	C	G	0.089	0.087	0.705	1.023	0.059	0.910	1.149	0.674	Imputed
rs450108	9	6,153,485	C	T	0.398	0.408	0.177	0.954	0.035	0.892	1.021	0.460	Imputed
rs60831665	9	6,153,708	A	C	0.083	0.084	0.790	0.984	0.061	0.873	1.109	0.969	Imputed
rs141347273	9	6,154,012	A	G	0.016	0.016	0.901	1.017	0.134	0.783	1.321	0.803	Imputed
rs74309218	9	6,154,139	G	T	0.030	0.029	0.709	1.038	0.100	0.854	1.263	0.167	Imputed
rs75188761	9	6,154,158	A	G	0.066	0.067	0.709	0.975	0.068	0.853	1.115	0.383	Imputed
rs117089548	9	6,154,200	A	G	0.009	0.012	0.083	0.732	0.180	0.514	1.042	0.241	Imputed
chr9:6154722:I	9	6,154,722	CT	C	0.090	0.088	0.646	1.028	0.059	0.915	1.154	0.579	Imputed
rs138994175	9	6,154,874	T	C	0.016	0.016	0.878	1.021	0.134	0.786	1.326	0.791	Imputed
rs10975465	9	6,155,014	A	G	0.066	0.066	0.820	0.985	0.068	0.861	1.126	0.370	Imputed
rs1116795	9	6,155,226	T	G	0.396	0.407	0.151	0.952	0.035	0.889	1.018	0.400	Imputed
chr9:6155340:D	9	6,155,340	C	CT	0.267	0.250	0.019	1.095	0.039	1.015	1.181	0.619	Imputed
rs10815359	9	6,155,865	T	C	0.394	0.406	0.130	0.949	0.035	0.887	1.015	0.409	Imputed
rs2183912	9	6,155,928	A	T	0.397	0.409	0.120	0.948	0.035	0.886	1.014	0.430	Imputed
rs12352464	9	6,157,329	T	C	0.065	0.066	0.778	0.981	0.069	0.858	1.122	0.372	Imputed
rs12352510	9	6,157,433	T	C	0.065	0.066	0.817	0.984	0.069	0.861	1.126	0.368	Imputed

rs75735167	9	6,158,026	G	A	0.011	0.014	0.199	0.814	0.161	0.594	1.115	0.966	Imputed
rs10117792	9	6,158,778	C	T	0.066	0.067	0.796	0.983	0.068	0.860	1.123	0.275	Genotyped
rs117457252	9	6,159,193	C	T	0.034	0.032	0.436	1.075	0.093	0.896	1.290	0.454	Imputed
rs10975467	9	6,159,758	T	C	0.244	0.259	0.038	0.922	0.039	0.853	0.995	0.484	Imputed
rs7850598	9	6,159,759	G	A	0.383	0.393	0.214	0.958	0.035	0.895	1.025	0.570	Imputed
rs4637906	9	6,160,049	G	C	0.396	0.407	0.144	0.951	0.035	0.889	1.017	0.356	Imputed
rs2890697	9	6,160,249	T	A	0.398	0.408	0.193	0.956	0.035	0.893	1.023	0.407	Imputed
rs2225537	9	6,160,578	T	C	0.397	0.407	0.194	0.956	0.035	0.893	1.023	0.377	Imputed
rs2210462	9	6,160,648	T	C	0.397	0.407	0.198	0.956	0.035	0.894	1.023	0.371	Imputed
rs116905864	9	6,161,095	C	T	0.016	0.017	0.805	0.967	0.134	0.744	1.258	0.100	Imputed
rs6477048	9	6,161,253	T	C	0.066	0.066	0.802	0.983	0.068	0.860	1.124	0.318	Imputed
rs10124250	9	6,161,686	T	C	0.396	0.405	0.243	0.960	0.035	0.898	1.028	0.339	Genotyped
rs11794800	9	6,162,489	T	C	0.084	0.080	0.410	1.052	0.061	0.933	1.185	0.722	Imputed
rs13302008	9	6,162,881	C	T	0.084	0.081	0.482	1.044	0.061	0.926	1.177	0.820	Imputed
rs114323046	9	6,163,183	T	C	0.015	0.017	0.444	0.900	0.138	0.687	1.179	0.806	Imputed
rs72699183	9	6,163,764	A	G	0.239	0.251	0.100	0.937	0.040	0.867	1.013	0.424	Imputed
rs10119713	9	6,163,823	A	G	0.396	0.405	0.268	0.962	0.035	0.899	1.030	0.338	Genotyped
rs7863536	9	6,164,771	A	C	0.065	0.066	0.800	0.983	0.069	0.859	1.124	0.400	Genotyped
rs4367609	9	6,165,405	T	C	0.066	0.066	0.827	0.985	0.068	0.862	1.126	0.345	Imputed
rs2079	9	6,166,653	A	G	0.333	0.343	0.187	0.954	0.036	0.889	1.023	0.784	Genotyped
rs76396502	9	6,166,769	A	G	0.017	0.017	0.618	0.936	0.132	0.723	1.212	0.199	Imputed
rs4579584	9	6,166,919	C	T	0.085	0.086	0.913	0.993	0.061	0.882	1.119	0.755	Imputed
rs2381413	9	6,167,017	G	C	0.399	0.408	0.200	0.957	0.035	0.894	1.024	0.495	Imputed
rs12003769	9	6,168,335	C	T	0.066	0.066	0.829	0.985	0.068	0.862	1.126	0.346	Imputed
rs117248841	9	6,169,383	A	G	0.027	0.030	0.314	0.901	0.104	0.735	1.104	0.655	Imputed
chr9:6169508:D	9	6,169,508	A	AAT	0.065	0.066	0.587	0.963	0.069	0.842	1.102	0.242	Imputed
chr9:6169509:D	9	6,169,509	A	AT	0.065	0.066	0.619	0.966	0.069	0.845	1.106	0.268	Imputed
rs10118918	9	6,170,162	G	T	0.067	0.068	0.565	0.962	0.068	0.842	1.098	0.427	Genotyped
rs116863868	9	6,170,847	T	C	0.009	0.012	0.053	0.709	0.178	0.501	1.005	0.146	Imputed
rs17582360	9	6,170,914	C	T	0.020	0.020	0.788	1.033	0.121	0.815	1.310	0.698	Imputed
rs13293142	9	6,170,924	C	A	0.065	0.064	0.673	1.029	0.069	0.900	1.178	0.737	Imputed
rs72699184	9	6,171,224	T	C	0.029	0.029	0.831	0.979	0.102	0.802	1.194	0.112	Imputed
rs74879126	9	6,171,296	T	C	0.021	0.023	0.504	0.925	0.117	0.736	1.163	0.689	Imputed
rs13302749	9	6,171,731	C	T	0.067	0.066	0.925	1.006	0.068	0.881	1.149	0.527	Imputed
rs4142528	9	6,172,296	T	A	0.311	0.321	0.175	0.952	0.037	0.886	1.022	0.202	Imputed
rs7032572	9	6,172,380	G	A	0.151	0.164	0.046	0.910	0.047	0.830	0.998	0.180	Imputed
rs34282845	9	6,173,499	C	G	0.066	0.065	0.855	1.013	0.068	0.886	1.157	0.628	Imputed
rs10815362	9	6,173,798	T	C	0.088	0.088	0.995	1.000	0.060	0.889	1.124	0.503	Imputed
rs10815363	9	6,174,316	T	C	0.310	0.320	0.175	0.952	0.037	0.886	1.022	0.181	Imputed
rs72699185	9	6,175,522	T	G	0.047	0.054	0.039	0.848	0.080	0.726	0.992	0.040	Imputed

rs72699186	9	6,175,855	T	A	0.150	0.163	0.035	0.905	0.047	0.825	0.993	0.153	Imputed
rs117865403	9	6,175,872	T	A	0.019	0.020	0.575	0.933	0.124	0.731	1.190	0.600	Imputed
rs189451214	9	6,176,322	A	T	0.021	0.024	0.259	0.876	0.117	0.697	1.102	0.195	Imputed
rs78436571	9	6,176,534	C	T	0.059	0.061	0.525	0.955	0.072	0.830	1.099	0.291	Imputed
rs4742165	9	6,176,770	T	G	0.064	0.065	0.747	0.978	0.069	0.854	1.120	0.387	Genotyped
rs72699188	9	6,176,871	G	C	0.151	0.163	0.043	0.909	0.047	0.829	0.997	0.153	Imputed
rs67923960	9	6,177,208	A	T	0.067	0.066	0.937	1.005	0.068	0.880	1.148	0.533	Imputed
rs10815364	9	6,177,291	G	T	0.322	0.331	0.190	0.954	0.036	0.888	1.024	0.144	Imputed
rs10975468	9	6,177,302	T	C	0.150	0.163	0.037	0.906	0.047	0.826	0.994	0.149	Imputed
rs9777458	9	6,177,420	C	T	0.219	0.230	0.120	0.938	0.041	0.866	1.017	0.312	Imputed
rs9775039	9	6,177,453	A	G	0.152	0.164	0.056	0.914	0.047	0.834	1.002	0.138	Imputed
rs150513184	9	6,177,677	A	G	0.016	0.017	0.509	0.914	0.135	0.701	1.192	0.151	Imputed
rs144511606	9	6,178,525	A	G	0.150	0.163	0.038	0.907	0.047	0.827	0.995	0.163	Imputed
rs143479997	9	6,178,624	C	G	0.012	0.014	0.284	0.849	0.153	0.630	1.146	0.797	Imputed
rs182666245	9	6,178,999	T	G	0.025	0.022	0.358	1.106	0.110	0.892	1.372	0.473	Imputed
rs146719350	9	6,179,189	G	A	0.121	0.122	0.865	0.991	0.052	0.895	1.097	0.977	Imputed
rs7874783	9	6,180,636	T	C	0.078	0.079	0.760	0.981	0.063	0.867	1.110	0.155	Imputed
rs4097694	9	6,181,307	T	C	0.166	0.169	0.574	0.975	0.046	0.891	1.066	0.661	Imputed
rs4529511	9	6,181,525	T	C	0.113	0.123	0.053	0.902	0.053	0.812	1.001	0.052	Imputed
chr9:6181890:D	9	6,181,890	T	TC	0.067	0.067	0.901	1.008	0.068	0.883	1.151	0.602	Imputed
rs138460274	9	6,182,285	A	G	0.082	0.082	0.768	1.018	0.062	0.902	1.149	0.982	Imputed
rs181891991	9	6,182,467	A	T	0.012	0.012	0.847	0.970	0.156	0.714	1.318	0.850	Imputed
rs10975473	9	6,182,542	T	A	0.048	0.055	0.048	0.855	0.079	0.732	0.998	0.055	Imputed
rs10975474	9	6,182,582	T	G	0.048	0.055	0.057	0.860	0.079	0.737	1.004	0.025	Imputed
rs76862848	9	6,183,622	T	A	0.016	0.016	0.778	0.962	0.137	0.736	1.258	0.367	Imputed
rs2890704	9	6,184,165	T	C	0.088	0.088	0.873	0.990	0.060	0.881	1.114	0.544	Genotyped
rs12349858	9	6,185,295	T	C	0.156	0.169	0.034	0.906	0.047	0.827	0.992	0.181	Imputed
rs13291323	9	6,185,360	C	T	0.063	0.061	0.563	1.041	0.070	0.908	1.193	0.505	Genotyped
rs76962799	9	6,187,132	A	C	0.035	0.036	0.993	1.001	0.092	0.836	1.198	0.524	Imputed
rs13298301	9	6,187,242	G	A	0.067	0.066	0.980	1.002	0.068	0.877	1.144	0.459	Imputed
rs117455876	9	6,187,324	G	T	0.017	0.017	0.999	1.000	0.130	0.775	1.291	0.349	Imputed
rs13296741	9	6,187,395	T	G	0.067	0.066	0.968	1.003	0.068	0.878	1.145	0.475	Imputed
rs1929996	9	6,187,636	C	G	0.308	0.318	0.180	0.952	0.037	0.886	1.023	0.280	Imputed
rs10758748	9	6,187,862	T	C	0.086	0.087	0.905	0.993	0.060	0.882	1.117	0.579	Imputed
rs4742166	9	6,188,124	G	C	0.309	0.318	0.191	0.953	0.037	0.887	1.024	0.288	Imputed
rs1412426	9	6,188,652	A	C	0.309	0.318	0.213	0.956	0.037	0.889	1.026	0.250	Genotyped
rs1412425	9	6,188,740	A	C	0.307	0.315	0.269	0.960	0.037	0.894	1.032	0.302	Imputed
rs9299029	9	6,189,633	C	T	0.145	0.144	0.856	1.009	0.048	0.918	1.108	0.698	Imputed
rs1342327	9	6,189,874	G	C	0.154	0.153	0.989	1.001	0.047	0.913	1.097	0.948	Imputed
rs1342326	9	6,190,076	C	A	0.151	0.162	0.063	0.916	0.047	0.835	1.005	0.147	Imputed

rs10739087	9	6,191,105	A	C	0.087	0.087	0.931	0.995	0.060	0.884	1.119	0.531	Imputed
rs10739088	9	6,191,110	T	C	0.087	0.087	0.931	0.995	0.060	0.884	1.119	0.531	Imputed
rs116018425	9	6,191,508	A	G	0.012	0.014	0.482	0.898	0.154	0.664	1.213	0.608	Imputed
rs77773750	9	6,192,209	T	C	0.039	0.039	0.976	1.003	0.087	0.846	1.189	0.730	Imputed
rs148990158	9	6,192,650	A	C	0.022	0.023	0.726	0.960	0.117	0.764	1.206	0.153	Imputed
rs2095044	9	6,192,796	T	C	0.241	0.251	0.151	0.945	0.040	0.875	1.021	0.116	Imputed
rs2210465	9	6,193,022	T	C	0.087	0.087	1.000	1.000	0.060	0.889	1.125	0.584	Imputed
rs2381416	9	6,193,455	C	A	0.244	0.257	0.084	0.934	0.039	0.865	1.009	0.147	Imputed
rs10815370	9	6,194,831	C	A	0.307	0.318	0.157	0.950	0.037	0.884	1.020	0.331	Imputed
rs4742167	9	6,195,285	C	T	0.307	0.318	0.162	0.950	0.037	0.884	1.021	0.322	Imputed
rs7033058	9	6,196,645	T	C	0.308	0.319	0.158	0.950	0.037	0.884	1.020	0.255	Imputed
rs10975479	9	6,197,377	G	A	0.149	0.162	0.037	0.906	0.047	0.826	0.994	0.142	Imputed
rs1888909	9	6,197,392	T	C	0.237	0.251	0.056	0.927	0.040	0.857	1.002	0.085	Imputed
rs13285109	9	6,197,408	G	A	0.067	0.067	0.847	1.013	0.067	0.888	1.156	0.437	Imputed
rs113690127	9	6,197,547	A	G	0.023	0.026	0.329	0.896	0.112	0.719	1.117	0.205	Imputed
rs138714180	9	6,197,753	G	C	0.009	0.010	0.320	0.836	0.180	0.587	1.190	0.885	Imputed
rs77632918	9	6,198,384	G	A	0.025	0.028	0.165	0.861	0.108	0.696	1.064	0.236	Imputed
rs28551499	9	6,198,578	C	A	0.051	0.061	0.014	0.829	0.076	0.714	0.962	0.036	Imputed
rs144925331	9	6,199,285	A	G	0.022	0.022	0.725	0.960	0.117	0.764	1.206	0.193	Imputed
rs928411	9	6,199,492	A	T	0.309	0.320	0.145	0.948	0.037	0.883	1.019	0.238	Imputed
rs928410	9	6,199,536	T	C	0.087	0.087	0.815	0.986	0.060	0.876	1.109	0.498	Imputed
rs66887324	9	6,200,562	T	G	0.063	0.061	0.581	1.039	0.070	0.906	1.192	0.442	Imputed
rs13301448	9	6,201,001	C	T	0.064	0.061	0.443	1.055	0.069	0.921	1.208	0.433	Imputed
rs10046872	9	6,201,136	G	C	0.087	0.088	0.848	0.989	0.060	0.879	1.112	0.576	Imputed
rs1929995	9	6,201,163	C	T	0.154	0.168	0.022	0.898	0.047	0.820	0.984	0.144	Imputed
rs2150970	9	6,201,364	G	A	0.088	0.088	0.926	0.995	0.060	0.885	1.118	0.539	Genotyped
rs1475658	9	6,201,574	T	A	0.314	0.328	0.077	0.938	0.036	0.873	1.007	0.152	Imputed
rs77121712	9	6,201,575	T	A	0.209	0.218	0.154	0.943	0.042	0.869	1.023	0.380	Imputed
rs13302358	9	6,202,170	G	C	0.064	0.061	0.486	1.049	0.069	0.916	1.202	0.360	Imputed
rs13284060	9	6,202,701	C	A	0.063	0.061	0.575	1.040	0.070	0.907	1.193	0.439	Imputed
rs139141884	9	6,204,435	A	T	0.022	0.022	0.734	0.961	0.117	0.765	1.208	0.196	Imputed
rs10975481	9	6,204,469	G	A	0.422	0.435	0.117	0.948	0.034	0.886	1.014	0.369	Imputed
rs117793869	9	6,204,491	T	A	0.017	0.017	0.946	0.991	0.130	0.768	1.279	0.366	Imputed
rs4366128	9	6,205,394	T	C	0.096	0.098	0.577	0.969	0.058	0.865	1.084	0.565	Imputed
rs144829310	9	6,208,030	T	G	0.150	0.163	0.039	0.907	0.047	0.827	0.995	0.128	Imputed
rs11794953	9	6,208,855	G	A	0.014	0.014	0.910	1.016	0.142	0.770	1.342	0.315	Imputed
rs117239796	9	6,208,893	C	T	0.014	0.016	0.355	0.876	0.143	0.662	1.159	0.387	Imputed
rs7046661	9	6,209,199	C	G	0.312	0.322	0.174	0.952	0.036	0.886	1.022	0.181	Imputed
rs992969	9	6,209,697	A	G	0.236	0.249	0.060	0.928	0.040	0.858	1.003	0.092	Genotyped
rs13283694	9	6,209,755	G	A	0.067	0.067	0.895	1.009	0.068	0.884	1.152	0.436	Imputed

rs3939286	9	6,210,099	T	C	0.239	0.251	0.092	0.936	0.040	0.866	1.011	0.102	Genotyped
rs34110711	9	6,210,107	C	A	0.067	0.066	0.883	1.010	0.068	0.885	1.153	0.431	Imputed
rs72699191	9	6,211,813	C	T	0.155	0.169	0.023	0.899	0.047	0.821	0.985	0.149	Imputed
chr9:6212208:D	9	6,212,208	A	AC	0.019	0.021	0.426	0.906	0.123	0.712	1.154	0.239	Imputed
rs928412	9	6,213,148	A	G	0.240	0.256	0.029	0.917	0.040	0.849	0.991	0.117	Imputed
rs928413	9	6,213,387	G	A	0.244	0.258	0.039	0.922	0.039	0.854	0.996	0.102	Imputed
rs7848215	9	6,213,468	T	C	0.245	0.260	0.042	0.923	0.039	0.855	0.997	0.060	Genotyped
rs10815374	9	6,213,705	A	G	0.081	0.084	0.476	0.957	0.062	0.847	1.080	0.471	Imputed
rs142807069	9	6,213,820	G	A	0.142	0.151	0.138	0.931	0.048	0.846	1.023	0.138	Imputed
rs10975488	9	6,213,829	G	A	0.139	0.146	0.202	0.939	0.049	0.853	1.034	0.302	Imputed
rs186440475	9	6,213,849	A	C	0.023	0.019	0.101	1.208	0.115	0.964	1.515	0.740	Imputed
rs35012226	9	6,213,853	G	A	0.067	0.073	0.185	0.915	0.067	0.801	1.044	0.239	Imputed
rs10975489	9	6,213,864	C	T	0.156	0.158	0.790	0.988	0.047	0.901	1.083	0.937	Imputed
rs140684613	9	6,213,915	G	A	0.022	0.022	0.873	0.981	0.117	0.781	1.233	0.219	Imputed
rs183337634	9	6,213,985	T	C	0.020	0.020	0.747	0.962	0.121	0.758	1.219	0.964	Imputed
rs149023172	9	6,214,036	A	G	0.032	0.036	0.159	0.873	0.096	0.723	1.054	0.606	Imputed
rs34077494	9	6,214,050	G	T	0.025	0.023	0.279	1.125	0.109	0.909	1.392	0.484	Imputed
rs62559598	9	6,214,347	C	T	0.159	0.164	0.429	0.964	0.046	0.881	1.055	0.350	Imputed
chr9:6216119:D	9	6,216,119	C	CTTTAT	0.075	0.075	0.936	0.995	0.064	0.878	1.128	0.332	Imputed
rs72614079	9	6,216,138	G	A	0.096	0.091	0.315	1.060	0.058	0.946	1.186	0.208	Imputed
rs1157505	9	6,216,240	G	C	0.233	0.210	7.60E-04	1.145	0.040	1.058	1.239	0.737	Imputed
rs11791561	9	6,216,516	G	C	0.508	0.483	1.76E-03	1.112	0.034	1.040	1.188	0.343	Imputed
rs142929873	9	6,216,663	A	G	0.018	0.015	0.110	1.228	0.129	0.954	1.581	0.683	Imputed
rs111896044	9	6,216,815	A	G	0.079	0.083	0.409	0.950	0.063	0.840	1.074	0.784	Imputed
rs78121143	9	6,218,346	A	G	0.014	0.016	0.188	0.827	0.144	0.624	1.096	0.239	Imputed
rs72614080	9	6,218,595	T	C	0.092	0.089	0.459	1.044	0.059	0.931	1.171	0.303	Imputed
chr9:6218684:D	9	6,218,684	A	ATGT	0.122	0.134	0.020	0.887	0.052	0.801	0.981	0.346	Imputed
rs2066361	9	6,218,960	A	T	0.220	0.232	0.096	0.934	0.041	0.863	1.012	0.254	Imputed
rs2066362	9	6,219,176	T	G	0.145	0.158	0.025	0.898	0.048	0.817	0.986	0.028	Genotyped
rs1891385	9	6,219,845	C	A	0.109	0.102	0.120	1.088	0.054	0.978	1.211	0.194	Imputed
rs73396601	9	6,221,162	G	C	0.029	0.031	0.405	0.920	0.100	0.756	1.120	0.497	Imputed
rs16924144	9	6,221,246	C	T	0.358	0.340	0.020	1.086	0.035	1.013	1.163	0.759	Genotyped
rs11794419	9	6,222,110	C	T	0.030	0.032	0.471	0.932	0.098	0.768	1.130	0.608	Genotyped
rs10815376	9	6,222,149	T	C	0.301	0.319	0.017	0.916	0.037	0.852	0.984	0.205	Imputed
rs996029	9	6,222,302	T	G	0.046	0.043	0.317	1.085	0.081	0.925	1.272	0.537	Genotyped
rs11787939	9	6,222,553	A	G	0.029	0.032	0.368	0.915	0.099	0.753	1.111	0.582	Imputed
rs118148121	9	6,223,837	A	T	0.038	0.038	0.979	0.998	0.089	0.838	1.188	0.123	Imputed
rs2210464	9	6,223,903	T	A	0.211	0.223	0.083	0.931	0.041	0.858	1.009	0.751	Imputed
rs1929994	9	6,224,308	C	T	0.029	0.031	0.416	0.922	0.100	0.757	1.122	0.518	Imputed
rs10815377	9	6,224,971	G	C	0.243	0.258	0.037	0.921	0.039	0.853	0.995	0.897	Imputed

rs10815378	9	6,224,977	C	T	0.243	0.258	0.037	0.921	0.039	0.853	0.995	0.897	Imputed
rs10435816	9	6,225,535	G	A	0.245	0.262	0.023	0.914	0.039	0.847	0.988	0.536	Genotyped
rs7048482	9	6,225,659	A	G	0.030	0.030	0.999	1.000	0.100	0.823	1.216	0.156	Imputed
rs10815379	9	6,225,825	A	C	0.210	0.223	0.074	0.929	0.041	0.856	1.007	0.792	Imputed
rs10815380	9	6,226,086	T	C	0.213	0.226	0.065	0.927	0.041	0.855	1.005	0.782	Imputed
rs1418386	9	6,226,207	T	C	0.243	0.258	0.039	0.922	0.039	0.853	0.996	0.904	Imputed
rs10815381	9	6,226,289	G	A	0.213	0.226	0.065	0.927	0.041	0.855	1.005	0.783	Imputed
rs1418385	9	6,226,295	T	C	0.243	0.258	0.038	0.922	0.039	0.853	0.996	0.903	Imputed
rs10975497	9	6,226,592	T	C	0.242	0.258	0.035	0.920	0.039	0.852	0.994	0.869	Imputed
rs10975498	9	6,226,688	C	T	0.243	0.258	0.041	0.923	0.039	0.854	0.997	0.897	Imputed
rs2006682	9	6,227,045	G	C	0.277	0.293	0.027	0.920	0.038	0.854	0.991	0.183	Imputed
rs118018163	9	6,227,111	A	G	0.016	0.019	0.083	0.792	0.135	0.608	1.031	0.772	Imputed
rs10118776	9	6,227,418	G	A	0.069	0.065	0.377	1.061	0.067	0.930	1.210	0.291	Imputed
rs2210463	9	6,227,752	G	A	0.242	0.257	0.046	0.924	0.039	0.856	0.998	0.839	Genotyped
rs914602	9	6,228,228	T	C	0.063	0.059	0.267	1.080	0.070	0.942	1.238	0.424	Imputed
rs2026990	9	6,228,694	G	A	0.242	0.257	0.045	0.924	0.039	0.856	0.998	0.838	Imputed
chr9:6228906:I	9	6,228,906	CA	C	0.485	0.455	2.54E-04	1.132	0.034	1.059	1.210	0.139	Imputed
rs4740840	9	6,229,110	G	A	0.242	0.257	0.039	0.922	0.039	0.853	0.996	0.795	Imputed
rs16924159	9	6,229,417	A	G	0.345	0.329	0.043	1.075	0.036	1.002	1.152	0.559	Genotyped
rs60076497	9	6,229,661	G	T	0.029	0.031	0.468	0.930	0.100	0.765	1.131	0.581	Imputed
rs56713080	9	6,229,662	C	A	0.029	0.031	0.468	0.930	0.100	0.765	1.131	0.581	Imputed
rs10975499	9	6,230,072	A	G	0.213	0.226	0.067	0.927	0.041	0.855	1.005	0.699	Imputed
rs10758750	9	6,230,513	G	C	0.240	0.255	0.047	0.925	0.040	0.856	0.999	0.911	Imputed
rs10815383	9	6,230,670	G	C	0.240	0.255	0.047	0.925	0.040	0.856	0.999	0.911	Imputed
rs16924161	9	6,230,912	C	T	0.159	0.169	0.128	0.932	0.046	0.852	1.020	0.824	Genotyped
rs73398519	9	6,230,947	G	A	0.028	0.029	0.648	0.954	0.103	0.780	1.167	0.554	Imputed
rs12551256	9	6,231,239	G	A	0.491	0.459	1.30E-04	1.138	0.034	1.065	1.216	0.192	Genotyped
rs12551268	9	6,231,318	A	C	0.269	0.287	0.019	0.914	0.038	0.849	0.985	0.178	Imputed
rs10815388	9	6,232,242	C	T	0.305	0.315	0.213	0.955	0.037	0.889	1.027	0.840	Genotyped
rs12351913	9	6,232,703	C	G	0.303	0.313	0.181	0.952	0.037	0.886	1.023	0.725	Imputed
rs12683567	9	6,232,985	T	C	0.303	0.313	0.203	0.954	0.037	0.888	1.026	0.804	Imputed
rs12339348	9	6,233,082	T	A	0.204	0.227	8.91E-04	0.870	0.042	0.802	0.945	0.057	Imputed
rs10975501	9	6,233,221	A	C	0.065	0.060	0.183	1.096	0.069	0.958	1.255	0.630	Genotyped
rs16924171	9	6,233,279	A	T	0.303	0.313	0.193	0.953	0.037	0.887	1.025	0.763	Imputed
rs17582919	9	6,233,376	C	T	0.206	0.227	1.42E-03	0.875	0.042	0.807	0.950	0.090	Imputed
rs61151237	9	6,233,790	G	C	0.028	0.029	0.663	0.956	0.103	0.782	1.169	0.546	Imputed
rs7022391	9	6,234,125	T	C	0.064	0.059	0.205	1.092	0.069	0.953	1.250	0.626	Imputed
rs7033258	9	6,234,131	G	A	0.303	0.313	0.192	0.953	0.037	0.887	1.024	0.750	Imputed
rs58924681	9	6,234,332	C	T	0.027	0.029	0.599	0.947	0.103	0.774	1.159	0.580	Imputed
rs7034720	9	6,234,546	C	A	0.303	0.313	0.192	0.953	0.037	0.887	1.024	0.750	Imputed

chr9:6234761:I	9	6,234,761	T	TC	0.276	0.285	0.244	0.957	0.038	0.889	1.031	0.914	Imputed
rs10975504	9	6,235,009	G	A	0.206	0.228	1.03E-03	0.872	0.042	0.804	0.946	0.044	Imputed
rs12341955	9	6,235,217	T	A	0.490	0.458	7.94E-05	1.143	0.034	1.069	1.221	0.210	Imputed
rs10815390	9	6,235,343	C	T	0.303	0.313	0.191	0.953	0.037	0.887	1.024	0.752	Imputed
rs1375	9	6,235,753	G	T	0.311	0.321	0.199	0.954	0.037	0.888	1.025	0.767	Imputed
rs928414	9	6,236,350	G	A	0.311	0.321	0.198	0.954	0.037	0.888	1.025	0.768	Imputed
rs55645596	9	6,236,407	G	T	0.153	0.159	0.341	0.956	0.047	0.872	1.048	0.369	Imputed
rs4237163	9	6,236,496	A	T	0.484	0.451	9.49E-05	1.141	0.034	1.068	1.220	0.213	Imputed
rs4237164	9	6,236,501	G	T	0.484	0.451	9.49E-05	1.141	0.034	1.068	1.220	0.213	Imputed
rs112935616	9	6,236,830	T	C	0.193	0.212	4.53E-03	0.886	0.043	0.815	0.963	0.026	Imputed
rs10975507	9	6,236,977	T	A	0.205	0.227	1.25E-03	0.874	0.042	0.805	0.948	0.063	Imputed
rs17498168	9	6,237,186	C	T	0.017	0.020	0.165	0.835	0.130	0.647	1.078	0.851	Imputed
rs10975509	9	6,237,263	G	A	0.283	0.292	0.259	0.959	0.038	0.891	1.032	0.925	Imputed
rs143387549	9	6,237,360	A	G	0.016	0.016	0.958	1.007	0.134	0.774	1.311	0.718	Imputed
rs17498196	9	6,237,547	C	A	0.203	0.226	8.06E-04	0.869	0.042	0.801	0.943	0.072	Imputed
rs116947965	9	6,238,629	A	G	0.025	0.026	0.632	0.950	0.107	0.770	1.173	0.572	Imputed
rs72689561	9	6,238,750	C	A	0.203	0.226	8.18E-04	0.869	0.042	0.801	0.944	0.072	Imputed
rs7025417	9	6,240,084	C	T	0.212	0.226	0.057	0.925	0.041	0.853	1.002	0.671	Imputed
rs10815391	9	6,240,235	G	T	0.194	0.210	0.011	0.897	0.043	0.825	0.975	0.023	Imputed
rs10815392	9	6,240,236	C	T	0.196	0.214	6.32E-03	0.890	0.043	0.819	0.968	0.021	Imputed
rs10815393	9	6,240,324	C	T	0.205	0.227	1.20E-03	0.874	0.042	0.805	0.948	0.062	Imputed
rs10118795	9	6,240,658	T	C	0.303	0.313	0.187	0.953	0.037	0.886	1.024	0.728	Imputed
rs79890344	9	6,240,684	T	C	0.023	0.025	0.233	0.874	0.113	0.700	1.091	0.825	Imputed
rs72689565	9	6,240,953	C	A	0.206	0.228	8.85E-04	0.870	0.042	0.802	0.945	0.059	Imputed
rs4742170	9	6,242,950	C	T	0.312	0.323	0.183	0.953	0.036	0.887	1.023	0.882	Genotyped
rs7035413	9	6,243,119	G	A	0.204	0.226	7.85E-04	0.869	0.042	0.801	0.943	0.063	Imputed
rs7038893	9	6,243,392	C	T	0.203	0.226	8.44E-04	0.870	0.042	0.801	0.944	0.075	Imputed
rs73398539	9	6,243,645	A	G	0.027	0.029	0.562	0.942	0.103	0.770	1.153	0.534	Imputed
rs10975512	9	6,243,819	C	T	0.215	0.229	0.048	0.922	0.041	0.851	0.999	0.565	Imputed
rs7019575	9	6,243,935	C	G	0.416	0.453	4.98E-06	0.855	0.034	0.800	0.915	0.067	Imputed
chr9:6245140:D	9	6,245,140	A	AT	0.013	0.012	0.769	1.046	0.152	0.776	1.409	0.909	Imputed
rs72689566	9	6,245,393	C	G	0.077	0.080	0.649	0.972	0.063	0.858	1.100	0.715	Imputed
rs113372176	9	6,245,931	T	C	0.027	0.027	0.778	0.971	0.105	0.791	1.192	0.562	Imputed
rs112175146	9	6,245,971	A	G	0.050	0.053	0.448	0.943	0.077	0.811	1.097	0.351	Imputed
rs10975514	9	6,246,144	A	G	0.335	0.301	1.05E-05	1.172	0.036	1.092	1.257	0.457	Imputed
rs111841620	9	6,246,225	G	C	0.016	0.017	0.637	0.938	0.135	0.720	1.223	0.578	Imputed
rs7849201	9	6,246,256	C	T	0.335	0.301	1.05E-05	1.172	0.036	1.092	1.257	0.457	Imputed
rs10975515	9	6,247,117	A	G	0.406	0.369	3.25E-06	1.174	0.035	1.097	1.256	0.244	Imputed
rs7037276	9	6,247,430	C	T	0.073	0.070	0.469	1.048	0.065	0.923	1.191	0.330	Genotyped
rs11792139	9	6,247,551	T	C	0.307	0.273	4.40E-06	1.184	0.037	1.102	1.273	0.552	Imputed

rs10975516	9	6,247,693	A	G	0.336	0.302	1.28E-05	1.170	0.036	1.090	1.255	0.398	Genotyped
rs78100995	9	6,248,007	G	C	0.202	0.175	2.43E-05	1.196	0.042	1.101	1.300	0.870	Imputed
rs11792633	9	6,248,035	T	C	0.335	0.301	1.37E-05	1.169	0.036	1.090	1.255	0.464	Imputed
chr9:6248244:I	9	6,248,244	TC	T	0.018	0.020	0.338	0.884	0.128	0.688	1.137	0.207	Imputed
rs76864631	9	6,248,408	A	G	0.209	0.182	2.80E-05	1.192	0.042	1.098	1.294	0.930	Imputed
rs10975517	9	6,248,457	T	C	0.012	0.014	0.360	0.868	0.155	0.640	1.176	0.583	Imputed
rs1317230	9	6,251,012	A	C	0.305	0.271	3.62E-06	1.186	0.037	1.103	1.275	0.537	Imputed
rs1854709	9	6,251,455	T	C	0.055	0.052	0.330	1.075	0.074	0.929	1.243	0.430	Imputed
rs1330383	9	6,251,507	T	G	0.332	0.299	1.02E-05	1.172	0.036	1.092	1.258	0.491	Genotyped
rs1929992	9	6,251,588	C	T	0.332	0.299	1.27E-05	1.170	0.036	1.090	1.256	0.453	Imputed
rs1330382	9	6,251,986	A	G	0.010	0.011	0.550	0.905	0.168	0.651	1.257	0.850	Imputed
chr9:6252065:D	9	6,252,065	A	ACCC	0.231	0.207	6.46E-04	1.149	0.041	1.061	1.244	0.302	Imputed
chr9:6252330:D	9	6,252,330	TAA	T	0.395	0.358	5.02E-06	1.171	0.035	1.094	1.254	0.224	Imputed
rs73398552	9	6,252,689	C	T	0.027	0.028	0.704	0.961	0.105	0.783	1.180	0.564	Imputed
rs10815394	9	6,253,297	T	C	0.332	0.299	1.29E-05	1.170	0.036	1.090	1.256	0.454	Imputed
rs1113573	9	6,253,301	C	T	0.339	0.306	2.31E-05	1.164	0.036	1.085	1.249	0.359	Imputed
rs10975519	9	6,253,571	T	C	0.334	0.300	1.04E-05	1.172	0.036	1.092	1.258	0.389	Genotyped
rs10975520	9	6,253,710	C	G	0.332	0.299	1.27E-05	1.170	0.036	1.090	1.256	0.453	Imputed
rs7044343	9	6,254,208	C	T	0.395	0.359	6.02E-06	1.170	0.035	1.093	1.252	0.239	Imputed
chr9:6254567:D	9	6,254,567	A	AT	0.326	0.293	1.46E-05	1.170	0.036	1.090	1.256	0.393	Imputed
rs75228791	9	6,254,568	A	T	0.321	0.289	1.29E-05	1.172	0.036	1.091	1.258	0.400	Imputed
rs7857724	9	6,254,694	A	C	0.389	0.352	2.95E-06	1.176	0.035	1.099	1.260	0.306	Imputed
rs150599805	9	6,254,738	C	G	0.018	0.019	0.545	0.926	0.128	0.721	1.188	0.531	Imputed
rs116996596	9	6,254,860	C	A	0.025	0.026	0.456	0.923	0.108	0.746	1.141	0.606	Imputed
rs7871381	9	6,254,900	G	A	0.390	0.352	2.05E-06	1.179	0.035	1.102	1.263	0.301	Imputed
rs1412421	9	6,255,010	A	C	0.390	0.352	2.10E-06	1.179	0.035	1.102	1.262	0.299	Imputed
rs1412420	9	6,255,152	A	G	0.054	0.052	0.432	1.060	0.075	0.916	1.228	0.423	Imputed
rs7047921	9	6,255,319	A	G	0.334	0.299	6.57E-06	1.176	0.036	1.096	1.262	0.437	Imputed
rs12336076	9	6,255,789	C	A	0.334	0.299	6.57E-06	1.176	0.036	1.096	1.262	0.437	Imputed
rs1332290	9	6,255,881	T	G	0.389	0.352	2.74E-06	1.177	0.035	1.099	1.260	0.275	Genotyped
rs1048274	9	6,256,292	A	G	0.333	0.299	8.78E-06	1.174	0.036	1.094	1.259	0.428	Genotyped
rs1048279	9	6,256,529	A	C	0.054	0.051	0.467	1.056	0.075	0.912	1.223	0.518	Imputed
rs55726619	9	6,256,678	C	T	0.027	0.028	0.712	0.962	0.105	0.784	1.181	0.560	Imputed
rs16924243	9	6,257,054	C	T	0.027	0.028	0.712	0.962	0.104	0.786	1.179	0.661	Genotyped
rs12000491	9	6,257,367	C	T	0.027	0.028	0.704	0.961	0.104	0.785	1.178	0.665	Genotyped
rs73398574	9	6,257,724	G	A	0.027	0.028	0.704	0.961	0.104	0.785	1.178	0.665	Imputed
rs8172	9	6,257,898	A	G	0.083	0.081	0.574	1.035	0.061	0.918	1.167	0.440	Imputed
chr9:6257919:D	9	6,257,919	T	TTAAA	0.024	0.026	0.434	0.917	0.110	0.739	1.139	0.455	Imputed
chr9:6257936:D	9	6,257,936	TCTA	T	0.083	0.081	0.583	1.034	0.061	0.917	1.166	0.445	Imputed
chr9:6257940:D	9	6,257,940	CT	C	0.130	0.135	0.379	0.957	0.050	0.867	1.056	0.201	Imputed

rs149510692	9	6,258,846	C	A	0.027	0.028	0.704	0.961	0.104	0.785	1.178	0.665	Imputed
rs7020305	9	6,258,932	T	C	0.060	0.057	0.354	1.068	0.071	0.929	1.228	0.438	Imputed
rs182703486	9	6,259,502	T	C	0.027	0.028	0.704	0.961	0.104	0.785	1.178	0.665	Imputed
rs4352903	9	6,259,655	T	C	0.086	0.086	0.932	0.995	0.060	0.884	1.120	0.309	Imputed
rs4369048	9	6,259,977	G	T	0.060	0.059	0.681	1.030	0.071	0.895	1.184	0.378	Imputed
rs7863253	9	6,260,249	T	C	0.100	0.104	0.480	0.961	0.056	0.860	1.073	0.253	Imputed
rs76528640	9	6,260,411	A	G	0.115	0.117	0.735	0.982	0.053	0.885	1.090	0.341	Imputed
rs150138020	9	6,260,496	C	G	0.304	0.270	3.04E-06	1.188	0.037	1.105	1.277	0.519	Imputed
rs7036484	9	6,260,683	C	T	0.027	0.028	0.704	0.961	0.104	0.785	1.178	0.665	Imputed
rs2150964	9	6,261,153	A	G	0.055	0.053	0.564	1.044	0.074	0.903	1.207	0.536	Imputed
rs4008353	9	6,261,695	C	T	0.158	0.161	0.738	0.985	0.047	0.898	1.079	0.771	Imputed
rs140384822	9	6,261,899	A	G	0.024	0.024	0.941	0.992	0.112	0.797	1.234	0.737	Imputed
rs56026930	9	6,262,338	A	G	0.037	0.041	0.174	0.887	0.089	0.745	1.055	0.266	Imputed
rs7025665	9	6,262,458	C	G	0.091	0.095	0.426	0.954	0.059	0.850	1.071	0.335	Imputed
rs189201498	9	6,263,240	T	C	0.011	0.014	0.077	0.758	0.158	0.556	1.033	0.109	Imputed
rs10733524	9	6,263,559	C	G	0.110	0.111	0.800	0.986	0.054	0.887	1.097	0.236	Imputed
rs10733525	9	6,263,560	T	A	0.110	0.111	0.800	0.986	0.054	0.887	1.097	0.236	Imputed
rs2183916	9	6,263,871	G	A	0.027	0.028	0.744	0.967	0.104	0.789	1.185	0.644	Imputed
rs2150972	9	6,264,055	C	T	0.028	0.030	0.471	0.930	0.101	0.763	1.134	0.489	Imputed
rs2150971	9	6,264,168	T	C	0.054	0.051	0.379	1.068	0.075	0.922	1.237	0.432	Imputed
rs57572183	9	6,264,410	G	A	0.027	0.028	0.769	0.970	0.104	0.792	1.189	0.631	Imputed
rs2051003	9	6,264,432	T	C	0.054	0.051	0.379	1.068	0.075	0.922	1.237	0.432	Imputed
rs73398588	9	6,264,741	G	C	0.027	0.028	0.744	0.967	0.104	0.789	1.185	0.644	Imputed
rs2381417	9	6,264,768	G	T	0.061	0.056	0.165	1.103	0.071	0.960	1.268	0.733	Imputed
rs10815397	9	6,265,256	G	C	0.222	0.229	0.333	0.961	0.041	0.888	1.041	0.387	Imputed
rs6477060	9	6,265,305	T	A	0.054	0.051	0.379	1.068	0.075	0.922	1.237	0.432	Imputed
rs1855022	9	6,265,967	T	A	0.054	0.051	0.412	1.063	0.075	0.918	1.232	0.450	Imputed
rs1855021	9	6,266,037	C	T	0.028	0.029	0.754	0.968	0.102	0.792	1.184	0.596	Imputed
chr9:6266169:I	9	6,266,169	G	GAACT	0.114	0.113	0.921	1.005	0.053	0.906	1.116	0.221	Imputed
rs7467297	9	6,266,244	G	A	0.027	0.028	0.752	0.968	0.104	0.790	1.186	0.639	Imputed
rs2026991	9	6,266,440	A	G	0.083	0.081	0.615	1.031	0.061	0.915	1.163	0.440	Genotyped
rs113897148	9	6,266,871	T	C	0.013	0.014	0.579	0.920	0.150	0.686	1.234	0.545	Imputed
rs73398594	9	6,266,958	A	T	0.027	0.027	0.737	0.966	0.105	0.787	1.185	0.548	Imputed
chr9:6267518:I	9	6,267,518	GTA	G	0.020	0.021	0.688	0.952	0.121	0.751	1.208	0.239	Imputed
chr9:6267538:I	9	6,267,538	ATC	A	0.027	0.028	0.814	0.976	0.103	0.797	1.195	0.748	Imputed
chr9:6267558:D	9	6,267,558	A	ATC	0.087	0.078	0.046	1.129	0.061	1.002	1.272	0.938	Imputed
rs7047769	9	6,267,718	T	C	0.027	0.028	0.752	0.968	0.104	0.790	1.186	0.639	Imputed
rs2039386	9	6,268,204	G	C	0.083	0.080	0.529	1.039	0.061	0.922	1.172	0.373	Imputed
rs2000198	9	6,268,569	T	C	0.054	0.051	0.452	1.058	0.075	0.913	1.226	0.470	Imputed
rs141794281	9	6,268,707	C	T	0.021	0.016	0.026	1.306	0.121	1.031	1.654	0.550	Imputed

rs2000199	9	6,268,893	G	A	0.083	0.081	0.559	1.036	0.061	0.919	1.169	0.453	Imputed
rs78261624	9	6,268,906	A	T	0.008	0.011	0.042	0.687	0.185	0.478	0.987	0.418	Imputed
rs980850	9	6,269,458	G	C	0.054	0.051	0.452	1.058	0.075	0.913	1.226	0.470	Imputed
rs149744716	9	6,269,581	C	T	0.011	0.011	0.889	1.023	0.163	0.744	1.406	0.334	Imputed
rs980849	9	6,269,689	T	C	0.054	0.051	0.398	1.066	0.075	0.920	1.235	0.465	Imputed
rs73398601	9	6,270,094	C	A	0.027	0.028	0.785	0.972	0.104	0.793	1.191	0.622	Imputed
rs1041538	9	6,270,359	A	G	0.083	0.080	0.555	1.037	0.061	0.919	1.169	0.451	Imputed
rs1041537	9	6,271,238	A	G	0.083	0.080	0.555	1.037	0.061	0.919	1.169	0.451	Imputed
rs10815398	9	6,272,766	C	A	0.393	0.357	7.28E-06	1.168	0.035	1.091	1.251	0.208	Genotyped
rs4740841	9	6,273,645	C	T	0.083	0.081	0.564	1.036	0.061	0.919	1.168	0.455	Imputed
rs10122305	9	6,274,892	T	C	0.058	0.054	0.284	1.081	0.073	0.937	1.247	0.479	Imputed
rs10125053	9	6,275,018	G	C	0.054	0.051	0.398	1.066	0.075	0.920	1.235	0.465	Imputed
rs10125090	9	6,275,119	G	A	0.054	0.051	0.398	1.066	0.075	0.920	1.235	0.465	Imputed
rs10123059	9	6,275,456	T	C	0.083	0.081	0.564	1.036	0.061	0.919	1.168	0.455	Imputed
rs10123132	9	6,275,720	T	C	0.053	0.051	0.437	1.060	0.075	0.915	1.229	0.487	Imputed
rs73400509	9	6,276,216	T	C	0.027	0.028	0.585	0.945	0.104	0.771	1.158	0.733	Imputed
rs34649179	9	6,276,378	T	C	0.054	0.052	0.553	1.045	0.075	0.903	1.210	0.554	Imputed
chr9:6276380:D	9	6,276,380	TCA	T	0.130	0.130	0.815	1.012	0.051	0.916	1.118	0.717	Imputed
rs35950099	9	6,276,381	C	T	0.067	0.063	0.302	1.073	0.068	0.939	1.226	0.797	Imputed
rs35319333	9	6,276,383	T	C	0.082	0.083	0.890	0.992	0.062	0.879	1.119	0.627	Imputed
rs4742172	9	6,276,733	C	T	0.054	0.051	0.384	1.067	0.075	0.922	1.236	0.471	Imputed
rs4742173	9	6,277,200	C	G	0.082	0.080	0.597	1.033	0.062	0.916	1.166	0.370	Imputed
rs7863442	9	6,277,317	G	A	0.083	0.081	0.527	1.039	0.061	0.922	1.172	0.438	Imputed
rs7853322	9	6,277,389	T	C	0.053	0.051	0.453	1.058	0.075	0.913	1.227	0.470	Imputed
rs62568088	9	6,277,534	G	A	0.153	0.159	0.379	0.960	0.047	0.876	1.052	0.296	Imputed
rs10758754	9	6,277,740	G	A	0.082	0.080	0.609	1.032	0.062	0.915	1.164	0.345	Imputed
rs1330381	9	6,277,820	A	G	0.053	0.051	0.453	1.058	0.075	0.913	1.227	0.470	Imputed
rs2169284	9	6,278,071	A	G	0.082	0.080	0.609	1.032	0.062	0.915	1.164	0.345	Imputed
chr9:6279424:I	9	6,279,424	GT	G	0.016	0.018	0.390	0.891	0.134	0.685	1.158	0.095	Imputed
rs11560535	9	6,279,768	A	G	0.083	0.080	0.617	1.031	0.061	0.914	1.163	0.348	Imputed
rs10758755	9	6,279,987	T	C	0.082	0.079	0.584	1.034	0.062	0.917	1.167	0.413	Imputed
rs147209310	9	6,280,227	A	C	0.014	0.019	0.021	0.720	0.143	0.545	0.952	0.350	Imputed
rs183823454	9	6,280,246	G	A	0.012	0.011	0.909	0.982	0.158	0.721	1.338	0.671	Imputed
rs2169285	9	6,280,786	A	G	0.083	0.080	0.594	1.033	0.061	0.916	1.166	0.449	Genotyped
rs2381422	9	6,281,211	C	T	0.052	0.050	0.509	1.052	0.076	0.906	1.221	0.410	Imputed
rs4740842	9	6,281,214	C	T	0.082	0.079	0.559	1.037	0.062	0.919	1.170	0.385	Imputed
rs3955036	9	6,281,660	A	G	0.051	0.048	0.476	1.057	0.077	0.908	1.229	0.511	Imputed
rs1322168	9	6,281,929	C	G	0.052	0.050	0.477	1.056	0.076	0.909	1.226	0.418	Imputed
chr9:6281990:I	9	6,281,990	G	GAAAC	0.082	0.080	0.626	1.031	0.062	0.913	1.163	0.319	Imputed
rs1322167	9	6,282,089	A	T	0.052	0.050	0.477	1.056	0.076	0.909	1.226	0.418	Imputed

rs78757963	9	6,282,511	A	G	0.027	0.029	0.550	0.940	0.104	0.766	1.152	0.673	Imputed
rs74438701	9	6,282,794	C	T	0.201	0.172	8.32E-06	1.209	0.043	1.112	1.314	0.828	Imputed
chr9:6282860:D	9	6,282,860	GAAAGAA	G	0.060	0.060	0.910	1.008	0.071	0.877	1.159	0.791	Imputed
rs10758756	9	6,283,418	A	G	0.058	0.055	0.415	1.061	0.072	0.920	1.223	0.515	Imputed
chr9:6283462:I	9	6,283,462	C	CGTG	0.116	0.113	0.645	1.025	0.053	0.923	1.137	0.952	Imputed
rs10815400	9	6,283,626	G	T	0.052	0.050	0.488	1.054	0.076	0.908	1.224	0.400	Imputed
rs10815401	9	6,283,836	C	A	0.052	0.050	0.472	1.056	0.076	0.910	1.226	0.415	Imputed
rs73400516	9	6,284,249	A	T	0.029	0.029	0.949	0.994	0.100	0.816	1.209	0.643	Imputed
rs73400517	9	6,284,665	G	A	0.028	0.029	0.810	0.976	0.102	0.799	1.191	0.683	Imputed
rs13284599	9	6,284,723	C	T	0.051	0.048	0.515	1.052	0.077	0.904	1.224	0.532	Imputed
rs73400519	9	6,284,901	A	G	0.028	0.028	0.853	0.981	0.103	0.802	1.200	0.699	Imputed
rs4742175	9	6,285,050	G	A	0.083	0.080	0.526	1.040	0.061	0.922	1.173	0.371	Imputed
rs7048092	9	6,285,589	G	A	0.052	0.050	0.488	1.054	0.076	0.908	1.224	0.400	Imputed
chr9:6286688:I	9	6,286,688	GA	G	0.012	0.011	0.286	1.181	0.155	0.871	1.601	0.107	Imputed
rs10739090	9	6,287,142	C	A	0.052	0.050	0.472	1.056	0.076	0.910	1.226	0.415	Imputed
rs113797636	9	6,287,556	C	T	0.032	0.031	0.898	1.012	0.097	0.838	1.223	0.694	Imputed
rs10125110	9	6,287,726	C	A	0.052	0.050	0.472	1.056	0.076	0.910	1.226	0.415	Imputed
rs6477063	9	6,288,724	G	A	0.052	0.050	0.467	1.057	0.076	0.910	1.227	0.413	Imputed
rs7858440	9	6,288,823	G	A	0.086	0.082	0.338	1.059	0.060	0.941	1.192	0.325	Imputed
rs7858457	9	6,288,871	G	A	0.053	0.050	0.446	1.059	0.076	0.913	1.229	0.464	Imputed
rs10758757	9	6,288,915	T	C	0.052	0.050	0.537	1.048	0.076	0.903	1.217	0.423	Imputed
rs59210574	9	6,288,927	T	C	0.030	0.031	0.551	0.943	0.099	0.777	1.144	0.276	Imputed
rs7035741	9	6,289,504	C	T	0.052	0.050	0.473	1.056	0.076	0.910	1.226	0.414	Imputed
rs2065073	9	6,290,324	G	A	0.131	0.128	0.706	1.019	0.051	0.923	1.125	0.248	Imputed
rs7036336	9	6,290,673	T	C	0.080	0.078	0.609	1.032	0.062	0.914	1.166	0.389	Imputed
rs59097727	9	6,291,125	T	C	0.087	0.083	0.359	1.057	0.060	0.939	1.189	0.415	Imputed
chr9:6291188:D	9	6,291,188	G	GT	0.034	0.034	0.945	1.007	0.094	0.838	1.210	0.768	Imputed
rs2054315	9	6,291,242	T	C	0.082	0.079	0.537	1.039	0.062	0.921	1.173	0.488	Imputed
rs744567	9	6,292,602	G	C	0.210	0.232	1.12E-03	0.874	0.041	0.806	0.948	0.074	Imputed
rs10739091	9	6,292,919	A	T	0.082	0.079	0.523	1.040	0.062	0.922	1.174	0.481	Imputed
rs141191252	9	6,293,151	A	G	0.022	0.021	0.865	1.020	0.116	0.813	1.279	0.208	Imputed
rs16924291	9	6,293,305	A	C	0.156	0.162	0.412	0.963	0.047	0.879	1.055	0.100	Imputed
rs10739092	9	6,293,434	G	A	0.053	0.050	0.414	1.064	0.076	0.917	1.234	0.620	Imputed
rs10739093	9	6,293,552	T	C	0.052	0.049	0.511	1.051	0.076	0.905	1.222	0.482	Imputed
rs144002592	9	6,293,637	T	A	0.012	0.010	0.275	1.186	0.156	0.873	1.611	0.879	Imputed
rs10815402	9	6,293,715	A	G	0.305	0.270	2.60E-06	1.189	0.037	1.106	1.279	0.595	Genotyped
rs143347713	9	6,294,418	A	G	0.014	0.017	0.193	0.833	0.142	0.630	1.100	0.014	Imputed
rs7034861	9	6,294,824	C	T	0.053	0.050	0.390	1.067	0.076	0.920	1.238	0.604	Imputed
rs7861354	9	6,294,877	G	C	0.053	0.050	0.390	1.067	0.076	0.920	1.238	0.604	Imputed
rs6477064	9	6,294,976	C	T	0.062	0.060	0.597	1.038	0.070	0.904	1.191	0.272	Imputed

rs73400541	9	6,295,277	T	C	0.027	0.028	0.697	0.960	0.105	0.782	1.178	0.676	Imputed
rs4742176	9	6,295,557	C	G	0.061	0.060	0.702	1.027	0.071	0.895	1.180	0.195	Imputed
rs2085853	9	6,296,051	A	G	0.080	0.084	0.347	0.943	0.062	0.835	1.066	0.213	Imputed
rs2085854	9	6,296,085	T	C	0.123	0.126	0.607	0.974	0.052	0.880	1.078	0.665	Imputed
chr9:6296150:D	9	6,296,150	GCAAC	G	0.073	0.074	0.799	0.984	0.065	0.866	1.117	0.410	Imputed
rs148391631	9	6,296,678	A	C	0.027	0.028	0.799	0.974	0.103	0.796	1.193	0.917	Imputed
rs150527924	9	6,296,722	C	T	0.013	0.014	0.524	0.909	0.150	0.677	1.220	0.521	Imputed
rs4304373	9	6,296,994	T	C	0.130	0.130	0.911	0.994	0.051	0.900	1.099	0.968	Imputed
rs4387026	9	6,296,999	A	C	0.139	0.142	0.703	0.981	0.049	0.891	1.081	0.833	Imputed
rs7468538	9	6,297,089	T	A	0.068	0.067	0.858	1.012	0.067	0.887	1.155	0.732	Imputed
rs143305466	9	6,297,108	T	C	0.200	0.216	0.019	0.906	0.042	0.834	0.984	0.056	Imputed
rs10758758	9	6,297,659	A	G	0.053	0.050	0.434	1.061	0.076	0.914	1.231	0.632	Imputed
rs4579586	9	6,297,887	A	G	0.064	0.063	0.989	0.999	0.069	0.872	1.144	0.509	Imputed
rs189010572	9	6,298,328	C	A	0.078	0.079	0.782	0.983	0.063	0.868	1.112	0.192	Imputed
rs192274747	9	6,298,402	C	T	0.107	0.106	0.965	1.002	0.055	0.900	1.117	0.491	Imputed
rs144224570	9	6,298,640	A	G	0.113	0.113	0.946	0.996	0.054	0.897	1.107	0.641	Imputed
rs147733416	9	6,298,770	C	A	0.126	0.125	0.728	1.018	0.051	0.921	1.126	0.540	Imputed
rs189444901	9	6,298,785	C	G	0.122	0.124	0.702	0.980	0.052	0.885	1.085	0.983	Imputed
rs145132871	9	6,299,162	T	A	0.062	0.060	0.480	1.051	0.070	0.916	1.206	0.912	Imputed
rs7389206	9	6,299,359	C	A	0.054	0.053	0.746	1.024	0.075	0.885	1.186	0.714	Imputed
rs139242145	9	6,299,445	T	G	0.031	0.031	0.985	0.998	0.098	0.824	1.209	0.628	Imputed
rs7389144	9	6,299,586	G	A	0.095	0.095	0.927	0.995	0.058	0.888	1.115	0.722	Imputed
rs4364679	9	6,299,810	G	A	0.063	0.062	0.905	1.008	0.070	0.880	1.156	0.161	Imputed
rs4361805	9	6,300,041	A	T	0.054	0.052	0.477	1.055	0.075	0.911	1.221	0.939	Imputed
rs4460429	9	6,300,078	T	C	0.097	0.093	0.394	1.050	0.057	0.939	1.175	0.877	Imputed
rs7036472	9	6,300,410	G	C	0.053	0.050	0.429	1.062	0.076	0.915	1.232	0.629	Imputed
rs6477066	9	6,300,861	A	G	0.060	0.057	0.530	1.046	0.072	0.909	1.203	0.680	Imputed
rs139263223	9	6,301,311	C	T	0.077	0.068	0.031	1.148	0.064	1.013	1.301	0.280	Imputed
rs10815403	9	6,301,348	C	A	0.107	0.105	0.586	1.030	0.055	0.925	1.148	0.945	Imputed
rs17756142	9	6,301,578	C	A	0.223	0.245	1.72E-03	0.881	0.041	0.814	0.954	0.168	Imputed
rs7858373	9	6,301,948	T	G	0.052	0.049	0.506	1.052	0.077	0.906	1.222	0.479	Imputed
rs144676527	9	6,301,951	T	C	0.010	0.011	0.520	0.895	0.172	0.639	1.254	0.669	Imputed
rs1407357	9	6,302,126	C	G	0.052	0.049	0.506	1.052	0.077	0.906	1.222	0.479	Imputed
rs1407358	9	6,302,297	T	G	0.052	0.049	0.506	1.052	0.077	0.906	1.222	0.479	Imputed
rs77011952	9	6,302,569	C	T	0.032	0.032	0.782	0.974	0.097	0.806	1.177	0.348	Imputed
rs67541153	9	6,302,609	C	G	0.050	0.048	0.522	1.051	0.078	0.903	1.223	0.562	Imputed
rs6477067	9	6,302,794	G	A	0.053	0.050	0.390	1.067	0.076	0.920	1.238	0.604	Imputed
rs77836669	9	6,303,154	A	T	0.104	0.095	0.060	1.110	0.056	0.996	1.238	0.360	Imputed
rs4475552	9	6,303,236	T	C	0.056	0.052	0.204	1.098	0.074	0.950	1.269	0.615	Imputed
rs112695858	9	6,303,358	A	G	0.251	0.272	2.76E-03	0.890	0.039	0.825	0.961	0.216	Imputed

rs141092841	9	6,303,390	A	G	0.020	0.020	0.863	0.979	0.122	0.771	1.243	0.329	Imputed
rs10758759	9	6,303,429	A	G	0.063	0.061	0.552	1.042	0.070	0.909	1.195	0.683	Imputed
rs67128098	9	6,303,498	T	A	0.250	0.271	3.70E-03	0.893	0.039	0.827	0.964	0.237	Imputed
rs4742177	9	6,303,825	A	C	0.053	0.050	0.390	1.067	0.076	0.920	1.238	0.604	Imputed
rs993951	9	6,304,401	G	A	0.388	0.403	0.085	0.942	0.035	0.880	1.008	0.670	Imputed
rs142400474	9	6,304,455	C	T	0.021	0.016	0.013	1.346	0.120	1.063	1.705	0.828	Imputed
rs993952	9	6,304,510	G	C	0.053	0.050	0.395	1.066	0.076	0.919	1.237	0.607	Imputed
chr9:6305451:D	9	6,305,451	GTGTT	G	0.053	0.050	0.404	1.065	0.076	0.918	1.236	0.613	Imputed
rs7467258	9	6,305,622	G	A	0.306	0.324	0.018	0.917	0.037	0.853	0.985	0.340	Imputed
rs1935491	9	6,305,699	A	G	0.069	0.069	0.962	1.003	0.067	0.880	1.144	0.235	Imputed
rs2169287	9	6,305,904	A	C	0.244	0.265	3.18E-03	0.891	0.039	0.825	0.962	0.158	Imputed
rs3858051	9	6,306,061	G	A	0.053	0.050	0.400	1.066	0.076	0.919	1.236	0.610	Imputed
rs16924301	9	6,306,093	G	A	0.202	0.175	3.04E-05	1.194	0.042	1.098	1.297	0.873	Imputed
rs7859139	9	6,306,294	G	A	0.053	0.050	0.400	1.066	0.076	0.919	1.236	0.610	Imputed
rs4742178	9	6,306,802	C	T	0.053	0.050	0.400	1.066	0.076	0.919	1.236	0.610	Imputed
rs12237914	9	6,306,896	G	A	0.391	0.405	0.086	0.942	0.035	0.880	1.009	0.656	Genotyped
rs7864212	9	6,307,313	C	T	0.052	0.049	0.511	1.051	0.076	0.905	1.222	0.482	Imputed
rs2019737	9	6,308,381	C	T	0.053	0.050	0.414	1.064	0.076	0.917	1.234	0.591	Imputed
rs10975539	9	6,308,729	T	C	0.215	0.236	1.42E-03	0.877	0.041	0.809	0.951	0.141	Genotyped
rs3847261	9	6,309,188	T	A	0.053	0.050	0.414	1.064	0.076	0.917	1.234	0.591	Imputed
chr9:6309552:D	9	6,309,552	T	TTC	0.030	0.030	0.866	1.017	0.099	0.838	1.233	0.696	Imputed
chr9:6309553:D	9	6,309,553	T	TC	0.030	0.030	0.866	1.017	0.099	0.838	1.233	0.696	Imputed
rs1322166	9	6,309,862	C	T	0.306	0.324	0.014	0.914	0.037	0.851	0.982	0.379	Imputed
rs10975540	9	6,310,195	G	A	0.246	0.266	3.78E-03	0.893	0.039	0.827	0.964	0.100	Imputed
rs1923359	9	6,310,773	C	T	0.052	0.049	0.472	1.056	0.076	0.910	1.227	0.462	Imputed
rs1330380	9	6,310,829	C	A	0.052	0.049	0.472	1.056	0.076	0.910	1.227	0.462	Imputed
rs17705436	9	6,310,908	G	C	0.217	0.238	1.68E-03	0.879	0.041	0.812	0.953	0.114	Imputed
rs1330379	9	6,310,933	T	C	0.052	0.049	0.472	1.056	0.076	0.910	1.227	0.462	Imputed
rs7040412	9	6,311,247	C	T	0.053	0.050	0.400	1.066	0.076	0.919	1.236	0.610	Imputed
rs78370148	9	6,311,511	C	T	0.060	0.062	0.653	0.969	0.071	0.842	1.113	0.850	Imputed
rs62568154	9	6,311,651	T	C	0.153	0.157	0.526	0.971	0.047	0.885	1.064	0.184	Imputed
rs62568155	9	6,312,633	C	T	0.157	0.160	0.609	0.977	0.046	0.892	1.070	0.179	Imputed
rs10815405	9	6,312,731	T	C	0.052	0.049	0.472	1.056	0.076	0.910	1.227	0.462	Imputed
rs10758760	9	6,313,017	C	T	0.053	0.050	0.395	1.066	0.076	0.919	1.237	0.607	Imputed
rs10758761	9	6,313,102	G	A	0.053	0.050	0.395	1.066	0.076	0.919	1.237	0.607	Imputed
rs1973136	9	6,313,276	G	A	0.251	0.273	2.58E-03	0.889	0.039	0.824	0.960	0.219	Imputed
rs1551761	9	6,313,518	C	G	0.053	0.050	0.395	1.066	0.076	0.919	1.237	0.607	Imputed
rs78154521	9	6,313,669	C	T	0.022	0.017	0.031	1.285	0.117	1.022	1.615	0.752	Imputed
rs1330371	9	6,313,785	T	C	0.052	0.050	0.493	1.054	0.076	0.907	1.224	0.473	Imputed
chr9:6313945:D	9	6,313,945	A	ATT	0.045	0.051	0.090	0.872	0.081	0.744	1.022	0.578	Imputed

rs10121888	9	6,314,179	T	C	0.052	0.049	0.472	1.056	0.076	0.910	1.227	0.462	Imputed
rs10975542	9	6,314,293	A	T	0.210	0.226	0.024	0.911	0.041	0.840	0.988	0.521	Imputed
rs7851000	9	6,314,487	T	C	0.244	0.265	2.97E-03	0.890	0.039	0.824	0.961	0.161	Imputed
rs72691711	9	6,315,489	A	G	0.244	0.265	2.97E-03	0.890	0.039	0.824	0.961	0.161	Imputed
rs10758762	9	6,315,544	G	A	0.053	0.050	0.362	1.071	0.075	0.924	1.242	0.586	Imputed
chr9:6316120:I	9	6,316,120	GA	G	0.011	0.012	0.868	0.974	0.160	0.711	1.333	0.678	Imputed
rs1832885	9	6,316,439	C	G	0.052	0.049	0.435	1.061	0.076	0.914	1.232	0.444	Imputed
rs10114407	9	6,317,169	G	C	0.307	0.325	0.015	0.914	0.037	0.851	0.982	0.407	Imputed
rs10975543	9	6,317,385	C	T	0.052	0.050	0.451	1.059	0.076	0.912	1.230	0.452	Imputed
rs7863130	9	6,317,936	G	A	0.072	0.069	0.555	1.040	0.066	0.914	1.182	0.615	Imputed
rs7866963	9	6,318,054	C	T	0.057	0.053	0.339	1.073	0.073	0.929	1.239	0.674	Imputed
rs7867079	9	6,318,105	C	G	0.064	0.059	0.140	1.107	0.069	0.967	1.269	0.550	Imputed
rs111819898	9	6,318,195	C	T	0.245	0.268	8.37E-04	0.877	0.039	0.812	0.947	0.149	Imputed
rs146323377	9	6,319,760	G	A	0.013	0.015	0.696	0.944	0.147	0.708	1.259	0.348	Imputed
rs7857170	9	6,321,472	G	A	0.053	0.050	0.298	1.082	0.075	0.933	1.254	0.625	Imputed
rs7034801	9	6,322,415	C	A	0.053	0.050	0.340	1.075	0.076	0.927	1.246	0.627	Imputed
rs59522894	9	6,322,482	A	G	0.027	0.028	0.838	0.979	0.104	0.799	1.200	0.786	Imputed
rs7020048	9	6,322,644	A	G	0.052	0.049	0.420	1.063	0.076	0.916	1.235	0.537	Imputed
rs55779033	9	6,322,680	C	T	0.271	0.295	8.06E-04	0.881	0.038	0.817	0.949	0.198	Imputed
rs7861651	9	6,322,729	A	G	0.271	0.295	8.06E-04	0.881	0.038	0.817	0.949	0.198	Imputed
rs4742179	9	6,324,376	A	C	0.297	0.315	0.012	0.911	0.037	0.848	0.980	0.324	Imputed
rs16924328	9	6,324,744	C	T	0.112	0.107	0.245	1.064	0.054	0.958	1.183	0.174	Genotyped
rs150209927	9	6,325,197	G	A	0.015	0.016	0.566	0.923	0.140	0.701	1.214	0.804	Imputed
rs10491835	9	6,325,345	A	G	0.198	0.173	1.42E-04	1.177	0.043	1.082	1.280	0.634	Imputed
rs7039968	9	6,326,094	T	G	0.052	0.049	0.420	1.063	0.076	0.916	1.235	0.537	Imputed
rs7036990	9	6,326,256	A	C	0.052	0.049	0.420	1.063	0.076	0.916	1.235	0.537	Imputed
rs10758763	9	6,326,371	T	C	0.052	0.049	0.420	1.063	0.076	0.916	1.235	0.537	Imputed
rs7864863	9	6,326,763	A	G	0.077	0.080	0.538	0.962	0.063	0.850	1.089	0.821	Imputed
rs10758764	9	6,326,825	T	A	0.297	0.316	0.012	0.911	0.037	0.847	0.980	0.308	Imputed
rs7041863	9	6,327,690	A	C	0.053	0.050	0.371	1.070	0.076	0.922	1.241	0.649	Imputed
rs7045164	9	6,327,778	T	C	0.052	0.049	0.420	1.063	0.076	0.916	1.235	0.537	Imputed
rs11553814	9	6,328,440	T	C	0.104	0.098	0.186	1.076	0.056	0.965	1.200	0.243	Imputed
rs117475693	9	6,328,504	T	G	0.033	0.035	0.561	0.947	0.094	0.787	1.139	0.925	Imputed
rs3847262	9	6,328,947	T	C	0.053	0.050	0.390	1.067	0.076	0.920	1.238	0.662	Imputed
rs117022582	9	6,328,996	A	G	0.009	0.011	0.095	0.741	0.180	0.520	1.054	0.641	Imputed
chr9:6329010:D	9	6,329,010	T	TG	0.018	0.017	0.687	1.053	0.128	0.819	1.354	0.813	Imputed
rs898673	9	6,329,888	A	G	0.053	0.050	0.376	1.069	0.076	0.922	1.240	0.652	Imputed
rs1052335	9	6,330,380	C	A	0.221	0.191	6.28E-06	1.204	0.041	1.111	1.305	0.612	Genotyped
rs1037885	9	6,330,915	G	A	0.053	0.050	0.385	1.068	0.076	0.921	1.238	0.630	Imputed
rs2890707	9	6,331,324	A	G	0.053	0.050	0.371	1.070	0.076	0.923	1.241	0.679	Genotyped

rs10491836	9	6,331,421	A	C	0.244	0.266	1.69E-03	0.884	0.039	0.818	0.955	0.241	Imputed
rs16924356	9	6,331,610	A	G	0.244	0.266	2.06E-03	0.886	0.039	0.820	0.957	0.271	Genotyped
chr9:6331746:D	9	6,331,746	T	TG	0.051	0.048	0.466	1.058	0.077	0.909	1.231	0.587	Imputed
rs147698134	9	6,332,119	G	C	0.029	0.027	0.482	1.074	0.101	0.881	1.310	0.407	Imputed
rs79822462	9	6,332,216	A	G	0.061	0.069	0.037	0.864	0.070	0.753	0.992	0.841	Imputed
rs721352	9	6,332,901	A	C	0.299	0.317	0.012	0.912	0.037	0.848	0.980	0.391	Imputed
rs1381038	9	6,333,156	C	A	0.053	0.050	0.367	1.071	0.076	0.923	1.241	0.589	Imputed
rs2381426	9	6,333,666	A	C	0.302	0.321	0.010	0.910	0.037	0.847	0.978	0.377	Imputed
rs10739094	9	6,333,685	A	T	0.244	0.266	1.72E-03	0.884	0.039	0.819	0.955	0.242	Imputed
rs7048431	9	6,333,884	T	C	0.057	0.054	0.460	1.055	0.073	0.915	1.218	0.674	Imputed
chr9:6334994:D	9	6,334,994	AATATTCATTTAAATACAT	A	0.052	0.050	0.477	1.056	0.076	0.909	1.226	0.517	Imputed
rs7850988	9	6,335,760	T	A	0.244	0.266	1.53E-03	0.883	0.039	0.817	0.954	0.234	Imputed
rs4008349	9	6,336,933	C	T	0.055	0.054	0.652	1.034	0.074	0.894	1.196	0.542	Imputed
rs6477070	9	6,337,798	A	G	0.071	0.070	0.952	1.004	0.066	0.882	1.143	0.210	Imputed
rs77585556	9	6,338,950	C	G	0.302	0.321	7.61E-03	0.907	0.037	0.843	0.974	0.350	Imputed
rs148013890	9	6,339,453	T	C	0.025	0.026	0.601	0.945	0.109	0.764	1.169	0.740	Imputed
rs184096960	9	6,339,541	C	T	0.014	0.015	0.653	0.937	0.145	0.706	1.244	0.581	Imputed
rs145927359	9	6,339,589	T	C	0.016	0.018	0.402	0.894	0.134	0.688	1.162	0.617	Imputed
rs148533485	9	6,339,711	C	A	0.053	0.052	0.636	1.036	0.075	0.894	1.201	0.638	Imputed
rs113612935	9	6,341,835	T	C	0.011	0.011	0.814	0.963	0.163	0.700	1.324	0.680	Imputed
rs731585	9	6,342,328	G	A	0.302	0.322	6.28E-03	0.904	0.037	0.841	0.972	0.308	Genotyped
rs1381039	9	6,342,682	A	G	0.055	0.054	0.710	1.028	0.074	0.889	1.188	0.586	Imputed
rs2381438	9	6,342,855	G	A	0.055	0.053	0.538	1.047	0.074	0.905	1.210	0.448	Genotyped
rs10975547	9	6,343,945	A	G	0.244	0.265	1.95E-03	0.885	0.039	0.820	0.956	0.250	Imputed
rs1574817	9	6,345,296	T	G	0.302	0.321	8.86E-03	0.908	0.037	0.845	0.976	0.321	Imputed
rs187800028	9	6,345,588	G	T	0.024	0.023	0.738	1.038	0.111	0.835	1.289	0.925	Imputed
rs7027505	9	6,345,740	T	C	0.244	0.266	1.55E-03	0.883	0.039	0.818	0.954	0.222	Imputed
rs7026758	9	6,345,895	G	C	0.066	0.064	0.709	1.026	0.068	0.897	1.173	0.773	Imputed
rs117509016	9	6,346,602	C	T	0.013	0.012	0.490	1.111	0.153	0.824	1.499	0.217	Imputed
rs7020404	9	6,346,844	T	C	0.302	0.321	8.38E-03	0.908	0.037	0.844	0.975	0.325	Imputed
chr9:6347175:D	9	6,347,175	T	TTG	0.263	0.280	0.015	0.911	0.038	0.845	0.982	0.335	Imputed
rs7034658	9	6,347,185	G	A	0.097	0.099	0.685	0.977	0.057	0.873	1.093	0.985	Imputed
rs34373422	9	6,347,189	A	G	0.237	0.261	6.12E-04	0.873	0.040	0.808	0.944	0.184	Imputed
rs7035333	9	6,347,651	A	G	0.217	0.239	1.52E-03	0.878	0.041	0.811	0.952	0.206	Imputed
chr9:6347745:D	9	6,347,745	C	CTCAATTTAGAT	0.020	0.020	0.882	1.018	0.120	0.805	1.287	0.253	Imputed
rs7035574	9	6,347,838	G	T	0.056	0.054	0.654	1.034	0.074	0.895	1.194	0.656	Imputed
rs7039959	9	6,348,295	C	G	0.431	0.441	0.233	0.960	0.034	0.898	1.027	0.960	Imputed
rs7039175	9	6,348,355	G	A	0.426	0.439	0.111	0.947	0.034	0.886	1.013	0.940	Imputed
rs7040374	9	6,348,594	C	T	0.055	0.054	0.651	1.034	0.074	0.894	1.196	0.637	Imputed
rs12551796	9	6,349,624	C	G	0.028	0.029	0.692	0.960	0.103	0.785	1.174	0.880	Imputed

rs2169282	9	6,350,235	A	G	0.388	0.404	0.038	0.930	0.035	0.869	0.996	0.785	Imputed
rs150673352	9	6,350,441	T	A	0.029	0.027	0.489	1.073	0.101	0.880	1.309	0.404	Imputed
rs10975551	9	6,350,680	A	G	0.010	0.011	0.542	0.902	0.168	0.649	1.255	0.315	Imputed
rs149902466	9	6,351,038	G	A	0.029	0.027	0.489	1.073	0.101	0.880	1.309	0.404	Imputed
rs16924428	9	6,351,111	G	A	0.245	0.267	1.34E-03	0.882	0.039	0.816	0.952	0.236	Genotyped
rs10815411	9	6,351,521	C	T	0.418	0.432	0.070	0.940	0.034	0.879	1.005	0.992	Imputed
rs10975552	9	6,351,834	C	T	0.360	0.377	0.032	0.927	0.035	0.865	0.993	0.924	Imputed
rs117050463	9	6,352,321	T	A	0.037	0.037	0.880	1.014	0.090	0.851	1.208	0.855	Imputed
rs4742180	9	6,352,776	C	T	0.052	0.050	0.555	1.046	0.076	0.901	1.215	0.530	Imputed
rs10975553	9	6,352,819	C	T	0.360	0.377	0.033	0.928	0.035	0.866	0.994	0.934	Imputed
rs118049402	9	6,353,634	A	T	0.025	0.026	0.585	0.943	0.109	0.762	1.166	0.710	Imputed
rs10758768	9	6,354,290	A	T	0.052	0.050	0.549	1.047	0.076	0.901	1.216	0.527	Imputed
rs7032411	9	6,354,990	T	C	0.053	0.051	0.571	1.044	0.076	0.900	1.211	0.554	Imputed
rs191804856	9	6,356,269	G	A	0.009	0.011	0.161	0.776	0.181	0.545	1.106	0.556	Imputed
rs7021445	9	6,356,657	C	T	0.052	0.050	0.572	1.044	0.076	0.899	1.212	0.684	Imputed
rs7350177	9	6,357,262	C	T	0.052	0.050	0.585	1.043	0.077	0.897	1.212	0.545	Imputed
rs16924434	9	6,358,334	G	A	0.118	0.113	0.254	1.062	0.052	0.958	1.177	0.261	Imputed
rs13298872	9	6,358,385	A	G	0.050	0.048	0.587	1.043	0.078	0.896	1.215	0.526	Imputed
rs6477071	9	6,358,849	T	C	0.051	0.050	0.627	1.038	0.077	0.893	1.206	0.472	Imputed
rs7022186	9	6,359,144	C	T	0.360	0.377	0.034	0.928	0.035	0.866	0.995	0.939	Imputed
rs113214932	9	6,359,742	C	G	0.030	0.030	0.901	0.988	0.100	0.813	1.201	0.677	Imputed
rs10815412	9	6,360,078	T	C	0.414	0.428	0.073	0.940	0.034	0.879	1.006	0.891	Imputed
rs1969731	9	6,360,165	C	T	0.360	0.377	0.036	0.929	0.035	0.867	0.995	0.972	Imputed
rs10975555	9	6,360,299	G	C	0.144	0.155	0.068	0.916	0.048	0.834	1.006	0.731	Imputed
rs141563240	9	6,360,766	T	C	0.021	0.025	0.061	0.802	0.118	0.636	1.010	0.738	Imputed
rs59606381	9	6,362,124	T	C	0.242	0.264	1.86E-03	0.885	0.039	0.819	0.956	0.334	Imputed
rs7851246	9	6,362,365	A	G	0.242	0.264	1.86E-03	0.885	0.039	0.819	0.956	0.334	Imputed
rs10975556	9	6,363,043	G	C	0.057	0.059	0.559	0.958	0.073	0.831	1.105	0.573	Imputed
rs4742181	9	6,363,694	A	G	0.414	0.429	0.068	0.939	0.034	0.878	1.005	0.928	Imputed
rs10975557	9	6,364,332	G	A	0.371	0.387	0.042	0.931	0.035	0.870	0.997	0.883	Imputed
rs10975558	9	6,364,449	T	C	0.241	0.262	2.01E-03	0.885	0.039	0.820	0.957	0.438	Imputed
rs7875811	9	6,364,532	G	A	0.347	0.360	0.120	0.946	0.036	0.883	1.014	0.729	Imputed
rs7875812	9	6,364,533	T	A	0.347	0.360	0.120	0.946	0.036	0.883	1.014	0.729	Imputed
rs719724	9	6,365,614	T	A	0.416	0.430	0.070	0.940	0.034	0.879	1.005	0.965	Imputed
rs719725	9	6,365,683	C	A	0.362	0.379	0.032	0.927	0.035	0.866	0.993	0.887	Imputed
rs4361812	9	6,365,737	A	C	0.150	0.159	0.091	0.923	0.047	0.841	1.013	0.262	Imputed
rs149305145	9	6,367,571	A	G	0.020	0.020	0.818	1.028	0.120	0.813	1.301	0.624	Imputed
rs144616231	9	6,367,994	T	C	0.021	0.015	0.014	1.346	0.121	1.061	1.706	0.829	Imputed
rs1322171	9	6,368,103	T	C	0.403	0.420	0.039	0.931	0.034	0.871	0.997	0.740	Imputed
rs1575264	9	6,368,793	T	C	0.412	0.428	0.057	0.937	0.034	0.876	1.002	0.762	Imputed

rs1575263	9	6,368,811	G	A	0.412	0.427	0.058	0.937	0.034	0.876	1.002	0.730	Imputed
rs4008347	9	6,370,088	A	G	0.052	0.049	0.555	1.046	0.077	0.900	1.216	0.440	Imputed
rs7020964	9	6,370,314	C	A	0.360	0.378	0.026	0.925	0.035	0.863	0.991	0.964	Imputed
rs7036261	9	6,370,537	C	T	0.052	0.050	0.561	1.045	0.077	0.900	1.215	0.443	Imputed
chr9:6370563:I	9	6,370,563	AT	A	0.355	0.368	0.115	0.946	0.035	0.883	1.014	0.919	Imputed
rs10758769	9	6,370,579	G	A	0.414	0.429	0.064	0.939	0.034	0.878	1.004	0.777	Imputed
rs7036800	9	6,370,970	C	T	0.412	0.428	0.057	0.937	0.034	0.876	1.002	0.687	Imputed
rs10815415	9	6,371,119	G	C	0.413	0.428	0.053	0.936	0.034	0.875	1.001	0.706	Imputed
rs66514552	9	6,371,626	A	G	0.360	0.378	0.026	0.924	0.035	0.863	0.991	0.966	Imputed
rs60170566	9	6,371,855	A	G	0.142	0.154	0.057	0.912	0.048	0.830	1.003	0.580	Imputed
rs1841522	9	6,372,380	G	T	0.415	0.429	0.071	0.940	0.034	0.879	1.005	0.758	Imputed
rs117479012	9	6,372,747	A	C	0.015	0.017	0.473	0.906	0.137	0.693	1.186	0.511	Imputed
rs1841521	9	6,372,748	G	A	0.415	0.430	0.071	0.940	0.034	0.879	1.005	0.760	Imputed
rs79391222	9	6,373,017	C	T	0.011	0.011	0.961	0.992	0.159	0.726	1.356	0.462	Imputed
rs2381440	9	6,373,548	G	A	0.052	0.050	0.520	1.050	0.076	0.904	1.220	0.380	Imputed
chr9:6373683:I	9	6,373,683	TA	T	0.022	0.017	0.013	1.338	0.117	1.063	1.683	0.166	Imputed
rs146342883	9	6,373,933	A	C	0.031	0.032	0.991	0.999	0.097	0.826	1.208	0.755	Imputed
rs2085856	9	6,374,700	G	C	0.067	0.067	0.997	1.000	0.068	0.875	1.142	0.516	Imputed
rs139561193	9	6,374,734	A	C	0.359	0.377	0.024	0.923	0.035	0.862	0.989	0.976	Imputed
rs2085855	9	6,374,850	A	G	0.039	0.047	0.025	0.824	0.086	0.696	0.976	0.945	Imputed
rs10481526	9	6,374,992	G	A	0.422	0.439	0.045	0.934	0.034	0.873	0.998	0.753	Imputed
rs150868241	9	6,375,129	G	T	0.359	0.377	0.024	0.924	0.035	0.862	0.990	0.978	Imputed
rs148629067	9	6,375,523	T	C	0.021	0.015	0.018	1.331	0.122	1.049	1.690	0.866	Imputed
rs10758771	9	6,375,721	G	C	0.054	0.054	0.840	1.015	0.075	0.877	1.175	0.462	Imputed
rs139930515	9	6,375,732	A	C	0.192	0.163	6.26E-06	1.216	0.043	1.117	1.324	0.806	Imputed
rs149428115	9	6,375,915	A	C	0.039	0.040	0.634	0.959	0.088	0.808	1.139	0.746	Imputed
rs4742186	9	6,376,072	C	G	0.052	0.050	0.526	1.050	0.076	0.904	1.219	0.382	Imputed
rs7031546	9	6,376,567	C	T	0.434	0.449	0.072	0.941	0.034	0.880	1.006	0.946	Imputed
rs7020277	9	6,376,880	T	G	0.422	0.438	0.047	0.934	0.034	0.874	0.999	0.762	Imputed
rs6477075	9	6,377,001	C	A	0.058	0.056	0.552	1.044	0.073	0.906	1.204	0.675	Imputed
rs7862235	9	6,377,420	G	A	0.053	0.051	0.568	1.044	0.075	0.901	1.210	0.364	Imputed
rs148346756	9	6,377,554	C	G	0.192	0.163	6.74E-06	1.215	0.043	1.116	1.323	0.793	Imputed
rs115800789	9	6,377,630	T	A	0.414	0.429	0.065	0.939	0.034	0.878	1.004	0.766	Imputed
rs144102778	9	6,377,811	A	G	0.023	0.022	0.801	1.029	0.114	0.823	1.287	0.328	Imputed
rs7863762	9	6,377,924	C	T	0.088	0.091	0.554	0.965	0.060	0.859	1.085	0.637	Imputed
rs150625359	9	6,378,088	A	G	0.050	0.048	0.544	1.048	0.078	0.900	1.220	0.455	Imputed
rs2381441	9	6,378,321	C	A	0.082	0.085	0.468	0.956	0.062	0.847	1.079	0.286	Imputed
rs7469403	9	6,378,348	C	A	0.081	0.083	0.581	0.966	0.062	0.856	1.091	0.433	Imputed
rs150723012	9	6,379,761	C	T	0.057	0.059	0.568	0.959	0.073	0.832	1.107	0.579	Imputed
rs151067330	9	6,379,829	T	G	0.426	0.441	0.060	0.938	0.034	0.877	1.003	0.738	Imputed

rs2381442	9	6,380,582	A	G	0.058	0.057	0.576	1.041	0.072	0.904	1.199	0.326	Imputed
rs7045441	9	6,381,124	A	C	0.081	0.083	0.541	0.963	0.062	0.853	1.087	0.385	Imputed
rs2381443	9	6,381,854	G	A	0.068	0.068	0.900	1.008	0.067	0.884	1.150	0.328	Imputed
rs140828452	9	6,381,890	C	T	0.021	0.015	0.017	1.337	0.122	1.053	1.697	0.852	Imputed
rs7850770	9	6,382,357	T	C	0.415	0.429	0.071	0.940	0.034	0.879	1.005	0.720	Imputed
chr9:6382912:D	9	6,382,912	A	AATG	0.483	0.472	0.261	1.039	0.034	0.972	1.111	0.863	Imputed
rs111546416	9	6,383,035	T	G	0.057	0.059	0.535	0.956	0.073	0.828	1.103	0.559	Imputed
rs117627540	9	6,383,392	C	A	0.008	0.011	0.061	0.709	0.185	0.494	1.018	0.979	Imputed
rs1322170	9	6,384,413	G	C	0.058	0.056	0.547	1.044	0.072	0.907	1.203	0.702	Imputed
chr9:6385412:I	9	6,385,412	A	AC	0.424	0.439	0.063	0.939	0.034	0.878	1.004	0.808	Imputed
rs9802183	9	6,385,594	G	A	0.191	0.163	1.26E-05	1.209	0.043	1.110	1.316	0.968	Imputed
rs7860427	9	6,385,637	A	G	0.418	0.433	0.071	0.940	0.034	0.879	1.005	0.724	Imputed
rs146228568	9	6,386,173	T	G	0.034	0.032	0.356	1.090	0.094	0.907	1.310	0.696	Imputed
rs67852216	9	6,386,227	T	C	0.364	0.382	0.027	0.925	0.035	0.864	0.991	0.953	Imputed
rs13288823	9	6,386,584	T	C	0.051	0.048	0.514	1.052	0.077	0.904	1.224	0.441	Imputed
rs60842789	9	6,387,076	T	C	0.363	0.381	0.029	0.926	0.035	0.864	0.992	0.940	Imputed
rs117715439	9	6,387,093	C	A	0.054	0.060	0.081	0.879	0.074	0.760	1.017	0.201	Imputed
rs4742192	9	6,387,438	T	C	0.418	0.433	0.071	0.940	0.034	0.879	1.005	0.724	Imputed
rs143805769	9	6,387,543	G	T	0.033	0.039	0.049	0.832	0.093	0.693	0.999	0.633	Imputed
rs10758779	9	6,388,101	A	G	0.159	0.156	0.642	1.022	0.047	0.933	1.119	0.787	Imputed
rs11515413	9	6,388,284	C	G	0.057	0.059	0.576	0.960	0.073	0.833	1.107	0.610	Imputed
rs113659694	9	6,388,425	T	C	0.073	0.076	0.599	0.967	0.065	0.851	1.097	0.698	Imputed
rs7857005	9	6,388,782	G	T	0.037	0.039	0.385	0.925	0.090	0.776	1.103	0.596	Imputed
rs10481598	9	6,388,803	T	C	0.432	0.448	0.054	0.936	0.034	0.876	1.001	0.730	Imputed
rs147244958	9	6,389,024	A	T	0.011	0.012	0.449	0.885	0.162	0.644	1.215	0.906	Imputed
rs4512433	9	6,389,392	A	G	0.113	0.107	0.234	1.066	0.054	0.960	1.184	0.089	Imputed
rs7044863	9	6,390,196	A	G	0.349	0.363	0.074	0.939	0.035	0.875	1.006	0.930	Imputed
rs10739095	9	6,391,650	C	T	0.052	0.050	0.548	1.047	0.076	0.902	1.216	0.392	Imputed
rs117380846	9	6,391,928	C	G	0.161	0.165	0.630	0.978	0.046	0.894	1.070	0.579	Imputed
rs180739808	9	6,392,305	C	G	0.010	0.010	0.707	1.065	0.167	0.768	1.476	0.694	Imputed
rs1853203	9	6,393,489	A	G	0.053	0.051	0.517	1.050	0.075	0.906	1.217	0.385	Imputed
chr9:6394036:D	9	6,394,036	CA	C	0.052	0.050	0.548	1.047	0.076	0.902	1.216	0.392	Imputed
rs4742193	9	6,394,193	G	C	0.052	0.050	0.531	1.049	0.076	0.903	1.218	0.385	Imputed
rs117419310	9	6,394,314	G	T	0.119	0.115	0.380	1.047	0.052	0.945	1.160	0.124	Imputed
rs7025295	9	6,395,247	C	T	0.360	0.377	0.029	0.926	0.035	0.864	0.992	0.857	Genotyped
rs7850497	9	6,395,540	A	T	0.414	0.429	0.072	0.940	0.034	0.879	1.005	0.749	Imputed
rs7850991	9	6,395,945	A	G	0.053	0.051	0.546	1.047	0.076	0.902	1.214	0.363	Imputed
rs10217560	9	6,396,215	G	T	0.053	0.050	0.507	1.051	0.076	0.906	1.220	0.497	Imputed
rs10217561	9	6,396,245	G	A	0.414	0.429	0.071	0.940	0.034	0.879	1.005	0.750	Imputed
rs61285187	9	6,396,460	T	A	0.360	0.377	0.028	0.926	0.035	0.864	0.992	0.996	Imputed

rs1322169	9	6,397,144	C	A	0.052	0.050	0.526	1.050	0.076	0.904	1.219	0.382	Imputed
rs186324104	9	6,397,412	A	G	0.011	0.012	0.522	0.901	0.164	0.654	1.241	0.440	Imputed
rs10217461	9	6,397,990	A	G	0.052	0.050	0.526	1.050	0.076	0.904	1.219	0.382	Imputed
rs10975575	9	6,398,151	A	G	0.356	0.374	0.024	0.924	0.035	0.862	0.990	0.987	Imputed
rs10448242	9	6,398,529	T	C	0.053	0.051	0.464	1.057	0.075	0.912	1.225	0.475	Imputed
rs79975071	9	6,399,107	C	T	0.016	0.018	0.282	0.864	0.136	0.662	1.127	0.471	Imputed
rs922221	9	6,399,588	C	G	0.052	0.050	0.526	1.050	0.076	0.904	1.219	0.382	Imputed
rs187087646	9	6,399,935	A	G	0.010	0.012	0.260	0.829	0.167	0.598	1.150	0.627	Imputed
rs10815428	9	6,400,030	T	C	0.394	0.405	0.179	0.955	0.035	0.892	1.022	0.560	Imputed
rs76843856	9	6,400,111	A	G	0.202	0.177	9.42E-05	1.180	0.042	1.086	1.282	0.700	Imputed
rs7852138	9	6,400,367	T	C	0.052	0.050	0.526	1.050	0.076	0.904	1.219	0.382	Imputed
rs78554705	9	6,400,482	A	T	0.197	0.170	3.49E-05	1.194	0.043	1.098	1.299	0.686	Imputed
rs76381166	9	6,400,828	A	G	0.197	0.170	3.49E-05	1.194	0.043	1.098	1.299	0.686	Imputed
rs62566113	9	6,400,884	A	G	0.160	0.164	0.693	0.982	0.046	0.897	1.075	0.558	Imputed
rs4742194	9	6,401,077	A	G	0.225	0.234	0.226	0.952	0.040	0.880	1.031	0.624	Genotyped
rs10115362	9	6,401,096	A	C	0.361	0.378	0.032	0.928	0.035	0.866	0.994	0.992	Imputed
chr9:6401331:D	9	6,401,331	A	AT	0.349	0.364	0.064	0.937	0.035	0.874	1.004	0.897	Imputed
chr9:6401669:D	9	6,401,669	T	TG	0.363	0.381	0.026	0.925	0.035	0.863	0.991	0.952	Imputed
rs12115625	9	6,402,181	C	G	0.199	0.172	3.30E-05	1.194	0.043	1.098	1.298	0.657	Imputed
rs10758780	9	6,402,450	G	C	0.417	0.432	0.081	0.942	0.034	0.881	1.007	0.690	Imputed
rs60412035	9	6,402,546	T	C	0.143	0.139	0.380	1.043	0.048	0.949	1.147	0.090	Imputed
chr9:6402799:D	9	6,402,799	TC	T	0.080	0.080	0.942	1.005	0.062	0.889	1.135	0.283	Imputed
chr9:6402801:D	9	6,402,801	CT	C	0.065	0.064	0.862	1.012	0.069	0.885	1.157	0.274	Imputed
rs7045097	9	6,402,856	C	T	0.415	0.430	0.055	0.936	0.034	0.876	1.001	0.716	Imputed
rs2019676	9	6,403,232	G	A	0.415	0.430	0.065	0.939	0.034	0.878	1.004	0.697	Imputed
rs922219	9	6,403,491	A	G	0.054	0.051	0.422	1.062	0.075	0.917	1.231	0.518	Imputed
rs117180222	9	6,403,709	A	G	0.019	0.017	0.394	1.112	0.125	0.871	1.419	0.170	Imputed
rs6477078	9	6,404,133	A	T	0.053	0.050	0.481	1.055	0.076	0.909	1.224	0.421	Imputed
rs4742195	9	6,404,430	G	T	0.057	0.055	0.579	1.041	0.073	0.903	1.201	0.536	Imputed
rs10739096	9	6,404,888	A	G	0.054	0.052	0.461	1.057	0.075	0.912	1.224	0.555	Imputed
rs7854026	9	6,405,525	G	T	0.059	0.057	0.620	1.036	0.072	0.900	1.193	0.229	Imputed
rs7873206	9	6,405,568	T	C	0.054	0.051	0.427	1.061	0.075	0.916	1.230	0.520	Imputed
chr9:6405721:D	9	6,405,721	TG	T	0.424	0.439	0.052	0.936	0.034	0.875	1.001	0.477	Imputed
chr9:6405725:D	9	6,405,725	GT	G	0.423	0.439	0.044	0.933	0.034	0.873	0.998	0.503	Imputed
rs10815429	9	6,405,753	A	T	0.048	0.045	0.397	1.069	0.079	0.916	1.249	0.566	Imputed
rs115196067	9	6,406,374	T	C	0.010	0.010	0.772	0.952	0.168	0.685	1.325	0.896	Imputed
chr9:6406388:D	9	6,406,388	CGA	C	0.077	0.075	0.739	1.021	0.064	0.902	1.157	0.308	Imputed
rs10758782	9	6,406,428	G	T	0.057	0.056	0.694	1.029	0.073	0.892	1.187	0.737	Imputed
rs10123605	9	6,406,514	T	G	0.054	0.051	0.427	1.061	0.075	0.916	1.230	0.520	Imputed
rs7031407	9	6,406,588	C	T	0.054	0.051	0.427	1.061	0.075	0.916	1.230	0.520	Imputed

rs7031967	9	6,406,911	C	T	0.057	0.053	0.336	1.073	0.073	0.929	1.238	0.512	Imputed
rs10758783	9	6,407,799	T	C	0.414	0.429	0.063	0.938	0.034	0.877	1.003	0.699	Imputed
rs10739097	9	6,407,843	T	C	0.414	0.429	0.072	0.940	0.034	0.879	1.006	0.686	Genotyped
rs7865955	9	6,408,247	G	C	0.414	0.430	0.051	0.935	0.034	0.874	1.000	0.671	Imputed
rs62566116	9	6,408,393	G	A	0.064	0.067	0.559	0.960	0.069	0.839	1.100	0.581	Imputed
rs10125313	9	6,408,429	T	C	0.054	0.051	0.452	1.058	0.075	0.913	1.226	0.484	Genotyped
rs10115294	9	6,408,732	G	T	0.054	0.051	0.452	1.058	0.075	0.913	1.226	0.509	Imputed
rs10815430	9	6,408,875	T	C	0.054	0.051	0.452	1.058	0.075	0.913	1.226	0.509	Imputed
rs7043887	9	6,409,139	C	A	0.054	0.051	0.452	1.058	0.075	0.913	1.226	0.509	Imputed
chr9:6409390:I	9	6,409,390	C	CCAAA	0.055	0.052	0.369	1.069	0.074	0.924	1.237	0.595	Imputed
rs79196351	9	6,409,407	C	A	0.109	0.110	0.968	1.002	0.054	0.901	1.114	0.650	Imputed
rs7857628	9	6,409,874	G	A	0.359	0.377	0.020	0.921	0.035	0.860	0.987	0.812	Imputed
rs138399546	9	6,410,215	T	C	0.029	0.027	0.440	1.082	0.102	0.886	1.321	0.639	Imputed
rs72691740	9	6,411,087	A	G	0.072	0.079	0.075	0.891	0.065	0.784	1.012	0.997	Imputed
rs77122447	9	6,411,110	C	T	0.042	0.044	0.609	0.958	0.084	0.812	1.130	0.704	Imputed
rs28615746	9	6,411,160	C	T	0.053	0.051	0.528	1.049	0.075	0.905	1.216	0.459	Imputed
rs7851921	9	6,411,167	A	T	0.052	0.050	0.589	1.042	0.076	0.897	1.210	0.366	Imputed
rs10815431	9	6,411,638	C	G	0.058	0.057	0.874	1.012	0.072	0.878	1.166	0.170	Imputed
rs12376117	9	6,411,838	C	G	0.413	0.429	0.046	0.934	0.034	0.873	0.999	0.617	Imputed
chr9:6412187:I	9	6,412,187	AC	A	0.011	0.011	0.627	0.924	0.163	0.672	1.271	0.905	Imputed
chr9:6412951:I	9	6,412,951	T	TG	0.053	0.055	0.727	0.974	0.075	0.841	1.129	0.400	Imputed
rs2381444	9	6,413,895	C	T	0.206	0.205	0.773	1.012	0.042	0.933	1.099	0.011	Imputed
rs10975579	9	6,413,904	T	A	0.206	0.204	0.697	1.016	0.042	0.936	1.103	0.013	Imputed
rs2169283	9	6,413,919	A	C	0.205	0.204	0.788	1.011	0.042	0.932	1.098	0.014	Imputed
rs41313446	9	6,414,479	A	C	0.017	0.015	0.301	1.147	0.133	0.884	1.488	0.777	Imputed
rs10975580	9	6,414,839	G	A	0.091	0.099	0.185	0.925	0.059	0.825	1.038	0.514	Genotyped
rs10758784	9	6,415,025	G	C	0.203	0.199	0.428	1.034	0.042	0.952	1.123	0.058	Imputed
rs10758785	9	6,415,230	T	G	0.174	0.171	0.637	1.021	0.045	0.936	1.115	0.028	Imputed
rs7044749	9	6,415,241	T	C	0.052	0.050	0.492	1.054	0.076	0.908	1.223	0.449	Imputed
rs12351492	9	6,415,338	A	G	0.221	0.196	1.45E-04	1.168	0.041	1.078	1.266	0.713	Imputed
rs41306063	9	6,415,643	G	A	0.035	0.035	0.973	1.003	0.092	0.838	1.202	0.434	Imputed
chr9:6416076:D	9	6,416,076	T	TG	0.010	0.011	0.494	0.889	0.172	0.634	1.246	0.271	Imputed
rs10975582	9	6,416,635	C	G	0.192	0.188	0.372	1.039	0.043	0.955	1.130	0.107	Imputed
rs10815432	9	6,416,762	C	G	0.206	0.206	0.904	1.005	0.042	0.926	1.091	0.067	Imputed
rs188704894	9	6,416,819	T	G	0.017	0.014	0.330	1.139	0.134	0.877	1.480	0.857	Imputed
rs10975583	9	6,416,844	C	T	0.174	0.172	0.651	1.020	0.045	0.935	1.113	0.029	Genotyped
rs10739098	9	6,417,677	G	A	0.174	0.171	0.637	1.021	0.045	0.936	1.115	0.028	Imputed
rs1156869	9	6,418,000	G	C	0.053	0.050	0.424	1.062	0.076	0.916	1.233	0.502	Imputed
rs2065075	9	6,418,041	T	A	0.175	0.173	0.629	1.022	0.044	0.936	1.115	0.033	Imputed
rs10758786	9	6,418,822	C	G	0.174	0.172	0.610	1.023	0.045	0.937	1.116	0.032	Imputed

rs10758787	9	6,418,829	C	T	0.174	0.171	0.593	1.024	0.045	0.938	1.118	0.031	Imputed
rs4742198	9	6,419,653	C	A	0.052	0.050	0.476	1.056	0.076	0.909	1.226	0.466	Imputed
rs4742199	9	6,419,853	C	T	0.204	0.200	0.431	1.034	0.042	0.952	1.122	0.055	Imputed
rs2065074	9	6,420,225	C	T	0.189	0.185	0.509	1.029	0.043	0.945	1.120	0.067	Imputed
rs56411118	9	6,421,475	T	C	0.093	0.099	0.254	0.936	0.058	0.835	1.049	0.547	Imputed
rs10733529	9	6,423,110	C	T	0.206	0.201	0.392	1.036	0.042	0.955	1.125	0.062	Imputed
rs1156747	9	6,423,233	G	A	0.155	0.152	0.528	1.030	0.047	0.940	1.129	0.098	Genotyped
rs56939673	9	6,423,672	A	G	0.154	0.152	0.739	1.016	0.047	0.927	1.113	0.059	Imputed
rs10815433	9	6,423,738	G	A	0.471	0.446	2.93E-03	1.106	0.034	1.035	1.182	0.412	Imputed
rs184047209	9	6,423,949	T	A	0.020	0.025	0.090	0.819	0.118	0.650	1.033	0.420	Imputed
chr9:6424014:I	9	6,424,014	A	AC	0.052	0.050	0.482	1.055	0.076	0.909	1.225	0.469	Imputed
rs10975585	9	6,424,252	T	C	0.154	0.150	0.476	1.034	0.047	0.943	1.133	0.105	Imputed
chr9:6424380:D	9	6,424,380	AAG	A	0.209	0.203	0.248	1.049	0.042	0.967	1.138	0.050	Imputed
rs4740844	9	6,424,566	T	C	0.206	0.200	0.312	1.043	0.042	0.961	1.132	0.058	Imputed
rs4740845	9	6,424,634	G	A	0.206	0.200	0.320	1.042	0.042	0.960	1.132	0.067	Imputed
rs2169286	9	6,425,256	C	A	0.154	0.150	0.460	1.035	0.047	0.944	1.135	0.103	Imputed
rs147715587	9	6,425,699	A	G	0.010	0.011	0.303	0.837	0.172	0.598	1.173	0.205	Imputed
rs10739099	9	6,427,481	T	A	0.176	0.173	0.587	1.024	0.044	0.939	1.117	0.033	Imputed
rs10739100	9	6,427,532	A	C	0.176	0.173	0.573	1.025	0.044	0.940	1.119	0.032	Imputed
rs10739101	9	6,427,663	T	C	0.053	0.050	0.435	1.061	0.076	0.914	1.231	0.659	Imputed
rs10739102	9	6,427,684	C	T	0.184	0.181	0.588	1.024	0.044	0.940	1.115	0.084	Imputed
rs7043824	9	6,427,721	T	C	0.053	0.050	0.461	1.058	0.076	0.911	1.227	0.574	Imputed
rs10758788	9	6,428,051	T	C	0.176	0.173	0.571	1.025	0.044	0.940	1.119	0.025	Imputed
rs7028825	9	6,428,575	T	C	0.106	0.107	0.909	0.994	0.055	0.892	1.107	0.018	Imputed
rs76402967	9	6,429,192	A	C	0.029	0.031	0.456	0.928	0.101	0.762	1.130	0.359	Imputed
rs10975587	9	6,429,259	A	G	0.233	0.244	0.184	0.948	0.040	0.877	1.026	0.500	Imputed
rs1381035	9	6,429,909	A	T	0.173	0.171	0.635	1.021	0.045	0.936	1.115	0.032	Imputed
rs1381036	9	6,429,924	A	G	0.174	0.171	0.624	1.022	0.045	0.937	1.116	0.031	Imputed
rs138563954	9	6,430,098	T	C	0.017	0.015	0.354	1.132	0.134	0.871	1.470	0.820	Imputed
rs1381037	9	6,430,156	G	A	0.180	0.177	0.580	1.025	0.044	0.940	1.117	0.071	Imputed
rs1824537	9	6,430,182	G	A	0.174	0.172	0.667	1.019	0.045	0.934	1.112	0.030	Imputed
rs4742200	9	6,430,254	T	C	0.053	0.050	0.471	1.056	0.076	0.910	1.226	0.555	Imputed
rs4742201	9	6,430,502	T	C	0.174	0.171	0.621	1.022	0.045	0.937	1.116	0.031	Imputed
rs4742202	9	6,430,598	A	G	0.174	0.171	0.621	1.022	0.045	0.937	1.116	0.031	Imputed
rs55765959	9	6,430,769	T	A	0.011	0.011	0.973	1.005	0.159	0.737	1.372	0.144	Imputed
rs10758789	9	6,431,091	T	G	0.174	0.171	0.600	1.024	0.045	0.938	1.117	0.030	Imputed
rs10758790	9	6,431,946	A	T	0.174	0.171	0.555	1.027	0.045	0.941	1.121	0.039	Imputed
rs10739103	9	6,432,156	C	A	0.052	0.050	0.514	1.051	0.076	0.905	1.220	0.579	Imputed
rs10739104	9	6,432,353	T	G	0.053	0.050	0.454	1.058	0.076	0.912	1.228	0.546	Genotyped
rs10975588	9	6,432,484	G	C	0.027	0.028	0.714	0.963	0.103	0.787	1.179	0.884	Imputed

rs56218473	9	6,432,790	A	G	0.121	0.122	0.955	1.003	0.052	0.906	1.110	0.039	Imputed
rs1903419	9	6,433,304	G	A	0.174	0.172	0.639	1.021	0.045	0.936	1.114	0.036	Imputed
rs142920247	9	6,434,525	C	G	0.021	0.019	0.562	1.071	0.118	0.849	1.351	0.740	Imputed
rs143948077	9	6,434,705	T	C	0.017	0.015	0.354	1.132	0.134	0.871	1.470	0.820	Imputed
rs10758791	9	6,435,559	C	T	0.177	0.176	0.818	1.010	0.044	0.926	1.102	0.033	Imputed
rs4742203	9	6,435,768	A	G	0.053	0.050	0.497	1.053	0.076	0.907	1.222	0.570	Imputed
rs4742204	9	6,435,819	C	G	0.175	0.172	0.601	1.023	0.044	0.938	1.117	0.041	Imputed
rs10758792	9	6,436,089	C	A	0.174	0.172	0.598	1.024	0.045	0.938	1.117	0.039	Imputed
rs7853059	9	6,436,138	G	A	0.122	0.121	0.901	1.006	0.052	0.910	1.114	0.046	Imputed
rs73401679	9	6,436,677	C	T	0.121	0.121	0.915	1.006	0.052	0.909	1.113	0.047	Imputed
rs7853834	9	6,436,779	T	A	0.121	0.121	0.925	1.005	0.052	0.908	1.112	0.047	Imputed
rs10815435	9	6,437,518	T	C	0.174	0.171	0.591	1.024	0.045	0.939	1.118	0.038	Imputed
rs7871134	9	6,437,689	G	C	0.174	0.171	0.591	1.024	0.045	0.939	1.118	0.038	Imputed
rs10491838	9	6,438,023	G	A	0.051	0.048	0.531	1.050	0.077	0.902	1.222	0.596	Imputed
rs380674	9	6,438,131	T	A	0.174	0.171	0.589	1.024	0.045	0.939	1.118	0.040	Imputed
rs118166192	9	6,438,439	T	C	0.028	0.029	0.779	0.972	0.102	0.795	1.188	0.806	Imputed
rs503315	9	6,439,075	T	C	0.053	0.050	0.471	1.056	0.076	0.910	1.226	0.555	Imputed
rs117829702	9	6,439,179	T	C	0.016	0.015	0.422	1.114	0.135	0.856	1.450	0.871	Imputed
rs414107	9	6,439,294	C	T	0.173	0.170	0.534	1.028	0.045	0.942	1.122	0.015	Imputed
rs10815436	9	6,439,587	G	A	0.268	0.249	0.015	1.098	0.038	1.019	1.184	0.318	Imputed
rs424119	9	6,439,943	G	A	0.053	0.050	0.519	1.050	0.076	0.905	1.219	0.582	Imputed
rs7033570	9	6,440,123	A	G	0.091	0.088	0.496	1.041	0.059	0.928	1.168	0.037	Genotyped
rs7033940	9	6,440,419	C	G	0.120	0.120	0.912	1.006	0.052	0.908	1.113	0.018	Imputed
rs394137	9	6,440,619	A	G	0.174	0.171	0.536	1.028	0.045	0.942	1.122	0.035	Imputed
rs452135	9	6,441,207	A	G	0.173	0.170	0.584	1.025	0.045	0.939	1.119	0.020	Imputed
rs117443648	9	6,441,733	T	C	0.015	0.017	0.552	0.922	0.137	0.704	1.206	0.789	Imputed
rs35301257	9	6,442,076	A	T	0.051	0.048	0.532	1.050	0.077	0.902	1.221	0.594	Imputed
rs553048	9	6,443,567	C	G	0.053	0.050	0.466	1.057	0.076	0.911	1.227	0.550	Imputed
rs183037891	9	6,444,673	T	C	0.016	0.015	0.431	1.112	0.134	0.854	1.447	0.877	Imputed
chr9:6445124:D	9	6,445,124	CT	C	0.011	0.013	0.439	0.884	0.159	0.648	1.207	0.678	Imputed
rs13293015	9	6,445,171	C	G	0.051	0.048	0.472	1.057	0.077	0.908	1.230	0.560	Imputed
rs11790625	9	6,445,304	T	C	0.148	0.145	0.534	1.030	0.048	0.938	1.131	0.069	Imputed
rs142677414	9	6,445,631	G	C	0.028	0.029	0.824	0.978	0.102	0.801	1.194	0.782	Imputed
rs147354178	9	6,445,667	T	C	0.061	0.067	0.143	0.903	0.070	0.787	1.036	0.133	Imputed
rs145329042	9	6,446,919	C	T	0.123	0.121	0.531	1.033	0.051	0.934	1.142	0.076	Imputed
rs7861559	9	6,447,140	T	C	0.201	0.195	0.358	1.040	0.042	0.957	1.129	0.062	Imputed
rs560059	9	6,447,196	G	A	0.266	0.244	2.73E-03	1.122	0.038	1.041	1.210	0.218	Imputed
rs7860966	9	6,447,394	A	G	0.200	0.195	0.373	1.038	0.042	0.956	1.128	0.053	Imputed
rs7861073	9	6,447,439	A	G	0.200	0.195	0.370	1.039	0.042	0.956	1.128	0.053	Imputed
rs563779	9	6,447,591	C	A	0.471	0.446	2.51E-03	1.108	0.034	1.037	1.184	0.565	Imputed

rs12346381	9	6,448,171	A	C	0.051	0.049	0.446	1.060	0.077	0.912	1.233	0.644	Imputed
rs56396114	9	6,448,582	C	G	0.148	0.146	0.630	1.023	0.048	0.932	1.123	0.081	Imputed
rs12351293	9	6,448,615	G	A	0.149	0.147	0.601	1.025	0.047	0.934	1.125	0.073	Imputed
rs12551361	9	6,448,621	T	G	0.208	0.181	4.91E-05	1.186	0.042	1.092	1.288	0.731	Imputed
rs13292799	9	6,448,764	G	A	0.196	0.192	0.443	1.033	0.043	0.950	1.123	0.081	Imputed
rs4742205	9	6,448,912	C	G	0.209	0.183	8.88E-05	1.178	0.042	1.085	1.279	0.605	Imputed
rs10124572	9	6,448,991	T	A	0.149	0.147	0.572	1.027	0.047	0.936	1.127	0.070	Imputed
rs34386249	9	6,449,243	G	A	0.017	0.017	0.981	1.003	0.130	0.778	1.294	0.951	Imputed
rs78602504	9	6,449,260	T	C	0.066	0.053	8.51E-04	1.259	0.069	1.099	1.442	0.524	Imputed
rs79793188	9	6,449,701	T	C	0.209	0.181	4.01E-05	1.188	0.042	1.094	1.290	0.763	Imputed
rs12554047	9	6,450,183	G	A	0.209	0.184	1.81E-04	1.170	0.042	1.077	1.270	0.694	Genotyped
rs16924562	9	6,450,551	T	G	0.149	0.146	0.537	1.030	0.048	0.938	1.130	0.069	Imputed
rs143032305	9	6,450,866	A	G	0.021	0.020	0.715	1.045	0.120	0.827	1.320	0.783	Imputed
rs7027361	9	6,451,088	A	G	0.408	0.377	1.10E-04	1.143	0.034	1.068	1.222	0.147	Imputed
chr9:6451181:I	9	6,451,181	GT	G	0.038	0.035	0.339	1.089	0.089	0.915	1.296	0.848	Imputed
rs427077	9	6,451,579	A	G	0.213	0.188	1.40E-04	1.171	0.042	1.080	1.271	0.960	Imputed
rs407351	9	6,451,638	T	A	0.211	0.184	4.61E-05	1.185	0.042	1.092	1.287	0.933	Imputed
rs12686562	9	6,451,751	A	G	0.209	0.184	2.57E-04	1.165	0.042	1.074	1.265	0.906	Imputed
rs13440427	9	6,451,847	C	G	0.148	0.147	0.655	1.021	0.048	0.931	1.121	0.084	Imputed
rs13284392	9	6,452,034	G	A	0.048	0.046	0.539	1.050	0.079	0.899	1.226	0.729	Imputed
rs34530211	9	6,452,525	G	A	0.051	0.048	0.481	1.056	0.077	0.908	1.229	0.640	Imputed
rs10975595	9	6,452,555	A	G	0.200	0.195	0.417	1.035	0.042	0.953	1.124	0.064	Imputed
rs36089474	9	6,452,673	C	T	0.051	0.048	0.481	1.056	0.077	0.908	1.229	0.640	Imputed
rs1870696	9	6,452,927	C	A	0.120	0.116	0.323	1.053	0.052	0.951	1.166	0.154	Imputed
rs13291494	9	6,453,939	G	A	0.051	0.048	0.520	1.051	0.077	0.903	1.223	0.663	Imputed
chr9:6454028:D	9	6,454,028	C	CT	0.199	0.195	0.424	1.034	0.042	0.952	1.124	0.071	Imputed
rs401733	9	6,455,048	T	C	0.405	0.376	2.55E-04	1.134	0.035	1.060	1.214	0.186	Genotyped
chr9:6455130:D	9	6,455,130	T	TTTTG	0.079	0.079	0.951	0.996	0.063	0.881	1.127	0.779	Imputed
rs111822258	9	6,455,277	G	A	0.149	0.147	0.569	1.027	0.047	0.936	1.127	0.056	Imputed
rs559438	9	6,455,445	C	A	0.013	0.015	0.555	0.917	0.146	0.689	1.222	0.876	Imputed
rs568386	9	6,455,777	G	A	0.464	0.437	8.72E-04	1.120	0.034	1.048	1.197	0.562	Imputed
chr9:6455790:I	9	6,455,790	G	GC	0.261	0.240	3.05E-03	1.121	0.039	1.040	1.210	0.274	Imputed
rs13302032	9	6,455,809	G	C	0.051	0.048	0.470	1.057	0.077	0.909	1.230	0.633	Imputed
rs13285152	9	6,456,081	A	G	0.051	0.048	0.470	1.057	0.077	0.909	1.230	0.633	Imputed
rs386632	9	6,456,155	G	A	0.263	0.241	2.68E-03	1.123	0.039	1.041	1.211	0.266	Imputed
rs370132	9	6,456,221	C	T	0.472	0.448	5.03E-03	1.100	0.034	1.029	1.175	0.311	Imputed
rs375835	9	6,456,230	A	T	0.470	0.446	5.91E-03	1.098	0.034	1.027	1.173	0.306	Imputed
rs13285458	9	6,456,258	A	G	0.051	0.048	0.466	1.058	0.077	0.909	1.231	0.630	Imputed
rs370818	9	6,456,266	G	T	0.262	0.240	3.24E-03	1.120	0.039	1.039	1.209	0.289	Imputed
rs2777945	9	6,457,102	A	G	0.464	0.437	7.09E-04	1.122	0.034	1.050	1.199	0.548	Imputed

chr9:6457392:D	9	6,457,392	T	TAAG	0.208	0.193	0.025	1.099	0.042	1.012	1.193	0.135	Imputed
rs4384035	9	6,457,938	G	A	0.151	0.148	0.415	1.039	0.047	0.947	1.140	0.061	Imputed
rs578124	9	6,458,054	G	A	0.314	0.290	1.67E-03	1.122	0.037	1.044	1.205	0.455	Imputed
rs7027302	9	6,458,236	T	G	0.151	0.148	0.519	1.031	0.047	0.940	1.131	0.073	Imputed
rs55960819	9	6,459,121	T	A	0.121	0.116	0.250	1.061	0.052	0.959	1.175	0.152	Imputed
chr9:6459220:D	9	6,459,220	A	AG	0.119	0.110	0.104	1.090	0.053	0.983	1.208	0.517	Imputed
rs7467926	9	6,459,221	A	G	0.263	0.240	1.76E-03	1.128	0.039	1.046	1.217	0.212	Imputed
rs524888	9	6,459,274	C	T	0.466	0.438	5.61E-04	1.124	0.034	1.052	1.201	0.611	Genotyped
rs513526	9	6,459,604	A	G	0.473	0.447	1.51E-03	1.113	0.034	1.042	1.190	0.380	Imputed
rs112036350	9	6,461,306	G	C	0.018	0.018	0.992	1.001	0.127	0.781	1.284	0.849	Imputed
rs13285688	9	6,461,678	T	C	0.041	0.039	0.650	1.040	0.086	0.879	1.230	0.842	Imputed
rs13284998	9	6,461,911	A	G	0.035	0.033	0.556	1.056	0.092	0.881	1.266	0.828	Imputed
rs535725	9	6,462,346	C	T	0.472	0.448	3.64E-03	1.104	0.034	1.033	1.179	0.621	Imputed
rs16924591	9	6,462,585	T	C	0.121	0.117	0.306	1.054	0.052	0.953	1.167	0.144	Genotyped
rs530722	9	6,462,813	T	C	0.204	0.199	0.355	1.040	0.042	0.957	1.129	0.046	Imputed
rs185465898	9	6,463,044	C	G	0.010	0.011	0.564	0.907	0.170	0.650	1.264	0.751	Imputed
rs504698	9	6,463,435	A	G	0.203	0.199	0.401	1.036	0.042	0.954	1.125	0.048	Imputed
rs79309408	9	6,463,547	G	T	0.149	0.146	0.546	1.029	0.047	0.938	1.129	0.067	Imputed
rs76552338	9	6,465,342	G	T	0.121	0.116	0.255	1.061	0.052	0.958	1.174	0.120	Imputed
rs541378	9	6,465,465	G	A	0.412	0.382	1.85E-04	1.137	0.034	1.063	1.217	0.195	Imputed
rs569866	9	6,466,285	C	A	0.203	0.199	0.407	1.035	0.042	0.954	1.124	0.057	Imputed
rs145719533	9	6,466,294	C	T	0.011	0.011	0.912	0.982	0.162	0.715	1.350	0.319	Imputed
rs184028792	9	6,466,535	G	A	0.010	0.010	0.646	0.925	0.171	0.662	1.292	0.906	Imputed
rs11791651	9	6,467,989	C	T	0.151	0.149	0.589	1.026	0.047	0.935	1.125	0.081	Imputed
rs117597069	9	6,468,307	C	T	0.030	0.029	0.854	1.019	0.100	0.837	1.239	0.249	Imputed
rs3739654	9	6,468,447	G	A	0.203	0.198	0.413	1.035	0.042	0.953	1.124	0.054	Imputed
rs72693587	9	6,469,163	G	T	0.052	0.049	0.511	1.052	0.077	0.905	1.222	0.507	Imputed
rs4556138	9	6,469,486	C	T	0.203	0.198	0.387	1.037	0.042	0.955	1.126	0.049	Imputed
rs12346455	9	6,469,651	G	A	0.203	0.198	0.421	1.034	0.042	0.953	1.123	0.044	Genotyped
chr9:6469703:D	9	6,469,703	T	TAC	0.029	0.028	0.716	1.038	0.102	0.850	1.267	0.675	Imputed
rs35408061	9	6,469,738	G	A	0.052	0.049	0.500	1.053	0.077	0.906	1.224	0.501	Imputed
rs4740846	9	6,469,793	C	T	0.051	0.040	8.30E-04	1.297	0.078	1.113	1.511	0.560	Imputed
rs13296481	9	6,469,937	C	T	0.052	0.049	0.500	1.053	0.077	0.906	1.224	0.501	Imputed
rs7869419	9	6,469,964	G	T	0.052	0.050	0.461	1.058	0.076	0.911	1.228	0.647	Imputed
rs6477086	9	6,470,232	G	A	0.203	0.198	0.411	1.035	0.042	0.953	1.124	0.052	Imputed
rs4380997	9	6,470,639	G	A	0.151	0.149	0.602	1.025	0.047	0.934	1.124	0.079	Imputed
rs7020390	9	6,470,921	G	A	0.471	0.447	3.73E-03	1.103	0.034	1.032	1.179	0.624	Imputed
rs74898627	9	6,471,797	A	G	0.040	0.043	0.467	0.940	0.086	0.794	1.112	0.821	Imputed
rs34581400	9	6,472,112	G	T	0.052	0.049	0.479	1.056	0.077	0.908	1.227	0.489	Imputed
rs12347573	9	6,472,503	A	G	0.186	0.182	0.501	1.030	0.044	0.946	1.121	0.058	Imputed

rs6477087	9	6,472,714	A	C	0.185	0.182	0.496	1.030	0.044	0.946	1.122	0.058	Genotyped
rs13283464	9	6,473,103	C	A	0.035	0.034	0.758	1.029	0.092	0.859	1.231	0.462	Imputed
rs13283687	9	6,473,296	G	A	0.052	0.049	0.452	1.059	0.077	0.912	1.231	0.476	Imputed
rs7873756	9	6,473,409	A	G	0.202	0.197	0.386	1.037	0.042	0.955	1.127	0.063	Imputed
rs7860627	9	6,473,630	G	A	0.267	0.249	0.014	1.099	0.038	1.019	1.185	0.228	Imputed
rs7852539	9	6,475,319	A	G	0.202	0.197	0.388	1.037	0.042	0.955	1.126	0.067	Imputed
rs13299896	9	6,475,633	G	T	0.052	0.049	0.539	1.048	0.077	0.902	1.218	0.521	Imputed
rs73399080	9	6,475,805	G	A	0.148	0.145	0.519	1.031	0.048	0.939	1.132	0.079	Imputed
rs7872349	9	6,475,893	C	T	0.037	0.037	0.987	0.999	0.090	0.838	1.190	0.618	Imputed
rs78689655	9	6,475,899	G	T	0.036	0.036	0.980	0.998	0.091	0.834	1.193	0.886	Imputed
rs10975603	9	6,476,213	G	A	0.148	0.145	0.519	1.031	0.048	0.939	1.132	0.079	Imputed
rs118131406	9	6,476,330	G	C	0.027	0.029	0.327	0.903	0.105	0.736	1.108	0.297	Imputed
rs7876000	9	6,476,496	C	T	0.148	0.145	0.518	1.031	0.048	0.939	1.132	0.079	Imputed
chr9:6477233:I	9	6,477,233	CA	C	0.008	0.012	0.030	0.676	0.183	0.472	0.967	0.078	Imputed
rs7034328	9	6,477,350	A	C	0.120	0.115	0.263	1.060	0.052	0.957	1.174	0.129	Imputed
chr9:6477525:I	9	6,477,525	TC	T	0.035	0.035	0.843	1.018	0.092	0.851	1.219	0.497	Imputed
rs73399085	9	6,477,791	T	C	0.027	0.029	0.422	0.921	0.103	0.752	1.127	0.347	Imputed
rs13291472	9	6,478,065	G	A	0.186	0.183	0.512	1.029	0.043	0.945	1.120	0.075	Imputed
rs62566152	9	6,479,068	G	A	0.164	0.169	0.462	0.967	0.046	0.884	1.057	0.568	Imputed
rs10122116	9	6,479,215	T	C	0.151	0.148	0.536	1.030	0.047	0.939	1.130	0.110	Imputed
rs10125914	9	6,479,464	C	T	0.151	0.148	0.538	1.030	0.047	0.938	1.129	0.107	Imputed
rs11795355	9	6,479,531	C	T	0.149	0.145	0.491	1.033	0.048	0.941	1.134	0.091	Imputed
rs10115883	9	6,479,626	A	G	0.151	0.148	0.536	1.030	0.047	0.939	1.130	0.110	Imputed
rs10975605	9	6,480,005	A	G	0.151	0.148	0.540	1.029	0.047	0.938	1.129	0.111	Imputed
rs113506777	9	6,480,139	G	T	0.016	0.016	0.878	1.021	0.134	0.785	1.327	0.766	Imputed
rs4463484	9	6,480,382	T	C	0.150	0.148	0.561	1.028	0.047	0.937	1.128	0.114	Imputed
rs16924624	9	6,480,538	C	A	0.151	0.148	0.523	1.031	0.047	0.939	1.131	0.108	Imputed
rs67797327	9	6,480,658	A	C	0.034	0.033	0.749	1.030	0.094	0.857	1.238	0.566	Imputed
rs16924626	9	6,480,726	C	G	0.150	0.148	0.558	1.028	0.047	0.937	1.128	0.114	Imputed
rs13289925	9	6,483,120	G	A	0.183	0.180	0.516	1.029	0.044	0.944	1.121	0.108	Imputed
rs13290488	9	6,483,440	G	A	0.035	0.035	0.733	1.032	0.092	0.862	1.234	0.577	Imputed
rs143172497	9	6,483,444	A	G	0.022	0.021	0.696	1.046	0.116	0.834	1.312	0.980	Imputed
rs13294924	9	6,484,019	G	C	0.035	0.035	0.733	1.032	0.092	0.862	1.234	0.577	Imputed
rs13296692	9	6,484,689	G	A	0.034	0.033	0.704	1.036	0.094	0.863	1.245	0.699	Imputed
chr9:6484778:I	9	6,484,778	CTTCT	C	0.145	0.142	0.515	1.032	0.048	0.939	1.133	0.071	Imputed
chr9:6484787:I	9	6,484,787	TTC	T	0.145	0.142	0.515	1.032	0.048	0.939	1.133	0.071	Imputed
chr9:6484788:I	9	6,484,788	TC	T	0.145	0.142	0.498	1.033	0.048	0.940	1.135	0.072	Imputed
rs11795228	9	6,484,789	C	T	0.121	0.122	0.966	0.998	0.052	0.902	1.104	0.094	Imputed
rs75813410	9	6,485,925	A	G	0.041	0.043	0.556	0.951	0.085	0.805	1.123	0.917	Imputed
rs16924631	9	6,486,308	C	G	0.147	0.145	0.589	1.026	0.048	0.935	1.127	0.088	Imputed

rs13291887	9	6,486,539	C	T	0.035	0.035	0.703	1.035	0.092	0.865	1.239	0.563	Imputed
rs66509816	9	6,486,591	G	A	0.149	0.146	0.552	1.029	0.048	0.937	1.129	0.071	Imputed
rs34905133	9	6,487,393	G	T	0.035	0.035	0.756	1.029	0.092	0.860	1.231	0.522	Imputed
rs34529113	9	6,487,462	G	A	0.036	0.035	0.633	1.044	0.090	0.875	1.247	0.673	Imputed
chr9:6488822:I	9	6,488,822	GT	G	0.038	0.037	0.740	1.030	0.088	0.866	1.224	0.633	Imputed
rs7875172	9	6,489,209	G	A	0.036	0.035	0.619	1.046	0.090	0.876	1.249	0.665	Imputed
rs13288875	9	6,489,597	C	T	0.036	0.035	0.585	1.051	0.091	0.879	1.256	0.627	Imputed
chr9:6490014:D	9	6,490,014	C	CAG	0.038	0.036	0.571	1.052	0.089	0.884	1.251	0.790	Imputed
rs10739105	9	6,490,248	A	G	0.154	0.163	0.110	0.928	0.047	0.847	1.017	0.654	Genotyped
rs73401017	9	6,490,301	C	G	0.028	0.030	0.480	0.931	0.102	0.761	1.137	0.321	Imputed
rs13300059	9	6,490,342	G	A	0.036	0.035	0.585	1.051	0.091	0.879	1.256	0.627	Imputed
rs113626350	9	6,490,491	A	G	0.034	0.032	0.562	1.056	0.094	0.878	1.271	0.059	Imputed
rs13285125	9	6,490,861	G	T	0.036	0.035	0.569	1.053	0.090	0.882	1.257	0.710	Imputed
rs13283631	9	6,491,191	C	G	0.036	0.035	0.585	1.051	0.091	0.879	1.256	0.627	Imputed
rs13285035	9	6,491,371	T	C	0.037	0.035	0.513	1.060	0.090	0.890	1.264	0.810	Genotyped
rs13285949	9	6,491,535	G	A	0.036	0.035	0.569	1.053	0.090	0.882	1.257	0.710	Imputed
rs13290126	9	6,492,715	A	G	0.036	0.035	0.606	1.048	0.091	0.877	1.253	0.638	Imputed
rs73401025	9	6,492,741	C	T	0.148	0.146	0.604	1.025	0.048	0.934	1.125	0.104	Imputed
rs117882882	9	6,492,761	T	C	0.027	0.029	0.327	0.903	0.105	0.736	1.108	0.297	Imputed
chr9:6493294:I	9	6,493,294	TC	T	0.154	0.151	0.507	1.031	0.047	0.941	1.131	0.043	Imputed
rs79198297	9	6,493,295	C	T	0.174	0.169	0.342	1.043	0.045	0.956	1.139	0.184	Imputed
rs13296970	9	6,493,297	C	A	0.174	0.169	0.342	1.043	0.045	0.956	1.139	0.184	Imputed
rs13286720	9	6,494,216	C	T	0.036	0.035	0.585	1.051	0.091	0.879	1.256	0.627	Imputed
chr9:6494289:I	9	6,494,289	TC	T	0.010	0.013	0.091	0.755	0.167	0.544	1.046	0.289	Imputed
rs143243762	9	6,494,579	G	T	0.012	0.013	0.710	0.945	0.152	0.702	1.273	0.439	Imputed
rs16924634	9	6,495,789	A	C	0.120	0.115	0.272	1.059	0.052	0.956	1.173	0.198	Genotyped
rs1563	9	6,495,905	G	A	0.036	0.035	0.583	1.051	0.091	0.880	1.256	0.627	Imputed
chr9:6495998:D	9	6,495,998	T	TA	0.026	0.026	0.906	1.013	0.107	0.822	1.248	0.257	Imputed
rs55988623	9	6,496,064	G	A	0.072	0.076	0.391	0.945	0.065	0.832	1.075	0.326	Imputed
rs10119350	9	6,496,811	C	T	0.037	0.036	0.659	1.040	0.090	0.872	1.241	0.599	Imputed
rs7029772	9	6,497,589	T	A	0.185	0.180	0.346	1.042	0.044	0.957	1.135	0.083	Imputed
rs41281765	9	6,498,287	T	C	0.019	0.016	0.238	1.159	0.126	0.907	1.483	0.546	Imputed
rs13284032	9	6,498,676	T	G	0.036	0.035	0.634	1.044	0.091	0.874	1.248	0.584	Imputed
rs117579654	9	6,498,773	A	C	0.023	0.023	0.998	1.000	0.114	0.800	1.249	0.239	Imputed
rs1929929	9	6,499,994	T	C	0.120	0.113	0.162	1.076	0.052	0.971	1.191	0.276	Imputed
rs13292030	9	6,501,212	G	A	0.036	0.035	0.612	1.047	0.091	0.877	1.251	0.574	Imputed
rs10975611	9	6,502,279	A	T	0.279	0.263	0.034	1.084	0.038	1.006	1.167	0.176	Imputed
rs148219930	9	6,502,601	T	C	0.012	0.012	0.843	1.031	0.156	0.760	1.399	0.223	Imputed
rs13298500	9	6,502,612	T	A	0.157	0.150	0.161	1.067	0.047	0.974	1.169	0.233	Imputed
rs13283381	9	6,503,517	G	C	0.185	0.181	0.424	1.035	0.044	0.951	1.128	0.122	Imputed

rs190700657	9	6,503,556	A	G	0.014	0.013	0.627	1.074	0.147	0.806	1.432	0.800	Imputed

Table S2e NHRNPA3P1-LOC100130539 region on chromosome 10

SNP	CHR	BP	Allele 1	Allele 2	F_A	F_U	P cmh test	OR cmh	SE	L95	U95	P het	Method
rs2005093	10	44,291,660	A	G	0.094	0.095	0.860	0.990	0.058	0.884	1.109	0.350	Imputed
rs2902564	10	44,291,716	G	A	0.133	0.137	0.435	0.962	0.050	0.873	1.060	0.792	Imputed
rs2005094	10	44,291,719	A	G	0.105	0.107	0.542	0.967	0.055	0.868	1.077	0.826	Imputed
rs59039806	10	44,291,756	A	G	0.324	0.331	0.369	0.968	0.036	0.902	1.039	0.778	Imputed
rs2009557	10	44,291,763	C	T	0.324	0.331	0.368	0.968	0.036	0.902	1.039	0.727	Imputed
rs915421	10	44,291,766	A	G	0.143	0.138	0.374	1.044	0.048	0.950	1.148	0.576	Imputed
rs61528945	10	44,291,884	T	C	0.028	0.030	0.630	0.952	0.102	0.780	1.163	0.324	Imputed
rs751466	10	44,292,188	G	C	0.097	0.097	0.771	0.984	0.057	0.879	1.100	0.520	Imputed
rs751464	10	44,292,364	C	T	0.193	0.194	0.837	0.991	0.043	0.911	1.078	0.748	Genotyped
rs74993456	10	44,292,441	T	C	0.086	0.087	0.872	0.990	0.060	0.880	1.115	0.348	Imputed
chr10:44293131:D	10	44,293,131	G	GTATT	0.035	0.035	0.984	0.998	0.093	0.833	1.197	0.206	Imputed
rs7077599	10	44,293,482	C	T	0.303	0.306	0.642	0.983	0.037	0.915	1.057	0.964	Imputed
rs10899911	10	44,293,839	A	G	0.277	0.278	0.752	0.988	0.038	0.918	1.064	0.700	Genotyped
rs61861585	10	44,293,962	T	G	0.105	0.108	0.582	0.970	0.055	0.871	1.081	0.779	Imputed
rs4948777	10	44,294,030	C	T	0.306	0.309	0.685	0.985	0.037	0.917	1.059	0.947	Imputed
chr10:44294039:D	10	44,294,039	C	CCTT	0.029	0.030	0.759	0.969	0.101	0.795	1.182	0.309	Imputed
rs4948778	10	44,294,226	C	T	0.306	0.309	0.690	0.986	0.037	0.917	1.059	0.930	Imputed
rs76585778	10	44,294,344	G	A	0.072	0.069	0.468	1.049	0.066	0.922	1.192	0.741	Imputed
rs4948779	10	44,294,515	C	G	0.171	0.170	0.928	1.004	0.045	0.920	1.096	0.871	Imputed
rs7910172	10	44,294,699	C	T	0.307	0.309	0.690	0.986	0.037	0.917	1.059	0.924	Imputed
rs7910184	10	44,294,740	C	T	0.307	0.309	0.690	0.986	0.037	0.917	1.059	0.924	Imputed
rs6593368	10	44,294,814	A	G	0.307	0.309	0.684	0.985	0.037	0.917	1.059	0.921	Imputed
rs11238713	10	44,295,539	C	T	0.089	0.092	0.487	0.960	0.059	0.855	1.078	0.498	Genotyped
rs1886723	10	44,296,028	T	C	0.213	0.223	0.098	0.934	0.041	0.862	1.013	0.549	Imputed
chr10:44296840:D	10	44,296,840	A	AAAC	0.027	0.028	0.878	0.984	0.104	0.803	1.206	0.303	Imputed
rs10899912	10	44,296,893	A	G	0.088	0.091	0.413	0.953	0.060	0.848	1.070	0.509	Genotyped
rs79977099	10	44,297,002	T	G	0.027	0.027	0.868	0.983	0.105	0.799	1.208	0.338	Imputed
rs74394503	10	44,297,019	T	C	0.078	0.072	0.247	1.076	0.064	0.950	1.219	0.815	Imputed
rs10899913	10	44,297,179	G	A	0.390	0.398	0.183	0.955	0.035	0.892	1.022	0.718	Imputed
rs77736210	10	44,297,433	A	G	0.077	0.072	0.287	1.070	0.064	0.945	1.212	0.893	Imputed
rs2863760	10	44,297,579	G	A	0.387	0.397	0.158	0.952	0.035	0.890	1.019	0.739	Imputed
rs10899914	10	44,297,933	T	C	0.088	0.091	0.439	0.955	0.060	0.850	1.073	0.508	Imputed
rs10899915	10	44,298,421	T	C	0.088	0.091	0.469	0.958	0.059	0.853	1.076	0.476	Imputed
rs12360144	10	44,298,636	T	C	0.214	0.225	0.075	0.929	0.041	0.857	1.007	0.482	Imputed

rs12357048	10	44,298,644	A	G	0.215	0.226	0.097	0.934	0.041	0.862	1.012	0.481	Genotyped
rs12360468	10	44,298,868	T	C	0.188	0.198	0.085	0.928	0.043	0.853	1.010	0.701	Genotyped
rs76142590	10	44,298,984	C	G	0.077	0.072	0.259	1.075	0.064	0.948	1.218	0.894	Imputed
rs12254433	10	44,299,045	C	G	0.389	0.398	0.192	0.956	0.035	0.893	1.023	0.766	Imputed
rs55874105	10	44,299,236	A	G	0.214	0.226	0.067	0.927	0.041	0.855	1.005	0.503	Imputed
rs10899916	10	44,299,320	T	C	0.389	0.398	0.190	0.956	0.035	0.893	1.023	0.763	Imputed
rs7093062	10	44,299,610	A	G	0.089	0.091	0.540	0.964	0.059	0.859	1.083	0.438	Imputed
chr10:44299738:D	10	44,299,738	C	CAA	0.009	0.014	5.32E-03	0.612	0.178	0.432	0.868	0.883	Imputed
chr10:44299768:D	10	44,299,768	A	AAAT	0.019	0.018	0.638	1.062	0.126	0.829	1.359	0.044	Imputed
rs75843051	10	44,300,131	A	C	0.027	0.028	0.807	0.975	0.105	0.793	1.198	0.347	Imputed
chr10:44300345:D	10	44,300,345	A	AC	0.122	0.133	0.066	0.910	0.052	0.822	1.007	0.266	Imputed
rs61861587	10	44,302,302	C	T	0.066	0.077	8.73E-03	0.837	0.068	0.733	0.956	0.529	Imputed
rs76538344	10	44,302,532	T	C	0.072	0.069	0.549	1.040	0.066	0.915	1.183	0.633	Imputed
rs17154436	10	44,302,830	A	G	0.027	0.028	0.798	0.973	0.105	0.792	1.196	0.344	Imputed
rs4245610	10	44,302,862	G	T	0.385	0.395	0.129	0.949	0.035	0.886	1.015	0.616	Imputed
rs7922745	10	44,303,219	C	T	0.385	0.395	0.117	0.947	0.035	0.885	1.014	0.610	Genotyped
rs61861588	10	44,303,415	A	T	0.188	0.198	0.075	0.926	0.043	0.851	1.008	0.676	Imputed
rs10899917	10	44,303,616	G	C	0.385	0.395	0.120	0.947	0.035	0.885	1.014	0.615	Imputed
rs17463850	10	44,303,667	G	A	0.101	0.104	0.519	0.965	0.056	0.864	1.077	0.815	Imputed
rs1540966	10	44,303,920	C	T	0.385	0.395	0.126	0.948	0.035	0.886	1.015	0.625	Imputed
rs1540967	10	44,303,930	T	C	0.082	0.078	0.432	1.050	0.062	0.930	1.186	0.510	Genotyped
rs61861589	10	44,304,009	T	C	0.066	0.076	9.95E-03	0.840	0.068	0.735	0.959	0.504	Imputed
rs1540968	10	44,304,097	C	T	0.385	0.395	0.126	0.948	0.035	0.886	1.015	0.625	Imputed
rs1537796	10	44,304,222	A	C	0.385	0.395	0.126	0.948	0.035	0.886	1.015	0.625	Imputed
rs78931007	10	44,304,600	C	A	0.072	0.069	0.457	1.050	0.066	0.923	1.194	0.639	Imputed
rs76233217	10	44,305,409	A	G	0.019	0.022	0.320	0.887	0.122	0.699	1.126	0.004	Imputed
rs7916028	10	44,305,716	A	C	0.381	0.390	0.172	0.954	0.035	0.891	1.021	0.688	Imputed
rs10793499	10	44,305,838	G	C	0.090	0.091	0.683	0.976	0.059	0.869	1.096	0.523	Imputed
rs4948579	10	44,307,024	G	A	0.330	0.337	0.312	0.964	0.036	0.899	1.035	0.326	Imputed
rs7905888	10	44,307,202	G	A	0.330	0.335	0.357	0.967	0.036	0.902	1.038	0.344	Imputed
rs7920120	10	44,307,275	A	G	0.081	0.077	0.463	1.047	0.062	0.926	1.183	0.450	Imputed
rs7906426	10	44,307,607	G	A	0.121	0.116	0.423	1.043	0.052	0.941	1.155	0.310	Imputed
rs141448275	10	44,307,658	A	G	0.016	0.015	0.598	1.073	0.134	0.825	1.396	0.646	Imputed
rs7900485	10	44,307,687	T	G	0.119	0.114	0.379	1.047	0.052	0.945	1.161	0.379	Imputed
rs7917583	10	44,307,693	G	A	0.119	0.114	0.379	1.047	0.052	0.945	1.161	0.379	Imputed
rs7920802	10	44,307,717	A	T	0.119	0.114	0.379	1.047	0.052	0.945	1.161	0.379	Imputed
rs7900896	10	44,308,000	A	G	0.329	0.334	0.372	0.968	0.036	0.902	1.039	0.325	Genotyped
rs11238718	10	44,308,204	C	T	0.328	0.334	0.340	0.966	0.036	0.900	1.037	0.334	Imputed
rs77711378	10	44,308,602	A	G	0.067	0.065	0.797	1.018	0.068	0.891	1.162	0.722	Imputed
chr10:44308627:D	10	44,308,627	A	AG	0.130	0.130	0.978	0.999	0.050	0.905	1.102	0.817	Imputed

rs11238719	10	44,308,718	G	A	0.065	0.061	0.355	1.066	0.069	0.931	1.221	0.706	Imputed
rs76321579	10	44,308,784	G	A	0.101	0.100	0.861	1.010	0.056	0.905	1.128	0.872	Imputed
rs12220592	10	44,308,978	A	G	0.142	0.143	0.944	0.997	0.048	0.906	1.096	0.477	Imputed
rs28475171	10	44,309,073	G	A	0.140	0.150	0.070	0.916	0.049	0.832	1.007	0.775	Imputed
rs28654354	10	44,309,107	G	C	0.140	0.150	0.059	0.912	0.049	0.829	1.003	0.748	Imputed
rs74138087	10	44,309,155	A	T	0.038	0.040	0.550	0.949	0.088	0.799	1.127	0.008	Imputed
rs12264397	10	44,309,461	A	G	0.035	0.035	0.713	0.967	0.092	0.806	1.159	0.544	Imputed
rs61861590	10	44,309,677	C	G	0.075	0.084	0.035	0.874	0.064	0.771	0.991	0.825	Imputed
rs79900593	10	44,310,560	C	T	0.052	0.053	0.903	0.991	0.076	0.854	1.150	0.528	Imputed
rs12411389	10	44,311,711	A	G	0.108	0.111	0.493	0.964	0.054	0.866	1.072	0.099	Imputed
rs7083186	10	44,311,824	C	T	0.155	0.163	0.168	0.938	0.047	0.856	1.028	0.977	Imputed
rs118167625	10	44,312,050	T	C	0.009	0.010	0.450	0.874	0.176	0.620	1.234	0.023	Imputed
rs7079939	10	44,312,106	C	A	0.132	0.141	0.090	0.919	0.050	0.834	1.013	0.817	Imputed
rs58009513	10	44,312,195	A	T	0.019	0.018	0.711	1.047	0.124	0.821	1.336	0.545	Imputed
rs61071701	10	44,312,229	T	A	0.019	0.018	0.711	1.047	0.124	0.821	1.336	0.545	Imputed
rs17154467	10	44,312,301	T	C	0.019	0.018	0.711	1.047	0.124	0.821	1.336	0.545	Imputed
rs1414491	10	44,312,358	G	A	0.151	0.160	0.132	0.932	0.047	0.849	1.022	0.978	Imputed
rs4146640	10	44,312,843	T	C	0.340	0.333	0.308	1.037	0.036	0.967	1.112	0.397	Imputed
rs11238720	10	44,313,122	G	C	0.137	0.145	0.104	0.923	0.049	0.839	1.017	0.931	Imputed
rs11238721	10	44,313,156	G	C	0.132	0.141	0.093	0.920	0.050	0.834	1.014	0.804	Imputed
rs56887761	10	44,313,313	C	T	0.019	0.018	0.700	1.049	0.125	0.822	1.339	0.541	Imputed
rs1144486	10	44,313,417	G	T	0.215	0.222	0.263	0.955	0.041	0.881	1.035	0.450	Imputed
rs7071492	10	44,313,732	T	C	0.129	0.138	0.099	0.920	0.050	0.834	1.016	0.525	Imputed
rs7913725	10	44,314,431	C	A	0.150	0.159	0.105	0.926	0.047	0.844	1.016	0.872	Genotyped
rs78110873	10	44,314,614	A	G	0.053	0.053	0.820	0.983	0.076	0.848	1.140	0.585	Imputed
rs140552456	10	44,314,709	T	C	0.047	0.047	0.897	0.990	0.080	0.846	1.158	0.151	Imputed
rs79564327	10	44,314,736	C	T	0.019	0.018	0.765	1.038	0.125	0.812	1.327	0.567	Imputed
chr10:44314872:D	10	44,314,872	G	GGGAGGCCGA	0.018	0.018	0.831	1.027	0.126	0.803	1.315	0.595	Imputed
rs1563885	10	44,314,893	T	C	0.404	0.401	0.638	1.016	0.034	0.950	1.087	0.862	Imputed
rs79966190	10	44,314,973	A	G	0.019	0.018	0.670	1.054	0.125	0.826	1.346	0.527	Imputed
rs2863237	10	44,314,976	A	G	0.412	0.407	0.519	1.022	0.034	0.956	1.094	0.918	Imputed
rs4522124	10	44,314,985	C	G	0.412	0.407	0.519	1.022	0.034	0.956	1.094	0.918	Imputed
rs11238722	10	44,315,371	G	A	0.412	0.406	0.451	1.026	0.034	0.959	1.098	0.912	Genotyped
rs11238723	10	44,315,580	A	G	0.150	0.158	0.130	0.931	0.047	0.849	1.021	0.862	Imputed
rs75899883	10	44,315,843	A	G	0.019	0.018	0.670	1.054	0.125	0.826	1.346	0.527	Imputed
rs7069081	10	44,316,331	T	C	0.430	0.426	0.664	1.015	0.034	0.949	1.085	0.918	Imputed
rs7080911	10	44,316,351	A	G	0.413	0.407	0.481	1.025	0.034	0.958	1.096	0.922	Imputed
rs75206944	10	44,316,447	T	A	0.078	0.086	0.059	0.888	0.063	0.786	1.005	0.593	Imputed
chr10:44316703:D	10	44,316,703	C	CT	0.024	0.028	0.174	0.862	0.109	0.697	1.068	0.751	Imputed
rs57438040	10	44,316,984	T	A	0.019	0.018	0.670	1.054	0.125	0.826	1.346	0.527	Imputed

rs12264669	10	44,317,048	C	T	0.131	0.140	0.075	0.915	0.050	0.829	1.009	0.977	Imputed
rs7086449	10	44,317,118	A	C	0.127	0.137	0.064	0.911	0.051	0.825	1.005	0.774	Imputed
rs142732313	10	44,317,157	T	C	0.011	0.013	0.402	0.874	0.160	0.639	1.196	0.578	Imputed
rs75285815	10	44,317,272	T	C	0.052	0.053	0.715	0.973	0.076	0.838	1.129	0.637	Imputed
rs34552744	10	44,317,319	A	G	0.090	0.088	0.637	1.028	0.059	0.916	1.155	0.345	Imputed
rs7087019	10	44,317,521	G	C	0.131	0.140	0.075	0.915	0.050	0.829	1.009	0.977	Imputed
rs57376602	10	44,318,029	G	T	0.019	0.018	0.660	1.056	0.125	0.828	1.348	0.523	Imputed
rs12266918	10	44,318,235	C	T	0.131	0.140	0.080	0.916	0.050	0.831	1.011	0.952	Imputed
rs57697881	10	44,318,343	A	G	0.019	0.018	0.660	1.056	0.125	0.828	1.348	0.523	Imputed
rs79293109	10	44,318,589	T	A	0.050	0.052	0.666	0.967	0.077	0.831	1.125	0.352	Imputed
rs60207525	10	44,319,471	A	G	0.019	0.018	0.660	1.056	0.125	0.828	1.348	0.523	Imputed
rs59298548	10	44,319,834	T	G	0.019	0.018	0.660	1.056	0.125	0.828	1.348	0.523	Imputed
rs7075076	10	44,319,891	T	C	0.079	0.087	0.059	0.889	0.063	0.786	1.005	0.570	Genotyped
rs7096797	10	44,320,304	C	T	0.078	0.086	0.060	0.889	0.063	0.786	1.005	0.617	Imputed
rs111799357	10	44,320,751	T	A	0.027	0.025	0.449	1.083	0.106	0.881	1.333	0.746	Imputed
chr10:44320789:D	10	44,320,789	G	GC	0.128	0.136	0.114	0.923	0.050	0.836	1.019	0.933	Imputed
rs2863236	10	44,320,813	T	C	0.130	0.138	0.106	0.922	0.050	0.836	1.017	0.929	Imputed
rs2863235	10	44,320,817	T	G	0.132	0.140	0.113	0.924	0.050	0.838	1.019	0.875	Imputed
rs2863234	10	44,321,156	T	C	0.143	0.151	0.112	0.926	0.048	0.843	1.018	0.822	Imputed
rs76022111	10	44,321,157	T	G	0.020	0.021	0.740	0.961	0.122	0.757	1.219	0.596	Imputed
rs2863233	10	44,321,162	A	T	0.139	0.149	0.083	0.919	0.049	0.835	1.011	0.786	Imputed
rs76795752	10	44,321,166	G	A	0.019	0.019	0.813	1.030	0.124	0.808	1.312	0.821	Imputed
rs61859378	10	44,321,288	G	A	0.159	0.169	0.112	0.929	0.046	0.849	1.017	0.979	Imputed
rs10899918	10	44,321,351	T	G	0.400	0.396	0.545	1.021	0.035	0.954	1.093	0.468	Imputed
rs79327290	10	44,321,520	T	C	0.024	0.021	0.334	1.115	0.112	0.894	1.389	0.898	Imputed
rs78380092	10	44,321,584	G	C	0.014	0.013	0.512	1.098	0.143	0.830	1.452	0.578	Imputed
rs12570694	10	44,321,629	A	G	0.051	0.053	0.594	0.960	0.076	0.827	1.115	0.020	Genotyped
rs59912226	10	44,321,649	T	C	0.018	0.014	0.043	1.304	0.131	1.009	1.685	0.289	Imputed
rs112266781	10	44,321,692	T	C	0.021	0.017	0.101	1.218	0.121	0.962	1.543	0.672	Imputed
rs1870633	10	44,321,753	C	T	0.446	0.441	0.501	1.023	0.034	0.957	1.094	0.825	Imputed
rs1870632	10	44,321,786	T	C	0.446	0.441	0.551	1.021	0.034	0.955	1.091	0.776	Imputed
rs10793500	10	44,322,048	T	C	0.412	0.406	0.464	1.026	0.034	0.959	1.097	0.689	Imputed
rs10793501	10	44,322,100	G	T	0.422	0.418	0.509	1.023	0.034	0.957	1.094	0.776	Imputed
rs79172649	10	44,322,427	C	T	0.010	0.011	0.790	0.956	0.169	0.686	1.332	0.533	Imputed
rs729247	10	44,322,446	A	T	0.412	0.407	0.432	1.027	0.034	0.961	1.099	0.710	Imputed
rs912804	10	44,322,539	A	G	0.412	0.407	0.432	1.027	0.034	0.961	1.099	0.710	Imputed
rs117856840	10	44,323,578	T	C	0.022	0.026	0.182	0.857	0.115	0.684	1.073	0.003	Imputed
rs1147898	10	44,324,490	T	A	0.082	0.084	0.822	0.986	0.062	0.874	1.113	0.425	Imputed
rs4948780	10	44,324,537	T	C	0.413	0.407	0.408	1.029	0.034	0.962	1.101	0.620	Imputed
rs7094300	10	44,324,621	G	A	0.413	0.407	0.414	1.028	0.034	0.962	1.100	0.616	Imputed

rs76985588	10	44,325,377	C	T	0.010	0.011	0.790	0.956	0.169	0.686	1.332	0.533	Imputed
rs11238725	10	44,325,795	A	G	0.416	0.411	0.471	1.025	0.034	0.958	1.096	0.805	Imputed
rs61859379	10	44,325,941	G	A	0.146	0.154	0.114	0.927	0.048	0.844	1.018	0.980	Imputed
rs11238726	10	44,326,688	T	C	0.432	0.429	0.676	1.014	0.034	0.949	1.085	0.740	Imputed
rs10899919	10	44,327,128	G	A	0.413	0.407	0.442	1.027	0.034	0.960	1.098	0.623	Imputed
rs78973389	10	44,327,192	A	G	0.420	0.412	0.294	1.037	0.034	0.969	1.109	0.825	Imputed
rs189730164	10	44,327,234	C	T	0.052	0.054	0.665	0.968	0.076	0.834	1.123	0.663	Imputed
rs144056674	10	44,327,422	T	C	0.080	0.080	0.827	1.014	0.062	0.897	1.146	0.366	Imputed
rs138650915	10	44,327,423	A	G	0.052	0.053	0.687	0.970	0.076	0.835	1.126	0.631	Imputed
rs11238728	10	44,327,434	G	A	0.413	0.408	0.434	1.027	0.034	0.960	1.099	0.608	Imputed
rs11238729	10	44,327,625	T	C	0.411	0.407	0.554	1.021	0.034	0.954	1.092	0.505	Imputed
rs11238730	10	44,327,714	C	T	0.426	0.423	0.639	1.016	0.034	0.950	1.087	0.864	Imputed
rs117682209	10	44,328,286	C	G	0.010	0.011	0.790	0.956	0.169	0.686	1.332	0.533	Imputed
rs142522109	10	44,328,564	T	C	0.127	0.140	0.015	0.884	0.051	0.801	0.976	0.655	Imputed
rs60581994	10	44,328,897	T	G	0.010	0.010	0.895	0.978	0.172	0.698	1.369	0.619	Imputed
chr10:44329000:D	10	44,329,000	A	AG	0.010	0.010	0.989	1.002	0.172	0.716	1.404	0.576	Imputed
rs10899920	10	44,329,188	T	C	0.423	0.418	0.453	1.026	0.034	0.959	1.097	0.729	Imputed
rs11238731	10	44,329,329	T	C	0.414	0.408	0.453	1.026	0.034	0.959	1.098	0.614	Imputed
rs146228222	10	44,329,525	A	T	0.097	0.106	0.069	0.902	0.057	0.807	1.008	0.652	Imputed
rs117046105	10	44,329,569	T	C	0.144	0.153	0.084	0.920	0.048	0.838	1.011	0.997	Imputed
rs148434212	10	44,329,743	T	C	0.145	0.153	0.102	0.925	0.048	0.842	1.016	0.996	Imputed
rs11528509	10	44,329,961	T	C	0.423	0.418	0.435	1.027	0.034	0.960	1.098	0.741	Imputed
rs11238732	10	44,330,094	C	T	0.423	0.418	0.434	1.027	0.034	0.961	1.098	0.741	Imputed
rs118027007	10	44,330,156	A	G	0.034	0.034	0.987	0.999	0.093	0.832	1.198	0.037	Imputed
rs11238733	10	44,330,276	C	G	0.422	0.414	0.271	1.038	0.034	0.971	1.111	0.547	Imputed
rs2863232	10	44,330,344	A	G	0.354	0.348	0.400	1.030	0.035	0.961	1.104	0.314	Imputed
rs2147873	10	44,330,428	T	C	0.414	0.408	0.401	1.029	0.034	0.962	1.101	0.643	Imputed
rs113406493	10	44,330,862	C	T	0.052	0.053	0.687	0.970	0.076	0.835	1.126	0.631	Imputed
rs7913475	10	44,330,934	T	C	0.414	0.408	0.401	1.029	0.034	0.962	1.101	0.643	Imputed
rs149030202	10	44,331,285	A	T	0.010	0.011	0.790	0.956	0.169	0.686	1.332	0.533	Imputed
rs7914001	10	44,331,289	T	G	0.375	0.371	0.502	1.024	0.035	0.956	1.096	0.446	Imputed
rs188561064	10	44,331,555	C	G	0.010	0.010	0.723	1.060	0.166	0.766	1.469	0.137	Imputed
rs4350323	10	44,331,679	T	G	0.414	0.407	0.383	1.030	0.034	0.963	1.102	0.643	Imputed
rs61859384	10	44,331,702	G	A	0.069	0.074	0.189	0.916	0.067	0.804	1.044	0.956	Imputed
rs4429020	10	44,331,803	A	G	0.414	0.408	0.401	1.029	0.034	0.962	1.101	0.643	Imputed
rs12358602	10	44,331,876	C	G	0.431	0.428	0.686	1.014	0.034	0.948	1.084	0.738	Imputed
rs2085798	10	44,332,080	C	T	0.414	0.407	0.383	1.030	0.034	0.963	1.102	0.643	Imputed
rs112097644	10	44,332,168	C	T	0.060	0.061	0.893	0.991	0.071	0.862	1.138	0.288	Imputed
rs12357341	10	44,332,372	G	A	0.431	0.428	0.698	1.013	0.034	0.948	1.084	0.744	Imputed
rs1841390	10	44,332,603	G	A	0.145	0.153	0.101	0.924	0.048	0.842	1.016	0.988	Imputed

rs77211970	10	44,333,255	G	T	0.052	0.053	0.687	0.970	0.076	0.835	1.126	0.631	Imputed
rs12219425	10	44,333,491	A	T	0.424	0.418	0.419	1.028	0.034	0.961	1.099	0.731	Imputed
rs11238734	10	44,334,916	T	G	0.145	0.153	0.105	0.925	0.048	0.842	1.016	0.999	Genotyped
rs4948781	10	44,335,013	A	C	0.413	0.407	0.375	1.031	0.034	0.964	1.103	0.635	Imputed
rs4948782	10	44,335,184	T	C	0.353	0.347	0.384	1.031	0.035	0.962	1.106	0.304	Imputed
rs4948783	10	44,335,193	A	G	0.414	0.408	0.423	1.028	0.034	0.961	1.100	0.654	Imputed
rs79746840	10	44,335,562	A	G	0.052	0.053	0.677	0.969	0.076	0.835	1.125	0.583	Imputed
rs80307761	10	44,335,624	T	C	0.046	0.047	0.948	0.995	0.081	0.849	1.166	0.203	Imputed
rs59084049	10	44,335,980	G	A	0.010	0.011	0.879	0.975	0.167	0.702	1.353	0.503	Imputed
rs7901526	10	44,336,374	A	C	0.479	0.486	0.397	0.972	0.034	0.909	1.038	0.524	Genotyped
rs34759112	10	44,336,734	C	T	0.058	0.052	0.138	1.114	0.073	0.966	1.285	0.367	Imputed
rs147322823	10	44,336,865	G	A	0.022	0.026	0.149	0.846	0.116	0.674	1.060	0.006	Imputed
rs11238736	10	44,337,098	T	G	0.427	0.425	0.784	1.009	0.034	0.944	1.079	0.717	Imputed
rs1870636	10	44,337,215	T	G	0.451	0.444	0.345	1.033	0.034	0.966	1.104	0.995	Imputed
rs10899921	10	44,337,481	T	C	0.432	0.429	0.726	1.012	0.034	0.947	1.082	0.719	Imputed
rs117030511	10	44,337,768	T	C	0.010	0.010	0.867	1.029	0.170	0.737	1.436	0.533	Imputed
rs76720082	10	44,337,849	A	G	0.010	0.010	0.867	1.029	0.170	0.737	1.436	0.533	Imputed
rs78957593	10	44,337,870	T	C	0.010	0.010	0.867	1.029	0.170	0.737	1.436	0.533	Imputed
rs11238737	10	44,338,110	A	G	0.431	0.429	0.749	1.011	0.034	0.946	1.081	0.732	Imputed
rs61859386	10	44,338,283	T	C	0.135	0.148	0.016	0.888	0.049	0.807	0.978	0.873	Imputed
rs4948784	10	44,338,535	A	G	0.451	0.444	0.384	1.030	0.034	0.964	1.101	0.991	Imputed
rs112414482	10	44,338,565	T	C	0.051	0.053	0.678	0.969	0.077	0.834	1.126	0.663	Imputed
rs7918642	10	44,338,657	G	A	0.060	0.060	0.974	0.998	0.071	0.868	1.147	0.237	Imputed
rs74459788	10	44,338,664	A	G	0.010	0.010	0.867	1.029	0.170	0.737	1.436	0.533	Imputed
rs187635443	10	44,339,108	A	G	0.010	0.010	0.723	1.060	0.166	0.766	1.469	0.137	Imputed
rs41531444	10	44,339,185	A	G	0.051	0.053	0.678	0.969	0.077	0.834	1.126	0.663	Imputed
rs1675291	10	44,339,315	T	C	0.178	0.166	0.043	1.094	0.044	1.003	1.194	0.851	Imputed
rs11238738	10	44,339,418	G	A	0.461	0.455	0.404	1.029	0.034	0.963	1.099	0.906	Imputed
rs59021054	10	44,339,590	T	G	0.010	0.010	0.867	1.029	0.170	0.737	1.436	0.533	Imputed
rs58426522	10	44,339,911	G	C	0.010	0.010	0.867	1.029	0.170	0.737	1.436	0.533	Imputed
rs78964431	10	44,340,062	T	C	0.051	0.053	0.678	0.969	0.077	0.834	1.126	0.663	Imputed
rs118052809	10	44,340,072	A	T	0.027	0.020	0.010	1.318	0.108	1.068	1.627	0.691	Imputed
rs74862729	10	44,340,199	G	A	0.010	0.010	0.867	1.029	0.170	0.737	1.436	0.533	Imputed
rs11238739	10	44,340,493	T	C	0.451	0.444	0.372	1.031	0.034	0.964	1.102	0.985	Imputed
chr10:44340783:I	10	44,340,783	GC	G	0.060	0.060	0.986	0.999	0.071	0.869	1.148	0.234	Imputed
chr10:44340788:I	10	44,340,788	CA	C	0.050	0.052	0.462	0.945	0.077	0.812	1.100	0.211	Imputed
rs4948785	10	44,340,865	A	G	0.451	0.444	0.374	1.031	0.034	0.964	1.102	0.987	Imputed
chr10:44341072:D	10	44,341,072	C	CATTGATT	0.431	0.429	0.740	1.011	0.034	0.946	1.081	0.727	Imputed
rs187919802	10	44,341,415	C	T	0.010	0.010	0.867	1.029	0.170	0.737	1.436	0.533	Imputed
rs12783842	10	44,341,534	G	A	0.027	0.025	0.399	1.092	0.104	0.890	1.339	0.701	Imputed

rs10899922	10	44,341,964	G	A	0.461	0.455	0.394	1.029	0.034	0.963	1.100	0.911	Genotyped
rs75436290	10	44,342,040	A	G	0.010	0.010	0.837	1.036	0.170	0.742	1.445	0.587	Imputed
rs118085865	10	44,342,135	C	T	0.010	0.010	0.867	1.029	0.170	0.737	1.436	0.533	Imputed
rs73275930	10	44,342,146	A	T	0.051	0.053	0.655	0.966	0.077	0.832	1.123	0.648	Imputed
rs12241117	10	44,342,352	A	G	0.018	0.018	0.929	1.011	0.125	0.791	1.293	0.031	Imputed
rs58280468	10	44,342,733	C	T	0.010	0.010	0.837	1.036	0.170	0.742	1.445	0.587	Imputed
chr10:44342953:I	10	44,342,953	CTTTA	C	0.010	0.010	0.837	1.036	0.170	0.742	1.445	0.587	Imputed
rs748715	10	44,343,087	T	C	0.424	0.418	0.496	1.024	0.034	0.957	1.095	0.915	Imputed
rs78628306	10	44,343,169	T	C	0.010	0.010	0.837	1.036	0.170	0.742	1.445	0.587	Imputed
rs77100799	10	44,343,384	T	C	0.053	0.054	0.960	0.996	0.075	0.860	1.154	0.837	Imputed
chr10:44343939:I	10	44,343,939	AATCCATCCATCC	A	0.011	0.011	0.956	1.009	0.164	0.732	1.391	0.451	Imputed
rs74873320	10	44,344,612	A	G	0.010	0.010	0.595	1.093	0.167	0.788	1.515	0.830	Imputed
rs11238740	10	44,344,644	C	T	0.019	0.014	0.022	1.335	0.126	1.043	1.710	0.244	Imputed
rs10793503	10	44,344,738	C	T	0.446	0.439	0.424	1.028	0.034	0.961	1.098	0.819	Genotyped
chr10:44344885:I	10	44,344,885	AT	A	0.080	0.081	0.983	0.999	0.062	0.884	1.129	0.439	Imputed
rs78197031	10	44,345,134	G	A	0.054	0.055	0.718	0.973	0.075	0.841	1.127	0.760	Imputed
rs74420036	10	44,345,353	T	C	0.046	0.046	0.882	1.012	0.081	0.863	1.186	0.110	Imputed
rs80145431	10	44,345,430	G	C	0.046	0.046	0.882	1.012	0.081	0.863	1.186	0.110	Imputed
rs75148678	10	44,345,432	T	C	0.010	0.010	0.866	1.029	0.170	0.737	1.436	0.668	Imputed
rs75900646	10	44,345,821	G	A	0.010	0.010	0.866	1.029	0.170	0.737	1.436	0.668	Imputed
rs1254846	10	44,346,094	G	A	0.070	0.074	0.446	0.951	0.066	0.835	1.082	0.126	Genotyped
rs117769578	10	44,346,388	G	C	0.010	0.010	0.880	1.026	0.170	0.735	1.432	0.673	Imputed
rs2125978	10	44,346,594	C	T	0.019	0.014	0.034	1.309	0.127	1.021	1.678	0.240	Imputed
rs61110542	10	44,346,863	G	A	0.010	0.010	0.880	1.026	0.170	0.735	1.432	0.673	Imputed
rs60606176	10	44,346,883	A	G	0.010	0.010	0.880	1.026	0.170	0.735	1.432	0.673	Imputed
rs7909314	10	44,347,063	A	G	0.454	0.450	0.637	1.016	0.034	0.951	1.086	0.724	Imputed
rs77452812	10	44,347,103	A	G	0.054	0.056	0.740	0.976	0.074	0.843	1.129	0.664	Imputed
rs4948580	10	44,347,246	T	A	0.484	0.476	0.321	1.034	0.034	0.968	1.105	0.865	Imputed
rs10793504	10	44,347,717	T	A	0.407	0.415	0.264	0.962	0.034	0.900	1.029	0.932	Imputed
rs11238741	10	44,347,970	T	C	0.473	0.465	0.320	1.034	0.034	0.968	1.105	0.959	Imputed
rs1468061	10	44,348,098	A	G	0.454	0.450	0.636	1.016	0.034	0.951	1.086	0.727	Imputed
rs59274145	10	44,348,379	A	G	0.010	0.010	0.880	1.026	0.170	0.735	1.432	0.673	Imputed
rs78226429	10	44,349,049	T	C	0.010	0.010	0.880	1.026	0.170	0.735	1.432	0.673	Imputed
rs74828131	10	44,349,238	A	G	0.010	0.010	0.880	1.026	0.170	0.735	1.432	0.673	Imputed
rs80183712	10	44,349,483	T	C	0.010	0.010	0.880	1.026	0.170	0.735	1.432	0.673	Imputed
rs17464560	10	44,349,516	G	C	0.454	0.450	0.636	1.016	0.034	0.951	1.086	0.727	Imputed
rs78129090	10	44,349,580	A	G	0.010	0.010	0.880	1.026	0.170	0.735	1.432	0.673	Imputed
rs79694172	10	44,349,940	C	T	0.011	0.012	0.435	0.880	0.164	0.639	1.213	0.864	Imputed
rs17154506	10	44,350,037	G	A	0.055	0.056	0.829	0.984	0.074	0.851	1.138	0.619	Genotyped
chr10:44350064:I	10	44,350,064	G	GACTTT	0.431	0.436	0.385	0.971	0.034	0.908	1.038	0.946	Imputed

rs78158539	10	44,350,143	G	T	0.010	0.010	0.880	1.026	0.170	0.735	1.432	0.673	Imputed
rs1078113	10	44,350,464	C	G	0.410	0.417	0.278	0.963	0.034	0.901	1.031	0.871	Imputed
rs57882812	10	44,351,049	G	C	0.010	0.010	0.880	1.026	0.170	0.735	1.432	0.673	Imputed
rs140930168	10	44,351,176	T	C	0.010	0.012	0.199	0.803	0.171	0.575	1.122	0.685	Imputed
rs74858657	10	44,351,355	A	G	0.010	0.010	0.760	1.052	0.166	0.759	1.458	0.906	Imputed
rs116978589	10	44,351,411	A	T	0.010	0.010	0.880	1.026	0.170	0.735	1.432	0.673	Imputed
rs113327206	10	44,351,488	T	G	0.032	0.037	0.084	0.848	0.096	0.703	1.023	0.716	Imputed
rs76750991	10	44,351,665	A	G	0.012	0.011	0.501	1.111	0.157	0.817	1.511	0.822	Imputed
rs78554189	10	44,352,094	C	T	0.010	0.011	0.936	1.014	0.168	0.730	1.408	0.559	Imputed
rs118162120	10	44,352,374	C	T	0.046	0.046	0.938	1.006	0.081	0.859	1.180	0.123	Imputed
rs115566767	10	44,352,463	A	G	0.010	0.011	0.936	1.014	0.168	0.730	1.408	0.559	Imputed
rs4948786	10	44,352,569	A	G	0.462	0.460	0.717	1.012	0.034	0.947	1.082	0.687	Imputed
chr10:44352571:I	10	44,352,571	AAG	A	0.010	0.011	0.936	1.014	0.168	0.730	1.408	0.559	Imputed
rs881714	10	44,353,871	A	C	0.453	0.450	0.672	1.015	0.034	0.949	1.084	0.734	Imputed
rs78336318	10	44,354,068	T	G	0.054	0.056	0.746	0.976	0.074	0.844	1.129	0.661	Imputed
rs61859388	10	44,354,542	C	A	0.054	0.055	0.903	0.991	0.074	0.856	1.147	0.509	Imputed
rs713496	10	44,355,400	T	C	0.010	0.011	0.936	1.014	0.168	0.730	1.408	0.559	Imputed
rs11238745	10	44,355,590	G	C	0.045	0.045	0.989	1.001	0.082	0.853	1.175	0.094	Imputed
rs79958406	10	44,356,010	T	G	0.010	0.010	0.880	1.026	0.170	0.735	1.432	0.673	Imputed
rs76846594	10	44,356,323	C	T	0.010	0.011	0.936	1.014	0.168	0.730	1.408	0.559	Imputed
rs72781106	10	44,356,609	A	T	0.023	0.026	0.194	0.863	0.113	0.692	1.078	0.550	Imputed
rs185490731	10	44,356,718	G	A	0.017	0.016	0.797	1.035	0.133	0.798	1.342	0.150	Imputed
rs58838440	10	44,356,828	A	G	0.010	0.011	0.950	1.011	0.168	0.727	1.404	0.564	Imputed
rs59983288	10	44,357,126	G	T	0.010	0.011	0.936	1.014	0.168	0.730	1.408	0.559	Imputed
rs58526299	10	44,357,192	G	C	0.010	0.011	0.936	1.014	0.168	0.730	1.408	0.559	Imputed
rs59668941	10	44,357,249	G	A	0.010	0.011	0.936	1.014	0.168	0.730	1.408	0.559	Imputed
rs78981046	10	44,357,453	T	A	0.018	0.014	0.046	1.297	0.130	1.005	1.673	0.223	Imputed
rs10899923	10	44,357,854	C	T	0.464	0.462	0.699	1.013	0.034	0.948	1.083	0.639	Imputed
rs61525746	10	44,358,117	A	C	0.010	0.011	0.936	1.014	0.168	0.730	1.408	0.559	Imputed
rs2169200	10	44,358,219	A	C	0.454	0.450	0.625	1.017	0.034	0.951	1.087	0.708	Genotyped
rs1008803	10	44,358,300	G	A	0.463	0.461	0.658	1.015	0.034	0.950	1.085	0.643	Genotyped
rs1008804	10	44,358,550	G	A	0.408	0.416	0.238	0.960	0.034	0.898	1.027	0.954	Imputed
rs148469743	10	44,358,708	T	C	0.022	0.026	0.087	0.821	0.115	0.655	1.029	0.027	Imputed
rs77383005	10	44,358,738	T	C	0.020	0.015	0.016	1.352	0.125	1.059	1.726	0.252	Imputed
rs75419445	10	44,358,747	A	G	0.019	0.015	0.028	1.318	0.126	1.030	1.687	0.291	Imputed
rs11238747	10	44,359,262	C	T	0.045	0.045	0.960	0.996	0.082	0.848	1.170	0.088	Imputed
rs10899924	10	44,359,632	T	C	0.408	0.416	0.247	0.961	0.034	0.898	1.028	0.999	Imputed
rs78028152	10	44,359,999	A	G	0.040	0.042	0.435	0.935	0.086	0.790	1.107	0.146	Imputed
rs77229889	10	44,360,132	G	T	0.010	0.011	0.936	1.014	0.168	0.730	1.408	0.559	Imputed
rs79466182	10	44,360,297	T	C	0.010	0.011	0.936	1.014	0.168	0.730	1.408	0.559	Imputed

rs9633744	10	44,360,599	C	A	0.464	0.462	0.666	1.015	0.034	0.950	1.085	0.622	Imputed
rs78359121	10	44,360,748	C	T	0.055	0.056	0.755	0.977	0.074	0.845	1.130	0.656	Imputed
rs187682205	10	44,360,819	A	G	0.013	0.018	0.018	0.704	0.149	0.526	0.943	0.503	Imputed
rs1009771	10	44,361,106	G	A	0.464	0.462	0.653	1.015	0.034	0.950	1.085	0.611	Imputed
rs1009770	10	44,361,284	T	C	0.453	0.450	0.633	1.016	0.034	0.951	1.086	0.714	Imputed
chr10:44361314:D	10	44,361,314	G	GC	0.010	0.010	0.806	1.042	0.168	0.750	1.449	0.513	Imputed
rs184139035	10	44,361,315	A	C	0.010	0.010	0.776	1.049	0.168	0.754	1.458	0.567	Imputed
rs189205953	10	44,361,316	G	A	0.010	0.010	0.776	1.049	0.168	0.754	1.458	0.567	Imputed
rs1009769	10	44,361,352	C	T	0.464	0.461	0.611	1.017	0.034	0.952	1.087	0.631	Imputed
rs117574853	10	44,361,403	A	T	0.024	0.024	0.984	1.002	0.110	0.808	1.243	0.016	Imputed
rs75728939	10	44,361,562	T	C	0.010	0.010	0.939	1.013	0.170	0.726	1.413	0.696	Imputed
rs11238748	10	44,361,608	A	G	0.404	0.413	0.212	0.958	0.034	0.895	1.025	0.982	Imputed
rs75376675	10	44,361,948	G	A	0.028	0.026	0.259	1.123	0.103	0.918	1.373	0.954	Imputed
rs11238749	10	44,362,231	T	C	0.453	0.450	0.626	1.017	0.034	0.951	1.087	0.722	Imputed
rs11238750	10	44,362,266	A	G	0.453	0.450	0.626	1.017	0.034	0.951	1.087	0.709	Genotyped
chr10:44362593:D	10	44,362,593	A	AT	0.011	0.011	0.918	0.983	0.164	0.713	1.355	0.740	Imputed
rs60833858	10	44,362,681	G	T	0.010	0.011	0.932	0.986	0.170	0.707	1.374	0.605	Imputed
rs7921870	10	44,362,711	T	C	0.404	0.413	0.212	0.958	0.034	0.895	1.025	0.984	Imputed
rs60927183	10	44,362,721	T	C	0.010	0.011	0.932	0.986	0.170	0.707	1.374	0.605	Imputed
rs79664857	10	44,362,779	T	A	0.025	0.017	1.78E-03	1.418	0.112	1.139	1.767	0.349	Imputed
rs997193	10	44,363,286	T	C	0.160	0.168	0.178	0.940	0.046	0.859	1.029	0.559	Imputed
rs146311176	10	44,363,429	G	C	0.019	0.024	0.054	0.788	0.124	0.618	1.003	0.032	Imputed
rs79823602	10	44,364,477	A	G	0.010	0.011	0.918	0.983	0.170	0.705	1.370	0.611	Imputed
rs11238751	10	44,364,641	A	G	0.018	0.013	0.018	1.360	0.130	1.054	1.756	0.405	Imputed
rs912805	10	44,364,706	C	T	0.464	0.462	0.644	1.016	0.034	0.951	1.086	0.623	Imputed
rs57645083	10	44,364,804	C	T	0.011	0.012	0.924	0.985	0.162	0.717	1.352	0.436	Imputed
rs57678939	10	44,364,875	A	G	0.054	0.056	0.699	0.972	0.074	0.840	1.124	0.685	Imputed
rs2031513	10	44,365,019	G	T	0.453	0.450	0.626	1.017	0.034	0.951	1.087	0.722	Imputed
rs4948581	10	44,365,238	G	C	0.464	0.462	0.644	1.016	0.034	0.951	1.086	0.623	Imputed
rs76777017	10	44,365,368	T	C	0.010	0.011	0.918	0.983	0.170	0.705	1.370	0.611	Imputed
rs10899925	10	44,365,540	C	T	0.465	0.462	0.645	1.016	0.034	0.950	1.086	0.623	Imputed
rs79332046	10	44,365,656	C	T	0.010	0.010	0.910	1.019	0.170	0.731	1.422	0.684	Imputed
rs1547163	10	44,365,715	A	G	0.404	0.413	0.218	0.958	0.034	0.896	1.025	0.856	Imputed
rs73275973	10	44,365,762	A	C	0.011	0.012	0.924	0.985	0.162	0.717	1.352	0.436	Imputed
rs193301865	10	44,366,185	G	A	0.056	0.058	0.552	0.957	0.073	0.829	1.105	0.833	Imputed
rs59230148	10	44,366,402	A	G	0.054	0.056	0.699	0.972	0.074	0.840	1.124	0.685	Imputed
rs2985833	10	44,366,558	T	C	0.015	0.013	0.168	1.214	0.141	0.921	1.599	0.704	Imputed
rs12359360	10	44,366,565	C	T	0.472	0.472	0.879	1.005	0.034	0.941	1.074	0.711	Imputed
rs146028854	10	44,366,737	A	G	0.010	0.012	0.234	0.818	0.169	0.588	1.138	0.789	Imputed
rs11238753	10	44,366,841	A	G	0.454	0.450	0.599	1.018	0.034	0.952	1.088	0.708	Imputed

rs12778827	10	44,367,114	T	C	0.028	0.026	0.259	1.123	0.103	0.918	1.373	0.954	Imputed
rs11238754	10	44,367,155	T	C	0.454	0.450	0.588	1.019	0.034	0.953	1.089	0.716	Imputed
rs11238755	10	44,367,267	A	C	0.479	0.483	0.459	0.975	0.034	0.913	1.042	0.967	Imputed
rs11238756	10	44,367,287	C	T	0.465	0.462	0.613	1.017	0.034	0.952	1.087	0.609	Imputed
rs75486433	10	44,367,818	T	C	0.010	0.011	0.918	0.983	0.170	0.705	1.370	0.611	Imputed
rs73275981	10	44,368,138	C	T	0.011	0.012	0.937	0.987	0.162	0.719	1.356	0.432	Imputed
rs10899926	10	44,368,244	T	C	0.057	0.055	0.620	1.037	0.073	0.898	1.198	0.241	Imputed
rs55718913	10	44,368,307	C	T	0.012	0.012	0.811	0.963	0.158	0.706	1.312	0.003	Imputed
rs59658376	10	44,368,366	A	G	0.019	0.017	0.361	1.120	0.125	0.877	1.430	0.130	Imputed
chr10:44368736:I	10	44,368,736	TA	T	0.054	0.056	0.699	0.972	0.074	0.840	1.124	0.685	Imputed
rs56942692	10	44,369,284	A	G	0.011	0.012	0.924	0.985	0.162	0.717	1.352	0.436	Imputed
rs10899927	10	44,369,899	T	C	0.454	0.450	0.596	1.018	0.034	0.953	1.088	0.708	Imputed
rs10899928	10	44,369,906	G	A	0.465	0.461	0.587	1.019	0.034	0.953	1.089	0.595	Imputed
rs7070116	10	44,370,167	A	G	0.409	0.416	0.332	0.967	0.034	0.904	1.035	0.965	Imputed
rs73275989	10	44,370,483	C	A	0.011	0.012	0.951	0.990	0.162	0.721	1.359	0.428	Imputed
rs73275990	10	44,370,723	G	T	0.011	0.012	0.951	0.990	0.162	0.721	1.359	0.428	Imputed
rs11238759	10	44,370,755	C	T	0.454	0.450	0.577	1.019	0.034	0.954	1.089	0.697	Imputed
rs11238761	10	44,370,872	C	T	0.454	0.450	0.575	1.019	0.034	0.954	1.089	0.695	Imputed
rs118076558	10	44,371,229	T	C	0.010	0.010	0.895	1.023	0.170	0.733	1.427	0.679	Imputed
rs145986044	10	44,371,263	A	G	0.012	0.012	0.824	0.965	0.158	0.708	1.315	0.004	Imputed
rs138765708	10	44,371,307	T	A	0.026	0.024	0.414	1.091	0.106	0.886	1.344	0.921	Imputed
rs141818853	10	44,371,316	C	T	0.049	0.049	0.989	0.999	0.079	0.856	1.165	0.066	Imputed
rs2298204	10	44,371,674	G	A	0.010	0.011	0.991	1.002	0.168	0.721	1.392	0.517	Imputed
rs148712598	10	44,371,696	C	T	0.015	0.014	0.728	1.051	0.142	0.796	1.387	0.393	Imputed
chr10:44371782:I	10	44,371,782	GT	G	0.463	0.459	0.563	1.020	0.034	0.954	1.090	0.590	Imputed
rs7903927	10	44,371,903	G	A	0.463	0.460	0.590	1.018	0.034	0.953	1.088	0.580	Imputed
rs78275993	10	44,372,082	T	C	0.010	0.011	0.932	0.986	0.170	0.707	1.374	0.605	Imputed
rs75944252	10	44,372,085	T	A	0.010	0.011	0.932	0.986	0.170	0.707	1.374	0.605	Imputed
rs141044783	10	44,372,161	A	G	0.009	0.012	0.170	0.787	0.175	0.559	1.109	0.914	Imputed
rs79700343	10	44,372,223	T	C	0.010	0.010	0.895	1.023	0.170	0.733	1.427	0.679	Imputed
rs73275995	10	44,372,318	A	G	0.011	0.012	0.979	0.996	0.162	0.725	1.367	0.472	Imputed
rs11238762	10	44,373,079	A	C	0.014	0.011	0.134	1.249	0.149	0.934	1.671	0.700	Imputed
rs12242985	10	44,373,172	A	G	0.056	0.055	0.678	1.031	0.074	0.893	1.191	0.224	Imputed
rs10899929	10	44,373,192	A	G	0.468	0.461	0.345	1.033	0.034	0.966	1.103	0.782	Imputed
rs73275997	10	44,373,377	C	T	0.011	0.011	0.980	1.004	0.162	0.731	1.379	0.408	Imputed
rs73275998	10	44,373,540	T	C	0.011	0.011	0.966	1.007	0.162	0.733	1.383	0.404	Imputed
rs78155419	10	44,373,721	T	C	0.010	0.010	0.852	1.032	0.170	0.740	1.441	0.593	Imputed
rs10899930	10	44,373,858	A	G	0.057	0.055	0.630	1.036	0.073	0.897	1.196	0.188	Genotyped
rs10899931	10	44,374,085	A	C	0.056	0.055	0.643	1.035	0.074	0.896	1.195	0.218	Imputed
rs10899932	10	44,374,702	A	G	0.477	0.473	0.560	1.020	0.034	0.954	1.090	0.561	Imputed

rs74138916	10	44,374,786	A	G	0.010	0.011	0.830	0.965	0.165	0.698	1.334	0.414	Imputed
rs7920511	10	44,374,899	T	C	0.441	0.447	0.354	0.969	0.034	0.906	1.036	0.966	Genotyped
chr10:44374973:D	10	44,374,973	C	CCG	0.050	0.050	0.958	1.004	0.078	0.862	1.170	0.087	Imputed
rs11238764	10	44,375,162	T	C	0.478	0.475	0.665	1.015	0.034	0.950	1.084	0.541	Imputed
rs2613102	10	44,375,239	T	C	0.084	0.086	0.663	0.974	0.061	0.864	1.097	0.204	Genotyped
rs7901541	10	44,375,264	T	C	0.050	0.051	0.912	0.991	0.078	0.851	1.155	0.096	Imputed
rs79453258	10	44,375,320	T	C	0.010	0.010	0.937	0.986	0.172	0.704	1.381	0.540	Imputed
rs10751346	10	44,375,543	C	T	0.442	0.448	0.391	0.971	0.034	0.909	1.038	0.936	Imputed
rs7076199	10	44,375,605	A	G	0.125	0.136	0.033	0.897	0.051	0.812	0.991	0.919	Imputed
rs10899933	10	44,375,635	T	C	0.059	0.061	0.808	0.983	0.072	0.854	1.131	0.132	Genotyped
rs59834212	10	44,375,676	A	G	0.010	0.010	0.923	0.984	0.172	0.702	1.377	0.545	Imputed
rs10899934	10	44,375,680	G	C	0.059	0.061	0.848	0.986	0.072	0.857	1.135	0.143	Imputed
rs17465611	10	44,375,711	A	C	0.491	0.485	0.501	1.023	0.034	0.957	1.093	0.702	Imputed
chr10:44375778:D	10	44,375,778	T	TG	0.059	0.061	0.897	0.991	0.072	0.861	1.140	0.191	Imputed
rs191019429	10	44,375,781	T	G	0.021	0.025	0.083	0.816	0.118	0.648	1.028	0.463	Imputed
rs11238765	10	44,375,912	T	G	0.049	0.049	0.949	1.005	0.079	0.861	1.173	0.059	Imputed
rs10899935	10	44,375,925	T	G	0.051	0.051	0.986	0.999	0.077	0.859	1.162	0.087	Imputed
rs10899936	10	44,376,051	G	A	0.050	0.051	0.897	0.990	0.078	0.850	1.154	0.085	Imputed
rs7921967	10	44,376,205	A	G	0.447	0.450	0.560	0.980	0.034	0.917	1.048	0.962	Imputed
rs10899937	10	44,376,756	T	C	0.492	0.486	0.435	1.027	0.034	0.961	1.097	0.668	Genotyped
rs7073044	10	44,376,927	C	T	0.059	0.061	0.910	0.992	0.072	0.862	1.141	0.150	Imputed
rs72781126	10	44,377,080	A	G	0.007	0.011	0.044	0.673	0.198	0.456	0.993	0.352	Imputed
rs117253976	10	44,377,795	G	A	0.026	0.024	0.518	1.071	0.107	0.869	1.321	0.789	Imputed
chr10:44378266:I	10	44,378,266	AT	A	0.061	0.058	0.449	1.055	0.071	0.918	1.213	0.120	Imputed
rs72781128	10	44,378,684	T	G	0.020	0.018	0.382	1.111	0.121	0.877	1.409	0.346	Imputed
rs7097453	10	44,378,740	G	A	0.050	0.051	0.961	0.996	0.078	0.855	1.160	0.055	Genotyped
rs56067768	10	44,379,021	A	G	0.023	0.020	0.272	1.134	0.115	0.906	1.419	0.376	Imputed
rs1325490	10	44,379,136	T	C	0.050	0.050	0.904	0.991	0.078	0.850	1.154	0.063	Imputed
rs117628389	10	44,379,570	A	G	0.021	0.025	0.077	0.813	0.118	0.645	1.024	0.474	Imputed
rs13313099	10	44,380,000	C	T	0.057	0.056	0.693	1.029	0.073	0.892	1.188	0.231	Imputed
rs7086120	10	44,380,589	T	C	0.050	0.050	0.929	0.993	0.078	0.852	1.157	0.054	Imputed
rs12220508	10	44,380,818	T	C	0.013	0.010	0.154	1.246	0.154	0.921	1.687	0.429	Imputed
rs73277915	10	44,381,350	G	C	0.050	0.050	0.992	0.999	0.078	0.858	1.164	0.059	Imputed
rs12248836	10	44,381,492	A	G	0.050	0.050	0.992	0.999	0.078	0.858	1.164	0.059	Imputed
rs117222048	10	44,381,835	G	T	0.026	0.018	1.09E-03	1.434	0.111	1.154	1.781	0.286	Imputed
rs144766059	10	44,381,926	T	A	0.031	0.034	0.295	0.904	0.097	0.747	1.092	0.731	Imputed
rs145949137	10	44,382,445	A	C	0.015	0.012	0.067	1.293	0.141	0.982	1.704	0.550	Imputed
rs12220920	10	44,382,680	T	C	0.013	0.010	0.154	1.246	0.154	0.921	1.687	0.429	Imputed
rs2224985	10	44,382,946	A	G	0.057	0.056	0.621	1.037	0.073	0.899	1.196	0.220	Imputed
rs10899938	10	44,383,053	C	A	0.058	0.056	0.587	1.040	0.073	0.902	1.200	0.230	Genotyped

rs11238766	10	44,384,191	T	C	0.484	0.485	0.860	0.994	0.034	0.930	1.062	0.994	Genotyped
rs79128199	10	44,384,345	T	A	0.013	0.010	0.154	1.246	0.154	0.921	1.687	0.429	Imputed
rs12218783	10	44,384,758	C	T	0.013	0.010	0.071	1.311	0.151	0.977	1.761	0.548	Genotyped
rs1460540	10	44,384,897	A	C	0.050	0.051	0.961	0.996	0.078	0.855	1.160	0.045	Imputed
rs77830065	10	44,385,391	G	A	0.057	0.056	0.790	1.020	0.073	0.884	1.176	0.476	Imputed
rs7097229	10	44,385,605	G	T	0.057	0.056	0.699	1.029	0.073	0.891	1.187	0.234	Imputed
rs1947672	10	44,386,102	G	A	0.057	0.056	0.693	1.029	0.073	0.892	1.188	0.236	Imputed
rs10899939	10	44,386,566	T	C	0.049	0.050	0.863	0.986	0.079	0.846	1.151	0.034	Imputed
rs10899940	10	44,388,083	G	C	0.050	0.050	0.937	0.994	0.078	0.853	1.158	0.054	Imputed
rs78242607	10	44,388,243	T	G	0.125	0.136	0.030	0.895	0.051	0.810	0.989	0.969	Imputed
rs1325486	10	44,388,675	A	C	0.057	0.056	0.689	1.030	0.073	0.892	1.189	0.237	Imputed
rs12218376	10	44,389,430	C	T	0.013	0.010	0.110	1.275	0.152	0.947	1.717	0.332	Imputed
rs56034709	10	44,390,053	G	A	0.463	0.465	0.913	0.996	0.034	0.932	1.065	0.398	Imputed
rs10899941	10	44,390,503	C	G	0.049	0.050	0.869	0.987	0.078	0.847	1.151	0.053	Imputed
rs10899942	10	44,390,863	A	G	0.050	0.051	0.864	0.987	0.078	0.847	1.150	0.065	Genotyped
rs1578854	10	44,391,001	T	G	0.401	0.404	0.562	0.980	0.035	0.916	1.049	0.641	Genotyped
rs7096312	10	44,391,132	G	A	0.126	0.137	0.032	0.897	0.051	0.812	0.991	0.876	Imputed
rs10899943	10	44,391,917	G	A	0.050	0.052	0.826	0.983	0.077	0.845	1.144	0.083	Imputed
rs7895918	10	44,392,068	T	C	0.458	0.460	0.590	0.982	0.034	0.919	1.049	0.875	Genotyped
rs7893153	10	44,392,411	A	G	0.457	0.460	0.571	0.981	0.034	0.918	1.048	0.972	Imputed
rs77100892	10	44,392,564	C	A	0.047	0.054	0.038	0.847	0.080	0.724	0.990	0.096	Imputed
rs61859401	10	44,392,652	T	C	0.028	0.033	0.076	0.833	0.102	0.682	1.018	0.068	Imputed
rs78212924	10	44,392,826	T	C	0.049	0.049	0.913	0.991	0.079	0.849	1.157	0.041	Imputed
rs10899944	10	44,393,232	T	C	0.462	0.462	0.976	1.001	0.034	0.937	1.070	0.457	Imputed
chr10:44393287:D	10	44,393,287	ACTCT	A	0.458	0.460	0.591	0.982	0.034	0.919	1.049	0.939	Imputed
rs11238768	10	44,393,321	G	C	0.027	0.029	0.370	0.911	0.104	0.743	1.117	0.465	Imputed
rs10899945	10	44,393,363	T	C	0.476	0.473	0.649	1.016	0.034	0.950	1.085	0.511	Imputed
rs75658212	10	44,393,675	G	A	0.015	0.014	0.511	1.097	0.141	0.832	1.447	0.503	Imputed
rs1617058	10	44,394,465	A	G	0.286	0.285	0.857	0.993	0.037	0.923	1.069	0.463	Imputed
rs79885402	10	44,394,466	C	T	0.278	0.277	0.845	0.993	0.038	0.922	1.069	0.633	Imputed
rs1147900	10	44,394,568	C	G	0.274	0.274	0.875	0.994	0.038	0.923	1.071	0.516	Imputed
rs7920691	10	44,394,825	T	C	0.038	0.040	0.601	0.954	0.089	0.802	1.136	0.023	Imputed
rs883400	10	44,395,825	G	T	0.387	0.383	0.733	1.012	0.035	0.945	1.083	0.183	Imputed
rs12358963	10	44,396,221	T	A	0.028	0.029	0.676	0.958	0.102	0.784	1.171	0.705	Imputed
rs898548	10	44,396,631	A	G	0.324	0.321	0.727	1.013	0.036	0.944	1.087	0.535	Genotyped
rs2290877	10	44,396,830	A	T	0.080	0.081	0.793	0.984	0.062	0.871	1.112	0.027	Imputed
chr10:44397286:D	10	44,397,286	T	TA	0.015	0.013	0.238	1.177	0.139	0.897	1.544	0.525	Imputed
chr10:44397293:D	10	44,397,293	A	AT	0.015	0.013	0.231	1.180	0.139	0.899	1.548	0.520	Imputed
rs7899385	10	44,398,291	T	C	0.386	0.383	0.744	1.011	0.035	0.945	1.083	0.191	Imputed
rs1468059	10	44,398,591	A	G	0.034	0.034	0.750	0.971	0.094	0.808	1.166	0.672	Imputed

rs7915088	10	44,398,861	C	T	0.398	0.394	0.675	1.015	0.035	0.948	1.086	0.115	Imputed
rs1468060	10	44,398,862	A	G	0.034	0.034	0.744	0.970	0.094	0.807	1.165	0.668	Imputed
rs10899947	10	44,399,052	C	T	0.453	0.448	0.595	1.018	0.034	0.953	1.088	0.392	Imputed
rs7900414	10	44,399,192	A	C	0.398	0.394	0.673	1.015	0.035	0.948	1.086	0.119	Imputed
rs80113207	10	44,399,258	T	G	0.012	0.014	0.659	0.935	0.152	0.695	1.260	0.770	Imputed
rs1147901	10	44,399,660	G	C	0.059	0.061	0.634	0.967	0.072	0.840	1.112	0.212	Imputed
rs1303233	10	44,400,326	G	C	0.335	0.332	0.710	1.013	0.036	0.945	1.087	0.385	Imputed
rs10793506	10	44,400,396	G	C	0.435	0.434	0.857	1.006	0.034	0.941	1.076	0.490	Imputed
rs10751347	10	44,400,595	T	C	0.398	0.394	0.708	1.013	0.035	0.947	1.084	0.133	Imputed
rs78233369	10	44,400,719	C	T	0.016	0.014	0.364	1.131	0.136	0.867	1.477	0.513	Imputed
rs10508874	10	44,400,997	C	T	0.391	0.388	0.716	1.013	0.035	0.946	1.084	0.167	Imputed
rs10899948	10	44,401,195	A	G	0.398	0.394	0.672	1.015	0.035	0.948	1.086	0.126	Imputed
rs11238771	10	44,401,456	G	C	0.038	0.039	0.722	0.969	0.089	0.814	1.153	0.027	Imputed
rs117615891	10	44,401,706	A	G	0.023	0.024	0.782	0.969	0.113	0.777	1.209	0.130	Imputed
rs1570847	10	44,402,377	T	C	0.335	0.332	0.716	1.013	0.036	0.944	1.087	0.387	Imputed
rs61859402	10	44,402,622	G	A	0.071	0.073	0.473	0.954	0.066	0.838	1.085	0.770	Imputed
rs2169198	10	44,402,926	T	C	0.335	0.332	0.716	1.013	0.036	0.944	1.087	0.387	Imputed
rs10751348	10	44,403,522	G	A	0.398	0.394	0.686	1.014	0.035	0.948	1.085	0.125	Imputed
rs1749801	10	44,403,820	G	C	0.059	0.061	0.634	0.967	0.072	0.840	1.112	0.212	Imputed
rs4948787	10	44,403,834	A	G	0.397	0.394	0.699	1.013	0.035	0.947	1.085	0.131	Imputed
rs75541195	10	44,403,927	T	C	0.016	0.014	0.248	1.170	0.136	0.896	1.528	0.541	Imputed
rs1778431	10	44,404,001	T	G	0.059	0.061	0.669	0.970	0.071	0.843	1.116	0.203	Imputed
rs55901602	10	44,404,397	A	C	0.080	0.081	0.768	0.982	0.062	0.869	1.110	0.028	Imputed
rs71505689	10	44,404,415	C	G	0.031	0.029	0.338	1.098	0.098	0.906	1.331	0.937	Imputed
rs882902	10	44,405,258	T	C	0.398	0.394	0.726	1.012	0.035	0.946	1.083	0.131	Genotyped
rs144252155	10	44,405,334	G	A	0.020	0.024	0.100	0.823	0.118	0.653	1.039	0.367	Imputed
rs80132231	10	44,405,370	G	A	0.047	0.054	0.044	0.852	0.080	0.728	0.996	0.066	Imputed
rs143746987	10	44,405,561	G	A	0.011	0.013	0.521	0.903	0.159	0.661	1.232	0.283	Imputed
rs75554270	10	44,405,852	A	G	0.080	0.081	0.809	0.985	0.062	0.872	1.113	0.031	Imputed
rs7914991	10	44,405,997	T	C	0.038	0.039	0.701	0.966	0.089	0.812	1.150	0.029	Imputed
rs2764797	10	44,406,117	G	A	0.072	0.073	0.902	0.992	0.066	0.872	1.128	0.695	Imputed
rs10899949	10	44,406,205	G	A	0.038	0.039	0.701	0.966	0.089	0.812	1.150	0.029	Imputed
rs7090750	10	44,406,302	G	T	0.398	0.394	0.708	1.013	0.035	0.947	1.084	0.133	Imputed
rs56016019	10	44,406,366	G	A	0.025	0.023	0.460	1.083	0.109	0.876	1.340	0.394	Imputed
chr10:44406743:I	10	44,406,743	G	GA	0.398	0.395	0.728	1.012	0.035	0.946	1.083	0.128	Imputed
rs1813245	10	44,407,430	A	G	0.397	0.394	0.744	1.011	0.035	0.945	1.082	0.139	Imputed
rs74618327	10	44,407,432	T	A	0.016	0.014	0.200	1.189	0.135	0.912	1.550	0.505	Imputed
rs2274241	10	44,407,897	T	G	0.397	0.394	0.721	1.012	0.035	0.946	1.083	0.135	Imputed
rs2798991	10	44,408,026	A	C	0.059	0.061	0.594	0.963	0.072	0.837	1.108	0.223	Imputed
rs2798992	10	44,408,190	A	G	0.059	0.061	0.594	0.963	0.072	0.837	1.108	0.223	Imputed

rs76647584	10	44,409,934	G	A	0.080	0.081	0.780	0.983	0.062	0.870	1.111	0.035	Imputed
rs141819858	10	44,410,425	A	G	0.016	0.014	0.206	1.186	0.135	0.910	1.547	0.510	Imputed
rs9732790	10	44,410,434	T	C	0.400	0.397	0.756	1.011	0.035	0.945	1.082	0.088	Imputed
rs9731633	10	44,410,441	T	C	0.037	0.039	0.598	0.954	0.090	0.800	1.137	0.036	Imputed
rs145409428	10	44,410,898	C	T	0.037	0.039	0.605	0.954	0.090	0.800	1.138	0.036	Imputed
rs138142462	10	44,411,090	A	G	0.022	0.028	0.031	0.780	0.115	0.623	0.977	0.010	Imputed
rs1254852	10	44,411,868	T	C	0.058	0.061	0.515	0.954	0.072	0.829	1.099	0.261	Imputed
rs150641035	10	44,412,233	C	T	0.058	0.056	0.727	1.026	0.073	0.890	1.182	0.536	Imputed
chr10:44412390:D	10	44,412,390	C	CTTA	0.017	0.014	0.235	1.171	0.133	0.902	1.520	0.354	Imputed
rs149848171	10	44,412,503	A	G	0.021	0.020	0.601	1.064	0.119	0.843	1.342	0.554	Imputed
rs1254853	10	44,412,512	T	C	0.165	0.171	0.357	0.959	0.046	0.877	1.049	0.575	Imputed
rs140055485	10	44,412,955	T	C	0.016	0.014	0.200	1.189	0.135	0.912	1.550	0.505	Imputed
rs4948789	10	44,412,980	T	A	0.399	0.395	0.644	1.016	0.035	0.950	1.087	0.132	Imputed
rs4255490	10	44,413,412	C	T	0.055	0.047	0.022	1.186	0.075	1.024	1.374	0.647	Imputed
rs148711975	10	44,414,360	G	A	0.044	0.040	0.152	1.126	0.083	0.957	1.324	0.599	Imputed
rs4948790	10	44,414,610	T	C	0.080	0.081	0.784	0.983	0.062	0.870	1.111	0.043	Imputed
rs117116899	10	44,414,988	A	G	0.016	0.014	0.159	1.208	0.135	0.928	1.572	0.471	Imputed
rs11238774	10	44,415,929	T	G	0.473	0.474	0.839	0.993	0.034	0.929	1.061	0.730	Imputed
rs12359315	10	44,416,074	T	C	0.466	0.469	0.666	0.986	0.034	0.922	1.053	0.703	Imputed
rs1254850	10	44,416,237	T	G	0.061	0.062	0.825	0.985	0.071	0.857	1.130	0.253	Imputed
rs1813897	10	44,416,505	C	T	0.410	0.406	0.681	1.014	0.034	0.948	1.085	0.259	Imputed
rs140329514	10	44,416,604	G	C	0.020	0.018	0.394	1.110	0.122	0.873	1.410	0.331	Imputed
rs144084509	10	44,417,071	A	G	0.007	0.011	0.081	0.715	0.193	0.490	1.044	0.865	Imputed
chr10:44417931:I	10	44,417,931	TC	T	0.015	0.012	0.032	1.350	0.140	1.026	1.776	0.839	Imputed
chr10:44417935:I	10	44,417,935	GC	G	0.015	0.011	0.034	1.351	0.142	1.022	1.785	0.631	Imputed
rs10899950	10	44,418,020	T	C	0.451	0.457	0.544	0.980	0.034	0.916	1.047	0.835	Imputed
rs1254851	10	44,418,024	A	C	0.218	0.225	0.262	0.955	0.041	0.881	1.035	0.552	Imputed
rs117415520	10	44,418,225	T	C	0.051	0.057	0.171	0.901	0.076	0.775	1.046	0.382	Imputed
rs2147876	10	44,418,600	T	C	0.339	0.334	0.583	1.020	0.036	0.951	1.094	0.333	Imputed
rs2147875	10	44,418,619	C	T	0.337	0.333	0.696	1.014	0.036	0.945	1.088	0.381	Imputed
rs2613103	10	44,418,721	A	C	0.061	0.063	0.730	0.976	0.070	0.850	1.120	0.360	Genotyped
rs1147902	10	44,418,891	G	A	0.338	0.334	0.719	1.013	0.036	0.944	1.087	0.351	Genotyped
rs10899951	10	44,418,976	A	T	0.456	0.462	0.447	0.975	0.034	0.912	1.042	0.363	Imputed
rs75649433	10	44,419,729	A	G	0.073	0.081	0.094	0.897	0.065	0.790	1.019	0.758	Imputed
rs17381521	10	44,419,892	T	C	0.016	0.019	0.243	0.858	0.132	0.663	1.111	0.228	Imputed
rs79377175	10	44,420,428	T	C	0.051	0.056	0.107	0.883	0.077	0.759	1.027	0.043	Imputed
rs12241154	10	44,420,538	C	T	0.038	0.039	0.725	0.969	0.089	0.814	1.153	0.026	Imputed
rs74593039	10	44,421,069	C	G	0.149	0.156	0.172	0.937	0.047	0.854	1.029	0.473	Imputed
rs1254744	10	44,421,479	G	A	0.341	0.335	0.505	1.024	0.036	0.955	1.098	0.365	Imputed
rs10793507	10	44,422,032	G	C	0.452	0.448	0.601	1.018	0.034	0.952	1.088	0.486	Imputed

rs1270513	10	44,422,512	C	T	0.060	0.062	0.773	0.980	0.071	0.853	1.126	0.268	Imputed
rs11238778	10	44,422,554	A	G	0.455	0.461	0.416	0.973	0.034	0.910	1.040	0.351	Imputed
rs75463954	10	44,422,595	A	T	0.015	0.012	0.057	1.305	0.141	0.991	1.720	0.704	Imputed
rs1254855	10	44,422,688	T	C	0.339	0.334	0.583	1.020	0.036	0.951	1.094	0.336	Imputed
rs1254856	10	44,423,030	A	G	0.059	0.061	0.618	0.965	0.072	0.839	1.110	0.216	Imputed
rs11238779	10	44,423,144	A	T	0.036	0.038	0.592	0.953	0.090	0.798	1.137	0.041	Imputed
rs1254857	10	44,423,334	T	G	0.339	0.334	0.597	1.019	0.036	0.950	1.093	0.329	Imputed
rs80218655	10	44,424,173	C	T	0.031	0.035	0.238	0.893	0.096	0.739	1.078	0.519	Imputed
rs1254858	10	44,424,688	A	G	0.339	0.334	0.612	1.018	0.036	0.949	1.092	0.354	Imputed
rs1254859	10	44,424,807	T	C	0.339	0.334	0.612	1.018	0.036	0.949	1.092	0.354	Imputed
rs79327017	10	44,425,161	A	G	0.023	0.019	0.052	1.247	0.114	0.998	1.558	0.700	Imputed
rs10508875	10	44,425,575	C	T	0.038	0.039	0.711	0.968	0.089	0.813	1.151	0.026	Imputed
rs912803	10	44,425,639	T	C	0.337	0.333	0.726	1.013	0.036	0.944	1.086	0.391	Imputed
rs57279559	10	44,425,932	T	C	0.015	0.012	0.057	1.305	0.141	0.991	1.720	0.704	Imputed
rs12244340	10	44,426,435	T	C	0.038	0.039	0.725	0.969	0.089	0.814	1.153	0.026	Imputed
rs76587907	10	44,426,829	C	T	0.015	0.012	0.057	1.305	0.141	0.991	1.720	0.704	Imputed
rs1325487	10	44,426,842	A	G	0.339	0.334	0.616	1.018	0.036	0.949	1.092	0.356	Imputed
rs10899952	10	44,427,021	T	G	0.038	0.040	0.684	0.964	0.089	0.810	1.147	0.024	Genotyped
rs1408939	10	44,427,264	G	A	0.453	0.449	0.604	1.018	0.034	0.952	1.088	0.575	Genotyped
rs2613104	10	44,427,773	T	C	0.059	0.061	0.641	0.967	0.071	0.841	1.113	0.236	Imputed
chr10:44427845:I	10	44,427,845	AT	A	0.032	0.035	0.301	0.906	0.096	0.751	1.093	0.560	Imputed
rs1749799	10	44,428,446	C	G	0.338	0.334	0.640	1.017	0.036	0.948	1.091	0.375	Imputed
rs1749798	10	44,428,806	G	A	0.353	0.346	0.384	1.031	0.035	0.962	1.105	0.333	Genotyped
rs11238781	10	44,428,985	G	A	0.038	0.039	0.731	0.970	0.089	0.815	1.154	0.027	Imputed
rs77897657	10	44,429,049	T	C	0.016	0.012	0.030	1.350	0.139	1.028	1.772	0.622	Imputed
rs984000	10	44,429,119	T	G	0.326	0.323	0.712	1.013	0.036	0.944	1.088	0.228	Imputed
rs984001	10	44,429,319	T	C	0.061	0.062	0.822	0.984	0.070	0.858	1.130	0.254	Genotyped
rs79862904	10	44,429,892	G	A	0.015	0.012	0.048	1.320	0.141	1.001	1.739	0.747	Imputed
rs898550	10	44,430,746	C	T	0.453	0.449	0.636	1.016	0.034	0.951	1.086	0.537	Genotyped
rs118010507	10	44,430,946	T	C	0.022	0.024	0.548	0.933	0.115	0.745	1.168	0.075	Imputed
rs2798993	10	44,431,576	T	C	0.327	0.322	0.548	1.022	0.036	0.952	1.097	0.232	Imputed
rs2169199	10	44,431,648	T	G	0.459	0.465	0.489	0.977	0.034	0.914	1.044	0.389	Imputed
rs2798994	10	44,431,828	T	C	0.059	0.062	0.406	0.942	0.072	0.819	1.084	0.179	Imputed
rs7070754	10	44,431,829	A	G	0.038	0.039	0.694	0.966	0.089	0.811	1.149	0.037	Imputed
rs2798995	10	44,431,939	A	G	0.059	0.062	0.398	0.941	0.072	0.818	1.083	0.181	Imputed
rs12572433	10	44,432,455	A	G	0.090	0.087	0.588	1.033	0.059	0.919	1.161	0.005	Genotyped
rs1254847	10	44,433,210	C	A	0.067	0.066	0.906	1.008	0.068	0.883	1.151	0.334	Imputed
rs6593372	10	44,433,340	G	A	0.262	0.252	0.088	1.068	0.039	0.990	1.152	0.046	Imputed
rs1254848	10	44,434,432	C	T	0.103	0.096	0.183	1.077	0.056	0.966	1.202	0.566	Imputed
rs17154663	10	44,434,473	T	C	0.093	0.094	0.995	1.000	0.058	0.892	1.121	0.002	Imputed

rs4948582	10	44,435,246	C	T	0.314	0.300	0.046	1.075	0.037	1.001	1.155	0.718	Genotyped
rs4948583	10	44,435,312	G	C	0.314	0.299	0.046	1.075	0.037	1.001	1.155	0.747	Imputed
rs34636575	10	44,435,521	G	C	0.051	0.044	0.051	1.164	0.078	0.999	1.356	0.921	Imputed
rs10899953	10	44,435,882	T	A	0.313	0.298	0.037	1.079	0.037	1.005	1.160	0.752	Imputed
rs1254849	10	44,437,185	C	T	0.067	0.066	0.864	1.012	0.068	0.886	1.155	0.320	Imputed
rs2863230	10	44,437,232	G	A	0.128	0.123	0.302	1.054	0.051	0.954	1.164	0.371	Genotyped
rs4948792	10	44,437,325	C	T	0.313	0.298	0.032	1.081	0.037	1.007	1.162	0.752	Imputed
rs10793509	10	44,437,946	A	C	0.171	0.163	0.220	1.057	0.045	0.968	1.154	0.288	Genotyped
rs2798996	10	44,438,425	T	C	0.066	0.065	0.965	1.003	0.068	0.878	1.146	0.372	Imputed
rs180692959	10	44,438,786	A	G	0.010	0.011	0.234	0.816	0.171	0.583	1.142	0.760	Imputed
rs11238782	10	44,438,863	G	A	0.405	0.392	0.070	1.064	0.034	0.995	1.139	0.140	Genotyped
rs11238783	10	44,438,866	G	A	0.389	0.381	0.263	1.040	0.035	0.971	1.113	0.235	Imputed
rs112026996	10	44,439,087	A	G	0.093	0.093	0.971	0.998	0.058	0.890	1.119	0.002	Imputed
rs17154683	10	44,439,126	A	G	0.094	0.095	0.772	0.983	0.058	0.877	1.102	0.003	Imputed
rs12413005	10	44,439,316	T	C	0.056	0.054	0.709	1.028	0.074	0.890	1.187	0.431	Imputed
rs34764480	10	44,439,740	T	C	0.051	0.044	0.036	1.177	0.078	1.011	1.371	0.954	Imputed
rs76274553	10	44,440,953	T	C	0.078	0.082	0.444	0.953	0.063	0.842	1.078	0.002	Imputed
rs117526004	10	44,441,976	T	A	0.013	0.014	0.832	0.968	0.150	0.721	1.300	0.110	Imputed
rs55991952	10	44,442,064	A	G	0.061	0.064	0.520	0.955	0.071	0.832	1.097	0.192	Imputed
rs78250945	10	44,442,320	A	T	0.056	0.054	0.667	1.032	0.074	0.893	1.192	0.339	Imputed
chr10:44442452:I	10	44,442,452	TA	T	0.023	0.028	0.126	0.844	0.111	0.678	1.049	0.664	Imputed
rs7099274	10	44,443,008	T	C	0.234	0.227	0.183	1.055	0.040	0.975	1.141	0.006	Imputed
rs80108658	10	44,443,332	T	C	0.094	0.094	0.877	1.009	0.058	0.900	1.131	0.004	Imputed
rs2798997	10	44,443,729	G	A	0.031	0.026	0.028	1.241	0.098	1.024	1.504	0.914	Genotyped
rs2798998	10	44,444,143	G	C	0.103	0.096	0.170	1.080	0.056	0.968	1.205	0.448	Imputed
rs144976296	10	44,445,354	A	G	0.022	0.025	0.333	0.894	0.115	0.714	1.120	0.084	Imputed
rs140120498	10	44,445,586	T	C	0.023	0.017	0.017	1.318	0.116	1.051	1.652	0.892	Imputed
rs1380838	10	44,446,009	T	C	0.103	0.096	0.182	1.077	0.056	0.966	1.202	0.382	Imputed
rs11528455	10	44,446,217	C	T	0.405	0.391	0.059	1.067	0.034	0.998	1.142	0.166	Imputed
rs4948793	10	44,446,480	T	C	0.235	0.227	0.175	1.056	0.040	0.976	1.142	0.005	Imputed
rs75310974	10	44,446,692	A	C	0.032	0.028	0.173	1.141	0.097	0.943	1.380	0.483	Imputed
rs6593374	10	44,446,878	A	G	0.201	0.198	0.497	1.029	0.042	0.947	1.118	0.001	Genotyped
rs78044001	10	44,447,008	C	A	0.032	0.028	0.170	1.142	0.097	0.944	1.382	0.479	Imputed
rs55738485	10	44,447,046	T	C	0.094	0.093	0.940	1.004	0.058	0.896	1.126	0.002	Imputed
rs58635230	10	44,447,328	C	T	0.094	0.094	0.878	1.009	0.058	0.900	1.131	0.003	Imputed
rs77795806	10	44,447,599	A	G	0.032	0.028	0.173	1.141	0.097	0.943	1.380	0.483	Imputed
rs1254860	10	44,448,020	C	T	0.103	0.096	0.172	1.079	0.056	0.967	1.204	0.374	Imputed
rs898551	10	44,448,299	G	A	0.406	0.392	0.068	1.065	0.034	0.995	1.139	0.181	Imputed
rs4948794	10	44,448,664	T	G	0.094	0.094	0.880	1.009	0.058	0.900	1.130	0.003	Genotyped
rs1325488	10	44,448,756	T	C	0.195	0.217	7.10E-04	0.866	0.043	0.797	0.941	0.107	Genotyped

rs4948795	10	44,448,975	C	T	0.094	0.094	0.873	1.009	0.058	0.901	1.131	0.004	Imputed
rs7067919	10	44,449,117	A	G	0.085	0.085	0.904	1.007	0.061	0.894	1.135	0.118	Imputed
rs79844083	10	44,449,281	G	C	0.018	0.021	0.238	0.861	0.127	0.672	1.104	0.848	Imputed
rs80130675	10	44,449,668	T	C	0.015	0.013	0.114	1.246	0.140	0.948	1.638	0.683	Imputed
rs1254861	10	44,449,913	T	C	0.046	0.041	0.135	1.129	0.081	0.963	1.324	0.902	Imputed
rs1325489	10	44,450,060	T	C	0.027	0.022	0.026	1.266	0.106	1.028	1.558	0.588	Imputed
rs2764793	10	44,450,561	G	C	0.066	0.065	0.964	1.003	0.068	0.878	1.146	0.324	Imputed
rs139516867	10	44,450,563	G	A	0.013	0.014	0.832	0.968	0.150	0.721	1.300	0.110	Imputed
rs2764794	10	44,450,572	T	C	0.066	0.065	0.964	1.003	0.068	0.878	1.146	0.324	Imputed
rs192041803	10	44,450,970	C	G	0.012	0.011	0.449	1.123	0.154	0.831	1.517	0.500	Imputed
rs2764795	10	44,451,307	T	C	0.066	0.065	0.965	1.003	0.068	0.878	1.146	0.324	Imputed
rs2764796	10	44,451,356	A	C	0.066	0.065	0.965	1.003	0.068	0.878	1.146	0.324	Imputed
rs146522905	10	44,451,423	T	G	0.016	0.017	0.852	0.976	0.133	0.752	1.266	0.511	Imputed
rs7100261	10	44,451,599	C	T	0.198	0.195	0.445	1.033	0.043	0.951	1.123	0.001	Imputed
rs61856523	10	44,451,648	C	G	0.013	0.013	0.973	1.005	0.151	0.748	1.350	0.538	Imputed
rs1408941	10	44,451,840	T	G	0.028	0.026	0.478	1.076	0.103	0.879	1.317	0.522	Imputed
chr10:44452151:D	10	44,452,151	G	GGCA	0.032	0.028	0.159	1.146	0.097	0.948	1.387	0.502	Imputed
rs78121076	10	44,452,589	C	A	0.096	0.095	0.850	1.011	0.058	0.903	1.132	0.004	Imputed
rs1254671	10	44,453,049	T	C	0.046	0.041	0.145	1.126	0.081	0.960	1.320	0.883	Imputed
rs143024235	10	44,453,380	A	G	0.011	0.013	0.351	0.863	0.158	0.633	1.176	0.723	Imputed
rs7088951	10	44,453,597	C	A	0.406	0.392	0.063	1.066	0.034	0.997	1.141	0.209	Imputed
rs17154727	10	44,453,721	T	C	0.015	0.013	0.106	1.253	0.140	0.953	1.647	0.696	Imputed
rs10899954	10	44,453,833	C	A	0.401	0.386	0.053	1.069	0.035	0.999	1.144	0.165	Imputed
rs1006870	10	44,454,153	T	C	0.015	0.013	0.127	1.237	0.139	0.941	1.626	0.734	Genotyped
rs76901374	10	44,454,177	C	T	0.027	0.019	4.33E-04	1.452	0.106	1.178	1.788	0.811	Imputed
rs10508876	10	44,454,186	T	C	0.076	0.078	0.807	0.985	0.064	0.869	1.116	0.351	Genotyped
rs17467041	10	44,454,532	T	C	0.014	0.015	0.336	0.870	0.145	0.655	1.156	0.676	Imputed
rs117073736	10	44,454,557	T	C	0.015	0.013	0.102	1.256	0.140	0.955	1.651	0.703	Imputed
chr10:44454669:D	10	44,454,669	G	GGTGAGGACA	0.066	0.065	0.964	1.003	0.068	0.878	1.146	0.324	Imputed
rs78180983	10	44,454,825	A	G	0.015	0.013	0.102	1.256	0.140	0.955	1.651	0.703	Imputed
rs78621342	10	44,455,661	A	C	0.028	0.026	0.477	1.076	0.103	0.880	1.316	0.599	Imputed
rs11238788	10	44,455,772	A	G	0.087	0.088	0.984	0.999	0.060	0.888	1.124	0.127	Imputed
rs76108423	10	44,455,964	A	G	0.035	0.030	0.109	1.161	0.093	0.967	1.393	0.577	Imputed
rs7924201	10	44,456,029	G	T	0.405	0.390	0.047	1.071	0.035	1.001	1.146	0.171	Genotyped
rs74910987	10	44,456,267	A	T	0.036	0.032	0.180	1.130	0.091	0.945	1.352	0.589	Imputed
rs1977341	10	44,456,327	A	G	0.169	0.161	0.265	1.052	0.045	0.962	1.149	0.195	Imputed
rs1570846	10	44,456,480	A	G	0.199	0.196	0.503	1.029	0.042	0.947	1.118	0.001	Imputed
rs117147824	10	44,456,622	T	C	0.021	0.022	0.647	0.947	0.119	0.750	1.196	0.705	Imputed
rs1532232	10	44,456,716	A	G	0.097	0.096	0.814	1.014	0.057	0.906	1.134	0.003	Genotyped
rs75614193	10	44,456,895	G	A	0.035	0.030	0.109	1.161	0.093	0.967	1.393	0.577	Imputed

rs75555492	10	44,457,051	G	T	0.035	0.030	0.092	1.170	0.093	0.974	1.404	0.587	Imputed
rs147037320	10	44,457,394	T	C	0.008	0.011	0.203	0.790	0.185	0.550	1.136	0.815	Imputed
rs1262195	10	44,458,221	A	G	0.066	0.065	0.964	1.003	0.068	0.878	1.146	0.341	Imputed
rs898549	10	44,458,714	C	T	0.403	0.391	0.096	1.059	0.035	0.990	1.133	0.215	Genotyped
rs74138964	10	44,458,962	T	C	0.031	0.029	0.261	1.115	0.097	0.922	1.350	0.520	Imputed
rs912801	10	44,459,354	T	C	0.027	0.026	0.570	1.061	0.104	0.865	1.301	0.682	Imputed
rs7915624	10	44,459,459	T	C	0.198	0.195	0.542	1.026	0.043	0.944	1.116	0.001	Imputed
rs66887775	10	44,459,605	C	T	0.402	0.391	0.105	1.058	0.035	0.988	1.132	0.177	Imputed
rs7919084	10	44,459,724	T	C	0.232	0.226	0.254	1.047	0.040	0.968	1.133	0.004	Imputed
rs79845004	10	44,459,766	T	C	0.015	0.013	0.106	1.253	0.140	0.953	1.646	0.768	Imputed
rs151227898	10	44,460,161	A	G	0.020	0.018	0.536	1.080	0.123	0.848	1.374	0.254	Imputed
rs1613279	10	44,460,411	A	G	0.067	0.066	0.902	1.008	0.068	0.883	1.152	0.479	Imputed
rs1613984	10	44,460,433	T	G	0.067	0.066	0.923	1.007	0.068	0.881	1.149	0.525	Genotyped
rs78662260	10	44,460,923	G	C	0.015	0.013	0.106	1.253	0.140	0.953	1.646	0.768	Imputed
rs12413613	10	44,461,478	C	T	0.012	0.011	0.441	1.127	0.155	0.832	1.528	0.189	Imputed
rs67743392	10	44,462,194	C	T	0.077	0.078	0.947	0.996	0.064	0.879	1.128	0.200	Imputed
rs71505691	10	44,462,291	T	A	0.028	0.028	0.934	1.009	0.103	0.824	1.234	0.597	Imputed
rs75949050	10	44,462,325	G	C	0.015	0.013	0.106	1.253	0.140	0.953	1.646	0.768	Imputed
rs7072297	10	44,462,360	G	C	0.155	0.156	0.994	1.000	0.047	0.912	1.096	0.001	Imputed
rs61856524	10	44,462,480	T	A	0.013	0.013	0.960	1.008	0.151	0.750	1.354	0.544	Imputed
rs4948796	10	44,462,577	T	C	0.043	0.039	0.146	1.130	0.084	0.959	1.332	0.653	Imputed
rs1749800	10	44,462,941	A	G	0.067	0.066	0.901	1.008	0.068	0.883	1.152	0.479	Imputed
rs10899955	10	44,462,959	T	C	0.400	0.387	0.094	1.060	0.035	0.990	1.134	0.141	Imputed
rs58037492	10	44,462,974	G	A	0.095	0.095	0.886	1.008	0.058	0.900	1.129	0.006	Imputed
rs11238790	10	44,463,442	A	G	0.087	0.087	0.846	1.012	0.060	0.900	1.138	0.104	Imputed
rs7901081	10	44,463,444	T	G	0.198	0.196	0.522	1.028	0.043	0.946	1.117	0.001	Genotyped
rs7894721	10	44,464,507	C	T	0.234	0.228	0.214	1.051	0.040	0.972	1.137	0.005	Imputed
rs79717271	10	44,464,595	A	G	0.015	0.013	0.106	1.253	0.140	0.953	1.646	0.768	Imputed
rs1147904	10	44,464,611	C	T	0.101	0.096	0.321	1.057	0.056	0.947	1.180	0.418	Imputed
rs114782853	10	44,464,614	G	A	0.036	0.032	0.124	1.151	0.091	0.962	1.377	0.369	Imputed
rs74138971	10	44,464,804	C	A	0.036	0.032	0.132	1.147	0.091	0.959	1.372	0.378	Imputed
rs71505692	10	44,464,869	T	A	0.028	0.028	0.934	1.009	0.103	0.824	1.234	0.597	Imputed
rs71505693	10	44,464,873	A	G	0.028	0.028	0.934	1.009	0.103	0.824	1.234	0.597	Imputed
rs11238791	10	44,465,110	A	G	0.076	0.077	0.905	0.992	0.064	0.876	1.125	0.291	Imputed
rs58297052	10	44,465,356	A	G	0.028	0.026	0.538	1.066	0.104	0.870	1.306	0.700	Imputed
rs1147905	10	44,465,381	G	C	0.067	0.066	0.876	1.011	0.068	0.885	1.154	0.448	Imputed
rs57549604	10	44,465,655	C	T	0.015	0.013	0.106	1.253	0.140	0.953	1.646	0.768	Imputed
rs60798435	10	44,465,757	A	G	0.198	0.196	0.519	1.028	0.043	0.946	1.117	0.001	Imputed
rs74138972	10	44,466,005	T	C	0.033	0.030	0.199	1.130	0.095	0.938	1.362	0.385	Imputed
rs1147906	10	44,466,151	G	T	0.027	0.022	0.040	1.242	0.105	1.010	1.527	0.669	Imputed

rs10899956	10	44,466,480	C	T	0.403	0.391	0.091	1.060	0.035	0.991	1.134	0.146	Imputed
rs61856544	10	44,466,942	T	C	0.013	0.013	0.853	1.028	0.149	0.767	1.378	0.598	Imputed
rs1147907	10	44,467,046	A	G	0.102	0.096	0.312	1.058	0.056	0.948	1.181	0.386	Imputed
rs7904114	10	44,467,185	G	A	0.235	0.228	0.187	1.054	0.040	0.975	1.140	0.005	Imputed
rs77625284	10	44,467,355	T	C	0.015	0.013	0.106	1.253	0.140	0.953	1.646	0.768	Imputed
rs75439663	10	44,467,417	A	G	0.036	0.045	6.95E-03	0.785	0.090	0.658	0.936	0.416	Imputed
rs74692261	10	44,467,449	T	C	0.034	0.031	0.159	1.140	0.093	0.950	1.369	0.489	Imputed
rs12570158	10	44,467,520	T	C	0.029	0.027	0.369	1.095	0.101	0.898	1.336	0.855	Imputed
rs6593375	10	44,467,596	C	G	0.155	0.156	0.988	1.001	0.047	0.913	1.097	0.001	Imputed
chr10:44467958:D	10	44,467,958	TG	T	0.404	0.391	0.082	1.062	0.035	0.992	1.136	0.140	Imputed
rs6593376	10	44,469,148	T	C	0.198	0.196	0.540	1.026	0.043	0.944	1.116	0.001	Genotyped
rs112316747	10	44,469,326	G	C	0.035	0.036	0.852	0.983	0.092	0.822	1.177	0.049	Imputed
rs1147908	10	44,469,459	A	G	0.102	0.096	0.310	1.059	0.056	0.948	1.182	0.385	Imputed
rs4948799	10	44,469,683	G	C	0.111	0.108	0.518	1.036	0.054	0.932	1.151	0.004	Imputed
rs74138974	10	44,469,786	T	C	0.032	0.029	0.225	1.125	0.097	0.930	1.360	0.494	Imputed
rs77092103	10	44,470,416	T	G	0.095	0.095	0.926	1.005	0.058	0.898	1.126	0.007	Imputed
rs1147909	10	44,470,909	T	C	0.102	0.096	0.315	1.058	0.056	0.948	1.181	0.405	Genotyped
rs73283848	10	44,470,955	A	C	0.055	0.054	0.888	1.010	0.074	0.874	1.168	0.361	Imputed
rs1460542	10	44,471,109	A	C	0.168	0.162	0.382	1.040	0.045	0.952	1.137	0.269	Genotyped
rs4948800	10	44,471,284	T	C	0.400	0.388	0.111	1.057	0.035	0.987	1.131	0.144	Imputed
rs12359058	10	44,471,343	T	G	0.399	0.386	0.091	1.060	0.035	0.991	1.135	0.151	Imputed
rs77055497	10	44,471,437	G	A	0.015	0.013	0.106	1.253	0.140	0.953	1.646	0.768	Imputed
rs2085797	10	44,471,736	T	C	0.400	0.387	0.096	1.059	0.035	0.990	1.133	0.139	Imputed
rs75016070	10	44,472,122	A	C	0.031	0.036	0.093	0.849	0.097	0.702	1.027	0.100	Imputed
rs10899957	10	44,472,469	C	T	0.402	0.389	0.080	1.062	0.035	0.993	1.137	0.145	Imputed
rs10899958	10	44,473,036	C	T	0.089	0.088	0.750	1.019	0.060	0.907	1.146	0.093	Genotyped
rs78163145	10	44,473,270	T	C	0.035	0.036	0.960	0.995	0.091	0.832	1.191	0.047	Imputed
rs61856545	10	44,473,280	G	A	0.013	0.013	0.947	1.010	0.151	0.752	1.357	0.550	Imputed
rs1254672	10	44,473,731	G	A	0.067	0.066	0.824	1.015	0.068	0.889	1.159	0.427	Imputed
rs1254673	10	44,473,793	A	T	0.101	0.096	0.322	1.057	0.056	0.947	1.180	0.470	Imputed
rs1254674	10	44,474,571	T	G	0.101	0.096	0.331	1.056	0.056	0.946	1.179	0.458	Imputed
rs74555986	10	44,474,945	A	T	0.053	0.054	0.758	0.977	0.075	0.843	1.133	0.576	Imputed
rs185545954	10	44,475,145	A	G	0.010	0.010	0.927	0.985	0.169	0.707	1.372	0.245	Imputed
rs2279553	10	44,476,107	A	C	0.015	0.013	0.118	1.242	0.139	0.946	1.632	0.899	Genotyped
rs2279554	10	44,476,131	T	C	0.015	0.013	0.099	1.258	0.139	0.958	1.654	0.989	Imputed
rs2279555	10	44,476,596	G	A	0.172	0.169	0.724	1.016	0.045	0.930	1.109	0.178	Imputed
chr10:44476722:I	10	44,476,722	TC	T	0.135	0.126	0.089	1.088	0.050	0.987	1.200	0.370	Imputed
chr10:44477098:D	10	44,477,098	GC	G	0.437	0.411	9.20E-04	1.120	0.034	1.047	1.197	0.983	Imputed
rs10899959	10	44,477,463	A	G	0.114	0.105	0.064	1.104	0.054	0.994	1.227	0.516	Imputed
rs58563011	10	44,477,693	G	A	0.094	0.092	0.602	1.031	0.058	0.920	1.156	0.039	Imputed

chr10:44478185:D	10	44,478,185	T	TA	0.015	0.013	0.161	1.219	0.141	0.924	1.607	0.881	Imputed
rs11238793	10	44,478,187	C	T	0.116	0.107	0.054	1.108	0.053	0.998	1.229	0.589	Imputed
rs75385417	10	44,478,378	G	A	0.015	0.013	0.161	1.219	0.141	0.924	1.607	0.881	Imputed
rs80009740	10	44,478,467	C	T	0.015	0.013	0.161	1.219	0.141	0.924	1.607	0.881	Imputed
rs75926385	10	44,478,759	T	G	0.030	0.027	0.197	1.136	0.099	0.936	1.378	0.211	Imputed
rs139711366	10	44,478,774	G	A	0.016	0.016	0.917	0.986	0.136	0.755	1.287	0.368	Imputed
rs11238794	10	44,478,975	C	A	0.333	0.314	0.014	1.093	0.036	1.018	1.172	0.336	Imputed
rs10793510	10	44,479,033	A	T	0.179	0.176	0.655	1.020	0.044	0.935	1.112	0.369	Imputed
rs4948803	10	44,479,236	T	C	0.219	0.208	0.127	1.065	0.041	0.982	1.154	0.133	Imputed
rs75141475	10	44,479,449	C	T	0.086	0.085	0.665	1.027	0.060	0.912	1.156	0.073	Imputed
rs10899960	10	44,479,890	C	A	0.214	0.204	0.210	1.053	0.041	0.971	1.142	0.142	Imputed
rs71505696	10	44,480,166	T	C	0.035	0.030	0.061	1.188	0.092	0.992	1.423	0.088	Imputed
rs116940170	10	44,480,287	A	G	0.018	0.020	0.374	0.894	0.126	0.698	1.145	0.553	Imputed
rs56338358	10	44,480,670	A	T	0.138	0.127	0.035	1.110	0.049	1.007	1.222	0.822	Imputed
rs1870635	10	44,480,694	C	T	0.351	0.331	0.010	1.095	0.036	1.022	1.174	0.261	Imputed
rs71505697	10	44,480,727	T	C	0.035	0.030	0.063	1.187	0.093	0.990	1.424	0.088	Imputed
rs1147913	10	44,480,798	T	C	0.027	0.022	0.036	1.249	0.106	1.014	1.539	0.830	Imputed
rs1870634	10	44,480,811	T	G	0.351	0.331	0.011	1.095	0.036	1.021	1.174	0.255	Imputed
rs7088607	10	44,481,022	A	C	0.138	0.127	0.031	1.112	0.049	1.010	1.225	0.840	Imputed
rs6593377	10	44,481,304	T	C	0.214	0.204	0.166	1.059	0.041	0.977	1.148	0.162	Imputed
rs72781190	10	44,481,328	A	G	0.027	0.023	0.150	1.165	0.106	0.946	1.435	0.618	Imputed
rs148291414	10	44,481,622	T	C	0.022	0.018	0.045	1.262	0.117	1.004	1.587	0.607	Imputed
rs988738	10	44,481,639	C	G	0.213	0.203	0.176	1.058	0.041	0.975	1.147	0.151	Imputed
rs118163675	10	44,481,644	T	C	0.017	0.020	0.293	0.874	0.129	0.679	1.124	0.853	Imputed
rs988739	10	44,481,673	G	A	0.351	0.331	0.011	1.095	0.036	1.021	1.174	0.262	Imputed
rs79580903	10	44,481,813	G	T	0.023	0.024	0.566	0.937	0.113	0.751	1.170	0.857	Imputed
rs7092322	10	44,482,051	A	G	0.138	0.127	0.035	1.109	0.049	1.007	1.222	0.820	Imputed
rs72781191	10	44,482,264	G	A	0.026	0.022	0.203	1.147	0.108	0.928	1.418	0.615	Imputed
rs61856546	10	44,482,538	C	T	0.013	0.012	0.665	1.067	0.150	0.795	1.432	0.520	Imputed
rs34919292	10	44,482,558	T	G	0.035	0.030	0.062	1.187	0.092	0.991	1.421	0.089	Imputed
rs17382710	10	44,482,857	T	A	0.060	0.074	7.13E-04	0.787	0.071	0.685	0.904	0.025	Imputed
rs10508877	10	44,482,885	A	G	0.138	0.127	0.029	1.114	0.049	1.011	1.227	0.781	Genotyped
rs1147914	10	44,482,988	T	G	0.031	0.026	0.035	1.230	0.098	1.014	1.492	0.712	Imputed
rs17154773	10	44,483,336	C	T	0.086	0.085	0.633	1.029	0.060	0.914	1.159	0.077	Imputed
rs4948804	10	44,483,355	G	C	0.128	0.124	0.553	1.031	0.051	0.933	1.138	0.615	Imputed
rs2862856	10	44,483,397	G	A	0.109	0.103	0.316	1.056	0.055	0.949	1.175	0.841	Genotyped
rs4145117	10	44,483,521	G	A	0.487	0.459	3.57E-04	1.128	0.034	1.056	1.206	0.500	Imputed
rs4145118	10	44,483,728	G	T	0.214	0.203	0.165	1.059	0.041	0.977	1.148	0.162	Imputed
rs17467664	10	44,483,778	T	C	0.026	0.029	0.271	0.891	0.106	0.724	1.096	0.187	Imputed
rs78523020	10	44,483,919	A	G	0.086	0.085	0.650	1.028	0.060	0.913	1.157	0.071	Imputed

rs55872338	10	44,484,296	A	G	0.075	0.078	0.569	0.964	0.064	0.850	1.093	0.053	Imputed
chr10:44484823:D	10	44,484,823	T	TTTTTTA	0.088	0.091	0.463	0.957	0.059	0.852	1.076	0.359	Imputed
rs7918198	10	44,484,939	T	C	0.227	0.217	0.156	1.059	0.040	0.978	1.146	0.212	Imputed
rs11238795	10	44,485,063	G	A	0.138	0.127	0.034	1.110	0.049	1.008	1.223	0.824	Imputed
rs4322346	10	44,485,267	G	A	0.228	0.220	0.299	1.043	0.040	0.963	1.129	0.196	Imputed
rs150210000	10	44,485,396	T	C	0.040	0.037	0.615	1.045	0.087	0.881	1.240	0.643	Imputed
rs9633746	10	44,485,575	G	C	0.223	0.215	0.274	1.046	0.041	0.965	1.132	0.243	Imputed
rs4307670	10	44,486,040	T	A	0.187	0.184	0.769	1.013	0.043	0.930	1.103	0.628	Imputed
rs4948805	10	44,486,148	T	C	0.214	0.204	0.210	1.053	0.041	0.971	1.142	0.142	Imputed
chr10:44486424:D	10	44,486,424	CTT	C	0.289	0.284	0.498	1.026	0.038	0.953	1.104	0.215	Imputed
rs147333030	10	44,486,939	G	A	0.015	0.013	0.088	1.268	0.139	0.965	1.666	0.852	Imputed
rs145726402	10	44,487,027	T	C	0.035	0.030	0.075	1.178	0.092	0.983	1.412	0.097	Imputed
rs150732849	10	44,487,037	C	T	0.078	0.080	0.662	0.973	0.063	0.860	1.101	0.065	Imputed
rs190708151	10	44,487,231	A	G	0.024	0.025	0.664	0.953	0.110	0.768	1.183	0.459	Imputed
rs9633745	10	44,487,298	C	T	0.487	0.459	5.95E-04	1.123	0.034	1.051	1.200	0.572	Imputed
rs9633747	10	44,487,413	C	A	0.220	0.209	0.152	1.060	0.041	0.979	1.149	0.119	Imputed
rs144804680	10	44,487,504	T	C	0.036	0.030	0.036	1.212	0.092	1.012	1.451	0.102	Imputed
rs146760397	10	44,487,535	A	G	0.086	0.085	0.715	1.022	0.061	0.908	1.151	0.081	Imputed
rs11238796	10	44,487,567	T	C	0.111	0.104	0.123	1.087	0.054	0.978	1.209	0.606	Imputed
rs141975006	10	44,487,743	A	G	0.105	0.112	0.174	0.928	0.055	0.833	1.034	0.933	Imputed
rs4582922	10	44,487,877	A	G	0.226	0.214	0.112	1.067	0.041	0.985	1.155	0.406	Imputed
rs11238797	10	44,487,934	A	G	0.138	0.127	0.034	1.110	0.049	1.008	1.223	0.782	Imputed
rs138271684	10	44,487,950	T	C	0.027	0.023	0.147	1.166	0.106	0.947	1.436	0.612	Imputed
rs11238798	10	44,487,973	A	G	0.141	0.129	0.021	1.120	0.049	1.017	1.233	0.804	Imputed
rs4320909	10	44,487,991	T	G	0.257	0.251	0.399	1.033	0.039	0.958	1.115	0.274	Imputed
rs139352978	10	44,488,057	C	A	0.100	0.093	0.134	1.089	0.057	0.974	1.217	0.581	Imputed
rs11818958	10	44,488,192	C	A	0.498	0.470	3.72E-04	1.128	0.034	1.056	1.205	0.674	Imputed
rs10899961	10	44,488,202	T	C	0.230	0.221	0.220	1.051	0.040	0.971	1.137	0.423	Imputed
rs11238799	10	44,488,220	A	G	0.139	0.128	0.032	1.111	0.049	1.009	1.223	0.925	Imputed
rs56397518	10	44,488,226	G	A	0.086	0.085	0.738	1.020	0.061	0.906	1.149	0.083	Imputed
rs11238800	10	44,488,327	G	A	0.500	0.472	4.75E-04	1.126	0.034	1.053	1.203	0.713	Imputed
rs11238801	10	44,488,484	C	G	0.352	0.332	0.011	1.095	0.035	1.021	1.174	0.324	Imputed
rs1032408	10	44,488,843	G	T	0.213	0.204	0.217	1.052	0.041	0.970	1.141	0.174	Imputed
rs34925084	10	44,488,907	C	T	0.035	0.030	0.061	1.188	0.092	0.992	1.423	0.088	Imputed
rs75797980	10	44,489,057	T	C	0.032	0.029	0.206	1.128	0.096	0.935	1.362	0.225	Imputed
rs10793511	10	44,489,255	G	A	0.487	0.459	5.11E-04	1.125	0.034	1.053	1.202	0.545	Imputed
rs10736824	10	44,489,375	C	G	0.214	0.205	0.208	1.053	0.041	0.971	1.142	0.181	Imputed
rs4948806	10	44,489,563	A	G	0.177	0.172	0.575	1.025	0.044	0.940	1.118	0.514	Imputed
rs4948589	10	44,489,626	A	G	0.213	0.203	0.186	1.056	0.041	0.974	1.145	0.179	Imputed
rs4948590	10	44,489,655	A	G	0.487	0.459	4.12E-04	1.127	0.034	1.055	1.204	0.548	Imputed

rs79674034	10	44,489,923	C	T	0.034	0.030	0.182	1.134	0.094	0.943	1.363	0.182	Imputed
chr10:44489998:D	10	44,489,998	ATCC	A	0.351	0.331	0.010	1.095	0.035	1.022	1.174	0.277	Imputed
chr10:44490002:D	10	44,490,002	T	TC	0.087	0.087	0.883	1.009	0.060	0.897	1.135	0.346	Imputed
rs61856547	10	44,490,082	T	A	0.013	0.012	0.690	1.062	0.150	0.791	1.424	0.507	Imputed
rs17154815	10	44,490,330	G	T	0.015	0.013	0.088	1.268	0.139	0.965	1.666	0.852	Imputed
rs7090343	10	44,490,338	A	G	0.487	0.459	5.54E-04	1.124	0.034	1.052	1.201	0.553	Imputed
rs12411546	10	44,490,402	A	C	0.023	0.024	0.550	0.935	0.113	0.749	1.166	0.867	Imputed
rs4948591	10	44,490,414	G	T	0.487	0.459	5.88E-04	1.123	0.034	1.051	1.200	0.549	Genotyped
chr10:44490427:I	10	44,490,427	T	TAA	0.379	0.357	8.38E-03	1.097	0.035	1.024	1.174	0.358	Imputed
rs4948807	10	44,490,428	T	A	0.357	0.338	0.015	1.090	0.035	1.017	1.168	0.280	Imputed
rs4948592	10	44,490,429	C	A	0.357	0.338	0.013	1.091	0.035	1.018	1.170	0.277	Imputed
chr10:44490429:I	10	44,490,429	C	CAA	0.374	0.354	0.014	1.090	0.035	1.018	1.168	0.438	Imputed
rs17154827	10	44,490,532	G	T	0.086	0.085	0.742	1.020	0.061	0.906	1.149	0.083	Imputed
rs4948593	10	44,490,574	C	T	0.213	0.203	0.186	1.056	0.041	0.974	1.145	0.179	Imputed
rs4948594	10	44,490,669	C	G	0.213	0.203	0.186	1.056	0.041	0.974	1.145	0.179	Imputed
rs72781195	10	44,490,834	A	G	0.111	0.103	0.121	1.088	0.054	0.978	1.210	0.587	Imputed
rs4948808	10	44,490,854	A	G	0.218	0.208	0.177	1.057	0.041	0.975	1.145	0.239	Imputed
rs4948809	10	44,490,865	A	G	0.213	0.203	0.186	1.056	0.041	0.974	1.145	0.179	Imputed
rs56952006	10	44,490,960	C	T	0.120	0.115	0.302	1.055	0.052	0.953	1.169	0.468	Imputed
rs10793512	10	44,491,154	T	A	0.213	0.203	0.185	1.056	0.041	0.974	1.146	0.178	Imputed
rs11238802	10	44,491,156	T	C	0.060	0.064	0.349	0.936	0.071	0.814	1.075	0.383	Imputed
rs80079896	10	44,491,356	T	G	0.042	0.041	0.699	1.033	0.084	0.876	1.218	0.225	Imputed
rs7906332	10	44,491,991	T	C	0.138	0.127	0.035	1.110	0.049	1.007	1.222	0.800	Imputed
rs17154842	10	44,492,045	A	G	0.015	0.013	0.088	1.268	0.139	0.965	1.666	0.852	Imputed
rs12761349	10	44,492,898	T	A	0.035	0.030	0.056	1.193	0.093	0.995	1.430	0.134	Imputed
rs955858	10	44,494,209	A	G	0.138	0.127	0.034	1.110	0.049	1.008	1.223	0.783	Imputed
rs10793513	10	44,494,546	G	A	0.352	0.331	9.14E-03	1.097	0.035	1.023	1.176	0.363	Genotyped
rs7091447	10	44,494,658	G	A	0.488	0.459	3.38E-04	1.129	0.034	1.056	1.206	0.587	Imputed
rs75156299	10	44,494,779	C	T	0.015	0.013	0.088	1.268	0.139	0.965	1.666	0.852	Imputed
rs4997087	10	44,495,365	T	C	0.214	0.203	0.156	1.060	0.041	0.978	1.150	0.262	Imputed
rs7908336	10	44,495,410	T	C	0.209	0.200	0.210	1.054	0.042	0.971	1.143	0.177	Imputed
rs111550935	10	44,495,515	G	C	0.027	0.023	0.169	1.157	0.106	0.940	1.425	0.599	Imputed
chr10:44495692:D	10	44,495,692	T	TG	0.026	0.028	0.723	0.963	0.105	0.783	1.184	0.936	Imputed
rs6593381	10	44,495,877	G	A	0.138	0.127	0.034	1.110	0.049	1.008	1.223	0.855	Imputed
rs6593382	10	44,495,969	A	T	0.138	0.127	0.034	1.110	0.049	1.008	1.223	0.855	Imputed
rs6593383	10	44,496,064	C	A	0.138	0.127	0.034	1.110	0.049	1.008	1.223	0.855	Imputed
rs11238804	10	44,496,077	T	A	0.070	0.069	0.966	1.003	0.067	0.880	1.142	0.789	Imputed
rs117208452	10	44,496,213	A	G	0.015	0.013	0.131	1.233	0.139	0.939	1.619	0.780	Imputed
rs10793514	10	44,496,971	T	C	0.352	0.331	8.17E-03	1.098	0.035	1.025	1.177	0.372	Imputed
rs10793515	10	44,496,985	C	T	0.488	0.459	3.38E-04	1.129	0.034	1.056	1.206	0.587	Imputed

rs75003649	10	44,496,992	C	T	0.034	0.030	0.161	1.140	0.094	0.949	1.370	0.173	Imputed
rs10899962	10	44,497,073	A	T	0.214	0.203	0.153	1.061	0.041	0.978	1.150	0.260	Imputed
rs12570705	10	44,497,432	A	G	0.138	0.127	0.034	1.110	0.049	1.008	1.223	0.854	Imputed
rs2902340	10	44,497,495	T	G	0.269	0.255	0.066	1.073	0.038	0.995	1.156	0.441	Imputed
rs7084766	10	44,498,334	A	G	0.138	0.127	0.033	1.111	0.049	1.008	1.223	0.857	Imputed
rs6593384	10	44,498,373	G	A	0.222	0.211	0.147	1.061	0.041	0.979	1.149	0.223	Imputed
rs6593385	10	44,498,375	G	A	0.216	0.206	0.193	1.055	0.041	0.973	1.144	0.143	Imputed
rs148532901	10	44,498,420	A	G	0.016	0.014	0.096	1.251	0.135	0.960	1.629	0.615	Imputed
rs183216249	10	44,498,451	A	G	0.018	0.014	0.036	1.306	0.128	1.016	1.679	0.814	Imputed
rs71491005	10	44,498,617	A	G	0.037	0.032	0.044	1.198	0.090	1.004	1.428	0.170	Imputed
rs4948811	10	44,499,939	G	T	0.241	0.229	0.092	1.069	0.040	0.989	1.155	0.266	Imputed
rs1873756	10	44,500,350	G	A	0.488	0.460	3.61E-04	1.128	0.034	1.056	1.206	0.581	Imputed
rs77512972	10	44,500,778	T	C	0.033	0.038	0.157	0.875	0.094	0.728	1.052	0.031	Imputed
rs7478408	10	44,500,807	C	T	0.488	0.460	3.17E-04	1.130	0.034	1.057	1.207	0.568	Imputed
rs11238806	10	44,500,927	C	G	0.009	0.012	0.095	0.747	0.175	0.530	1.052	0.541	Imputed
rs192769802	10	44,501,062	T	C	0.009	0.011	0.139	0.769	0.178	0.543	1.089	0.503	Imputed
rs56091632	10	44,501,079	T	C	0.138	0.127	0.035	1.110	0.049	1.007	1.222	0.872	Imputed
rs10899963	10	44,501,123	T	G	0.478	0.447	7.44E-05	1.144	0.034	1.070	1.222	0.520	Imputed
rs7922924	10	44,501,266	C	T	0.225	0.213	0.108	1.067	0.041	0.986	1.156	0.316	Imputed
rs78265338	10	44,501,412	G	A	0.015	0.013	0.088	1.267	0.139	0.965	1.665	0.850	Imputed
rs36078900	10	44,501,447	T	C	0.036	0.030	0.038	1.208	0.092	1.009	1.446	0.106	Imputed
rs11238807	10	44,501,460	T	C	0.138	0.127	0.029	1.113	0.049	1.011	1.226	0.870	Genotyped
rs7923335	10	44,501,501	C	A	0.218	0.209	0.222	1.051	0.041	0.970	1.139	0.219	Imputed
rs147000035	10	44,501,570	A	G	0.012	0.016	0.042	0.733	0.153	0.542	0.990	0.828	Imputed
rs10899964	10	44,501,813	G	A	0.138	0.127	0.038	1.108	0.049	1.006	1.220	0.784	Genotyped
rs10899965	10	44,501,946	G	A	0.501	0.471	1.41E-04	1.137	0.034	1.064	1.216	0.741	Imputed
rs79901882	10	44,502,027	A	G	0.009	0.011	0.143	0.771	0.178	0.544	1.092	0.509	Imputed
rs78340246	10	44,502,163	A	G	0.015	0.013	0.088	1.267	0.139	0.965	1.665	0.850	Imputed
rs10218889	10	44,502,466	T	C	0.027	0.023	0.198	1.146	0.106	0.931	1.411	0.461	Imputed
rs76398711	10	44,502,525	A	G	0.015	0.013	0.088	1.267	0.139	0.965	1.665	0.850	Imputed
rs10899966	10	44,503,210	A	G	0.111	0.103	0.097	1.094	0.054	0.984	1.217	0.537	Imputed
rs77703727	10	44,503,754	A	G	0.034	0.030	0.119	1.157	0.094	0.963	1.390	0.282	Imputed
rs4948595	10	44,504,359	T	C	0.224	0.212	0.091	1.071	0.041	0.989	1.160	0.315	Imputed
rs4948812	10	44,504,669	C	T	0.224	0.212	0.091	1.071	0.041	0.989	1.160	0.315	Imputed
rs72782914	10	44,504,996	G	A	0.111	0.104	0.115	1.089	0.054	0.979	1.211	0.547	Imputed
rs12573239	10	44,505,025	G	A	0.224	0.212	0.091	1.071	0.041	0.989	1.160	0.315	Imputed
rs36140788	10	44,505,143	C	G	0.036	0.030	0.038	1.208	0.092	1.009	1.446	0.106	Imputed
rs75725146	10	44,505,171	T	C	0.033	0.030	0.159	1.142	0.095	0.949	1.375	0.338	Imputed
rs11238808	10	44,505,391	G	T	0.502	0.471	1.20E-04	1.139	0.034	1.066	1.217	0.807	Imputed
rs7084564	10	44,505,466	G	T	0.224	0.212	0.096	1.070	0.041	0.988	1.159	0.332	Imputed

rs10793516	10	44,505,534	G	A	0.502	0.472	1.47E-04	1.137	0.034	1.064	1.215	0.801	Imputed
rs61856549	10	44,505,718	G	A	0.013	0.012	0.703	1.059	0.150	0.789	1.421	0.350	Imputed
chr10:44506089:I	10	44,506,089	G	GTA	0.223	0.210	0.082	1.073	0.041	0.991	1.163	0.267	Imputed
chr10:44506094:I	10	44,506,094	T	TA	0.283	0.286	0.701	0.986	0.038	0.915	1.062	0.338	Imputed
rs191016282	10	44,506,279	A	G	0.014	0.011	0.098	1.268	0.144	0.956	1.681	0.360	Imputed
rs77219123	10	44,506,297	T	C	0.055	0.053	0.580	1.042	0.074	0.901	1.205	0.506	Imputed
rs1254675	10	44,506,383	C	T	0.061	0.063	0.503	0.954	0.070	0.831	1.095	0.610	Imputed
rs79680665	10	44,506,396	C	T	0.018	0.021	0.278	0.873	0.126	0.682	1.116	0.349	Imputed
rs72782918	10	44,506,857	A	G	0.026	0.023	0.315	1.114	0.108	0.902	1.377	0.585	Imputed
rs4948813	10	44,507,102	T	A	0.088	0.087	0.663	1.026	0.060	0.913	1.154	0.103	Imputed
rs17383514	10	44,507,259	G	A	0.013	0.017	0.057	0.760	0.145	0.573	1.010	0.307	Imputed
chr10:44507540:D	10	44,507,540	T	TGTG	0.026	0.023	0.321	1.113	0.108	0.901	1.375	0.590	Imputed
rs141217630	10	44,507,704	T	G	0.021	0.020	0.992	1.001	0.118	0.794	1.263	0.209	Imputed
rs145164502	10	44,507,758	A	G	0.027	0.024	0.232	1.133	0.105	0.922	1.391	0.073	Imputed
rs7074248	10	44,508,242	C	T	0.224	0.211	0.074	1.075	0.041	0.993	1.165	0.382	Imputed
rs71491006	10	44,508,583	G	C	0.035	0.029	0.011	1.263	0.093	1.054	1.515	0.188	Imputed
rs4948814	10	44,508,933	G	A	0.261	0.245	0.011	1.103	0.039	1.023	1.190	0.448	Imputed
rs10899967	10	44,509,338	A	G	0.222	0.211	0.113	1.067	0.041	0.985	1.156	0.314	Imputed
rs188104366	10	44,509,604	T	A	0.021	0.021	0.965	1.005	0.118	0.797	1.267	0.538	Imputed
rs10899968	10	44,509,620	G	C	0.222	0.211	0.113	1.067	0.041	0.985	1.156	0.314	Imputed
rs11238809	10	44,509,716	A	C	0.111	0.103	0.090	1.096	0.054	0.986	1.219	0.611	Imputed
rs10793517	10	44,509,828	C	G	0.499	0.469	1.34E-04	1.138	0.034	1.065	1.216	0.748	Imputed
rs79308829	10	44,510,131	A	G	0.094	0.091	0.513	1.039	0.058	0.927	1.164	0.413	Imputed
rs117232461	10	44,510,310	T	C	0.030	0.036	0.074	0.839	0.098	0.692	1.017	0.101	Imputed
rs10793518	10	44,510,460	A	G	0.187	0.182	0.535	1.027	0.043	0.943	1.119	0.592	Imputed
rs117258076	10	44,510,480	C	T	0.021	0.025	0.084	0.816	0.118	0.647	1.028	0.728	Imputed
rs74617223	10	44,510,893	C	T	0.015	0.013	0.124	1.240	0.140	0.942	1.632	0.984	Imputed
rs4948815	10	44,511,413	C	T	0.501	0.471	1.15E-04	1.139	0.034	1.066	1.218	0.755	Imputed
rs4948816	10	44,511,715	G	A	0.209	0.195	0.030	1.095	0.042	1.009	1.188	0.409	Imputed
rs4948596	10	44,511,734	G	A	0.209	0.195	0.030	1.095	0.042	1.009	1.188	0.409	Imputed
rs34013492	10	44,511,783	G	A	0.035	0.029	0.019	1.241	0.092	1.036	1.488	0.182	Imputed
rs4948817	10	44,511,785	C	A	0.209	0.195	0.030	1.095	0.042	1.009	1.188	0.410	Imputed
rs10899969	10	44,511,792	A	T	0.278	0.264	0.036	1.083	0.038	1.005	1.166	0.639	Imputed
rs4948818	10	44,511,815	G	C	0.500	0.469	7.48E-05	1.143	0.034	1.070	1.222	0.698	Imputed
rs4948819	10	44,511,905	A	C	0.210	0.195	0.030	1.095	0.042	1.009	1.188	0.409	Genotyped
rs1492703	10	44,512,062	T	C	0.029	0.024	0.057	1.214	0.102	0.994	1.484	0.707	Imputed
rs34942475	10	44,512,390	T	C	0.030	0.025	0.023	1.253	0.099	1.031	1.522	0.067	Imputed
rs17468466	10	44,512,416	A	T	0.115	0.106	0.066	1.103	0.053	0.994	1.225	0.597	Imputed
rs10508879	10	44,512,553	C	T	0.015	0.013	0.120	1.243	0.140	0.944	1.637	0.977	Imputed
rs1472168	10	44,512,570	G	A	0.510	0.474	5.53E-06	1.166	0.034	1.091	1.246	0.792	Imputed

rs6593388	10	44,512,749	A	G	0.186	0.173	0.056	1.087	0.044	0.998	1.184	0.951	Imputed
rs10736825	10	44,513,143	G	T	0.222	0.202	3.98E-03	1.125	0.041	1.038	1.219	0.558	Imputed
rs4948820	10	44,513,368	A	C	0.222	0.202	3.98E-03	1.125	0.041	1.038	1.219	0.558	Imputed
rs75240510	10	44,513,413	T	C	0.036	0.029	0.011	1.262	0.092	1.053	1.512	0.191	Imputed
rs4948821	10	44,513,425	G	A	0.277	0.263	0.031	1.085	0.038	1.008	1.169	0.670	Imputed
rs4948822	10	44,513,645	G	T	0.242	0.233	0.137	1.061	0.040	0.981	1.146	0.404	Genotyped
rs1352999	10	44,513,936	A	G	0.499	0.466	2.88E-05	1.152	0.034	1.078	1.231	0.875	Imputed
rs2902339	10	44,514,106	G	A	0.222	0.202	4.01E-03	1.125	0.041	1.038	1.219	0.558	Imputed
rs2185946	10	44,514,285	G	A	0.222	0.202	4.04E-03	1.125	0.041	1.038	1.219	0.559	Imputed
rs2185947	10	44,514,336	C	T	0.222	0.202	4.04E-03	1.125	0.041	1.038	1.219	0.559	Imputed
rs1602716	10	44,514,421	G	A	0.222	0.202	4.04E-03	1.125	0.041	1.038	1.219	0.559	Imputed
rs72782941	10	44,514,795	T	A	0.141	0.130	0.026	1.115	0.049	1.013	1.226	0.817	Imputed
rs1492704	10	44,515,130	T	C	0.087	0.085	0.649	1.028	0.060	0.913	1.157	0.074	Imputed
rs10899970	10	44,515,716	G	A	0.496	0.463	3.17E-05	1.151	0.034	1.077	1.230	0.750	Genotyped
rs74976165	10	44,516,025	T	C	0.035	0.030	0.063	1.187	0.093	0.990	1.423	0.249	Imputed
rs74471716	10	44,516,229	C	A	0.015	0.013	0.124	1.240	0.140	0.942	1.632	0.984	Imputed
rs7919657	10	44,516,377	A	G	0.222	0.202	4.07E-03	1.125	0.041	1.038	1.218	0.560	Imputed
rs9332446	10	44,516,421	G	A	0.515	0.479	4.21E-06	1.169	0.034	1.093	1.249	0.793	Imputed
rs71491007	10	44,516,475	T	C	0.036	0.029	0.011	1.263	0.092	1.054	1.514	0.189	Imputed
rs10899971	10	44,516,604	C	T	0.500	0.466	2.42E-05	1.154	0.034	1.080	1.233	0.874	Imputed
rs7903881	10	44,516,610	C	T	0.222	0.202	4.10E-03	1.124	0.041	1.038	1.218	0.561	Imputed
rs11238813	10	44,516,908	A	C	0.089	0.086	0.447	1.046	0.060	0.931	1.176	0.303	Imputed
rs7908204	10	44,517,536	T	C	0.278	0.264	0.034	1.084	0.038	1.006	1.167	0.635	Genotyped
rs1388975	10	44,517,790	G	A	0.222	0.202	4.41E-03	1.123	0.041	1.037	1.217	0.519	Imputed
rs114441762	10	44,518,010	A	G	0.053	0.051	0.502	1.052	0.075	0.907	1.220	0.459	Imputed
rs77896782	10	44,518,268	C	A	0.015	0.013	0.129	1.237	0.140	0.940	1.628	0.915	Imputed
rs1492705	10	44,518,492	C	G	0.238	0.216	1.56E-03	1.134	0.040	1.049	1.227	0.510	Imputed
rs955582	10	44,518,652	G	A	0.222	0.202	4.48E-03	1.123	0.041	1.037	1.217	0.521	Imputed
rs955583	10	44,518,696	G	A	0.222	0.202	4.22E-03	1.124	0.041	1.037	1.218	0.564	Imputed
rs955584	10	44,518,915	C	A	0.500	0.466	2.39E-05	1.154	0.034	1.080	1.233	0.873	Imputed
rs34029778	10	44,518,962	G	A	0.036	0.029	9.77E-03	1.267	0.092	1.058	1.517	0.226	Imputed
rs17154903	10	44,519,408	T	C	0.015	0.013	0.174	1.209	0.140	0.919	1.591	0.901	Genotyped
rs2862837	10	44,519,687	T	G	0.278	0.263	0.030	1.086	0.038	1.008	1.169	0.690	Imputed
rs1353000	10	44,519,876	T	G	0.278	0.263	0.030	1.086	0.038	1.008	1.169	0.690	Imputed
rs17468732	10	44,520,099	C	G	0.013	0.010	0.104	1.283	0.154	0.949	1.733	0.835	Imputed
rs6593389	10	44,520,215	T	C	0.278	0.263	0.030	1.086	0.038	1.008	1.169	0.690	Imputed
rs60719295	10	44,520,231	T	C	0.242	0.232	0.128	1.062	0.040	0.983	1.148	0.363	Imputed
rs6593390	10	44,520,349	A	G	0.278	0.263	0.030	1.086	0.038	1.008	1.169	0.690	Imputed
rs6593391	10	44,520,403	A	C	0.035	0.030	0.061	1.188	0.093	0.991	1.424	0.247	Genotyped
rs76725314	10	44,520,424	T	C	0.186	0.173	0.059	1.086	0.044	0.997	1.183	0.961	Imputed

rs6593393	10	44,520,658	C	T	0.186	0.173	0.059	1.086	0.044	0.997	1.183	0.961	Imputed
rs71491008	10	44,520,791	T	C	0.061	0.063	0.715	0.975	0.071	0.849	1.119	0.241	Imputed
rs10899972	10	44,520,836	C	G	0.142	0.130	0.026	1.115	0.049	1.013	1.227	0.841	Imputed
rs56803324	10	44,520,886	T	C	0.087	0.085	0.649	1.028	0.060	0.913	1.157	0.079	Imputed
rs955826	10	44,521,222	C	A	0.278	0.263	0.030	1.086	0.038	1.008	1.169	0.690	Imputed
rs61856550	10	44,521,524	T	C	0.056	0.057	0.608	0.963	0.074	0.834	1.112	0.854	Imputed
rs1388977	10	44,521,561	C	G	0.013	0.017	0.074	0.773	0.145	0.582	1.027	0.210	Imputed
rs1492706	10	44,521,578	T	A	0.278	0.263	0.030	1.086	0.038	1.008	1.169	0.689	Imputed
rs76095133	10	44,521,669	A	G	0.015	0.013	0.125	1.240	0.140	0.942	1.632	0.909	Imputed
rs72782960	10	44,521,919	T	C	0.026	0.024	0.366	1.101	0.107	0.893	1.359	0.385	Imputed
rs77695516	10	44,521,943	T	G	0.018	0.021	0.202	0.850	0.127	0.663	1.091	0.412	Imputed
chr10:44522079:I	10	44,522,079	TAA	T	0.015	0.013	0.099	1.258	0.139	0.957	1.653	0.871	Imputed
rs150749671	10	44,522,120	A	G	0.008	0.011	0.292	0.825	0.183	0.577	1.180	0.760	Imputed
rs17154915	10	44,522,596	G	A	0.087	0.085	0.645	1.028	0.060	0.914	1.157	0.080	Imputed
rs7073647	10	44,522,975	A	T	0.034	0.029	0.058	1.195	0.095	0.993	1.439	0.296	Imputed
rs10899973	10	44,523,504	T	C	0.515	0.479	3.86E-06	1.169	0.034	1.094	1.249	0.772	Imputed
rs17154922	10	44,523,587	C	T	0.278	0.263	0.029	1.086	0.038	1.008	1.170	0.692	Imputed
rs188106451	10	44,523,588	A	G	0.018	0.013	0.016	1.371	0.131	1.060	1.774	0.955	Imputed
rs55719822	10	44,524,214	C	G	0.026	0.023	0.341	1.107	0.107	0.897	1.367	0.452	Imputed
rs78947610	10	44,524,262	A	G	0.034	0.029	0.099	1.168	0.094	0.971	1.406	0.362	Imputed
rs10793519	10	44,524,465	G	C	0.277	0.262	0.023	1.090	0.038	1.012	1.174	0.701	Imputed
rs17154929	10	44,524,675	T	C	0.015	0.013	0.125	1.240	0.140	0.942	1.632	0.909	Imputed
rs7095296	10	44,525,115	A	G	0.034	0.030	0.108	1.163	0.094	0.967	1.400	0.374	Imputed
rs10508880	10	44,525,123	A	C	0.087	0.085	0.645	1.028	0.060	0.914	1.157	0.080	Imputed
rs34224281	10	44,525,213	T	C	0.036	0.029	0.011	1.263	0.092	1.054	1.514	0.189	Imputed
rs10899974	10	44,525,938	G	C	0.242	0.232	0.132	1.061	0.040	0.982	1.147	0.347	Imputed
rs145935960	10	44,526,279	T	C	0.008	0.011	0.292	0.825	0.183	0.577	1.180	0.760	Imputed
rs17154938	10	44,526,783	C	T	0.015	0.013	0.125	1.240	0.140	0.942	1.632	0.909	Imputed
rs71491009	10	44,526,909	C	T	0.036	0.029	0.011	1.263	0.092	1.054	1.514	0.189	Imputed
rs17384177	10	44,527,028	A	T	0.142	0.130	0.026	1.115	0.049	1.013	1.227	0.841	Imputed
rs75568040	10	44,527,038	A	G	0.015	0.013	0.095	1.261	0.139	0.960	1.657	0.865	Imputed
rs1492711	10	44,527,132	C	T	0.222	0.202	4.13E-03	1.124	0.041	1.038	1.218	0.512	Imputed
rs75446270	10	44,527,150	G	A	0.087	0.085	0.645	1.028	0.060	0.914	1.157	0.080	Imputed
rs72782973	10	44,527,309	C	A	0.142	0.130	0.026	1.115	0.049	1.013	1.227	0.841	Imputed
rs1492710	10	44,527,328	G	A	0.278	0.263	0.027	1.087	0.038	1.010	1.171	0.705	Imputed
rs1492709	10	44,527,366	C	T	0.278	0.263	0.029	1.086	0.038	1.008	1.170	0.692	Imputed
rs1492708	10	44,527,426	A	C	0.277	0.263	0.032	1.085	0.038	1.007	1.168	0.680	Imputed
rs11238817	10	44,527,590	C	T	0.515	0.479	4.24E-06	1.168	0.034	1.093	1.249	0.731	Imputed
rs1492707	10	44,527,733	A	G	0.278	0.263	0.030	1.086	0.038	1.008	1.169	0.696	Imputed
rs181220122	10	44,527,767	T	C	0.011	0.010	0.363	1.160	0.163	0.843	1.594	0.527	Imputed

rs956111	10	44,528,090	C	G	0.015	0.013	0.125	1.240	0.140	0.942	1.632	0.909	Imputed
rs950682	10	44,528,099	G	A	0.087	0.085	0.642	1.028	0.060	0.914	1.157	0.080	Imputed
chr10:44529212:I	10	44,529,212	G	GT	0.213	0.197	0.017	1.104	0.041	1.018	1.198	0.448	Imputed
rs12570314	10	44,529,320	A	G	0.499	0.465	2.54E-05	1.153	0.034	1.079	1.232	0.828	Imputed
rs1833024	10	44,530,124	G	A	0.121	0.114	0.230	1.064	0.052	0.961	1.179	0.707	Imputed
rs74879622	10	44,530,366	T	A	0.015	0.013	0.125	1.240	0.140	0.942	1.632	0.909	Imputed
rs75182140	10	44,530,522	A	C	0.036	0.029	0.011	1.263	0.092	1.054	1.514	0.189	Imputed
rs10793520	10	44,530,588	G	T	0.277	0.262	0.023	1.090	0.038	1.012	1.174	0.652	Imputed
rs80116876	10	44,530,678	A	G	0.036	0.029	0.011	1.263	0.092	1.054	1.514	0.189	Imputed
rs11238818	10	44,530,978	G	A	0.515	0.479	4.89E-06	1.167	0.034	1.092	1.247	0.743	Imputed
rs11238819	10	44,532,117	T	C	0.115	0.106	0.065	1.103	0.053	0.994	1.225	0.620	Imputed
rs1492712	10	44,532,138	C	T	0.277	0.262	0.024	1.089	0.038	1.011	1.173	0.562	Genotyped
rs1845050	10	44,532,446	A	G	0.041	0.032	1.53E-03	1.316	0.087	1.110	1.561	0.978	Imputed
rs75407615	10	44,532,876	G	A	0.053	0.051	0.444	1.059	0.075	0.914	1.228	0.516	Imputed
rs10899975	10	44,532,885	C	A	0.278	0.263	0.026	1.088	0.038	1.010	1.172	0.609	Imputed
rs78107691	10	44,532,942	A	G	0.012	0.016	0.056	0.752	0.150	0.560	1.009	0.347	Imputed
rs149771612	10	44,533,609	A	G	0.137	0.126	0.030	1.113	0.049	1.011	1.227	0.791	Imputed
rs187157934	10	44,533,616	G	A	0.087	0.085	0.626	1.030	0.060	0.915	1.159	0.050	Imputed
rs145731205	10	44,533,655	G	T	0.468	0.504	6.59E-06	0.858	0.034	0.803	0.917	0.913	Imputed
rs149003742	10	44,533,988	T	G	0.205	0.196	0.119	1.068	0.042	0.983	1.160	0.919	Imputed
rs9732443	10	44,534,180	T	C	0.250	0.233	0.015	1.100	0.039	1.018	1.188	0.792	Imputed
rs188871832	10	44,534,652	T	C	0.015	0.013	0.108	1.253	0.140	0.951	1.649	0.957	Imputed
rs7475017	10	44,534,720	T	C	0.243	0.228	0.050	1.081	0.040	1.000	1.168	0.467	Imputed
chr10:44534764:D	10	44,534,764	A	AAAT	0.040	0.035	0.076	1.166	0.087	0.984	1.383	0.404	Imputed
rs1254676	10	44,534,764	T	A	0.013	0.014	0.667	0.939	0.148	0.703	1.253	0.884	Imputed
rs12415001	10	44,534,767	A	T	0.075	0.069	0.184	1.090	0.065	0.960	1.237	0.743	Imputed
rs6593394	10	44,535,508	A	G	0.259	0.236	1.84E-03	1.128	0.039	1.046	1.217	0.285	Imputed
chr10:44535778:I	10	44,535,778	AG	A	0.034	0.029	0.057	1.194	0.094	0.994	1.435	0.306	Imputed
rs11238822	10	44,535,833	G	C	0.499	0.465	2.37E-05	1.154	0.034	1.080	1.233	0.899	Imputed
rs79194564	10	44,536,092	A	C	0.035	0.030	0.038	1.211	0.093	1.010	1.452	0.274	Imputed
rs1891883	10	44,536,318	A	G	0.223	0.204	5.43E-03	1.120	0.041	1.034	1.213	0.499	Imputed
rs79671379	10	44,536,443	G	A	0.123	0.116	0.140	1.080	0.052	0.975	1.195	0.308	Imputed
rs11238823	10	44,536,722	G	A	0.265	0.247	5.17E-03	1.114	0.038	1.033	1.201	0.390	Imputed
chr10:44536796:D	10	44,536,796	A	AATAG	0.265	0.247	5.17E-03	1.114	0.038	1.033	1.201	0.390	Imputed
rs72782990	10	44,537,033	T	C	0.265	0.247	5.31E-03	1.113	0.038	1.032	1.200	0.388	Imputed
rs72782991	10	44,537,078	T	C	0.112	0.104	0.074	1.101	0.054	0.991	1.223	0.642	Imputed
rs7917534	10	44,537,423	G	C	0.221	0.202	5.16E-03	1.121	0.041	1.035	1.215	0.500	Imputed
rs111688882	10	44,537,662	A	T	0.013	0.017	0.075	0.772	0.146	0.580	1.028	0.277	Imputed
rs4615977	10	44,537,706	A	G	0.221	0.202	5.13E-03	1.121	0.041	1.035	1.215	0.500	Imputed
rs7897744	10	44,537,892	A	G	0.278	0.263	0.026	1.088	0.038	1.010	1.171	0.606	Imputed

rs17154976	10	44,537,960	T	G	0.013	0.017	0.075	0.772	0.146	0.580	1.028	0.277	Imputed
rs2051120	10	44,538,506	T	G	0.185	0.173	0.057	1.087	0.044	0.997	1.184	0.909	Imputed
rs11238824	10	44,538,602	G	C	0.155	0.148	0.191	1.063	0.047	0.970	1.165	0.740	Imputed
rs1565661	10	44,538,622	A	C	0.278	0.262	0.022	1.090	0.038	1.012	1.174	0.602	Imputed
rs1565660	10	44,538,672	A	G	0.221	0.202	5.13E-03	1.121	0.041	1.035	1.215	0.500	Imputed
rs7903121	10	44,538,921	C	T	0.515	0.479	4.81E-06	1.167	0.034	1.092	1.248	0.867	Imputed
rs1565659	10	44,538,935	A	G	0.035	0.030	0.038	1.211	0.093	1.010	1.452	0.274	Imputed
rs1873757	10	44,539,016	G	A	0.499	0.465	2.29E-05	1.154	0.034	1.080	1.233	0.881	Imputed
rs11238825	10	44,539,019	G	T	0.141	0.130	0.033	1.110	0.049	1.008	1.221	0.845	Imputed
rs7923238	10	44,539,457	C	T	0.123	0.115	0.105	1.088	0.052	0.983	1.204	0.303	Imputed
rs11238826	10	44,539,579	T	C	0.278	0.263	0.029	1.086	0.038	1.008	1.170	0.591	Imputed
rs2047009	10	44,539,913	T	G	0.515	0.479	5.76E-06	1.166	0.034	1.091	1.246	0.834	Genotyped
rs146069355	10	44,540,177	T	C	0.012	0.013	0.729	0.947	0.157	0.695	1.289	0.277	Imputed
rs6593395	10	44,540,495	T	C	0.141	0.130	0.036	1.108	0.049	1.007	1.219	0.830	Imputed
rs79651966	10	44,540,702	G	T	0.015	0.013	0.176	1.206	0.139	0.919	1.583	0.837	Imputed
rs79232840	10	44,540,905	G	T	0.033	0.029	0.117	1.160	0.095	0.963	1.397	0.290	Imputed
rs72784804	10	44,541,444	T	C	0.011	0.010	0.468	1.124	0.161	0.820	1.540	0.975	Imputed
rs10508881	10	44,541,565	A	G	0.443	0.403	3.60E-07	1.189	0.034	1.113	1.272	0.632	Genotyped
rs4948823	10	44,541,676	C	G	0.088	0.086	0.581	1.034	0.060	0.919	1.163	0.055	Imputed
rs9804352	10	44,541,780	A	G	0.439	0.400	6.54E-07	1.185	0.034	1.108	1.267	0.668	Imputed
rs61856552	10	44,542,522	T	A	0.013	0.012	0.715	1.056	0.150	0.788	1.417	0.547	Imputed
rs74337803	10	44,542,714	A	C	0.015	0.013	0.138	1.231	0.140	0.935	1.620	0.929	Imputed
rs17154988	10	44,542,848	G	T	0.088	0.086	0.591	1.033	0.060	0.918	1.162	0.054	Imputed
rs78892197	10	44,543,257	G	A	0.088	0.085	0.568	1.035	0.060	0.920	1.164	0.056	Imputed
rs72784807	10	44,543,293	T	C	0.026	0.024	0.421	1.090	0.108	0.883	1.347	0.536	Imputed
rs12266297	10	44,543,714	G	A	0.064	0.065	0.759	0.979	0.069	0.856	1.121	0.596	Imputed
chr10:44543777:D	10	44,543,777	T	TTTTG	0.015	0.013	0.127	1.236	0.139	0.941	1.624	0.917	Imputed
rs1114215	10	44,544,575	T	A	0.015	0.013	0.143	1.228	0.140	0.933	1.616	0.935	Imputed
rs1114216	10	44,544,726	C	A	0.015	0.013	0.170	1.209	0.139	0.921	1.588	0.902	Imputed
rs2639469	10	44,544,935	C	A	0.225	0.205	2.19E-03	1.133	0.041	1.046	1.227	0.309	Genotyped
rs7898618	10	44,545,096	T	C	0.088	0.085	0.559	1.036	0.060	0.921	1.165	0.049	Imputed
rs2639468	10	44,545,164	A	G	0.223	0.202	1.50E-03	1.138	0.041	1.051	1.233	0.277	Imputed
rs77433193	10	44,545,193	T	C	0.009	0.011	0.161	0.779	0.178	0.550	1.104	0.482	Imputed
rs55810528	10	44,545,266	A	G	0.025	0.023	0.450	1.086	0.109	0.877	1.345	0.672	Imputed
rs2818916	10	44,546,022	A	G	0.222	0.204	4.64E-03	1.122	0.041	1.036	1.216	0.221	Imputed
rs74629040	10	44,546,822	T	C	0.041	0.041	0.894	1.011	0.085	0.857	1.194	0.227	Imputed
rs2624688	10	44,547,210	T	C	0.183	0.167	0.013	1.116	0.044	1.024	1.216	0.633	Imputed
rs7100747	10	44,547,255	G	A	0.042	0.042	0.978	1.002	0.085	0.849	1.183	0.232	Imputed
rs11238828	10	44,547,357	A	G	0.022	0.020	0.461	1.090	0.117	0.867	1.370	0.979	Imputed
rs71491012	10	44,547,394	A	G	0.032	0.026	0.025	1.242	0.097	1.027	1.502	0.256	Imputed

rs77419579	10	44,547,504	T	C	0.088	0.085	0.533	1.038	0.060	0.923	1.168	0.051	Imputed
rs7096716	10	44,548,259	A	C	0.088	0.085	0.555	1.036	0.060	0.921	1.166	0.049	Imputed
chr10:44548705:I	10	44,548,705	AT	A	0.088	0.085	0.478	1.044	0.060	0.928	1.174	0.040	Imputed
rs142050245	10	44,548,767	T	G	0.018	0.016	0.129	1.213	0.127	0.945	1.556	0.764	Imputed
chr10:44548870:I	10	44,548,870	GA	G	0.017	0.014	0.116	1.234	0.134	0.949	1.603	0.997	Imputed
rs11598807	10	44,549,239	G	A	0.062	0.063	0.615	0.965	0.070	0.841	1.108	0.485	Imputed
rs268330	10	44,549,754	A	G	0.013	0.017	0.054	0.754	0.147	0.565	1.007	0.290	Imputed
rs2624695	10	44,549,767	T	C	0.463	0.496	2.86E-05	0.868	0.034	0.812	0.927	0.652	Imputed
rs268329	10	44,549,886	A	G	0.013	0.017	0.058	0.759	0.146	0.570	1.011	0.424	Genotyped
rs138088010	10	44,550,015	T	C	0.011	0.013	0.412	0.878	0.159	0.644	1.198	0.660	Imputed
rs12412267	10	44,550,276	A	G	0.068	0.067	0.707	1.026	0.067	0.899	1.170	0.725	Imputed
chr10:44550497:I	10	44,550,497	TG	T	0.082	0.076	0.202	1.082	0.062	0.958	1.222	0.388	Imputed
chr10:44550502:D	10	44,550,502	G	GGGGC	0.150	0.137	0.021	1.116	0.048	1.017	1.226	0.691	Imputed
rs59056359	10	44,550,502	A	G	0.017	0.018	0.818	0.971	0.130	0.752	1.253	0.073	Imputed
chr10:44550503:D	10	44,550,503	G	GGGC	0.208	0.195	0.042	1.089	0.042	1.003	1.182	0.102	Imputed
rs268328	10	44,550,504	A	G	0.237	0.210	1.01E-04	1.168	0.040	1.080	1.263	0.614	Imputed
rs148255421	10	44,550,597	A	G	0.022	0.019	0.155	1.178	0.116	0.939	1.478	0.206	Imputed
rs2128363	10	44,550,715	G	A	0.462	0.495	3.81E-05	0.870	0.034	0.814	0.929	0.683	Imputed
rs35484462	10	44,551,225	C	T	0.103	0.095	0.085	1.101	0.056	0.987	1.228	0.305	Imputed
rs78238737	10	44,551,252	G	C	0.058	0.059	0.848	0.986	0.072	0.857	1.136	0.522	Imputed
rs2624694	10	44,551,328	T	C	0.462	0.496	2.06E-05	0.866	0.034	0.810	0.925	0.700	Imputed
chr10:44551837:D	10	44,551,837	G	GC	0.333	0.302	8.02E-05	1.152	0.036	1.074	1.237	0.331	Imputed
chr10:44551841:D	10	44,551,841	G	GT	0.333	0.302	8.02E-05	1.152	0.036	1.074	1.237	0.331	Imputed
rs111385732	10	44,551,842	G	T	0.332	0.302	1.04E-04	1.150	0.036	1.072	1.234	0.318	Imputed
rs2802492	10	44,552,242	G	A	0.366	0.338	3.05E-04	1.135	0.035	1.060	1.217	0.172	Imputed
rs268327	10	44,553,000	T	C	0.033	0.034	0.803	0.977	0.095	0.811	1.176	0.305	Imputed
rs268326	10	44,553,202	T	C	0.334	0.304	7.95E-05	1.152	0.036	1.074	1.237	0.303	Imputed
rs75676906	10	44,553,233	A	C	0.116	0.116	0.823	1.012	0.053	0.912	1.122	0.040	Imputed
rs268325	10	44,553,342	A	C	0.333	0.303	9.15E-05	1.151	0.036	1.073	1.235	0.303	Genotyped
rs268324	10	44,553,532	T	C	0.013	0.017	0.042	0.741	0.148	0.554	0.991	0.199	Imputed
rs2802493	10	44,553,583	C	T	0.366	0.338	2.99E-04	1.136	0.035	1.060	1.217	0.180	Genotyped
rs143648341	10	44,554,340	A	G	0.024	0.026	0.424	0.916	0.110	0.738	1.137	0.143	Imputed
rs268323	10	44,554,677	C	G	0.332	0.303	1.53E-04	1.146	0.036	1.068	1.230	0.315	Imputed
rs74464319	10	44,555,094	T	G	0.058	0.056	0.507	1.049	0.072	0.911	1.209	0.637	Imputed
rs141990551	10	44,555,140	A	G	0.017	0.013	0.162	1.206	0.134	0.927	1.569	0.626	Imputed
rs78708084	10	44,556,043	T	C	0.116	0.116	0.808	1.013	0.053	0.913	1.124	0.041	Imputed
rs77179512	10	44,556,188	A	T	0.020	0.017	0.100	1.221	0.122	0.962	1.551	0.855	Imputed
rs75875295	10	44,556,530	A	T	0.059	0.056	0.423	1.059	0.072	0.920	1.220	0.682	Imputed
rs268322	10	44,556,602	T	C	0.092	0.091	0.659	1.026	0.058	0.915	1.151	0.331	Imputed
rs268321	10	44,556,742	C	T	0.092	0.091	0.671	1.025	0.058	0.914	1.149	0.335	Imputed

rs187901	10	44,557,502	T	C	0.093	0.091	0.616	1.030	0.058	0.918	1.154	0.373	Genotyped
rs2804029	10	44,558,309	T	C	0.459	0.495	8.71E-06	0.860	0.034	0.805	0.919	0.713	Imputed
rs79888387	10	44,558,327	T	C	0.081	0.075	0.164	1.090	0.062	0.965	1.231	0.288	Imputed
rs268320	10	44,558,546	T	C	0.013	0.017	0.042	0.741	0.148	0.554	0.991	0.199	Imputed
rs34743310	10	44,558,937	G	A	0.018	0.019	0.691	0.951	0.126	0.742	1.218	0.895	Imputed
rs268319	10	44,558,945	A	G	0.115	0.110	0.313	1.055	0.053	0.951	1.171	0.208	Genotyped
rs61856554	10	44,558,976	G	C	0.021	0.020	0.968	0.995	0.119	0.788	1.256	0.811	Imputed
rs7088907	10	44,558,990	A	G	0.152	0.147	0.291	1.051	0.047	0.958	1.153	0.072	Imputed
rs268318	10	44,559,086	T	C	0.013	0.017	0.045	0.744	0.149	0.556	0.995	0.195	Imputed
rs77736290	10	44,559,188	G	A	0.020	0.017	0.103	1.219	0.122	0.960	1.548	0.850	Imputed
rs182076	10	44,559,393	C	T	0.115	0.110	0.331	1.053	0.053	0.949	1.168	0.148	Imputed
rs17155030	10	44,559,434	A	G	0.116	0.115	0.756	1.017	0.053	0.917	1.128	0.028	Imputed
rs268317	10	44,559,713	A	G	0.037	0.028	4.05E-04	1.381	0.091	1.154	1.652	0.613	Imputed
rs72784819	10	44,560,005	T	C	0.036	0.032	0.127	1.150	0.092	0.961	1.376	0.784	Imputed
rs268316	10	44,560,053	G	T	0.013	0.017	0.057	0.757	0.147	0.567	1.011	0.283	Imputed
rs2818904	10	44,560,319	G	A	0.459	0.494	1.17E-05	0.862	0.034	0.806	0.921	0.734	Imputed
rs77421689	10	44,560,469	C	A	0.037	0.036	0.815	1.021	0.089	0.857	1.216	0.053	Imputed
rs268315	10	44,560,607	A	G	0.013	0.017	0.057	0.757	0.147	0.567	1.011	0.283	Imputed
rs117738545	10	44,560,620	T	C	0.019	0.022	0.254	0.870	0.122	0.684	1.105	0.272	Imputed
rs268314	10	44,561,651	A	G	0.229	0.223	0.323	1.041	0.040	0.962	1.126	0.595	Genotyped
rs17155040	10	44,561,922	A	G	0.020	0.017	0.126	1.206	0.122	0.949	1.533	0.818	Imputed
rs78503841	10	44,562,429	A	G	0.020	0.017	0.126	1.206	0.122	0.949	1.533	0.818	Imputed
rs17155044	10	44,562,490	C	G	0.020	0.017	0.126	1.206	0.122	0.949	1.533	0.818	Imputed
rs268313	10	44,562,645	T	C	0.114	0.110	0.349	1.051	0.053	0.947	1.167	0.177	Imputed
rs7898629	10	44,562,687	A	G	0.081	0.075	0.180	1.087	0.062	0.962	1.227	0.300	Genotyped
rs7919249	10	44,562,831	C	T	0.081	0.075	0.189	1.085	0.062	0.961	1.225	0.307	Imputed
rs75396296	10	44,562,999	T	C	0.020	0.017	0.126	1.206	0.122	0.949	1.533	0.818	Imputed
rs74231460	10	44,563,504	G	A	0.020	0.017	0.126	1.206	0.122	0.949	1.533	0.818	Imputed
rs268312	10	44,563,530	G	A	0.114	0.110	0.416	1.044	0.053	0.941	1.159	0.162	Imputed
rs79372555	10	44,563,991	G	A	0.104	0.101	0.598	1.030	0.056	0.923	1.149	0.018	Imputed
rs12572132	10	44,564,178	C	T	0.117	0.108	0.040	1.115	0.053	1.005	1.236	0.637	Genotyped
rs75758167	10	44,564,255	G	T	0.081	0.075	0.169	1.089	0.062	0.964	1.230	0.320	Imputed
rs182075	10	44,564,364	G	A	0.114	0.109	0.333	1.053	0.053	0.948	1.169	0.187	Imputed
rs11817296	10	44,565,194	G	T	0.150	0.144	0.235	1.058	0.048	0.964	1.162	0.068	Imputed
rs2639463	10	44,566,061	T	C	0.464	0.502	5.21E-06	0.857	0.034	0.802	0.916	0.782	Imputed
rs1938557	10	44,566,383	A	G	0.134	0.129	0.466	1.037	0.050	0.941	1.143	0.316	Imputed
rs79544393	10	44,566,978	T	G	0.020	0.017	0.142	1.197	0.122	0.942	1.521	0.864	Imputed
rs61856555	10	44,567,182	G	A	0.101	0.095	0.287	1.062	0.056	0.951	1.186	0.444	Imputed
rs117404811	10	44,567,315	C	T	0.020	0.022	0.341	0.891	0.121	0.703	1.129	0.322	Imputed
rs61856556	10	44,567,398	T	A	0.101	0.095	0.290	1.062	0.056	0.950	1.186	0.446	Imputed

rs268287	10	44,567,421	A	G	0.214	0.186	2.59E-05	1.191	0.042	1.098	1.292	0.890	Imputed
rs141674893	10	44,567,437	C	T	0.008	0.010	0.359	0.846	0.183	0.592	1.210	0.882	Imputed
rs187900	10	44,567,765	A	G	0.134	0.131	0.550	1.030	0.050	0.935	1.136	0.313	Genotyped
rs75387338	10	44,568,143	A	T	0.042	0.039	0.458	1.064	0.084	0.902	1.256	0.354	Imputed
rs268286	10	44,568,488	C	T	0.033	0.035	0.615	0.954	0.094	0.793	1.147	0.503	Imputed
rs117373101	10	44,568,952	C	A	0.020	0.022	0.329	0.888	0.121	0.701	1.126	0.345	Imputed
rs1580387	10	44,568,960	C	T	0.013	0.017	0.032	0.732	0.147	0.548	0.976	0.317	Imputed
rs12359076	10	44,569,041	A	G	0.115	0.103	0.012	1.143	0.053	1.030	1.269	0.619	Imputed
rs2804027	10	44,569,665	C	T	0.465	0.502	4.26E-06	0.856	0.034	0.801	0.915	0.841	Imputed
rs268285	10	44,569,670	A	T	0.134	0.130	0.493	1.035	0.050	0.939	1.141	0.314	Imputed
rs2639465	10	44,569,677	C	T	0.465	0.502	4.90E-06	0.857	0.034	0.801	0.915	0.853	Imputed
rs11517247	10	44,569,796	T	C	0.102	0.096	0.279	1.063	0.056	0.952	1.186	0.432	Imputed
rs117879379	10	44,569,894	A	C	0.023	0.024	0.682	0.955	0.113	0.766	1.191	0.265	Imputed
rs76685063	10	44,569,965	T	C	0.018	0.019	0.568	0.929	0.129	0.722	1.195	0.039	Imputed
rs117614020	10	44,570,030	C	G	0.012	0.016	0.028	0.716	0.153	0.531	0.967	0.379	Imputed
rs268284	10	44,570,165	A	G	0.013	0.017	0.032	0.732	0.147	0.548	0.976	0.317	Imputed
rs268283	10	44,570,969	T	G	0.134	0.130	0.497	1.034	0.050	0.938	1.140	0.316	Imputed
rs268282	10	44,571,489	C	T	0.134	0.130	0.492	1.035	0.050	0.939	1.141	0.323	Imputed
rs268281	10	44,572,160	C	T	0.134	0.130	0.489	1.035	0.050	0.939	1.141	0.322	Imputed
rs189723	10	44,572,403	T	C	0.213	0.186	2.60E-05	1.191	0.042	1.098	1.292	0.889	Imputed
rs139244001	10	44,572,588	A	G	0.037	0.036	0.676	1.038	0.089	0.871	1.237	0.128	Imputed
rs34931306	10	44,572,948	A	G	0.036	0.032	0.117	1.153	0.091	0.965	1.379	0.641	Imputed
rs9422581	10	44,572,992	C	T	0.113	0.107	0.215	1.069	0.054	0.962	1.187	0.655	Imputed
rs268280	10	44,573,044	G	A	0.134	0.130	0.483	1.035	0.050	0.939	1.141	0.320	Imputed
rs150766493	10	44,573,140	A	G	0.020	0.017	0.132	1.201	0.122	0.946	1.525	0.749	Imputed
rs174848	10	44,573,494	A	T	0.134	0.130	0.530	1.032	0.050	0.936	1.137	0.364	Imputed
rs61856557	10	44,573,572	G	C	0.101	0.095	0.300	1.060	0.056	0.949	1.184	0.519	Imputed
rs268279	10	44,573,597	T	C	0.134	0.129	0.420	1.041	0.050	0.944	1.148	0.374	Imputed
rs268278	10	44,574,013	A	G	0.013	0.017	0.032	0.732	0.147	0.548	0.976	0.317	Imputed
rs268277	10	44,574,303	C	T	0.134	0.130	0.544	1.031	0.050	0.935	1.136	0.356	Imputed
rs268276	10	44,574,542	C	T	0.125	0.125	0.896	1.007	0.051	0.911	1.113	0.326	Imputed
rs268275	10	44,574,639	G	A	0.134	0.130	0.519	1.033	0.050	0.937	1.138	0.359	Imputed
rs11238833	10	44,574,805	G	T	0.101	0.095	0.306	1.059	0.056	0.949	1.183	0.504	Imputed
rs77304113	10	44,575,702	G	A	0.017	0.015	0.151	1.206	0.131	0.933	1.558	0.982	Imputed
rs75933787	10	44,576,017	C	G	0.020	0.017	0.159	1.188	0.122	0.935	1.510	0.909	Imputed
rs2086625	10	44,576,165	G	C	0.134	0.131	0.525	1.032	0.050	0.936	1.138	0.349	Imputed
rs74467584	10	44,576,195	A	T	0.113	0.110	0.560	1.032	0.054	0.929	1.146	0.017	Imputed
rs2804024	10	44,576,255	A	G	0.251	0.223	6.03E-05	1.170	0.039	1.084	1.264	0.380	Imputed
rs35611843	10	44,576,525	A	T	0.036	0.032	0.100	1.162	0.091	0.971	1.389	0.653	Imputed
rs2624679	10	44,576,626	A	G	0.098	0.083	1.60E-03	1.198	0.057	1.071	1.341	0.450	Imputed

rs11238835	10	44,577,326	A	G	0.101	0.096	0.330	1.056	0.056	0.946	1.179	0.551	Imputed
rs2818911	10	44,577,495	C	T	0.067	0.065	0.530	1.043	0.068	0.914	1.191	0.608	Imputed
rs76201301	10	44,577,906	A	G	0.079	0.074	0.254	1.074	0.063	0.950	1.215	0.393	Imputed
rs150364194	10	44,578,325	G	C	0.023	0.024	0.640	0.949	0.113	0.761	1.183	0.256	Imputed
rs931225	10	44,578,575	G	A	0.134	0.131	0.524	1.032	0.050	0.936	1.138	0.348	Imputed
rs1254530	10	44,578,967	C	T	0.134	0.130	0.544	1.031	0.050	0.935	1.136	0.356	Imputed
rs1254531	10	44,579,062	T	C	0.134	0.130	0.537	1.031	0.050	0.935	1.137	0.367	Genotyped
chr10:44579248:D	10	44,579,248	G	GC	0.478	0.449	2.39E-04	1.133	0.034	1.060	1.211	0.339	Imputed
chr10:44579249:D	10	44,579,249	CA	C	0.465	0.501	7.42E-06	0.859	0.034	0.804	0.918	0.955	Imputed
chr10:44579252:D	10	44,579,252	A	AT	0.458	0.432	1.19E-03	1.117	0.034	1.045	1.195	0.915	Imputed
rs61855683	10	44,579,609	T	C	0.101	0.095	0.312	1.059	0.056	0.948	1.182	0.508	Imputed
rs268299	10	44,580,627	G	T	0.033	0.035	0.686	0.963	0.094	0.800	1.158	0.502	Imputed
chr10:44580655:I	10	44,580,655	TA	T	0.037	0.036	0.726	1.032	0.089	0.866	1.229	0.126	Imputed
rs268300	10	44,580,839	A	G	0.033	0.035	0.615	0.954	0.094	0.793	1.147	0.502	Genotyped
rs1384333	10	44,580,847	T	C	0.101	0.095	0.310	1.059	0.056	0.948	1.183	0.507	Imputed
rs79367776	10	44,581,386	A	T	0.037	0.036	0.719	1.033	0.089	0.867	1.230	0.125	Imputed
rs268301	10	44,581,425	G	A	0.047	0.048	0.991	1.001	0.080	0.857	1.170	0.528	Imputed
rs75770699	10	44,581,432	A	G	0.020	0.017	0.142	1.197	0.122	0.941	1.521	0.930	Imputed
chr10:44581476:D	10	44,581,476	C	CT	0.013	0.013	0.618	1.076	0.147	0.806	1.435	0.184	Imputed
rs268302	10	44,581,485	A	T	0.044	0.048	0.315	0.921	0.082	0.785	1.081	0.496	Imputed
rs268303	10	44,581,562	A	T	0.250	0.223	8.01E-05	1.167	0.039	1.081	1.261	0.399	Imputed
rs268304	10	44,581,683	G	A	0.283	0.273	0.140	1.057	0.038	0.982	1.138	0.448	Genotyped
rs79715587	10	44,581,742	A	C	0.020	0.017	0.142	1.197	0.122	0.941	1.521	0.930	Imputed
rs137943088	10	44,581,922	G	A	0.012	0.010	0.241	1.205	0.159	0.882	1.647	0.605	Imputed
rs268305	10	44,581,989	G	T	0.182	0.177	0.363	1.041	0.044	0.955	1.135	0.154	Imputed
rs11238836	10	44,582,256	A	G	0.035	0.032	0.152	1.141	0.092	0.953	1.367	0.723	Imputed
rs11814490	10	44,582,268	A	G	0.149	0.142	0.213	1.061	0.048	0.967	1.165	0.054	Imputed
chr10:44582395:D	10	44,582,395	T	TAAATC	0.185	0.181	0.374	1.040	0.044	0.954	1.133	0.154	Imputed
chr10:44582558:D	10	44,582,558	G	GTGAATT	0.283	0.273	0.144	1.056	0.038	0.981	1.137	0.460	Imputed
rs268306	10	44,582,623	G	T	0.013	0.017	0.027	0.722	0.148	0.540	0.966	0.367	Imputed
rs11238837	10	44,582,763	T	C	0.100	0.095	0.367	1.052	0.057	0.942	1.175	0.479	Imputed
rs2818912	10	44,583,027	T	C	0.366	0.338	5.01E-04	1.130	0.035	1.055	1.211	0.148	Genotyped
rs67269496	10	44,583,297	A	G	0.036	0.032	0.140	1.145	0.092	0.956	1.371	0.802	Imputed
rs66536810	10	44,583,314	A	T	0.036	0.032	0.140	1.145	0.092	0.956	1.371	0.802	Imputed
rs11238838	10	44,583,333	C	A	0.269	0.255	0.041	1.081	0.038	1.003	1.166	0.316	Imputed
rs76042707	10	44,583,436	G	A	0.012	0.016	0.027	0.715	0.153	0.530	0.965	0.413	Imputed
rs11238839	10	44,583,553	A	G	0.100	0.095	0.363	1.053	0.057	0.942	1.176	0.476	Imputed
rs11238840	10	44,583,742	A	G	0.120	0.112	0.157	1.077	0.052	0.972	1.193	0.495	Imputed
rs17155150	10	44,584,367	C	T	0.149	0.142	0.202	1.063	0.048	0.968	1.167	0.056	Imputed
rs7913521	10	44,585,046	G	A	0.149	0.142	0.193	1.064	0.048	0.969	1.169	0.058	Imputed

rs17155153	10	44,585,268	T	C	0.022	0.018	0.071	1.235	0.117	0.982	1.555	0.674	Imputed
rs17155155	10	44,585,357	G	T	0.020	0.017	0.150	1.192	0.122	0.938	1.515	0.919	Imputed
rs80269158	10	44,585,642	T	C	0.020	0.017	0.150	1.192	0.122	0.938	1.515	0.919	Imputed
rs2013670	10	44,585,687	G	C	0.037	0.036	0.816	1.021	0.089	0.857	1.216	0.120	Imputed
rs72784830	10	44,585,741	A	G	0.149	0.142	0.207	1.062	0.048	0.967	1.166	0.055	Imputed
rs72784831	10	44,585,772	A	G	0.149	0.142	0.207	1.062	0.048	0.967	1.166	0.055	Imputed
rs67748318	10	44,585,815	A	G	0.169	0.160	0.084	1.081	0.045	0.990	1.182	0.073	Imputed
rs75552256	10	44,585,895	A	C	0.020	0.017	0.150	1.192	0.122	0.938	1.515	0.919	Imputed
rs72784832	10	44,585,945	G	T	0.169	0.160	0.087	1.081	0.045	0.989	1.181	0.068	Imputed
rs4948824	10	44,586,383	C	G	0.169	0.160	0.087	1.081	0.045	0.989	1.181	0.068	Imputed
rs4948826	10	44,586,527	G	A	0.169	0.160	0.085	1.081	0.045	0.989	1.182	0.072	Imputed
rs61855684	10	44,586,658	C	T	0.038	0.038	0.954	0.995	0.088	0.837	1.182	0.255	Imputed
rs146067239	10	44,586,806	A	G	0.020	0.017	0.150	1.192	0.122	0.938	1.515	0.919	Imputed
rs12263882	10	44,587,142	G	C	0.169	0.160	0.089	1.080	0.045	0.988	1.181	0.066	Imputed
rs150498042	10	44,587,193	T	C	0.037	0.036	0.816	1.021	0.089	0.857	1.216	0.120	Imputed
rs56067601	10	44,587,469	T	C	0.134	0.131	0.559	1.030	0.050	0.934	1.135	0.025	Imputed
rs75576973	10	44,587,475	A	C	0.020	0.017	0.150	1.192	0.122	0.938	1.515	0.919	Imputed
rs12242296	10	44,587,528	G	A	0.169	0.160	0.088	1.081	0.045	0.989	1.181	0.067	Imputed
rs2624691	10	44,587,857	A	T	0.464	0.501	7.47E-06	0.859	0.034	0.804	0.918	0.995	Imputed
rs149440139	10	44,588,013	G	C	0.020	0.017	0.150	1.192	0.122	0.938	1.515	0.919	Imputed
rs144775045	10	44,588,073	T	G	0.100	0.095	0.373	1.052	0.057	0.941	1.175	0.501	Imputed
rs189354749	10	44,588,086	A	G	0.020	0.017	0.150	1.192	0.122	0.938	1.515	0.919	Imputed
rs148563860	10	44,588,119	A	G	0.020	0.017	0.150	1.192	0.122	0.938	1.515	0.919	Imputed
rs4948597	10	44,588,177	C	A	0.149	0.142	0.209	1.062	0.048	0.967	1.166	0.055	Imputed
rs4417222	10	44,588,324	C	G	0.489	0.455	3.88E-05	1.150	0.034	1.076	1.229	0.804	Imputed
rs188634338	10	44,588,333	T	G	0.020	0.017	0.150	1.192	0.122	0.938	1.515	0.919	Imputed
rs4948599	10	44,588,364	C	T	0.108	0.099	0.077	1.102	0.055	0.990	1.228	0.238	Imputed
rs268296	10	44,588,427	G	T	0.013	0.017	0.030	0.728	0.147	0.546	0.971	0.354	Imputed
rs140376137	10	44,588,457	T	C	0.020	0.017	0.150	1.192	0.122	0.938	1.515	0.919	Imputed
rs141021328	10	44,589,255	G	T	0.020	0.017	0.150	1.192	0.122	0.938	1.515	0.919	Imputed
rs138317077	10	44,589,372	T	G	0.149	0.142	0.207	1.062	0.048	0.967	1.166	0.055	Imputed
rs116863659	10	44,589,423	C	T	0.169	0.160	0.089	1.080	0.045	0.988	1.181	0.066	Imputed
rs268297	10	44,589,515	A	C	0.250	0.223	8.73E-05	1.166	0.039	1.080	1.260	0.405	Imputed
rs268298	10	44,589,536	G	C	0.213	0.186	3.88E-05	1.186	0.042	1.094	1.287	0.890	Imputed
rs11517248	10	44,589,639	C	A	0.100	0.095	0.401	1.049	0.057	0.939	1.172	0.518	Imputed
rs141385860	10	44,589,843	T	C	0.530	0.495	1.60E-05	1.157	0.034	1.083	1.237	0.981	Imputed
rs2624692	10	44,589,893	G	A	0.466	0.503	7.51E-06	0.859	0.034	0.804	0.918	0.934	Imputed
rs117751587	10	44,589,910	A	C	0.100	0.095	0.401	1.049	0.057	0.939	1.172	0.518	Imputed
rs76647354	10	44,590,063	T	A	0.020	0.017	0.150	1.192	0.122	0.938	1.515	0.919	Imputed
rs141489385	10	44,590,108	A	G	0.148	0.143	0.311	1.050	0.048	0.956	1.153	0.059	Imputed

rs115604578	10	44,590,142	T	C	0.100	0.095	0.379	1.051	0.057	0.941	1.174	0.505	Imputed
rs144332235	10	44,590,428	T	C	0.020	0.017	0.159	1.188	0.122	0.935	1.510	0.909	Imputed
rs150854097	10	44,590,480	A	G	0.169	0.160	0.087	1.081	0.045	0.989	1.181	0.067	Imputed
rs4948828	10	44,590,916	T	C	0.169	0.159	0.057	1.090	0.045	0.998	1.192	0.058	Imputed
rs4948829	10	44,591,209	G	T	0.168	0.158	0.072	1.085	0.045	0.993	1.186	0.080	Imputed
rs142652436	10	44,591,215	A	T	0.146	0.139	0.172	1.068	0.048	0.972	1.174	0.072	Imputed
rs151011406	10	44,591,222	A	T	0.146	0.139	0.172	1.068	0.048	0.972	1.174	0.072	Imputed
rs4316463	10	44,591,808	T	C	0.168	0.160	0.093	1.079	0.045	0.987	1.180	0.061	Imputed
rs75445168	10	44,591,835	A	G	0.035	0.032	0.161	1.138	0.092	0.950	1.364	0.912	Imputed
rs36090148	10	44,591,928	C	T	0.149	0.142	0.207	1.062	0.048	0.967	1.166	0.055	Imputed
rs75178276	10	44,592,000	G	T	0.147	0.141	0.210	1.062	0.048	0.967	1.166	0.054	Imputed
rs12245032	10	44,592,346	T	C	0.037	0.033	0.106	1.157	0.090	0.969	1.381	0.809	Imputed
rs78928345	10	44,592,685	C	T	0.020	0.017	0.153	1.192	0.123	0.937	1.517	0.919	Imputed
rs78180150	10	44,592,686	A	G	0.020	0.017	0.153	1.192	0.123	0.937	1.517	0.919	Imputed
rs4948830	10	44,592,911	A	G	0.169	0.160	0.091	1.080	0.045	0.988	1.180	0.070	Imputed
rs4948831	10	44,593,302	A	T	0.114	0.111	0.495	1.037	0.053	0.934	1.152	0.020	Imputed
chr10:44593553:I	10	44,593,553	GA	G	0.168	0.160	0.143	1.069	0.045	0.978	1.168	0.099	Imputed
rs117402718	10	44,593,885	A	G	0.030	0.029	0.818	1.023	0.100	0.841	1.245	0.372	Imputed
rs79328003	10	44,593,889	T	C	0.020	0.018	0.220	1.160	0.121	0.915	1.472	0.971	Imputed
chr10:44594527:D	10	44,594,527	A	AAGG	0.141	0.118	1.94E-05	1.233	0.049	1.120	1.358	0.416	Imputed
chr10:44594528:D	10	44,594,528	A	AGG	0.518	0.483	3.14E-05	1.151	0.034	1.077	1.231	0.844	Imputed
rs74231463	10	44,594,687	G	A	0.020	0.017	0.150	1.192	0.122	0.938	1.515	0.919	Imputed
rs12265136	10	44,594,773	T	G	0.149	0.142	0.213	1.061	0.048	0.967	1.165	0.057	Imputed
rs2101160	10	44,595,048	A	G	0.020	0.017	0.150	1.192	0.122	0.938	1.515	0.919	Imputed
chr10:44595093:I	10	44,595,093	AC	A	0.149	0.142	0.211	1.062	0.048	0.967	1.166	0.058	Imputed
rs2086627	10	44,595,113	T	A	0.020	0.017	0.150	1.192	0.122	0.938	1.515	0.919	Imputed
rs12258788	10	44,595,196	G	A	0.149	0.142	0.211	1.062	0.048	0.967	1.166	0.058	Imputed
rs12265471	10	44,595,244	A	G	0.149	0.142	0.211	1.062	0.048	0.967	1.166	0.058	Imputed
rs58920354	10	44,595,627	A	T	0.099	0.095	0.440	1.045	0.057	0.935	1.168	0.471	Imputed
rs67594964	10	44,595,752	T	G	0.149	0.142	0.211	1.062	0.048	0.967	1.166	0.058	Imputed
rs55774075	10	44,595,964	C	T	0.169	0.160	0.087	1.081	0.045	0.989	1.181	0.071	Imputed
rs1482478	10	44,596,130	G	A	0.459	0.493	2.84E-05	0.868	0.034	0.812	0.927	0.981	Imputed
chr10:44596204:D	10	44,596,204	T	TA	0.079	0.074	0.309	1.066	0.063	0.942	1.206	0.319	Imputed
rs76775731	10	44,596,634	T	A	0.020	0.017	0.150	1.192	0.122	0.938	1.515	0.919	Imputed
rs79225358	10	44,596,884	A	C	0.037	0.036	0.833	1.019	0.090	0.855	1.215	0.145	Imputed
rs6593399	10	44,596,885	A	G	0.078	0.077	0.935	1.005	0.063	0.888	1.137	0.523	Imputed
rs79736799	10	44,596,896	A	C	0.113	0.110	0.562	1.032	0.054	0.929	1.146	0.023	Imputed
rs74565757	10	44,597,065	C	T	0.020	0.017	0.150	1.192	0.122	0.938	1.515	0.919	Imputed
rs75601522	10	44,597,118	C	G	0.020	0.017	0.150	1.192	0.122	0.938	1.515	0.919	Imputed
rs4948832	10	44,597,607	G	C	0.161	0.154	0.171	1.065	0.046	0.973	1.166	0.044	Imputed

rs4948833	10	44,597,608	G	A	0.161	0.154	0.171	1.065	0.046	0.973	1.166	0.044	Imputed
rs4948834	10	44,597,769	C	T	0.113	0.111	0.566	1.031	0.054	0.929	1.145	0.022	Imputed
rs268288	10	44,597,776	C	A	0.013	0.018	0.037	0.740	0.146	0.556	0.984	0.299	Imputed
rs4948835	10	44,597,802	A	G	0.169	0.160	0.088	1.080	0.045	0.989	1.181	0.071	Imputed
rs4948836	10	44,597,875	T	A	0.149	0.142	0.209	1.062	0.048	0.967	1.166	0.058	Imputed
rs11238850	10	44,597,898	G	A	0.079	0.078	0.962	1.003	0.063	0.887	1.135	0.495	Genotyped
rs72784836	10	44,598,022	T	G	0.011	0.010	0.655	1.076	0.164	0.780	1.484	0.857	Imputed
rs11238851	10	44,598,039	T	A	0.078	0.077	0.951	1.004	0.063	0.887	1.136	0.510	Imputed
rs10508882	10	44,598,058	G	A	0.169	0.160	0.080	1.083	0.045	0.991	1.183	0.062	Genotyped
rs268289	10	44,598,146	G	A	0.250	0.223	1.38E-04	1.161	0.039	1.075	1.254	0.482	Genotyped
rs11238852	10	44,598,207	A	G	0.040	0.035	0.066	1.174	0.087	0.989	1.392	0.646	Imputed
rs78120009	10	44,598,240	A	G	0.020	0.017	0.150	1.192	0.122	0.938	1.515	0.919	Imputed
rs11238853	10	44,598,284	C	A	0.169	0.160	0.088	1.080	0.045	0.989	1.181	0.071	Imputed
rs268290	10	44,598,285	A	C	0.013	0.017	0.032	0.732	0.147	0.548	0.976	0.317	Imputed
rs79306778	10	44,598,561	A	G	0.036	0.036	0.905	0.989	0.091	0.828	1.182	0.557	Imputed
rs2802490	10	44,599,013	A	G	0.512	0.480	6.06E-05	1.145	0.034	1.072	1.224	0.958	Genotyped
chr10:44599232:D	10	44,599,232	A	CTGGAAGTGCAGGCGGC	0.122	0.119	0.562	1.031	0.052	0.931	1.141	0.163	Imputed
rs34012822	10	44,599,391	G	A	0.149	0.142	0.209	1.062	0.048	0.967	1.166	0.058	Imputed
rs12268501	10	44,599,692	T	G	0.149	0.142	0.205	1.062	0.048	0.968	1.167	0.059	Imputed
chr10:44599772:D	10	44,599,772	C	CA	0.148	0.141	0.196	1.064	0.048	0.969	1.168	0.051	Imputed
rs34857457	10	44,599,778	T	C	0.169	0.160	0.085	1.081	0.045	0.989	1.182	0.072	Imputed
rs4948837	10	44,599,862	C	T	0.149	0.142	0.201	1.063	0.048	0.968	1.167	0.059	Imputed
rs268291	10	44,599,967	T	G	0.013	0.018	0.037	0.740	0.146	0.556	0.984	0.299	Imputed
rs12254583	10	44,600,248	T	C	0.169	0.160	0.085	1.081	0.045	0.989	1.182	0.072	Imputed
rs12254704	10	44,600,423	T	C	0.169	0.160	0.085	1.081	0.045	0.989	1.182	0.072	Imputed
rs12241133	10	44,600,717	T	G	0.168	0.160	0.097	1.078	0.045	0.987	1.179	0.076	Imputed
rs74231465	10	44,600,844	G	A	0.020	0.017	0.155	1.190	0.122	0.936	1.512	0.914	Imputed
rs12766445	10	44,600,911	G	A	0.169	0.160	0.087	1.081	0.045	0.989	1.181	0.071	Imputed
rs12766682	10	44,601,052	G	A	0.169	0.160	0.087	1.081	0.045	0.989	1.181	0.071	Imputed
rs12764460	10	44,601,054	T	G	0.169	0.160	0.087	1.081	0.045	0.989	1.181	0.071	Imputed
rs77832622	10	44,601,196	G	A	0.037	0.036	0.833	1.019	0.090	0.855	1.215	0.145	Imputed
rs12766571	10	44,601,338	T	C	0.035	0.032	0.167	1.136	0.092	0.948	1.361	0.835	Imputed
rs12256661	10	44,601,368	T	C	0.035	0.032	0.155	1.140	0.092	0.952	1.365	0.778	Imputed
rs12265921	10	44,601,565	C	T	0.169	0.160	0.087	1.081	0.045	0.989	1.181	0.071	Imputed
rs12267582	10	44,601,844	A	T	0.169	0.160	0.087	1.081	0.045	0.989	1.181	0.071	Imputed
rs35141318	10	44,602,002	T	A	0.148	0.142	0.255	1.056	0.048	0.962	1.160	0.057	Imputed
chr10:44602013:D	10	44,602,013	G	GAC	0.030	0.025	0.029	1.244	0.100	1.022	1.515	0.802	Imputed
rs58626048	10	44,602,019	T	C	0.138	0.134	0.487	1.035	0.049	0.940	1.140	0.052	Imputed
rs268292	10	44,602,043	C	G	0.013	0.018	0.036	0.738	0.146	0.555	0.983	0.302	Imputed
rs35224046	10	44,602,173	T	C	0.168	0.159	0.104	1.077	0.045	0.985	1.177	0.058	Imputed

rs34352681	10	44,602,238	C	G	0.167	0.159	0.109	1.076	0.046	0.984	1.176	0.053	Imputed
rs149476987	10	44,602,399	T	C	0.018	0.019	0.963	0.994	0.126	0.777	1.272	0.362	Imputed
rs12771977	10	44,602,405	C	G	0.143	0.140	0.454	1.037	0.048	0.943	1.140	0.093	Imputed
rs12774221	10	44,602,421	G	A	0.149	0.144	0.263	1.055	0.048	0.961	1.159	0.066	Imputed
rs4285828	10	44,602,514	C	T	0.439	0.413	9.91E-04	1.120	0.034	1.047	1.198	0.415	Imputed
rs17598084	10	44,602,630	G	A	0.136	0.128	0.112	1.082	0.050	0.982	1.193	0.277	Imputed
rs9422424	10	44,602,778	T	C	0.018	0.016	0.300	1.141	0.127	0.889	1.464	0.823	Imputed
rs185365860	10	44,602,984	A	G	0.020	0.017	0.155	1.190	0.122	0.936	1.512	0.914	Imputed
rs187374306	10	44,603,101	A	G	0.014	0.013	0.432	1.121	0.145	0.844	1.490	0.544	Imputed
rs268293	10	44,603,146	G	A	0.247	0.220	7.79E-05	1.168	0.039	1.081	1.262	0.358	Imputed
rs190892650	10	44,603,274	A	G	0.020	0.017	0.121	1.211	0.123	0.951	1.542	0.706	Imputed
rs11238856	10	44,603,498	C	T	0.163	0.156	0.149	1.069	0.046	0.977	1.170	0.074	Imputed
rs11238857	10	44,603,807	C	T	0.168	0.160	0.094	1.079	0.045	0.987	1.179	0.053	Imputed
rs11238858	10	44,603,859	G	A	0.168	0.160	0.106	1.076	0.045	0.985	1.176	0.053	Imputed
rs143830690	10	44,604,163	G	A	0.023	0.024	0.590	0.941	0.113	0.755	1.174	0.250	Imputed
rs268295	10	44,604,364	A	C	0.013	0.018	0.037	0.740	0.146	0.556	0.984	0.299	Imputed
rs75318176	10	44,604,395	A	G	0.168	0.159	0.087	1.081	0.046	0.989	1.182	0.065	Imputed
rs145133423	10	44,604,483	G	A	0.170	0.162	0.107	1.076	0.045	0.985	1.175	0.039	Imputed
rs7393222	10	44,604,565	C	T	0.023	0.019	0.089	1.215	0.114	0.971	1.520	0.855	Imputed
rs138581014	10	44,604,819	G	T	0.163	0.154	0.098	1.079	0.046	0.986	1.181	0.049	Imputed
rs181779632	10	44,604,894	C	T	0.020	0.018	0.182	1.177	0.122	0.926	1.496	0.882	Imputed
rs140307048	10	44,605,010	G	A	0.131	0.127	0.423	1.041	0.050	0.943	1.149	0.741	Imputed
rs117122834	10	44,605,028	C	T	0.023	0.024	0.993	1.001	0.112	0.803	1.247	0.327	Imputed
chr10:44605035:D	10	44,605,035	C	CT	0.035	0.036	0.845	1.018	0.092	0.851	1.218	0.497	Imputed
rs71491028	10	44,605,127	T	C	0.158	0.150	0.137	1.072	0.047	0.978	1.174	0.072	Imputed
rs149527045	10	44,605,341	C	A	0.039	0.038	0.797	1.023	0.087	0.862	1.213	0.768	Imputed
rs150731039	10	44,605,534	G	A	0.058	0.055	0.234	1.090	0.073	0.946	1.257	0.631	Imputed
rs139084442	10	44,605,557	A	G	0.039	0.034	0.073	1.173	0.089	0.985	1.396	0.646	Imputed
rs77958978	10	44,605,611	G	A	0.130	0.125	0.340	1.050	0.051	0.951	1.159	0.052	Imputed
rs140876535	10	44,605,861	C	T	0.157	0.148	0.073	1.088	0.047	0.992	1.192	0.042	Imputed
rs149829600	10	44,605,876	T	C	0.127	0.123	0.417	1.042	0.051	0.943	1.152	0.088	Imputed
rs117844549	10	44,606,018	T	C	0.020	0.017	0.124	1.206	0.122	0.950	1.531	0.759	Imputed
rs117914296	10	44,606,021	C	T	0.020	0.017	0.124	1.206	0.122	0.950	1.531	0.759	Imputed
rs145805044	10	44,606,072	A	G	0.163	0.154	0.119	1.075	0.046	0.982	1.176	0.035	Imputed
rs4948602	10	44,606,221	T	G	0.163	0.155	0.101	1.078	0.046	0.986	1.180	0.039	Imputed
rs4948838	10	44,606,365	G	C	0.154	0.148	0.233	1.058	0.047	0.965	1.160	0.036	Imputed
rs139262624	10	44,606,411	G	A	0.020	0.017	0.132	1.201	0.122	0.946	1.525	0.749	Imputed
rs4948839	10	44,606,540	T	C	0.143	0.137	0.215	1.062	0.049	0.966	1.168	0.036	Imputed
rs185240693	10	44,606,618	G	T	0.016	0.016	0.947	1.009	0.134	0.776	1.312	0.299	Imputed
rs4948603	10	44,606,643	C	G	0.159	0.150	0.069	1.088	0.047	0.993	1.192	0.096	Imputed

rs4948604	10	44,606,676	G	A	0.169	0.161	0.096	1.078	0.045	0.987	1.178	0.049	Imputed
rs7901170	10	44,606,846	T	A	0.125	0.119	0.190	1.070	0.051	0.967	1.183	0.053	Imputed
chr10:44606856:I	10	44,606,856	AT	A	0.314	0.282	8.64E-06	1.178	0.037	1.096	1.267	0.136	Imputed
chr10:44606858:I	10	44,606,858	TTC	T	0.335	0.299	2.20E-06	1.187	0.036	1.106	1.275	0.224	Imputed
rs7904643	10	44,606,956	C	T	0.169	0.160	0.086	1.081	0.045	0.989	1.181	0.049	Imputed
rs7901445	10	44,607,045	G	A	0.149	0.143	0.209	1.062	0.048	0.967	1.166	0.047	Imputed
rs150100949	10	44,607,049	A	C	0.015	0.016	0.677	0.944	0.138	0.721	1.237	0.640	Imputed
rs11238860	10	44,607,059	A	G	0.079	0.078	1.000	1.000	0.063	0.884	1.131	0.471	Imputed
rs2818914	10	44,607,154	G	T	0.502	0.472	1.44E-04	1.137	0.034	1.064	1.215	0.925	Imputed
rs11238861	10	44,607,180	T	C	0.079	0.078	1.000	1.000	0.063	0.884	1.131	0.471	Imputed
rs11238862	10	44,607,298	C	T	0.168	0.159	0.080	1.083	0.045	0.991	1.184	0.061	Imputed
rs11238863	10	44,607,426	G	A	0.161	0.152	0.085	1.083	0.046	0.989	1.186	0.061	Imputed
rs11238864	10	44,607,434	C	A	0.156	0.149	0.169	1.066	0.047	0.973	1.169	0.092	Imputed
rs11238865	10	44,607,454	C	T	0.021	0.021	0.936	0.991	0.118	0.786	1.248	0.341	Imputed
rs11238866	10	44,607,478	G	A	0.170	0.161	0.075	1.084	0.045	0.992	1.185	0.046	Imputed
rs11238867	10	44,607,525	C	T	0.169	0.160	0.082	1.082	0.045	0.990	1.182	0.050	Imputed
rs148496104	10	44,607,558	G	C	0.026	0.022	0.037	1.252	0.108	1.014	1.547	0.516	Imputed
rs187747813	10	44,607,560	C	T	0.019	0.016	0.087	1.236	0.124	0.969	1.577	0.890	Imputed
rs12777927	10	44,607,638	G	T	0.169	0.159	0.057	1.090	0.045	0.998	1.192	0.062	Imputed
rs35799836	10	44,607,690	C	T	0.176	0.168	0.098	1.077	0.045	0.987	1.175	0.051	Imputed
rs35178639	10	44,607,782	T	C	0.169	0.160	0.081	1.082	0.045	0.991	1.183	0.054	Imputed
rs12778108	10	44,607,919	C	A	0.177	0.168	0.085	1.080	0.044	0.990	1.178	0.035	Imputed
rs10899980	10	44,608,130	C	G	0.137	0.133	0.382	1.044	0.049	0.948	1.150	0.033	Imputed
rs10899981	10	44,608,139	C	T	0.147	0.140	0.196	1.064	0.048	0.969	1.169	0.049	Imputed
rs10899982	10	44,608,151	A	G	0.157	0.151	0.174	1.066	0.047	0.972	1.168	0.033	Imputed
rs10899983	10	44,608,292	C	A	0.162	0.154	0.097	1.080	0.046	0.986	1.182	0.063	Imputed
rs77811174	10	44,608,293	G	A	0.155	0.149	0.237	1.057	0.047	0.964	1.159	0.104	Imputed
rs10899985	10	44,608,326	T	G	0.169	0.160	0.084	1.081	0.045	0.990	1.182	0.049	Imputed
rs61855692	10	44,608,481	A	C	0.021	0.020	0.958	0.994	0.119	0.787	1.254	0.805	Imputed
rs12358579	10	44,608,525	T	C	0.079	0.078	0.995	1.000	0.063	0.884	1.131	0.473	Imputed
rs55877657	10	44,608,745	T	C	0.165	0.157	0.093	1.080	0.046	0.987	1.181	0.064	Imputed
rs56090994	10	44,608,746	G	A	0.159	0.153	0.197	1.062	0.046	0.969	1.163	0.056	Imputed
rs4948840	10	44,608,836	C	T	0.169	0.160	0.093	1.079	0.045	0.987	1.180	0.057	Imputed
rs150151054	10	44,609,072	T	C	0.022	0.024	0.326	0.894	0.115	0.714	1.118	0.778	Imputed
rs4948841	10	44,609,138	G	C	0.169	0.161	0.093	1.079	0.045	0.988	1.179	0.073	Imputed
rs11238868	10	44,609,231	T	C	0.079	0.078	0.989	0.999	0.063	0.883	1.130	0.476	Imputed
rs1873758	10	44,609,232	A	C	0.169	0.160	0.097	1.078	0.045	0.987	1.178	0.067	Imputed
rs75381300	10	44,609,711	A	T	0.020	0.017	0.132	1.201	0.122	0.946	1.525	0.749	Imputed
rs56386015	10	44,609,954	T	C	0.022	0.022	0.808	1.028	0.115	0.822	1.287	0.544	Imputed
rs268310	10	44,610,005	C	G	0.013	0.018	0.047	0.752	0.145	0.566	0.998	0.251	Imputed

rs11238869	10	44,610,008	T	C	0.168	0.160	0.097	1.078	0.045	0.987	1.179	0.063	Imputed
rs17155193	10	44,610,079	C	T	0.020	0.018	0.152	1.190	0.122	0.938	1.511	0.723	Genotyped
rs117878582	10	44,610,211	A	C	0.023	0.024	0.574	0.939	0.113	0.753	1.171	0.234	Imputed
rs79132306	10	44,610,423	T	C	0.020	0.017	0.132	1.201	0.122	0.946	1.525	0.749	Imputed
rs7082703	10	44,610,474	T	C	0.168	0.160	0.091	1.080	0.045	0.988	1.180	0.054	Imputed
rs6593400	10	44,610,817	G	A	0.168	0.160	0.097	1.078	0.045	0.987	1.179	0.063	Imputed
rs7909907	10	44,610,989	A	G	0.148	0.142	0.238	1.058	0.048	0.964	1.162	0.061	Imputed
rs74470917	10	44,611,016	T	C	0.020	0.017	0.132	1.201	0.122	0.946	1.525	0.749	Imputed
rs268309	10	44,611,148	T	C	0.036	0.027	4.80E-04	1.382	0.093	1.152	1.658	0.793	Genotyped
rs268308	10	44,611,209	C	A	0.036	0.027	5.83E-04	1.376	0.093	1.147	1.652	0.777	Imputed
rs76221378	10	44,611,534	A	G	0.020	0.017	0.132	1.201	0.122	0.946	1.525	0.749	Imputed
rs17155200	10	44,611,917	C	A	0.020	0.017	0.132	1.201	0.122	0.946	1.525	0.749	Imputed
rs12246545	10	44,611,995	G	A	0.168	0.160	0.097	1.078	0.045	0.987	1.179	0.063	Imputed
rs12268442	10	44,612,151	T	C	0.148	0.142	0.238	1.058	0.048	0.964	1.162	0.061	Imputed
rs141874221	10	44,612,414	G	A	0.021	0.019	0.484	1.086	0.118	0.862	1.367	0.345	Imputed
rs2220032	10	44,612,416	G	A	0.013	0.017	0.049	0.752	0.146	0.565	1.000	0.250	Imputed
rs12268604	10	44,612,433	T	C	0.168	0.160	0.097	1.078	0.045	0.987	1.179	0.063	Imputed
rs10218904	10	44,612,623	T	A	0.148	0.142	0.238	1.058	0.048	0.964	1.162	0.061	Imputed
rs1871931	10	44,612,935	G	A	0.168	0.160	0.097	1.078	0.045	0.987	1.179	0.063	Imputed
rs1871930	10	44,612,937	G	A	0.168	0.160	0.097	1.078	0.045	0.987	1.179	0.063	Imputed
rs10218996	10	44,613,091	A	G	0.168	0.160	0.097	1.078	0.045	0.987	1.179	0.063	Imputed
rs11238870	10	44,613,170	G	T	0.168	0.160	0.097	1.078	0.045	0.987	1.179	0.063	Imputed
rs11815950	10	44,613,217	A	G	0.168	0.160	0.097	1.078	0.045	0.987	1.179	0.063	Imputed
rs17155223	10	44,613,251	G	A	0.020	0.017	0.132	1.201	0.122	0.946	1.525	0.749	Imputed
rs11238871	10	44,613,591	G	C	0.078	0.078	0.975	1.002	0.063	0.886	1.134	0.500	Imputed
rs12255658	10	44,613,658	T	G	0.168	0.160	0.097	1.078	0.045	0.987	1.179	0.063	Imputed
rs2804021	10	44,613,887	G	T	0.510	0.477	5.73E-05	1.146	0.034	1.072	1.224	0.866	Imputed
rs12250586	10	44,613,905	C	A	0.168	0.160	0.097	1.078	0.045	0.987	1.179	0.063	Imputed
rs12252239	10	44,613,934	C	T	0.168	0.160	0.097	1.078	0.045	0.987	1.179	0.063	Imputed
rs12252300	10	44,614,003	C	T	0.168	0.160	0.097	1.078	0.045	0.987	1.179	0.063	Imputed
rs12243154	10	44,614,243	T	C	0.168	0.160	0.097	1.078	0.045	0.987	1.179	0.063	Imputed
chr10:44614446:I	10	44,614,446	CA	C	0.020	0.017	0.132	1.201	0.122	0.946	1.525	0.749	Imputed
rs78710849	10	44,614,794	T	C	0.037	0.036	0.818	1.021	0.090	0.856	1.217	0.130	Imputed
rs35960141	10	44,614,810	G	T	0.148	0.142	0.238	1.058	0.048	0.964	1.162	0.061	Imputed
rs174850	10	44,614,916	T	C	0.211	0.183	2.22E-05	1.194	0.042	1.100	1.295	0.757	Imputed
rs55770498	10	44,614,950	A	G	0.024	0.023	0.529	1.072	0.111	0.863	1.332	0.696	Imputed
rs77816723	10	44,615,152	C	T	0.039	0.037	0.736	1.030	0.088	0.867	1.223	0.585	Imputed
rs4948842	10	44,615,287	C	A	0.148	0.142	0.238	1.058	0.048	0.964	1.162	0.061	Imputed
rs17155241	10	44,615,356	G	A	0.020	0.017	0.132	1.201	0.122	0.946	1.525	0.749	Imputed
rs4948606	10	44,615,371	A	G	0.148	0.142	0.238	1.058	0.048	0.964	1.162	0.061	Imputed

chr10:44615461:I	10	44,615,461	GT	G	0.020	0.018	0.136	1.199	0.122	0.945	1.522	0.744	Imputed
rs17155243	10	44,615,520	G	A	0.020	0.018	0.136	1.199	0.122	0.945	1.522	0.744	Imputed
rs174849	10	44,615,859	T	C	0.099	0.083	7.65E-04	1.212	0.057	1.083	1.356	0.413	Genotyped
rs78959988	10	44,616,214	A	G	0.020	0.018	0.152	1.190	0.122	0.938	1.511	0.786	Imputed
rs77072151	10	44,616,454	G	A	0.026	0.029	0.305	0.897	0.106	0.728	1.104	0.067	Imputed
rs6593401	10	44,616,548	A	C	0.168	0.160	0.096	1.079	0.045	0.987	1.179	0.064	Imputed
rs77704045	10	44,616,814	T	A	0.020	0.018	0.136	1.199	0.122	0.945	1.522	0.744	Imputed
rs7081420	10	44,616,825	T	C	0.113	0.110	0.575	1.031	0.054	0.928	1.145	0.026	Imputed
rs75567911	10	44,616,983	A	T	0.020	0.017	0.132	1.201	0.122	0.946	1.525	0.749	Imputed
rs7080696	10	44,616,992	A	G	0.113	0.110	0.575	1.031	0.054	0.928	1.145	0.026	Imputed
rs7068995	10	44,617,119	C	T	0.166	0.157	0.085	1.082	0.046	0.989	1.183	0.071	Imputed
rs145628276	10	44,617,220	C	T	0.020	0.018	0.136	1.199	0.122	0.945	1.522	0.744	Imputed
rs17155249	10	44,617,465	A	C	0.020	0.018	0.136	1.199	0.122	0.945	1.522	0.744	Imputed
rs79929039	10	44,617,715	G	A	0.033	0.033	0.959	0.995	0.095	0.827	1.198	0.555	Imputed
rs55695076	10	44,617,931	A	G	0.113	0.110	0.571	1.031	0.054	0.928	1.145	0.027	Imputed
rs77811112	10	44,618,151	A	G	0.020	0.017	0.132	1.201	0.122	0.946	1.525	0.749	Imputed
rs17155254	10	44,618,323	A	G	0.020	0.018	0.136	1.199	0.122	0.945	1.522	0.744	Imputed
rs268307	10	44,618,365	T	C	0.242	0.214	2.62E-05	1.181	0.040	1.093	1.277	0.269	Imputed
rs80217463	10	44,618,441	A	G	0.020	0.017	0.132	1.201	0.122	0.946	1.525	0.749	Imputed
rs4948843	10	44,618,667	A	G	0.148	0.142	0.267	1.055	0.048	0.960	1.158	0.056	Imputed
rs17386524	10	44,618,762	A	G	0.012	0.016	0.031	0.724	0.152	0.538	0.974	0.360	Imputed
rs4948844	10	44,618,925	G	A	0.248	0.238	0.133	1.061	0.039	0.982	1.146	0.265	Genotyped
rs17155271	10	44,619,104	T	A	0.020	0.018	0.136	1.199	0.122	0.945	1.522	0.744	Imputed
rs10899986	10	44,619,521	G	A	0.148	0.142	0.245	1.057	0.048	0.963	1.161	0.056	Imputed
rs61855693	10	44,619,684	A	G	0.079	0.078	0.979	0.998	0.063	0.883	1.129	0.521	Imputed
rs74231467	10	44,619,747	T	C	0.020	0.017	0.132	1.201	0.122	0.946	1.525	0.749	Imputed
rs7895805	10	44,619,943	A	C	0.149	0.142	0.229	1.059	0.048	0.965	1.163	0.044	Genotyped
rs76192047	10	44,619,973	A	G	0.020	0.018	0.136	1.199	0.122	0.945	1.522	0.744	Imputed
rs118021691	10	44,620,349	C	T	0.020	0.018	0.136	1.199	0.122	0.945	1.522	0.744	Imputed
rs7895545	10	44,620,434	A	G	0.168	0.159	0.101	1.078	0.045	0.986	1.178	0.059	Imputed
rs76387630	10	44,620,451	C	A	0.020	0.018	0.136	1.199	0.122	0.945	1.522	0.744	Imputed
rs7916025	10	44,620,456	C	T	0.168	0.159	0.101	1.078	0.045	0.986	1.178	0.059	Imputed
rs74897318	10	44,621,304	C	G	0.105	0.100	0.313	1.058	0.055	0.949	1.179	0.034	Imputed
rs11238873	10	44,621,398	G	A	0.168	0.159	0.101	1.078	0.045	0.986	1.178	0.059	Imputed
rs17155274	10	44,622,014	G	A	0.020	0.018	0.136	1.199	0.122	0.945	1.522	0.744	Imputed
rs12263003	10	44,622,116	T	G	0.035	0.032	0.227	1.119	0.093	0.933	1.343	0.899	Imputed
rs12263005	10	44,622,135	T	G	0.148	0.142	0.245	1.057	0.048	0.963	1.161	0.056	Imputed
rs77457878	10	44,622,246	C	A	0.020	0.018	0.136	1.199	0.122	0.945	1.522	0.744	Imputed
chr10:44622259:I	10	44,622,259	CTG	C	0.020	0.018	0.136	1.199	0.122	0.945	1.522	0.744	Imputed
rs75925097	10	44,622,554	T	C	0.020	0.018	0.136	1.199	0.122	0.945	1.522	0.744	Imputed

rs76035718	10	44,622,639	A	C	0.020	0.017	0.132	1.201	0.122	0.946	1.525	0.749	Imputed
rs78927358	10	44,622,646	A	T	0.020	0.018	0.136	1.199	0.122	0.945	1.522	0.744	Imputed
rs77357533	10	44,622,688	A	T	0.020	0.017	0.132	1.201	0.122	0.946	1.525	0.749	Imputed
rs4948845	10	44,622,786	G	A	0.168	0.159	0.101	1.078	0.045	0.986	1.178	0.059	Imputed
rs75817481	10	44,622,915	T	C	0.020	0.018	0.136	1.199	0.122	0.945	1.522	0.744	Imputed
rs79327366	10	44,622,992	G	T	0.020	0.018	0.136	1.199	0.122	0.945	1.522	0.744	Imputed
rs72472009	10	44,623,671	A	G	0.020	0.017	0.132	1.201	0.122	0.946	1.525	0.749	Imputed
chr10:44623741:D	10	44,623,741	T	TCTAC	0.148	0.141	0.159	1.070	0.048	0.974	1.175	0.054	Imputed
rs79655606	10	44,623,751	C	T	0.020	0.017	0.155	1.190	0.122	0.936	1.512	0.914	Imputed
rs4948607	10	44,623,928	T	A	0.148	0.142	0.245	1.057	0.048	0.963	1.161	0.056	Imputed
rs79216634	10	44,623,967	T	C	0.020	0.018	0.136	1.199	0.122	0.945	1.522	0.744	Imputed
rs4948846	10	44,624,136	T	C	0.148	0.142	0.245	1.057	0.048	0.963	1.161	0.056	Imputed
rs75288314	10	44,624,146	C	A	0.020	0.018	0.136	1.199	0.122	0.945	1.522	0.744	Imputed
chr10:44624163:I	10	44,624,163	TG	T	0.020	0.017	0.132	1.201	0.122	0.946	1.525	0.749	Imputed
rs75269081	10	44,624,287	A	T	0.037	0.036	0.826	1.020	0.090	0.856	1.216	0.121	Imputed
rs77078374	10	44,624,380	T	C	0.020	0.017	0.132	1.201	0.122	0.946	1.525	0.749	Imputed
rs78285738	10	44,625,617	A	G	0.020	0.018	0.136	1.199	0.122	0.945	1.522	0.744	Imputed
rs75647319	10	44,625,810	T	C	0.020	0.017	0.132	1.201	0.122	0.946	1.525	0.749	Imputed
rs76784824	10	44,625,862	G	C	0.020	0.018	0.136	1.199	0.122	0.945	1.522	0.744	Imputed
rs78180702	10	44,626,039	A	G	0.037	0.036	0.803	1.023	0.090	0.858	1.219	0.117	Imputed
rs78571975	10	44,626,354	T	C	0.020	0.018	0.136	1.199	0.122	0.945	1.522	0.744	Imputed
rs12266428	10	44,626,440	C	T	0.168	0.159	0.101	1.078	0.045	0.986	1.178	0.059	Imputed
rs75600364	10	44,626,633	T	C	0.020	0.018	0.136	1.199	0.122	0.945	1.522	0.744	Imputed
rs76894777	10	44,626,654	G	T	0.020	0.018	0.136	1.199	0.122	0.945	1.522	0.744	Imputed
rs77316803	10	44,626,805	G	T	0.020	0.017	0.132	1.201	0.122	0.946	1.525	0.749	Imputed
rs76310352	10	44,626,866	C	T	0.020	0.018	0.136	1.199	0.122	0.945	1.522	0.744	Imputed
rs7895878	10	44,627,224	A	C	0.168	0.159	0.101	1.078	0.045	0.986	1.178	0.059	Imputed
rs17155285	10	44,627,467	T	C	0.020	0.018	0.136	1.199	0.122	0.945	1.522	0.744	Imputed
rs79974747	10	44,627,512	A	G	0.020	0.018	0.156	1.188	0.122	0.936	1.508	0.845	Imputed
rs79425099	10	44,627,532	A	G	0.020	0.018	0.136	1.199	0.122	0.945	1.522	0.744	Imputed
rs59750388	10	44,627,571	T	C	0.113	0.110	0.505	1.036	0.054	0.933	1.151	0.028	Imputed
rs169162	10	44,627,832	T	C	0.013	0.017	0.053	0.756	0.146	0.568	1.006	0.242	Imputed
chr10:44628059:I	10	44,628,059	ATCCT	A	0.020	0.017	0.132	1.201	0.122	0.946	1.525	0.749	Imputed
rs72786816	10	44,628,192	C	T	0.168	0.159	0.101	1.078	0.045	0.986	1.178	0.059	Imputed
rs72786818	10	44,628,193	A	G	0.148	0.142	0.245	1.057	0.048	0.963	1.161	0.056	Imputed
rs35874476	10	44,628,303	G	T	0.148	0.142	0.245	1.057	0.048	0.963	1.161	0.056	Imputed
rs17155293	10	44,628,475	G	A	0.020	0.018	0.136	1.199	0.122	0.945	1.522	0.744	Imputed
rs17155296	10	44,628,533	T	C	0.020	0.018	0.136	1.199	0.122	0.945	1.522	0.744	Imputed
rs17155299	10	44,628,565	A	G	0.020	0.018	0.136	1.199	0.122	0.945	1.522	0.744	Imputed
rs35024595	10	44,628,833	A	C	0.035	0.032	0.227	1.119	0.093	0.932	1.342	0.900	Imputed

rs77933503	10	44,628,895	C	T	0.020	0.017	0.132	1.201	0.122	0.946	1.525	0.749	Imputed
rs78834114	10	44,629,000	C	T	0.020	0.018	0.136	1.199	0.122	0.945	1.522	0.744	Imputed
rs17155302	10	44,629,352	G	A	0.020	0.018	0.136	1.199	0.122	0.945	1.522	0.744	Imputed
rs76111997	10	44,629,639	T	G	0.020	0.018	0.136	1.199	0.122	0.945	1.522	0.744	Imputed
rs80158492	10	44,629,826	T	C	0.020	0.017	0.132	1.201	0.122	0.946	1.525	0.749	Imputed
rs17155305	10	44,629,884	A	G	0.020	0.017	0.124	1.206	0.122	0.950	1.531	0.759	Imputed
rs75697937	10	44,631,081	T	C	0.022	0.020	0.330	1.120	0.116	0.891	1.406	0.426	Imputed
rs2047008	10	44,631,156	G	A	0.161	0.153	0.271	1.052	0.046	0.961	1.151	0.025	Genotyped
rs60914779	10	44,631,193	A	C	0.070	0.062	0.028	1.158	0.067	1.016	1.319	0.141	Imputed
rs4948850	10	44,631,258	G	T	0.023	0.020	0.122	1.194	0.114	0.954	1.494	0.926	Imputed
rs116910971	10	44,631,285	A	G	0.018	0.021	0.299	0.878	0.126	0.686	1.122	0.543	Imputed
rs4948608	10	44,631,355	T	C	0.022	0.019	0.141	1.189	0.118	0.944	1.496	0.787	Imputed
rs4948609	10	44,631,661	T	C	0.022	0.018	0.109	1.207	0.118	0.959	1.520	0.797	Imputed
rs141882920	10	44,632,244	T	C	0.013	0.012	0.714	1.057	0.150	0.788	1.418	0.001	Imputed
rs268274	10	44,632,262	C	T	0.211	0.192	5.77E-03	1.122	0.042	1.034	1.217	0.191	Genotyped
rs78575328	10	44,632,287	T	C	0.015	0.013	0.151	1.220	0.139	0.929	1.603	0.462	Imputed
rs17155313	10	44,632,370	C	G	0.021	0.018	0.143	1.188	0.118	0.943	1.498	0.787	Imputed
rs17155319	10	44,632,504	A	G	0.021	0.018	0.143	1.188	0.118	0.943	1.498	0.787	Imputed
rs147674444	10	44,632,772	A	T	0.021	0.018	0.143	1.188	0.118	0.943	1.498	0.787	Imputed
rs12777551	10	44,632,828	A	G	0.027	0.027	0.902	1.013	0.104	0.827	1.241	0.628	Imputed
rs117318616	10	44,632,848	A	G	0.042	0.042	0.872	1.014	0.084	0.860	1.196	0.430	Imputed
rs140552732	10	44,632,957	T	C	0.041	0.034	0.016	1.230	0.086	1.039	1.457	0.093	Imputed
rs137933100	10	44,633,242	T	C	0.021	0.018	0.143	1.188	0.118	0.943	1.498	0.787	Imputed
rs140942374	10	44,633,404	T	A	0.021	0.018	0.143	1.188	0.118	0.943	1.498	0.787	Imputed
rs144593438	10	44,633,503	A	G	0.010	0.012	0.607	0.918	0.167	0.661	1.274	0.910	Imputed
rs7901877	10	44,633,766	C	G	0.211	0.192	7.58E-03	1.117	0.042	1.030	1.213	0.157	Imputed
rs79076780	10	44,633,804	A	G	0.021	0.018	0.143	1.188	0.118	0.943	1.498	0.787	Imputed
rs75564349	10	44,633,854	T	C	0.021	0.018	0.143	1.188	0.118	0.943	1.498	0.787	Imputed
rs4948851	10	44,634,073	T	A	0.518	0.483	1.57E-05	1.157	0.034	1.083	1.237	0.637	Imputed
rs4948610	10	44,634,099	G	A	0.021	0.018	0.143	1.188	0.118	0.943	1.498	0.787	Imputed
rs139953701	10	44,634,319	G	C	0.037	0.035	0.509	1.061	0.090	0.890	1.265	0.766	Imputed
rs4948611	10	44,634,397	A	G	0.407	0.380	1.12E-03	1.119	0.035	1.046	1.198	0.258	Imputed
rs6593402	10	44,634,459	C	T	0.100	0.099	0.782	1.016	0.056	0.910	1.134	0.348	Imputed
rs149839423	10	44,634,670	G	A	0.021	0.018	0.143	1.188	0.118	0.943	1.498	0.787	Imputed
rs142916567	10	44,634,809	C	G	0.022	0.019	0.179	1.170	0.117	0.930	1.473	0.722	Imputed
rs118027696	10	44,635,256	A	C	0.021	0.018	0.143	1.188	0.118	0.943	1.498	0.787	Imputed
rs138078514	10	44,635,664	C	T	0.091	0.087	0.361	1.055	0.059	0.940	1.184	0.062	Imputed
rs150056367	10	44,635,853	A	G	0.027	0.027	0.902	1.013	0.104	0.827	1.241	0.628	Imputed
rs138842148	10	44,635,901	C	T	0.023	0.021	0.352	1.112	0.114	0.889	1.392	0.685	Imputed
rs150067838	10	44,636,919	C	T	0.021	0.018	0.158	1.182	0.119	0.937	1.492	0.862	Imputed

rs146594357	10	44,637,209	A	G	0.014	0.017	0.131	0.807	0.142	0.611	1.067	0.194	Imputed
rs11238875	10	44,637,338	G	A	0.525	0.494	1.54E-04	1.137	0.034	1.064	1.215	0.163	Imputed
rs143845670	10	44,637,348	A	G	0.014	0.017	0.131	0.807	0.142	0.611	1.067	0.194	Imputed
rs11238876	10	44,637,365	T	C	0.019	0.018	0.770	1.037	0.124	0.813	1.323	0.956	Imputed
rs187934805	10	44,637,435	C	A	0.024	0.023	0.488	1.079	0.110	0.870	1.340	0.718	Imputed
rs148859283	10	44,637,437	C	A	0.014	0.017	0.131	0.807	0.142	0.611	1.067	0.194	Imputed
rs143433810	10	44,637,736	C	T	0.021	0.018	0.143	1.188	0.118	0.943	1.498	0.787	Imputed
rs10899988	10	44,637,810	T	C	0.140	0.131	0.079	1.090	0.049	0.990	1.199	0.867	Imputed
rs184438337	10	44,638,136	A	G	0.022	0.020	0.400	1.102	0.116	0.879	1.382	0.665	Imputed
rs138779660	10	44,638,240	T	C	0.210	0.192	8.13E-03	1.116	0.042	1.029	1.211	0.161	Imputed
rs185068013	10	44,638,271	T	A	0.015	0.014	0.728	1.050	0.141	0.797	1.385	0.436	Imputed
chr10:44638286:D	10	44,638,286	A	AT	0.142	0.133	0.097	1.084	0.049	0.986	1.193	0.675	Imputed
rs1979470	10	44,638,417	A	G	0.061	0.064	0.423	0.945	0.071	0.823	1.085	0.832	Imputed
rs61855695	10	44,638,496	T	G	0.032	0.033	0.473	0.933	0.096	0.773	1.127	0.425	Imputed
chr10:44638659:D	10	44,638,659	G	GA	0.022	0.019	0.141	1.189	0.118	0.944	1.497	0.908	Imputed
rs766927	10	44,639,092	T	C	0.466	0.499	6.86E-05	0.874	0.034	0.818	0.934	0.401	Imputed
rs2802476	10	44,640,030	C	G	0.466	0.499	6.86E-05	0.874	0.034	0.818	0.934	0.401	Imputed
rs10899989	10	44,640,048	T	C	0.210	0.192	8.02E-03	1.117	0.042	1.029	1.212	0.160	Imputed
rs10793523	10	44,640,224	C	G	0.140	0.131	0.073	1.092	0.049	0.992	1.201	0.852	Imputed
rs79460943	10	44,640,760	G	T	0.021	0.018	0.143	1.188	0.118	0.943	1.498	0.787	Imputed
rs17155323	10	44,640,792	G	A	0.021	0.018	0.143	1.188	0.118	0.943	1.498	0.787	Imputed
rs10899990	10	44,640,797	G	A	0.140	0.132	0.092	1.086	0.049	0.987	1.195	0.876	Genotyped
rs113336822	10	44,640,925	T	A	0.050	0.046	0.292	1.085	0.078	0.932	1.264	0.043	Imputed
rs17158901	10	44,641,015	G	C	0.021	0.018	0.143	1.188	0.118	0.943	1.498	0.787	Imputed
rs17158902	10	44,641,066	A	G	0.021	0.018	0.143	1.188	0.118	0.943	1.498	0.787	Imputed
rs145817508	10	44,641,345	T	C	0.021	0.018	0.143	1.188	0.118	0.943	1.498	0.787	Imputed
rs35526908	10	44,641,409	T	C	0.030	0.029	0.694	1.039	0.098	0.857	1.261	0.374	Imputed
rs11598966	10	44,641,416	T	C	0.210	0.192	8.13E-03	1.116	0.042	1.029	1.211	0.161	Imputed
rs148040454	10	44,641,861	A	C	0.037	0.035	0.509	1.061	0.090	0.890	1.265	0.766	Imputed
rs4948852	10	44,641,865	C	A	0.022	0.019	0.098	1.213	0.117	0.965	1.526	0.725	Imputed
rs4948853	10	44,642,021	T	C	0.021	0.018	0.136	1.192	0.118	0.946	1.503	0.835	Imputed
rs11238878	10	44,642,157	A	G	0.112	0.103	0.074	1.101	0.054	0.991	1.224	0.908	Imputed
rs184312206	10	44,642,224	C	T	0.112	0.103	0.074	1.101	0.054	0.991	1.224	0.908	Imputed
chr10:44642324:I	10	44,642,324	GT	G	0.114	0.110	0.337	1.053	0.053	0.948	1.169	0.104	Imputed
rs113920162	10	44,642,556	A	C	0.112	0.109	0.525	1.035	0.054	0.931	1.150	0.051	Imputed
rs4411243	10	44,642,567	T	C	0.012	0.016	0.039	0.733	0.152	0.545	0.987	0.394	Imputed
rs142539486	10	44,643,198	T	G	0.117	0.103	8.35E-03	1.150	0.053	1.036	1.276	0.872	Imputed
rs150535609	10	44,643,216	G	A	0.023	0.019	0.059	1.239	0.114	0.992	1.547	0.723	Imputed
rs145306112	10	44,644,211	T	A	0.050	0.054	0.298	0.923	0.077	0.793	1.074	0.902	Imputed
rs144980272	10	44,644,237	T	C	0.014	0.009	6.98E-03	1.487	0.148	1.113	1.987	0.971	Imputed

rs191599060	10	44,644,692	T	C	0.035	0.026	7.17E-04	1.374	0.094	1.143	1.653	0.509	Imputed
rs141311557	10	44,644,756	G	T	0.021	0.018	0.136	1.193	0.118	0.946	1.503	0.834	Imputed
rs117277200	10	44,644,770	A	G	0.029	0.025	0.124	1.169	0.102	0.958	1.428	0.366	Imputed
rs144958065	10	44,644,970	T	C	0.140	0.131	0.062	1.096	0.049	0.996	1.206	0.865	Imputed
rs142204626	10	44,645,068	A	G	0.020	0.021	0.423	0.907	0.122	0.715	1.151	0.962	Imputed
rs138353310	10	44,645,259	C	T	0.021	0.018	0.136	1.193	0.118	0.946	1.503	0.834	Imputed
rs140378847	10	44,645,449	T	C	0.021	0.018	0.136	1.193	0.118	0.946	1.503	0.834	Imputed
rs139467514	10	44,645,682	A	T	0.021	0.018	0.136	1.193	0.118	0.946	1.503	0.834	Imputed
rs182356612	10	44,645,841	T	G	0.013	0.010	0.076	1.309	0.153	0.970	1.765	0.562	Imputed
rs145237294	10	44,646,123	T	C	0.030	0.029	0.694	1.039	0.098	0.857	1.261	0.374	Imputed
rs138787101	10	44,646,547	A	G	0.021	0.018	0.136	1.193	0.118	0.946	1.503	0.834	Imputed
rs117634861	10	44,646,903	A	G	0.026	0.022	0.053	1.230	0.107	0.997	1.517	0.920	Imputed
rs4631834	10	44,647,120	A	C	0.021	0.018	0.136	1.192	0.118	0.946	1.503	0.833	Imputed
rs10899991	10	44,647,160	T	C	0.219	0.198	3.20E-03	1.129	0.041	1.041	1.223	0.130	Imputed
rs71491034	10	44,647,570	C	T	0.030	0.030	0.743	1.033	0.098	0.852	1.253	0.366	Imputed
rs2862835	10	44,647,574	A	G	0.210	0.192	8.36E-03	1.116	0.042	1.028	1.211	0.162	Imputed
rs72786825	10	44,647,614	T	C	0.025	0.024	0.442	1.087	0.108	0.879	1.344	0.760	Imputed
chr10:44648252:D	10	44,648,252	C	CA	0.102	0.097	0.351	1.054	0.056	0.944	1.177	0.023	Imputed
rs192424365	10	44,648,258	T	G	0.102	0.097	0.351	1.054	0.056	0.944	1.177	0.025	Imputed
rs7476352	10	44,648,340	A	T	0.021	0.018	0.136	1.192	0.118	0.946	1.503	0.833	Imputed
rs117369924	10	44,648,343	T	A	0.030	0.029	0.694	1.039	0.098	0.857	1.261	0.374	Imputed
rs10899992	10	44,648,515	T	C	0.210	0.192	7.77E-03	1.117	0.042	1.030	1.212	0.169	Imputed
rs10793524	10	44,648,621	T	C	0.140	0.131	0.062	1.096	0.049	0.996	1.206	0.865	Imputed
rs117026329	10	44,648,996	C	G	0.012	0.016	0.039	0.733	0.152	0.545	0.987	0.394	Imputed
rs2035416	10	44,649,690	C	T	0.210	0.192	7.88E-03	1.117	0.042	1.029	1.212	0.170	Imputed
rs17155325	10	44,650,008	C	A	0.021	0.018	0.136	1.192	0.118	0.946	1.503	0.833	Imputed
rs75322804	10	44,650,061	C	T	0.021	0.018	0.136	1.193	0.118	0.946	1.503	0.834	Imputed
rs139198274	10	44,650,183	C	T	0.010	0.010	0.655	0.926	0.173	0.660	1.298	0.484	Imputed
rs17155326	10	44,650,508	G	T	0.021	0.018	0.136	1.193	0.118	0.946	1.503	0.834	Imputed
rs74941207	10	44,650,532	C	T	0.021	0.018	0.136	1.193	0.118	0.946	1.503	0.834	Imputed
rs17155329	10	44,650,861	A	G	0.021	0.018	0.136	1.193	0.118	0.946	1.503	0.834	Imputed
rs75844521	10	44,651,338	G	A	0.091	0.087	0.358	1.055	0.059	0.941	1.184	0.065	Imputed
rs3900567	10	44,651,421	T	C	0.210	0.192	8.24E-03	1.116	0.042	1.029	1.211	0.168	Imputed
rs4082374	10	44,651,619	A	G	0.021	0.018	0.136	1.193	0.118	0.946	1.503	0.834	Imputed
rs34941677	10	44,652,166	A	G	0.030	0.029	0.694	1.039	0.098	0.857	1.261	0.374	Imputed
rs1994107	10	44,652,274	A	G	0.023	0.019	0.075	1.227	0.115	0.979	1.538	0.750	Genotyped
rs35155049	10	44,653,651	C	T	0.030	0.029	0.686	1.041	0.098	0.858	1.262	0.371	Imputed
rs142685514	10	44,654,868	A	G	0.024	0.025	0.698	0.959	0.109	0.774	1.188	0.221	Imputed
chr10:44655094:D	10	44,655,094	A	AAGC	0.021	0.018	0.139	1.191	0.118	0.944	1.501	0.840	Imputed
rs2154401	10	44,655,275	T	C	0.466	0.499	6.89E-05	0.874	0.034	0.818	0.934	0.407	Imputed

rs7918766	10	44,655,592	T	C	0.030	0.029	0.735	1.034	0.098	0.852	1.254	0.363	Imputed
rs1871928	10	44,656,034	G	A	0.021	0.018	0.136	1.193	0.118	0.946	1.503	0.834	Imputed
rs117928666	10	44,656,147	G	C	0.029	0.029	0.993	1.001	0.101	0.822	1.219	0.365	Imputed
rs10899993	10	44,656,614	G	T	0.232	0.211	2.32E-03	1.130	0.040	1.044	1.223	0.140	Imputed
rs10899994	10	44,656,695	G	A	0.211	0.192	6.98E-03	1.119	0.042	1.031	1.214	0.159	Imputed
rs118044339	10	44,657,116	A	G	0.022	0.019	0.180	1.168	0.116	0.931	1.465	0.873	Imputed
rs1118004	10	44,657,556	T	C	0.021	0.018	0.128	1.197	0.118	0.949	1.509	0.883	Imputed
rs10899995	10	44,658,164	A	G	0.511	0.476	1.38E-05	1.159	0.034	1.084	1.238	0.594	Imputed
rs61855737	10	44,659,076	T	C	0.231	0.210	2.90E-03	1.127	0.040	1.042	1.220	0.163	Imputed
rs79816830	10	44,659,181	A	T	0.021	0.018	0.194	1.167	0.119	0.924	1.475	0.775	Imputed
chr10:44659960:D	10	44,659,960	C	CCTT	0.021	0.018	0.194	1.167	0.119	0.924	1.475	0.775	Imputed
rs2804042	10	44,660,051	A	G	0.466	0.498	9.31E-05	0.876	0.034	0.820	0.936	0.413	Imputed
rs55727850	10	44,660,151	T	C	0.023	0.020	0.149	1.178	0.114	0.943	1.472	0.573	Imputed
rs75149305	10	44,660,286	A	T	0.021	0.018	0.194	1.167	0.119	0.924	1.475	0.775	Imputed
rs11238884	10	44,660,408	C	T	0.232	0.212	2.89E-03	1.127	0.040	1.042	1.220	0.174	Imputed
rs74453874	10	44,661,230	T	A	0.021	0.018	0.194	1.167	0.119	0.924	1.475	0.775	Imputed
rs150099413	10	44,662,376	A	G	0.023	0.024	0.730	0.962	0.112	0.772	1.199	0.173	Imputed
rs149329101	10	44,662,825	G	C	0.013	0.012	0.667	1.069	0.153	0.792	1.443	0.000	Imputed
chr10:44662877:D	10	44,662,877	G	GTC	0.430	0.396	1.98E-05	1.157	0.034	1.082	1.237	0.058	Imputed
rs746141	10	44,663,102	C	G	0.014	0.017	0.109	0.795	0.144	0.600	1.054	0.233	Imputed
chr10:44663157:I	10	44,663,157	CA	C	0.021	0.018	0.235	1.153	0.120	0.911	1.458	0.812	Imputed
chr10:44663869:I	10	44,663,869	TAAATAAGG	T	0.021	0.019	0.328	1.123	0.119	0.890	1.418	0.891	Imputed
rs900329	10	44,663,874	C	T	0.014	0.017	0.109	0.795	0.144	0.600	1.054	0.233	Imputed
rs4948612	10	44,664,025	A	G	0.021	0.018	0.235	1.153	0.120	0.911	1.458	0.812	Imputed
rs11238886	10	44,664,290	C	T	0.135	0.126	0.081	1.091	0.050	0.989	1.202	0.711	Imputed
rs4948856	10	44,664,489	T	C	0.021	0.018	0.235	1.153	0.120	0.911	1.458	0.812	Imputed
rs2802487	10	44,664,524	A	G	0.466	0.497	1.11E-04	0.877	0.034	0.821	0.938	0.437	Genotyped
rs72786836	10	44,664,557	A	G	0.024	0.023	0.561	1.066	0.111	0.858	1.325	0.607	Imputed
rs74398000	10	44,664,949	A	C	0.109	0.102	0.121	1.088	0.055	0.978	1.211	0.917	Imputed
rs11238887	10	44,664,965	A	G	0.135	0.126	0.082	1.090	0.050	0.989	1.202	0.713	Imputed
rs2170555	10	44,665,753	C	G	0.021	0.018	0.235	1.153	0.120	0.911	1.458	0.812	Imputed
rs12359898	10	44,666,146	T	C	0.210	0.192	8.72E-03	1.115	0.042	1.028	1.210	0.197	Imputed
rs2804040	10	44,666,579	A	C	0.374	0.346	3.64E-04	1.133	0.035	1.058	1.214	0.003	Imputed
rs17155364	10	44,666,968	A	G	0.016	0.016	0.669	1.060	0.135	0.813	1.382	0.976	Imputed
rs11238888	10	44,667,265	A	T	0.135	0.121	0.014	1.130	0.050	1.024	1.245	0.883	Imputed
rs35374150	10	44,667,396	G	A	0.029	0.028	0.635	1.049	0.100	0.862	1.276	0.306	Imputed
rs7092358	10	44,667,426	C	G	0.250	0.231	6.56E-03	1.112	0.039	1.030	1.201	0.234	Imputed
rs2154399	10	44,667,721	G	A	0.015	0.015	0.825	1.031	0.140	0.785	1.356	0.626	Imputed
chr10:44667855:I	10	44,667,855	TG	T	0.014	0.018	0.066	0.772	0.141	0.586	1.017	0.162	Imputed
rs12775466	10	44,667,926	A	G	0.022	0.021	0.358	1.112	0.115	0.887	1.393	0.855	Imputed

rs2802486	10	44,667,958	C	T	0.390	0.382	0.369	1.032	0.035	0.964	1.104	0.998	Imputed
rs11238889	10	44,668,724	A	G	0.108	0.098	0.071	1.104	0.055	0.991	1.229	0.515	Imputed
rs11238890	10	44,669,226	T	A	0.108	0.098	0.071	1.104	0.055	0.991	1.229	0.515	Imputed
rs12240324	10	44,669,337	G	A	0.014	0.015	0.350	0.874	0.145	0.659	1.160	0.272	Genotyped
rs17155389	10	44,670,020	A	G	0.010	0.013	0.104	0.762	0.168	0.548	1.059	0.683	Imputed
rs74138235	10	44,670,058	G	A	0.032	0.031	0.851	1.018	0.097	0.842	1.231	0.353	Imputed
rs12264159	10	44,670,187	T	C	0.010	0.013	0.104	0.762	0.168	0.548	1.059	0.683	Imputed
rs12264354	10	44,670,500	T	C	0.013	0.015	0.291	0.855	0.148	0.640	1.144	0.435	Imputed
rs11238891	10	44,670,992	T	G	0.108	0.098	0.059	1.109	0.055	0.996	1.235	0.532	Imputed
rs72786842	10	44,671,028	A	G	0.028	0.023	0.027	1.259	0.104	1.026	1.545	0.284	Imputed
rs11238892	10	44,671,194	T	C	0.108	0.098	0.059	1.109	0.055	0.996	1.235	0.532	Imputed
rs74333644	10	44,671,544	G	C	0.064	0.056	0.041	1.153	0.070	1.006	1.322	0.956	Imputed
rs3979458	10	44,671,713	A	G	0.121	0.124	0.565	0.971	0.052	0.877	1.074	0.680	Imputed
rs2624676	10	44,671,715	A	C	0.236	0.227	0.218	1.050	0.040	0.971	1.136	0.545	Imputed
rs139846062	10	44,671,738	C	T	0.009	0.010	0.662	0.927	0.175	0.658	1.304	0.819	Imputed
rs11238894	10	44,671,851	C	T	0.108	0.098	0.059	1.109	0.055	0.996	1.235	0.532	Imputed
rs3892077	10	44,672,151	C	T	0.306	0.290	0.062	1.071	0.037	0.997	1.151	0.380	Imputed
rs142530900	10	44,672,260	A	G	0.024	0.024	0.697	0.958	0.111	0.772	1.190	0.113	Imputed
rs1704228	10	44,673,050	T	C	0.108	0.097	0.048	1.115	0.055	1.001	1.241	0.578	Imputed
rs1680635	10	44,673,148	A	G	0.305	0.290	0.064	1.071	0.037	0.996	1.151	0.372	Genotyped
rs2802484	10	44,673,214	A	C	0.323	0.310	0.106	1.060	0.036	0.988	1.138	0.457	Imputed
rs78431960	10	44,673,314	T	G	0.064	0.056	0.041	1.153	0.070	1.006	1.322	0.953	Imputed
rs7919208	10	44,673,557	A	G	0.023	0.028	0.074	0.818	0.113	0.656	1.020	0.878	Imputed
rs2804038	10	44,673,642	G	A	0.324	0.310	0.099	1.062	0.036	0.989	1.140	0.455	Imputed
rs7099567	10	44,673,828	C	G	0.026	0.024	0.569	1.063	0.107	0.862	1.310	0.096	Imputed
rs17155437	10	44,675,056	G	A	0.196	0.193	0.625	1.021	0.043	0.939	1.110	0.122	Genotyped
chr10:44675236:I	10	44,675,236	TA	T	0.301	0.288	0.135	1.057	0.037	0.983	1.136	0.513	Imputed
rs115885922	10	44,675,425	G	T	0.064	0.056	0.039	1.155	0.070	1.007	1.324	0.930	Imputed
rs7896736	10	44,675,510	A	G	0.196	0.193	0.602	1.022	0.043	0.941	1.112	0.116	Imputed
rs112286887	10	44,675,700	A	G	0.016	0.011	6.54E-03	1.461	0.140	1.110	1.923	0.874	Imputed
rs2802482	10	44,675,851	G	A	0.127	0.117	0.098	1.088	0.051	0.985	1.203	0.434	Imputed
rs1680636	10	44,675,941	A	G	0.109	0.097	0.034	1.123	0.055	1.008	1.250	0.511	Imputed
rs76053741	10	44,676,024	A	G	0.016	0.011	6.95E-03	1.457	0.140	1.107	1.917	0.867	Imputed
rs74138238	10	44,676,292	A	G	0.012	0.015	0.173	0.812	0.153	0.602	1.096	0.960	Imputed
rs12247410	10	44,676,465	T	C	0.012	0.015	0.173	0.812	0.153	0.602	1.096	0.960	Imputed
rs11594667	10	44,676,695	A	G	0.109	0.097	0.034	1.123	0.055	1.009	1.250	0.506	Imputed
rs1704223	10	44,677,014	T	C	0.109	0.097	0.034	1.123	0.055	1.008	1.250	0.511	Imputed
rs17155453	10	44,677,015	A	G	0.012	0.015	0.173	0.812	0.153	0.602	1.096	0.960	Imputed
rs2624681	10	44,677,301	C	G	0.127	0.117	0.101	1.087	0.051	0.984	1.202	0.439	Imputed
rs2664860	10	44,677,870	G	A	0.124	0.114	0.093	1.090	0.052	0.986	1.206	0.465	Imputed

rs56095445	10	44,677,927	A	G	0.196	0.193	0.642	1.020	0.043	0.938	1.109	0.152	Imputed
rs1615053	10	44,677,935	C	T	0.109	0.097	0.034	1.123	0.055	1.009	1.250	0.508	Imputed
rs149399290	10	44,677,967	T	C	0.014	0.018	0.068	0.773	0.141	0.586	1.020	0.987	Imputed
rs17155463	10	44,678,218	T	A	0.012	0.015	0.173	0.812	0.153	0.602	1.096	0.960	Imputed
rs2624682	10	44,678,240	T	C	0.127	0.117	0.101	1.087	0.051	0.984	1.202	0.439	Imputed
rs17155474	10	44,678,262	C	T	0.012	0.015	0.173	0.812	0.153	0.602	1.096	0.960	Imputed
rs1704226	10	44,678,387	A	C	0.109	0.097	0.036	1.122	0.055	1.008	1.249	0.515	Imputed
rs78786147	10	44,678,412	T	G	0.014	0.011	0.055	1.317	0.144	0.993	1.747	0.253	Imputed
rs77411558	10	44,678,413	T	G	0.014	0.011	0.055	1.317	0.144	0.993	1.747	0.253	Imputed
rs17155478	10	44,678,454	T	C	0.013	0.015	0.249	0.842	0.149	0.629	1.128	0.760	Genotyped
rs74138240	10	44,678,476	T	C	0.010	0.013	0.150	0.790	0.164	0.573	1.090	0.597	Imputed
rs1680637	10	44,678,682	T	C	0.018	0.020	0.468	0.913	0.126	0.713	1.169	0.701	Imputed
rs1704227	10	44,678,775	C	T	0.018	0.019	0.447	0.908	0.127	0.708	1.165	0.901	Genotyped
rs12249837	10	44,678,898	G	A	0.012	0.015	0.168	0.811	0.153	0.601	1.093	0.966	Imputed
rs11238898	10	44,679,016	A	T	0.109	0.097	0.036	1.122	0.055	1.008	1.249	0.515	Imputed
rs2624683	10	44,679,140	G	A	0.127	0.117	0.105	1.086	0.051	0.983	1.201	0.446	Imputed
rs7075227	10	44,679,319	A	G	0.010	0.013	0.138	0.784	0.164	0.569	1.082	0.613	Imputed
rs61855739	10	44,679,603	A	G	0.109	0.097	0.035	1.122	0.055	1.008	1.249	0.513	Imputed
rs1545844	10	44,679,763	A	C	0.197	0.194	0.577	1.024	0.043	0.942	1.113	0.144	Imputed
rs1545845	10	44,679,802	C	A	0.127	0.117	0.097	1.088	0.051	0.985	1.203	0.457	Imputed
rs61855740	10	44,679,810	G	A	0.109	0.097	0.035	1.122	0.055	1.008	1.249	0.513	Imputed
rs151220070	10	44,680,094	G	C	0.010	0.012	0.175	0.796	0.169	0.572	1.108	0.773	Imputed
rs145014402	10	44,680,298	A	G	0.016	0.011	6.95E-03	1.457	0.140	1.107	1.917	0.867	Imputed
rs2804036	10	44,680,405	C	T	0.127	0.117	0.092	1.090	0.051	0.986	1.204	0.473	Genotyped
rs7098818	10	44,680,487	G	A	0.198	0.195	0.627	1.021	0.043	0.939	1.110	0.159	Imputed
rs145692892	10	44,680,518	A	T	0.014	0.011	0.075	1.296	0.146	0.973	1.725	0.843	Imputed
rs2246891	10	44,680,591	G	A	0.127	0.117	0.096	1.089	0.051	0.985	1.203	0.455	Imputed
rs11595159	10	44,680,608	C	T	0.109	0.097	0.036	1.122	0.055	1.008	1.249	0.515	Imputed
chr10:44680902:D	10	44,680,902	T	TAAAGA	0.012	0.015	0.196	0.821	0.153	0.608	1.108	0.928	Imputed
rs11238901	10	44,681,012	A	C	0.109	0.097	0.036	1.122	0.055	1.008	1.249	0.515	Imputed
rs11238902	10	44,681,091	T	C	0.109	0.097	0.036	1.122	0.055	1.008	1.249	0.515	Imputed
chr10:44681231:I	10	44,681,231	GCC	G	0.127	0.116	0.077	1.095	0.051	0.990	1.210	0.453	Imputed
rs17155512	10	44,681,417	A	C	0.012	0.015	0.186	0.817	0.153	0.606	1.103	0.941	Imputed
rs117066820	10	44,681,559	C	T	0.012	0.010	0.119	1.271	0.155	0.938	1.722	0.381	Imputed
rs60685600	10	44,681,591	G	A	0.181	0.165	0.014	1.115	0.044	1.022	1.215	0.632	Imputed
rs35339178	10	44,681,592	C	T	0.180	0.164	0.012	1.117	0.044	1.024	1.218	0.868	Imputed
rs61855743	10	44,681,729	G	A	0.109	0.097	0.034	1.123	0.055	1.008	1.250	0.511	Imputed
rs1509931	10	44,682,080	T	C	0.127	0.116	0.076	1.095	0.051	0.990	1.210	0.452	Imputed
rs3912305	10	44,682,214	T	C	0.066	0.058	0.037	1.154	0.069	1.009	1.320	0.981	Imputed
rs1509930	10	44,682,222	A	G	0.308	0.298	0.275	1.041	0.037	0.969	1.118	0.456	Imputed

rs117476616	10	44,682,568	T	A	0.014	0.013	0.641	1.072	0.147	0.803	1.431	0.002	Imputed
rs1704214	10	44,682,729	G	A	0.127	0.116	0.074	1.096	0.051	0.991	1.211	0.447	Imputed
chr10:44682776:I	10	44,682,776	AC	A	0.127	0.116	0.074	1.096	0.051	0.991	1.211	0.447	Imputed
rs1509929	10	44,682,829	C	A	0.190	0.172	6.00E-03	1.126	0.043	1.035	1.226	0.534	Imputed
rs1482472	10	44,682,973	C	T	0.388	0.366	0.011	1.093	0.035	1.021	1.170	0.441	Imputed
rs117988613	10	44,683,277	A	G	0.064	0.056	0.041	1.153	0.070	1.006	1.322	0.955	Imputed
rs4948857	10	44,683,281	T	C	0.127	0.116	0.074	1.096	0.051	0.991	1.211	0.447	Imputed
rs113230273	10	44,683,598	G	A	0.016	0.011	6.95E-03	1.457	0.140	1.107	1.917	0.867	Imputed
rs10793525	10	44,683,833	A	C	0.190	0.172	5.92E-03	1.127	0.043	1.035	1.226	0.580	Imputed
rs10899997	10	44,683,841	G	T	0.190	0.172	6.04E-03	1.126	0.043	1.035	1.226	0.572	Imputed
rs74138248	10	44,684,362	C	T	0.018	0.019	0.597	0.935	0.127	0.728	1.200	0.796	Imputed
rs11819122	10	44,684,676	C	G	0.012	0.015	0.201	0.823	0.153	0.610	1.110	0.975	Imputed
rs11238904	10	44,684,707	T	C	0.126	0.116	0.080	1.094	0.051	0.989	1.209	0.518	Imputed
rs7069262	10	44,684,766	C	G	0.182	0.182	0.907	0.995	0.044	0.913	1.084	0.132	Imputed
rs61855758	10	44,684,854	C	T	0.126	0.116	0.080	1.094	0.051	0.989	1.209	0.518	Imputed
rs11817838	10	44,684,912	G	A	0.126	0.116	0.080	1.094	0.051	0.989	1.209	0.518	Imputed
rs11238905	10	44,685,136	A	T	0.126	0.116	0.080	1.094	0.051	0.989	1.209	0.518	Imputed
rs11815837	10	44,685,145	T	C	0.012	0.015	0.201	0.823	0.153	0.610	1.110	0.975	Imputed
rs113304033	10	44,685,386	A	C	0.022	0.024	0.467	0.920	0.115	0.734	1.153	0.646	Imputed
rs2802479	10	44,685,402	C	T	0.029	0.030	0.533	0.940	0.101	0.772	1.144	0.027	Imputed
rs55823236	10	44,685,619	G	A	0.012	0.015	0.191	0.819	0.153	0.607	1.105	0.987	Imputed
rs11595946	10	44,685,695	T	C	0.108	0.097	0.044	1.117	0.055	1.003	1.244	0.493	Imputed
rs11598296	10	44,685,776	G	A	0.126	0.116	0.081	1.093	0.051	0.989	1.209	0.519	Imputed
rs11598314	10	44,685,795	G	A	0.126	0.116	0.081	1.093	0.051	0.989	1.209	0.519	Imputed
rs117701844	10	44,685,836	T	C	0.064	0.056	0.041	1.153	0.070	1.006	1.322	0.955	Imputed
rs11238906	10	44,685,925	C	T	0.134	0.124	0.107	1.084	0.050	0.983	1.195	0.734	Imputed
rs12415866	10	44,686,664	G	A	0.126	0.116	0.083	1.093	0.051	0.988	1.208	0.523	Imputed
rs7893354	10	44,687,276	G	T	0.010	0.013	0.150	0.790	0.164	0.573	1.090	0.643	Imputed
MERGED_DEL_2_59567	10	44,687,305	C	ACTCTCTGTACTTTCACTC/	0.028	0.030	0.652	0.956	0.101	0.784	1.165	0.027	Imputed
rs11238907	10	44,687,780	G	T	0.126	0.116	0.084	1.092	0.051	0.988	1.208	0.525	Imputed
rs11238908	10	44,688,080	G	T	0.114	0.101	0.020	1.132	0.054	1.019	1.258	0.520	Imputed
rs11238909	10	44,688,088	T	C	0.114	0.101	0.019	1.133	0.054	1.020	1.259	0.494	Imputed
rs61855759	10	44,688,128	A	G	0.039	0.039	0.870	0.986	0.087	0.831	1.170	0.444	Imputed
rs10899998	10	44,688,524	C	G	0.114	0.101	0.021	1.131	0.054	1.019	1.257	0.525	Imputed
rs7917089	10	44,688,587	A	G	0.127	0.116	0.078	1.094	0.051	0.990	1.210	0.575	Imputed
rs61857460	10	44,688,877	C	A	0.108	0.097	0.037	1.121	0.055	1.007	1.248	0.588	Imputed
rs7909598	10	44,688,935	C	T	0.190	0.172	6.47E-03	1.125	0.043	1.034	1.225	0.653	Imputed
rs112609292	10	44,689,200	T	C	0.032	0.032	0.843	0.981	0.096	0.813	1.184	0.190	Imputed
rs6593403	10	44,689,394	A	G	0.181	0.181	0.969	0.998	0.044	0.916	1.088	0.131	Imputed
rs6593404	10	44,689,406	C	A	0.194	0.190	0.606	1.022	0.043	0.940	1.112	0.219	Imputed

rs1704229	10	44,689,529	A	G	0.128	0.117	0.057	1.102	0.051	0.997	1.217	0.597	Imputed
chr10:44689756:D	10	44,689,756	T	TG	0.108	0.097	0.037	1.121	0.055	1.007	1.248	0.588	Imputed
rs77259563	10	44,689,955	A	C	0.069	0.062	0.044	1.144	0.067	1.003	1.305	0.949	Imputed
rs10899999	10	44,690,185	G	A	0.125	0.114	0.073	1.096	0.051	0.991	1.213	0.564	Imputed
rs2802478	10	44,690,235	A	G	0.029	0.030	0.678	0.960	0.100	0.789	1.167	0.023	Imputed
rs12268863	10	44,690,620	A	G	0.011	0.013	0.155	0.795	0.162	0.578	1.092	0.584	Imputed
rs11591406	10	44,690,710	A	G	0.108	0.097	0.037	1.121	0.055	1.007	1.248	0.588	Imputed
rs1623851	10	44,691,241	G	A	0.191	0.173	6.28E-03	1.125	0.043	1.034	1.225	0.633	Genotyped
rs1704231	10	44,691,543	T	C	0.121	0.112	0.133	1.081	0.052	0.976	1.197	0.470	Imputed
rs1626459	10	44,691,548	C	T	0.127	0.116	0.081	1.093	0.051	0.989	1.209	0.581	Imputed
rs1627329	10	44,691,633	C	T	0.190	0.172	6.57E-03	1.125	0.043	1.033	1.225	0.655	Imputed
rs76731931	10	44,691,723	T	G	0.064	0.056	0.045	1.151	0.070	1.003	1.319	0.941	Imputed
rs76818605	10	44,691,981	C	G	0.064	0.056	0.045	1.151	0.070	1.003	1.319	0.941	Imputed
rs12266055	10	44,692,236	G	A	0.010	0.013	0.154	0.792	0.164	0.574	1.093	0.637	Imputed
rs77449969	10	44,692,444	A	C	0.066	0.058	0.039	1.153	0.069	1.007	1.319	0.713	Imputed
chr10:44692452:D	10	44,692,452	G	GT	0.083	0.078	0.314	1.064	0.062	0.943	1.201	0.921	Imputed
chr10:44692457:D	10	44,692,457	G	GT	0.053	0.047	0.113	1.128	0.076	0.972	1.310	0.999	Imputed
chr10:44692458:I	10	44,692,458	TTTGG	T	0.083	0.077	0.140	1.095	0.062	0.971	1.236	0.775	Imputed
rs2804034	10	44,692,552	C	T	0.419	0.399	0.021	1.082	0.034	1.012	1.158	0.932	Imputed
rs78783043	10	44,692,678	T	C	0.065	0.058	0.055	1.141	0.069	0.997	1.306	0.814	Imputed
rs10160170	10	44,692,843	G	A	0.119	0.110	0.142	1.080	0.053	0.974	1.197	0.391	Imputed
rs10508883	10	44,693,544	C	A	0.014	0.015	0.397	0.885	0.145	0.666	1.175	0.679	Imputed
rs117030693	10	44,693,712	C	T	0.064	0.056	0.041	1.153	0.070	1.006	1.322	0.954	Imputed
rs7900182	10	44,693,742	G	T	0.186	0.186	0.926	0.996	0.044	0.915	1.085	0.167	Imputed
rs189932865	10	44,693,808	T	C	0.023	0.020	0.140	1.184	0.114	0.946	1.481	0.870	Imputed
rs12262428	10	44,693,901	G	C	0.014	0.015	0.413	0.888	0.145	0.669	1.180	0.623	Imputed
rs72788723	10	44,693,970	T	C	0.016	0.017	0.636	0.938	0.136	0.719	1.224	0.804	Imputed
rs11597731	10	44,694,201	T	C	0.187	0.187	0.930	0.996	0.043	0.915	1.085	0.132	Genotyped
rs12570491	10	44,694,606	T	G	0.251	0.253	0.570	0.978	0.039	0.906	1.056	0.289	Genotyped
rs10900000	10	44,694,892	A	G	0.109	0.097	0.035	1.123	0.055	1.008	1.250	0.492	Imputed
rs7902040	10	44,695,308	G	A	0.110	0.098	0.022	1.133	0.054	1.018	1.260	0.462	Genotyped
rs112516368	10	44,695,482	A	G	0.016	0.011	0.010	1.428	0.140	1.086	1.879	0.813	Imputed
rs10900001	10	44,695,585	G	C	0.109	0.097	0.034	1.123	0.055	1.009	1.250	0.488	Imputed
rs79104299	10	44,695,640	A	G	0.065	0.057	0.043	1.151	0.069	1.005	1.319	0.841	Imputed
rs11238911	10	44,695,862	A	G	0.109	0.097	0.033	1.124	0.055	1.009	1.251	0.425	Imputed
rs11238912	10	44,695,909	C	T	0.109	0.097	0.033	1.123	0.055	1.009	1.250	0.427	Imputed
rs11238913	10	44,695,973	C	T	0.365	0.344	0.012	1.093	0.035	1.020	1.171	0.354	Imputed
rs2802477	10	44,696,034	G	A	0.390	0.419	5.82E-04	0.888	0.035	0.830	0.950	0.588	Genotyped
rs72788727	10	44,696,149	C	G	0.183	0.184	0.811	0.990	0.044	0.908	1.078	0.173	Imputed
rs11594522	10	44,696,352	A	G	0.183	0.184	0.811	0.990	0.044	0.908	1.078	0.173	Imputed

chr10:44696529:D	10	44,696,529	C	CAG	0.188	0.187	0.957	1.002	0.043	0.921	1.091	0.105	Imputed
rs11238915	10	44,696,665	A	C	0.109	0.097	0.033	1.123	0.055	1.009	1.250	0.427	Imputed
rs11238916	10	44,696,781	C	G	0.108	0.097	0.036	1.122	0.055	1.007	1.249	0.391	Imputed
rs6593405	10	44,696,879	C	G	0.354	0.334	0.016	1.089	0.035	1.016	1.167	0.302	Imputed
rs7068801	10	44,696,978	G	C	0.191	0.190	0.815	1.010	0.043	0.928	1.099	0.095	Imputed
rs149593252	10	44,697,011	A	G	0.065	0.057	0.040	1.153	0.069	1.006	1.320	0.750	Imputed
rs10900002	10	44,697,564	T	A	0.108	0.097	0.041	1.118	0.055	1.004	1.245	0.396	Imputed
rs7894238	10	44,697,616	A	G	0.183	0.185	0.809	0.990	0.044	0.908	1.078	0.173	Genotyped
rs7906261	10	44,697,755	T	C	0.310	0.301	0.290	1.040	0.037	0.967	1.117	0.422	Imputed
rs17389211	10	44,698,016	T	C	0.183	0.184	0.804	0.989	0.044	0.908	1.078	0.172	Imputed
rs116487021	10	44,698,030	G	T	0.065	0.057	0.042	1.152	0.069	1.005	1.319	0.844	Imputed
rs10900003	10	44,698,044	A	C	0.108	0.097	0.041	1.118	0.055	1.004	1.245	0.396	Imputed
rs2054620	10	44,698,075	C	T	0.366	0.345	0.011	1.094	0.035	1.021	1.172	0.459	Genotyped
rs72788733	10	44,698,096	T	C	0.183	0.184	0.804	0.989	0.044	0.908	1.078	0.172	Imputed
rs61857461	10	44,698,155	G	C	0.031	0.031	0.779	0.973	0.098	0.803	1.179	0.149	Imputed
rs2054621	10	44,698,213	C	T	0.364	0.344	0.014	1.090	0.035	1.017	1.168	0.368	Imputed
rs7906240	10	44,698,397	A	G	0.188	0.187	0.971	1.002	0.043	0.920	1.090	0.107	Imputed
rs112611703	10	44,698,400	T	C	0.016	0.011	6.24E-03	1.457	0.138	1.112	1.911	0.503	Imputed
rs7100370	10	44,698,508	C	A	0.108	0.097	0.041	1.118	0.055	1.004	1.245	0.396	Imputed
rs7082177	10	44,698,630	A	G	0.108	0.097	0.041	1.118	0.055	1.004	1.245	0.396	Imputed
rs7895640	10	44,698,668	A	T	0.183	0.184	0.804	0.989	0.044	0.908	1.078	0.172	Imputed
rs7086639	10	44,698,695	T	C	0.108	0.097	0.041	1.118	0.055	1.004	1.245	0.396	Imputed
rs72788735	10	44,699,424	C	T	0.030	0.025	0.052	1.215	0.100	0.998	1.478	0.755	Imputed
rs881828	10	44,699,562	T	C	0.187	0.187	0.977	0.999	0.043	0.917	1.088	0.112	Imputed
rs7900072	10	44,699,647	C	T	0.108	0.097	0.042	1.118	0.055	1.004	1.245	0.398	Imputed
chr10:44699763:D	10	44,699,763	G	GA	0.244	0.231	0.083	1.071	0.040	0.991	1.158	0.045	Imputed
rs7900062	10	44,699,801	G	A	0.046	0.041	0.142	1.127	0.081	0.961	1.321	0.651	Imputed
rs11238921	10	44,699,910	T	G	0.359	0.341	0.026	1.082	0.035	1.010	1.160	0.395	Imputed
rs12573602	10	44,700,120	A	G	0.187	0.187	0.977	0.999	0.043	0.917	1.088	0.112	Imputed
rs12573610	10	44,700,218	A	G	0.183	0.184	0.804	0.989	0.044	0.908	1.078	0.172	Imputed
rs78921357	10	44,700,227	A	G	0.065	0.057	0.042	1.152	0.069	1.005	1.319	0.844	Imputed
chr10:44700375:I	10	44,700,375	AT	A	0.199	0.172	4.33E-05	1.191	0.043	1.095	1.295	0.539	Imputed
rs12572886	10	44,700,378	A	T	0.187	0.186	0.983	0.999	0.043	0.918	1.088	0.108	Imputed
rs2804032	10	44,700,389	G	C	0.456	0.435	0.020	1.082	0.034	1.013	1.157	0.879	Imputed
rs12572910	10	44,700,657	G	T	0.183	0.184	0.801	0.989	0.044	0.908	1.078	0.171	Imputed
rs12571719	10	44,700,731	A	C	0.187	0.187	0.973	0.999	0.043	0.917	1.087	0.111	Imputed
rs17480411	10	44,700,753	T	C	0.183	0.184	0.801	0.989	0.044	0.908	1.078	0.171	Imputed
rs17389302	10	44,701,054	T	C	0.068	0.069	0.682	0.973	0.067	0.853	1.110	0.810	Imputed
rs11238923	10	44,701,232	T	A	0.108	0.097	0.047	1.115	0.055	1.001	1.242	0.411	Imputed
rs75482055	10	44,701,308	G	T	0.065	0.057	0.042	1.152	0.069	1.005	1.319	0.844	Imputed

rs7097076	10	44,701,352	A	C	0.108	0.097	0.046	1.115	0.055	1.002	1.242	0.409	Imputed
rs117999233	10	44,701,478	A	T	0.078	0.069	0.038	1.141	0.064	1.007	1.293	0.841	Imputed
rs1680639	10	44,701,754	G	T	0.108	0.097	0.042	1.118	0.055	1.004	1.245	0.398	Imputed
rs74901982	10	44,701,880	G	A	0.065	0.057	0.042	1.152	0.069	1.005	1.319	0.946	Imputed
rs76755610	10	44,701,988	C	T	0.065	0.057	0.041	1.152	0.069	1.006	1.320	0.950	Imputed
rs115718339	10	44,702,147	T	A	0.065	0.057	0.033	1.159	0.069	1.012	1.328	0.952	Imputed
rs78116779	10	44,702,177	T	G	0.064	0.057	0.044	1.150	0.069	1.004	1.318	0.906	Imputed
rs1680640	10	44,702,276	A	C	0.365	0.344	0.011	1.094	0.035	1.021	1.172	0.432	Genotyped
rs79728267	10	44,702,383	C	G	0.064	0.057	0.043	1.151	0.069	1.004	1.319	0.909	Imputed
rs75692060	10	44,702,422	C	G	0.064	0.057	0.043	1.151	0.069	1.004	1.319	0.909	Imputed
rs80336515	10	44,702,546	C	A	0.064	0.057	0.043	1.151	0.069	1.004	1.319	0.909	Imputed
rs768676	10	44,702,681	A	T	0.039	0.043	0.167	0.888	0.087	0.749	1.052	0.055	Imputed
chr10:44702714:I	10	44,702,714	GA	G	0.155	0.134	3.22E-04	1.185	0.047	1.080	1.300	0.976	Imputed
rs77809667	10	44,702,809	A	G	0.065	0.057	0.039	1.154	0.069	1.008	1.322	0.960	Imputed
rs114597445	10	44,702,895	G	A	0.064	0.057	0.043	1.151	0.069	1.004	1.319	0.909	Imputed
rs2804031	10	44,702,928	G	T	0.108	0.097	0.037	1.121	0.055	1.007	1.248	0.382	Imputed
rs2664856	10	44,702,967	T	G	0.108	0.097	0.037	1.121	0.055	1.007	1.248	0.384	Imputed
rs78943887	10	44,702,976	A	G	0.064	0.057	0.043	1.151	0.069	1.004	1.319	0.909	Imputed
rs17480530	10	44,703,214	G	C	0.183	0.184	0.790	0.988	0.044	0.907	1.077	0.176	Imputed
rs7897728	10	44,703,260	T	C	0.188	0.187	0.906	1.005	0.043	0.923	1.094	0.100	Imputed
rs117285640	10	44,703,367	A	G	0.010	0.011	0.480	0.887	0.170	0.636	1.237	0.787	Imputed
rs17155635	10	44,703,545	C	G	0.185	0.185	0.996	1.000	0.044	0.918	1.089	0.107	Imputed
rs11238925	10	44,703,730	G	A	0.108	0.097	0.037	1.121	0.055	1.007	1.248	0.382	Imputed
chr10:44703934:I	10	44,703,934	CTT	C	0.064	0.057	0.040	1.154	0.070	1.007	1.322	0.860	Imputed
rs190908222	10	44,703,940	A	C	0.064	0.057	0.040	1.154	0.070	1.007	1.322	0.860	Imputed
rs7901938	10	44,704,065	T	C	0.185	0.185	0.996	1.000	0.044	0.918	1.089	0.107	Imputed
rs11238927	10	44,704,112	T	C	0.108	0.097	0.037	1.121	0.055	1.007	1.248	0.382	Imputed
rs76183815	10	44,704,207	T	C	0.064	0.057	0.040	1.154	0.070	1.007	1.322	0.860	Imputed
rs7918355	10	44,704,259	G	A	0.362	0.342	0.013	1.091	0.035	1.019	1.170	0.413	Imputed
rs12355852	10	44,704,280	T	G	0.108	0.097	0.037	1.121	0.055	1.007	1.248	0.382	Imputed
rs7921850	10	44,704,401	C	T	0.362	0.342	0.013	1.092	0.035	1.019	1.170	0.415	Imputed
rs118066217	10	44,704,776	A	C	0.064	0.057	0.051	1.145	0.070	0.999	1.313	0.915	Imputed
rs7906325	10	44,705,019	T	C	0.012	0.014	0.351	0.865	0.155	0.638	1.173	0.484	Genotyped
chr10:44705167:D	10	44,705,167	A	AG	0.064	0.057	0.049	1.146	0.070	1.000	1.314	0.920	Imputed
rs58540467	10	44,705,170	C	T	0.108	0.097	0.033	1.124	0.055	1.010	1.251	0.368	Imputed
rs75546245	10	44,705,191	T	C	0.065	0.059	0.060	1.138	0.069	0.994	1.303	0.871	Imputed
rs75601172	10	44,705,324	G	A	0.064	0.057	0.051	1.145	0.070	0.999	1.312	0.913	Imputed
rs906944	10	44,705,439	A	G	0.108	0.097	0.033	1.124	0.055	1.009	1.251	0.370	Imputed
rs12570720	10	44,705,453	T	G	0.183	0.184	0.815	0.990	0.044	0.908	1.078	0.156	Imputed
rs79787081	10	44,705,476	A	G	0.064	0.057	0.051	1.145	0.070	0.999	1.312	0.913	Imputed

rs78005216	10	44,705,485	C	T	0.064	0.057	0.051	1.145	0.070	0.999	1.312	0.913	Imputed
rs61857479	10	44,705,516	G	A	0.173	0.154	2.35E-03	1.147	0.045	1.050	1.252	0.534	Imputed
rs17598923	10	44,705,747	T	C	0.065	0.057	0.042	1.152	0.069	1.005	1.319	0.745	Genotyped
rs3865770	10	44,705,969	A	G	0.183	0.184	0.815	0.990	0.044	0.908	1.078	0.156	Imputed
rs76512791	10	44,706,228	G	A	0.064	0.057	0.051	1.145	0.070	0.999	1.312	0.913	Imputed
chr10:44706264:D	10	44,706,264	A	AT	0.108	0.097	0.034	1.123	0.055	1.009	1.250	0.384	Imputed
rs116524689	10	44,706,702	G	A	0.064	0.057	0.051	1.145	0.070	0.999	1.312	0.912	Imputed
rs58129751	10	44,706,720	A	G	0.108	0.097	0.035	1.122	0.055	1.008	1.249	0.405	Imputed
chr10:44707570:D	10	44,707,570	A	AT	0.176	0.157	3.05E-03	1.141	0.045	1.046	1.246	0.427	Imputed
chr10:44707577:D	10	44,707,577	T	TG	0.021	0.021	0.948	1.008	0.119	0.798	1.273	0.569	Imputed
rs1482473	10	44,707,598	C	G	0.177	0.156	1.01E-03	1.158	0.045	1.061	1.264	0.530	Imputed
rs17155641	10	44,708,346	G	A	0.177	0.156	6.73E-04	1.164	0.045	1.066	1.270	0.507	Imputed
rs11238929	10	44,708,596	A	T	0.109	0.097	0.034	1.123	0.055	1.009	1.250	0.369	Imputed
rs116688124	10	44,709,029	C	G	0.064	0.057	0.051	1.145	0.070	0.999	1.312	0.913	Imputed
rs72788737	10	44,709,168	T	G	0.187	0.187	0.990	0.999	0.043	0.918	1.088	0.102	Imputed
rs3851257	10	44,709,171	T	G	0.365	0.344	0.012	1.092	0.035	1.019	1.170	0.454	Imputed
rs77858453	10	44,709,366	A	G	0.064	0.057	0.051	1.145	0.070	0.999	1.312	0.913	Imputed
rs11238930	10	44,709,452	A	C	0.019	0.019	0.808	1.030	0.123	0.809	1.312	0.780	Imputed
rs1680641	10	44,709,657	G	A	0.177	0.156	1.01E-03	1.158	0.045	1.061	1.264	0.530	Imputed
rs3952245	10	44,709,741	A	G	0.187	0.187	0.990	0.999	0.043	0.918	1.088	0.102	Imputed
rs113598887	10	44,710,033	C	A	0.016	0.011	6.99E-03	1.452	0.139	1.106	1.906	0.776	Imputed
rs9787462	10	44,710,075	A	T	0.183	0.185	0.761	0.987	0.044	0.906	1.075	0.145	Imputed
rs9787518	10	44,710,171	A	G	0.183	0.185	0.761	0.987	0.044	0.906	1.075	0.145	Imputed
rs1704222	10	44,710,465	A	T	0.109	0.097	0.034	1.123	0.055	1.009	1.250	0.369	Imputed
rs117885909	10	44,710,592	A	T	0.013	0.011	0.521	1.103	0.153	0.818	1.489	0.686	Imputed
chr10:44710669:D	10	44,710,669	C	CTG	0.064	0.057	0.051	1.145	0.070	0.999	1.312	0.913	Imputed
rs115305466	10	44,710,670	A	T	0.064	0.057	0.051	1.145	0.070	0.999	1.312	0.913	Imputed
rs116783823	10	44,710,671	A	G	0.064	0.057	0.051	1.145	0.070	0.999	1.312	0.913	Imputed
rs1704221	10	44,710,930	A	G	0.109	0.097	0.033	1.124	0.055	1.009	1.251	0.366	Imputed
rs1704220	10	44,711,016	C	T	0.364	0.343	0.011	1.094	0.035	1.021	1.172	0.420	Imputed
rs3979460	10	44,711,269	A	G	0.183	0.185	0.750	0.986	0.044	0.905	1.075	0.143	Imputed
rs114435799	10	44,711,328	T	C	0.064	0.057	0.051	1.145	0.070	0.999	1.312	0.913	Imputed
chr10:44711556:I	10	44,711,556	ACAATCCC	A	0.369	0.346	3.74E-03	1.107	0.035	1.034	1.186	0.503	Imputed
rs55926521	10	44,711,567	T	G	0.117	0.109	0.165	1.076	0.053	0.970	1.194	0.244	Imputed
rs75554500	10	44,711,812	T	C	0.064	0.057	0.051	1.145	0.070	0.999	1.312	0.913	Imputed
chr10:44712036:I	10	44,712,036	ATAGAG	A	0.359	0.340	0.018	1.087	0.035	1.015	1.165	0.758	Imputed
rs1680642	10	44,712,096	A	G	0.364	0.343	0.012	1.093	0.035	1.020	1.171	0.416	Imputed
rs12573558	10	44,712,128	A	C	0.185	0.186	0.814	0.990	0.044	0.909	1.078	0.160	Imputed
rs141391332	10	44,712,229	G	A	0.016	0.011	6.99E-03	1.452	0.139	1.106	1.906	0.776	Imputed
rs118105294	10	44,712,279	T	C	0.017	0.017	0.985	0.998	0.131	0.773	1.288	0.558	Imputed

chr10:44712318:D	10	44,712,318	A	AAAT	0.183	0.185	0.699	0.983	0.044	0.902	1.071	0.152	Imputed
rs966161	10	44,712,574	T	C	0.109	0.097	0.034	1.123	0.055	1.009	1.250	0.369	Imputed
rs1482477	10	44,713,185	G	T	0.364	0.343	0.011	1.094	0.035	1.021	1.172	0.424	Imputed
rs1905150	10	44,713,311	A	G	0.178	0.157	7.71E-04	1.161	0.045	1.064	1.267	0.595	Genotyped
rs2224769	10	44,713,475	A	C	0.183	0.185	0.754	0.986	0.044	0.905	1.075	0.144	Imputed
rs11238933	10	44,714,250	G	C	0.109	0.097	0.035	1.122	0.055	1.008	1.250	0.371	Imputed
rs11238935	10	44,714,402	T	C	0.109	0.097	0.035	1.122	0.055	1.008	1.249	0.373	Imputed
rs11594110	10	44,714,528	G	A	0.183	0.185	0.785	0.988	0.044	0.907	1.077	0.144	Imputed
rs1746034	10	44,714,768	C	T	0.109	0.097	0.035	1.122	0.055	1.008	1.250	0.371	Imputed
rs7911865	10	44,714,973	T	A	0.187	0.187	0.973	0.999	0.043	0.917	1.087	0.094	Imputed
chr10:44715181:I	10	44,715,181	CA	C	0.109	0.097	0.025	1.130	0.055	1.015	1.258	0.373	Imputed
rs6593406	10	44,715,200	T	G	0.187	0.187	0.977	0.999	0.043	0.917	1.088	0.092	Imputed
rs74936961	10	44,715,454	G	A	0.009	0.012	0.062	0.722	0.175	0.513	1.017	0.718	Imputed
rs72788744	10	44,715,491	C	A	0.175	0.173	0.808	1.011	0.045	0.926	1.103	0.188	Imputed
rs11595176	10	44,715,598	C	T	0.187	0.187	0.971	0.998	0.043	0.917	1.087	0.091	Imputed
rs61857481	10	44,715,733	C	A	0.021	0.023	0.388	0.905	0.117	0.720	1.137	0.290	Imputed
rs11511598	10	44,716,036	T	C	0.109	0.097	0.035	1.122	0.055	1.008	1.250	0.371	Imputed
rs2209066	10	44,716,144	G	A	0.187	0.187	0.975	0.999	0.043	0.917	1.087	0.092	Imputed
rs2209067	10	44,716,469	A	G	0.187	0.187	0.952	0.997	0.043	0.916	1.086	0.105	Imputed
rs75551514	10	44,716,475	G	A	0.064	0.057	0.051	1.145	0.070	0.999	1.312	0.913	Imputed
chr10:44716876:I	10	44,716,876	CT	C	0.278	0.258	7.14E-03	1.108	0.038	1.028	1.193	0.093	Imputed
rs1334316	10	44,716,885	T	A	0.296	0.284	0.123	1.059	0.037	0.985	1.139	0.496	Imputed
rs61857482	10	44,716,905	T	C	0.109	0.097	0.035	1.122	0.055	1.008	1.249	0.373	Imputed
rs77125692	10	44,716,982	T	C	0.038	0.045	0.046	0.839	0.088	0.707	0.997	0.209	Imputed
rs74639149	10	44,717,166	C	A	0.064	0.057	0.050	1.146	0.070	1.000	1.313	0.916	Imputed
rs4245612	10	44,717,447	G	C	0.381	0.356	1.90E-03	1.115	0.035	1.041	1.193	0.451	Imputed
chr10:44717485:D	10	44,717,485	T	TATA	0.064	0.057	0.050	1.146	0.070	1.000	1.313	0.916	Imputed
chr10:44717499:I	10	44,717,499	ATG	A	0.364	0.344	0.014	1.090	0.035	1.018	1.168	0.455	Imputed
rs4415709	10	44,717,531	C	T	0.064	0.057	0.050	1.146	0.070	1.000	1.313	0.916	Imputed
rs183531569	10	44,717,621	C	A	0.011	0.011	0.985	1.003	0.163	0.729	1.379	0.735	Imputed
rs77412767	10	44,719,165	T	G	0.064	0.057	0.053	1.144	0.070	0.998	1.312	0.907	Imputed
rs61857484	10	44,719,215	A	C	0.109	0.098	0.036	1.121	0.055	1.007	1.248	0.403	Imputed
rs61857485	10	44,719,292	T	C	0.109	0.097	0.030	1.126	0.055	1.012	1.253	0.454	Imputed
rs61857486	10	44,719,361	G	A	0.109	0.097	0.033	1.123	0.055	1.009	1.250	0.409	Imputed
rs77212901	10	44,719,491	G	C	0.064	0.057	0.050	1.146	0.070	1.000	1.313	0.916	Imputed
rs11238937	10	44,719,816	A	T	0.109	0.097	0.033	1.123	0.055	1.009	1.250	0.409	Imputed
rs10900005	10	44,720,143	A	G	0.364	0.343	0.013	1.091	0.035	1.018	1.169	0.426	Imputed
rs75415231	10	44,720,300	G	A	0.064	0.057	0.050	1.146	0.070	1.000	1.313	0.916	Imputed
rs61857488	10	44,720,540	C	T	0.019	0.019	0.905	1.015	0.124	0.796	1.294	0.833	Imputed
rs56253458	10	44,720,778	A	G	0.183	0.184	0.796	0.989	0.044	0.907	1.077	0.140	Imputed

rs2038676	10	44,720,951	T	G	0.364	0.343	0.013	1.091	0.035	1.018	1.169	0.418	Imputed
rs77192376	10	44,721,373	T	C	0.064	0.057	0.050	1.146	0.070	1.000	1.313	0.916	Imputed
rs11597129	10	44,721,411	A	G	0.385	0.359	1.36E-03	1.118	0.035	1.044	1.197	0.500	Imputed
rs144189083	10	44,721,450	T	G	0.023	0.025	0.433	0.917	0.112	0.736	1.141	0.035	Imputed
rs11238940	10	44,721,459	A	G	0.113	0.102	0.031	1.123	0.054	1.011	1.247	0.290	Imputed
rs146548505	10	44,721,468	G	T	0.020	0.015	0.011	1.365	0.123	1.073	1.737	0.940	Imputed
rs148389281	10	44,721,848	G	A	0.064	0.057	0.050	1.146	0.070	1.000	1.313	0.916	Imputed
rs145661168	10	44,722,289	G	A	0.015	0.015	0.768	1.042	0.140	0.792	1.371	0.516	Imputed
rs2209068	10	44,722,308	G	T	0.350	0.330	0.015	1.090	0.036	1.017	1.169	0.986	Imputed
rs137976681	10	44,722,356	C	T	0.064	0.057	0.050	1.146	0.070	1.000	1.313	0.916	Imputed
rs2209069	10	44,722,489	T	C	0.173	0.174	0.925	0.996	0.045	0.912	1.087	0.549	Imputed
rs115441801	10	44,722,649	C	T	0.064	0.056	0.041	1.153	0.070	1.006	1.322	0.957	Imputed
chr10:44722690:D	10	44,722,690	A	AAGTTT	0.013	0.013	0.917	1.016	0.150	0.758	1.362	0.001	Imputed
chr10:44722790:I	10	44,722,790	GCTGTTTTGATTA	G	0.290	0.276	0.087	1.066	0.037	0.991	1.147	0.979	Imputed
rs146007102	10	44,723,194	G	T	0.064	0.057	0.059	1.141	0.070	0.995	1.308	0.923	Imputed
rs10793526	10	44,723,344	C	T	0.350	0.330	0.014	1.091	0.036	1.018	1.170	0.946	Imputed
rs151074559	10	44,723,524	T	C	0.064	0.057	0.060	1.140	0.070	0.995	1.307	0.919	Imputed
rs149779784	10	44,723,551	A	G	0.064	0.057	0.060	1.140	0.070	0.995	1.307	0.919	Imputed
rs11238943	10	44,723,623	T	A	0.109	0.097	0.026	1.129	0.055	1.015	1.257	0.396	Imputed
rs4948615	10	44,723,741	T	C	0.350	0.330	0.012	1.093	0.036	1.019	1.172	0.959	Imputed
rs72788749	10	44,723,775	A	G	0.170	0.172	0.773	0.987	0.045	0.904	1.078	0.669	Imputed
rs11238944	10	44,723,801	C	T	0.109	0.097	0.029	1.127	0.055	1.012	1.254	0.409	Imputed
rs142019897	10	44,723,967	C	T	0.064	0.057	0.060	1.140	0.070	0.995	1.307	0.919	Imputed
rs11517251	10	44,724,052	G	A	0.109	0.097	0.026	1.129	0.055	1.014	1.256	0.400	Imputed
rs11511599	10	44,724,172	T	C	0.109	0.097	0.026	1.129	0.055	1.015	1.257	0.398	Imputed
rs56001350	10	44,724,233	A	G	0.145	0.143	0.857	1.009	0.048	0.918	1.109	0.365	Imputed
rs56063172	10	44,724,234	T	C	0.148	0.146	0.754	1.015	0.048	0.924	1.115	0.435	Imputed
rs55889486	10	44,724,254	T	C	0.160	0.159	0.782	1.013	0.046	0.925	1.109	0.481	Imputed
rs11492882	10	44,724,322	C	G	0.109	0.097	0.026	1.129	0.055	1.014	1.256	0.400	Imputed
rs117243802	10	44,724,367	A	G	0.036	0.038	0.688	0.964	0.090	0.808	1.151	0.166	Imputed
rs187736657	10	44,724,554	T	G	0.015	0.015	0.731	1.049	0.139	0.799	1.377	0.650	Imputed
rs11238945	10	44,724,581	G	A	0.350	0.330	0.012	1.093	0.036	1.020	1.172	0.957	Imputed
rs11238946	10	44,724,842	A	G	0.109	0.097	0.027	1.128	0.055	1.014	1.256	0.402	Imputed
rs11238947	10	44,725,030	G	A	0.110	0.097	0.018	1.138	0.054	1.022	1.266	0.548	Imputed
rs148752891	10	44,725,304	A	C	0.041	0.051	4.92E-03	0.788	0.085	0.668	0.931	0.259	Imputed
rs117910195	10	44,725,381	T	C	0.170	0.172	0.738	0.985	0.045	0.902	1.076	0.685	Imputed
rs114725146	10	44,725,406	G	T	0.064	0.057	0.057	1.141	0.070	0.996	1.309	0.894	Imputed
rs150252111	10	44,726,297	C	G	0.027	0.022	0.063	1.220	0.107	0.989	1.504	0.435	Imputed
rs113059641	10	44,726,620	C	T	0.034	0.028	0.062	1.195	0.095	0.992	1.441	0.024	Imputed
rs7921861	10	44,726,655	T	C	0.386	0.360	1.55E-03	1.116	0.035	1.043	1.195	0.572	Imputed

rs7913299	10	44,726,720	A	T	0.170	0.170	0.942	1.003	0.045	0.919	1.096	0.647	Imputed
rs115706337	10	44,726,781	T	C	0.064	0.057	0.057	1.141	0.070	0.996	1.309	0.894	Imputed
rs77861488	10	44,726,934	G	T	0.064	0.057	0.057	1.141	0.070	0.996	1.309	0.894	Imputed
rs6593407	10	44,726,975	G	A	0.351	0.331	9.62E-03	1.096	0.036	1.023	1.175	0.876	Imputed
rs11517223	10	44,727,038	C	T	0.111	0.097	0.014	1.143	0.054	1.028	1.272	0.348	Imputed
rs11511571	10	44,727,167	C	T	0.111	0.098	0.017	1.138	0.054	1.023	1.266	0.312	Imputed
rs116413589	10	44,727,369	A	T	0.064	0.057	0.062	1.139	0.070	0.993	1.306	0.852	Imputed
rs149377868	10	44,727,618	A	G	0.064	0.057	0.061	1.140	0.070	0.994	1.307	0.853	Imputed
rs112497268	10	44,727,831	G	A	0.036	0.038	0.688	0.964	0.090	0.808	1.151	0.166	Imputed
rs1746040	10	44,727,838	A	G	0.352	0.331	8.59E-03	1.098	0.036	1.024	1.177	0.880	Imputed
chr10:44727872:D	10	44,727,872	T	TC	0.341	0.321	0.014	1.092	0.036	1.018	1.171	0.969	Imputed
rs72788751	10	44,727,924	T	C	0.170	0.172	0.736	0.985	0.045	0.902	1.076	0.649	Imputed
rs113235064	10	44,728,054	A	G	0.035	0.029	0.031	1.225	0.093	1.020	1.471	0.063	Imputed
rs76348658	10	44,728,442	T	C	0.064	0.057	0.052	1.145	0.070	0.999	1.312	0.878	Imputed
rs7906368	10	44,728,592	A	G	0.173	0.174	0.898	0.994	0.045	0.911	1.085	0.537	Imputed
rs7073818	10	44,728,797	C	T	0.350	0.330	0.011	1.094	0.036	1.021	1.173	0.990	Imputed
rs11597208	10	44,729,184	C	T	0.170	0.172	0.740	0.985	0.045	0.902	1.076	0.651	Imputed
rs115213152	10	44,729,291	A	C	0.064	0.057	0.052	1.145	0.070	0.999	1.312	0.878	Imputed
rs1704217	10	44,729,307	C	T	0.109	0.097	0.026	1.129	0.055	1.014	1.256	0.433	Imputed
rs75175972	10	44,729,553	G	A	0.064	0.057	0.051	1.145	0.070	0.999	1.313	0.881	Imputed
rs1704218	10	44,729,599	T	C	0.109	0.097	0.023	1.132	0.055	1.017	1.260	0.507	Imputed
rs7899802	10	44,729,744	T	A	0.170	0.172	0.679	0.982	0.045	0.899	1.072	0.670	Imputed
rs1704219	10	44,729,958	C	G	0.350	0.330	0.011	1.094	0.036	1.020	1.173	0.997	Imputed
rs11238948	10	44,730,165	A	T	0.061	0.054	0.072	1.137	0.071	0.989	1.308	0.455	Imputed
rs7079375	10	44,730,310	C	T	0.110	0.097	0.021	1.134	0.055	1.019	1.262	0.466	Imputed
rs7918527	10	44,730,775	A	G	0.241	0.233	0.231	1.049	0.040	0.970	1.133	0.605	Imputed
rs2505745	10	44,730,964	G	A	0.424	0.403	0.013	1.089	0.034	1.019	1.165	0.674	Imputed
rs7907961	10	44,730,995	C	T	0.241	0.233	0.268	1.045	0.040	0.967	1.129	0.631	Genotyped
rs2505746	10	44,731,273	G	C	0.461	0.439	0.012	1.089	0.034	1.019	1.164	0.596	Imputed
rs72788756	10	44,731,276	C	A	0.062	0.068	0.173	0.909	0.070	0.793	1.043	0.859	Imputed
rs79801881	10	44,731,451	A	G	0.012	0.010	0.115	1.274	0.155	0.941	1.726	0.237	Imputed
rs596152	10	44,731,627	A	G	0.110	0.097	0.021	1.134	0.055	1.019	1.262	0.466	Imputed
rs58575067	10	44,731,695	T	C	0.241	0.233	0.231	1.049	0.040	0.970	1.133	0.605	Imputed
rs1746042	10	44,731,897	T	C	0.110	0.097	0.021	1.134	0.055	1.019	1.262	0.466	Imputed
rs584411	10	44,731,946	G	C	0.350	0.330	0.011	1.094	0.036	1.020	1.173	0.997	Imputed
rs2505747	10	44,732,332	A	G	0.404	0.430	3.35E-03	0.904	0.034	0.845	0.967	0.884	Imputed
rs556434	10	44,732,519	A	T	0.110	0.097	0.020	1.136	0.055	1.020	1.264	0.476	Imputed
rs1746043	10	44,732,825	C	T	0.350	0.330	0.011	1.094	0.036	1.020	1.173	0.997	Imputed
rs677465	10	44,732,917	A	G	0.110	0.097	0.026	1.129	0.055	1.015	1.257	0.437	Genotyped
rs11592445	10	44,732,945	T	G	0.110	0.097	0.021	1.134	0.055	1.019	1.262	0.464	Imputed

rs677058	10	44,732,973	A	G	0.018	0.014	0.101	1.240	0.131	0.960	1.604	0.136	Imputed
rs77128494	10	44,733,189	A	G	0.047	0.041	0.053	1.168	0.080	0.998	1.368	0.802	Imputed
rs549054	10	44,733,315	T	C	0.037	0.043	0.094	0.864	0.088	0.727	1.026	0.018	Imputed
rs77295392	10	44,733,363	C	T	0.064	0.057	0.053	1.144	0.070	0.998	1.311	0.875	Imputed
rs56203766	10	44,733,382	G	A	0.015	0.016	0.661	0.942	0.137	0.720	1.232	0.895	Imputed
rs1619661	10	44,733,383	C	T	0.110	0.097	0.021	1.134	0.055	1.019	1.262	0.464	Imputed
rs79961501	10	44,733,483	T	C	0.028	0.029	0.641	0.954	0.102	0.781	1.165	0.007	Imputed
chr10:44733520:D	10	44,733,520	G	GA	0.017	0.012	0.034	1.332	0.135	1.021	1.736	0.887	Imputed
rs674746	10	44,733,522	T	A	0.110	0.097	0.020	1.135	0.055	1.020	1.263	0.462	Imputed
rs673778	10	44,733,739	T	G	0.110	0.097	0.019	1.137	0.055	1.021	1.265	0.514	Imputed
rs673354	10	44,733,816	A	C	0.110	0.097	0.021	1.134	0.055	1.019	1.262	0.464	Imputed
rs78619035	10	44,733,999	T	C	0.064	0.057	0.054	1.144	0.070	0.998	1.311	0.937	Imputed
rs191270205	10	44,734,356	A	G	0.150	0.152	0.787	0.987	0.047	0.900	1.083	0.517	Imputed
rs184133661	10	44,734,360	G	T	0.145	0.147	0.778	0.987	0.048	0.898	1.084	0.400	Imputed
rs2781545	10	44,734,391	A	C	0.110	0.098	0.030	1.125	0.054	1.011	1.252	0.519	Imputed
rs1657349	10	44,734,464	G	C	0.110	0.098	0.029	1.126	0.055	1.012	1.253	0.472	Imputed
rs113512102	10	44,734,505	G	A	0.035	0.030	0.084	1.178	0.094	0.980	1.416	0.006	Imputed
rs56366664	10	44,734,559	A	G	0.152	0.155	0.635	0.978	0.047	0.892	1.072	0.684	Imputed
rs1746044	10	44,734,602	C	T	0.110	0.098	0.025	1.130	0.054	1.015	1.257	0.402	Imputed
rs647927	10	44,734,860	C	T	0.287	0.274	0.103	1.063	0.037	0.988	1.144	0.916	Imputed
rs647854	10	44,734,910	A	C	0.353	0.334	0.022	1.085	0.035	1.012	1.163	0.970	Imputed
rs647419	10	44,734,995	A	G	0.374	0.357	0.044	1.073	0.035	1.002	1.149	0.555	Imputed
rs72788758	10	44,735,490	A	G	0.169	0.172	0.685	0.982	0.045	0.899	1.073	0.632	Imputed
rs631414	10	44,736,284	T	C	0.286	0.276	0.195	1.050	0.037	0.975	1.130	0.786	Imputed
rs736136	10	44,736,389	A	G	0.172	0.174	0.782	0.988	0.045	0.905	1.078	0.595	Genotyped
rs545025	10	44,736,544	T	C	0.111	0.100	0.035	1.121	0.054	1.008	1.246	0.403	Imputed
rs76710600	10	44,736,546	G	A	0.027	0.028	0.687	0.960	0.103	0.784	1.174	0.007	Imputed
rs118074410	10	44,736,560	G	A	0.026	0.024	0.639	1.052	0.108	0.852	1.299	0.084	Imputed
rs629213	10	44,736,784	T	C	0.111	0.100	0.035	1.121	0.054	1.008	1.246	0.403	Imputed
rs79345631	10	44,737,022	T	G	0.014	0.017	0.143	0.814	0.141	0.618	1.074	0.151	Imputed
rs88796	10	44,737,036	C	T	0.287	0.276	0.198	1.049	0.037	0.975	1.129	0.828	Imputed
rs617481	10	44,737,144	C	G	0.287	0.276	0.198	1.049	0.037	0.975	1.129	0.828	Imputed
rs617019	10	44,737,246	A	G	0.111	0.100	0.034	1.121	0.054	1.008	1.246	0.401	Imputed
rs10900007	10	44,737,368	T	A	0.175	0.176	0.887	0.994	0.045	0.911	1.084	0.679	Imputed
rs17155733	10	44,737,433	G	A	0.172	0.174	0.807	0.989	0.045	0.906	1.080	0.575	Imputed
rs17657853	10	44,737,535	G	A	0.169	0.171	0.686	0.982	0.045	0.899	1.073	0.694	Imputed
chr10:44738619:D	10	44,738,619	C	CA	0.092	0.086	0.233	1.072	0.059	0.956	1.203	0.901	Imputed
rs583489	10	44,738,688	G	C	0.118	0.105	0.018	1.133	0.053	1.022	1.257	0.428	Imputed
rs680091	10	44,738,932	A	G	0.112	0.100	0.035	1.120	0.054	1.008	1.245	0.406	Imputed
rs676966	10	44,739,594	T	C	0.112	0.100	0.035	1.120	0.054	1.008	1.245	0.406	Imputed

rs2457480	10	44,740,010	G	A	0.108	0.099	0.089	1.097	0.055	0.986	1.222	0.573	Imputed
rs113317060	10	44,740,014	G	A	0.112	0.101	0.044	1.115	0.054	1.003	1.239	0.469	Imputed
rs661697	10	44,740,698	T	G	0.111	0.101	0.055	1.109	0.054	0.998	1.233	0.357	Genotyped
rs498810	10	44,740,776	C	A	0.153	0.134	1.35E-03	1.164	0.047	1.061	1.277	0.929	Imputed
rs649192	10	44,741,179	C	G	0.119	0.106	0.017	1.134	0.053	1.022	1.257	0.471	Imputed
rs494207	10	44,741,256	A	G	0.119	0.106	0.017	1.134	0.053	1.023	1.257	0.481	Imputed
rs646890	10	44,741,710	T	C	0.119	0.106	0.015	1.136	0.053	1.025	1.260	0.531	Imputed
rs2760670	10	44,742,021	T	G	0.119	0.106	0.015	1.136	0.053	1.025	1.259	0.533	Imputed
rs140525041	10	44,742,441	T	G	0.119	0.106	0.017	1.133	0.053	1.022	1.257	0.486	Imputed
rs2476352	10	44,742,469	C	T	0.358	0.342	0.056	1.070	0.035	0.998	1.146	0.669	Imputed
rs77086803	10	44,742,518	A	T	0.110	0.099	0.040	1.118	0.054	1.005	1.244	0.719	Imputed
rs146921255	10	44,742,572	A	G	0.112	0.101	0.040	1.117	0.054	1.005	1.242	0.440	Imputed
rs117713796	10	44,742,798	G	T	0.065	0.057	0.032	1.160	0.069	1.013	1.328	0.891	Imputed
rs114070077	10	44,742,911	G	A	0.119	0.106	0.016	1.135	0.053	1.024	1.259	0.535	Imputed
rs115299170	10	44,742,925	C	T	0.119	0.106	0.016	1.135	0.053	1.024	1.258	0.538	Imputed
rs146862843	10	44,742,971	A	G	0.119	0.106	0.016	1.135	0.053	1.024	1.259	0.535	Imputed
rs617542	10	44,743,636	A	G	0.119	0.105	0.013	1.139	0.053	1.027	1.262	0.488	Imputed
rs150599809	10	44,743,681	A	G	0.015	0.016	0.974	0.996	0.139	0.759	1.306	0.408	Imputed
rs617035	10	44,743,768	C	A	0.119	0.106	0.014	1.138	0.053	1.026	1.261	0.512	Imputed
rs149726213	10	44,743,832	T	C	0.035	0.029	0.027	1.229	0.093	1.024	1.475	0.086	Imputed
rs605414	10	44,744,110	T	C	0.119	0.106	0.013	1.140	0.053	1.028	1.263	0.465	Imputed
rs146787399	10	44,744,206	T	C	0.035	0.029	0.027	1.229	0.093	1.024	1.475	0.086	Imputed
rs144399321	10	44,744,252	T	C	0.111	0.100	0.039	1.118	0.054	1.005	1.243	0.396	Imputed
rs7083765	10	44,744,431	G	A	0.172	0.172	0.939	1.003	0.045	0.919	1.096	0.566	Imputed
rs7099184	10	44,744,492	A	G	0.172	0.172	0.943	1.003	0.045	0.919	1.095	0.564	Imputed
rs2781543	10	44,744,566	G	T	0.111	0.099	0.029	1.125	0.054	1.012	1.251	0.604	Imputed
rs138708625	10	44,744,907	A	G	0.035	0.029	0.027	1.229	0.093	1.024	1.475	0.086	Imputed
rs486234	10	44,744,946	T	G	0.310	0.291	0.013	1.096	0.037	1.020	1.177	0.527	Imputed
rs116111684	10	44,745,146	T	A	0.065	0.057	0.032	1.160	0.069	1.013	1.328	0.891	Imputed
rs589655	10	44,745,315	G	C	0.111	0.100	0.051	1.112	0.054	1.000	1.237	0.436	Imputed
rs587375	10	44,745,808	C	T	0.151	0.131	6.39E-04	1.177	0.048	1.072	1.292	0.715	Imputed
rs684666	10	44,745,847	C	T	0.154	0.135	9.58E-04	1.169	0.047	1.065	1.282	0.833	Imputed
rs684196	10	44,745,950	T	A	0.154	0.135	9.58E-04	1.169	0.047	1.065	1.282	0.833	Imputed
rs683297	10	44,746,140	G	A	0.149	0.131	1.79E-03	1.161	0.048	1.057	1.275	0.732	Imputed
rs541483	10	44,746,395	G	A	0.154	0.134	9.36E-04	1.169	0.047	1.066	1.283	0.931	Genotyped
rs535176	10	44,747,059	T	C	0.154	0.134	7.88E-04	1.172	0.047	1.068	1.285	0.949	Genotyped
rs150091831	10	44,747,196	G	A	0.040	0.044	0.192	0.893	0.086	0.754	1.058	0.401	Imputed
chr10:44747289:D	10	44,747,289	C	CT	0.154	0.134	8.82E-04	1.170	0.047	1.067	1.284	0.869	Imputed
rs635612	10	44,748,512	A	G	0.154	0.134	8.64E-04	1.170	0.047	1.067	1.284	0.871	Imputed
rs474281	10	44,749,120	C	T	0.153	0.134	1.13E-03	1.167	0.047	1.063	1.280	0.888	Imputed

rs473501	10	44,749,171	A	G	0.154	0.134	8.64E-04	1.170	0.047	1.067	1.284	0.871	Imputed
rs622472	10	44,749,211	C	A	0.154	0.134	8.64E-04	1.170	0.047	1.067	1.284	0.871	Imputed
rs112608796	10	44,749,282	A	T	0.035	0.029	0.027	1.230	0.093	1.025	1.476	0.087	Imputed
chr10:44749462:I	10	44,749,462	GA	G	0.154	0.134	8.64E-04	1.170	0.047	1.067	1.284	0.871	Imputed
rs513391	10	44,749,708	C	A	0.154	0.134	8.64E-04	1.170	0.047	1.067	1.284	0.871	Imputed
rs11238956	10	44,749,854	C	T	0.315	0.351	6.62E-06	0.849	0.036	0.791	0.912	0.973	Genotyped
rs510785	10	44,749,990	A	C	0.160	0.140	7.23E-04	1.170	0.047	1.068	1.282	0.895	Imputed
rs607609	10	44,750,209	C	T	0.154	0.134	7.73E-04	1.172	0.047	1.068	1.286	0.884	Imputed
rs607592	10	44,750,215	G	A	0.154	0.134	7.73E-04	1.172	0.047	1.068	1.286	0.884	Imputed
rs2576355	10	44,750,362	G	A	0.154	0.134	9.23E-04	1.169	0.047	1.066	1.283	0.827	Imputed
rs2576354	10	44,750,393	A	G	0.146	0.130	5.19E-03	1.144	0.048	1.041	1.258	0.810	Imputed
chr10:44750411:D	10	44,750,411	C	CA	0.147	0.130	4.65E-03	1.146	0.048	1.043	1.259	0.829	Imputed
rs606700	10	44,750,412	G	A	0.148	0.131	3.71E-03	1.150	0.048	1.046	1.263	0.789	Imputed
chr10:44750414:I	10	44,750,414	GT	G	0.155	0.136	1.59E-03	1.160	0.047	1.058	1.273	0.910	Imputed
rs485838	10	44,750,414	T	G	0.147	0.130	3.56E-03	1.151	0.048	1.047	1.265	0.760	Imputed
rs606314	10	44,750,479	G	C	0.111	0.100	0.037	1.119	0.054	1.007	1.245	0.455	Imputed
rs605425	10	44,750,669	C	T	0.156	0.136	1.15E-03	1.165	0.047	1.063	1.278	0.869	Imputed
rs687175	10	44,751,910	C	T	0.152	0.132	9.02E-04	1.171	0.048	1.067	1.285	0.938	Imputed
rs559580	10	44,752,078	C	T	0.150	0.131	1.99E-03	1.159	0.048	1.055	1.273	0.797	Imputed
rs559469	10	44,752,118	C	T	0.152	0.132	9.02E-04	1.171	0.048	1.067	1.285	0.938	Imputed
rs2437935	10	44,752,268	G	A	0.362	0.346	0.060	1.069	0.035	0.997	1.145	0.631	Imputed
rs10900012	10	44,752,319	C	G	0.009	0.012	0.066	0.727	0.175	0.516	1.023	0.903	Imputed
rs535949	10	44,752,330	T	G	0.152	0.132	9.02E-04	1.171	0.048	1.067	1.285	0.938	Imputed
rs684521	10	44,752,523	A	G	0.152	0.132	1.01E-03	1.169	0.048	1.065	1.283	0.903	Imputed
rs534079	10	44,752,558	T	G	0.152	0.132	9.20E-04	1.170	0.048	1.066	1.284	0.940	Imputed
rs17155842	10	44,752,633	T	C	0.017	0.013	0.019	1.364	0.133	1.051	1.770	0.286	Imputed
rs78647284	10	44,752,867	C	T	0.042	0.039	0.732	1.030	0.085	0.871	1.217	0.934	Imputed
rs671765	10	44,752,976	G	A	0.152	0.132	9.20E-04	1.170	0.048	1.066	1.284	0.940	Imputed
rs670056	10	44,753,375	T	C	0.152	0.132	9.20E-04	1.170	0.048	1.066	1.284	0.940	Imputed
rs504799	10	44,753,456	T	C	0.152	0.132	9.20E-04	1.170	0.048	1.066	1.284	0.940	Imputed
rs503859	10	44,753,560	C	G	0.152	0.132	9.20E-04	1.170	0.048	1.066	1.284	0.940	Imputed
rs501120	10	44,753,867	C	T	0.152	0.132	9.96E-04	1.169	0.047	1.065	1.283	0.882	Genotyped
rs656779	10	44,754,032	A	G	0.028	0.025	0.269	1.121	0.103	0.916	1.373	0.635	Imputed
rs471451	10	44,754,785	C	G	0.090	0.084	0.227	1.074	0.059	0.956	1.207	0.569	Imputed
rs579058	10	44,755,104	G	A	0.152	0.132	9.20E-04	1.170	0.048	1.066	1.284	0.940	Imputed
rs622956	10	44,755,183	G	A	0.152	0.132	9.20E-04	1.170	0.048	1.066	1.284	0.940	Imputed
rs915083	10	44,755,406	G	T	0.152	0.132	1.06E-03	1.168	0.048	1.064	1.282	0.956	Imputed
rs554568	10	44,755,446	T	C	0.152	0.132	9.02E-04	1.171	0.048	1.067	1.285	0.938	Imputed
rs554565	10	44,755,448	A	G	0.152	0.132	9.02E-04	1.171	0.048	1.067	1.285	0.938	Imputed
rs57454918	10	44,755,924	T	C	0.013	0.014	0.717	0.948	0.147	0.711	1.264	0.671	Imputed

rs607760	10	44,756,236	G	A	0.150	0.132	1.59E-03	1.162	0.048	1.059	1.276	0.928	Imputed
rs523297	10	44,756,557	T	C	0.110	0.098	0.037	1.120	0.054	1.006	1.246	0.261	Imputed
rs605956	10	44,756,648	G	A	0.324	0.305	0.012	1.095	0.036	1.020	1.176	0.600	Imputed
rs522293	10	44,756,703	A	G	0.152	0.132	1.12E-03	1.167	0.048	1.064	1.281	0.985	Imputed
rs605445	10	44,756,779	T	C	0.152	0.132	1.12E-03	1.167	0.048	1.064	1.281	0.985	Imputed
rs75036392	10	44,756,834	C	G	0.034	0.034	0.842	0.982	0.093	0.818	1.179	0.891	Imputed
rs145671441	10	44,756,850	T	C	0.015	0.015	0.665	1.061	0.138	0.810	1.391	0.837	Imputed
rs604674	10	44,756,894	T	G	0.152	0.132	1.12E-03	1.167	0.048	1.064	1.281	0.985	Imputed
rs518594	10	44,757,107	C	T	0.151	0.132	1.16E-03	1.167	0.048	1.063	1.281	0.938	Imputed
rs187207148	10	44,757,502	A	C	0.012	0.010	0.136	1.260	0.156	0.928	1.711	0.250	Imputed
rs492152	10	44,757,677	T	C	0.152	0.131	7.37E-04	1.174	0.048	1.069	1.289	0.886	Imputed
rs665855	10	44,757,751	C	T	0.152	0.131	7.37E-04	1.174	0.048	1.069	1.289	0.886	Imputed
rs78406026	10	44,758,128	A	C	0.035	0.029	0.039	1.214	0.094	1.010	1.459	0.064	Imputed
rs487465	10	44,758,197	C	A	0.151	0.131	8.25E-04	1.173	0.048	1.068	1.287	0.828	Imputed
rs71494723	10	44,758,343	A	G	0.015	0.016	0.915	0.985	0.138	0.751	1.292	0.436	Imputed
rs573141	10	44,758,587	A	G	0.151	0.131	9.10E-04	1.171	0.048	1.067	1.286	0.839	Imputed
rs112107106	10	44,759,020	T	C	0.035	0.029	0.039	1.214	0.094	1.010	1.459	0.064	Imputed
rs78938385	10	44,759,192	C	T	0.044	0.038	0.076	1.161	0.084	0.985	1.369	0.042	Imputed
rs75046680	10	44,759,215	G	A	0.028	0.030	0.350	0.909	0.102	0.744	1.110	0.300	Imputed
rs634963	10	44,760,033	C	T	0.151	0.131	9.47E-04	1.171	0.048	1.066	1.285	0.777	Imputed
rs479596	10	44,760,473	T	C	0.140	0.123	3.49E-03	1.154	0.049	1.048	1.271	0.950	Imputed
rs622725	10	44,760,494	C	G	0.099	0.091	0.096	1.099	0.057	0.983	1.228	0.188	Imputed
rs475926	10	44,760,887	G	T	0.323	0.303	0.010	1.098	0.036	1.022	1.179	0.708	Imputed
rs620828	10	44,760,958	G	A	0.151	0.131	9.47E-04	1.171	0.048	1.066	1.285	0.777	Imputed
rs620356	10	44,761,073	C	A	0.151	0.131	9.47E-04	1.171	0.048	1.066	1.285	0.777	Imputed
rs73286667	10	44,761,477	T	C	0.017	0.013	0.019	1.364	0.133	1.051	1.770	0.286	Imputed
rs607363	10	44,761,623	G	A	0.150	0.131	1.41E-03	1.165	0.048	1.060	1.279	0.776	Genotyped
rs694425	10	44,761,804	G	C	0.150	0.131	1.30E-03	1.166	0.048	1.062	1.281	0.765	Imputed
rs528668	10	44,762,584	A	G	0.151	0.131	9.28E-04	1.171	0.048	1.066	1.286	0.774	Imputed
rs527785	10	44,762,686	A	G	0.151	0.131	9.28E-04	1.171	0.048	1.066	1.286	0.774	Imputed
rs620205	10	44,762,791	G	A	0.151	0.131	9.28E-04	1.171	0.048	1.066	1.286	0.774	Imputed
rs111675425	10	44,763,066	A	G	0.035	0.029	0.039	1.214	0.094	1.010	1.459	0.064	Imputed
rs7897860	10	44,763,520	A	C	0.173	0.172	0.867	1.008	0.045	0.923	1.100	0.489	Imputed
rs495627	10	44,763,914	C	T	0.151	0.132	9.98E-04	1.169	0.048	1.065	1.284	0.832	Imputed
rs4515919	10	44,764,014	T	C	0.173	0.172	0.867	1.008	0.045	0.923	1.100	0.489	Imputed
rs493874	10	44,764,077	T	C	0.151	0.131	9.15E-04	1.171	0.048	1.067	1.286	0.773	Imputed
rs192983311	10	44,764,198	A	G	0.035	0.029	0.039	1.214	0.094	1.010	1.459	0.064	Imputed
rs640577	10	44,764,219	C	T	0.154	0.134	9.33E-04	1.169	0.047	1.066	1.283	0.983	Imputed
rs12415357	10	44,764,540	G	C	0.013	0.011	0.261	1.187	0.153	0.880	1.600	0.937	Imputed
rs627135	10	44,765,000	G	T	0.105	0.096	0.088	1.099	0.055	0.986	1.225	0.114	Imputed

rs11238961	10	44,765,095	G	C	0.142	0.125	3.45E-03	1.153	0.049	1.048	1.269	0.595	Imputed
rs11238962	10	44,765,126	T	C	0.141	0.124	3.48E-03	1.154	0.049	1.048	1.270	0.878	Imputed
rs11524366	10	44,765,163	C	G	0.124	0.111	0.022	1.125	0.052	1.017	1.246	0.677	Imputed
chr10:44765350:D	10	44,765,350	T	TC	0.036	0.037	0.817	0.980	0.090	0.821	1.168	0.147	Imputed
rs9702116	10	44,765,640	C	A	0.125	0.111	0.013	1.137	0.052	1.027	1.258	0.874	Imputed
rs11517224	10	44,765,751	G	A	0.139	0.124	6.41E-03	1.143	0.049	1.038	1.259	0.925	Imputed
rs181505060	10	44,765,830	A	G	0.035	0.029	0.039	1.214	0.094	1.010	1.459	0.064	Imputed
rs9703833	10	44,765,933	T	C	0.314	0.296	0.021	1.088	0.037	1.013	1.168	0.576	Imputed
rs9703834	10	44,765,962	G	C	0.111	0.099	0.031	1.124	0.054	1.010	1.250	0.275	Imputed
rs80066128	10	44,766,178	A	G	0.098	0.090	0.122	1.092	0.057	0.977	1.221	0.219	Imputed
rs145647795	10	44,766,354	G	T	0.131	0.115	4.50E-03	1.154	0.050	1.045	1.274	0.653	Imputed
rs141429003	10	44,766,508	A	C	0.060	0.067	0.122	0.896	0.071	0.780	1.030	0.976	Imputed
rs192163972	10	44,767,229	G	A	0.285	0.271	0.084	1.067	0.038	0.991	1.149	0.763	Imputed
rs145086163	10	44,767,325	C	T	0.131	0.113	1.49E-03	1.174	0.051	1.063	1.297	0.909	Imputed
chr10:44767726:D	10	44,767,726	A	ATAAAT	0.013	0.010	0.079	1.302	0.151	0.968	1.750	0.233	Imputed
rs2917636	10	44,768,538	A	C	0.094	0.087	0.174	1.082	0.058	0.966	1.212	0.199	Imputed
rs149415784	10	44,768,597	T	C	0.039	0.031	8.86E-03	1.262	0.089	1.061	1.502	0.046	Imputed
rs189467428	10	44,769,964	C	T	0.017	0.014	0.131	1.219	0.131	0.942	1.576	0.822	Imputed
rs642222	10	44,770,473	G	A	0.151	0.131	9.95E-04	1.170	0.048	1.065	1.284	0.803	Imputed
rs148016611	10	44,770,731	T	C	0.174	0.173	0.826	1.010	0.045	0.925	1.102	0.469	Imputed
rs12570878	10	44,771,056	A	G	0.174	0.174	0.940	1.003	0.045	0.919	1.095	0.495	Imputed
rs149398475	10	44,771,559	T	C	0.107	0.097	0.058	1.110	0.055	0.996	1.236	0.154	Imputed
rs552794	10	44,771,666	A	G	0.151	0.131	1.05E-03	1.169	0.048	1.065	1.283	0.789	Imputed
rs11238970	10	44,772,821	C	T	0.130	0.123	0.272	1.057	0.051	0.958	1.167	0.208	Imputed
rs2437936	10	44,772,894	C	G	0.146	0.128	2.29E-03	1.158	0.048	1.054	1.273	0.918	Imputed
rs3123689	10	44,773,121	A	G	0.151	0.131	1.05E-03	1.169	0.048	1.065	1.283	0.789	Imputed
rs494192	10	44,773,446	C	A	0.148	0.130	1.90E-03	1.161	0.048	1.056	1.275	0.932	Imputed
rs494045	10	44,773,498	T	C	0.146	0.128	2.90E-03	1.155	0.048	1.050	1.269	0.970	Imputed
rs111613307	10	44,773,666	A	G	0.035	0.029	0.043	1.210	0.094	1.006	1.455	0.087	Imputed
rs1746046	10	44,773,956	C	T	0.322	0.303	0.013	1.095	0.036	1.020	1.175	0.698	Imputed
rs1632484	10	44,773,984	T	C	0.147	0.129	2.66E-03	1.156	0.048	1.051	1.270	0.875	Genotyped
rs1746047	10	44,774,086	G	T	0.150	0.131	1.15E-03	1.168	0.048	1.063	1.282	0.820	Imputed
chr10:44774291:I	10	44,774,291	CAG	C	0.150	0.131	1.17E-03	1.167	0.048	1.063	1.282	0.822	Imputed
chr10:44774293:I	10	44,774,293	GA	G	0.150	0.131	1.21E-03	1.167	0.048	1.063	1.281	0.825	Imputed
chr10:44774558:D	10	44,774,558	G	GCT	0.143	0.125	1.69E-03	1.165	0.049	1.059	1.282	0.485	Imputed
chr10:44774561:D	10	44,774,561	C	CCTG	0.149	0.129	8.19E-04	1.173	0.048	1.068	1.289	0.293	Imputed
rs79962386	10	44,774,733	T	C	0.018	0.023	0.089	0.808	0.125	0.632	1.033	0.668	Imputed
rs117996904	10	44,774,870	G	A	0.027	0.027	0.918	0.989	0.105	0.806	1.214	0.448	Imputed
rs117726283	10	44,775,377	A	G	0.010	0.012	0.252	0.828	0.166	0.598	1.146	0.148	Imputed
rs1746048	10	44,775,824	T	C	0.150	0.131	1.42E-03	1.164	0.048	1.060	1.279	0.865	Imputed

rs112266201	10	44,776,108	C	T	0.035	0.029	0.043	1.210	0.094	1.006	1.455	0.087	Imputed
rs1746049	10	44,776,310	T	C	0.147	0.129	2.29E-03	1.158	0.048	1.054	1.273	0.989	Imputed
rs1746050	10	44,777,188	A	C	0.147	0.129	2.29E-03	1.158	0.048	1.054	1.273	0.989	Imputed
rs1657346	10	44,777,560	C	G	0.106	0.096	0.058	1.110	0.055	0.996	1.237	0.195	Imputed
rs1746051	10	44,778,124	C	T	0.035	0.040	0.127	0.872	0.091	0.730	1.041	0.054	Imputed
rs12784106	10	44,778,490	T	C	0.025	0.032	0.034	0.797	0.107	0.646	0.983	0.277	Imputed
rs1746052	10	44,778,546	C	A	0.146	0.128	2.14E-03	1.160	0.048	1.055	1.275	0.577	Imputed
rs111537293	10	44,778,827	A	T	0.034	0.029	0.048	1.206	0.094	1.002	1.451	0.083	Imputed
rs1657345	10	44,779,078	G	A	0.142	0.125	4.09E-03	1.150	0.049	1.045	1.266	0.724	Imputed
rs1657344	10	44,781,234	T	C	0.135	0.119	5.01E-03	1.150	0.050	1.043	1.268	0.982	Imputed
rs11238978	10	44,781,343	A	C	0.182	0.176	0.262	1.051	0.044	0.964	1.145	0.231	Imputed
rs116884438	10	44,781,374	C	T	0.013	0.016	0.158	0.813	0.148	0.609	1.086	0.105	Imputed
rs1746053	10	44,781,506	A	C	0.023	0.027	0.126	0.844	0.111	0.679	1.050	0.146	Imputed
rs61856370	10	44,782,297	A	G	0.033	0.032	0.748	1.031	0.095	0.856	1.241	0.236	Imputed
rs920172	10	44,782,724	C	T	0.106	0.090	9.40E-04	1.202	0.056	1.078	1.340	0.441	Imputed
rs117250281	10	44,782,790	C	G	0.026	0.027	0.775	0.970	0.106	0.788	1.194	0.505	Imputed
rs112311610	10	44,785,548	C	T	0.030	0.024	0.017	1.274	0.101	1.045	1.553	0.023	Imputed
rs812331	10	44,786,201	C	T	0.023	0.027	0.126	0.844	0.111	0.678	1.050	0.216	Imputed
rs800314	10	44,786,364	G	A	0.075	0.066	0.020	1.162	0.065	1.024	1.319	0.717	Genotyped
rs800315	10	44,786,833	T	C	0.075	0.065	0.024	1.158	0.065	1.019	1.315	0.678	Imputed
rs144960850	10	44,786,863	T	C	0.026	0.032	0.047	0.812	0.105	0.661	0.997	0.245	Imputed
rs61856371	10	44,787,259	A	G	0.031	0.031	0.890	0.987	0.097	0.816	1.194	0.140	Imputed
rs2760671	10	44,787,629	G	A	0.023	0.027	0.134	0.847	0.111	0.681	1.053	0.210	Imputed
chr10:44788266:D	10	44,788,266	A	AC	0.036	0.028	4.86E-03	1.300	0.093	1.083	1.560	0.447	Imputed
chr10:44788485:D	10	44,788,485	AG	A	0.157	0.150	0.198	1.062	0.047	0.969	1.164	0.719	Imputed
rs58189594	10	44,788,826	T	C	0.182	0.180	0.654	1.020	0.044	0.936	1.111	0.347	Imputed
rs75327503	10	44,789,191	C	G	0.032	0.025	0.014	1.273	0.098	1.050	1.543	0.058	Imputed
chr10:44789412:D	10	44,789,412	C	CTG	0.030	0.027	0.210	1.133	0.100	0.932	1.378	0.708	Imputed
rs72788791	10	44,789,554	A	G	0.181	0.179	0.698	1.017	0.044	0.933	1.109	0.360	Imputed
rs809601	10	44,789,602	G	A	0.024	0.028	0.153	0.855	0.110	0.690	1.061	0.221	Genotyped
rs74357308	10	44,789,700	A	T	0.016	0.019	0.131	0.814	0.136	0.624	1.062	0.112	Imputed
rs808972	10	44,789,989	T	A	0.023	0.027	0.126	0.844	0.111	0.678	1.050	0.199	Imputed
rs57055606	10	44,790,341	T	C	0.031	0.024	7.32E-03	1.306	0.099	1.075	1.586	0.037	Imputed
rs79405863	10	44,790,554	G	A	0.030	0.024	0.015	1.280	0.101	1.051	1.559	0.025	Imputed
rs11598523	10	44,791,433	A	T	0.182	0.180	0.668	1.019	0.044	0.935	1.111	0.341	Imputed
