# Supplementary material for: Genome-Wide Association Study Link Novel Loci to Endometriosis
Source: PLoS One. 2013 Mar 5;8(3):e58257. doi: 10.1371/journal.pone.0058257 (PMC3589333; doi:10.1371/journal.pone.0058257)
Supplement: Table S3 — Haplotype analysis for the WNT4-region. (PDF) [file pone.0058257.s007.pdf]

Table S3 Haplotype analysis of key associated SNPs from the WNT4 region shows evidence of the causal signal.

SNP	Position	MAF_{case}	$MAF_{control}$	HAP-1*	HAP-2	HAP-3
rs10917151[i]	22,422,721	0.193	0.156	A	G	G
rs4654783[g]	22,439,520	0.336	0.295	T	T	C
rs2235529[g]	22,450,487	0.186	0.151	T	C	C
			HAF_{case}	0.187	0.139	0.674
			$HAF_{controls}$	0.152	0.139	0.709
			P-value	7.26E-09	0.912	6.26E-06
			OR	1.28	0.99	0.84

SNP	Position	MAF_{case}	$MAF_{control}$	HAP-1a*	HAP-1b*	HAP-2a	HAP-2b	HAP-3a	HAP-3b	HAP-3c
rs10917151[i]	22,422,721	0.193	0.156	A	A	G	G	G	G	G
rs4654783[g]	22,439,520	0.336	0.295	T	T	T	T	C	C	C
rs2235529[g]	22,450,487	0.186	0.151	T	T	C	C	C	C	C
rs16826658[i]	22,485,871	0.412	0.396	G	T	T	G	G	T	G
rs7521902[i]	22,490,724	0.260	0.232	A	C	C	C	A	C	C
			HAF_{case}	0.163	0.023	0.124	0.010	0.089	0.450	0.142
			$HAF_{controls}$	0.134	0.016	0.123	0.012	0.090	0.473	0.152
			P-value	8.33E-07	0.001	0.899	0.311	0.752	0.006	0.107
			OR	1.25	1.53	1.00	0.73	0.98	0.90	0.92

The Table show the haplotypes for the key markers in the WNT4 region. The upper half show three SNPs that together define three haplotypes. The risk-haplotype, HAP-1, correlate perfectly with the minor alleles of rs10917151 and rs2235529. The haplotypes presented in the lower half also include rs16826658 identified by Uno et al. (2010) and rs7521902 identified by Painter et al. (2011) and together the five markers define seven haplotypes. The original risk haplotype, HAP-1, dissolve in the lower panel into two haplotypes, HAP-1a and HAP-1b, that both carry risk. The haplotype analysis provide evidence that recombination has separated rs16826658 and rs7521902 from the risk locus. HAF = haplotype frequency. P values are calculated by 1 degree of freedom $\chi 2$-test. Odds-ratios (OR) are calculated using the non-reference haplotype. * = The risk Haplotype. i = imputed SNP. g = genotyped SNP.
