# Supplementary material for: Genome-Wide Association Study Link Novel Loci to Endometriosis
Source: PLoS One. 2013 Mar 5;8(3):e58257. doi: 10.1371/journal.pone.0058257 (PMC3589333; doi:10.1371/journal.pone.0058257)
Supplement: Table S4 — a No association with CDKN2BAS on 9p21 in Europeans. b Tentative replication of rs13271465 located on 8p22 between MTMR7 and SLC7A2. c SNP rs12700667 fails replication. (PDF) [file pone.0058257.s008.pdf]

Table S4a CDKN2BAS show no evidence of association with endometriosis in a Caucasian population.

Name	Chr	Pos	OR_allelic	P_trend	Geno_Aff	Geno_UnAff	F_A	F_U	HWDaff	HWDunaff	HWDall	GeneSymbol	GeneLocation
rs3218005	9	22,000,247	0.98	0.753	22/355/1639	148/2613/11705	0.099	0.101	0.533	0.854	0.730	CDKN2BAS | CDKN2B	INTERGENIC
rs3217992	9	22,003,223	0.99	0.709	277/956/774	2086/6793/5578	0.376	0.379	0.536	0.805	1.000	CDKN2B	UTR
rs1063192	9	22,003,367	1.02	0.600	382/985/643	2643/7138/4642	0.435	0.431	0.892	0.277	0.332	CDKN2B	UTR
rs3217986	9	22,005,330	1.01	0.817	15/325/1678	102/2310/12059	0.088	0.087	1.000	0.496	0.492	CDKN2B	UTR
rs13298881	9	22,012,051	0.92	0.130	22/364/1625	192/2773/11467	0.101	0.109	0.714	0.104	0.098	CDKN2B | CDKN2BAS	INTERGENIC
rs545226	9	22,012,422	0.96	0.258	315/955/709	2444/6853/5025	0.401	0.410	0.852	0.195	0.250	CDKN2B | CDKN2BAS	INTERGENIC
rs7049105	9	22,028,801	0.99	0.655	464/991/556	3337/7211/3889	0.477	0.481	0.592	0.973	0.888	CDKN2B | CDKN2BAS	INTERGENIC
rs564398	9	22,029,547	1.03	0.465	352/979/686	2411/7068/4980	0.417	0.411	0.927	0.257	0.303	CDKN2BAS	UTR
rs1333035	9	22,044,059	0.98	0.684	27/382/1610	171/2844/11452	0.108	0.110	0.418	0.734	0.968	CDKN2BAS	INTRON
rs1333034	9	22,044,122	0.98	0.769	23/360/1630	140/2672/11610	0.101	0.102	0.538	0.341	0.496	CDKN2BAS	INTRON
rs11790231	9	22,053,591	0.94	0.282	19/379/1619	184/2783/11489	0.103	0.109	0.631	0.285	0.420	CDKN2BAS	INTRON
rs10120688	9	22,056,499	0.99	0.671	489/991/538	3506/7208/3754	0.488	0.491	0.449	0.690	0.523	CDKN2BAS	INTRON
rs1011970	9	22,062,134	0.99	0.789	50/567/1379	373/4101/9885	0.167	0.169	0.421	0.032	0.021	CDKN2BAS	INTRON
rs10811650	9	22,067,593	0.99	0.721	361/999/658	2676/7071/4719	0.426	0.429	0.617	0.760	0.924	CDKN2BAS	INTRON
rs4977756	9	22,068,652	1.02	0.672	327/950/739	2233/6943/5290	0.398	0.394	0.457	0.578	0.794	CDKN2BAS	INTRON
rs9632885	9	22,072,638	0.98	0.496	464/1006/540	3437/7203/3818	0.481	0.487	0.929	0.727	0.779	CDKN2BAS	INTRON
rs10757270	9	22,072,719	0.98	0.582	360/995/655	2701/7014/4682	0.427	0.431	0.616	0.415	0.567	CDKN2BAS	INTRON
rs1412832	9	22,077,543	1.03	0.488	201/843/963	1344/6075/6957	0.310	0.305	0.404	0.738	0.543	CDKN2BAS	INTRON
rs1547705	9	22,082,375	0.94	0.235	28/425/1565	245/3149/11072	0.119	0.126	1.000	0.226	0.270	CDKN2BAS	INTRON
rs1333040	9	22,083,404	1.04	0.264	355/985/678	2469/6946/5052	0.420	0.411	0.964	0.328	0.377	CDKN2BAS	INTRON
rs1537370	9	22,084,310	1.00	0.924	479/1003/524	3454/7251/3755	0.489	0.490	1.000	0.702	0.708	CDKN2BAS	INTRON
rs10757272	9	22,088,260	0.98	0.462	477/1006/534	3504/7230/3734	0.486	0.492	0.964	0.974	0.950	CDKN2BAS	INTRON
rs10757274	9	22,096,055	0.97	0.335	477/1008/531	3533/7226/3686	0.487	0.495	1.000	0.947	0.950	CDKN2BAS	INTRON
rs4977574	9	22,098,574	0.97	0.370	478/1008/531	3541/7219/3703	0.487	0.494	1.000	0.855	0.864	CDKN2BAS	INTRON
rs1333042	9	22,103,813	1.01	0.704	499/996/521	3503/7204/3753	0.495	0.491	0.593	0.690	0.575	CDKN2BAS	INTRON
rs10738609	9	22,114,495	1.03	0.445	518/992/502	3575/7240/3646	0.504	0.498	0.533	0.881	0.950	CDKN2BAS	INTRON
rs2383206	9	22,115,026	1.01	0.769	487/975/557	3345/7206/3918	0.483	0.480	0.141	0.777	0.435	CDKN2BAS	INTRON
rs2383207	9	22,115,959	1.01	0.825	477/975/562	3296/7205/3960	0.479	0.477	0.181	0.868	0.532	CDKN2BAS	INTRON
rs10757281	9	22,127,613	1.06	0.230	36/459/1458	207/3271/10799	0.136	0.129	1.000	0.023	0.034	CDKN2BAS | LOC729983	INTERGENIC
rs10811658	9	22,128,600	1.02	0.583	166/877/969	1270/6027/7163	0.300	0.296	0.101	0.968	0.601	CDKN2BAS | LOC729983	INTERGENIC
rs10965243	9	22,130,065	1.00	0.982	18/329/1669	118/2382/11943	0.091	0.091	0.684	1.000	0.925	CDKN2BAS | LOC729983	INTERGENIC
rs7045889	9	22,133,251	0.96	0.197	315/976/723	2381/7070/5000	0.399	0.409	0.642	0.164	0.142	CDKN2BAS | LOC729983	INTERGENIC
rs10217762	9	22,133,645	1.01	0.825	368/1003/648	2637/7130/4696	0.431	0.429	0.586	0.455	0.365	CDKN2BAS | LOC729983	INTERGENIC
rs10811659	9	22,133,716	1.01	0.789	97/722/1197	775/4969/8723	0.227	0.225	0.410	0.054	0.135	CDKN2BAS | LOC729983	INTERGENIC
rs10757282	9	22,133,984	1.01	0.814	369/1001/647	2638/7131/4687	0.431	0.429	0.618	0.425	0.348	CDKN2BAS | LOC729983	INTERGENIC
rs10757283	9	22,134,172	1.01	0.686	371/1001/642	2629/7135/4668	0.433	0.429	0.618	0.292	0.239	CDKN2BAS | LOC729983	INTERGENIC
rs7018475	9	22,137,685	1.04	0.358	142/827/1050	1033/5686/7728	0.275	0.268	0.241	0.783	0.501	CDKN2BAS | LOC729983	INTERGENIC
rs4977761	9	22,138,762	0.98	0.608	228/915/867	1728/6532/6209	0.341	0.345	0.585	0.883	0.973	CDKN2BAS | LOC729983	INTERGENIC
rs2065501	9	22,140,224	1.04	0.276	207/922/888	1518/6285/6643	0.331	0.323	0.160	0.581	0.986	CDKN2BAS | LOC729983	INTERGENIC
rs7854629	9	22,141,034	0.94	0.085	341/983/695	2706/6931/4829	0.412	0.427	0.854	0.013	0.024	CDKN2BAS | LOC729983	INTERGENIC
rs7026735	9	22,141,269	1.08	0.031	228/870/921	1400/6210/6850	0.328	0.312	0.313	0.907	0.800	CDKN2BAS | LOC729983	INTERGENIC
rs2065504	9	22,141,552	0.97	0.440	481/1010/527	3551/7227/3693	0.489	0.495	0.965	0.894	0.926	CDKN2BAS | LOC729983	INTERGENIC
rs2065505	9	22,141,790	0.93	0.058	227/901/890	1818/6509/6129	0.336	0.351	1.000	0.165	0.193	CDKN2BAS | LOC729983	INTERGENIC
rs10811664	9	22,142,907	1.02	0.619	61/512/1404	366/3748/10129	0.160	0.157	0.094	0.393	0.156	CDKN2BAS | LOC729983	INTERGENIC
rs2065500	9	22,145,694	1.01	0.820	62/537/1420	384/3925/10150	0.164	0.162	0.194	0.854	0.529	CDKN2BAS | LOC729983	INTERGENIC
rs944802	9	22,155,709	1.07	0.190	36/432/1549	218/2973/11276	0.125	0.118	0.359	0.174	0.109	LOC729983	UTR
rs1333052	9	22,157,908	1.06	0.110	250/904/863	1615/6461/6374	0.348	0.335	0.589	0.723	0.903	LOC729983 | DMRTA1	INTERGENIC
rs10757288	9	22,159,416	0.92	0.011	396/967/648	3041/7128/4233	0.437	0.459	0.319	0.700	0.450	LOC729983 | DMRTA1	INTERGENIC
rs10811667	9	22,159,982	0.92	0.017	351/942/726	2622/7112/4735	0.407	0.427	0.140	0.598	0.975	LOC729983 | DMRTA1	INTERGENIC
rs10965267	9	22,161,828	1.01	0.824	9/315/1688	118/2124/12196	0.083	0.082	0.187	0.020	0.087	LOC729983 | DMRTA1	INTERGENIC

rs10965269	9	22,162,061	1.03	0.503	54/500/1454	314/3620/10477	0.151	0.147	0.165	0.947	0.577	LOC729983 | DMRTA1	INTERGENIC
rs17694933	9	22,164,309	1.06	0.115	384/987/645	2572/7059/4822	0.435	0.422	0.856	0.905	0.975	LOC729983 | DMRTA1	INTERGENIC
rs10811668	9	22,164,991	1.05	0.277	102/649/1244	622/4715/9103	0.214	0.206	0.162	0.722	0.393	LOC729983 | DMRTA1	INTERGENIC
rs2779748	9	22,166,769	0.93	0.048	337/935/714	2519/7073/4770	0.405	0.422	0.306	0.245	0.480	LOC729983 | DMRTA1	INTERGENIC
rs7863846	9	22,168,128	1.06	0.090	226/871/880	1438/6237/6513	0.335	0.321	0.649	0.336	0.472	LOC729983 | DMRTA1	INTERGENIC
rs10757292	9	22,176,961	1.12	0.119	4/219/1792	42/1377/13032	0.056	0.051	0.405	0.385	0.631	LOC729983 | DMRTA1	INTERGENIC
rs4977764	9	22,184,674	1.12	0.161	2/181/1830	14/1165/13287	0.046	0.041	0.438	0.026	0.011	LOC729983 | DMRTA1	INTERGENIC
rs2767409	9	22,184,997	1.02	0.671	289/954/730	2035/6874/5314	0.388	0.385	0.448	0.013	0.009	LOC729983 | DMRTA1	INTERGENIC
rs1095898	9	22,189,716	0.99	0.685	238/909/851	1793/6460/6104	0.347	0.350	0.882	0.193	0.249	LOC729983 | DMRTA1	INTERGENIC
rs1679029	9	22,195,321	0.93	0.053	272/928/798	2109/6823/5436	0.368	0.384	0.924	0.685	0.728	LOC729983 | DMRTA1	INTERGENIC
rs2219849	9	22,195,820	1.00	0.912	436/1017/556	3163/7190/4050	0.470	0.469	0.502	0.802	0.627	LOC729983 | DMRTA1	INTERGENIC
rs12350824	9	22,201,586	1.00	0.975	69/568/1381	441/4183/9837	0.175	0.175	0.280	0.908	0.788	LOC729983 | DMRTA1	INTERGENIC

Table S4B rs13271465 is suggestive of association with endometriosis in a Caucasian population

Name	Chr	Pos	OR_allelic	P_trend	Geno_Aff	Geno_UnAff	F_A	F_U	HWDaff	HWDunaff	HWDall	GeneSymbol	GeneLocation
rs2239865	8	17,182,541	1.09	0.017	171/846/994	1077/5861/7511	0.295	0.277	0.668	0.158	0.141	MTMR7	INTRON
rs2239868	8	17,182,965	1.08	0.131	30/465/1524	211/3098/11154	0.130	0.122	0.491	0.846	0.638	MTMR7	INTRON
rs2239869	8	17,183,714	1.04	0.446	23/411/1582	160/2844/11465	0.113	0.109	0.580	0.286	0.217	MTMR7	INTRON
rs10453161	8	17,187,235	0.99	0.832	467/1025/525	3443/7204/3807	0.486	0.487	0.449	0.765	0.988	MTMR7	INTRON
rs2269692	8	17,193,011	1.09	0.189	10/248/1761	49/1669/12752	0.066	0.061	0.718	0.514	0.686	MTMR7	INTRON
rs2269693	8	17,193,274	0.91	0.022	76/626/1317	656/4714/9095	0.193	0.208	0.886	0.151	0.154	MTMR7	INTRON
rs2410518	8	17,197,888	0.94	0.179	73/588/1356	511/4496/9453	0.182	0.191	0.369	0.419	0.667	MTMR7	INTRON
rs17124423	8	17,197,967	1.07	0.186	21/402/1596	148/2688/11625	0.110	0.103	0.496	0.621	0.477	MTMR7	INTRON
rs4921756	8	17,198,953	0.91	0.050	38/475/1506	327/3632/10509	0.137	0.148	0.925	0.531	0.514	MTMR7	CODING
rs7005753	8	17,200,048	0.99	0.863	189/866/955	1370/6237/6835	0.310	0.311	0.754	0.341	0.308	MTMR7	INTRON
rs739336	8	17,202,736	1.08	0.404	4/141/1872	13/968/13480	0.037	0.034	0.350	0.379	0.641	MTMR7	INTRON
rs739337	8	17,202,871	1.06	0.522	4/150/1860	19/1038/13413	0.039	0.037	0.547	0.908	1.000	MTMR7	INTRON
rs7011504	8	17,208,533	1.07	0.426	5/171/1842	24/1171/13265	0.045	0.042	0.598	0.837	1.000	MTMR7	INTRON
rs7386192	8	17,209,290	0.95	0.235	81/630/1308	600/4707/9159	0.196	0.204	0.622	0.898	0.981	MTMR7	INTRON
rs1024355	8	17,218,629	0.91	0.046	41/477/1494	346/3672/10442	0.139	0.151	0.709	0.284	0.244	MTMR7	CODING
rs7822451	8	17,222,410	1.08	0.299	6/204/1806	33/1372/13044	0.054	0.050	0.826	0.725	0.744	MTMR7	INTRON
rs11203846	8	17,222,590	0.96	0.337	82/622/1313	584/4657/9227	0.195	0.201	0.435	0.918	0.884	MTMR7	INTRON
rs4921763	8	17,226,856	0.91	0.048	40/472/1502	332/3642/10493	0.137	0.149	0.706	0.450	0.386	MTMR7	INTRON
rs10087746	8	17,236,268	1.01	0.825	22/388/1607	144/2775/11537	0.107	0.106	0.907	0.114	0.118	MTMR7	INTRON
rs10089145	8	17,237,608	1.03	0.511	29/482/1508	253/3254/10953	0.134	0.130	0.211	0.532	0.918	MTMR7	INTRON
rs7837826	8	17,240,780	1.10	0.264	5/144/1869	20/964/13483	0.038	0.035	0.212	0.533	0.299	MTMR7	INTRON
rs7823989	8	17,240,957	0.98	0.518	451/984/582	3238/7198/4020	0.468	0.473	0.371	0.881	0.650	MTMR7	INTRON
rs7828510	8	17,241,040	1.09	0.354	3/145/1870	20/960/13489	0.037	0.035	0.758	0.455	0.487	MTMR7	INTRON
rs7004626	8	17,243,260	1.03	0.616	14/416/1588	161/2786/11523	0.110	0.107	0.017	0.634	0.195	MTMR7	INTRON
rs4921768	8	17,244,051	1.01	0.782	419/989/611	2905/7209/4347	0.453	0.450	0.621	0.401	0.550	MTMR7	INTRON
rs2898461	8	17,244,248	1.00	0.975	393/970/656	2684/7222/4561	0.435	0.435	0.319	0.063	0.168	MTMR7	INTRON
rs1002810	8	17,257,720	0.94	0.060	241/884/889	1759/6706/5965	0.339	0.354	0.345	0.061	0.151	MTMR7	INTRON
rs2898463	8	17,258,798	1.03	0.574	22/417/1575	180/2858/11397	0.114	0.112	0.380	0.933	0.814	MTMR7	INTRON
rs2157640	8	17,258,906	1.03	0.392	513/993/512	3507/7249/3708	0.500	0.493	0.477	0.765	0.988	MTMR7	INTRON
rs11203852	8	17,261,568	0.95	0.142	308/928/780	2224/6974/5260	0.383	0.395	0.239	0.273	0.535	MTMR7	INTRON
rs12548066	8	17,266,143	0.93	0.044	188/816/1011	1370/6264/6822	0.296	0.311	0.219	0.222	0.477	MTMR7	INTRON
rs13254505	8	17,267,569	0.95	0.163	176/808/1035	1274/6074/7120	0.287	0.298	0.302	0.691	0.985	MTMR7	INTRON
rs9802064	8	17,268,961	0.97	0.373	288/923/806	2073/6814/5578	0.372	0.379	0.365	0.930	0.830	MTMR7	INTRON
rs1003299	8	17,269,633	0.97	0.433	465/990/563	3371/7207/3883	0.476	0.482	0.475	0.816	0.629	MTMR7	INTRON
rs2073695	8	17,271,136	1.01	0.900	11/306/1693	101/2139/12208	0.082	0.081	0.554	0.502	0.676	MTMR7 | SLC7A2	INTERGENIC

rs7817826	8	17,271,478	0.95	0.165	158/783/1078	1118/5943/7407	0.272	0.283	0.340	0.124	0.264	MTMR7 | SLC7A2	INTERGENIC
rs6981038	8	17,274,709	1.10	0.194	3/208/1808	24/1349/13092	0.053	0.048	0.370	0.085	0.036	MTMR7 | SLC7A2	INTERGENIC
rs13271465	8	17,282,411	1.14	0.006	53/542/1420	322/3531/10606	0.161	0.144	0.869	0.168	0.170	MTMR7 | SLC7A2	INTERGENIC
rs10503595	8	17,283,970	0.96	0.281	73/641/1304	602/4648/9216	0.195	0.202	0.670	0.606	0.753	MTMR7 | SLC7A2	INTERGENIC
rs17587917	8	17,284,486	1.10	0.160	9/229/1779	43/1528/12898	0.061	0.056	0.559	0.813	1.000	MTMR7 | SLC7A2	INTERGENIC
rs10086318	8	17,284,564	1.02	0.665	259/966/782	1933/6692/5791	0.370	0.366	0.151	1.000	0.614	MTMR7 | SLC7A2	INTERGENIC
rs7462219	8	17,287,780	0.98	0.661	117/796/1103	952/5572/7926	0.256	0.259	0.101	0.529	0.238	MTMR7 | SLC7A2	INTERGENIC
rs4921772	8	17,290,124	1.03	0.442	273/982/761	2011/6751/5690	0.379	0.373	0.130	0.901	0.677	MTMR7 | SLC7A2	INTERGENIC
rs7387693	8	17,290,628	1.02	0.510	178/909/929	1349/6220/6879	0.314	0.309	0.038	0.293	0.086	MTMR7 | SLC7A2	INTERGENIC
rs17587994	8	17,292,283	1.06	0.093	292/990/736	2066/6752/5644	0.390	0.376	0.175	0.524	0.908	MTMR7 | SLC7A2	INTERGENIC
rs11203855	8	17,292,466	1.13	0.017	34/409/1575	187/2685/11582	0.118	0.106	0.239	0.028	0.013	MTMR7 | SLC7A2	INTERGENIC
rs4921773	8	17,298,699	1.07	0.256	26/357/1636	118/2529/11805	0.101	0.096	0.220	0.178	0.447	MTMR7 | SLC7A2	INTERGENIC
rs4921774	8	17,298,825	1.06	0.108	249/966/796	1763/6598/6058	0.364	0.351	0.102	0.622	0.290	MTMR7 | SLC7A2	INTERGENIC
rs6986498	8	17,299,625	1.05	0.288	45/507/1464	271/3555/10618	0.148	0.142	0.860	0.194	0.251	MTMR7 | SLC7A2	INTERGENIC
rs2027922	8	17,299,950	1.04	0.336	113/747/1157	769/5232/8444	0.241	0.234	0.627	0.266	0.225	MTMR7 | SLC7A2	INTERGENIC
rs12546964	8	17,301,152	0.98	0.501	296/937/785	2107/6904/5453	0.379	0.384	0.539	0.308	0.459	MTMR7 | SLC7A2	INTERGENIC
rs1557591	8	17,303,403	1.04	0.288	151/813/1055	1060/5640/7766	0.276	0.268	0.781	0.409	0.514	MTMR7 | SLC7A2	INTERGENIC
rs17632013	8	17,303,717	1.01	0.849	8/248/1741	58/1769/12605	0.066	0.065	1.000	0.683	0.655	MTMR7 | SLC7A2	INTERGENIC
rs973986	8	17,309,151	1.04	0.419	37/502/1477	265/3465/10728	0.143	0.138	0.524	0.463	0.344	MTMR7 | SLC7A2	INTERGENIC
rs1012640	8	17,312,407	1.01	0.791	35/486/1495	263/3417/10777	0.138	0.136	0.575	0.698	0.574	MTMR7 | SLC7A2	INTERGENIC
rs4554505	8	17,314,613	0.99	0.847	489/969/556	3441/7144/3875	0.483	0.485	0.099	0.189	0.070	MTMR7 | SLC7A2	INTERGENIC
rs12543516	8	17,320,152	1.01	0.845	72/620/1326	515/4407/9537	0.189	0.188	1.000	0.828	0.858	MTMR7 | SLC7A2	INTERGENIC
rs2705044	8	17,322,180	1.00	0.971	40/415/1562	219/3107/11143	0.123	0.123	0.049	0.877	0.405	MTMR7 | SLC7A2	INTERGENIC
rs17632120	8	17,322,899	0.96	0.636	7/173/1826	27/1341/13068	0.047	0.048	0.200	0.276	0.551	MTMR7 | SLC7A2	INTERGENIC
rs2517105	8	17,325,343	1.07	0.099	149/781/1082	965/5472/8020	0.268	0.256	0.610	0.445	0.370	MTMR7 | SLC7A2	INTERGENIC
rs17632167	8	17,327,208	0.95	0.245	113/674/1229	770/5152/8535	0.223	0.231	0.108	0.852	0.725	MTMR7 | SLC7A2	INTERGENIC
rs2517111	8	17,328,731	1.03	0.491	66/583/1370	416/4164/9885	0.177	0.173	0.702	0.383	0.514	MTMR7 | SLC7A2	INTERGENIC
rs2588240	8	17,330,523	0.96	0.288	271/909/821	1993/6714/5705	0.363	0.371	0.439	0.803	0.616	MTMR7 | SLC7A2	INTERGENIC
rs2517120	8	17,331,692	1.00	0.999	452/979/560	3205/7151/3983	0.473	0.473	0.559	0.973	0.863	MTMR7 | SLC7A2	INTERGENIC
rs10448129	8	17,332,465	0.95	0.216	115/677/1222	791/5187/8487	0.225	0.234	0.110	0.982	0.601	MTMR7 | SLC7A2	INTERGENIC
rs2517124	8	17,333,462	1.11	0.061	24/376/1617	145/2481/11837	0.105	0.096	0.637	0.249	0.213	MTMR7 | SLC7A2	INTERGENIC
rs11985222	8	17,334,887	0.99	0.844	3/258/1742	68/1794/12599	0.066	0.067	0.043	0.640	0.851	MTMR7 | SLC7A2	INTERGENIC
rs2720524	8	17,341,027	1.08	0.201	18/283/1717	75/1972/12406	0.079	0.073	0.123	0.760	0.776	MTMR7 | SLC7A2	INTERGENIC
rs1007691	8	17,344,517	0.98	0.622	412/986/616	2998/7106/4342	0.449	0.454	0.653	0.365	0.307	MTMR7 | SLC7A2	INTERGENIC
rs2720546	8	17,354,086	1.02	0.630	39/446/1519	225/3240/10947	0.131	0.128	0.376	0.433	0.676	MTMR7 | SLC7A2	INTERGENIC
rs2517217	8	17,357,290	1.09	0.146	23/315/1664	94/2204/12052	0.090	0.083	0.075	0.585	0.879	MTMR7 | SLC7A2	INTERGENIC
rs4921552	8	17,357,438	0.93	0.030	227/913/874	1827/6636/5954	0.339	0.357	0.655	0.758	0.658	MTMR7 | SLC7A2	INTERGENIC
rs11203867	8	17,359,171	1.07	0.072	141/764/1112	865/5382/8195	0.259	0.246	0.524	0.654	0.851	MTMR7 | SLC7A2	INTERGENIC
rs2720540	8	17,360,318	1.07	0.099	78/626/1312	478/4342/9641	0.194	0.183	0.776	0.718	0.816	MTMR7 | SLC7A2	INTERGENIC
rs17632417	8	17,363,239	1.01	0.699	301/971/747	2175/6829/5462	0.390	0.386	0.640	0.599	0.743	MTMR7 | SLC7A2	INTERGENIC
rs17588888	8	17,363,941	1.06	0.279	21/402/1595	147/2726/11586	0.110	0.104	0.496	0.374	0.263	MTMR7 | SLC7A2	INTERGENIC
rs11203868	8	17,369,376	1.00	0.903	258/994/763	1965/6878/5618	0.375	0.374	0.020	0.055	0.009	MTMR7 | SLC7A2	INTERGENIC
rs17124738	8	17,371,287	0.94	0.608	0/71/1948	3/536/13917	0.018	0.019	1.000	0.496	0.387	MTMR7 | SLC7A2	INTERGENIC
rs17124742	8	17,372,560	1.06	0.373	8/278/1733	68/1860/12535	0.073	0.069	0.508	1.000	0.718	MTMR7 | SLC7A2	INTERGENIC
rs2188020	8	17,374,960	0.98	0.591	160/863/993	1264/6069/7114	0.293	0.298	0.162	0.564	0.296	MTMR7 | SLC7A2	INTERGENIC
rs2517237	8	17,375,959	1.00	0.980	72/657/1287	552/4626/9256	0.199	0.199	0.327	0.403	0.240	MTMR7 | SLC7A2	INTERGENIC
rs2188021	8	17,377,242	1.00	0.986	246/981/789	1863/6830/5760	0.365	0.365	0.031	0.022	0.003	MTMR7 | SLC7A2	INTERGENIC
rs13270915	8	17,377,307	0.93	0.343	2/189/1826	34/1417/13010	0.048	0.051	0.322	0.550	0.333	MTMR7 | SLC7A2	INTERGENIC
rs10110252	8	17,380,076	0.99	0.712	350/1009/646	2700/6997/4743	0.426	0.429	0.201	0.185	0.426	MTMR7 | SLC7A2	INTERGENIC
rs2720493	8	17,383,689	1.01	0.683	365/996/643	2679/6978/4780	0.431	0.427	0.554	0.139	0.238	MTMR7 | SLC7A2	INTERGENIC

Table S4c. Imputed rs12700667 and rs7798431 and SNPs in strong LD with rs12700667 and rs7798431 do not show support for association with endometriosis in a Caucasian population.

Name	Chr	Position	All affected genotypes	ModSev genotypes	Control genotypes	MAF Affected	MAF ModSev	MAF Controls	P Affected	OR Affected	P ModSev	OR ModSev	r^2 rs12700667	D' rs12700667	r^2 rs7798431	D' rs7798431
rs12537117	7	25,780,315	3/259/1754	2/113/727	60/1777/12607	0.066	0.069	0.066	0.989	1.00	0.539	1.06	0.06	0.63	0.09	0.69
rs17152544	7	25,789,508	211/853/955	74/373/396	1318/6004/7141	0.316	0.309	0.299	0.028	1.08	0.370	1.05	0.05	0.24	0.04	0.21
rs4722520	7	25,792,666	106/706/1206	36/303/504	684/4734/9050	0.228	0.222	0.211	0.017	1.10	0.263	1.07	0.03	0.20	0.01	0.09
rs17152613	7	25,798,864	127/758/1132	41/321/480	819/5074/8556	0.251	0.239	0.232	0.009	1.11	0.508	1.04	0.01	0.10	0.00	0.02
rs4141277	7	25,800,124	430/987/602	183/408/252	3235/7198/4029	0.457	0.459	0.473	0.071	0.94	0.282	0.95	0.14	0.67	0.10	0.64
rs9639517	7	25,801,760	180/859/979	60/369/413	1183/5871/7411	0.302	0.290	0.285	0.023	1.09	0.617	1.03	0.02	0.13	0.01	0.10
rs4722522	7	25,802,471	216/863/939	77/372/394	1313/6051/7087	0.321	0.312	0.300	0.008	1.10	0.307	1.06	0.04	0.22	0.03	0.19
rs10480044	7	25,807,078	5/206/1807	2/80/760	53/1448/12940	0.054	0.050	0.054	0.940	0.99	0.490	0.92	0.01	1	0.01	1
rs4719825	7	25,813,918	0/169/1846	0/75/766	27/1121/13314	0.042	0.045	0.041	0.693	1.03	0.425	1.10	0.00	0.02	0.01	0.36
rs4719826	7	25,814,109	0/168/1851	0/74/769	26/1111/13331	0.042	0.044	0.040	0.669	1.04	0.454	1.10	0.00	0.00	0.01	0.36
rs7350035	7	25,819,209	138/733/1148	71/303/469	1023/5521/7922	0.250	0.264	0.262	0.116	0.94	0.829	1.01	0.01	0.10	0.00	0.02
rs960589	7	25,823,647	59/574/1386	29/223/591	358/3784/10329	0.171	0.167	0.156	0.010	1.12	0.220	1.09	0.14	0.58	0.18	0.61
rs1376526	7	25,823,776	155/771/1093	77/319/447	1149/5805/7515	0.268	0.281	0.280	0.104	0.94	0.962	1.00	0.02	0.19	0.01	0.11
rs122505	7	25,827,799	137/728/1153	71/301/471	1000/5484/7973	0.248	0.263	0.259	0.153	0.95	0.723	1.02	0.01	0.10	0.00	0.02
rs9639521	7	25,830,647	156/766/1097	77/318/448	1148/5800/7519	0.267	0.280	0.280	0.089	0.94	0.990	1.00	0.02	0.19	0.01	0.11
rs1993094	7	25,845,010	28/474/1515	16/210/617	246/3237/10982	0.131	0.144	0.129	0.659	1.02	0.082	1.13	0.12	0.53	0.16	0.57
rs10226258	7	25,856,295	10/293/1714	6/119/717	119/2194/12152	0.078	0.078	0.084	0.166	0.92	0.370	0.92	0.05	0.64	0.07	0.68
rs7798431 *	7	25,860,812	106/718/1195	46/283/514	820/5326/8323	0.230	0.222	0.241	0.145	0.94	0.086	0.90	0.85	1	1.00	1
rs12535837	7	25,861,138	110/730/1172	48/288/505	836/5382/8247	0.236	0.228	0.244	0.281	0.96	0.147	0.92	0.85	1	1	1
rs10282436	7	25,873,110	61/626/1330	32/243/567	465/4428/9561	0.185	0.182	0.185	0.991	1.00	0.753	0.98	0.71	1	0.83	1
rs12700664	7	25,875,151	507/975/534	212/404/226	3476/7303/3662	0.493	0.492	0.494	0.976	1.00	0.881	0.99	0.30	1	0.25	1
rs10232819	7	25,876,612	116/733/1170	48/294/501	854/5399/8214	0.239	0.231	0.246	0.356	0.96	0.183	0.92	0.89	1	0.94	1
rs2290263	7	25,887,278	144/744/1123	61/317/459	873/5384/8182	0.257	0.262	0.247	0.183	1.05	0.157	1.08	0.04	0.51	0.12	1
rs12700667 *	7	25,901,639	123/747/1149	49/300/494	898/5527/8045	0.246	0.236	0.253	0.326	0.96	0.117	0.91	1.00	1	0.85	1
rs17152823	7	25,904,794	40/480/1489	19/202/618	281/3333/10854	0.139	0.143	0.135	0.410	1.04	0.329	1.07	0.04	1	0.04	1
rs3823944	7	25,904,853	433/996/556	179/411/238	3228/7295/3826	0.469	0.464	0.479	0.226	0.96	0.237	0.94	0.03	0.31	0.05	0.50
rs10235225	7	25,905,599	358/977/677	148/404/289	2516/7073/4843	0.421	0.416	0.419	0.871	1.01	0.795	0.99	0.15	0.87	0.12	0.85
rs10230872	7	25,908,210	175/833/1009	78/344/421	1344/6066/7038	0.293	0.297	0.303	0.210	0.95	0.580	0.97	0.01	0.27	0.04	0.53
rs1451386	7	25,909,208	19/316/1684	10/131/702	118/2325/12011	0.088	0.090	0.089	0.847	0.99	0.892	1.01	0.00	0.29	0.00	0.17
rs10272701	7	25,911,033	68/575/1375	28/232/582	472/4478/9518	0.176	0.171	0.187	0.084	0.93	0.092	0.89	0.00	0.01	0.00	0.07
rs7785415	7	25,911,327	24/379/1614	10/160/672	158/2617/11691	0.106	0.107	0.101	0.381	1.05	0.468	1.06	0.00	0.01	0.00	0.04
rs1868889	7	25,912,817	292/932/793	122/366/354	2054/6656/5757	0.376	0.362	0.372	0.644	1.02	0.423	0.96	0.07	0.64	0.05	0.60
rs1451389	7	25,914,286	210/905/904	100/388/355	1570/6327/6569	0.328	0.349	0.327	0.908	1.00	0.068	1.10	0.04	0.24	0.01	0.13
rs6461889	7	25,920,438	26/381/1611	12/162/669	152/2724/11588	0.107	0.110	0.105	0.612	1.03	0.461	1.06	0.03	1	0.03	1
rs4722536	7	25,921,083	204/903/912	92/395/356	1521/6333/6615	0.325	0.343	0.324	0.929	1.00	0.097	1.09	0.05	0.26	0.02	0.16
rs17152874	7	25,923,381	94/731/1192	49/319/474	769/5111/8581	0.228	0.248	0.230	0.768	0.99	0.093	1.10	0.06	0.25	0.02	0.15
rs6461892	7	25,930,573	33/411/1567	16/169/656	163/2903/11383	0.119	0.120	0.112	0.196	1.07	0.325	1.08	0.00	0.13	0.00	0.03
rs896312	7	25,935,030	194/916/899	87/399/351	1546/6367/6530	0.325	0.342	0.328	0.711	0.99	0.208	1.07	0.05	0.26	0.02	0.16
rs12672425	7	25,939,723	57/639/1310	26/274/536	498/4464/9504	0.188	0.195	0.189	0.875	0.99	0.523	1.04	0.10	0.41	0.10	0.38
rs7809764	7	25,945,652	264/945/808	109/397/336	1912/6674/5871	0.365	0.365	0.363	0.798	1.01	0.860	1.01	0.00	0.04	0.00	0.13
rs1966841	7	25,951,345	348/1017/647	146/409/283	2420/7000/5039	0.426	0.418	0.409	0.049	1.07	0.475	1.04	0.07	0.50	0.05	0.43
rs878655	7	25,955,056	5/173/1827	3/74/762	21/1193/13231	0.046	0.048	0.043	0.397	1.07	0.332	1.12	0.00	0.29	0.00	0.17
rs12154905	7	25,955,754	127/763/1126	55/324/464	989/5623/7841	0.252	0.257	0.263	0.146	0.95	0.615	0.97	0.09	0.35	0.09	0.33
rs10280475	7	25,963,672	181/850/982	76/357/409	1336/6108/6996	0.301	0.302	0.304	0.701	0.99	0.879	0.99	0.00	0.08	0.00	0.10
rs6972699	7	25,967,611	0/51/1967	0/20/823	1/302/14156	0.013	0.012	0.011	0.220	1.21	0.598	1.13	0.00	0.02	0.00	0.12
rs4722551	7	25,991,826	59/586/1351	33/232/567	370/3723/10237	0.176	0.179	0.156	8.67E-04	1.16	0.011	1.18	0.07	1	0.06	1
rs9639523	7	25,991,977	230/888/898	99/373/369	1757/6581/6114	0.334	0.340	0.349	0.062	0.94	0.413	0.96	0.01	0.13	0.02	0.18
rs4719841	7	25,997,536	318/972/726	127/412/302	2510/6945/5005	0.399	0.396	0.414	0.073	0.94	0.152	0.93	0.01	0.09	0.02	0.18
rs7786527	7	26,007,582	26/405/1563	12/181/644	227/3063/11081	0.115	0.123	0.122	0.161	0.93	0.991	1.00	0.00	0.13	0.00	0.02

SNPs listed in Table S4c are intergenic and located between NPVF and NFE2L3. OR represent the alleleic odds ratio and P values are calculated by the Trend method. All SNPs are in Hardy-Weinberg equlibrium (P>0.01). Pair-wise r^2 and D' between rs12700667 and rs7798431 and the SNPs listed in the table are calculated based on Hapmap3 data.

*) SNPs rs12700667 and rs7798431 were imputed using 2019 Endometriosis cases and 14471 population controls.
