# Supplementary material for: Genome-Wide Association Study Link Novel Loci to Endometriosis
Source: PLoS One. 2013 Mar 5;8(3):e58257. doi: 10.1371/journal.pone.0058257 (PMC3589333; doi:10.1371/journal.pone.0058257)
Supplement: Table S5 — a Severity of endometriosis is independent of the five top loci by logistic regression analysis. b Severity of endometriosis is independent of the top five loci by association analysis. (PDF) [file pone.0058257.s009.pdf]

Table S5a Severity of endometriosis is independent of the five top loci by logistic regression analysis.

SNPid	CHR	BP	Risk Allele	Category	OR	CI 95 %	Beta	SE	P
rs10917151	1	22,422,721	A	AA or AG or GG	1.07	[0.91-1.25]	0.068	0.080	0.396
rs6757804	2	151,635,832	C	CC or CT or TT	0.98	[0.86-1.11]	-0.020	0.065	0.762
rs6907340	6	19,803,768	T	TT or TC or CC	1.08	[0.95-1.23]	0.078	0.064	0.220
rs10975519	9	6,253,571	T	TT or TC or CC	1.00	[0.87-1.14]	-0.004	0.069	0.952
rs10508881	10	44,541,565	A	AA or AG or GG	1.04	[0.92-1.18]	0.039	0.064	0.544

Patients with Mild endometriosis (n=1177) were compared to patients with Moderate or Severe endometriosis (n=842) in the logistic regression analysis to determine if any of the five loci correlate with severity. The results show that severity does not depend on any of the tested loci. P-values (P) are calculated using Wald test. Beta is the regression coefficients and SE the standard error from logistic regression.

Table S5b Severity of endometriosis is independent of the top five loci by association analysis.

SNPid	CHR	BP	MAF Controls	MAF endo All	P endo All	OR endo All [95%CI]	MAF endo Sev	P endo Sev	OR endo Sev [95% CI]
rs10917151	1	22,422,721	0.156	0.193	1.54E-09	1.30[1.19-1.41]	0.199	2.39E-06	1.34[1.19-1.52]
rs6757804	2	151,635,832	0.401	0.446	4.05E-08	1.20[1.13-1.29]	0.445	4.72E-04	1.20[1.08-1.32]
rs6907340	6	19,803,768	0.372	0.415	1.25E-07	1.20[1.12-1.28]	0.425	3.97E-06	1.24[1.13-1.37]
rs10975519	9	6,253,571	0.300	0.338	1.01E-06	1.17[1.09-1.25]	0.333	1.72E-03	1.16[1.05-1.29]
rs10508881	10	44,541,565	0.403	0.442	1.57E-06	1.18[1.10-1.26]	0.448	2.47E-04	1.21[1.09-1.33]

All endometriosis patients (n=2019) and patients with Moderate or Severe endometriosis (n=842) were analysed against 14,471 population controls by association analysis to determine if any of the five loci correlate with severity. The results show that the minor allele frequency in are almost identical in the two analyses suggesting minimal to no stratification. P-values (P) are calculated using Trend test in PLINK.
