# Supplementary material for: Genome-Wide Association Study Link Novel Loci to Endometriosis
Source: PLoS One. 2013 Mar 5;8(3):e58257. doi: 10.1371/journal.pone.0058257 (PMC3589333; doi:10.1371/journal.pone.0058257)
Supplement: Table S6 — Pair-wise autosomal genetic distance among ethnic groups as measured by the Fixation Index ( F ST). (PDF) [file pone.0058257.s010.pdf]

Table S6 Fixation Index (F_{ST}) divergences between estimated populations

	Europe Central	Europe South-East	Europe North-East	Europe North-West	Italy	Europe South-West	Germany	African
Europe South East	0.009							
Europe North East	0.01	0.014						
Europe North West	0.004	0.011	0.01					
Italy	0.007	0.009	0.016	0.011				
Europe south West	0.004	0.01	0.013	0.006	0.006			
Germany	0.008	0.014	0.013	0.007	0.014	0.011		
African	0.169	0.169	0.176	0.172	0.16	0.157	0.175	
Asian	0.12	0.119	0.121	0.12	0.119	0.118	0.124	0.208

F_{ST} is a measure that indicate how well populations can be distinguished (Holsinger et al. 2009). A value of 0 indicate that no distinction is possible while 1 indicate complete distinction. The Table shows minimal separation within the European populations, while intercontinental populations are easily distinguished. Genetic distances were calculated based on 33,067 autosomal SNPs derived from ethnically defined samples from POPRES (Nelson et at. 2008) and internal studies (data not shown).
