# Supplementary material for: Genome-Wide Association Study Link Novel Loci to Endometriosis
Source: PLoS One. 2013 Mar 5;8(3):e58257. doi: 10.1371/journal.pone.0058257 (PMC3589333; doi:10.1371/journal.pone.0058257)
Supplement: Table S7 — Support for endometriosis association in a Caucasian cohort found at 3 of 7 loci reported by Nyholt et al. (PDF) [file pone.0058257.s011.pdf]

Table S7 Support for endometriosis association in a Caucasian cohort found at 3 of 7 loci reported by Nyholt et al.

Nyholt study	Chr	Pos	LD proxy SNP	r2 Pair-wise	D' Pair-wise	MAF controls	MAF all	P all	OR all [95%CI]	MAF sev	P sev	OR sev [95% CI]	Support for association
rs7521902[a)]	1	22,490,724	rs12042083	0.66	0.93	0.200	0.228	4.52E-05	1.18 [1.09-1.28]	0.230	2.40E-03	1.20 [1.07-1.35]	+
rs13394619[b)]	2	11,727,507	rs2358040	1	1	0.486	0.473	0.11	0.95 [0.89-1.01]	0.470	0.21	0.94 [0.85-1.04]	-
rs4141819[c)]	2	67,864,675	*na*	*na*	*na*	0.313	0.310	0.68	0.98 [0.92-1.06]	0.324	0.36	1.05 [0.95-1.17]	-
rs7739264[d)]	6	19,785,588	*na*	*na*	*na*	0.475	0.450	3.52E-03	0.91 [0.85-0.97]	0.441	6.12E-03	0.87 [0.79-0.96]	+
rs12700667[e)]	7	25,901,639	rs12535837	0.85	1	0.244	0.236	0.28	0.96 [0.89-1.04]	0.228	0.15	0.92 [0.82-1.03]	-
rs1537377[f)]	9	22,169,700	rs17694933	0.92	1	0.422	0.435	0.11	1.06 [0.99-1.13]	0.444	0.08	1.09 [0.99-1.20]	-
rs10859871[g)]	12	95,711,876	rs12298029	1	1	0.303	0.330	4.49E-04	1.14 [1.06-1.22]	0.344	3.84E-04	1.21 [1.09-1.34]	+

The table compares the seven significantly associated SNPs ($P < 5x10^{-8}$) reported by Nyholt et al. to results from the present study. Due to the different SNP sets considered in the two studies, a direct comparison is only possible for rs4141819 and rs7739264. Based on the Hapmap3 data we identified two SNPs in our data that are in perfect linkage disequlibrium with rs13394619 and rs10859871 respectively, and for the reaining three SNPs (rs7521902, rs12700667 and rs1537377) we selected the SNP from our study with the strongest pair-wise LD.

The association analysis was performed with all endometriosis cases (all, n=2019), and with moderately and severily affected endometriosis cases (sev, n=842), against 14,471 population controls.

[a)] rs7521902 is not present in our study but show moderate LD with rs12042083 in our study. rs12042083 show support for association in our study but we report rs2235529 to be more strongly associated.

[b)] rs13394619 is not pressent in our study but is in perfect LD with rs2358040. rs2358040 does not show assocan in our study.

[c)] rs4141819 is present in our study but does not show association.

[d)] rs7739264 is present in our study and does show association but we report rs6907340 to be more strongly associated.

[e)] rs12700667 is not present in our study but show strong LD with rs12535837 in our study. rs12535837 does not show association in our study.

[f)] rs1537377 is not present in our study but show strong LD with rs17694933 in our study. rs17694933 does not show association in our study.

[g)] rs10859871 is not present in our study but is in perfect LD with rs12298029. rs12298029 show support for association in our study but we report rs3596 to be more strongly associated.
